# dna2bit: high performance genomic distance estimation software for microbial genome analysis

**DOI:** 10.3389/fmicb.2024.1521181

**Published:** 2024-12-23

**Authors:** Juzeng Li, Yuxin Tian, Yi Wang, Li Jin

**Affiliations:** ^1^Ministry of Education Key Laboratory of Contemporary Anthropology, Department of Anthropology and Human Genetics, School of Life Sciences, Fudan University, Shanghai, China; ^2^State Key Laboratory of Genetic Engineering, Collaborative Innovation Center for Genetics and Development, School of Life Sciences and Human Phenome Institute, Fudan University, Shanghai, China; ^3^Research Unit of Dissecting the Population Genetics and Developing New Technologies for Treatment and Prevention of Skin Phenotypes and Dermatological Diseases (2019RU058), Chinese Academy of Medical Sciences, Beijing, China

**Keywords:** Hamming distance, average nucleotide identity, genome distance, metagenomic clustering, single amplified genomes

## Abstract

dna2bit is an ultra-fast software specifically engineered for microbial genome analysis, particularly adept at calculating genome distances within metagenome and single amplified genome datasets. Distinguished from existing software such as Mash and Dashing, dna2bit employs feature hashing technique and Hamming distance to achieve enhanced speed and memory utilization, without sacrifice in the accuracy of average nucleotide identity calculations. dna2bit has promising applications in various domains such as average nucleotide identity approximation, metagenomic sequence clustering, and homology querying. dna2bit significantly boosts computational efficiency in handling large datasets including single amplified genomes, thereby facilitating a better understanding of the population heterogeneity and comparative genomics of microorganisms. dna2bit is available at https://github.com/lijuzeng/dna2bit.

## Background

Metagenome analysis has emerged as a standard approach for the examination of microbial genomes. Initiatives such as Global Oceanographic Survey (GOS), Animal Metagenome Database, and MetaHIT Project have established comprehensive databases that reach terabyte scale. These databases recorded 16S rRNA and whole genome sequences from various environments including oceans across different latitude, animal tissues, and human intestines (Blanco-Miguez et al., 2017; Biller et al., 2018; Hu et al., 2022). The fundamental problem of downstream analysis is to determine the species, relative abundance and functional genes associated with each microorganism.

Despite metagenome analysis at macro level, whole genome amplification (WGA) techniques have been widely applied for the detection of picogram-level DNA in the environment. Uncultivable environmental DNA, also known as “microbial dark matter” cannot be detected by traditional cultivation methods, while WGA method including multi-displacement amplification (MDA) has facilitated the sequencing of single amplified genomes (SAGs) at the single-cell level (Maruyama et al., 2017). SAG data enables detailed research on population heterogeneity and gene expression of individual uncultured microorganisms (Wiegand et al., 2021). Due to partitioning of single cells and extensive parallel sequencing, number of SAG genomes present in a sample significantly exceeds that of traditional microbial genomes. Subsequent data analysis including clustering and co-assembly demands much higher throughput and efficiency (Hosokawa et al., 2017).

The computation of microbial genomic distances across varying assembly qualities has been emphasized in comparative genomics, metagenomic analysis and the co-assembly of SAGs (Wang et al., 2021). Existing methods for distance calculation categorized into two approaches: alignment-dependent and alignment-free methods. The alignment-dependent strategy focuses on determining the average nucleotide identity (ANI) through the simulation of DNA–DNA hybridization. ANI serves as a robust measure of overall similarity within homologous regions of two microbial genomes. On the other hand, alignment-free methods are regarded as a more efficient approach for handling large-scale genomic data. Drawing inspiration from text clustering algorithms in computer science, these methods effectively compress large genomes into more manageable sketches, thereby facilitating the estimation of Jaccard similarity in reduced representations (Zielezinski et al., 2017). The Jaccard similarity index can be obtained in fewer calculation steps and provides a practical approximation of ANI (Arahal, 2014).

The introduction of Mash by Ondov et al. (2016) marked a significant milestone in alignment-free genomic analysis. By employing MinHash to generate genomic sketches, Mash has become the most preeminent software for rapid and efficient genome comparison (Ondov et al., 2016). Spurred by Mash, FastANI has further established a more refined approximation of ANI, facilitating a more streamlined and accurate assessment across diverse prokaryotic genomes (Jain et al., 2018).

The remarkable success of Mash has sparked the development of analogous software. For instance, Dashing has introduced HyperLogLog sketches as a viable alternative to MinHash, effectively overcoming the challenges associated with disparate genome sizes (Baker and Langmead, 2019). BinDash leverages advancements in MinHash such as binary rolling hash technique to accelerate distance computation in large genomes (Zhao, 2019). Both Mash and its subsequent enhancements employ probabilistic data structures to achieve dimensionality reduction, which is accomplished through the creation of sketch representations for the original genomes. Similar algorithms have found widespread application in bioinformatics. SpaceSeed has been deployed for the classification of metagenomic data (Luo et al., 2019), while bloom filters have been utilized in assembly of genomes (Jackman et al., 2017).

Probabilistic data structures are suitable for intersection and union operations, which are essential in computing a range of similarity indices, including Jaccard index, simple matching coefficient, Sorensen–Dice coefficient, Salton’s cosine index, and overlap coefficient (Verma and Aggarwal, 2020). In scenarios that necessitate frequent computation of distances, reliance on intersection and union for similarity assessments presents significant computational challenges. Given the explosive growth of single-cell sequencing files, such situation is becoming increasingly common.

Beyond utilizing intersection and union for similarity measurement, researchers have explored alternative metrics such as cosine distance, Euclidean distance and Hamming distance (Singh et al., 2020). Dong et al. (2019) have developed an Euclidean-distance approach to characterize the distribution and covariance of nucleotides across genomes, enabling the efficient construction of phylogenetic trees. Zamora et al. (2016) have implemented a low-dimensional Hamming embedding technique to approximate pairwise similarity matrices in clustering tasks, which eliminates the need for extensive data storage and achieves better runtime and lower memory usage.

Our strategy incorporates Hamming distance to avoid frequent intersections and unions in distance calculation. This approach draws inspiration from Google’s SimHash, a robust and well-established technique originally implemented for text deduplication (Sadowski and Levin, 2007). In comparison to MinHash, SimHash has shown superior performance in processing voluminous text data while maintaining a comparable level of accuracy (Uddin et al., 2011). The application of SimHash in bioinformatics has shown promising outcomes as well. For instance, Firtina et al. (2023) have developed BLEND to manage read coverage in genome assembly task, demonstrating notable advantages in both speed and memory efficiency.

Similar to existing software like Mash, dna2bit employs a two-stage approach including sketch and distance calculation. During sketch stage, the input nucleotide sequences undergo dimensionality reduction through the feature hashing technique (Weinberger et al., 2009), converting genomic *k*-mers into a compact bit array representation. Subsequently, the Hamming metric will be applied to calculate pairwise distances between these bit arrays. As an open-source software with MIT license, dna2bit is readily available for download at https://github.com/lijuzeng/dna2bit, facilitating its integration into various bioinformatics pipelines.

## Results

### Hyperparameter screening for optimal prediction of ANI

Our initial investigation focuses on clarifying the correlation between distances computed by dna2bit and ANI derived from alignment-based software MUMmer4 (Marcais et al., 2018). This relationship is quantified by the Pearson correlation coefficient between dna2bit distance and 1-ANI. We further examine the impact of different hyperparameters on the performance of dna2bit. Specifically, we consider the length of the bit array referred to as sketch size (denoted as *l*), and the length of the *k*-mer (denoted as *k*).

**FIGURE 1:**
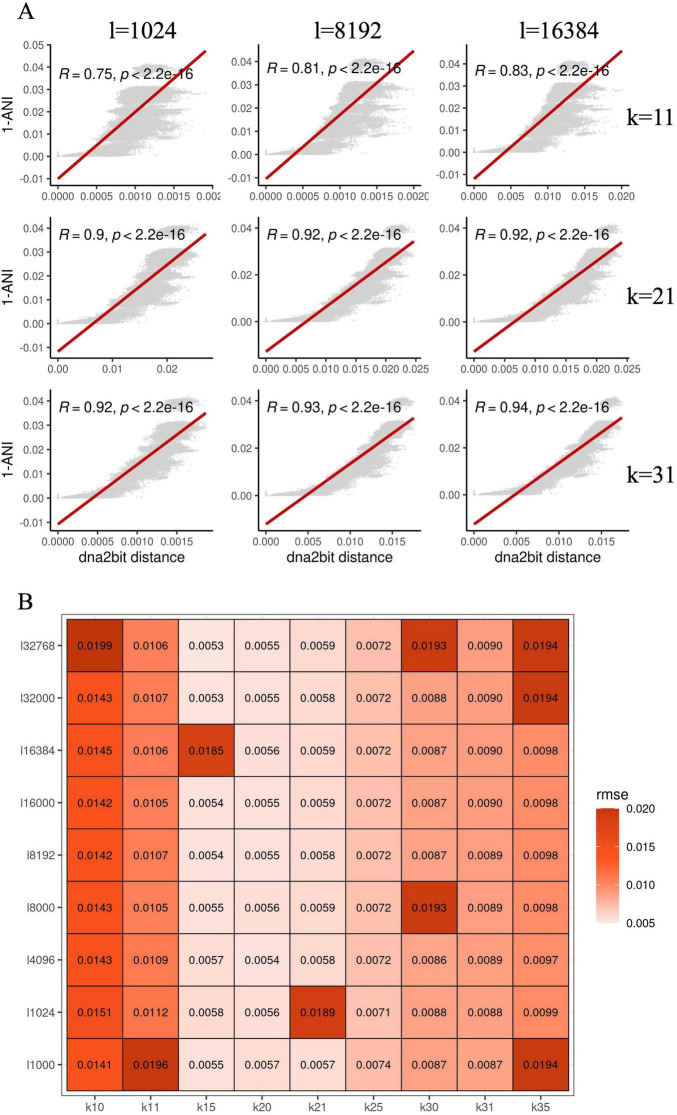
Hyperparameter optimization for dna2bit using *Escherichia coli* genomes: **(A)** Scatter plots show the relationship between 1-ANI and the distances computed by dna2bit, with the red line indicating the linear regression model’s prediction. The *x*-axis varies the bit array length (**l**), while the *y*-axis alters the *k*-mer size (**k**). **(B)** The heatmap presents the root mean square error (RMSE) between the dna2bit distances and 1-ANI, indicating the impact of different hyperparameter configurations.

To empirically assess this correlation, we randomly extracted a subset of 500 *Escherichia coli* genomes from the NCBI RefSeq database for distance calculation. For dna2bit, we set a range of *k*-mer lengths and sketch sizes. The Pearson correlation coefficients under various settings are illustrated in Figure 1A.

The heatmap indicates that as the values of *k* and *l* increase, there is a notable enhancement in the Pearson coefficient, suggesting a better correlation between dna2bit distance and 1-ANI. However, larger *k* and *l* results in an escalation in computational requirements for distance computation. Consequently, we screened the optimal combination of sketch size and *k*-mer length for subsequent analysis. Optimality was determined by minimizing the root mean square error (RMSE) while balancing computational expense. We explored an extended range of values for both *k* and *l*. The heatmap in Figure 1B illustrates RMSE results calculated by dna2bit.

Observation reveals that the RMSE values are relatively low for *k*-mer lengths between 15 and 21. Detailed outcomes for each *k*-mer length and sketch size are thoroughly documented in Supplementary Figure 1. Considering the collective findings from Figures 1A, B and Supplementary Figure 1, we have ascertained the default hyperparameters for dna2bit to be *bit*_*array*_*length* = 8,192 and *k*_*mer* = 17. These choices achieve an optimal balance between computational speed and commendable precision in genomic distance estimations.

### Clustering analysis on metagenomes and single-cell amplified genomes

**FIGURE 2:**
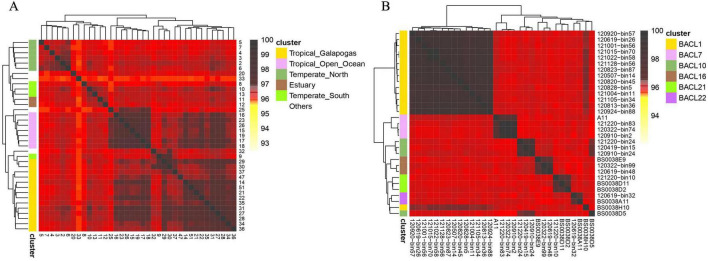
Hierarchical clustering of metagenomes and single-cell amplified genomes (SAGs) facilitated by dna2bit. **(A)** Metagenomes from the Global Oceanographic Survey (GOS) dataset. **(B)** SAGs from the Linnaeus Microbial Observatory (LMO) dataset. Pairwise distances for samples are computed using dna2bit and subsequently utilized for hierarchical clustering analysis.

Leveraging the robust correlation between bit distance and 1-ANI, we have conceptualized a distance-based genome clustering task. The most typical scenario for clustering involves metagenomes, and the clustering of single-cell amplified genomes (SAGs) has emerged as a hot topic in recent years in microbiomes. We downloaded the GOS dataset for our analysis (Biller et al., 2018). GOS study was designed to examine different microbial populations which are sampled across marine with various latitudes and temperatures. Our metagenome dataset encompasses 37 unique samples from the GOS study. Following the computation of pairwise distances using dna2bit, we employed the hclust package in R to perform hierarchical clustering. In alignment with the clustering labels from Mash’s work, we illustrated clustering results that correspond to six types of marine sampling sites as a heatmap in Figure 2A.

Adjacent to the coordinate axis, the hierarchical tree diagram indicates clear distinctions among samples collected from six sites. Essentially, the “Others” category (represented in white) segregates the remaining groups. Within the colored groups, only sample 9 from the “Temperate South” group is incorrectly categorized with the “Tropical Galapagos” group.

Furthermore, we obtained the SAG dataset from the Linnaeus Microbial Observatory (LMO). In the LMO study, Alneberg et al. (2018) collected 81 metagenomes (MAGs) and 17 SAGs to perform a seasonal analysis of microbial populations in the Baltic Sea. We conducted a re-clustering analysis of seven clusters which included SAG genomes. Our dataset included a total of 31 genomes, comprising 24 MAGs and 7 SAGs. The clusters were labeled following the BACL numbering system proposed by Hugerth et al. (2015). The results of the hierarchical clustering based on dna2bit distance are illustrated in Figure 2B.

dna2bit successfully separated BACL clusters that included MAGs, while there was a misclassification for the SAG samples BS0038H10 and BS0038D5. The actual BACL groupings for these samples are BACL 1 for BS0038H10 and BACL 10 for BS0038D5, corresponding to the γ-*Proteobacteria* and *Rhodobacter*, respectively. Incorrect samples may contain analogous genes responsible for the breakdown of organic carbon. Because both γ-*Proteobacteria* and *Rhodobacter* are the predominant marine microorganisms known to suppress the proliferation of dinoflagellates.

Beyond the challenge of overlapping gene sequences, the limitations inherent in the distance representation with a single parameter setting may also lead to misclassification. To enhance the performance in these edge cases, various configurations of the k and l parameters can be integrated to generate a regularized distance estimation for clustering process, thereby improving the accuracy and reliability of the classification outcomes.

### Computational efficiency and accuracy among different software

**FIGURE 3:**
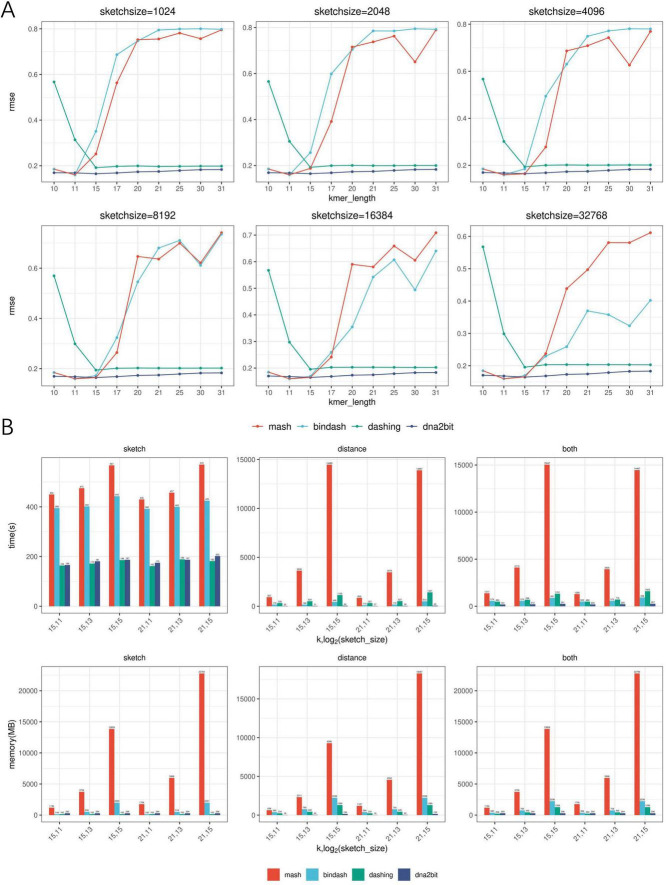
Assessment of dna2bit’s performance in accuracy and computational efficiency compared to Mash, BinDash, and Dashing across diverse hyperparameter configurations. **(A)** Line plots show the root mean square error (RMSE) for the four methods. **(B)** Histograms show the efficiency in terms of runtime and memory usage during the sketch and distance stages, as well as the overall process (combining both sketch and distance stages) for the four methods.

We compare the computational efficiency of dna2bit and existing software. Commonly used software such as Mash, BinDash, and Dashing (Ondov et al., 2016; Baker and Langmead, 2019; Zhao, 2019) were selected as the baseline. Each software was equipped with 10 threads and tested across a variety of *k*-mer length settings. However, the concept of sketch size varies between dna2bit and other software, resulting in differences in the actual sketch sizes used. We downloaded 37,004 genomes from the NCBI RefSeq database for evaluation, adhering to specific inclusion criteria: prokaryotic, latest, complete genome without any “contigs” notation. Detailed information about the dataset is provided in Supplementary material. Figure 3B presents the running records for each software. Metric of runtime and memory usage utilized in the experiment are the execution time and the peak memory usage, quantified as the elapsed (wall clock) time in seconds and maximum resident set size (RSS) in megabytes (MB).

The histogram analysis indicates that dna2bit achieves the shortest overall runtime, which is 6.4–59.9 times faster than Mash, 2.5–3.5 times faster than BinDash, and 2.4–6.0 times faster than Dashing. During the sketch stage, dna2bit outperforms both Mash and BinDash in runtime as well, while being slightly slower than Dashing. In terms of memory utilization, dna2bit shows the lowest RAM usage at the distance stage. As the sketch size increased, memory efficiency for dna2bit becomes increasingly evident. At the sketch stage, the RAM usage of dna2bit (294 MB) remains relatively stable regardless of sketch size, with a modest increase compared to Dashing (162 MB). Conversely, Mash and BinDash demonstrate increased RAM requirements across all configured sketch sizes (Mash: 1,191–22,748 MB, BinDash: 149–2,001 MB). Detailed runtime and RAM usage data are provided in Supplementary Tables 1–6.

To evaluate the accuracy of ANI estimations, we conducted a comparative analysis across different software. Similar to the hyperparameter screening under varying *k*-mer lengths and sketch sizes, we computed the RMSE between distances predicted by each software and MUMmer4-based 1-ANI. We randomly selected 100 genomes from above prokaryotic dataset and performed the calculations three times to ensure reliability. Figure 3A displays the results from one of these trials, while the remaining two are provided in Supplementary Figures 2, 3.

The line chart analysis reveals that dna2bit exhibits a lower RMSE value across a range of hyperparameter settings, which corroborates the robust correlation observed between bit distance and ANI again. dna2bit maintains a stable RMSE level despite changes in sketch size, whereas Mash and BinDash show significant variation. This can be attributed to the fact that Mash and BinDash incorrectly report distance value as k increased, which adversely affects the precision of the ANI calculations.

When estimating Jaccard similarity with a large *k* value, the likelihood of *k*-mer overlap may diminish due to uniqueness increases. MinHash-based Mash and BinDash methods tend to incorrectly estimate the cardinality of overlapping *k*-mers as zero, thereby misjudging distance as one and resulting in RMSE errors regardless of genetic relationship. Dashing does not exhibit this issue, but it also encounters estimation errors at lower *k* values due to high probability of short *k*-mer coincidences.

In contrast to these methods, dna2bit employs Hamming distance instead of Jaccard index for estimating ANI. Given that this methodology is independent of the *k*-mer coincidence probability, a factor known to be sensitive to parameter k, we hypothesize that employing Hamming distance can substantially avoid the impact of *k* value selection on the ANI estimation error. Consequently, dna2bit demonstrates commendable consistency and robustness across a broad range of *k* values.

## Methods

### dna2bit sketch

**FIGURE 4:**
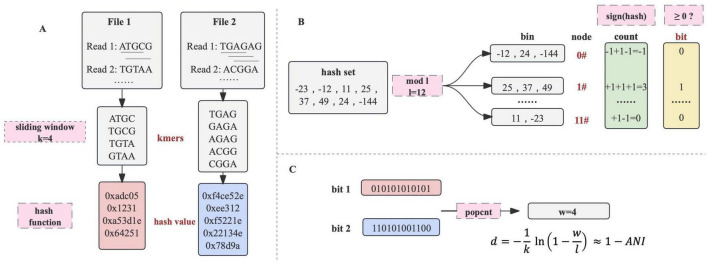
The principle of dna2bit. **(A)** Sequence files are processed using a sliding window to generate *k*-mers. Each *k*-mer is subsequently hashed into a 64-bit hash value through a hash function. **(B)** Hash values are allocated into bins associated with unique node by the modulo operation. A node’s bit is determined by the cumulative summation and binary projection with non-negative sums indicating “1.” **(C)** The bit arrays from two sequences undergo eXclusive OR (XOR) operation, followed by a population count (popcount) procedure. The dna2bit distance is subsequently computed based on a predefined formula.

In order to convert sequencing files into bit arrays, both the primary nucleotide sequences and their reverse complements are processed by a sliding window of length *k* to extract *k*-mers. Subsequently, each *k*-mer undergoes hashing by a specific hash function to generate a 64-bit hash value (Figure 4A).

Motivated by the principle of feature hashing, the hash values associated with *k*-mers are then allocated to bins of a fixed number (denoted by *l*) through the modulo operation. The term “node” is employed to describe the identifier suffixed to each bin. Consequently, every *k*-mer is mapped to a specific node via the hash value modulo the total count of bins. This modulo operation enables a consistent and uniform classification of *k*-mers, effectively reducing the dimensionality of the feature space (Attenberg et al., 2009).

To effectively represent the extensive collection of *k*-mers within a specific bin, we implemented a binary-projection approach inspired by the Simhash algorithm (Henzinger, 2006). Within the scope of dna2bit, the hash values for each bin are aggregated based on a sign function:


(1) $$s\,i\,g\,n\,\left(h\,a\,s\,h\right)=\left\{ \begin{array}{c} 1,i\,f\,s\,i\,g\,n\,b\,i\,t=1\\ -1,i\,f\,s\,i\,g\,n\,b\,i\,t=0 \end{array}\right.$$

Accumulated count of sign function will subsequently be mapped onto a single bit:


(2) $$b\,i\,t\,\_\,a\,r\,r\,a\,y_{\left[n\,o\,d\,e\right]}=\left\{ \begin{array}{c} \;1,i\,f\,\sum 2\times s\,i\,g\,n\,\left(h\,a\,s\,h\right)-1\geq 0\\ 0,i\,f\,\sum 2\times s\,i\,g\,n\,\left(h\,a\,s\,h\right)-1<0 \end{array}\right.$$

Through the processing of each node, a sequence is sketched into a binary array of a specified length *l* (Figure 4B). Following the concept of “approximate fingerprint” of a file in Simhash, dna2bit creates an “approximate genetic fingerprint” for nucleotide sequences. In subsequent analysis, dna2bit focuses on performing various operations solely on these fingerprints, which occupy minimal memory space and facilitate swift computations.

### dna2bit distance

Hamming distance was introduced as a measure to quantify the difference between two bit arrays. This metric is widely recognized for its ability to assess arrays of identical length by counting their different positions (Jarrous and Pinkas, 2009). Computation of the Hamming distance between bit arrays is conducted through the following procedure:


(3) $$\delta \,\left(a_{i},b_{i}\right)=\left\{ \begin{array}{c} 1,i\,f\,a_{i}\neq b_{i}\\ 0,i\,f\,a_{i}=b_{i} \end{array}\right.$$

(4) $$w\,\left(A,B\right)=\sum_{i=1}^{l}\delta \,\left(a_{i},b_{i}\right)$$

Here, “A” and “B” are used to represent two different bit arrays, with “a” and “b” representing the bit values at each position. Computation of Equations 3, 4 can be efficiently executed using bitwise eXclusive OR (XOR) operations and the population count (popcount) function in C++ (Figure 4C). Many processor architectures including x86 and ARM provide instructions for executing XOR and popcount operations, enhancing the efficiency at the hardware level by parallel processing of multiple bits.

Although Hamming distance efficiently highlights distinctions between binary arrays, it does not exhibit a strong correlation with the ANI of the original genomes. To establish a more biologically relevant distance metric, we refer to the research conducted by Fan et al. on the nucleotide substitution model. Under the assumption that the mutation frequency of *k*-mer follows a Poisson distribution with parameter λ = *kd*, the probability of no mutations occurring within a *k*-mer is given by *e*^−*kd*^ (Fan et al., 2015). If we further assume that the Hamming distance (denoted as *w*) is related to this probability, we can expect that the ratio of *w* to the length of the bit array (*l*) serves as an estimation, i.e., $e^{-k\,d}=1-\frac{w}{l}$. By solving the equation, we derive the following formula:


(5) $$d\,\left(A,B\right)=-\frac{1}{k}\,\ln\left(1-\frac{w}{l}\right)$$

Through the pairwise computation of bit arrays, a distance matrix can be derived. dna2bit offers two output formats for subsequent analysis: an upper triangular matrix and a symmetric matrix.

### Hash function

dna2bit provides users with the flexibility to select from three different hash functions: wyhash, nthash, and Murmurhash3, each with its unique characteristics and applications.

wyhash is a hash function developed by our team with robust performance. It has successfully passed various stringent tests, including SMHasher, BigCrush, and practrand. wyhash is engineered to operate effectively on both 64-bit and 32-bit systems, making it versatile for different computational environments. The source code for wyhash is available at our GitHub repository: https://github.com/wangyi-fudan/wyhash.

nthash is a hash function introduced by Mohamadi et al. (2016). It employs a rolling hashing algorithm specifically designed for processing nucleotide sequences such as adjacent *k*-mers (Mohamadi et al., 2016).

Murmurhash3 is a widely recognized hash function established by Austin Appleby. Both Google and Mash have integrated it into the MinHash algorithm. Murmurhash3 is known for its non-cryptographic properties and its performance, especially in terms of speed and collision resistance.

### dna2bit benchmarking

dna2bit leverages a variety of well-established packages to enhance its functionality and performance. kseq is utilized for reading input files, providing compatibility with both FASTA and FASTQ formats. zlib is employed for reading compressed (.gz) files. OpenMP is a programming framework to execute multi-threading operations across multiple processor cores, which significantly improves computational efficiency. nthash and Murmurhash3 are the candidate hash functions. dna2bit is written in C++11. Software testing task was carried out on a PC equipped with an Intel Core i7-11700 CPU, featuring 16 threads and 32 GB of memory.

## Conclusion

dna2bit presents a computationally efficient approach for the large-scale estimation of genomic distances, which is especially important for the scenes that need to deal with a large amount of data in metagenomics and single amplified genome analysis. In contrast to existing tools such as Mash, BinDash, and Dashing, the adoption of a binary fingerprint by dna2bit significantly accelerates the computation of distances while concurrently reducing memory consumption. The Hamming-based bit distance calculated by dna2bit retains a robust correlation with established metrics, including ANI as well as the Jaccard index. In clustering analysis involving metagenomics and SAGs, dna2bit exhibits exemplary performance, equipping researchers with a dependable and rapid tool for the analysis of microbial populations.

In the future, dna2bit holds considerable promise for further integration into bioinformatics workflows related to comparative genomics. Through ongoing advancements in parallel processing and computational algorithm optimization, dna2bit emerges as a critical tool in DNA assembly and real-time genomic diagnostics. The further development of dna2bit is anticipated to significantly contribute to large-scale microbial genomes clustering and abundance estimation in environmental microbiology, driving innovation and discovery in microbial genomics

## Data Availability

The source code for dna2bit is publicly accessible on GitHub at the following repository: https://github.com/lijuzeng/dna2bit. The Genome Assembly accession number (GCA) corresponding to Figure 1 and Supplementary Figure 1 are in Data Sheet 2. The metagenomes and SAGs utilized in this study originate from the research conducted by Biller et al. (2018) and Alneberg et al. (2018). The GCA numbers associated with Figure 3 and Supplementary Figures 2, 3 are in Data Sheet 3.
