# Supplementary material for: dna2bit: high performance genomic distance estimation software for microbial genome analysis
Source: Front Microbiol. 2024 Dec 23;15:1521181. doi: 10.3389/fmicb.2024.1521181 (PMC11701053; doi:10.3389/fmicb.2024.1521181)
Supplement: Supplementary file 1 [file Data_Sheet_1.pdf]

Supplement for **dna2bit: High Performance Genomic Distance Estimation Software for Microbial Genome Analysis**

Supplementary figures

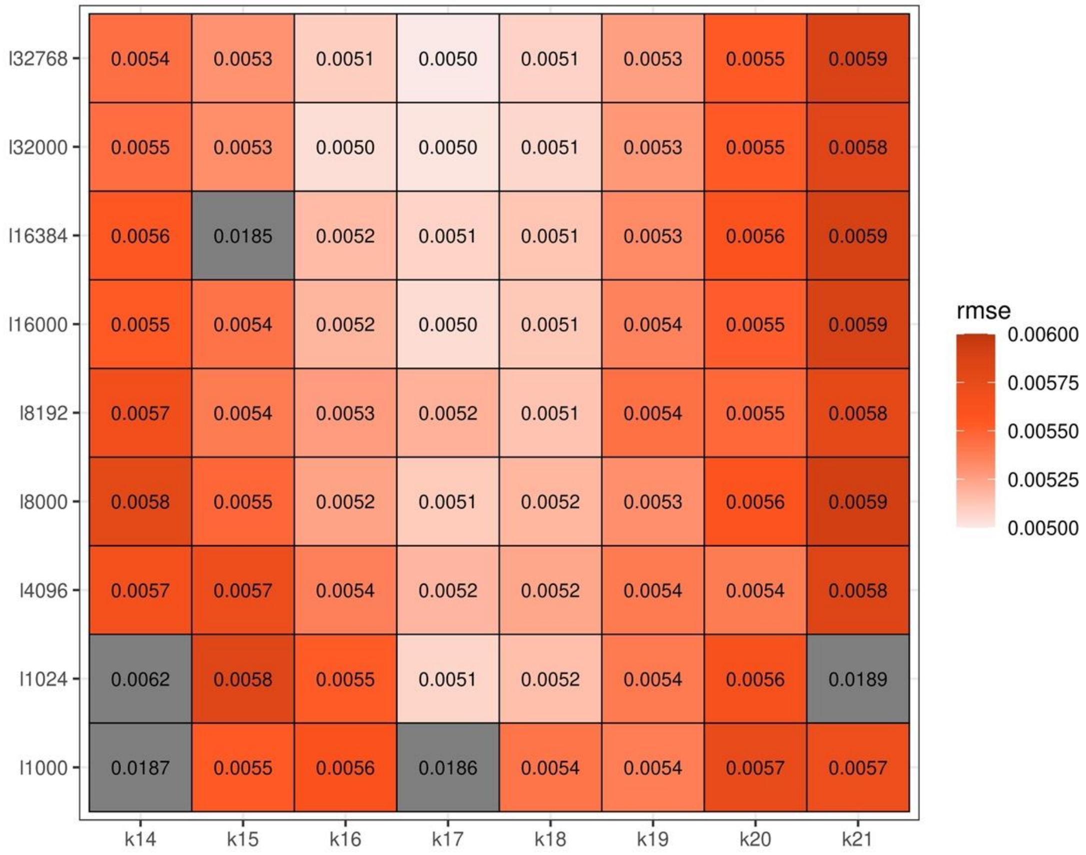

Fig S1 : The heatmap shows RMSE of dna2bit distance and ANI under the k-mer size range from 14 to 21. In order to find the best setting of dna2bit, we expanded multiple k-mer sizes between 15-21, and find that when k-mer size = 17, dna2bit has the best accuracy.

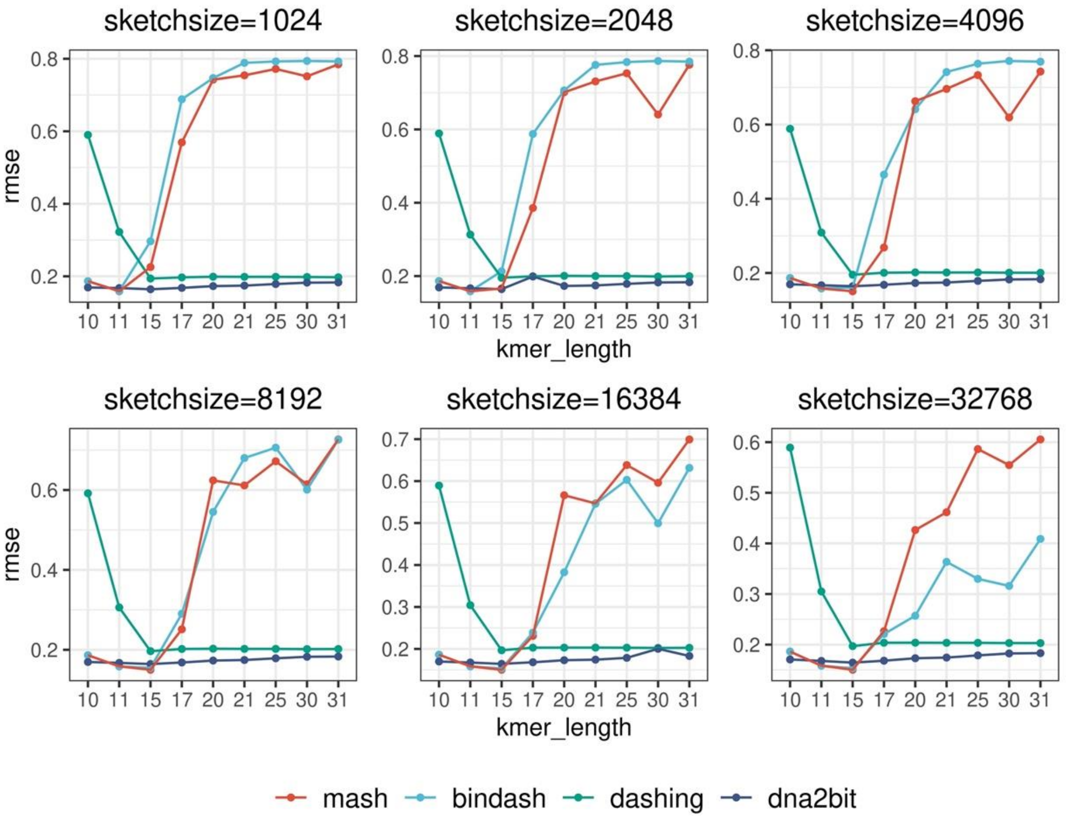

Fig S2 : Line plots show the root mead square error (RMSE) of four methods under different hyperparameter settings. These 100 data are randomly selected from the 37004 prokaryotic datasets.

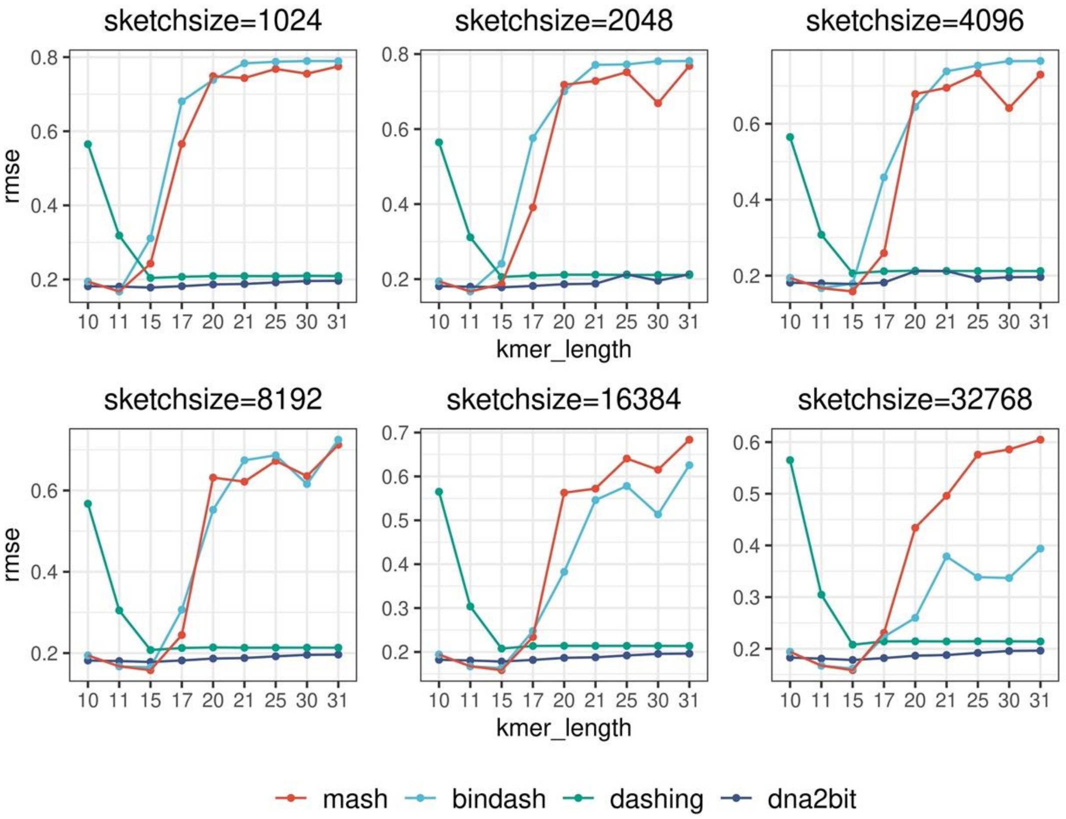

Fig S3 : Line plots show the root mead square error (RMSE) of four methods under different hyperparameter settings. These 100 data are randomly selected from the 37004 prokaryotic datasets.

Supplementary tables

Table 1. Runtime (s) during sketch stage for four methods

k,s	mash	bindash	dashing	dna2bit
15,11	450	395	164	166
15,13	475	402	172	181
15,15	567	443	186	187
21,11	430	392	162	175
21,13	457	400	189	187
21,15	570	425	182	202

Table 2. Runtime (s) during distance stage for four methods

k,s	mash	bindash	dashing	dna2bit
15,11	927	179	330	25
15,13	3635	168	524	31
15,15	14480	448	1145	64
21,11	859	110	327	27
21,13	3478	172	527	38
21,15	13897	513	1421	65

Table 3. Runtime (s) overall process for four methods

k,s	mash	bindash	dashing	dna2bit
15,11	1377	574	494	191
15,13	4110	570	696	212
15,15	15047	891	1331	251
21,11	1289	502	489	202
21,13	3935	572	716	225
21,15	14467	938	1603	267

Table 4. Memory usage (Mb) during sketch stage for four methods

k,s	mash	bindash	dashing	dna2bit
15,11	1194	149	162	293
15,13	3736	520	162	294
15,15	13859	2000	162	296
21,11	1756	150	162	294
21,13	5968	519	162	294
21,15	22784	2001	162	296

Table 5. Memory usage (Mb) during distance stage for four methods

k,s	mash	bindash	dashing	dna2bit
15,11	638	385	234	18
15,13	2311	760	445	44
15,15	9286	2238	1289	150
21,11	1197	384	234	18
21,13	4552	754	445	44
21,15	18287	2238	1289	150

Table 6. Memory usage (Mb) overall process for four methods

ks	mash	bindash	dashing	dna2bit
15,11	1194	385	234	293
15,13	3736	760	445	294
15,15	13859	2238	1289	296
21,11	1756	384	234	294
21,13	5968	754	445	294
21,15	22784	2238	1289	296

Supplementary notes

Note S1 Details on computation efficiency and accuracy experiment.

Note S1.1 mash

The command we used to perform sketch experiment was:

```
nohup {time_path} -v {mash_path} sketch -p {threads} -k {k-mer} -s {sketch_size}
        -l {input_filenames_path} -o mash.{k-mer}.{sketch_size}.msh &>> nohup.out
```

- {time_path} is the path to the time software that is used to count time and memory.
- {mash_path} is the path of the mash software.
- {threads} is the number of the parallel threads to use.
- {k-mer} is the k-mer size (the length of sliding window).
- {sketch_size} is the sketch size.
- {input_filenames_path} is a file which contain all sequence file paths in each line.

The command we used to perform distance experiment was:

```
nohup {time_path} -v {mash_path} triangle -p {threads} mash.{k-mer}.{sketch_size}.mash
        1 > {mash_reault}.csv 2 &>> nohup.out
```

- {time_path} is the path to the time software that is used to count time and memory.
- {mash_path} is the path of the mash software.
- {threads} is the number of the parallel threads to use.
- {k-mer} is the k-mer size (the length of sliding window).
- {sketch_size} is the sketch size.
- {mash_result} is the output file with a triangle matrix of mash distance.

Note S1.2 bindash

The command we used to perform sketch experiment was:

```
nohup {time_path} -v {bindash_path} sketch --nthreads={threads} --kmerlen={k-mer}
      --sketchsize64={sketch_size} --listfname={input_filenames_path}
      --outfname=bindash.{k-mer}.{sketch_size}.bdash &>> nohup.out
```

- {time_path} is the path to the time software that is used to count time and memory.
- {bindash_path} is the path of the bindash software.
- {threads} is the number of the parallel threads to use.
- {k-mer} is the k-mer size (the length of sliding window).
- {sketch_size} is the sketch size divided by 64.
- {input_filenames_path} is a file which contain all sequence file paths in each line.

The command we used to perform distance experiment was:

```
nohup {time_path} -v {bindash_path} dist --nthreads={threads} --mthres=1e300
      bindash.{k-mer}.{sketch_size}.bdash 1> {bindash_result}.csv 2&>> nohup.out
```

- {time_path} is the path to the time software that is used to count time and memory.
- {bindash_path} is the path of the bindash software.
- {threads} is the number of the parallel threads to use.
- {k-mer} is the k-mer size (the length of sliding window).
- {sketch_size} is the sketch size divided by 64.
- {bindash_result} is the output file with the pairwise bindash distance.

Note S1.3 dashing

The command we used to perform sketch experiment was:

```
nohup {time_path} -v {dashing_path} sketch -p {threads} -k {k-mer} -S {sketch_size}
        -F {input_filenames_path} &>> nohup.out
```

- {time_path} is the path to the time software that is used to count time and memory.
- {dashing_path} is the path of the dashing software.
- {threads} is the number of the parallel threads to use.
- {k-mer} is the k-mer size (the length of sliding window).
- {sketch_size} is the $\log_2 sketch\ size$.
- {input_filenames_path} is a file which contain all sequence file paths in each line.

The command we used to perform distance experiment was:

```
nohup {time_path} -v {dashing_path} dist -p {threads} -k {k-mer} -S {sketch_size}
        -F {input_filenames_path} -O {dashing_result}.csv &>> nohup.out
```

- {time_path} is the path to the time software that is used to count time and memory.
- {dashing_path} is the path of the dashing software.
- {threads} is the number of the parallel threads to use.
- {k-mer} is the k-mer size (the length of sliding window).
- {sketch_size} is the $\log_2 sketch\ size$.
- {input_filenames_path} is a file which contain all sequence file paths in each line.
- {dashing_result} is the output file with a triangle matrix of dashing distance.

Note S1.4 dna2bit

The command we used to perform sketch experiment was:

```
nohup {time_path} -v {dan2bit_path} sketch -n {threads} -k {k-mer} -l {sketch_size}
        -F {input_filenames_path} &>> nohup.out
```

- {time_path} is the path to the time software that is used to count time and memory.
- {dna2bit_path} is the path of the dna2bit software.
- {threads} is the number of the parallel threads to use.
- {k-mer} is the k-mer size (the length of sliding window).
- {sketch_size} is the bit array length.
- {input_filenames_path} is a file which contain all sequence file paths in each line.

The command we used to perform distance experiment was:

```
nohup {time_path} -v {dna2bit_path} dist -n {threads} -F {input_bitnames_path}
        -o {dna2bit_result}.csv &>> nohup.out
```

- {time_path} is the path to the time software that is used to count time and memory.
- {dna2bit_path} is the path of the dna2bit software.
- {threads} is the number of the parallel threads to use.
- {input_bitnames_path} is a file which contain all bit array file paths in each line.
- {dna2bit_result} is the output file with a triangle matrix of dna2bit distance.
