# Supplementary material for: dna2bit: high performance genomic distance estimation software for microbial genome analysis
Source: Front Microbiol. 2024 Dec 23;15:1521181. doi: 10.3389/fmicb.2024.1521181 (PMC11701053; doi:10.3389/fmicb.2024.1521181)
Supplement: Supplementary file 2 [file Data_Sheet_2.pdf]

500 E.coli Genome Assembly accession number(GCA) in NCBI
GCA_000005845.2
GCA_000006665.1
GCA_000007445.1
GCA_000008865.2
GCA_000009565.2
GCA_000010245.1
GCA_000010385.1
GCA_000010485.1
GCA_000010745.1
GCA_000010765.1
GCA_000013265.1
GCA_000013305.1
GCA_000014845.1
GCA_000017745.1
GCA_000017765.1
GCA_000017985.1
GCA_000019385.1
GCA_000019425.1
GCA_000019645.1
GCA_000021125.1
GCA_000022225.1
GCA_000022345.1
GCA_000022665.2
GCA_000023365.1
GCA_000023665.1
GCA_000025165.1
GCA_000025745.1
GCA_000026245.1
GCA_000026265.1
GCA_000026285.2
GCA_000026305.1
GCA_000026325.2
GCA_000026345.1
GCA_000026545.1
GCA_000027125.1
GCA_000091005.1
GCA_000146735.1
GCA_000147755.2
GCA_000148365.1
GCA_000148605.1
GCA_000155125.1
GCA_000157115.2
GCA_000158395.1
GCA_000159295.1
GCA_000163155.1
GCA_000163175.1
GCA_000163195.1
GCA_000163215.1
GCA_000163235.1
GCA_000164195.1
GCA_000164215.1
GCA_000164235.1
GCA_000164255.1

GCA_000164275.1
GCA_000164295.1
GCA_000164315.1
GCA_000164335.1
GCA_000164355.1
GCA_000164375.1
GCA_000164415.1
GCA_000164435.1
GCA_000164455.1
GCA_000164475.1
GCA_000164495.1
GCA_000164515.1
GCA_000164535.1
GCA_000164555.1
GCA_000164575.1
GCA_000164595.1
GCA_000164615.1
GCA_000165655.2
GCA_000166535.3
GCA_000166555.2
GCA_000166575.2
GCA_000166595.2
GCA_000166615.2
GCA_000167815.1
GCA_000167835.1
GCA_000167855.1
GCA_000167875.2
GCA_000167895.3
GCA_000167915.2
GCA_000168095.1
GCA_000171915.1
GCA_000171935.1
GCA_000171955.1
GCA_000171975.1
GCA_000171995.1
GCA_000172015.1
GCA_000172035.1
GCA_000172055.1
GCA_000175735.1
GCA_000175755.1
GCA_000176535.2
GCA_000176555.2
GCA_000176575.2
GCA_000176595.2
GCA_000176615.2
GCA_000176635.2
GCA_000176655.2
GCA_000176675.2
GCA_000176695.2
GCA_000176815.1
GCA_000178315.1
GCA_000178695.1
GCA_000178715.1
GCA_000178735.1

GCA_000178755.1
GCA_000178775.1
GCA_000178795.1
GCA_000179075.1
GCA_000179095.1
GCA_000179115.1
GCA_000179135.1
GCA_000179155.1
GCA_000179175.1
GCA_000179795.1
GCA_000181735.1
GCA_000181755.1
GCA_000181775.1
GCA_000183005.2
GCA_000183345.1
GCA_000184185.1
GCA_000184765.2
GCA_000187285.4
GCA_000187305.3
GCA_000187325.3
GCA_000187345.2
GCA_000187365.2
GCA_000187385.2
GCA_000188755.2
GCA_000188775.2
GCA_000188815.2
GCA_000188835.2
GCA_000188855.2
GCA_000188875.2
GCA_000190795.1
GCA_000190815.1
GCA_000190835.1
GCA_000190855.1
GCA_000190895.1
GCA_000190915.1
GCA_000190955.1
GCA_000190975.1
GCA_000190995.1
GCA_000191015.1
GCA_000192665.2
GCA_000192685.2
GCA_000193955.2
GCA_000193975.2
GCA_000193995.2
GCA_000194175.2
GCA_000194215.2
GCA_000194235.2
GCA_000194255.1
GCA_000194295.2
GCA_000194335.2
GCA_000194355.2
GCA_000194395.2
GCA_000194415.2
GCA_000194435.2

GCA_000194475.2
GCA_000194535.2
GCA_000194555.2
GCA_000194575.2
GCA_000194645.2
GCA_000194665.2
GCA_000194685.2
GCA_000194705.2
GCA_000194725.2
GCA_000210475.1
GCA_000211395.2
GCA_000212715.2
GCA_000214765.3
GCA_000215145.2
GCA_000215165.1
GCA_000215185.2
GCA_000215205.2
GCA_000215225.2
GCA_000215245.2
GCA_000215265.2
GCA_000215285.2
GCA_000215685.3
GCA_000217695.2
GCA_000217975.2
GCA_000219515.3
GCA_000220005.2
GCA_000220805.2
GCA_000221065.2
GCA_000221885.1
GCA_000222505.2
GCA_000222525.2
GCA_000222545.2
GCA_000223015.2
GCA_000223035.2
GCA_000224395.2
GCA_000225025.2
GCA_000225045.2
GCA_000225065.2
GCA_000225085.2
GCA_000225105.2
GCA_000225125.2
GCA_000225145.2
GCA_000225165.2
GCA_000225185.3
GCA_000225205.2
GCA_000225225.2
GCA_000225245.2
GCA_000227625.1
GCA_000233675.2
GCA_000233875.1
GCA_000233895.1
GCA_000234215.2
GCA_000234235.2
GCA_000234255.3

GCA_000234275.3
GCA_000234295.2
GCA_000234315.2
GCA_000234605.2
GCA_000235045.1
GCA_000235065.1
GCA_000235085.1
GCA_000235105.1
GCA_000235125.1
GCA_000235145.1
GCA_000235165.1
GCA_000235185.1
GCA_000235205.1
GCA_000235225.1
GCA_000235245.1
GCA_000235265.1
GCA_000235285.1
GCA_000241975.1
GCA_000241995.1
GCA_000242015.1
GCA_000242035.1
GCA_000242055.1
GCA_000243195.1
GCA_000245515.1
GCA_000247665.4
GCA_000249055.2
GCA_000249075.2
GCA_000249095.2
GCA_000249115.2
GCA_000249135.2
GCA_000249155.2
GCA_000249175.2
GCA_000249195.2
GCA_000249215.2
GCA_000249235.2
GCA_000249255.2
GCA_000249275.2
GCA_000249295.2
GCA_000249315.2
GCA_000249335.2
GCA_000249355.2
GCA_000249375.2
GCA_000249395.2
GCA_000249415.2
GCA_000249435.2
GCA_000249455.2
GCA_000249475.2
GCA_000249495.2
GCA_000249515.2
GCA_000249535.2
GCA_000249555.2
GCA_000249575.2
GCA_000249595.2
GCA_000249615.2

GCA_000249635.2
GCA_000249655.2
GCA_000249675.2
GCA_000249695.2
GCA_000249715.2
GCA_000249735.2
GCA_000249755.2
GCA_000249775.2
GCA_000249795.2
GCA_000249815.2
GCA_000249835.2
GCA_000249855.2
GCA_000249875.2
GCA_000249895.2
GCA_000249915.2
GCA_000249935.2
GCA_000249955.2
GCA_000249975.2
GCA_000249995.2
GCA_000250015.2
GCA_000250035.2
GCA_000250055.2
GCA_000250075.2
GCA_000250095.2
GCA_000250115.2
GCA_000250135.2
GCA_000250155.2
GCA_000250175.2
GCA_000250195.2
GCA_000250215.2
GCA_000250235.2
GCA_000250255.2
GCA_000250275.2
GCA_000250295.2
GCA_000250315.2
GCA_000250335.2
GCA_000250355.2
GCA_000250375.2
GCA_000250395.2
GCA_000250415.2
GCA_000250435.2
GCA_000250455.2
GCA_000250475.2
GCA_000250495.2
GCA_000250515.2
GCA_000250535.2
GCA_000250555.2
GCA_000250575.2
GCA_000252805.2
GCA_000257275.1
GCA_000258025.1
GCA_000258145.1
GCA_000258225.1
GCA_000258615.1

GCA_000258635.1
GCA_000258785.1
GCA_000258865.1
GCA_000259135.1
GCA_000259385.1
GCA_000259425.1
GCA_000259695.1
GCA_000260475.1
GCA_000261145.1
GCA_000261385.1
GCA_000261405.1
GCA_000262125.1
GCA_000263895.1
GCA_000263915.1
GCA_000263935.1
GCA_000263955.1
GCA_000263975.1
GCA_000263995.1
GCA_000264015.1
GCA_000264035.1
GCA_000264095.1
GCA_000264115.1
GCA_000264135.1
GCA_000264155.1
GCA_000264175.1
GCA_000264195.1
GCA_000264215.1
GCA_000264235.1
GCA_000267005.2
GCA_000267025.1
GCA_000267045.1
GCA_000267065.1
GCA_000267085.1
GCA_000267105.1
GCA_000267125.1
GCA_000267145.1
GCA_000267165.1
GCA_000267185.1
GCA_000267205.2
GCA_000267225.2
GCA_000267245.2
GCA_000267265.2
GCA_000267285.2
GCA_000267305.2
GCA_000267325.1
GCA_000267345.2
GCA_000267365.2
GCA_000267385.2
GCA_000267405.2
GCA_000267425.2
GCA_000267445.2
GCA_000267465.2
GCA_000267485.2
GCA_000267505.2

GCA_000267525.2
GCA_000267585.2
GCA_000267605.2
GCA_000267625.2
GCA_000267645.2
GCA_000267665.2
GCA_000267685.2
GCA_000267705.2
GCA_000267725.2
GCA_000267745.2
GCA_000267765.2
GCA_000267785.2
GCA_000267805.2
GCA_000267825.2
GCA_000267845.2
GCA_000267865.2
GCA_000267885.2
GCA_000267905.2
GCA_000267925.2
GCA_000267945.2
GCA_000267965.2
GCA_000268125.1
GCA_000268205.1
GCA_000269645.2
GCA_000270105.1
GCA_000273425.1
GCA_000276745.1
GCA_000276765.1
GCA_000276785.1
GCA_000276845.1
GCA_000276865.1
GCA_000276885.1
GCA_000276925.1
GCA_000276945.1
GCA_000281775.1
GCA_000284495.1
GCA_000285375.1
GCA_000285655.3
GCA_000295775.2
GCA_000297235.3
GCA_000299255.1
GCA_000299455.1
GCA_000299475.1
GCA_000302715.1
GCA_000302735.1
GCA_000303235.2
GCA_000303255.2
GCA_000303275.2
GCA_000303295.2
GCA_000303315.2
GCA_000303335.2
GCA_000303355.2
GCA_000303375.2
GCA_000303395.2

GCA_000303415.2
GCA_000303435.2
GCA_000303455.2
GCA_000303475.2
GCA_000303495.2
GCA_000303515.2
GCA_000303535.2
GCA_000303555.2
GCA_000303575.2
GCA_000303595.2
GCA_000303615.2
GCA_000303635.2
GCA_000303655.2
GCA_000303675.2
GCA_000303695.2
GCA_000303715.2
GCA_000303735.2
GCA_000303755.2
GCA_000303775.2
GCA_000303795.2
GCA_000303815.2
GCA_000303835.2
GCA_000303855.2
GCA_000303875.2
GCA_000303895.2
GCA_000303915.2
GCA_000303935.2
GCA_000303955.2
GCA_000303975.2
GCA_000303995.2
GCA_000304015.2
GCA_000304035.2
GCA_000304055.2
GCA_000304075.2
GCA_000304095.2
GCA_000304115.2
GCA_000304135.2
GCA_000304255.1
GCA_000304815.2
GCA_000304835.2
GCA_000304855.2
GCA_000304875.2
GCA_000305155.2
GCA_000305175.1
GCA_000305355.1
GCA_000305375.2
GCA_000305395.2
GCA_000305415.2
GCA_000305435.2
GCA_000305455.2
GCA_000307205.1
GCA_000308975.2
GCA_000313405.1
GCA_000313425.1

GCA_000313445.1
GCA_000316325.2
GCA_000316345.2
GCA_000316365.2
GCA_000316385.2
GCA_000316405.2
GCA_000316425.2
GCA_000316445.2
GCA_000316465.1
GCA_000316485.1
GCA_000316505.2
GCA_000316545.2
GCA_000316565.2
GCA_000316705.2
GCA_000316725.2
