# Supplementary material for: dna2bit: high performance genomic distance estimation software for microbial genome analysis
Source: Front Microbiol. 2024 Dec 23;15:1521181. doi: 10.3389/fmicb.2024.1521181 (PMC11701053; doi:10.3389/fmicb.2024.1521181)
Supplement: Supplementary file 3 [file Data_Sheet_3.pdf]

370004 prokaryotic genome Genome Assembly accession number(GCA) in NCBI
GCA_000005825.2
GCA_000005845.2
GCA_000006605.1
GCA_000006625.1
GCA_000006645.1
GCA_000006685.1
GCA_000006725.1
GCA_000006745.1
GCA_000006765.1
GCA_000006785.2
GCA_000006825.1
GCA_000006845.1
GCA_000006865.1
GCA_000006885.1
GCA_000006905.1
GCA_000006925.2
GCA_000006945.2
GCA_000006965.1
GCA_000006985.1
GCA_000007025.1
GCA_000007045.1
GCA_000007085.1
GCA_000007105.1
GCA_000007125.1
GCA_000007145.1
GCA_000007165.1
GCA_000007205.1
GCA_000007245.1
GCA_000007265.1
GCA_000007285.1
GCA_000007365.1
GCA_000007385.1
GCA_000007405.1
GCA_000007425.1
GCA_000007445.1
GCA_000007465.2
GCA_000007485.1
GCA_000007505.1
GCA_000007525.1
GCA_000007545.1
GCA_000007565.2
GCA_000007585.1
GCA_000007605.1
GCA_000007625.1
GCA_000007645.1
GCA_000007665.1
GCA_000007685.1
GCA_000007705.1
GCA_000007725.1
GCA_000007745.1
GCA_000007765.2
GCA_000007785.1

GCA_000007805.1
GCA_000007825.1
GCA_000007845.1
GCA_000007865.1
GCA_000007885.1
GCA_000007905.1
GCA_000007925.1
GCA_000007945.1
GCA_000007985.2
GCA_000008005.1
GCA_000008025.1
GCA_000008045.1
GCA_000008065.1
GCA_000008105.1
GCA_000008125.1
GCA_000008145.1
GCA_000008165.1
GCA_000008185.1
GCA_000008205.1
GCA_000008225.1
GCA_000008245.1
GCA_000008285.1
GCA_000008305.1
GCA_000008325.1
GCA_000008345.1
GCA_000008365.1
GCA_000008385.1
GCA_000008405.1
GCA_000008425.1
GCA_000008445.1
GCA_000008465.1
GCA_000008485.1
GCA_000008505.1
GCA_000008525.1
GCA_000008545.1
GCA_000008565.1
GCA_000008585.1
GCA_000008605.1
GCA_000008625.1
GCA_000008685.2
GCA_000008725.1
GCA_000008745.1
GCA_000008765.1
GCA_000008785.1
GCA_000008805.1
GCA_000008865.2
GCA_000008885.1
GCA_000008925.1
GCA_000008945.1
GCA_000008985.1
GCA_000009005.1
GCA_000009025.1
GCA_000009045.1
GCA_000009065.1

GCA_000009085.1
GCA_000009105.1
GCA_000009125.1
GCA_000009145.1
GCA_000009165.1
GCA_000009205.2
GCA_000009245.1
GCA_000009265.1
GCA_000009285.2
GCA_000009305.1
GCA_000009325.1
GCA_000009345.1
GCA_000009365.1
GCA_000009385.1
GCA_000009405.1
GCA_000009425.1
GCA_000009445.1
GCA_000009465.1
GCA_000009485.1
GCA_000009505.1
GCA_000009525.1
GCA_000009545.1
GCA_000009565.2
GCA_000009585.1
GCA_000009605.1
GCA_000009625.1
GCA_000009645.1
GCA_000009665.1
GCA_000009685.1
GCA_000009705.1
GCA_000009725.1
GCA_000009745.1
GCA_000009765.2
GCA_000009785.1
GCA_000009805.1
GCA_000009825.1
GCA_000009865.1
GCA_000009885.1
GCA_000009905.1
GCA_000009925.1
GCA_000009945.1
GCA_000009985.1
GCA_000010005.1
GCA_000010025.1
GCA_000010045.1
GCA_000010065.1
GCA_000010085.1
GCA_000010105.1
GCA_000010125.1
GCA_000010145.1
GCA_000010165.1
GCA_000010185.1
GCA_000010205.1
GCA_000010225.1

GCA_000010245.1
GCA_000010265.1
GCA_000010285.1
GCA_000010305.1
GCA_000010325.1
GCA_000010345.1
GCA_000010365.1
GCA_000010385.1
GCA_000010405.1
GCA_000010425.1
GCA_000010445.1
GCA_000010465.1
GCA_000010485.1
GCA_000010505.1
GCA_000010525.1
GCA_000010545.1
GCA_000010565.1
GCA_000010585.1
GCA_000010605.1
GCA_000010625.1
GCA_000010645.1
GCA_000010665.1
GCA_000010685.1
GCA_000010705.1
GCA_000010725.1
GCA_000010745.1
GCA_000010765.1
GCA_000010785.1
GCA_000010805.1
GCA_000010825.1
GCA_000010845.1
GCA_000010865.1
GCA_000010885.1
GCA_000010905.1
GCA_000010925.1
GCA_000010945.1
GCA_000010965.1
GCA_000010985.1
GCA_000011025.1
GCA_000011045.1
GCA_000011065.1
GCA_000011145.1
GCA_000011165.1
GCA_000011225.1
GCA_000011245.1
GCA_000011265.1
GCA_000011285.1
GCA_000011305.1
GCA_000011325.1
GCA_000011345.1
GCA_000011365.1
GCA_000011385.1
GCA_000011405.1
GCA_000011445.1

GCA_000011465.1
GCA_000011485.1
GCA_000011505.1
GCA_000011525.1
GCA_000011545.1
GCA_000011565.2
GCA_000011605.1
GCA_000011625.1
GCA_000011645.1
GCA_000011665.1
GCA_000011685.1
GCA_000011705.1
GCA_000011725.1
GCA_000011745.1
GCA_000011765.2
GCA_000011805.1
GCA_000011825.1
GCA_000011845.1
GCA_000011865.1
GCA_000011885.1
GCA_000011905.1
GCA_000011925.1
GCA_000011945.1
GCA_000011965.2
GCA_000011985.1
GCA_000012005.1
GCA_000012025.1
GCA_000012045.1
GCA_000012085.2
GCA_000012105.1
GCA_000012125.1
GCA_000012165.2
GCA_000012185.1
GCA_000012205.1
GCA_000012225.1
GCA_000012245.1
GCA_000012265.1
GCA_000012305.1
GCA_000012325.1
GCA_000012345.1
GCA_000012365.1
GCA_000012385.1
GCA_000012445.1
GCA_000012465.1
GCA_000012485.1
GCA_000012505.1
GCA_000012525.1
GCA_000012565.1
GCA_000012625.1
GCA_000012645.1
GCA_000012665.1
GCA_000012685.1
GCA_000012705.1
GCA_000012725.1

GCA_000012745.1
GCA_000012765.1
GCA_000012785.1
GCA_000012805.1
GCA_000012825.1
GCA_000012845.1
GCA_000012865.1
GCA_000012885.1
GCA_000012925.1
GCA_000012945.1
GCA_000012965.1
GCA_000012985.1
GCA_000013005.1
GCA_000013025.1
GCA_000013045.1
GCA_000013085.1
GCA_000013125.1
GCA_000013145.1
GCA_000013165.1
GCA_000013185.1
GCA_000013205.1
GCA_000013225.1
GCA_000013245.1
GCA_000013265.1
GCA_000013285.1
GCA_000013305.1
GCA_000013325.1
GCA_000013345.1
GCA_000013365.1
GCA_000013385.1
GCA_000013405.1
GCA_000013425.1
GCA_000013465.1
GCA_000013485.1
GCA_000013505.1
GCA_000013525.1
GCA_000013545.1
GCA_000013565.1
GCA_000013585.1
GCA_000013605.1
GCA_000013625.1
GCA_000013645.1
GCA_000013665.1
GCA_000013705.1
GCA_000013765.1
GCA_000013785.1
GCA_000013805.1
GCA_000013825.1
GCA_000013845.2
GCA_000013865.1
GCA_000013885.1
GCA_000013905.1
GCA_000013925.2
GCA_000013945.1

GCA_000013965.1
GCA_000013985.1
GCA_000014005.1
GCA_000014025.1
GCA_000014045.1
GCA_000014065.1
GCA_000014105.1
GCA_000014125.1
GCA_000014145.1
GCA_000014185.1
GCA_000014205.1
GCA_000014285.2
GCA_000014305.1
GCA_000014325.1
GCA_000014345.1
GCA_000014365.2
GCA_000014385.1
GCA_000014405.1
GCA_000014425.1
GCA_000014445.1
GCA_000014465.1
GCA_000014485.1
GCA_000014505.1
GCA_000014525.1
GCA_000014545.1
GCA_000014565.1
GCA_000014585.1
GCA_000014605.1
GCA_000014625.1
GCA_000014645.1
GCA_000014685.1
GCA_000014705.1
GCA_000014725.1
GCA_000014745.1
GCA_000014765.1
GCA_000014785.1
GCA_000014805.1
GCA_000014845.1
GCA_000014865.1
GCA_000014885.1
GCA_000014925.1
GCA_000014965.1
GCA_000014985.1
GCA_000015005.1
GCA_000015025.1
GCA_000015045.1
GCA_000015065.1
GCA_000015085.1
GCA_000015105.1
GCA_000015125.1
GCA_000015165.1
GCA_000015185.1
GCA_000015245.1
GCA_000015265.1

GCA_000015285.1
GCA_000015305.1
GCA_000015325.1
GCA_000015345.1
GCA_000015365.1
GCA_000015385.1
GCA_000015425.1
GCA_000015445.1
GCA_000015465.1
GCA_000015485.1
GCA_000015505.1
GCA_000015525.1
GCA_000015565.1
GCA_000015585.1
GCA_000015605.1
GCA_000015625.1
GCA_000015645.1
GCA_000015665.1
GCA_000015685.1
GCA_000015705.1
GCA_000015725.1
GCA_000015745.1
GCA_000015785.2
GCA_000015845.1
GCA_000015865.1
GCA_000015885.1
GCA_000015905.1
GCA_000015925.1
GCA_000015965.1
GCA_000016045.1
GCA_000016065.1
GCA_000016105.1
GCA_000016145.1
GCA_000016165.1
GCA_000016185.1
GCA_000016205.1
GCA_000016245.1
GCA_000016265.1
GCA_000016285.1
GCA_000016305.1
GCA_000016325.1
GCA_000016345.1
GCA_000016425.1
GCA_000016445.1
GCA_000016465.1
GCA_000016485.1
GCA_000016505.1
GCA_000016545.1
GCA_000016625.1
GCA_000016645.1
GCA_000016665.1
GCA_000016685.1
GCA_000016725.1
GCA_000016745.1

GCA_000016785.1
GCA_000016805.1
GCA_000016825.1
GCA_000016845.1
GCA_000016905.1
GCA_000016925.1
GCA_000016945.1
GCA_000016965.1
GCA_000016985.1
GCA_000017005.1
GCA_000017025.1
GCA_000017045.1
GCA_000017065.1
GCA_000017085.1
GCA_000017105.1
GCA_000017145.1
GCA_000017205.1
GCA_000017265.1
GCA_000017305.1
GCA_000017325.1
GCA_000017405.1
GCA_000017425.1
GCA_000017445.3
GCA_000017465.2
GCA_000017485.1
GCA_000017505.1
GCA_000017525.1
GCA_000017545.1
GCA_000017565.1
GCA_000017585.1
GCA_000017605.1
GCA_000017665.1
GCA_000017685.1
GCA_000017705.1
GCA_000017725.2
GCA_000017745.1
GCA_000017765.1
GCA_000017785.1
GCA_000017805.1
GCA_000017845.1
GCA_000017865.1
GCA_000017885.4
GCA_000017905.1
GCA_000017965.1
GCA_000017985.1
GCA_000018025.1
GCA_000018045.1
GCA_000018065.1
GCA_000018105.1
GCA_000018125.1
GCA_000018145.1
GCA_000018185.1
GCA_000018205.1
GCA_000018225.1

GCA_000018245.1
GCA_000018285.1
GCA_000018325.1
GCA_000018345.1
GCA_000018385.1
GCA_000018405.1
GCA_000018425.1
GCA_000018445.1
GCA_000018505.1
GCA_000018525.1
GCA_000018545.1
GCA_000018565.1
GCA_000018605.1
GCA_000018625.1
GCA_000018665.1
GCA_000018685.1
GCA_000018705.1
GCA_000018725.1
GCA_000018745.1
GCA_000018765.1
GCA_000018785.1
GCA_000018805.1
GCA_000018825.1
GCA_000018845.1
GCA_000018865.1
GCA_000018885.1
GCA_000018905.1
GCA_000018925.1
GCA_000018945.1
GCA_000018965.1
GCA_000018985.1
GCA_000019005.1
GCA_000019025.1
GCA_000019045.1
GCA_000019065.1
GCA_000019085.1
GCA_000019125.1
GCA_000019165.1
GCA_000019185.1
GCA_000019205.1
GCA_000019225.1
GCA_000019245.3
GCA_000019265.1
GCA_000019305.1
GCA_000019325.1
GCA_000019345.1
GCA_000019365.1
GCA_000019385.1
GCA_000019405.1
GCA_000019425.1
GCA_000019485.1
GCA_000019505.1
GCA_000019525.1
GCA_000019545.1

GCA_000019565.1
GCA_000019585.2
GCA_000019625.1
GCA_000019645.1
GCA_000019665.1
GCA_000019705.1
GCA_000019725.1
GCA_000019765.1
GCA_000019785.1
GCA_000019825.1
GCA_000019845.1
GCA_000019865.1
GCA_000019885.1
GCA_000019905.1
GCA_000019925.1
GCA_000019945.1
GCA_000019965.1
GCA_000019985.1
GCA_000020005.1
GCA_000020025.1
GCA_000020045.1
GCA_000020065.1
GCA_000020105.1
GCA_000020125.1
GCA_000020145.1
GCA_000020165.1
GCA_000020185.1
GCA_000020225.1
GCA_000020245.1
GCA_000020265.1
GCA_000020285.1
GCA_000020305.1
GCA_000020325.1
GCA_000020345.1
GCA_000020365.1
GCA_000020385.1
GCA_000020405.1
GCA_000020425.1
GCA_000020445.1
GCA_000020465.1
GCA_000020485.1
GCA_000020505.1
GCA_000020525.1
GCA_000020565.1
GCA_000020585.3
GCA_000020605.1
GCA_000020625.1
GCA_000020645.1
GCA_000020665.1
GCA_000020685.1
GCA_000020705.1
GCA_000020725.1
GCA_000020745.1
GCA_000020765.1

GCA_000020805.1
GCA_000020825.1
GCA_000020865.1
GCA_000020885.1
GCA_000020925.1
GCA_000020945.1
GCA_000020965.1
GCA_000020985.1
GCA_000021005.1
GCA_000021025.1
GCA_000021045.1
GCA_000021065.1
GCA_000021085.1
GCA_000021125.1
GCA_000021165.1
GCA_000021185.1
GCA_000021205.1
GCA_000021245.2
GCA_000021265.1
GCA_000021285.1
GCA_000021305.1
GCA_000021325.1
GCA_000021345.1
GCA_000021365.1
GCA_000021405.1
GCA_000021425.1
GCA_000021445.1
GCA_000021465.1
GCA_000021485.1
GCA_000021505.1
GCA_000021525.1
GCA_000021545.1
GCA_000021565.1
GCA_000021605.1
GCA_000021625.1
GCA_000021645.1
GCA_000021665.1
GCA_000021685.1
GCA_000021725.1
GCA_000021745.1
GCA_000021765.1
GCA_000021785.1
GCA_000021805.1
GCA_000021825.1
GCA_000021845.1
GCA_000021865.1
GCA_000021885.1
GCA_000021905.1
GCA_000021925.1
GCA_000021945.1
GCA_000021985.1
GCA_000022005.1
GCA_000022025.1
GCA_000022065.1

GCA_000022085.1
GCA_000022105.1
GCA_000022145.1
GCA_000022165.1
GCA_000022225.1
GCA_000022245.1
GCA_000022265.1
GCA_000022285.1
GCA_000022305.1
GCA_000022325.1
GCA_000022345.1
GCA_000022505.1
GCA_000022525.1
GCA_000022565.1
GCA_000022585.1
GCA_000022605.2
GCA_000022625.1
GCA_000022645.2
GCA_000022665.2
GCA_000022685.1
GCA_000022705.1
GCA_000022725.1
GCA_000022745.1
GCA_000022765.1
GCA_000022785.1
GCA_000022805.1
GCA_000022825.1
GCA_000022845.1
GCA_000022865.1
GCA_000022885.2
GCA_000022905.1
GCA_000022925.1
GCA_000022945.1
GCA_000022965.1
GCA_000022985.1
GCA_000023005.1
GCA_000023025.1
GCA_000023045.1
GCA_000023065.1
GCA_000023085.1
GCA_000023105.1
GCA_000023125.1
GCA_000023145.1
GCA_000023165.1
GCA_000023185.1
GCA_000023205.1
GCA_000023225.1
GCA_000023245.1
GCA_000023265.1
GCA_000023285.1
GCA_000023305.1
GCA_000023325.1
GCA_000023365.1
GCA_000023405.1

GCA_000023445.1
GCA_000023465.1
GCA_000023545.1
GCA_000023565.1
GCA_000023585.1
GCA_000023605.1
GCA_000023625.1
GCA_000023665.1
GCA_000023685.1
GCA_000023705.1
GCA_000023725.1
GCA_000023745.1
GCA_000023765.2
GCA_000023785.1
GCA_000023805.1
GCA_000023825.1
GCA_000023845.1
GCA_000023865.1
GCA_000023885.1
GCA_000023905.1
GCA_000023925.1
GCA_000024005.1
GCA_000024025.1
GCA_000024065.1
GCA_000024085.1
GCA_000024105.1
GCA_000024125.1
GCA_000024145.1
GCA_000024205.1
GCA_000024225.1
GCA_000024245.1
GCA_000024265.1
GCA_000024285.1
GCA_000024325.1
GCA_000024345.1
GCA_000024365.1
GCA_000024385.1
GCA_000024405.1
GCA_000024425.1
GCA_000024445.1
GCA_000024465.1
GCA_000024505.1
GCA_000024525.1
GCA_000024545.1
GCA_000024565.1
GCA_000024585.1
GCA_000024605.1
GCA_000024645.1
GCA_000024665.1
GCA_000024685.1
GCA_000024725.1
GCA_000024765.1
GCA_000024785.1
GCA_000024805.1

GCA_000024825.1
GCA_000024845.1
GCA_000024865.1
GCA_000024885.1
GCA_000024905.1
GCA_000024925.1
GCA_000024945.1
GCA_000024965.1
GCA_000024985.1
GCA_000025005.1
GCA_000025025.1
GCA_000025045.1
GCA_000025065.1
GCA_000025085.1
GCA_000025105.1
GCA_000025125.1
GCA_000025145.2
GCA_000025165.1
GCA_000025185.1
GCA_000025205.1
GCA_000025225.2
GCA_000025245.2
GCA_000025265.1
GCA_000025305.1
GCA_000025345.1
GCA_000025365.1
GCA_000025385.1
GCA_000025405.2
GCA_000025425.1
GCA_000025465.1
GCA_000025485.1
GCA_000025545.1
GCA_000025565.1
GCA_000025605.1
GCA_000025645.1
GCA_000025705.1
GCA_000025725.1
GCA_000025745.1
GCA_000025765.1
GCA_000025785.1
GCA_000025805.1
GCA_000025825.1
GCA_000025845.1
GCA_000025885.1
GCA_000025905.1
GCA_000025925.1
GCA_000025945.1
GCA_000025965.1
GCA_000025985.1
GCA_000026005.1
GCA_000026025.1
GCA_000026065.1
GCA_000026085.1
GCA_000026105.1

GCA_000026125.1
GCA_000026145.1
GCA_000026185.1
GCA_000026205.1
GCA_000026225.1
GCA_000026245.1
GCA_000026265.1
GCA_000026285.2
GCA_000026345.1
GCA_000026405.1
GCA_000026445.2
GCA_000026485.1
GCA_000026505.1
GCA_000026525.1
GCA_000026545.1
GCA_000026565.1
GCA_000026585.1
GCA_000026605.1
GCA_000026645.1
GCA_000026665.1
GCA_000026685.1
GCA_000026705.1
GCA_000026725.1
GCA_000026745.1
GCA_000026905.1
GCA_000026925.1
GCA_000026965.1
GCA_000027025.1
GCA_000027045.1
GCA_000027065.2
GCA_000027085.1
GCA_000027125.1
GCA_000027145.1
GCA_000027165.1
GCA_000027185.1
GCA_000027205.1
GCA_000027225.1
GCA_000027265.1
GCA_000027305.1
GCA_000027325.1
GCA_000027345.1
GCA_000039765.1
GCA_000043285.1
GCA_000046685.1
GCA_000046705.1
GCA_000046845.1
GCA_000047365.1
GCA_000048645.1
GCA_000048665.1
GCA_000050405.1
GCA_000050425.1
GCA_000054005.1
GCA_000055785.1
GCA_000055945.1

GCA_000056065.1
GCA_000058485.1
GCA_000060285.1
GCA_000060345.1
GCA_000061505.1
GCA_000062885.1
GCA_000063485.1
GCA_000063505.1
GCA_000063525.1
GCA_000063545.1
GCA_000063585.1
GCA_000063605.1
GCA_000064305.2
GCA_000067045.1
GCA_000067165.1
GCA_000067205.1
GCA_000068525.2
GCA_000068585.1
GCA_000069185.1
GCA_000069225.1
GCA_000069245.1
GCA_000069925.1
GCA_000069945.1
GCA_000069965.1
GCA_000070465.1
GCA_000070605.1
GCA_000072485.1
GCA_000083545.1
GCA_000083565.1
GCA_000085225.1
GCA_000085865.1
GCA_000087965.1
GCA_000089865.1
GCA_000090405.1
GCA_000090965.1
GCA_000091005.1
GCA_000091085.2
GCA_000091125.1
GCA_000091165.1
GCA_000091305.1
GCA_000091325.1
GCA_000091345.1
GCA_000091405.1
GCA_000091465.1
GCA_000091545.1
GCA_000091565.1
GCA_000091645.1
GCA_000091725.1
GCA_000091785.1
GCA_000091905.1
GCA_000091985.1
GCA_000092025.1
GCA_000092045.1
GCA_000092105.1

GCA_000092125.1
GCA_000092165.1
GCA_000092205.1
GCA_000092225.1
GCA_000092245.1
GCA_000092265.1
GCA_000092285.1
GCA_000092325.1
GCA_000092345.1
GCA_000092365.1
GCA_000092385.1
GCA_000092405.1
GCA_000092425.1
GCA_000092445.1
GCA_000092485.1
GCA_000092505.1
GCA_000092525.1
GCA_000092545.1
GCA_000092565.1
GCA_000092585.1
GCA_000092625.1
GCA_000092645.1
GCA_000092665.1
GCA_000092685.1
GCA_000092705.1
GCA_000092725.1
GCA_000092745.1
GCA_000092765.1
GCA_000092785.1
GCA_000092805.1
GCA_000092825.1
GCA_000092845.1
GCA_000092865.1
GCA_000092885.1
GCA_000092905.1
GCA_000092925.1
GCA_000092945.1
GCA_000092965.1
GCA_000092985.1
GCA_000093005.1
GCA_000093025.1
GCA_000093065.1
GCA_000093085.1
GCA_000093125.2
GCA_000093145.2
GCA_000093165.1
GCA_000093185.1
GCA_000143085.1
GCA_000143145.1
GCA_000143205.1
GCA_000143225.1
GCA_000143435.1
GCA_000143605.1
GCA_000143685.1

GCA_000143705.2
GCA_000143725.1
GCA_000143845.1
GCA_000143865.1
GCA_000143885.2
GCA_000143945.1
GCA_000143965.1
GCA_000143985.1
GCA_000144405.1
GCA_000144605.1
GCA_000144625.1
GCA_000144645.1
GCA_000144675.2
GCA_000144695.1
GCA_000144935.3
GCA_000144955.2
GCA_000145035.1
GCA_000145215.1
GCA_000145235.1
GCA_000145255.1
GCA_000145275.1
GCA_000145595.1
GCA_000145615.1
GCA_000145825.2
GCA_000145845.2
GCA_000145945.2
GCA_000146005.2
GCA_000146025.1
GCA_000146065.1
GCA_000146165.2
GCA_000146185.1
GCA_000146265.4
GCA_000146305.1
GCA_000146505.1
GCA_000146565.1
GCA_000146875.2
GCA_000146975.1
GCA_000147015.1
GCA_000147035.1
GCA_000147055.1
GCA_000147075.1
GCA_000147095.1
GCA_000147335.1
GCA_000147355.1
GCA_000147695.3
GCA_000147715.3
GCA_000147735.3
GCA_000147775.3
GCA_000147795.3
GCA_000147815.3
GCA_000147835.3
GCA_000148365.1
GCA_000148405.1
GCA_000148585.3

GCA_000148605.1
GCA_000148625.1
GCA_000148645.1
GCA_000148665.1
GCA_000148705.1
GCA_000148815.2
GCA_000148855.1
GCA_000148875.1
GCA_000148895.1
GCA_000148915.1
GCA_000148935.1
GCA_000152065.3
GCA_000152165.1
GCA_000152245.2
GCA_000152825.2
GCA_000152925.3
GCA_000152945.2
GCA_000153165.2
GCA_000153405.2
GCA_000153485.2
GCA_000153685.2
GCA_000154585.2
GCA_000154605.2
GCA_000154745.2
GCA_000154765.2
GCA_000154785.2
GCA_000155515.2
GCA_000155675.2
GCA_000155735.2
GCA_000156855.2
GCA_000156995.2
GCA_000157355.2
GCA_000157895.2
GCA_000158275.2
GCA_000159155.2
GCA_000159455.2
GCA_000159535.2
GCA_000160335.2
GCA_000161795.2
GCA_000162235.2
GCA_000163055.2
GCA_000163615.3
GCA_000163895.2
GCA_000163915.2
GCA_000164015.3
GCA_000164675.2
GCA_000164695.2
GCA_000164865.1
GCA_000164885.1
GCA_000164905.1
GCA_000164965.1
GCA_000164985.2
GCA_000165465.1
GCA_000165485.1

GCA_000165505.1
GCA_000165525.1
GCA_000165575.1
GCA_000165715.3
GCA_000165775.3
GCA_000165815.1
GCA_000165835.1
GCA_000165905.1
GCA_000165925.1
GCA_000166055.1
GCA_000166115.1
GCA_000166135.1
GCA_000166295.1
GCA_000166315.1
GCA_000166335.1
GCA_000166355.1
GCA_000166395.1
GCA_000166415.1
GCA_000166455.2
GCA_000166635.2
GCA_000166655.2
GCA_000166695.1
GCA_000166775.1
GCA_000167435.2
GCA_000167875.2
GCA_000168315.3
GCA_000168355.3
GCA_000168575.2
GCA_000168595.2
GCA_000168635.2
GCA_000168695.2
GCA_000168775.2
GCA_000168875.3
GCA_000169195.2
GCA_000171795.2
GCA_000172575.2
GCA_000172635.2
GCA_000172695.2
GCA_000172715.2
GCA_000172795.2
GCA_000174395.2
GCA_000174795.2
GCA_000175215.2
GCA_000175255.2
GCA_000175295.2
GCA_000175575.2
GCA_000175935.2
GCA_000176035.2
GCA_000176835.2
GCA_000176855.2
GCA_000176915.2
GCA_000177195.2
GCA_000177235.2
GCA_000177535.2

GCA_000177615.2
GCA_000177635.2
GCA_000177655.2
GCA_000178115.2
GCA_000178395.2
GCA_000178495.2
GCA_000178835.2
GCA_000178875.2
GCA_000178935.2
GCA_000178955.2
GCA_000178975.2
GCA_000179015.2
GCA_000179035.2
GCA_000179235.2
GCA_000179395.2
GCA_000179635.2
GCA_000179915.2
GCA_000180175.2
GCA_000180515.2
GCA_000182745.1
GCA_000182835.1
GCA_000182875.1
GCA_000183115.1
GCA_000183135.1
GCA_000183155.1
GCA_000183185.1
GCA_000183225.1
GCA_000183245.1
GCA_000183285.1
GCA_000183305.1
GCA_000183345.1
GCA_000183365.1
GCA_000183385.1
GCA_000183405.1
GCA_000183425.1
GCA_000183645.1
GCA_000183665.1
GCA_000183725.1
GCA_000183745.1
GCA_000184065.1
GCA_000184085.1
GCA_000184185.1
GCA_000184205.1
GCA_000184325.1
GCA_000184345.2
GCA_000184435.1
GCA_000184685.1
GCA_000184705.1
GCA_000184745.1
GCA_000184925.1
GCA_000185185.1
GCA_000185205.1
GCA_000185225.1
GCA_000185245.1

GCA_000185805.1
GCA_000185885.1
GCA_000185905.1
GCA_000185965.1
GCA_000185985.2
GCA_000186005.1
GCA_000186225.1
GCA_000186245.1
GCA_000186265.1
GCA_000186345.1
GCA_000186385.1
GCA_000186405.1
GCA_000186585.1
GCA_000186665.4
GCA_000186725.1
GCA_000186745.1
GCA_000186885.1
GCA_000186985.3
GCA_000187005.1
GCA_000187205.4
GCA_000187705.1
GCA_000188215.1
GCA_000188715.1
GCA_000188735.1
GCA_000188955.5
GCA_000189295.2
GCA_000189415.1
GCA_000189435.3
GCA_000189455.3
GCA_000189495.1
GCA_000189515.1
GCA_000189535.1
GCA_000189735.2
GCA_000189775.3
GCA_000190435.1
GCA_000190535.1
GCA_000190555.1
GCA_000190575.1
GCA_000190595.1
GCA_000190635.1
GCA_000190735.2
GCA_000191045.1
GCA_000191145.1
GCA_000191165.1
GCA_000191425.1
GCA_000191445.1
GCA_000191465.1
GCA_000191485.1
GCA_000191505.1
GCA_000191525.1
GCA_000191545.1
GCA_000191565.1
GCA_000191905.1
GCA_000191925.1

GCA_000192045.3
GCA_000192105.1
GCA_000192315.1
GCA_000192335.1
GCA_000192395.2
GCA_000192635.1
GCA_000192705.1
GCA_000192725.1
GCA_000192745.1
GCA_000192865.1
GCA_000192885.1
GCA_000193185.2
GCA_000193205.1
GCA_000193355.1
GCA_000193395.1
GCA_000193435.3
GCA_000193495.2
GCA_000193595.3
GCA_000193615.3
GCA_000194075.3
GCA_000194115.1
GCA_000194135.1
GCA_000194605.1
GCA_000194745.1
GCA_000194765.1
GCA_000194785.1
GCA_000194805.1
GCA_000195065.1
GCA_000195085.1
GCA_000195105.1
GCA_000195275.1
GCA_000195295.1
GCA_000195315.1
GCA_000195335.1
GCA_000195395.6
GCA_000195435.4
GCA_000195515.1
GCA_000195535.1
GCA_000195555.1
GCA_000195575.1
GCA_000195675.1
GCA_000195695.1
GCA_000195715.1
GCA_000195735.1
GCA_000195755.1
GCA_000195775.2
GCA_000195795.1
GCA_000195815.1
GCA_000195835.3
GCA_000195855.1
GCA_000195875.1
GCA_000195955.2
GCA_000195975.1
GCA_000195995.1

GCA_000196015.1
GCA_000196035.1
GCA_000196055.1
GCA_000196075.1
GCA_000196095.1
GCA_000196115.1
GCA_000196135.1
GCA_000196155.1
GCA_000196175.1
GCA_000196215.1
GCA_000196235.1
GCA_000196255.1
GCA_000196275.1
GCA_000196295.1
GCA_000196315.1
GCA_000196335.1
GCA_000196355.1
GCA_000196395.1
GCA_000196435.1
GCA_000196455.1
GCA_000196475.1
GCA_000196495.1
GCA_000196515.1
GCA_000196535.1
GCA_000196555.1
GCA_000196575.1
GCA_000196595.1
GCA_000196615.1
GCA_000196695.1
GCA_000196735.1
GCA_000196755.1
GCA_000196795.1
GCA_000196815.1
GCA_000196835.1
GCA_000196855.1
GCA_000196875.2
GCA_000197735.1
GCA_000197755.2
GCA_000197875.1
GCA_000198515.1
GCA_000198775.1
GCA_000199675.1
GCA_000200475.1
GCA_000200595.1
GCA_000200735.1
GCA_000202635.1
GCA_000202835.1
GCA_000203195.1
GCA_000203215.1
GCA_000203835.1
GCA_000203855.3
GCA_000203895.1
GCA_000203915.1
GCA_000203935.1

GCA_000203955.1
GCA_000204075.1
GCA_000204115.1
GCA_000204135.1
GCA_000204155.1
GCA_000204255.1
GCA_000204275.1
GCA_000204295.1
GCA_000204565.1
GCA_000204625.1
GCA_000204645.1
GCA_000204665.1
GCA_000204985.1
GCA_000208385.1
GCA_000208405.1
GCA_000209655.1
GCA_000209675.1
GCA_000209755.1
GCA_000209795.2
GCA_000210155.1
GCA_000210315.1
GCA_000210415.1
GCA_000210435.1
GCA_000210455.1
GCA_000210475.1
GCA_000210495.1
GCA_000210515.1
GCA_000210815.2
GCA_000210835.1
GCA_000210855.2
GCA_000210875.1
GCA_000210895.1
GCA_000210915.2
GCA_000210935.1
GCA_000210955.1
GCA_000210975.1
GCA_000210995.1
GCA_000211015.1
GCA_000211035.2
GCA_000211055.2
GCA_000211075.1
GCA_000211095.1
GCA_000211235.1
GCA_000211255.2
GCA_000211375.1
GCA_000211545.6
GCA_000211855.3
GCA_000212355.1
GCA_000212375.1
GCA_000212395.1
GCA_000212415.1
GCA_000212675.2
GCA_000212695.1
GCA_000212715.2

GCA_000212735.1
GCA_000212755.3
GCA_000212975.1
GCA_000213135.1
GCA_000213155.1
GCA_000213235.1
GCA_000213255.1
GCA_000213635.1
GCA_000213655.1
GCA_000213785.1
GCA_000213805.1
GCA_000213825.1
GCA_000213865.1
GCA_000213955.1
GCA_000214095.3
GCA_000214155.1
GCA_000214175.1
GCA_000214195.1
GCA_000214215.1
GCA_000214235.1
GCA_000214355.1
GCA_000214375.1
GCA_000214395.1
GCA_000214435.1
GCA_000214665.1
GCA_000214705.1
GCA_000214785.1
GCA_000214805.1
GCA_000214825.1
GCA_000214845.1
GCA_000215065.1
GCA_000215085.1
GCA_000215105.1
GCA_000215325.1
GCA_000215345.3
GCA_000215645.1
GCA_000215665.1
GCA_000215705.1
GCA_000215745.1
GCA_000215895.1
GCA_000215975.1
GCA_000217615.1
GCA_000217635.1
GCA_000217655.1
GCA_000217675.1
GCA_000217795.1
GCA_000217815.1
GCA_000217835.1
GCA_000218265.1
GCA_000218305.1
GCA_000218545.1
GCA_000218565.1
GCA_000218585.1
GCA_000218625.1

GCA_000218855.1
GCA_000218875.1
GCA_000218895.1
GCA_000218915.1
GCA_000219045.1
GCA_000219085.3
GCA_000219105.1
GCA_000219175.1
GCA_000219195.1
GCA_000219215.1
GCA_000219355.1
GCA_000219375.1
GCA_000219455.1
GCA_000219475.3
GCA_000219515.3
GCA_000219535.3
GCA_000219605.1
GCA_000219705.1
GCA_000219725.1
GCA_000219765.1
GCA_000219785.1
GCA_000219805.1
GCA_000219855.1
GCA_000219875.1
GCA_000219915.3
GCA_000220105.1
GCA_000220135.1
GCA_000220485.1
GCA_000220625.1
GCA_000220705.2
GCA_000220885.1
GCA_000220945.1
GCA_000220965.1
GCA_000221005.1
GCA_000221025.1
GCA_000221045.1
GCA_000221205.1
GCA_000221625.1
GCA_000221645.1
GCA_000221965.1
GCA_000221985.1
GCA_000222305.1
GCA_000222485.1
GCA_000222835.1
GCA_000222975.1
GCA_000223195.1
GCA_000223215.1
GCA_000223375.1
GCA_000223885.1
GCA_000224005.3
GCA_000224085.1
GCA_000224105.1
GCA_000224435.1
GCA_000224535.1

GCA_000224555.1
GCA_000224575.1
GCA_000224675.1
GCA_000224745.1
GCA_000224965.2
GCA_000224985.1
GCA_000225265.1
GCA_000225325.1
GCA_000225345.1
GCA_000225445.1
GCA_000225465.1
GCA_000225915.1
GCA_000225955.1
GCA_000226035.3
GCA_000226155.1
GCA_000226275.2
GCA_000226295.1
GCA_000226315.1
GCA_000226565.1
GCA_000226605.1
GCA_000226625.1
GCA_000227175.1
GCA_000227465.1
GCA_000227485.1
GCA_000227605.3
GCA_000227625.1
GCA_000227645.1
GCA_000227665.3
GCA_000227685.3
GCA_000227705.3
GCA_000227745.3
GCA_000230275.1
GCA_000230295.1
GCA_000230555.1
GCA_000230655.3
GCA_000230695.3
GCA_000230895.3
GCA_000230975.3
GCA_000230995.3
GCA_000231175.1
GCA_000231215.1
GCA_000231385.3
GCA_000231405.3
GCA_000231865.1
GCA_000231885.1
GCA_000231905.1
GCA_000231925.1
GCA_000233435.1
GCA_000233575.1
GCA_000233595.1
GCA_000233715.3
GCA_000233735.1
GCA_000233755.1
GCA_000233775.1

GCA_000233875.1
GCA_000233895.1
GCA_000233915.4
GCA_000234725.1
GCA_000234745.1
GCA_000234825.3
GCA_000235405.3
GCA_000235545.1
GCA_000235585.1
GCA_000235605.1
GCA_000235765.3
GCA_000236215.4
GCA_000236255.1
GCA_000236405.1
GCA_000236455.2
GCA_000236475.1
GCA_000236585.1
GCA_000236605.1
GCA_000236665.1
GCA_000236685.1
GCA_000236705.1
GCA_000236925.1
GCA_000237065.1
GCA_000237085.1
GCA_000237125.3
GCA_000237145.1
GCA_000237205.1
GCA_000237265.1
GCA_000237305.1
GCA_000237325.1
GCA_000237845.1
GCA_000237975.1
GCA_000237995.2
GCA_000238175.1
GCA_000238195.1
GCA_000238215.1
GCA_000238255.4
GCA_000238395.4
GCA_000238455.3
GCA_000238995.1
GCA_000239175.1
GCA_000239195.1
GCA_000239235.1
GCA_000239475.1
GCA_000239975.1
GCA_000240015.1
GCA_000240035.1
GCA_000240055.1
GCA_000240075.2
GCA_000240095.3
GCA_000240165.1
GCA_000240185.2
GCA_000240325.1
GCA_000240905.3

GCA_000241025.2
GCA_000241385.1
GCA_000241585.3
GCA_000241855.1
GCA_000241875.1
GCA_000241895.1
GCA_000241915.1
GCA_000241935.1
GCA_000242255.3
GCA_000242335.3
GCA_000242455.3
GCA_000242595.3
GCA_000242635.3
GCA_000242775.1
GCA_000242855.2
GCA_000242895.3
GCA_000242935.3
GCA_000243075.1
GCA_000243115.3
GCA_000243135.3
GCA_000243155.3
GCA_000244875.1
GCA_000245355.1
GCA_000245495.1
GCA_000245515.1
GCA_000245535.1
GCA_000246835.1
GCA_000246855.1
GCA_000247565.1
GCA_000247605.1
GCA_000247715.1
GCA_000248095.3
GCA_000248375.2
GCA_000248415.2
GCA_000248435.2
GCA_000250635.1
GCA_000250655.1
GCA_000250675.3
GCA_000250855.1
GCA_000250905.1
GCA_000250925.1
GCA_000250945.1
GCA_000251085.2
GCA_000252365.1
GCA_000252445.1
GCA_000252855.1
GCA_000252875.1
GCA_000252975.1
GCA_000252995.1
GCA_000253015.1
GCA_000253035.1
GCA_000253075.1
GCA_000253135.1
GCA_000253155.1

GCA_000253175.1
GCA_000253195.1
GCA_000253215.1
GCA_000253255.1
GCA_000253275.1
GCA_000253295.1
GCA_000253315.1
GCA_000253335.1
GCA_000253355.1
GCA_000253375.1
GCA_000253395.1
GCA_000255115.3
GCA_000255135.1
GCA_000255155.1
GCA_000255175.1
GCA_000255195.1
GCA_000255215.1
GCA_000255235.1
GCA_000255255.1
GCA_000255275.1
GCA_000255295.1
GCA_000255535.1
GCA_000255615.3
GCA_000255915.1
GCA_000255935.1
GCA_000255955.1
GCA_000257275.1
GCA_000257545.3
GCA_000258025.1
GCA_000258145.1
GCA_000258245.1
GCA_000258365.1
GCA_000258385.1
GCA_000258405.1
GCA_000258535.2
GCA_000258885.1
GCA_000258905.1
GCA_000259155.4
GCA_000259175.1
GCA_000259235.1
GCA_000259255.1
GCA_000259275.1
GCA_000259365.1
GCA_000259545.1
GCA_000260515.1
GCA_000260715.1
GCA_000260925.1
GCA_000260965.1
GCA_000260985.3
GCA_000261025.1
GCA_000261045.2
GCA_000261345.2
GCA_000262125.1
GCA_000262145.1

GCA_000262165.1
GCA_000262205.1
GCA_000262305.1
GCA_000262325.2
GCA_000262385.1
GCA_000262655.1
GCA_000262675.1
GCA_000262695.1
GCA_000262715.1
GCA_000263195.1
GCA_000263215.1
GCA_000263755.3
GCA_000263795.2
GCA_000264455.2
GCA_000264665.2
GCA_000264765.2
GCA_000264945.2
GCA_000265095.1
GCA_000265295.1
GCA_000265365.1
GCA_000265385.1
GCA_000265405.1
GCA_000265425.1
GCA_000265465.1
GCA_000265505.1
GCA_000265545.3
GCA_000266885.1
GCA_000266905.1
GCA_000266925.1
GCA_000266945.1
GCA_000267545.1
GCA_000269925.1
GCA_000269945.1
GCA_000269965.1
GCA_000269985.1
GCA_000270005.1
GCA_000270025.1
GCA_000270045.1
GCA_000270065.1
GCA_000270085.1
GCA_000270105.1
GCA_000270125.2
GCA_000270145.1
GCA_000270165.1
GCA_000270205.1
GCA_000270225.1
GCA_000270245.1
GCA_000270265.1
GCA_000270285.1
GCA_000270305.1
GCA_000270345.1
GCA_000270365.1
GCA_000270385.1
GCA_000270405.1

GCA_000270425.1
GCA_000270445.1
GCA_000270465.1
GCA_000270525.1
GCA_000271325.1
GCA_000271365.1
GCA_000271405.2
GCA_000271665.2
GCA_000271865.1
GCA_000271885.2
GCA_000271905.2
GCA_000271965.2
GCA_000271985.2
GCA_000272715.4
GCA_000272735.4
GCA_000272755.4
GCA_000272775.4
GCA_000272795.2
GCA_000272815.2
GCA_000272835.4
GCA_000272895.3
GCA_000276685.1
GCA_000276705.2
GCA_000276825.1
GCA_000277025.1
GCA_000277065.1
GCA_000277085.1
GCA_000277105.1
GCA_000277125.1
GCA_000277145.1
GCA_000277165.1
GCA_000277185.1
GCA_000277205.1
GCA_000277245.1
GCA_000277265.1
GCA_000277285.1
GCA_000277305.1
GCA_000277325.1
GCA_000277345.1
GCA_000277365.1
GCA_000277385.1
GCA_000277405.1
GCA_000277425.1
GCA_000277715.1
GCA_000277735.2
GCA_000277755.1
GCA_000277775.2
GCA_000277795.1
GCA_000277895.2
GCA_000279145.1
GCA_000279165.1
GCA_000280315.2
GCA_000280495.2
GCA_000280865.2

GCA_000280925.3
GCA_000281175.1
GCA_000281195.1
GCA_000281215.1
GCA_000281235.1
GCA_000281435.2
GCA_000281535.2
GCA_000281715.2
GCA_000282175.2
GCA_000282715.1
GCA_000283275.1
GCA_000283295.1
GCA_000283475.1
GCA_000283515.1
GCA_000283535.1
GCA_000283555.1
GCA_000283575.1
GCA_000283595.1
GCA_000283615.1
GCA_000283655.1
GCA_000283695.1
GCA_000283715.1
GCA_000283755.1
GCA_000283775.1
GCA_000283795.1
GCA_000283815.1
GCA_000283875.1
GCA_000283915.1
GCA_000283935.1
GCA_000283955.1
GCA_000283975.1
GCA_000283995.1
GCA_000284015.1
GCA_000284035.1
GCA_000284055.1
GCA_000284075.1
GCA_000284095.1
GCA_000284115.1
GCA_000284135.1
GCA_000284155.1
GCA_000284175.1
GCA_000284195.1
GCA_000284215.1
GCA_000284235.1
GCA_000284255.1
GCA_000284275.1
GCA_000284295.1
GCA_000284315.1
GCA_000284335.1
GCA_000284355.1
GCA_000284375.1
GCA_000284395.1
GCA_000284415.2
GCA_000284435.1

GCA_000284455.1
GCA_000284475.1
GCA_000284495.1
GCA_000284515.1
GCA_000284535.1
GCA_000284555.1
GCA_000284575.1
GCA_000284595.1
GCA_000284615.1
GCA_000284635.1
GCA_000285655.3
GCA_000286275.1
GCA_000286435.2
GCA_000286675.1
GCA_000286695.1
GCA_000286715.1
GCA_000286735.1
GCA_000286755.1
GCA_000286775.1
GCA_000286795.1
GCA_000286815.1
GCA_000287215.3
GCA_000287235.1
GCA_000287255.1
GCA_000287275.1
GCA_000287295.1
GCA_000287315.1
GCA_000287335.1
GCA_000287355.1
GCA_000292405.1
GCA_000292415.1
GCA_000292445.1
GCA_000292455.1
GCA_000292485.1
GCA_000292505.1
GCA_000292685.1
GCA_000292705.1
GCA_000292915.1
GCA_000293765.1
GCA_000293885.3
GCA_000294365.1
GCA_000294495.1
GCA_000294515.1
GCA_000294535.1
GCA_000294635.1
GCA_000294695.2
GCA_000294715.1
GCA_000294775.2
GCA_000295655.1
GCA_000295695.2
GCA_000296215.2
GCA_000296575.1
GCA_000296595.1
GCA_000296715.2

GCA_000297055.2
GCA_000297075.2
GCA_000297155.3
GCA_000298095.1
GCA_000298115.2
GCA_000298375.2
GCA_000298385.1
GCA_000298415.1
GCA_000298435.2
GCA_000298455.2
GCA_000298475.2
GCA_000298495.2
GCA_000298515.2
GCA_000298535.2
GCA_000298555.2
GCA_000298875.1
GCA_000299015.1
GCA_000299095.1
GCA_000299115.1
GCA_000299135.1
GCA_000299235.1
GCA_000299255.1
GCA_000299335.2
GCA_000299355.1
GCA_000299435.1
GCA_000299455.1
GCA_000299475.1
GCA_000299555.2
GCA_000299955.1
GCA_000299965.1
GCA_000299995.1
GCA_000300005.1
GCA_000300035.1
GCA_000300045.1
GCA_000300075.1
GCA_000300095.1
GCA_000300135.1
GCA_000300175.1
GCA_000300235.2
GCA_000300295.4
GCA_000300435.2
GCA_000300455.4
GCA_000302475.3
GCA_000302515.1
GCA_000302535.1
GCA_000302555.4
GCA_000302575.1
GCA_000304215.1
GCA_000304315.1
GCA_000304375.1
GCA_000304415.1
GCA_000304455.1
GCA_000304495.1
GCA_000304515.1

GCA_000304535.1
GCA_000304735.1
GCA_000305335.2
GCA_000305775.1
GCA_000305785.2
GCA_000305815.1
GCA_000305935.1
GCA_000306675.3
GCA_000306745.1
GCA_000306785.1
GCA_000306805.1
GCA_000306825.1
GCA_000306845.1
GCA_000306865.1
GCA_000306885.1
GCA_000306905.1
GCA_000306945.1
GCA_000306985.1
GCA_000307005.1
GCA_000307025.1
GCA_000307045.1
GCA_000307065.1
GCA_000307085.1
GCA_000307105.1
GCA_000307125.1
GCA_000307165.1
GCA_000307185.1
GCA_000307535.1
GCA_000307585.2
GCA_000307615.1
GCA_000307795.1
GCA_000307815.1
GCA_000307835.1
GCA_000309885.1
GCA_000310065.1
GCA_000310085.1
GCA_000310105.2
GCA_000310245.1
GCA_000311765.1
GCA_000312685.1
GCA_000312705.1
GCA_000313175.2
GCA_000313385.1
GCA_000315955.1
GCA_000316175.1
GCA_000316515.1
GCA_000316575.1
GCA_000316605.1
GCA_000316625.1
GCA_000316645.1
GCA_000316665.1
GCA_000316685.1
GCA_000317025.1
GCA_000317045.1

GCA_000317065.1
GCA_000317085.1
GCA_000317105.1
GCA_000317125.1
GCA_000317305.3
GCA_000317435.1
GCA_000317475.1
GCA_000317495.1
GCA_000317515.1
GCA_000317555.1
GCA_000317575.1
GCA_000317615.1
GCA_000317635.1
GCA_000317655.1
GCA_000317675.1
GCA_000317695.1
GCA_000317835.1
GCA_000317855.1
GCA_000317875.1
GCA_000317895.1
GCA_000317915.1
GCA_000317935.1
GCA_000317955.1
GCA_000317975.2
GCA_000318015.1
GCA_000318035.1
GCA_000318055.1
GCA_000318525.2
GCA_000318565.1
GCA_000318645.1
GCA_000318765.1
GCA_000318785.1
GCA_000318825.1
GCA_000318845.1
GCA_000318865.1
GCA_000318885.1
GCA_000318905.1
GCA_000318925.1
GCA_000318985.1
GCA_000319105.1
GCA_000319225.1
GCA_000319245.1
GCA_000319385.1
GCA_000319475.1
GCA_000319575.2
GCA_000319655.2
GCA_000319675.2
GCA_000320385.2
GCA_000321395.1
GCA_000321415.2
GCA_000325665.1
GCA_000325705.1
GCA_000325725.1
GCA_000325745.1

GCA_000327045.1
GCA_000327065.1
GCA_000327395.2
GCA_000328405.1
GCA_000328545.1
GCA_000328565.1
GCA_000328625.1
GCA_000328705.1
GCA_000328725.1
GCA_000328765.2
GCA_000329365.2
GCA_000330485.2
GCA_000330845.1
GCA_000330865.1
GCA_000330885.1
GCA_000331065.1
GCA_000331085.2
GCA_000331165.2
GCA_000331185.2
GCA_000331445.1
GCA_000331695.1
GCA_000331715.1
GCA_000331735.1
GCA_000331975.1
GCA_000331995.1
GCA_000332115.1
GCA_000332755.1
GCA_000333835.2
GCA_000334405.1
GCA_000334515.1
GCA_000334875.3
GCA_000335875.2
GCA_000335995.2
GCA_000336405.1
GCA_000336425.1
GCA_000336445.1
GCA_000336465.1
GCA_000338095.1
GCA_000338115.2
GCA_000338695.1
GCA_000338715.2
GCA_000338735.1
GCA_000338755.1
GCA_000339015.1
GCA_000340435.3
GCA_000340785.1
GCA_000340795.1
GCA_000340825.1
GCA_000340845.1
GCA_000340865.1
GCA_000340885.1
GCA_000340905.1
GCA_000340925.1
GCA_000341345.1

GCA_000341355.1
GCA_000341385.1
GCA_000341395.1
GCA_000341425.1
GCA_000341465.2
GCA_000341485.2
GCA_000341655.1
GCA_000341665.1
GCA_000341695.1
GCA_000341855.1
GCA_000341875.1
GCA_000342265.1
GCA_000344335.2
GCA_000344355.2
GCA_000344475.3
GCA_000344575.1
GCA_000344745.1
GCA_000344765.1
GCA_000344785.1
GCA_000344805.1
GCA_000346065.1
GCA_000346225.2
GCA_000346595.1
GCA_000347595.1
GCA_000347615.1
GCA_000347635.1
GCA_000347675.2
GCA_000347695.1
GCA_000348565.1
GCA_000348585.1
GCA_000348725.1
GCA_000348745.1
GCA_000348765.2
GCA_000348785.1
GCA_000348805.1
GCA_000348865.1
GCA_000348885.1
GCA_000349225.1
GCA_000349745.1
GCA_000349765.2
GCA_000349785.2
GCA_000349795.1
GCA_000349845.1
GCA_000349885.2
GCA_000349925.2
GCA_000349945.1
GCA_000349975.1
GCA_000350185.1
GCA_000350205.1
GCA_000354175.2
GCA_000355675.1
GCA_000355695.1
GCA_000355765.4
GCA_000359505.1

GCA_000359525.1
GCA_000359545.5
GCA_000359625.1
GCA_000363905.1
GCA_000364165.2
GCA_000364205.2
GCA_000364385.3
GCA_000364445.2
GCA_000364725.1
GCA_000364765.1
GCA_000364785.1
GCA_000364805.1
GCA_000367205.1
GCA_000367405.1
GCA_000367425.1
GCA_000367725.3
GCA_000376545.2
GCA_000376585.1
GCA_000376605.1
GCA_000376625.1
GCA_000376645.1
GCA_000376705.1
GCA_000380335.1
GCA_000380365.1
GCA_000382585.2
GCA_000382905.1
GCA_000382925.1
GCA_000382945.1
GCA_000382965.1
GCA_000382985.1
GCA_000383005.1
GCA_000385525.1
GCA_000385905.1
GCA_000385925.1
GCA_000385945.1
GCA_000387745.2
GCA_000389635.1
GCA_000389675.2
GCA_000389905.1
GCA_000389925.1
GCA_000389945.1
GCA_000389965.1
GCA_000390085.1
GCA_000390245.1
GCA_000390265.1
GCA_000391485.2
GCA_000392435.1
GCA_000392455.3
GCA_000392475.3
GCA_000392485.2
GCA_000392515.3
GCA_000392535.3
GCA_000397165.1
GCA_000397185.1

GCA_000397205.1
GCA_000400615.1
GCA_000400635.2
GCA_000400855.1
GCA_000400875.1
GCA_000400935.1
GCA_000400955.1
GCA_000401175.1
GCA_000401555.1
GCA_000401735.2
GCA_000402035.1
GCA_000403575.2
GCA_000403625.1
GCA_000404145.1
GCA_000404185.1
GCA_000404205.1
GCA_000404245.1
GCA_000406765.2
GCA_000408885.1
GCA_000410515.1
GCA_000410575.1
GCA_000410995.1
GCA_000412205.1
GCA_000412265.2
GCA_000412675.1
GCA_000412695.1
GCA_000412755.1
GCA_000412775.1
GCA_000414035.1
GCA_000414215.1
GCA_000417225.2
GCA_000417265.2
GCA_000418305.1
GCA_000418325.1
GCA_000418345.1
GCA_000418365.1
GCA_000418475.1
GCA_000418495.1
GCA_000418515.1
GCA_000418535.2
GCA_000419385.1
GCA_000419405.1
GCA_000419425.1
GCA_000422045.1
GCA_000422085.1
GCA_000422095.1
GCA_000422125.1
GCA_000422145.1
GCA_000422165.1
GCA_000427035.1
GCA_000427055.1
GCA_000427075.1
GCA_000427215.1
GCA_000427275.1

GCA_000430085.2
GCA_000430105.1
GCA_000430125.1
GCA_000430145.3
GCA_000430165.1
GCA_000430385.1
GCA_000430405.1
GCA_000430425.1
GCA_000430465.3
GCA_000438585.1
GCA_000438605.1
GCA_000438625.1
GCA_000438645.1
GCA_000438665.2
GCA_000438685.2
GCA_000438705.2
GCA_000438725.2
GCA_000438745.2
GCA_000438825.1
GCA_000439255.1
GCA_000439275.1
GCA_000439295.2
GCA_000439395.1
GCA_000439415.2
GCA_000439435.1
GCA_000439455.1
GCA_000439475.1
GCA_000439495.1
GCA_000439535.1
GCA_000439575.1
GCA_000439595.1
GCA_000439695.1
GCA_000439735.1
GCA_000439755.1
GCA_000439775.1
GCA_000439915.2
GCA_000441535.1
GCA_000441555.1
GCA_000441575.1
GCA_000441585.1
GCA_000441615.1
GCA_000441635.1
GCA_000441655.1
GCA_000441675.1
GCA_000441695.1
GCA_000441715.1
GCA_000441735.1
GCA_000441755.1
GCA_000441775.1
GCA_000441795.1
GCA_000441815.1
GCA_000442415.1
GCA_000442435.1
GCA_000442595.1

GCA_000442605.1
GCA_000442635.1
GCA_000442645.1
GCA_000444405.1
GCA_000444425.1
GCA_000444445.1
GCA_000444465.1
GCA_000444875.1
GCA_000444995.1
GCA_000445015.1
GCA_000445035.1
GCA_000445425.4
GCA_000445995.2
GCA_000447675.1
GCA_000448685.2
GCA_000452385.2
GCA_000452445.3
GCA_000452525.4
GCA_000452565.3
GCA_000452605.3
GCA_000452705.3
GCA_000452745.3
GCA_000454025.1
GCA_000454045.1
GCA_000455565.1
GCA_000455585.1
GCA_000455605.1
GCA_000462955.1
GCA_000462975.1
GCA_000462995.1
GCA_000463015.1
GCA_000463055.1
GCA_000463345.3
GCA_000463355.1
GCA_000463385.1
GCA_000463395.1
GCA_000463425.1
GCA_000463445.1
GCA_000463465.1
GCA_000463505.1
GCA_000464435.1
GCA_000464515.2
GCA_000464955.2
GCA_000465235.1
GCA_000465255.1
GCA_000465465.2
GCA_000465975.2
GCA_000466065.2
GCA_000466075.2
GCA_000466105.2
GCA_000466785.3
GCA_000466885.3
GCA_000466905.3
GCA_000466925.3

GCA_000468515.1
GCA_000468575.2
GCA_000468615.2
GCA_000468915.2
GCA_000468955.1
GCA_000469135.2
GCA_000470765.1
GCA_000470775.1
GCA_000470805.1
GCA_000470825.1
GCA_000470845.1
GCA_000470865.1
GCA_000470975.2
GCA_000471025.2
GCA_000471925.1
GCA_000471945.1
GCA_000471965.1
GCA_000471985.1
GCA_000473245.1
GCA_000473275.1
GCA_000473305.1
GCA_000473745.3
GCA_000473995.1
GCA_000474015.1
GCA_000474035.1
GCA_000477035.2
GCA_000477415.1
GCA_000477435.1
GCA_000478255.2
GCA_000478825.2
GCA_000478885.1
GCA_000478905.1
GCA_000478925.1
GCA_000479315.1
GCA_000479335.1
GCA_000479355.1
GCA_000483715.2
GCA_000484195.3
GCA_000484505.2
GCA_000484535.1
GCA_000485885.1
GCA_000485905.1
GCA_000486045.2
GCA_000486165.2
GCA_000486345.2
GCA_000486365.2
GCA_000486405.2
GCA_000486445.2
GCA_000486585.2
GCA_000486725.2
GCA_000486765.2
GCA_000486815.2
GCA_000486855.2
GCA_000486915.2

GCA_000487015.2
GCA_000487035.2
GCA_000487155.2
GCA_000487255.2
GCA_000487295.3
GCA_000487515.2
GCA_000487575.2
GCA_000487615.2
GCA_000487775.2
GCA_000487915.2
GCA_000487935.2
GCA_000487995.2
GCA_000492175.2
GCA_000493295.2
GCA_000493375.1
GCA_000493495.1
GCA_000493535.2
GCA_000493675.1
GCA_000493735.1
GCA_000493755.1
GCA_000493775.1
GCA_000494385.3
GCA_000494755.1
GCA_000494775.2
GCA_000494835.1
GCA_000494855.1
GCA_000494875.1
GCA_000494895.1
GCA_000494915.1
GCA_000495455.2
GCA_000495635.2
GCA_000495935.2
GCA_000496265.1
GCA_000496285.1
GCA_000496455.2
GCA_000496595.1
GCA_000496605.2
GCA_000496635.1
GCA_000496645.1
GCA_000497265.2
GCA_000497485.1
GCA_000497505.1
GCA_000497525.2
GCA_000498315.1
GCA_000498335.1
GCA_000498375.2
GCA_000498395.3
GCA_000498655.1
GCA_000498675.1
GCA_000498975.2
GCA_000499365.1
GCA_000499485.1
GCA_000499645.1
GCA_000499665.2

GCA_000499805.2
GCA_000500935.1
GCA_000503835.1
GCA_000503845.1
GCA_000503875.1
GCA_000503895.1
GCA_000503915.1
GCA_000504045.1
GCA_000504085.1
GCA_000504105.1
GCA_000504125.1
GCA_000504545.1
GCA_000504585.2
GCA_000505365.2
GCA_000505685.2
GCA_000505705.1
GCA_000505725.1
GCA_000506785.4
GCA_000507205.2
GCA_000507225.1
GCA_000507245.1
GCA_000508125.2
GCA_000508205.1
GCA_000508225.1
GCA_000508245.1
GCA_000508265.1
GCA_000508765.1
GCA_000510265.1
GCA_000510285.1
GCA_000510305.1
GCA_000510325.1
GCA_000511285.2
GCA_000511305.1
GCA_000511325.1
GCA_000511355.1
GCA_000511385.1
GCA_000511405.1
GCA_000511895.1
GCA_000511915.1
GCA_000512125.1
GCA_000512145.2
GCA_000512165.1
GCA_000512205.2
GCA_000512355.1
GCA_000512375.1
GCA_000512395.1
GCA_000512735.1
GCA_000512745.2
GCA_000512775.2
GCA_000512835.2
GCA_000512895.1
GCA_000512915.1
GCA_000512955.1
GCA_000513215.1

GCA_000513295.1
GCA_000513475.1
GCA_000513595.1
GCA_000513615.1
GCA_000513635.1
GCA_000513655.1
GCA_000513675.1
GCA_000513695.1
GCA_000517305.1
GCA_000517365.1
GCA_000517405.1
GCA_000517425.1
GCA_000517605.1
GCA_000520015.2
GCA_000520035.1
GCA_000520055.1
GCA_000520875.1
GCA_000520895.1
GCA_000521505.1
GCA_000521525.1
GCA_000521545.1
GCA_000521565.1
GCA_000521585.1
GCA_000521605.1
GCA_000521645.1
GCA_000521655.1
GCA_000521685.1
GCA_000521695.1
GCA_000521725.1
GCA_000521745.1
GCA_000521765.1
GCA_000522545.2
GCA_000522985.1
GCA_000523045.1
GCA_000523235.1
GCA_000524555.1
GCA_000524575.1
GCA_000524595.1
GCA_000525635.1
GCA_000525675.1
GCA_000525715.1
GCA_000530755.1
GCA_000534935.2
GCA_000550745.1
GCA_000550765.1
GCA_000550785.1
GCA_000550805.1
GCA_000565155.1
GCA_000565175.1
GCA_000565195.1
GCA_000565215.1
GCA_000567905.1
GCA_000567925.1
GCA_000567945.1

GCA_000568455.1
GCA_000568475.1
GCA_000568495.1
GCA_000568815.1
GCA_000568855.2
GCA_000568935.1
GCA_000568955.1
GCA_000568975.1
GCA_000569015.1
GCA_000569035.1
GCA_000569055.1
GCA_000569075.1
GCA_000572125.1
GCA_000572155.1
GCA_000572175.1
GCA_000572195.1
GCA_000576085.2
GCA_000576125.1
GCA_000576145.2
GCA_000576165.1
GCA_000576185.2
GCA_000576555.1
GCA_000577745.1
GCA_000577895.1
GCA_000582515.1
GCA_000582535.1
GCA_000582555.1
GCA_000582665.1
GCA_000582845.1
GCA_000583065.1
GCA_000583105.1
GCA_000583755.1
GCA_000583775.1
GCA_000583795.1
GCA_000583835.1
GCA_000583855.1
GCA_000583875.1
GCA_000585995.1
GCA_000590455.1
GCA_000590475.1
GCA_000590495.2
GCA_000590535.2
GCA_000590555.1
GCA_000590575.1
GCA_000590615.1
GCA_000590635.1
GCA_000590675.1
GCA_000590755.1
GCA_000590775.1
GCA_000590795.1
GCA_000590815.1
GCA_000590865.3
GCA_000590925.1
GCA_000597785.2

GCA_000597845.1
GCA_000597865.1
GCA_000597885.1
GCA_000597905.1
GCA_000597945.2
GCA_000597965.1
GCA_000597985.1
GCA_000598005.1
GCA_000599625.1
GCA_000599645.1
GCA_000599665.1
GCA_000599685.1
GCA_000599705.1
GCA_000599965.1
GCA_000599985.1
GCA_000600005.1
GCA_000600015.1
GCA_000600045.1
GCA_000600055.1
GCA_000600085.1
GCA_000600105.1
GCA_000600125.1
GCA_000600145.1
GCA_000600165.1
GCA_000600185.1
GCA_000600205.1
GCA_000600225.1
GCA_000604045.1
GCA_000604065.3
GCA_000604105.1
GCA_000604125.1
GCA_000612055.1
GCA_000612325.1
GCA_000612485.1
GCA_000612505.1
GCA_000612685.1
GCA_000612765.1
GCA_000612965.1
GCA_000613085.1
GCA_000618005.3
GCA_000618905.3
GCA_000619905.2
GCA_000623055.2
GCA_000623095.2
GCA_000623115.2
GCA_000623135.2
GCA_000623195.2
GCA_000623275.1
GCA_000623295.1
GCA_000623315.1
GCA_000623335.1
GCA_000623355.1
GCA_000623375.1
GCA_000623395.2

GCA_000623455.2
GCA_000623475.2
GCA_000623615.2
GCA_000623655.2
GCA_000623675.2
GCA_000623715.2
GCA_000623735.2
GCA_000624055.2
GCA_000624175.2
GCA_000624395.2
GCA_000625855.2
GCA_000626095.2
GCA_000626175.1
GCA_000626195.1
GCA_000626215.1
GCA_000626235.1
GCA_000626255.1
GCA_000626275.2
GCA_000626295.1
GCA_000626335.1
GCA_000626355.1
GCA_000626375.1
GCA_000626595.1
GCA_000626615.3
GCA_000626635.1
GCA_000626675.1
GCA_000626695.2
GCA_000632395.1
GCA_000632415.1
GCA_000632435.1
GCA_000632475.2
GCA_000632815.1
GCA_000632845.1
GCA_000632865.1
GCA_000632885.1
GCA_000632905.1
GCA_000632925.1
GCA_000632945.1
GCA_000632965.1
GCA_000632985.1
GCA_000633175.1
GCA_000635895.2
GCA_000635915.2
GCA_000635955.1
GCA_000636115.1
GCA_000636135.1
GCA_000648235.2
GCA_000648515.1
GCA_000648525.1
GCA_000648555.1
GCA_000648735.3
GCA_000661895.1
GCA_000661915.1
GCA_000662395.1

GCA_000671295.1
GCA_000681515.2
GCA_000685625.1
GCA_000685665.1
GCA_000685705.1
GCA_000685725.1
GCA_000685745.1
GCA_000688775.2
GCA_000688795.1
GCA_000689235.1
GCA_000689355.1
GCA_000689415.1
GCA_000689635.2
GCA_000691545.1
GCA_000691565.1
GCA_000691585.1
GCA_000691605.1
GCA_000695095.2
GCA_000695215.1
GCA_000695235.1
GCA_000695835.1
GCA_000695855.3
GCA_000695875.1
GCA_000695895.1
GCA_000695935.1
GCA_000695975.1
GCA_000695995.1
GCA_000696015.1
GCA_000696345.2
GCA_000696465.1
GCA_000696485.1
GCA_000696505.1
GCA_000696675.2
GCA_000697965.2
GCA_000698245.1
GCA_000698285.1
GCA_000698325.1
GCA_000698345.1
GCA_000698365.1
GCA_000698475.1
GCA_000698515.2
GCA_000698715.2
GCA_000698805.1
GCA_000698825.1
GCA_000698845.1
GCA_000698865.1
GCA_000698885.1
GCA_000699025.1
GCA_000699465.1
GCA_000699475.1
GCA_000699505.1
GCA_000699525.1
GCA_000701165.2
GCA_000705535.2

GCA_000705595.2
GCA_000706665.1
GCA_000706685.1
GCA_000706705.1
GCA_000706725.1
GCA_000706745.1
GCA_000706765.1
GCA_000709415.1
GCA_000709435.1
GCA_000709455.1
GCA_000709475.1
GCA_000709495.1
GCA_000709535.1
GCA_000709555.1
GCA_000710365.3
GCA_000710375.3
GCA_000714595.1
GCA_000714635.1
GCA_000714655.1
GCA_000714675.1
GCA_000715155.2
GCA_000717515.1
GCA_000717535.1
GCA_000722765.2
GCA_000723165.1
GCA_000723365.1
GCA_000723425.2
GCA_000723465.1
GCA_000723505.1
GCA_000723785.1
GCA_000724485.1
GCA_000724505.1
GCA_000724525.1
GCA_000724585.1
GCA_000724605.1
GCA_000724625.1
GCA_000724665.3
GCA_000724775.3
GCA_000724795.2
GCA_000724805.2
GCA_000724815.2
GCA_000725265.1
GCA_000725285.1
GCA_000725305.1
GCA_000725325.1
GCA_000725345.1
GCA_000725365.1
GCA_000725385.1
GCA_000725405.1
GCA_000730165.2
GCA_000730205.1
GCA_000730215.2
GCA_000730245.1
GCA_000730255.1

GCA_000730345.1
GCA_000730365.1
GCA_000730385.1
GCA_000730405.1
GCA_000730425.1
GCA_000730445.1
GCA_000731295.1
GCA_000731315.1
GCA_000732045.2
GCA_000732355.1
GCA_000732385.1
GCA_000732425.1
GCA_000732525.2
GCA_000732905.1
GCA_000732925.1
GCA_000732945.1
GCA_000732965.1
GCA_000733255.1
GCA_000733715.2
GCA_000733995.1
GCA_000734015.1
GCA_000734055.1
GCA_000734895.2
GCA_000734975.2
GCA_000735125.2
GCA_000736415.1
GCA_000737085.1
GCA_000737305.2
GCA_000737325.2
GCA_000737405.1
GCA_000737515.1
GCA_000737535.1
GCA_000737555.1
GCA_000737575.1
GCA_000737595.1
GCA_000737615.1
GCA_000737865.1
GCA_000737885.1
GCA_000738045.1
GCA_000738065.1
GCA_000738435.1
GCA_000738445.1
GCA_000738475.1
GCA_000739085.1
GCA_000739105.1
GCA_000739375.1
GCA_000739395.1
GCA_000739435.1
GCA_000739455.1
GCA_000739475.1
GCA_000739495.1
GCA_000740135.1
GCA_000740155.1
GCA_000740175.1

GCA_000740195.1
GCA_000740215.1
GCA_000740235.1
GCA_000740255.1
GCA_000740275.1
GCA_000740295.1
GCA_000740315.1
GCA_000740335.1
GCA_000740355.1
GCA_000740375.1
GCA_000740415.1
GCA_000740435.1
GCA_000740455.1
GCA_000740965.1
GCA_000740985.1
GCA_000741005.1
GCA_000742655.1
GCA_000742715.1
GCA_000742735.1
GCA_000742755.1
GCA_000742795.1
GCA_000742815.1
GCA_000742835.1
GCA_000742855.1
GCA_000742895.1
GCA_000742955.1
GCA_000742975.1
GCA_000743015.1
GCA_000743035.1
GCA_000743055.1
GCA_000743255.1
GCA_000743945.1
GCA_000743955.1
GCA_000743995.1
GCA_000746025.2
GCA_000746505.1
GCA_000746525.1
GCA_000746585.2
GCA_000746605.1
GCA_000746625.1
GCA_000746645.1
GCA_000746665.1
GCA_000747295.1
GCA_000747315.1
GCA_000747335.1
GCA_000747375.1
GCA_000747525.1
GCA_000747545.1
GCA_000747565.1
GCA_000747585.1
GCA_000748245.2
GCA_000748565.2
GCA_000750195.1
GCA_000750215.1

GCA_000750225.1
GCA_000750255.1
GCA_000750275.1
GCA_000750295.1
GCA_000750315.1
GCA_000750335.1
GCA_000750375.1
GCA_000750395.2
GCA_000750415.2
GCA_000750435.1
GCA_000750455.1
GCA_000750475.1
GCA_000750495.1
GCA_000750515.1
GCA_000750535.1
GCA_000750555.1
GCA_000751035.1
GCA_000754265.1
GCA_000754275.1
GCA_000754305.1
GCA_000754345.1
GCA_000754365.1
GCA_000754375.1
GCA_000754405.1
GCA_000755085.1
GCA_000755105.1
GCA_000755145.1
GCA_000755185.1
GCA_000755585.2
GCA_000755705.1
GCA_000755725.1
GCA_000755765.1
GCA_000755785.1
GCA_000755805.1
GCA_000755825.1
GCA_000755845.1
GCA_000755865.1
GCA_000755885.1
GCA_000755905.1
GCA_000755925.1
GCA_000755945.1
GCA_000755965.1
GCA_000755985.1
GCA_000756005.1
GCA_000756025.2
GCA_000756045.1
GCA_000756065.1
GCA_000756085.1
GCA_000756125.1
GCA_000756145.1
GCA_000756165.1
GCA_000756185.1
GCA_000756205.1
GCA_000756415.3

GCA_000756435.3
GCA_000756465.1
GCA_000756485.1
GCA_000756505.1
GCA_000756525.1
GCA_000756545.1
GCA_000756615.1
GCA_000757015.2
GCA_000757035.2
GCA_000757785.1
GCA_000757795.1
GCA_000757825.1
GCA_000757845.1
GCA_000757865.1
GCA_000758305.1
GCA_000758325.1
GCA_000758345.1
GCA_000758365.1
GCA_000758525.1
GCA_000758545.1
GCA_000758565.1
GCA_000758585.1
GCA_000758605.1
GCA_000758625.1
GCA_000758645.1
GCA_000758665.1
GCA_000758685.1
GCA_000758705.1
GCA_000758725.1
GCA_000759375.2
GCA_000759475.1
GCA_000759485.1
GCA_000759515.1
GCA_000759535.1
GCA_000759555.1
GCA_000759575.2
GCA_000760905.2
GCA_000761115.1
GCA_000761135.1
GCA_000761155.1
GCA_000761175.1
GCA_000761195.1
GCA_000761215.1
GCA_000762285.1
GCA_000763245.3
GCA_000763325.2
GCA_000763475.1
GCA_000763495.1
GCA_000763515.1
GCA_000763535.2
GCA_000763575.1
GCA_000764045.2
GCA_000764535.1
GCA_000764555.1

GCA_000764575.1
GCA_000764595.1
GCA_000764615.1
GCA_000764955.1
GCA_000765375.1
GCA_000765395.1
GCA_000765415.1
GCA_000766665.1
GCA_000766885.2
GCA_000767055.1
GCA_000767075.1
GCA_000767095.1
GCA_000767275.4
GCA_000767405.1
GCA_000767415.1
GCA_000767445.1
GCA_000767465.1
GCA_000767505.1
GCA_000767605.1
GCA_000767615.3
GCA_000767645.1
GCA_000767665.1
GCA_000767685.1
GCA_000767705.1
GCA_000767725.1
GCA_000767745.1
GCA_000769405.2
GCA_000769535.1
GCA_000769555.1
GCA_000769575.1
GCA_000769635.1
GCA_000770115.2
GCA_000770155.1
GCA_000770195.1
GCA_000770355.2
GCA_000770395.1
GCA_000770455.1
GCA_000770495.1
GCA_000770515.1
GCA_000770535.1
GCA_000770565.1
GCA_000772025.1
GCA_000772045.1
GCA_000772065.1
GCA_000772085.1
GCA_000772105.1
GCA_000772125.1
GCA_000772145.1
GCA_000772185.1
GCA_000772225.1
GCA_000772245.1
GCA_000772485.1
GCA_000772715.2
GCA_000772775.3

GCA_000774075.2
GCA_000775355.2
GCA_000775375.1
GCA_000775395.1
GCA_000775955.1
GCA_000775975.1
GCA_000775995.1
GCA_000782835.1
GCA_000782855.1
GCA_000783435.2
GCA_000783575.2
GCA_000783595.2
GCA_000783675.2
GCA_000783735.2
GCA_000783775.2
GCA_000783815.2
GCA_000783875.2
GCA_000783915.2
GCA_000783935.2
GCA_000783955.2
GCA_000783995.2
GCA_000784015.2
GCA_000784865.1
GCA_000784905.1
GCA_000784925.1
GCA_000784945.1
GCA_000784985.1
GCA_000785005.1
GCA_000785105.2
GCA_000785495.1
GCA_000785515.1
GCA_000785535.1
GCA_000785555.1
GCA_000785705.2
GCA_000786425.1
GCA_000786505.1
GCA_000786695.1
GCA_000786735.1
GCA_000787195.2
GCA_000788395.1
GCA_000788715.2
GCA_000789275.1
GCA_000789295.1
GCA_000789315.1
GCA_000789335.1
GCA_000789355.1
GCA_000789375.1
GCA_000789395.1
GCA_000794125.2
GCA_000798935.2
GCA_000800215.1
GCA_000800235.1
GCA_000800255.1
GCA_000800275.1

GCA_000800295.1
GCA_000800315.1
GCA_000800335.1
GCA_000800355.1
GCA_000800395.1
GCA_000800415.1
GCA_000800435.1
GCA_000800455.1
GCA_000800475.2
GCA_000800725.2
GCA_000800765.1
GCA_000800785.1
GCA_000800825.1
GCA_000800845.2
GCA_000801145.1
GCA_000801165.1
GCA_000801185.2
GCA_000801205.1
GCA_000801275.1
GCA_000801295.1
GCA_000801315.1
GCA_000801755.2
GCA_000802245.2
GCA_000803625.1
GCA_000803645.1
GCA_000803665.1
GCA_000803705.1
GCA_000807175.1
GCA_000807225.1
GCA_000807255.1
GCA_000807275.1
GCA_000807295.1
GCA_000807315.1
GCA_000807355.1
GCA_000807375.1
GCA_000807405.4
GCA_000807415.4
GCA_000807425.4
GCA_000807675.2
GCA_000807775.2
GCA_000807785.2
GCA_000808035.2
GCA_000808055.1
GCA_000808095.1
GCA_000812165.1
GCA_000812205.2
GCA_000812665.2
GCA_000813165.1
GCA_000813205.1
GCA_000814125.3
GCA_000814145.2
GCA_000814165.3
GCA_000814205.1
GCA_000814225.1

GCA_000814265.1
GCA_000814305.1
GCA_000814345.1
GCA_000814805.1
GCA_000814825.1
GCA_000814845.2
GCA_000814865.1
GCA_000815025.1
GCA_000815045.1
GCA_000815065.1
GCA_000815085.1
GCA_000815105.2
GCA_000815125.1
GCA_000815145.1
GCA_000815165.1
GCA_000815185.1
GCA_000815205.1
GCA_000815225.1
GCA_000815245.1
GCA_000815275.2
GCA_000816025.1
GCA_000816045.1
GCA_000816065.1
GCA_000816085.1
GCA_000816125.1
GCA_000816145.1
GCA_000816165.1
GCA_000816185.1
GCA_000816205.1
GCA_000816225.1
GCA_000816245.1
GCA_000816265.1
GCA_000816305.1
GCA_000816345.1
GCA_000816365.1
GCA_000816385.2
GCA_000816405.1
GCA_000816425.1
GCA_000816465.4
GCA_000816785.1
GCA_000816845.1
GCA_000816885.1
GCA_000816945.1
GCA_000816985.1
GCA_000817005.1
GCA_000817025.1
GCA_000817045.1
GCA_000817065.1
GCA_000817325.1
GCA_000817935.1
GCA_000817955.1
GCA_000817975.1
GCA_000817995.1
GCA_000818015.1

GCA_000818035.1
GCA_000818055.1
GCA_000818075.1
GCA_000818095.1
GCA_000818115.1
GCA_000819445.1
GCA_000819505.1
GCA_000819525.2
GCA_000819565.1
GCA_000819645.1
GCA_000819665.1
GCA_000821185.2
GCA_000826835.2
GCA_000826905.1
GCA_000826925.1
GCA_000826965.4
GCA_000826985.1
GCA_000827005.1
GCA_000827025.1
GCA_000827045.1
GCA_000827065.1
GCA_000827085.1
GCA_000827105.1
GCA_000827125.1
GCA_000827855.1
GCA_000827935.1
GCA_000827955.1
GCA_000828015.1
GCA_000828035.1
GCA_000828055.2
GCA_000828475.1
GCA_000828515.1
GCA_000828615.1
GCA_000828635.1
GCA_000828655.1
GCA_000828675.1
GCA_000828695.1
GCA_000828715.1
GCA_000828735.1
GCA_000828775.1
GCA_000828795.1
GCA_000828815.1
GCA_000828835.1
GCA_000828855.1
GCA_000828895.1
GCA_000828915.1
GCA_000828935.1
GCA_000828955.1
GCA_000828975.1
GCA_000828995.1
GCA_000829015.1
GCA_000829035.1
GCA_000829055.1
GCA_000829075.1

GCA_000829095.1
GCA_000829115.1
GCA_000829135.1
GCA_000829155.1
GCA_000829175.1
GCA_000829195.1
GCA_000829215.1
GCA_000829235.1
GCA_000829255.1
GCA_000829275.1
GCA_000829295.1
GCA_000829315.1
GCA_000829335.1
GCA_000829355.1
GCA_000829375.1
GCA_000829395.1
GCA_000829415.1
GCA_000829885.1
GCA_000829985.1
GCA_000830005.1
GCA_000830035.1
GCA_000830055.1
GCA_000830775.1
GCA_000830805.1
GCA_000830825.1
GCA_000830845.1
GCA_000830865.1
GCA_000830885.1
GCA_000830905.1
GCA_000830925.1
GCA_000830945.1
GCA_000830965.1
GCA_000830985.1
GCA_000831005.1
GCA_000831065.1
GCA_000831105.1
GCA_000831125.1
GCA_000831145.1
GCA_000831165.1
GCA_000831185.1
GCA_000831205.1
GCA_000831225.1
GCA_000831405.1
GCA_000831425.1
GCA_000831465.1
GCA_000831485.1
GCA_000831525.1
GCA_000831545.1
GCA_000831565.1
GCA_000831585.1
GCA_000831645.3
GCA_000831935.2
GCA_000832145.1
GCA_000832305.1

GCA_000832385.1
GCA_000832405.1
GCA_000832425.1
GCA_000832445.1
GCA_000832465.1
GCA_000832485.1
GCA_000832505.1
GCA_000832525.1
GCA_000832565.1
GCA_000832585.1
GCA_000832605.1
GCA_000832635.1
GCA_000832665.1
GCA_000832725.1
GCA_000832745.1
GCA_000832765.1
GCA_000832785.1
GCA_000832805.1
GCA_000832825.1
GCA_000832845.1
GCA_000832865.1
GCA_000832885.1
GCA_000832905.1
GCA_000832925.1
GCA_000832965.1
GCA_000832985.1
GCA_000833005.1
GCA_000833025.1
GCA_000833045.1
GCA_000833065.1
GCA_000833085.1
GCA_000833105.2
GCA_000833125.1
GCA_000833145.1
GCA_000833165.1
GCA_000833195.1
GCA_000833215.1
GCA_000833235.1
GCA_000833255.1
GCA_000833275.1
GCA_000833295.1
GCA_000833315.1
GCA_000833335.1
GCA_000833355.1
GCA_000833375.1
GCA_000833455.1
GCA_000833475.1
GCA_000833495.1
GCA_000833515.1
GCA_000833535.1
GCA_000833575.1
GCA_000833635.2
GCA_000834195.1
GCA_000834215.1

GCA_000834235.1
GCA_000834255.1
GCA_000834275.1
GCA_000834295.1
GCA_000834315.1
GCA_000834335.1
GCA_000834355.1
GCA_000834375.1
GCA_000834395.1
GCA_000834415.1
GCA_000834435.1
GCA_000834455.1
GCA_000834475.1
GCA_000834495.1
GCA_000834515.1
GCA_000834735.1
GCA_000834755.1
GCA_000834775.1
GCA_000834825.1
GCA_000834845.1
GCA_000834865.1
GCA_000834885.1
GCA_000834925.1
GCA_000834945.1
GCA_000834965.1
GCA_000834985.1
GCA_000835025.1
GCA_000835085.1
GCA_000835145.1
GCA_000835165.1
GCA_000835185.1
GCA_000835205.1
GCA_000835225.1
GCA_000835265.1
GCA_000835285.1
GCA_000835305.1
GCA_000835345.1
GCA_000835365.1
GCA_000875675.1
GCA_000875695.1
GCA_000875715.1
GCA_000875755.1
GCA_000876525.1
GCA_000876545.1
GCA_000876675.2
GCA_000877815.1
GCA_000931425.2
GCA_000931445.1
GCA_000931465.1
GCA_000931565.1
GCA_000931575.1
GCA_000931605.1
GCA_000931625.1
GCA_000932055.2

GCA_000934305.1
GCA_000934325.3
GCA_000934525.1
GCA_000934545.1
GCA_000934565.1
GCA_000934605.2
GCA_000934625.1
GCA_000935025.1
GCA_000935185.3
GCA_000939415.2
GCA_000940785.1
GCA_000940805.1
GCA_000940825.1
GCA_000940845.1
GCA_000940895.2
GCA_000940915.1
GCA_000940935.2
GCA_000940975.1
GCA_000940995.1
GCA_000941015.2
GCA_000941035.1
GCA_000941055.1
GCA_000941075.1
GCA_000941635.2
GCA_000943515.2
GCA_000943805.1
GCA_000947915.1
GCA_000948075.1
GCA_000948975.2
GCA_000948985.2
GCA_000949425.1
GCA_000950575.1
GCA_000950775.1
GCA_000952035.2
GCA_000952895.1
GCA_000952915.1
GCA_000952935.1
GCA_000952955.1
GCA_000952975.1
GCA_000953015.1
GCA_000953035.1
GCA_000953095.1
GCA_000953135.1
GCA_000953195.1
GCA_000953215.1
GCA_000953235.1
GCA_000953255.1
GCA_000953275.1
GCA_000953295.1
GCA_000953315.1
GCA_000953355.1
GCA_000953375.1
GCA_000953395.1
GCA_000953415.1

GCA_000953435.1
GCA_000953455.1
GCA_000953475.1
GCA_000953495.1
GCA_000953515.1
GCA_000953535.1
GCA_000953575.1
GCA_000953615.1
GCA_000953635.1
GCA_000953655.1
GCA_000953695.1
GCA_000953715.1
GCA_000953735.1
GCA_000954115.1
GCA_000954135.2
GCA_000954155.1
GCA_000954175.1
GCA_000954195.1
GCA_000955665.1
GCA_000956315.1
GCA_000958465.1
GCA_000959125.1
GCA_000959145.1
GCA_000959165.1
GCA_000959185.1
GCA_000959205.1
GCA_000959225.1
GCA_000959245.1
GCA_000959265.1
GCA_000959285.1
GCA_000959305.1
GCA_000959325.1
GCA_000959345.1
GCA_000959365.1
GCA_000959405.1
GCA_000959425.1
GCA_000959445.1
GCA_000959465.1
GCA_000959485.1
GCA_000959505.1
GCA_000959525.1
GCA_000959545.1
GCA_000959605.1
GCA_000959625.1
GCA_000959705.2
GCA_000959725.1
GCA_000960975.1
GCA_000960995.1
GCA_000961015.1
GCA_000961095.1
GCA_000961155.1
GCA_000961175.1
GCA_000961195.1
GCA_000961215.1

GCA_000961235.1
GCA_000961255.1
GCA_000961275.1
GCA_000961295.1
GCA_000961315.1
GCA_000961335.1
GCA_000961355.1
GCA_000961375.1
GCA_000961395.1
GCA_000961415.1
GCA_000961435.1
GCA_000961455.1
GCA_000961475.1
GCA_000961495.1
GCA_000961515.1
GCA_000962775.1
GCA_000963495.1
GCA_000963535.2
GCA_000963555.1
GCA_000963575.1
GCA_000963635.1
GCA_000963645.1
GCA_000963675.1
GCA_000963815.1
GCA_000963835.1
GCA_000963865.1
GCA_000964565.1
GCA_000965765.1
GCA_000965785.1
GCA_000966445.2
GCA_000967115.1
GCA_000967135.1
GCA_000967155.1
GCA_000967285.1
GCA_000967305.2
GCA_000967325.1
GCA_000967345.1
GCA_000967365.1
GCA_000967385.1
GCA_000967405.1
GCA_000967425.1
GCA_000967445.1
GCA_000967895.1
GCA_000967915.1
GCA_000968055.1
GCA_000968115.1
GCA_000968135.1
GCA_000968155.1
GCA_000968175.1
GCA_000968195.1
GCA_000968335.1
GCA_000968375.1
GCA_000968415.2
GCA_000968515.1

GCA_000968535.1
GCA_000968715.2
GCA_000968945.1
GCA_000969225.1
GCA_000969235.1
GCA_000969265.1
GCA_000969315.2
GCA_000969645.2
GCA_000969685.1
GCA_000969765.1
GCA_000970665.2
GCA_000971565.1
GCA_000971615.1
GCA_000971665.1
GCA_000971705.1
GCA_000971725.1
GCA_000971765.1
GCA_000971925.1
GCA_000972245.3
GCA_000972685.1
GCA_000972725.1
GCA_000972745.1
GCA_000972765.1
GCA_000972785.3
GCA_000972805.1
GCA_000972865.1
GCA_000973085.1
GCA_000973105.1
GCA_000973125.1
GCA_000973485.1
GCA_000973505.1
GCA_000973545.1
GCA_000973565.1
GCA_000973585.1
GCA_000973605.1
GCA_000973625.1
GCA_000973645.1
GCA_000973665.2
GCA_000973685.2
GCA_000973705.1
GCA_000973725.1
GCA_000974405.1
GCA_000974425.1
GCA_000974465.1
GCA_000974505.1
GCA_000974535.1
GCA_000974575.1
GCA_000974685.2
GCA_000974825.1
GCA_000974835.2
GCA_000974865.1
GCA_000974885.1
GCA_000975175.1
GCA_000975245.1

GCA_000975265.2
GCA_000978375.1
GCA_000978495.1
GCA_000978535.1
GCA_000978555.1
GCA_000978575.1
GCA_000978785.2
GCA_000978815.2
GCA_000978845.2
GCA_000980815.1
GCA_000980835.1
GCA_000981485.1
GCA_000981505.1
GCA_000981525.1
GCA_000981545.1
GCA_000981565.1
GCA_000981585.1
GCA_000981765.1
GCA_000981785.2
GCA_000981805.1
GCA_000981825.1
GCA_000982695.1
GCA_000982715.1
GCA_000982825.1
GCA_000986765.1
GCA_000987825.1
GCA_000987835.1
GCA_000987865.1
GCA_000987875.1
GCA_000987925.1
GCA_000988065.1
GCA_000988345.1
GCA_000988355.1
GCA_000988385.1
GCA_000988395.1
GCA_000988425.1
GCA_000988445.1
GCA_000988465.1
GCA_000988485.1
GCA_000988525.3
GCA_000988605.1
GCA_000988615.1
GCA_000988655.1
GCA_000988685.1
GCA_000988705.1
GCA_000988725.2
GCA_000988745.3
GCA_000993725.1
GCA_000993745.1
GCA_000993765.1
GCA_000993785.3
GCA_000993825.1
GCA_001005265.1
GCA_001005905.1

GCA_001005925.2
GCA_001005985.1
GCA_001006005.1
GCA_001006025.1
GCA_001006525.1
GCA_001007005.1
GCA_001007025.1
GCA_001007045.1
GCA_001007065.1
GCA_001007085.1
GCA_001007105.1
GCA_001007125.1
GCA_001007145.1
GCA_001007875.1
GCA_001007915.1
GCA_001007935.1
GCA_001007975.1
GCA_001007995.1
GCA_001008015.1
GCA_001008165.2
GCA_001010285.1
GCA_001010485.1
GCA_001010505.1
GCA_001010765.1
GCA_001010785.2
GCA_001010805.1
GCA_001010825.1
GCA_001011035.1
GCA_001011055.2
GCA_001011095.1
GCA_001011135.1
GCA_001011155.1
GCA_001011675.1
GCA_001013565.1
GCA_001013905.1
GCA_001014285.1
GCA_001014305.1
GCA_001015095.1
GCA_001017435.1
GCA_001017575.1
GCA_001017595.1
GCA_001017615.1
GCA_001017635.1
GCA_001017655.1
GCA_001017775.3
GCA_001018645.2
GCA_001018655.2
GCA_001018685.2
GCA_001018725.2
GCA_001018735.2
GCA_001018775.2
GCA_001018805.2
GCA_001018835.2
GCA_001018845.2

GCA_001018915.2
GCA_001018975.2
GCA_001019125.2
GCA_001019205.2
GCA_001019255.2
GCA_001019275.2
GCA_001019305.2
GCA_001019395.2
GCA_001019415.2
GCA_001019435.2
GCA_001019485.2
GCA_001019495.2
GCA_001019535.2
GCA_001019575.2
GCA_001019635.1
GCA_001019645.1
GCA_001019675.1
GCA_001019695.1
GCA_001020185.2
GCA_001020205.1
GCA_001020725.2
GCA_001020945.2
GCA_001020955.1
GCA_001020985.1
GCA_001021005.2
GCA_001021025.1
GCA_001021045.1
GCA_001021065.1
GCA_001021085.1
GCA_001021385.1
GCA_001021595.1
GCA_001021615.1
GCA_001021635.2
GCA_001021855.1
GCA_001021875.1
GCA_001021895.1
GCA_001021915.1
GCA_001021935.1
GCA_001021955.1
GCA_001021975.1
GCA_001021995.1
GCA_001022015.1
GCA_001022035.1
GCA_001022055.1
GCA_001022075.1
GCA_001022095.1
GCA_001022115.1
GCA_001022155.1
GCA_001022175.1
GCA_001022215.1
GCA_001022235.1
GCA_001022255.1
GCA_001022275.1
GCA_001022295.1

GCA_001023495.1
GCA_001023535.1
GCA_001023575.1
GCA_001023595.1
GCA_001025135.1
GCA_001025155.1
GCA_001025175.1
GCA_001025195.1
GCA_001025215.1
GCA_001026925.1
GCA_001026945.2
GCA_001026965.1
GCA_001026985.1
GCA_001027025.1
GCA_001027045.1
GCA_001027065.1
GCA_001027085.1
GCA_001027105.1
GCA_001027125.1
GCA_001027165.1
GCA_001027205.1
GCA_001027225.1
GCA_001027245.1
GCA_001027265.1
GCA_001027285.1
GCA_001027545.1
GCA_001028285.3
GCA_001028625.1
GCA_001028645.1
GCA_001028665.1
GCA_001028705.1
GCA_001029105.3
GCA_001029125.1
GCA_001029145.1
GCA_001029245.1
GCA_001029265.1
GCA_001029635.1
GCA_001029645.1
GCA_001029675.1
GCA_001029695.1
GCA_001029715.1
GCA_001029735.1
GCA_001029755.1
GCA_001029775.1
GCA_001029795.1
GCA_001029815.1
GCA_001029835.1
GCA_001037985.1
GCA_001038625.1
GCA_001038645.1
GCA_001039275.2
GCA_001039415.1
GCA_001039495.1
GCA_001039695.2

GCA_001040945.1
GCA_001042405.1
GCA_001042525.2
GCA_001042545.2
GCA_001042565.2
GCA_001042595.1
GCA_001042615.1
GCA_001042635.1
GCA_001042655.1
GCA_001042675.1
GCA_001042695.1
GCA_001042715.1
GCA_001042735.1
GCA_001042745.1
GCA_001042775.1
GCA_001042795.1
GCA_001042815.1
GCA_001042835.1
GCA_001042855.1
GCA_001042875.1
GCA_001043135.1
GCA_001043175.1
GCA_001043215.1
GCA_001043255.1
GCA_001043295.1
GCA_001045415.1
GCA_001045685.1
GCA_001045795.2
GCA_001045995.2
GCA_001046095.2
GCA_001046835.1
GCA_001047215.2
GCA_001047225.1
GCA_001047255.1
GCA_001047275.1
GCA_001047635.1
GCA_001047655.1
GCA_001047675.1
GCA_001047695.1
GCA_001047715.2
GCA_001048035.2
GCA_001048375.2
GCA_001050115.1
GCA_001050135.1
GCA_001050395.1
GCA_001050435.1
GCA_001050455.1
GCA_001050475.1
GCA_001050915.2
GCA_001051015.2
GCA_001051055.1
GCA_001051075.1
GCA_001051095.1
GCA_001051115.1

GCA_001051135.1
GCA_001051995.2
GCA_001077475.1
GCA_001077535.2
GCA_001077555.2
GCA_001077655.1
GCA_001077675.1
GCA_001077795.1
GCA_001077815.2
GCA_001077965.2
GCA_001078055.1
GCA_001078275.1
GCA_001078295.1
GCA_001078495.2
GCA_001078615.1
GCA_001079465.2
GCA_001088345.2
GCA_001095585.2
GCA_001104165.2
GCA_001104885.3
GCA_001106745.2
GCA_001118185.2
GCA_001119245.2
GCA_001121865.2
GCA_001122625.2
GCA_001127485.2
GCA_001134605.2
GCA_001135805.3
GCA_001148125.2
GCA_001148305.2
GCA_001157245.2
GCA_001163025.2
GCA_001165785.2
GCA_001173525.2
GCA_001182745.2
GCA_001182785.1
GCA_001183645.1
GCA_001183665.1
GCA_001183685.1
GCA_001183705.3
GCA_001183725.2
GCA_001183745.1
GCA_001183765.1
GCA_001183785.1
GCA_001183805.1
GCA_001183825.1
GCA_001183845.1
GCA_001183865.1
GCA_001185215.1
GCA_001185245.1
GCA_001186155.3
GCA_001186215.2
GCA_001186335.1
GCA_001186405.1

GCA_001186415.1
GCA_001186445.1
GCA_001186465.1
GCA_001187595.1
GCA_001187615.2
GCA_001187785.1
GCA_001187845.1
GCA_001187975.3
GCA_001188025.2
GCA_001188045.2
GCA_001188125.2
GCA_001188185.2
GCA_001188655.1
GCA_001188695.1
GCA_001188715.1
GCA_001188755.1
GCA_001188775.1
GCA_001188795.1
GCA_001188815.1
GCA_001188835.1
GCA_001188935.1
GCA_001189295.1
GCA_001189495.1
GCA_001189515.2
GCA_001189535.1
GCA_001189555.1
GCA_001190745.1
GCA_001190755.1
GCA_001190805.1
GCA_001190865.1
GCA_001190885.1
GCA_001190905.2
GCA_001190925.1
GCA_001190945.1
GCA_001191005.1
GCA_001191565.1
GCA_001191605.1
GCA_001240865.2
GCA_001242845.1
GCA_001244315.1
GCA_001244395.1
GCA_001250235.2
GCA_001255215.1
GCA_001261895.2
GCA_001262015.1
GCA_001262035.1
GCA_001262055.1
GCA_001262075.1
GCA_001262115.1
GCA_001262715.1
GCA_001263175.1
GCA_001263205.1
GCA_001263395.1
GCA_001263815.1

GCA_001264245.1
GCA_001266635.1
GCA_001266755.1
GCA_001266795.1
GCA_001267155.1
GCA_001267175.1
GCA_001267195.1
GCA_001267215.1
GCA_001267235.1
GCA_001267255.1
GCA_001267275.1
GCA_001267295.1
GCA_001267395.1
GCA_001267405.1
GCA_001267435.1
GCA_001267475.1
GCA_001267805.1
GCA_001267815.1
GCA_001267845.1
GCA_001267865.1
GCA_001267885.1
GCA_001267925.1
GCA_001269425.1
GCA_001271345.1
GCA_001272315.2
GCA_001272615.1
GCA_001272635.1
GCA_001272655.2
GCA_001272715.1
GCA_001272735.1
GCA_001272755.1
GCA_001272775.1
GCA_001272795.1
GCA_001272815.1
GCA_001272835.1
GCA_001272855.1
GCA_001272875.1
GCA_001272895.1
GCA_001272915.1
GCA_001273775.1
GCA_001273795.1
GCA_001274515.1
GCA_001274535.1
GCA_001274555.1
GCA_001274575.1
GCA_001274595.1
GCA_001274615.1
GCA_001274655.1
GCA_001274675.1
GCA_001274835.1
GCA_001274875.1
GCA_001274895.1
GCA_001275345.1
GCA_001275365.1

GCA_001275545.2
GCA_001275565.2
GCA_001276585.2
GCA_001276975.2
GCA_001277175.1
GCA_001277195.1
GCA_001277215.2
GCA_001277235.1
GCA_001277255.1
GCA_001277275.1
GCA_001277295.1
GCA_001277875.1
GCA_001277895.1
GCA_001277915.1
GCA_001277975.1
GCA_001277995.1
GCA_001278015.1
GCA_001278035.1
GCA_001278055.1
GCA_001278075.1
GCA_001278095.1
GCA_001278275.1
GCA_001278295.1
GCA_001278715.1
GCA_001278745.1
GCA_001278785.1
GCA_001278825.1
GCA_001278845.1
GCA_001278905.1
GCA_001280205.1
GCA_001280225.1
GCA_001280285.1
GCA_001280305.1
GCA_001280325.1
GCA_001280345.1
GCA_001280385.1
GCA_001280405.1
GCA_001280925.1
GCA_001281005.1
GCA_001281025.1
GCA_001281045.1
GCA_001281065.1
GCA_001281085.1
GCA_001281105.1
GCA_001281145.1
GCA_001281305.1
GCA_001281315.1
GCA_001281345.1
GCA_001281365.1
GCA_001281385.1
GCA_001281405.1
GCA_001281425.1
GCA_001281445.1
GCA_001281465.1

GCA_001281485.1
GCA_001281505.1
GCA_001281545.1
GCA_001281565.1
GCA_001286525.1
GCA_001291465.1
GCA_001293065.1
GCA_001293085.1
GCA_001293125.1
GCA_001293145.1
GCA_001293165.1
GCA_001293415.1
GCA_001293505.1
GCA_001294425.1
GCA_001294565.1
GCA_001294575.1
GCA_001294605.1
GCA_001294625.1
GCA_001294645.1
GCA_001295365.1
GCA_001296085.1
GCA_001296095.1
GCA_001296125.1
GCA_001296145.1
GCA_001296165.1
GCA_001296185.1
GCA_001296205.1
GCA_001296965.1
GCA_001296985.1
GCA_001298325.2
GCA_001298465.1
GCA_001298485.1
GCA_001298505.1
GCA_001298525.1
GCA_001299555.1
GCA_001299565.1
GCA_001299595.1
GCA_001302565.1
GCA_001302585.1
GCA_001302605.1
GCA_001302625.1
GCA_001302645.1
GCA_001304715.1
GCA_001304735.1
GCA_001304755.1
GCA_001304775.1
GCA_001304795.1
GCA_001305235.1
GCA_001305575.3
GCA_001305595.1
GCA_001305615.1
GCA_001305635.1
GCA_001305675.1
GCA_001305715.1

GCA_001305815.1
GCA_001305835.1
GCA_001307155.1
GCA_001307175.1
GCA_001307195.1
GCA_001307215.1
GCA_001307235.1
GCA_001307275.1
GCA_001307295.1
GCA_001307475.2
GCA_001307505.2
GCA_001307525.1
GCA_001307545.1
GCA_001307565.1
GCA_001307585.1
GCA_001307605.1
GCA_001307625.1
GCA_001307645.1
GCA_001307665.1
GCA_001307685.1
GCA_001307705.1
GCA_001307725.1
GCA_001307745.1
GCA_001307805.2
GCA_001308065.1
GCA_001308105.1
GCA_001308125.1
GCA_001308145.2
GCA_001308165.1
GCA_001308265.1
GCA_001308285.1
GCA_001310085.1
GCA_001310225.1
GCA_001310255.1
GCA_001310575.2
GCA_001314225.1
GCA_001314265.1
GCA_001314285.1
GCA_001314305.1
GCA_001314325.1
GCA_001314945.1
GCA_001314975.1
GCA_001314995.1
GCA_001315015.1
GCA_001316565.3
GCA_001317685.2
GCA_001318185.1
GCA_001318245.1
GCA_001318295.1
GCA_001318345.1
GCA_001318385.1
GCA_001331925.2
GCA_001356455.2
GCA_001357935.2

GCA_001359015.2
GCA_001360555.2
GCA_001361075.2
GCA_001362095.2
GCA_001362135.2
GCA_001362175.2
GCA_001362195.2
GCA_001362335.2
GCA_001364695.2
GCA_001367555.2
GCA_001399515.1
GCA_001399655.1
GCA_001399775.1
GCA_001401705.2
GCA_001402875.1
GCA_001402915.1
GCA_001406795.1
GCA_001409135.1
GCA_001409155.1
GCA_001409175.1
GCA_001409195.1
GCA_001411495.1
GCA_001411765.2
GCA_001411805.1
GCA_001412295.1
GCA_001412535.1
GCA_001412575.1
GCA_001412595.2
GCA_001412635.1
GCA_001412655.1
GCA_001412695.1
GCA_001414055.1
GCA_001417635.1
GCA_001417865.2
GCA_001420855.1
GCA_001420915.1
GCA_001420935.1
GCA_001420955.1
GCA_001420975.1
GCA_001420995.1
GCA_001421015.2
GCA_001430755.1
GCA_001430775.1
GCA_001430805.1
GCA_001430825.1
GCA_001430845.1
GCA_001430865.1
GCA_001430885.1
GCA_001430905.1
GCA_001431145.1
GCA_001431725.1
GCA_001431745.1
GCA_001431765.1
GCA_001431785.1

GCA_001431805.1
GCA_001432245.1
GCA_001433415.1
GCA_001433435.1
GCA_001433475.1
GCA_001433495.1
GCA_001433515.1
GCA_001433715.1
GCA_001433955.1
GCA_001439585.2
GCA_001441165.1
GCA_001441205.1
GCA_001441225.1
GCA_001441245.1
GCA_001442475.1
GCA_001442495.1
GCA_001442535.1
GCA_001442745.1
GCA_001442755.1
GCA_001442785.1
GCA_001442805.1
GCA_001442815.3
GCA_001443605.1
GCA_001443645.1
GCA_001444325.1
GCA_001444345.1
GCA_001444365.1
GCA_001444405.1
GCA_001444425.1
GCA_001444445.1
GCA_001444465.1
GCA_001445575.1
GCA_001446255.1
GCA_001446275.1
GCA_001447075.1
GCA_001447095.1
GCA_001447115.1
GCA_001447155.2
GCA_001447175.1
GCA_001447295.1
GCA_001447315.1
GCA_001447335.1
GCA_001447805.1
GCA_001447845.1
GCA_001447865.2
GCA_001447885.1
GCA_001447915.1
GCA_001448485.2
GCA_001448665.2
GCA_001448705.2
GCA_001448785.2
GCA_001448985.1
GCA_001449005.1
GCA_001449085.1

GCA_001454845.1
GCA_001454865.1
GCA_001454885.1
GCA_001454905.1
GCA_001454925.1
GCA_001454945.1
GCA_001454965.1
GCA_001454985.1
GCA_001455085.1
GCA_001455105.1
GCA_001455145.1
GCA_001455205.1
GCA_001455225.1
GCA_001455325.1
GCA_001455345.1
GCA_001455365.1
GCA_001455385.1
GCA_001455995.1
GCA_001456055.3
GCA_001456065.2
GCA_001456095.3
GCA_001456115.1
GCA_001456135.1
GCA_001456155.1
GCA_001456175.1
GCA_001456195.2
GCA_001456215.1
GCA_001456235.1
GCA_001456255.1
GCA_001456275.1
GCA_001456295.2
GCA_001456315.1
GCA_001456335.3
GCA_001456355.1
GCA_001457455.1
GCA_001457475.1
GCA_001457495.1
GCA_001457535.1
GCA_001457555.1
GCA_001457595.1
GCA_001457615.1
GCA_001457635.1
GCA_001457655.1
GCA_001457675.1
GCA_001457695.1
GCA_001458475.1
GCA_001458695.1
GCA_001459775.1
GCA_001460635.1
GCA_001460855.1
GCA_001461805.1
GCA_001465115.1
GCA_001465155.1
GCA_001465175.1

GCA_001465255.1
GCA_001465275.1
GCA_001465295.1
GCA_001465545.3
GCA_001465595.2
GCA_001465635.1
GCA_001465675.1
GCA_001465755.1
GCA_001465795.2
GCA_001465815.1
GCA_001465835.2
GCA_001465855.1
GCA_001466415.2
GCA_001466505.1
GCA_001466725.1
GCA_001468185.2
GCA_001468205.2
GCA_001469565.1
GCA_001469595.1
GCA_001469615.1
GCA_001469655.1
GCA_001469735.1
GCA_001469775.1
GCA_001469815.1
GCA_001477575.1
GCA_001477625.1
GCA_001479685.2
GCA_001481655.1
GCA_001481675.1
GCA_001481715.1
GCA_001481725.1
GCA_001481755.1
GCA_001482325.1
GCA_001482345.1
GCA_001482365.1
GCA_001482385.1
GCA_001482725.1
GCA_001483405.1
GCA_001483425.1
GCA_001483445.1
GCA_001483845.1
GCA_001483865.1
GCA_001483885.1
GCA_001483905.1
GCA_001483945.1
GCA_001483965.1
GCA_001483985.1
GCA_001484005.1
GCA_001484025.1
GCA_001484045.1
GCA_001484065.1
GCA_001484565.1
GCA_001484605.1
GCA_001484645.1

GCA_001484665.1
GCA_001484725.1
GCA_001484935.1
GCA_001485455.1
GCA_001499615.1
GCA_001499655.1
GCA_001506165.1
GCA_001506185.1
GCA_001506205.1
GCA_001506225.1
GCA_001506245.1
GCA_001506265.1
GCA_001506285.1
GCA_001506305.1
GCA_001506325.1
GCA_001506345.1
GCA_001506365.1
GCA_001506385.1
GCA_001506405.1
GCA_001506425.1
GCA_001506445.1
GCA_001506465.1
GCA_001506485.1
GCA_001506505.1
GCA_001506525.1
GCA_001506545.1
GCA_001506565.1
GCA_001506585.1
GCA_001506605.1
GCA_001506625.1
GCA_001506645.1
GCA_001506665.1
GCA_001506685.1
GCA_001506705.1
GCA_001506725.1
GCA_001506745.1
GCA_001506765.1
GCA_001506785.1
GCA_001506805.1
GCA_001506825.1
GCA_001506845.1
GCA_001506865.1
GCA_001506885.1
GCA_001506905.1
GCA_001506925.1
GCA_001506945.1
GCA_001506965.1
GCA_001506985.1
GCA_001507005.1
GCA_001507025.1
GCA_001507045.1
GCA_001507065.1
GCA_001507085.1
GCA_001507105.1

GCA_001507125.1
GCA_001507145.1
GCA_001507165.1
GCA_001507185.1
GCA_001507205.1
GCA_001507225.1
GCA_001507245.1
GCA_001507265.1
GCA_001507645.1
GCA_001507665.1
GCA_001509195.1
GCA_001509405.1
GCA_001509895.1
GCA_001509915.1
GCA_001511755.1
GCA_001511775.1
GCA_001511835.1
GCA_001513615.1
GCA_001513635.1
GCA_001513655.1
GCA_001513675.1
GCA_001513695.1
GCA_001513715.1
GCA_001513745.1
GCA_001514355.1
GCA_001514375.1
GCA_001514395.1
GCA_001514415.1
GCA_001514435.1
GCA_001514455.1
GCA_001515585.2
GCA_001515665.1
GCA_001515685.1
GCA_001515705.1
GCA_001515725.1
GCA_001515745.1
GCA_001515765.1
GCA_001515845.2
GCA_001515915.2
GCA_001516005.1
GCA_001516105.1
GCA_001516165.2
GCA_001516185.2
GCA_001516205.2
GCA_001516225.2
GCA_001516245.2
GCA_001516265.1
GCA_001516305.2
GCA_001516325.2
GCA_001516345.1
GCA_001516365.2
GCA_001516385.1
GCA_001517405.1
GCA_001517645.1

GCA_001518735.1
GCA_001518775.1
GCA_001518795.1
GCA_001518815.1
GCA_001518835.1
GCA_001518855.1
GCA_001518875.1
GCA_001518895.1
GCA_001518935.2
GCA_001518975.1
GCA_001518995.2
GCA_001521715.1
GCA_001521855.1
GCA_001521895.1
GCA_001522105.2
GCA_001522135.2
GCA_001522585.2
GCA_001522635.2
GCA_001523745.2
GCA_001523915.2
GCA_001524445.2
GCA_001524625.2
GCA_001524645.2
GCA_001529935.1
GCA_001530015.1
GCA_001532025.2
GCA_001534645.1
GCA_001534665.1
GCA_001534725.1
GCA_001534745.1
GCA_001534765.1
GCA_001534785.1
GCA_001535315.2
GCA_001535505.1
GCA_001535545.1
GCA_001535565.1
GCA_001536305.1
GCA_001536925.1
GCA_001540845.1
GCA_001541905.1
GCA_001542625.2
GCA_001542675.2
GCA_001542775.1
GCA_001542795.2
GCA_001542815.1
GCA_001542835.1
GCA_001543055.1
GCA_001543085.1
GCA_001543105.1
GCA_001543145.1
GCA_001543175.1
GCA_001543205.1
GCA_001543225.1
GCA_001543245.1

GCA_001543265.1
GCA_001543285.1
GCA_001543305.1
GCA_001543325.1
GCA_001543345.1
GCA_001543995.1
GCA_001544015.1
GCA_001544635.1
GCA_001544675.1
GCA_001544705.1
GCA_001544735.1
GCA_001544775.1
GCA_001544815.1
GCA_001544855.1
GCA_001544895.1
GCA_001544935.1
GCA_001544955.1
GCA_001544985.1
GCA_001545015.1
GCA_001545055.1
GCA_001545095.1
GCA_001545155.1
GCA_001547715.1
GCA_001547735.1
GCA_001547755.1
GCA_001547775.1
GCA_001547795.1
GCA_001547815.1
GCA_001547835.1
GCA_001547855.1
GCA_001547875.1
GCA_001547895.1
GCA_001547915.1
GCA_001547935.1
GCA_001547955.1
GCA_001547975.1
GCA_001547995.1
GCA_001548015.1
GCA_001548035.1
GCA_001548055.1
GCA_001548075.1
GCA_001548095.1
GCA_001548115.1
GCA_001548135.1
GCA_001548155.2
GCA_001548175.1
GCA_001548195.1
GCA_001548215.1
GCA_001548235.1
GCA_001548275.1
GCA_001548295.1
GCA_001548335.1
GCA_001548375.1
GCA_001548395.1

GCA_001548415.1
GCA_001548435.1
GCA_001548455.1
GCA_001548585.2
GCA_001548635.2
GCA_001549655.1
GCA_001549675.1
GCA_001549695.1
GCA_001549715.1
GCA_001549855.1
GCA_001549875.1
GCA_001549895.1
GCA_001549955.1
GCA_001551855.1
GCA_001552035.1
GCA_001553195.1
GCA_001553545.1
GCA_001553565.1
GCA_001553605.1
GCA_001553625.1
GCA_001553645.1
GCA_001553685.1
GCA_001553895.1
GCA_001553915.1
GCA_001553935.1
GCA_001553955.1
GCA_001554015.1
GCA_001554055.1
GCA_001554075.1
GCA_001554095.1
GCA_001554115.1
GCA_001554155.1
GCA_001557535.2
GCA_001558175.1
GCA_001558215.1
GCA_001558255.2
GCA_001558275.2
GCA_001558295.2
GCA_001558355.2
GCA_001558375.2
GCA_001558395.2
GCA_001558415.2
GCA_001558435.2
GCA_001558475.2
GCA_001558495.2
GCA_001558515.2
GCA_001558735.2
GCA_001558755.2
GCA_001558775.1
GCA_001558795.2
GCA_001558815.2
GCA_001558855.2
GCA_001558875.2
GCA_001558895.2

GCA_001558915.2
GCA_001558935.2
GCA_001558975.1
GCA_001558995.2
GCA_001559035.2
GCA_001559055.2
GCA_001559075.2
GCA_001559115.2
GCA_001559135.2
GCA_001559175.2
GCA_001559195.2
GCA_001559215.2
GCA_001559235.2
GCA_001559255.2
GCA_001559615.2
GCA_001559635.1
GCA_001559655.1
GCA_001559675.1
GCA_001560895.1
GCA_001561955.1
GCA_001561975.1
GCA_001562125.1
GCA_001562155.1
GCA_001562195.1
GCA_001562215.1
GCA_001562235.1
GCA_001562255.1
GCA_001562275.1
GCA_001562295.1
GCA_001562315.1
GCA_001563225.1
GCA_001563265.1
GCA_001563285.1
GCA_001563565.1
GCA_001564435.1
GCA_001564455.1
GCA_001565875.1
GCA_001566335.1
GCA_001566615.1
GCA_001566635.1
GCA_001566655.1
GCA_001566675.1
GCA_001571545.2
GCA_001572685.1
GCA_001572725.1
GCA_001572745.1
GCA_001573065.1
GCA_001573085.1
GCA_001573105.1
GCA_001573125.1
GCA_001576255.1
GCA_001576275.1
GCA_001576595.1
GCA_001577265.1

GCA_001577285.1
GCA_001577305.1
GCA_001577325.1
GCA_001577365.1
GCA_001577385.1
GCA_001577465.1
GCA_001577505.1
GCA_001577755.1
GCA_001577795.1
GCA_001578105.1
GCA_001578125.1
GCA_001578145.1
GCA_001578165.1
GCA_001578185.1
GCA_001578205.1
GCA_001578435.2
GCA_001578515.1
GCA_001579765.1
GCA_001579785.1
GCA_001579805.1
GCA_001579825.2
GCA_001579845.1
GCA_001579865.1
GCA_001579885.2
GCA_001579905.1
GCA_001579925.1
GCA_001579945.1
GCA_001579965.1
GCA_001580175.1
GCA_001580385.1
GCA_001580455.1
GCA_001580475.1
GCA_001580495.1
GCA_001580515.1
GCA_001581875.1
GCA_001581895.1
GCA_001581975.1
GCA_001583415.1
GCA_001583825.1
GCA_001584145.1
GCA_001584165.1
GCA_001584185.1
GCA_001584205.1
GCA_001584225.1
GCA_001584725.1
GCA_001586105.1
GCA_001586135.1
GCA_001586155.1
GCA_001586165.1
GCA_001586215.1
GCA_001586235.1
GCA_001586255.1
GCA_001586275.1
GCA_001587015.1

GCA_001587035.1
GCA_001587115.1
GCA_001587135.1
GCA_001587155.1
GCA_001587175.1
GCA_001589345.2
GCA_001590605.1
GCA_001590615.2
GCA_001590645.1
GCA_001590685.1
GCA_001590695.1
GCA_001592385.1
GCA_001592395.1
GCA_001592425.1
GCA_001592705.1
GCA_001593245.1
GCA_001593285.1
GCA_001593305.1
GCA_001593395.2
GCA_001593425.2
GCA_001593565.1
GCA_001593585.2
GCA_001593605.1
GCA_001593665.2
GCA_001593765.1
GCA_001593785.1
GCA_001594205.1
GCA_001594225.2
GCA_001594245.1
GCA_001594265.1
GCA_001594325.2
GCA_001594345.1
GCA_001595725.1
GCA_001596055.1
GCA_001596075.2
GCA_001596095.1
GCA_001596155.1
GCA_001596175.2
GCA_001596535.1
GCA_001596755.1
GCA_001596775.2
GCA_001597265.1
GCA_001597285.1
GCA_001597645.2
GCA_001597795.2
GCA_001598035.1
GCA_001598075.1
GCA_001598095.1
GCA_001598115.2
GCA_001598635.1
GCA_001602095.1
GCA_001602115.1
GCA_001602135.1
GCA_001602155.1

GCA_001602215.1
GCA_001602415.1
GCA_001602435.1
GCA_001602455.1
GCA_001604995.1
GCA_001605015.1
GCA_001605035.1
GCA_001605055.1
GCA_001605075.1
GCA_001605095.1
GCA_001605115.1
GCA_001605135.1
GCA_001605155.1
GCA_001605175.1
GCA_001605195.1
GCA_001605215.1
GCA_001605235.1
GCA_001605255.1
GCA_001605275.1
GCA_001605295.1
GCA_001605345.1
GCA_001605365.1
GCA_001605385.1
GCA_001605405.1
GCA_001605425.1
GCA_001605445.1
GCA_001605465.1
GCA_001605485.1
GCA_001605505.1
GCA_001605525.1
GCA_001605545.1
GCA_001605565.1
GCA_001605585.1
GCA_001605605.1
GCA_001605625.1
GCA_001605645.1
GCA_001605665.1
GCA_001605685.1
GCA_001605725.1
GCA_001605965.1
GCA_001606005.1
GCA_001606025.1
GCA_001606045.1
GCA_001606115.1
GCA_001606135.1
GCA_001606195.1
GCA_001606215.1
GCA_001606235.1
GCA_001606255.1
GCA_001606275.1
GCA_001606295.1
GCA_001606315.1
GCA_001606335.1
GCA_001610615.1

GCA_001610635.1
GCA_001610655.1
GCA_001610675.1
GCA_001610735.1
GCA_001610755.1
GCA_001610775.1
GCA_001610795.1
GCA_001610815.1
GCA_001610835.1
GCA_001610855.1
GCA_001610875.1
GCA_001610895.1
GCA_001610915.1
GCA_001610935.1
GCA_001610975.1
GCA_001610995.1
GCA_001611015.1
GCA_001611035.1
GCA_001611055.1
GCA_001611075.1
GCA_001611095.1
GCA_001611115.1
GCA_001611135.1
GCA_001611155.1
GCA_001611275.1
GCA_001611325.1
GCA_001611345.1
GCA_001611365.1
GCA_001611385.1
GCA_001611405.1
GCA_001611425.1
GCA_001611675.1
GCA_001611775.1
GCA_001611795.1
GCA_001611815.4
GCA_001611865.1
GCA_001611895.1
GCA_001611935.1
GCA_001611955.1
GCA_001611975.1
GCA_001612475.1
GCA_001612495.1
GCA_001612515.1
GCA_001612555.1
GCA_001612705.2
GCA_001617525.2
GCA_001617545.1
GCA_001617565.1
GCA_001617605.1
GCA_001617625.1
GCA_001617645.1
GCA_001618305.1
GCA_001618325.1
GCA_001618345.2

GCA_001618365.1
GCA_001618465.2
GCA_001618505.2
GCA_001618545.2
GCA_001618665.1
GCA_001618685.1
GCA_001618825.1
GCA_001618845.1
GCA_001618865.1
GCA_001618885.1
GCA_001618905.1
GCA_001618925.1
GCA_001620005.1
GCA_001620025.1
GCA_001620115.1
GCA_001620135.2
GCA_001620155.1
GCA_001620205.2
GCA_001620225.1
GCA_001620265.1
GCA_001620285.1
GCA_001620305.1
GCA_001620335.2
GCA_001623545.2
GCA_001623565.1
GCA_001623585.1
GCA_001623605.1
GCA_001623625.1
GCA_001623645.2
GCA_001623665.2
GCA_001623685.1
GCA_001623705.1
GCA_001623725.1
GCA_001623745.2
GCA_001623765.2
GCA_001623775.1
GCA_001623805.1
GCA_001623825.2
GCA_001623845.1
GCA_001628715.1
GCA_001628735.1
GCA_001628755.1
GCA_001628775.1
GCA_001629705.2
GCA_001629735.1
GCA_001629755.1
GCA_001629775.1
GCA_001630725.2
GCA_001632245.1
GCA_001632765.1
GCA_001632775.1
GCA_001632805.1
GCA_001632825.1
GCA_001632845.1

GCA_001632865.1
GCA_001633145.1
GCA_001633165.1
GCA_001634265.1
GCA_001634285.1
GCA_001634305.1
GCA_001634325.1
GCA_001634345.1
GCA_001634365.1
GCA_001634675.1
GCA_001635875.1
GCA_001635895.1
GCA_001635915.1
GCA_001635935.1
GCA_001635955.1
GCA_001635975.1
GCA_001635995.1
GCA_001636015.1
GCA_001636035.1
GCA_001636055.1
GCA_001636235.1
GCA_001636255.1
GCA_001636275.1
GCA_001636295.1
GCA_001636925.1
GCA_001636945.2
GCA_001638825.1
GCA_001638845.2
GCA_001638925.1
GCA_001639125.1
GCA_001640765.1
GCA_001640865.1
GCA_001640885.1
GCA_001640905.1
GCA_001640925.1
GCA_001640965.1
GCA_001640985.1
GCA_001641005.1
GCA_001641025.1
GCA_001641045.1
GCA_001641185.1
GCA_001641285.1
GCA_001641305.1
GCA_001641975.2
GCA_001642085.1
GCA_001642655.1
GCA_001642675.1
GCA_001642805.2
GCA_001642935.2
GCA_001643015.1
GCA_001643035.1
GCA_001643275.2
GCA_001643355.2
GCA_001643775.1

GCA_001643955.1
GCA_001643975.1
GCA_001643995.1
GCA_001644565.1
GCA_001644575.1
GCA_001644605.1
GCA_001644625.1
GCA_001644645.1
GCA_001644665.1
GCA_001644685.1
GCA_001644705.1
GCA_001644725.1
GCA_001644745.1
GCA_001644765.1
GCA_001645235.2
GCA_001645685.2
GCA_001646605.1
GCA_001646625.1
GCA_001647045.1
GCA_001647635.1
GCA_001647655.1
GCA_001647675.1
GCA_001647695.1
GCA_001647715.1
GCA_001647735.1
GCA_001647755.1
GCA_001647765.1
GCA_001647795.1
GCA_001647815.1
GCA_001647835.1
GCA_001647855.1
GCA_001647875.1
GCA_001647895.1
GCA_001647915.1
GCA_001648115.1
GCA_001648155.1
GCA_001648175.1
GCA_001648195.1
GCA_001648215.1
GCA_001650215.1
GCA_001650235.3
GCA_001650275.1
GCA_001650295.1
GCA_001651805.1
GCA_001651925.2
GCA_001651945.2
GCA_001651965.2
GCA_001652385.2
GCA_001652465.1
GCA_001652485.1
GCA_001652505.2
GCA_001652565.1
GCA_001652645.1
GCA_001652665.1

GCA_001652685.1
GCA_001653295.1
GCA_001653315.1
GCA_001653335.1
GCA_001653355.1
GCA_001653375.1
GCA_001653395.1
GCA_001653415.1
GCA_001653435.1
GCA_001653455.1
GCA_001653475.1
GCA_001653755.1
GCA_001653775.1
GCA_001653795.1
GCA_001653935.1
GCA_001653955.1
GCA_001654435.1
GCA_001654455.1
GCA_001654475.1
GCA_001654495.1
GCA_001654515.1
GCA_001655175.1
GCA_001655245.1
GCA_001655295.1
GCA_001655335.1
GCA_001655375.1
GCA_001655415.1
GCA_001655455.1
GCA_001655495.1
GCA_001655535.1
GCA_001655575.1
GCA_001655595.1
GCA_001655615.1
GCA_001656045.1
GCA_001656075.1
GCA_001658005.1
GCA_001658025.2
GCA_001659705.1
GCA_001659745.1
GCA_001659785.1
GCA_001660005.1
GCA_001660025.1
GCA_001660045.1
GCA_001660485.1
GCA_001660525.1
GCA_001660565.1
GCA_001660585.1
GCA_001661075.1
GCA_001661085.1
GCA_001661115.1
GCA_001661135.1
GCA_001661675.2
GCA_001661985.2
GCA_001662025.2

GCA_001663075.1
GCA_001663095.1
GCA_001663115.1
GCA_001663135.1
GCA_001663155.1
GCA_001663175.1
GCA_001663195.1
GCA_001663295.1
GCA_001663395.1
GCA_001663415.1
GCA_001663435.1
GCA_001663455.1
GCA_001663475.1
GCA_001663495.1
GCA_001663655.1
GCA_001663675.1
GCA_001663715.1
GCA_001663735.1
GCA_001663755.1
GCA_001663795.1
GCA_001663815.1
GCA_001663835.1
GCA_001663855.1
GCA_001664085.1
GCA_001664105.1
GCA_001664125.1
GCA_001664145.1
GCA_001664165.1
GCA_001664185.1
GCA_001664205.1
GCA_001664225.1
GCA_001664245.1
GCA_001664265.1
GCA_001664285.1
GCA_001664305.1
GCA_001664325.1
GCA_001664345.1
GCA_001664365.1
GCA_001664385.1
GCA_001664405.1
GCA_001664425.1
GCA_001664445.1
GCA_001664465.1
GCA_001664485.1
GCA_001664525.1
GCA_001664545.1
GCA_001668465.2
GCA_001669145.2
GCA_001670425.2
GCA_001670625.2
GCA_001670765.2
GCA_001672035.1
GCA_001672055.1
GCA_001672375.2

GCA_001672455.2
GCA_001673705.2
GCA_001674475.2
GCA_001674505.2
GCA_001675125.1
GCA_001675145.1
GCA_001675245.1
GCA_001675515.1
GCA_001676705.1
GCA_001676725.1
GCA_001676745.1
GCA_001676765.1
GCA_001676785.2
GCA_001676805.1
GCA_001676825.1
GCA_001677075.2
GCA_001677095.1
GCA_001677115.2
GCA_001677135.1
GCA_001677155.1
GCA_001677175.1
GCA_001677195.1
GCA_001677215.1
GCA_001677255.1
GCA_001677275.1
GCA_001677395.1
GCA_001677435.1
GCA_001677475.2
GCA_001677495.1
GCA_001677515.1
GCA_001678885.1
GCA_001678905.1
GCA_001678925.1
GCA_001678945.1
GCA_001678965.1
GCA_001679665.1
GCA_001679685.1
GCA_001679705.1
GCA_001679725.1
GCA_001679745.1
GCA_001679785.1
GCA_001679985.1
GCA_001680025.1
GCA_001680045.1
GCA_001680065.1
GCA_001680085.1
GCA_001680825.1
GCA_001682135.1
GCA_001682175.1
GCA_001682195.2
GCA_001682215.1
GCA_001682235.1
GCA_001682255.2
GCA_001682275.1

GCA_001682295.1
GCA_001682305.2
GCA_001682335.1
GCA_001682385.1
GCA_001682435.2
GCA_001683055.1
GCA_001683065.1
GCA_001683095.1
GCA_001683115.1
GCA_001683135.1
GCA_001683155.1
GCA_001683175.1
GCA_001683195.1
GCA_001683215.1
GCA_001683235.1
GCA_001683255.1
GCA_001683275.1
GCA_001683295.1
GCA_001683315.1
GCA_001683395.1
GCA_001683415.1
GCA_001683435.1
GCA_001683455.1
GCA_001683515.1
GCA_001683535.1
GCA_001685355.1
GCA_001685375.1
GCA_001685395.1
GCA_001685415.1
GCA_001685435.3
GCA_001685465.1
GCA_001685565.1
GCA_001685605.1
GCA_001685625.1
GCA_001685645.1
GCA_001685665.2
GCA_001686365.1
GCA_001686405.1
GCA_001686425.1
GCA_001686445.1
GCA_001686465.1
GCA_001686485.1
GCA_001686505.1
GCA_001686525.1
GCA_001686545.1
GCA_001686565.1
GCA_001686585.1
GCA_001686605.1
GCA_001686625.1
GCA_001686885.1
GCA_001686905.1
GCA_001686985.1
GCA_001687065.1
GCA_001687105.1

GCA_001687125.1
GCA_001687285.1
GCA_001687385.1
GCA_001687405.1
GCA_001687475.2
GCA_001687545.1
GCA_001687565.2
GCA_001687585.2
GCA_001687605.2
GCA_001687625.2
GCA_001687645.1
GCA_001687665.2
GCA_001687745.1
GCA_001687805.1
GCA_001688625.2
GCA_001688645.2
GCA_001688685.2
GCA_001688705.2
GCA_001688725.2
GCA_001688765.2
GCA_001688845.2
GCA_001688965.2
GCA_001689055.2
GCA_001689125.2
GCA_001689935.1
GCA_001690005.1
GCA_001690055.1
GCA_001690075.1
GCA_001690095.1
GCA_001690115.1
GCA_001690155.1
GCA_001691455.1
GCA_001692265.2
GCA_001692275.2
GCA_001692495.1
GCA_001692515.1
GCA_001692535.1
GCA_001692555.1
GCA_001692575.1
GCA_001692595.1
GCA_001692615.1
GCA_001692635.1
GCA_001692675.1
GCA_001692755.1
GCA_001693255.1
GCA_001693275.1
GCA_001693295.1
GCA_001693315.1
GCA_001693335.1
GCA_001693385.1
GCA_001693515.2
GCA_001693595.1
GCA_001693615.1
GCA_001693635.1

GCA_001693655.1
GCA_001693675.1
GCA_001695515.1
GCA_001695715.1
GCA_001695735.1
GCA_001696605.3
GCA_001696615.3
GCA_001697105.1
GCA_001697125.1
GCA_001697145.1
GCA_001697165.1
GCA_001697185.1
GCA_001697205.1
GCA_001697225.1
GCA_001697245.1
GCA_001697265.1
GCA_001697285.1
GCA_001697305.1
GCA_001697325.1
GCA_001697345.1
GCA_001697365.1
GCA_001697385.1
GCA_001697405.1
GCA_001697425.1
GCA_001697445.1
GCA_001697465.1
GCA_001697485.1
GCA_001697505.1
GCA_001697525.1
GCA_001697545.1
GCA_001697565.1
GCA_001697585.1
GCA_001697605.1
GCA_001697625.1
GCA_001697645.1
GCA_001697665.1
GCA_001697685.1
GCA_001697705.1
GCA_001697725.1
GCA_001697745.1
GCA_001697765.1
GCA_001697785.1
GCA_001697805.1
GCA_001697825.1
GCA_001697845.1
GCA_001697865.1
GCA_001697885.1
GCA_001697905.1
GCA_001697925.1
GCA_001697945.1
GCA_001697965.1
GCA_001697985.1
GCA_001698005.1
GCA_001698025.1

GCA_001698045.1
GCA_001698065.1
GCA_001698085.1
GCA_001698105.1
GCA_001698125.1
GCA_001698145.1
GCA_001698165.1
GCA_001698185.1
GCA_001698205.1
GCA_001698225.1
GCA_001699105.2
GCA_001700735.1
GCA_001700835.1
GCA_001700855.1
GCA_001700895.1
GCA_001700945.1
GCA_001700965.1
GCA_001700985.1
GCA_001701005.1
GCA_001701025.1
GCA_001701045.1
GCA_001701425.2
GCA_001701835.2
GCA_001701845.2
GCA_001702045.2
GCA_001702095.1
GCA_001702115.1
GCA_001702135.1
GCA_001702155.1
GCA_001702175.1
GCA_001702195.1
GCA_001702215.1
GCA_001702235.1
GCA_001702435.1
GCA_001703495.1
GCA_001703515.1
GCA_001703535.1
GCA_001703555.1
GCA_001703575.1
GCA_001703595.1
GCA_001704095.1
GCA_001704115.1
GCA_001704155.1
GCA_001704195.1
GCA_001704215.1
GCA_001704235.1
GCA_001704255.1
GCA_001704295.1
GCA_001704315.1
GCA_001704335.1
GCA_001704355.1
GCA_001704615.3
GCA_001704955.2
GCA_001704975.1

GCA_001705055.1
GCA_001705075.2
GCA_001705175.1
GCA_001705195.1
GCA_001705365.1
GCA_001705385.1
GCA_001705545.1
GCA_001705565.1
GCA_001705585.1
GCA_001705995.2
GCA_001708035.2
GCA_001708225.1
GCA_001708245.1
GCA_001708265.1
GCA_001708305.1
GCA_001708385.1
GCA_001708405.1
GCA_001708425.1
GCA_001708445.1
GCA_001708465.1
GCA_001708485.1
GCA_001708505.1
GCA_001708525.1
GCA_001708555.1
GCA_001708575.1
GCA_001708595.1
GCA_001708615.1
GCA_001708635.1
GCA_001708655.1
GCA_001709275.1
GCA_001709295.1
GCA_001709315.1
GCA_001712815.1
GCA_001712835.1
GCA_001712875.1
GCA_001713355.1
GCA_001713375.1
GCA_001713395.1
GCA_001713415.1
GCA_001713435.1
GCA_001713455.1
GCA_001713475.1
GCA_001713515.2
GCA_001714705.1
GCA_001714725.1
GCA_001714745.1
GCA_001715365.1
GCA_001715385.1
GCA_001715405.1
GCA_001715425.1
GCA_001715485.1
GCA_001715535.1
GCA_001715615.1
GCA_001717505.1

GCA_001717525.2
GCA_001717545.1
GCA_001717565.1
GCA_001717585.1
GCA_001717605.1
GCA_001717625.1
GCA_001717645.3
GCA_001717665.2
GCA_001717685.3
GCA_001717705.2
GCA_001717725.2
GCA_001717955.1
GCA_001717975.3
GCA_001718055.1
GCA_001718115.2
GCA_001718295.1
GCA_001718315.1
GCA_001718335.1
GCA_001718355.1
GCA_001718395.1
GCA_001718415.1
GCA_001718495.1
GCA_001718515.1
GCA_001718535.1
GCA_001718555.1
GCA_001718575.1
GCA_001718595.1
GCA_001718615.1
GCA_001718635.1
GCA_001718655.1
GCA_001718695.1
GCA_001718755.1
GCA_001718775.1
GCA_001718795.1
GCA_001718815.1
GCA_001718835.1
GCA_001718855.1
GCA_001718875.1
GCA_001718895.1
GCA_001718915.1
GCA_001718955.1
GCA_001718975.1
GCA_001718995.1
GCA_001719045.1
GCA_001719085.1
GCA_001719105.1
GCA_001719145.1
GCA_001719165.1
GCA_001719205.1
GCA_001719225.1
GCA_001720465.1
GCA_001720485.1
GCA_001720505.1
GCA_001720945.1

GCA_001721065.1
GCA_001721125.1
GCA_001721145.1
GCA_001721165.1
GCA_001721185.1
GCA_001721205.1
GCA_001721225.1
GCA_001721525.1
GCA_001721625.1
GCA_001721645.1
GCA_001721685.1
GCA_001721705.1
GCA_001721725.1
GCA_001721745.1
GCA_001721765.1
GCA_001721785.1
GCA_001721805.1
GCA_001721825.1
GCA_001721845.1
GCA_001721865.1
GCA_001721885.1
GCA_001721905.1
GCA_001721925.1
GCA_001721945.1
GCA_001721965.1
GCA_001721985.1
GCA_001722005.2
GCA_001722025.1
GCA_001722045.1
GCA_001723505.1
GCA_001723525.1
GCA_001723545.1
GCA_001723585.1
GCA_001723605.1
GCA_001723625.1
GCA_001725965.1
GCA_001725985.1
GCA_001726125.1
GCA_001729225.1
GCA_001729245.1
GCA_001729265.2
GCA_001729485.1
GCA_001729505.1
GCA_001729525.1
GCA_001729545.1
GCA_001729625.1
GCA_001729705.1
GCA_001729725.1
GCA_001729745.1
GCA_001729765.1
GCA_001729785.1
GCA_001729805.1
GCA_001729925.2
GCA_001729945.1

GCA_001735615.2
GCA_001735655.2
GCA_001735705.1
GCA_001735715.1
GCA_001735745.1
GCA_001735765.2
GCA_001735805.1
GCA_001741545.1
GCA_001741665.1
GCA_001741865.1
GCA_001742145.1
GCA_001742165.1
GCA_001742185.1
GCA_001742205.1
GCA_001742225.1
GCA_001746265.2
GCA_001746535.1
GCA_001746575.1
GCA_001746595.1
GCA_001746615.1
GCA_001746635.1
GCA_001746655.1
GCA_001746675.1
GCA_001746695.1
GCA_001746715.1
GCA_001746735.1
GCA_001746795.1
GCA_001746815.1
GCA_001746835.1
GCA_001747385.1
GCA_001747405.1
GCA_001747425.1
GCA_001747445.1
GCA_001750065.2
GCA_001750105.1
GCA_001750115.1
GCA_001750145.1
GCA_001750165.1
GCA_001750185.1
GCA_001750405.1
GCA_001750685.1
GCA_001750705.1
GCA_001750725.1
GCA_001750785.1
GCA_001750825.1
GCA_001750845.1
GCA_001750865.1
GCA_001750885.1
GCA_001752685.2
GCA_001752705.1
GCA_001752725.1
GCA_001752745.1
GCA_001752765.1
GCA_001752785.1

GCA_001752805.1
GCA_001752825.1
GCA_001752845.1
GCA_001752865.1
GCA_001752885.1
GCA_001752905.1
GCA_001752925.1
GCA_001752945.1
GCA_001752965.1
GCA_001753065.1
GCA_001753085.1
GCA_001753105.1
GCA_001753125.1
GCA_001753145.1
GCA_001753185.1
GCA_001753205.1
GCA_001753225.1
GCA_001753245.1
GCA_001753265.1
GCA_001753285.1
GCA_001753305.1
GCA_001753325.1
GCA_001753345.1
GCA_001753365.1
GCA_001753385.1
GCA_001753405.1
GCA_001753425.1
GCA_001753445.1
GCA_001753465.1
GCA_001753485.1
GCA_001753505.1
GCA_001753525.1
GCA_001753545.1
GCA_001753565.1
GCA_001756025.2
GCA_001758605.1
GCA_001758625.2
GCA_001761325.1
GCA_001761345.1
GCA_001761365.1
GCA_001761385.1
GCA_001761405.1
GCA_001761445.1
GCA_001761465.1
GCA_001761545.1
GCA_001765415.1
GCA_001766235.1
GCA_001766255.1
GCA_001766275.1
GCA_001766295.1
GCA_001766315.1
GCA_001766335.1
GCA_001766355.1
GCA_001766375.1

GCA_001767215.1
GCA_001767235.1
GCA_001767255.1
GCA_001767275.1
GCA_001767295.1
GCA_001767335.1
GCA_001767355.1
GCA_001767375.1
GCA_001767395.1
GCA_001787335.1
GCA_001787355.1
GCA_001792795.1
GCA_001792815.1
GCA_001792835.1
GCA_001792855.1
GCA_001792875.1
GCA_001806265.1
GCA_001806285.1
GCA_001806305.1
GCA_001806325.1
GCA_001806345.1
GCA_001806365.1
GCA_001806385.1
GCA_001806405.1
GCA_001806425.1
GCA_001806445.1
GCA_001806465.1
GCA_001806485.1
GCA_001808165.1
GCA_001808185.1
GCA_001808235.1
GCA_001816185.2
GCA_001831375.1
GCA_001831395.1
GCA_001831415.1
GCA_001831435.1
GCA_001831455.1
GCA_001831475.1
GCA_001831495.1
GCA_001831555.2
GCA_001831715.2
GCA_001831915.2
GCA_001831985.2
GCA_001832255.2
GCA_001833225.2
GCA_001833255.2
GCA_001833305.2
GCA_001834735.2
GCA_001834965.2
GCA_001839645.2
GCA_001839655.2
GCA_001854065.1
GCA_001854085.1
GCA_001854125.1

GCA_001854145.2
GCA_001854165.1
GCA_001854185.1
GCA_001854205.2
GCA_001854225.1
GCA_001854245.1
GCA_001854285.1
GCA_001854305.1
GCA_001854325.1
GCA_001854345.1
GCA_001854385.1
GCA_001854405.1
GCA_001854425.1
GCA_001855255.1
GCA_001855275.1
GCA_001855295.1
GCA_001855315.1
GCA_001855335.1
GCA_001855705.1
GCA_001856645.1
GCA_001856665.1
GCA_001856685.1
GCA_001856695.1
GCA_001856725.1
GCA_001857925.1
GCA_001857945.1
GCA_001857965.1
GCA_001857985.1
GCA_001858005.1
GCA_001860505.1
GCA_001865295.1
GCA_001865395.1
GCA_001865415.1
GCA_001865435.1
GCA_001865455.1
GCA_001865475.1
GCA_001865495.1
GCA_001865515.1
GCA_001865535.1
GCA_001865555.1
GCA_001865575.2
GCA_001865595.1
GCA_001865615.1
GCA_001865635.4
GCA_001865655.1
GCA_001865675.1
GCA_001865695.1
GCA_001865765.1
GCA_001865835.1
GCA_001865855.1
GCA_001866075.3
GCA_001866535.2
GCA_001866985.1
GCA_001867005.1

GCA_001867025.1
GCA_001867045.1
GCA_001867065.1
GCA_001867085.1
GCA_001867105.1
GCA_001867125.1
GCA_001867145.1
GCA_001867165.1
GCA_001870065.1
GCA_001870085.1
GCA_001870105.1
GCA_001870145.1
GCA_001870165.1
GCA_001870185.1
GCA_001870205.1
GCA_001870265.1
GCA_001870665.2
GCA_001874125.1
GCA_001874385.1
GCA_001874405.3
GCA_001874425.3
GCA_001874445.1
GCA_001874465.1
GCA_001874485.1
GCA_001874505.1
GCA_001874545.1
GCA_001874565.1
GCA_001874585.1
GCA_001874605.1
GCA_001874625.1
GCA_001874645.1
GCA_001877035.1
GCA_001877055.1
GCA_001878675.1
GCA_001879525.1
GCA_001879545.1
GCA_001879565.1
GCA_001879585.1
GCA_001879605.1
GCA_001879625.1
GCA_001879645.1
GCA_001880185.2
GCA_001880205.1
GCA_001880225.1
GCA_001880245.1
GCA_001880265.1
GCA_001880285.1
GCA_001880305.1
GCA_001880325.1
GCA_001885145.1
GCA_001885175.1
GCA_001885195.1
GCA_001885215.1
GCA_001885235.1

GCA_001885255.2
GCA_001885275.1
GCA_001886355.1
GCA_001886375.1
GCA_001886395.1
GCA_001886415.1
GCA_001886435.1
GCA_001886455.1
GCA_001886475.1
GCA_001886495.1
GCA_001886515.1
GCA_001886535.1
GCA_001886555.1
GCA_001886575.1
GCA_001886595.1
GCA_001886615.1
GCA_001886635.1
GCA_001886655.1
GCA_001886695.1
GCA_001886715.1
GCA_001886735.1
GCA_001886755.1
GCA_001886775.1
GCA_001886795.1
GCA_001886815.1
GCA_001886835.1
GCA_001886855.1
GCA_001886875.1
GCA_001886895.1
GCA_001886915.1
GCA_001886935.1
GCA_001886975.1
GCA_001886995.1
GCA_001887015.1
GCA_001887055.1
GCA_001887075.1
GCA_001887245.1
GCA_001887265.1
GCA_001887285.1
GCA_001887305.1
GCA_001887395.1
GCA_001887415.1
GCA_001887435.1
GCA_001887455.1
GCA_001887475.1
GCA_001887495.1
GCA_001887515.1
GCA_001887535.1
GCA_001887555.1
GCA_001887575.1
GCA_001887595.1
GCA_001887615.1
GCA_001887635.1
GCA_001887655.1

GCA_001887675.1
GCA_001887695.1
GCA_001887775.1
GCA_001887985.4
GCA_001887995.2
GCA_001888075.1
GCA_001888165.1
GCA_001888185.1
GCA_001888205.1
GCA_001888805.2
GCA_001888905.1
GCA_001888925.1
GCA_001888945.1
GCA_001888965.1
GCA_001888985.1
GCA_001889005.1
GCA_001889025.1
GCA_001889045.1
GCA_001889105.1
GCA_001889125.1
GCA_001889145.1
GCA_001889165.1
GCA_001889265.1
GCA_001889285.1
GCA_001889305.1
GCA_001889325.1
GCA_001889345.1
GCA_001889365.1
GCA_001889445.1
GCA_001889465.1
GCA_001889485.1
GCA_001889505.1
GCA_001889525.1
GCA_001889545.1
GCA_001889585.2
GCA_001889605.1
GCA_001889645.2
GCA_001890165.1
GCA_001890185.1
GCA_001890205.1
GCA_001890225.1
GCA_001890245.1
GCA_001890265.1
GCA_001890285.2
GCA_001890305.1
GCA_001890325.1
GCA_001890345.1
GCA_001890365.1
GCA_001890385.1
GCA_001890405.1
GCA_001890425.1
GCA_001890445.1
GCA_001891105.1
GCA_001891125.1

GCA_001895125.2
GCA_001895265.1
GCA_001895285.1
GCA_001895305.1
GCA_001895765.1
GCA_001895785.1
GCA_001895805.1
GCA_001895825.1
GCA_001895845.1
GCA_001895865.1
GCA_001895885.1
GCA_001895905.1
GCA_001895965.1
GCA_001895985.1
GCA_001896005.1
GCA_001896025.1
GCA_001896045.1
GCA_001896065.1
GCA_001896085.1
GCA_001900185.1
GCA_001900195.1
GCA_001900225.1
GCA_001900245.1
GCA_001900265.1
GCA_001900295.1
GCA_001900315.1
GCA_001900335.1
GCA_001900355.1
GCA_001900375.1
GCA_001900395.1
GCA_001900415.1
GCA_001900435.1
GCA_001900455.1
GCA_001900475.1
GCA_001900495.1
GCA_001900515.1
GCA_001900535.1
GCA_001900555.1
GCA_001900575.1
GCA_001900595.1
GCA_001900615.1
GCA_001900635.1
GCA_001900655.1
GCA_001900675.1
GCA_001900695.1
GCA_001900715.1
GCA_001900735.1
GCA_001900775.1
GCA_001900795.1
GCA_001900815.1
GCA_001900835.1
GCA_001900885.1
GCA_001900905.1
GCA_001900925.1

GCA_001900945.1
GCA_001900965.1
GCA_001900985.1
GCA_001901005.1
GCA_001901025.1
GCA_001901045.1
GCA_001901065.1
GCA_001901085.1
GCA_001901105.1
GCA_001901125.1
GCA_001901145.1
GCA_001901165.1
GCA_001901185.1
GCA_001901215.1
GCA_001901315.1
GCA_001901365.1
GCA_001901405.1
GCA_001901425.1
GCA_001901445.1
GCA_001901465.1
GCA_001901705.1
GCA_001902195.1
GCA_001902215.1
GCA_001902235.1
GCA_001902255.1
GCA_001902275.1
GCA_001902295.1
GCA_001902315.1
GCA_001902335.1
GCA_001902355.1
GCA_001902375.1
GCA_001902415.1
GCA_001902435.1
GCA_001902455.1
GCA_001902475.1
GCA_001902495.1
GCA_001902515.1
GCA_001902535.1
GCA_001902555.1
GCA_001908255.1
GCA_001908275.1
GCA_001908295.1
GCA_001908315.1
GCA_001908335.2
GCA_001908375.1
GCA_001908415.1
GCA_001908435.2
GCA_001908455.1
GCA_001908475.1
GCA_001908515.1
GCA_001908595.1
GCA_001908615.1
GCA_001908625.1
GCA_001908655.1

GCA_001908675.1
GCA_001908695.1
GCA_001908715.1
GCA_001908725.1
GCA_001908755.1
GCA_001908775.1
GCA_001908795.1
GCA_001908815.1
GCA_001908855.1
GCA_001908875.1
GCA_001908895.1
GCA_001913135.1
GCA_001913155.1
GCA_001913175.1
GCA_001913195.1
GCA_001913215.1
GCA_001913235.1
GCA_001921905.1
GCA_001921925.1
GCA_001921945.1
GCA_001921965.1
GCA_001921985.1
GCA_001922005.1
GCA_001922025.1
GCA_001922045.1
GCA_001922065.1
GCA_001922085.1
GCA_001922105.1
GCA_001922145.1
GCA_001922165.1
GCA_001922185.1
GCA_001922205.1
GCA_001922225.1
GCA_001922245.1
GCA_001922265.1
GCA_001922285.1
GCA_001922305.1
GCA_001922325.1
GCA_001922345.1
GCA_001922365.1
GCA_001922385.1
GCA_001922405.1
GCA_001922465.1
GCA_001922485.1
GCA_001922545.1
GCA_001927265.3
GCA_001927405.2
GCA_001928005.2
GCA_001928035.2
GCA_001928095.2
GCA_001928165.2
GCA_001928175.2
GCA_001928215.2
GCA_001928585.2

GCA_001928695.2
GCA_001928825.2
GCA_001928835.2
GCA_001928905.2
GCA_001928985.2
GCA_001928995.2
GCA_001929065.2
GCA_001929075.2
GCA_001929085.2
GCA_001929095.2
GCA_001929155.2
GCA_001929165.2
GCA_001929235.2
GCA_001929245.2
GCA_001931535.1
GCA_001931555.1
GCA_001931575.1
GCA_001931595.1
GCA_001931615.1
GCA_001931635.1
GCA_001931675.1
GCA_001931755.2
GCA_001931885.1
GCA_001932055.2
GCA_001932515.1
GCA_001932535.1
GCA_001932555.1
GCA_001932595.1
GCA_001932615.1
GCA_001932635.1
GCA_001932675.1
GCA_001932695.1
GCA_001932715.1
GCA_001932735.1
GCA_001932755.1
GCA_001932775.1
GCA_001932795.1
GCA_001932815.1
GCA_001932835.1
GCA_001932855.1
GCA_001932875.1
GCA_001932895.1
GCA_001932915.1
GCA_001932935.1
GCA_001932955.1
GCA_001936035.1
GCA_001936175.1
GCA_001936195.1
GCA_001936215.1
GCA_001936235.1
GCA_001936255.1
GCA_001936295.1
GCA_001936315.1
GCA_001936335.1

GCA_001936355.1
GCA_001936375.1
GCA_001936395.1
GCA_001936415.1
GCA_001938665.1
GCA_001938685.1
GCA_001938705.1
GCA_001938725.1
GCA_001939885.2
GCA_001940525.2
GCA_001941345.1
GCA_001941365.1
GCA_001941385.1
GCA_001941405.1
GCA_001941425.1
GCA_001941445.1
GCA_001941465.1
GCA_001941485.1
GCA_001941505.1
GCA_001941525.1
GCA_001941565.1
GCA_001941585.1
GCA_001941605.1
GCA_001941625.1
GCA_001941645.1
GCA_001941765.1
GCA_001941785.1
GCA_001941805.1
GCA_001941825.1
GCA_001941845.1
GCA_001941865.1
GCA_001941885.1
GCA_001941905.1
GCA_001941925.1
GCA_001941945.1
GCA_001942005.2
GCA_001942025.1
GCA_001945665.1
GCA_001950995.1
GCA_001951015.1
GCA_001951075.2
GCA_001951095.1
GCA_001951155.1
GCA_001951175.1
GCA_001951235.1
GCA_001951255.1
GCA_001951275.1
GCA_001951295.1
GCA_001951315.1
GCA_001951335.1
GCA_001952735.1
GCA_001952755.1
GCA_001952775.1
GCA_001952795.1

GCA_001952815.1
GCA_001952835.1
GCA_001952875.1
GCA_001952895.1
GCA_001952915.1
GCA_001952935.1
GCA_001952955.1
GCA_001952995.1
GCA_001953015.1
GCA_001953035.1
GCA_001953055.1
GCA_001953135.1
GCA_001953155.1
GCA_001953175.1
GCA_001953195.1
GCA_001953215.1
GCA_001953235.1
GCA_001953255.1
GCA_001953675.1
GCA_001953695.1
GCA_001953935.1
GCA_001953955.1
GCA_001955695.1
GCA_001955715.1
GCA_001955735.1
GCA_001956655.2
GCA_001956675.1
GCA_001956695.1
GCA_001956715.1
GCA_001956735.1
GCA_001956755.1
GCA_001956795.1
GCA_001956815.1
GCA_001956965.1
GCA_001956985.1
GCA_001969085.2
GCA_001969095.2
GCA_001969145.2
GCA_001969165.3
GCA_001969245.1
GCA_001969265.1
GCA_001969305.1
GCA_001969325.1
GCA_001969345.1
GCA_001969365.1
GCA_001969385.1
GCA_001969405.1
GCA_001969445.1
GCA_001970835.1
GCA_001971475.2
GCA_001971565.1
GCA_001971585.1
GCA_001971605.1
GCA_001971625.1

GCA_001971645.1
GCA_001971665.1
GCA_001971685.1
GCA_001971725.1
GCA_001971745.1
GCA_001971765.1
GCA_001971785.1
GCA_001971805.1
GCA_001972545.2
GCA_001972625.2
GCA_001973275.2
GCA_001973785.2
GCA_001974965.1
GCA_001974985.1
GCA_001975005.1
GCA_001975025.1
GCA_001975045.1
GCA_001975065.1
GCA_001975085.1
GCA_001975105.1
GCA_001975125.1
GCA_001975145.1
GCA_001975165.1
GCA_001975205.1
GCA_001975225.1
GCA_001975245.1
GCA_001975265.1
GCA_001975285.1
GCA_001975305.1
GCA_001975325.1
GCA_001975345.1
GCA_001975365.1
GCA_001975385.1
GCA_001975405.1
GCA_001975425.1
GCA_001975445.1
GCA_001975515.1
GCA_001975555.1
GCA_001975605.1
GCA_001975625.1
GCA_001975645.1
GCA_001975665.1
GCA_001975685.1
GCA_001975705.1
GCA_001975955.2
GCA_001980585.2
GCA_001983935.1
GCA_001983955.1
GCA_001983975.1
GCA_001983995.1
GCA_001984015.1
GCA_001984035.1
GCA_001984445.1
GCA_001984465.1

GCA_001984825.2
GCA_001984925.1
GCA_001984945.1
GCA_001984965.1
GCA_001984985.1
GCA_001985005.1
GCA_001985025.1
GCA_001985045.1
GCA_001985065.1
GCA_001985085.1
GCA_001985105.1
GCA_001985125.1
GCA_001985145.1
GCA_001985165.1
GCA_001985205.1
GCA_001985225.1
GCA_001985245.1
GCA_001985265.1
GCA_001985285.1
GCA_001985305.1
GCA_001985325.1
GCA_001985345.1
GCA_001985365.1
GCA_001985385.1
GCA_001985405.1
GCA_001985425.1
GCA_001985445.1
GCA_001985465.1
GCA_001985485.1
GCA_001985505.1
GCA_001985525.1
GCA_001985545.1
GCA_001985565.1
GCA_001985585.1
GCA_001985605.1
GCA_001985625.1
GCA_001985645.1
GCA_001985665.1
GCA_001985685.1
GCA_001985705.1
GCA_001985725.1
GCA_001985745.1
GCA_001985765.1
GCA_001985785.1
GCA_001985805.1
GCA_001985825.1
GCA_001985845.1
GCA_001985865.1
GCA_001985885.1
GCA_001985905.1
GCA_001985925.1
GCA_001985945.1
GCA_001985965.1
GCA_001985985.1

GCA_001986005.1
GCA_001986025.1
GCA_001986045.1
GCA_001986065.1
GCA_001986085.1
GCA_001986105.1
GCA_001986135.1
GCA_001986155.1
GCA_001986175.1
GCA_001986195.1
GCA_001986215.1
GCA_001986235.1
GCA_001986255.1
GCA_001986275.1
GCA_001986295.1
GCA_001986315.1
GCA_001986335.1
GCA_001986355.1
GCA_001986375.1
GCA_001986415.1
GCA_001986435.1
GCA_001986455.1
GCA_001986475.1
GCA_001986495.1
GCA_001986515.1
GCA_001986535.1
GCA_001986555.1
GCA_001986575.1
GCA_001986595.1
GCA_001986615.1
GCA_001986635.1
GCA_001986655.1
GCA_001986675.1
GCA_001986695.1
GCA_001986715.1
GCA_001986735.1
GCA_001986755.1
GCA_001986775.1
GCA_001986795.1
GCA_001986815.1
GCA_001986835.1
GCA_001986855.1
GCA_001986875.1
GCA_001986895.1
GCA_001986915.1
GCA_001986935.1
GCA_001986955.1
GCA_001986975.1
GCA_001986995.1
GCA_001987015.1
GCA_001987035.1
GCA_001987055.1
GCA_001987075.1
GCA_001987095.1

GCA_001987115.1
GCA_001987135.1
GCA_001987155.1
GCA_001987175.1
GCA_001987195.1
GCA_001987215.1
GCA_001987235.1
GCA_001987255.1
GCA_001987275.1
GCA_001987295.1
GCA_001987315.1
GCA_001987335.1
GCA_001987355.1
GCA_001987375.1
GCA_001987395.1
GCA_001987415.1
GCA_001987435.1
GCA_001987455.1
GCA_001987475.1
GCA_001987495.1
GCA_001987515.1
GCA_001987535.1
GCA_001987555.1
GCA_001987575.1
GCA_001987595.1
GCA_001987615.1
GCA_001987635.1
GCA_001987655.1
GCA_001987675.1
GCA_001987695.1
GCA_001987715.1
GCA_001987735.1
GCA_001987755.1
GCA_001987775.1
GCA_001987795.1
GCA_001987815.1
GCA_001987835.1
GCA_001987855.1
GCA_001987875.1
GCA_001987895.1
GCA_001987915.1
GCA_001987935.1
GCA_001987955.1
GCA_001987975.1
GCA_001987995.1
GCA_001988015.1
GCA_001988035.1
GCA_001988055.1
GCA_001988075.1
GCA_001988095.1
GCA_001988115.1
GCA_001988135.1
GCA_001988155.1
GCA_001988175.1

GCA_001988195.1
GCA_001988215.1
GCA_001988235.1
GCA_001988255.1
GCA_001988275.1
GCA_001988295.1
GCA_001988315.1
GCA_001988335.1
GCA_001988355.1
GCA_001988375.1
GCA_001988395.1
GCA_001988415.1
GCA_001988435.1
GCA_001988455.1
GCA_001988475.1
GCA_001988495.1
GCA_001988515.1
GCA_001988535.1
GCA_001988555.1
GCA_001988575.1
GCA_001988595.1
GCA_001988615.1
GCA_001988635.1
GCA_001988655.1
GCA_001988675.1
GCA_001988695.1
GCA_001988715.1
GCA_001988735.1
GCA_001988755.1
GCA_001988775.1
GCA_001988795.1
GCA_001988815.1
GCA_001988835.1
GCA_001988855.1
GCA_001988875.1
GCA_001988895.1
GCA_001988915.1
GCA_001988935.1
GCA_001988955.1
GCA_001988995.1
GCA_001989015.1
GCA_001989035.1
GCA_001989055.1
GCA_001989075.1
GCA_001989095.1
GCA_001989115.1
GCA_001989135.1
GCA_001989155.1
GCA_001989175.1
GCA_001989195.1
GCA_001989215.1
GCA_001989235.1
GCA_001989255.1
GCA_001989275.1

GCA_001989295.1
GCA_001989315.1
GCA_001989335.1
GCA_001989475.1
GCA_001989495.1
GCA_001989515.1
GCA_001989535.1
GCA_001989555.1
GCA_001989575.1
GCA_001989595.1
GCA_001989635.1
GCA_001990145.1
GCA_001990205.1
GCA_001990225.1
GCA_001990245.1
GCA_001991075.2
GCA_001991095.1
GCA_001995825.2
GCA_001996365.2
GCA_001997295.1
GCA_001997345.1
GCA_001997385.1
GCA_001998765.1
GCA_001998805.1
GCA_001998865.1
GCA_001998885.1
GCA_001998915.1
GCA_001998945.1
GCA_001998985.1
GCA_001999005.1
GCA_001999025.1
GCA_001999045.1
GCA_001999065.1
GCA_001999085.1
GCA_001999105.1
GCA_001999125.1
GCA_001999145.1
GCA_001999165.1
GCA_001999185.1
GCA_001999205.1
GCA_001999225.1
GCA_001999245.1
GCA_001999785.1
GCA_001999825.1
GCA_001999885.1
GCA_001999905.1
GCA_001999945.1
GCA_001999965.1
GCA_001999985.1
GCA_002000005.1
GCA_002000565.1
GCA_002000585.1
GCA_002000605.1
GCA_002000625.1

GCA_002000645.1
GCA_002000665.1
GCA_002000685.1
GCA_002000705.1
GCA_002000725.1
GCA_002000745.1
GCA_002000765.1
GCA_002000785.1
GCA_002000805.1
GCA_002000825.1
GCA_002000845.1
GCA_002000865.1
GCA_002000885.1
GCA_002000985.1
GCA_002002865.1
GCA_002002885.1
GCA_002002905.1
GCA_002002925.1
GCA_002003265.1
GCA_002003285.1
GCA_002003305.1
GCA_002003325.1
GCA_002003345.1
GCA_002003365.1
GCA_002003385.1
GCA_002005145.1
GCA_002005165.1
GCA_002005185.1
GCA_002005205.3
GCA_002005225.1
GCA_002005245.1
GCA_002005285.1
GCA_002005305.1
GCA_002005325.1
GCA_002005345.1
GCA_002005365.1
GCA_002005385.2
GCA_002005405.1
GCA_002005425.1
GCA_002005445.1
GCA_002005465.1
GCA_002005485.1
GCA_002005525.1
GCA_002006175.2
GCA_002006215.2
GCA_002006235.2
GCA_002006355.2
GCA_002006545.1
GCA_002006565.1
GCA_002007065.3
GCA_002007485.1
GCA_002007505.1
GCA_002007565.1
GCA_002007585.1

GCA_002007605.1
GCA_002007625.1
GCA_002007645.1
GCA_002007685.1
GCA_002007705.1
GCA_002007725.1
GCA_002007765.1
GCA_002007785.1
GCA_002007805.1
GCA_002007825.1
GCA_002007845.1
GCA_002007865.1
GCA_002007885.1
GCA_002007905.1
GCA_002008305.4
GCA_002009055.1
GCA_002009075.1
GCA_002009115.1
GCA_002009175.1
GCA_002009195.1
GCA_002009215.1
GCA_002009235.1
GCA_002009255.1
GCA_002009275.1
GCA_002009295.1
GCA_002009315.1
GCA_002009335.2
GCA_002009355.1
GCA_002009375.1
GCA_002009385.1
GCA_002009415.1
GCA_002009425.1
GCA_002011925.2
GCA_002011945.1
GCA_002011965.1
GCA_002011985.1
GCA_002012005.1
GCA_002012025.1
GCA_002012045.1
GCA_002012065.1
GCA_002012085.1
GCA_002012105.1
GCA_002012125.1
GCA_002012145.1
GCA_002012165.1
GCA_002012205.1
GCA_002012225.1
GCA_002012245.1
GCA_002012265.1
GCA_002012285.1
GCA_002012305.1
GCA_002012365.1
GCA_002018515.1
GCA_002019265.2

GCA_002021755.1
GCA_002021775.1
GCA_002021795.1
GCA_002021815.1
GCA_002021875.1
GCA_002021925.1
GCA_002021945.1
GCA_002021965.1
GCA_002021985.1
GCA_002022005.1
GCA_002022025.1
GCA_002022045.1
GCA_002022065.1
GCA_002022085.1
GCA_002022105.1
GCA_002022125.1
GCA_002022145.1
GCA_002022225.1
GCA_002022485.1
GCA_002022505.1
GCA_002022585.1
GCA_002022605.1
GCA_002022665.1
GCA_002022745.1
GCA_002023005.1
GCA_002023235.2
GCA_002023665.2
GCA_002024185.1
GCA_002024205.1
GCA_002024225.1
GCA_002024245.1
GCA_002024265.1
GCA_002024285.1
GCA_002024305.1
GCA_002024325.1
GCA_002024345.1
GCA_002024615.2
GCA_002024805.1
GCA_002024825.1
GCA_002024845.1
GCA_002024865.1
GCA_002025005.1
GCA_002025025.1
GCA_002025045.1
GCA_002025065.1
GCA_002025105.1
GCA_002025125.1
GCA_002025145.1
GCA_002025165.1
GCA_002025185.1
GCA_002025205.1
GCA_002025225.1
GCA_002025605.1
GCA_002025625.1

GCA_002025645.1
GCA_002025665.1
GCA_002025685.1
GCA_002025705.1
GCA_002025725.1
GCA_002028285.1
GCA_002028305.1
GCA_002028325.1
GCA_002028345.1
GCA_002028365.1
GCA_002028385.1
GCA_002028405.1
GCA_002037565.1
GCA_002043005.1
GCA_002043025.1
GCA_002043045.1
GCA_002055515.1
GCA_002055535.1
GCA_002055565.1
GCA_002055605.1
GCA_002055635.1
GCA_002055685.1
GCA_002055735.1
GCA_002055765.1
GCA_002055855.1
GCA_002055965.1
GCA_002056065.1
GCA_002056145.1
GCA_002056295.1
GCA_002056385.1
GCA_002056475.1
GCA_002056635.1
GCA_002056725.1
GCA_002056795.1
GCA_002057245.1
GCA_002057355.1
GCA_002057455.1
GCA_002057535.1
GCA_002058765.1
GCA_002059225.1
GCA_002067135.1
GCA_002068115.1
GCA_002068135.1
GCA_002068155.1
GCA_002068175.1
GCA_002068675.1
GCA_002068975.1
GCA_002069155.1
GCA_002071765.2
GCA_002072065.1
GCA_002072655.1
GCA_002072695.1
GCA_002072715.1
GCA_002072735.1

GCA_002072755.1
GCA_002072775.2
GCA_002072815.1
GCA_002073215.2
GCA_002073235.2
GCA_002073255.2
GCA_002073275.2
GCA_002073295.2
GCA_002073315.2
GCA_002073335.2
GCA_002073355.2
GCA_002073375.2
GCA_002073395.2
GCA_002073415.2
GCA_002073435.2
GCA_002073455.2
GCA_002073475.2
GCA_002073495.2
GCA_002073515.2
GCA_002073535.2
GCA_002073555.2
GCA_002073575.2
GCA_002073595.2
GCA_002073615.2
GCA_002073635.2
GCA_002073655.2
GCA_002073675.2
GCA_002073695.2
GCA_002073715.2
GCA_002073735.2
GCA_002073775.2
GCA_002073795.2
GCA_002073815.2
GCA_002073835.2
GCA_002073975.1
GCA_002073995.1
GCA_002074035.1
GCA_002074055.1
GCA_002074075.1
GCA_002074095.1
GCA_002074115.1
GCA_002074135.1
GCA_002074155.1
GCA_002075065.1
GCA_002075105.1
GCA_002075285.3
GCA_002075795.1
GCA_002075815.1
GCA_002076875.1
GCA_002076895.1
GCA_002076915.1
GCA_002076935.1
GCA_002076955.1
GCA_002077155.1

GCA_002077175.1
GCA_002077195.1
GCA_002077215.1
GCA_002078255.1
GCA_002078275.1
GCA_002078295.1
GCA_002078315.1
GCA_002078335.1
GCA_002078375.2
GCA_002078395.2
GCA_002078415.1
GCA_002078435.1
GCA_002078455.2
GCA_002078475.3
GCA_002078495.2
GCA_002078765.2
GCA_002078855.1
GCA_002078895.1
GCA_002078915.2
GCA_002078935.2
GCA_002078955.1
GCA_002078975.2
GCA_002078995.3
GCA_002079225.1
GCA_002079245.1
GCA_002079265.1
GCA_002079285.1
GCA_002079305.1
GCA_002079945.1
GCA_002079965.1
GCA_002080065.1
GCA_002080125.1
GCA_002080395.1
GCA_002080415.1
GCA_002080435.1
GCA_002080455.1
GCA_002080475.1
GCA_002081995.1
GCA_002082015.1
GCA_002082155.1
GCA_002082175.1
GCA_002082195.1
GCA_002082215.1
GCA_002082565.1
GCA_002082605.1
GCA_002082625.2
GCA_002082645.1
GCA_002082665.1
GCA_002082685.1
GCA_002082705.1
GCA_002082725.1
GCA_002082745.1
GCA_002082765.1
GCA_002082785.1

GCA_002082805.1
GCA_002082825.1
GCA_002082845.1
GCA_002082865.1
GCA_002082885.1
GCA_002085525.1
GCA_002085605.1
GCA_002085695.1
GCA_002085715.1
GCA_002085735.1
GCA_002085755.1
GCA_002085925.2
GCA_002088315.1
GCA_002088335.1
GCA_002088355.1
GCA_002088995.1
GCA_002089035.2
GCA_002089055.1
GCA_002089075.2
GCA_002089095.2
GCA_002089115.2
GCA_002090195.1
GCA_002090215.1
GCA_002090235.1
GCA_002090275.1
GCA_002090295.1
GCA_002090315.1
GCA_002090335.1
GCA_002090355.1
GCA_002091395.1
GCA_002094405.3
GCA_002094935.1
GCA_002094955.1
GCA_002094975.1
GCA_002094995.1
GCA_002095975.1
GCA_002095995.1
GCA_002096015.1
GCA_002096035.1
GCA_002096055.1
GCA_002096095.1
GCA_002097535.1
GCA_002097595.2
GCA_002097715.1
GCA_002101275.1
GCA_002101335.1
GCA_002101355.1
GCA_002101375.1
GCA_002101395.1
GCA_002102455.2
GCA_002103235.2
GCA_002104335.1
GCA_002104575.1
GCA_002104595.1

GCA_002104615.1
GCA_002105535.1
GCA_002105555.1
GCA_002105595.1
GCA_002105615.1
GCA_002105635.1
GCA_002105655.1
GCA_002105675.1
GCA_002105695.1
GCA_002105715.1
GCA_002105735.1
GCA_002105755.1
GCA_002105775.1
GCA_002108455.1
GCA_002109325.1
GCA_002109345.1
GCA_002109365.1
GCA_002109385.1
GCA_002109405.1
GCA_002109425.1
GCA_002110945.1
GCA_002110965.1
GCA_002111005.1
GCA_002111025.1
GCA_002111045.1
GCA_002111085.1
GCA_002111105.1
GCA_002111125.1
GCA_002111145.1
GCA_002111165.1
GCA_002111205.1
GCA_002111225.1
GCA_002111245.1
GCA_002111265.1
GCA_002111285.1
GCA_002111305.1
GCA_002111325.1
GCA_002111345.1
GCA_002111365.1
GCA_002111385.1
GCA_002111405.1
GCA_002113825.1
GCA_002113845.3
GCA_002113865.1
GCA_002116675.1
GCA_002116715.2
GCA_002116735.1
GCA_002116755.1
GCA_002116775.1
GCA_002116795.1
GCA_002116815.1
GCA_002116835.1
GCA_002116855.1
GCA_002116885.1

GCA_002116905.1
GCA_002116925.1
GCA_002116955.1
GCA_002117005.1
GCA_002117085.1
GCA_002117105.1
GCA_002117145.1
GCA_002117165.1
GCA_002117185.1
GCA_002117205.1
GCA_002117225.1
GCA_002117245.1
GCA_002117265.1
GCA_002117285.1
GCA_002117305.1
GCA_002117325.1
GCA_002117345.1
GCA_002117375.1
GCA_002117405.1
GCA_002117425.1
GCA_002117445.1
GCA_002117485.1
GCA_002117725.1
GCA_002117815.2
GCA_002117875.1
GCA_002118095.1
GCA_002119445.1
GCA_002119485.1
GCA_002119505.2
GCA_002119525.1
GCA_002119545.1
GCA_002119565.1
GCA_002119585.1
GCA_002119605.1
GCA_002119625.1
GCA_002119645.1
GCA_002119665.1
GCA_002119685.1
GCA_002119705.1
GCA_002119725.1
GCA_002119745.1
GCA_002119765.1
GCA_002119805.1
GCA_002119825.1
GCA_002119845.1
GCA_002127965.1
GCA_002127985.1
GCA_002128005.1
GCA_002128025.1
GCA_002128045.1
GCA_002128065.1
GCA_002128085.1
GCA_002128105.1
GCA_002128125.1

GCA_002128145.1
GCA_002128165.1
GCA_002128185.1
GCA_002128205.1
GCA_002128235.1
GCA_002128265.1
GCA_002128285.1
GCA_002128305.1
GCA_002128325.1
GCA_002128345.1
GCA_002128365.1
GCA_002128385.1
GCA_002135175.2
GCA_002138395.1
GCA_002138415.1
GCA_002139855.1
GCA_002139875.1
GCA_002139895.1
GCA_002139915.1
GCA_002139935.1
GCA_002139955.1
GCA_002139975.1
GCA_002139995.1
GCA_002140035.1
GCA_002140055.1
GCA_002140115.1
GCA_002142475.1
GCA_002142495.1
GCA_002142575.1
GCA_002142595.1
GCA_002142615.1
GCA_002142635.1
GCA_002142655.1
GCA_002142675.1
GCA_002142695.1
GCA_002142715.1
GCA_002147855.1
GCA_002148175.1
GCA_002148195.1
GCA_002148215.1
GCA_002148235.1
GCA_002148255.1
GCA_002149065.1
GCA_002149085.1
GCA_002149105.1
GCA_002150905.1
GCA_002151445.1
GCA_002151465.1
GCA_002151485.1
GCA_002151505.1
GCA_002151545.1
GCA_002155145.1
GCA_002155165.1
GCA_002155245.1

GCA_002155265.1
GCA_002156725.1
GCA_002156745.1
GCA_002156765.1
GCA_002156785.1
GCA_002156805.1
GCA_002156825.1
GCA_002156845.1
GCA_002157125.1
GCA_002157145.1
GCA_002157165.1
GCA_002157185.1
GCA_002157205.1
GCA_002157225.2
GCA_002157245.1
GCA_002157265.1
GCA_002157365.2
GCA_002157735.2
GCA_002157835.1
GCA_002157855.1
GCA_002157875.1
GCA_002157895.1
GCA_002158845.1
GCA_002158865.1
GCA_002158885.1
GCA_002158905.1
GCA_002158925.1
GCA_002158945.1
GCA_002162015.1
GCA_002162035.1
GCA_002162055.1
GCA_002162115.2
GCA_002162135.1
GCA_002162175.1
GCA_002162195.1
GCA_002162215.1
GCA_002162235.1
GCA_002162255.1
GCA_002162275.1
GCA_002162295.1
GCA_002162315.1
GCA_002162335.1
GCA_002162355.1
GCA_002162375.1
GCA_002163545.1
GCA_002163565.1
GCA_002163585.1
GCA_002163605.1
GCA_002163625.1
GCA_002163655.1
GCA_002163695.1
GCA_002163735.1
GCA_002163755.1
GCA_002163775.1

GCA_002163795.1
GCA_002163895.1
GCA_002163915.1
GCA_002163935.1
GCA_002163975.1
GCA_002163995.1
GCA_002164595.2
GCA_002164635.4
GCA_002164645.3
GCA_002164695.2
GCA_002164805.3
GCA_002164835.4
GCA_002164855.2
GCA_002164865.2
GCA_002164975.2
GCA_002165095.2
GCA_002165115.2
GCA_002165245.3
GCA_002165255.2
GCA_002165295.2
GCA_002165305.2
GCA_002165345.2
GCA_002165375.2
GCA_002166795.1
GCA_002173495.1
GCA_002173515.1
GCA_002173535.1
GCA_002173555.1
GCA_002173575.1
GCA_002173595.1
GCA_002173615.1
GCA_002173635.1
GCA_002173655.1
GCA_002173675.1
GCA_002173695.1
GCA_002173715.1
GCA_002173735.1
GCA_002173755.1
GCA_002173775.1
GCA_002174195.1
GCA_002174215.1
GCA_002174235.1
GCA_002174255.1
GCA_002174695.2
GCA_002176815.1
GCA_002176835.1
GCA_002176855.1
GCA_002180055.1
GCA_002180075.1
GCA_002180095.1
GCA_002180115.1
GCA_002180135.1
GCA_002180155.1
GCA_002180175.1

GCA_002180195.1
GCA_002180215.1
GCA_002180235.1
GCA_002180255.1
GCA_002180275.1
GCA_002180295.1
GCA_002180315.1
GCA_002180335.1
GCA_002184245.1
GCA_002187935.3
GCA_002189545.2
GCA_002189675.2
GCA_002189695.1
GCA_002191235.1
GCA_002191295.1
GCA_002191335.1
GCA_002191355.1
GCA_002191375.1
GCA_002191455.1
GCA_002191575.1
GCA_002191615.1
GCA_002191655.1
GCA_002191675.1
GCA_002191755.1
GCA_002191915.1
GCA_002192095.1
GCA_002192155.1
GCA_002192215.1
GCA_002192235.1
GCA_002192255.1
GCA_002192275.1
GCA_002192295.1
GCA_002192315.1
GCA_002192335.1
GCA_002192355.1
GCA_002192375.1
GCA_002192395.1
GCA_002192415.1
GCA_002192435.1
GCA_002192455.1
GCA_002192475.1
GCA_002192495.1
GCA_002192515.1
GCA_002192595.1
GCA_002193095.1
GCA_002196475.1
GCA_002196495.1
GCA_002196515.1
GCA_002196535.1
GCA_002196555.1
GCA_002197025.1
GCA_002197045.1
GCA_002197065.1
GCA_002197085.1

GCA_002197105.1
GCA_002197125.1
GCA_002197145.1
GCA_002197165.1
GCA_002197205.1
GCA_002197225.1
GCA_002197245.1
GCA_002197265.1
GCA_002197285.1
GCA_002197305.1
GCA_002197325.1
GCA_002197345.1
GCA_002197365.1
GCA_002197385.1
GCA_002197405.1
GCA_002197425.1
GCA_002197445.1
GCA_002197465.1
GCA_002197485.1
GCA_002197505.1
GCA_002197645.1
GCA_002201795.1
GCA_002201815.1
GCA_002201835.1
GCA_002201855.1
GCA_002201875.1
GCA_002201935.1
GCA_002201955.1
GCA_002201975.1
GCA_002201995.1
GCA_002202015.1
GCA_002202035.1
GCA_002202055.1
GCA_002202075.1
GCA_002202095.1
GCA_002202135.1
GCA_002202155.1
GCA_002202175.1
GCA_002202195.1
GCA_002202215.1
GCA_002202235.1
GCA_002202255.1
GCA_002202275.1
GCA_002202295.1
GCA_002204555.1
GCA_002204575.1
GCA_002204775.1
GCA_002204915.1
GCA_002205315.1
GCA_002205335.1
GCA_002205355.1
GCA_002205375.1
GCA_002205495.2
GCA_002205515.1

GCA_002205535.1
GCA_002205575.1
GCA_002205595.1
GCA_002205715.1
GCA_002205735.1
GCA_002205755.1
GCA_002205775.2
GCA_002208095.1
GCA_002208115.1
GCA_002208135.1
GCA_002208175.1
GCA_002208195.1
GCA_002208215.1
GCA_002208235.1
GCA_002208255.1
GCA_002208315.1
GCA_002208645.1
GCA_002208665.2
GCA_002208685.2
GCA_002208705.2
GCA_002208725.2
GCA_002208745.2
GCA_002208765.2
GCA_002208785.2
GCA_002208805.2
GCA_002208825.2
GCA_002208845.2
GCA_002208865.2
GCA_002208885.2
GCA_002208905.2
GCA_002208925.2
GCA_002208945.2
GCA_002208965.2
GCA_002208985.3
GCA_002209005.1
GCA_002209025.1
GCA_002209045.1
GCA_002209065.1
GCA_002209085.2
GCA_002209105.2
GCA_002209125.2
GCA_002209145.2
GCA_002209165.2
GCA_002209185.2
GCA_002209205.2
GCA_002209225.2
GCA_002209245.2
GCA_002209265.2
GCA_002209305.1
GCA_002209325.1
GCA_002209355.2
GCA_002209385.1
GCA_002209405.1
GCA_002209425.1

GCA_002209445.1
GCA_002209725.2
GCA_002210065.1
GCA_002211505.1
GCA_002211565.1
GCA_002211585.1
GCA_002211605.1
GCA_002211625.1
GCA_002211645.1
GCA_002211685.1
GCA_002211725.1
GCA_002211745.1
GCA_002211765.1
GCA_002211785.1
GCA_002211825.1
GCA_002211845.1
GCA_002211865.1
GCA_002211885.1
GCA_002211905.1
GCA_002211925.1
GCA_002211965.1
GCA_002211985.1
GCA_002212005.1
GCA_002212025.1
GCA_002213505.1
GCA_002213545.1
GCA_002213565.1
GCA_002213585.1
GCA_002213605.1
GCA_002213625.1
GCA_002213645.1
GCA_002213665.1
GCA_002213685.1
GCA_002213705.1
GCA_002213725.1
GCA_002213745.1
GCA_002213765.1
GCA_002213785.1
GCA_002213805.1
GCA_002213825.1
GCA_002213845.1
GCA_002213865.1
GCA_002213885.1
GCA_002213905.1
GCA_002213925.1
GCA_002213945.1
GCA_002213965.1
GCA_002213985.1
GCA_002214005.1
GCA_002214045.1
GCA_002214065.1
GCA_002214085.1
GCA_002214105.1
GCA_002214125.1

GCA_002214145.1
GCA_002214205.1
GCA_002214245.1
GCA_002214265.1
GCA_002214285.1
GCA_002214305.1
GCA_002214345.1
GCA_002214395.1
GCA_002214425.1
GCA_002214445.1
GCA_002214625.2
GCA_002214645.1
GCA_002214665.1
GCA_002214705.1
GCA_002214725.1
GCA_002214745.2
GCA_002214765.1
GCA_002214785.1
GCA_002214805.1
GCA_002214825.1
GCA_002215055.1
GCA_002215075.1
GCA_002215095.1
GCA_002215115.1
GCA_002215135.1
GCA_002215155.1
GCA_002215175.1
GCA_002215195.1
GCA_002215215.1
GCA_002215345.1
GCA_002215385.1
GCA_002215425.1
GCA_002215465.1
GCA_002215535.1
GCA_002215585.1
GCA_002216065.1
GCA_002216085.1
GCA_002216125.1
GCA_002216145.1
GCA_002216685.1
GCA_002216705.1
GCA_002216755.1
GCA_002216775.1
GCA_002216795.1
GCA_002216815.1
GCA_002216835.1
GCA_002216855.1
GCA_002216875.1
GCA_002217795.2
GCA_002218025.2
GCA_002218045.2
GCA_002218195.1
GCA_002218245.1
GCA_002218265.1

GCA_002218285.1
GCA_002219245.1
GCA_002219265.1
GCA_002219285.1
GCA_002220095.1
GCA_002220115.1
GCA_002220135.1
GCA_002220155.1
GCA_002220175.1
GCA_002220195.1
GCA_002220215.1
GCA_002220265.1
GCA_002220285.1
GCA_002220325.1
GCA_002220345.1
GCA_002220465.1
GCA_002220485.1
GCA_002220535.1
GCA_002220555.1
GCA_002220575.1
GCA_002220595.1
GCA_002220615.1
GCA_002220635.1
GCA_002220655.1
GCA_002220695.1
GCA_002220735.1
GCA_002220755.1
GCA_002220775.1
GCA_002220795.1
GCA_002220815.1
GCA_002220985.3
GCA_002221505.1
GCA_002221525.1
GCA_002222555.1
GCA_002222575.1
GCA_002222595.2
GCA_002222615.2
GCA_002222635.1
GCA_002222655.1
GCA_002223805.1
GCA_002224265.1
GCA_002224285.1
GCA_002224305.1
GCA_002224325.1
GCA_002224345.1
GCA_002224365.1
GCA_002224385.1
GCA_002224405.1
GCA_002224425.1
GCA_002224465.1
GCA_002224505.1
GCA_002224525.1
GCA_002224545.1
GCA_002224565.1

GCA_002224585.2
GCA_002224605.1
GCA_002224625.1
GCA_002224645.1
GCA_002232005.2
GCA_002234355.1
GCA_002234455.1
GCA_002234475.1
GCA_002234495.1
GCA_002234515.1
GCA_002234535.1
GCA_002234575.2
GCA_002234735.2
GCA_002234755.2
GCA_002234775.1
GCA_002234795.1
GCA_002234855.1
GCA_002236855.1
GCA_002236875.1
GCA_002236895.1
GCA_002237285.1
GCA_002237305.1
GCA_002237325.1
GCA_002237405.1
GCA_002237425.1
GCA_002237445.1
GCA_002237465.1
GCA_002237515.1
GCA_002237535.1
GCA_002237555.1
GCA_002237575.1
GCA_002237595.1
GCA_002237615.1
GCA_002238275.1
GCA_002238295.1
GCA_002238315.1
GCA_002238335.1
GCA_002238355.1
GCA_002238375.1
GCA_002238395.1
GCA_002239895.2
GCA_002240035.1
GCA_002240055.1
GCA_002240075.1
GCA_002240095.1
GCA_002240115.1
GCA_002240135.1
GCA_002240155.1
GCA_002240185.1
GCA_002240205.1
GCA_002240295.1
GCA_002240315.1
GCA_002240335.1
GCA_002240355.1

GCA_002240375.2
GCA_002240395.1
GCA_002240415.1
GCA_002240435.1
GCA_002240455.1
GCA_002240475.1
GCA_002240495.1
GCA_002240515.1
GCA_002242175.1
GCA_002242615.1
GCA_002243285.1
GCA_002243305.1
GCA_002243325.2
GCA_002243345.3
GCA_002243365.1
GCA_002243425.1
GCA_002243445.1
GCA_002243495.1
GCA_002243515.1
GCA_002243535.1
GCA_002243555.1
GCA_002243625.1
GCA_002243645.1
GCA_002243665.1
GCA_002243685.1
GCA_002246595.3
GCA_002248115.3
GCA_002249955.1
GCA_002249975.3
GCA_002250035.1
GCA_002250055.1
GCA_002250075.1
GCA_002250095.2
GCA_002250115.1
GCA_002250745.2
GCA_002250885.2
GCA_002250905.2
GCA_002250925.2
GCA_002250945.2
GCA_002250965.2
GCA_002251005.2
GCA_002251025.2
GCA_002251045.2
GCA_002251055.2
GCA_002251115.2
GCA_002251875.3
GCA_002257505.1
GCA_002257545.1
GCA_002257565.1
GCA_002257585.1
GCA_002257605.1
GCA_002257625.1
GCA_002257765.2
GCA_002257935.4

GCA_002258005.2
GCA_002258055.3
GCA_002258105.2
GCA_002258145.4
GCA_002258175.2
GCA_002260685.1
GCA_002262955.1
GCA_002262975.1
GCA_002262995.1
GCA_002263015.1
GCA_002263255.2
GCA_002263495.1
GCA_002263515.1
GCA_002265865.2
GCA_002266085.2
GCA_002266205.2
GCA_002266325.2
GCA_002266435.2
GCA_002266465.2
GCA_002269175.1
GCA_002269195.1
GCA_002269255.1
GCA_002269275.1
GCA_002269295.1
GCA_002269325.1
GCA_002269345.1
GCA_002269365.1
GCA_002269385.1
GCA_002276165.1
GCA_002277895.1
GCA_002277915.1
GCA_002277935.1
GCA_002277955.1
GCA_002277975.1
GCA_002277995.2
GCA_002278015.2
GCA_002278035.1
GCA_002278055.1
GCA_002278075.1
GCA_002278095.1
GCA_002278115.2
GCA_002278135.2
GCA_002282915.1
GCA_002284855.1
GCA_002284875.1
GCA_002284895.1
GCA_002284915.1
GCA_002285455.1
GCA_002285475.1
GCA_002285495.1
GCA_002285515.1
GCA_002285535.1
GCA_002285575.1
GCA_002285595.1

GCA_002285615.1
GCA_002285635.2
GCA_002285655.1
GCA_002285675.1
GCA_002285695.1
GCA_002285715.1
GCA_002285735.1
GCA_002285755.1
GCA_002285775.1
GCA_002285795.1
GCA_002285815.1
GCA_002285835.1
GCA_002285855.1
GCA_002285875.1
GCA_002285905.1
GCA_002285935.1
GCA_002285955.1
GCA_002285975.1
GCA_002285995.1
GCA_002286015.1
GCA_002286035.1
GCA_002286055.1
GCA_002286075.1
GCA_002286095.1
GCA_002286115.1
GCA_002286135.1
GCA_002286155.1
GCA_002286175.1
GCA_002286215.1
GCA_002286235.1
GCA_002286255.1
GCA_002286275.1
GCA_002286295.1
GCA_002286315.1
GCA_002286335.1
GCA_002286355.1
GCA_002286375.1
GCA_002287505.1
GCA_002287545.1
GCA_002287605.1
GCA_002287725.2
GCA_002287885.2
GCA_002287905.1
GCA_002287925.1
GCA_002287945.1
GCA_002287965.1
GCA_002288005.1
GCA_002288025.1
GCA_002288045.1
GCA_002288065.1
GCA_002288075.1
GCA_002288105.1
GCA_002288125.1
GCA_002288145.1

GCA_002288155.1
GCA_002288185.1
GCA_002288205.1
GCA_002288225.1
GCA_002288245.1
GCA_002288265.1
GCA_002288285.1
GCA_002288305.1
GCA_002288325.1
GCA_002288345.1
GCA_002288365.1
GCA_002288385.1
GCA_002288485.1
GCA_002288505.1
GCA_002288525.1
GCA_002288545.1
GCA_002288565.1
GCA_002288585.1
GCA_002289045.2
GCA_002289205.1
GCA_002290025.1
GCA_002290085.1
GCA_002290185.1
GCA_002290205.1
GCA_002290285.1
GCA_002290305.1
GCA_002290365.1
GCA_002291265.1
GCA_002291405.1
GCA_002291425.1
GCA_002291445.1
GCA_002300135.1
GCA_002300675.2
GCA_002302315.1
GCA_002302335.1
GCA_002302355.1
GCA_002302375.1
GCA_002302395.1
GCA_002302415.1
GCA_002302445.1
GCA_002302475.1
GCA_002302495.1
GCA_002302515.1
GCA_002302535.1
GCA_002302565.1
GCA_002302595.1
GCA_002302615.1
GCA_002302635.1
GCA_002302655.1
GCA_002303275.1
GCA_002304065.2
GCA_002305855.1
GCA_002305875.1
GCA_002305895.1

GCA_002307335.2
GCA_002307355.1
GCA_002309535.1
GCA_002309555.1
GCA_002310295.2
GCA_002310315.1
GCA_002310335.1
GCA_002310375.3
GCA_002310395.1
GCA_002310435.1
GCA_002310455.2
GCA_002310475.1
GCA_002310495.1
GCA_002310515.1
GCA_002310535.1
GCA_002310555.1
GCA_002310575.1
GCA_002310595.1
GCA_002310615.1
GCA_002310635.1
GCA_002310655.1
GCA_002310675.1
GCA_002310695.1
GCA_002310715.1
GCA_002310735.1
GCA_002310795.1
GCA_002310815.1
GCA_002310835.1
GCA_002310855.1
GCA_002310875.1
GCA_002310895.1
GCA_002310915.1
GCA_002312985.1
GCA_002313005.1
GCA_002313025.1
GCA_002313045.1
GCA_002313065.1
GCA_002313085.1
GCA_002313105.1
GCA_002313125.1
GCA_002327085.1
GCA_002327105.1
GCA_002327145.1
GCA_002327165.1
GCA_002327185.1
GCA_002327205.1
GCA_002334625.1
GCA_002334685.1
GCA_002335685.1
GCA_002345985.1
GCA_002346005.1
GCA_002346025.1
GCA_002346045.1
GCA_002346065.1

GCA_002346085.1
GCA_002346105.1
GCA_002346125.1
GCA_002346145.1
GCA_002346165.1
GCA_002346185.1
GCA_002346205.1
GCA_002354875.1
GCA_002354895.1
GCA_002355135.1
GCA_002355155.1
GCA_002355175.1
GCA_002355195.1
GCA_002355215.1
GCA_002355275.1
GCA_002355295.1
GCA_002355315.1
GCA_002355335.1
GCA_002355355.1
GCA_002355375.1
GCA_002355395.1
GCA_002355415.1
GCA_002355435.1
GCA_002355455.1
GCA_002355475.1
GCA_002355495.1
GCA_002355515.1
GCA_002355535.1
GCA_002355555.1
GCA_002355575.1
GCA_002355595.1
GCA_002355675.1
GCA_002355695.1
GCA_002355715.1
GCA_002355735.1
GCA_002355755.1
GCA_002355775.1
GCA_002355795.1
GCA_002355815.1
GCA_002355835.1
GCA_002355855.1
GCA_002355875.1
GCA_002355895.1
GCA_002355935.1
GCA_002355955.1
GCA_002355975.1
GCA_002355995.1
GCA_002356015.1
GCA_002356035.1
GCA_002356055.1
GCA_002356075.1
GCA_002356095.1
GCA_002356115.1
GCA_002356135.1

GCA_002356155.1
GCA_002356175.1
GCA_002356195.1
GCA_002356215.1
GCA_002356235.1
GCA_002356255.1
GCA_002356275.1
GCA_002356295.1
GCA_002356315.1
GCA_002356335.1
GCA_002356355.1
GCA_002356375.1
GCA_002356415.1
GCA_002356435.2
GCA_002356455.1
GCA_002356535.2
GCA_002356555.2
GCA_002356575.1
GCA_002356595.1
GCA_002356635.1
GCA_002356675.1
GCA_002356695.1
GCA_002357115.1
GCA_002357135.1
GCA_002357155.1
GCA_002357175.1
GCA_002357195.1
GCA_002357215.1
GCA_002357235.1
GCA_002357255.1
GCA_002357275.1
GCA_002357295.1
GCA_002357875.1
GCA_002357895.1
GCA_002357915.1
GCA_002357935.1
GCA_002357955.1
GCA_002357975.1
GCA_002357995.1
GCA_002368175.1
GCA_002368255.1
GCA_002368355.1
GCA_002368395.1
GCA_002368455.1
GCA_002369955.1
GCA_002370195.2
GCA_002370355.1
GCA_002370375.1
GCA_002370395.1
GCA_002370415.1
GCA_002370525.2
GCA_002374815.1
GCA_002374845.2
GCA_002379145.2

GCA_002381265.1
GCA_002381285.1
GCA_002381305.1
GCA_002381325.1
GCA_002381365.1
GCA_002381385.1
GCA_002386165.1
GCA_002386185.1
GCA_002386205.1
GCA_002386245.1
GCA_002386265.1
GCA_002386285.1
GCA_002386305.1
GCA_002386325.1
GCA_002386345.1
GCA_002386365.1
GCA_002386385.1
GCA_002393225.1
GCA_002393245.1
GCA_002393265.1
GCA_002393365.1
GCA_002393385.1
GCA_002393405.1
GCA_002393425.1
GCA_002393445.1
GCA_002393465.1
GCA_002393485.1
GCA_002393505.1
GCA_002393525.1
GCA_002407065.1
GCA_002407085.1
GCA_002407125.1
GCA_002407145.1
GCA_002407165.1
GCA_002407185.1
GCA_002407205.1
GCA_002407245.1
GCA_002407265.1
GCA_002407485.1
GCA_002407505.1
GCA_002407525.3
GCA_002411865.3
GCA_002412025.1
GCA_002412045.1
GCA_002441855.2
GCA_002441935.1
GCA_002441955.1
GCA_002441975.1
GCA_002442495.2
GCA_002442515.1
GCA_002442535.1
GCA_002442555.1
GCA_002442575.1
GCA_002442595.2

GCA_002442615.1
GCA_002442635.1
GCA_002442655.1
GCA_002442675.1
GCA_002442695.1
GCA_002442715.1
GCA_002442735.1
GCA_002442755.1
GCA_002442775.1
GCA_002442795.1
GCA_002442815.1
GCA_002442835.1
GCA_002442855.1
GCA_002442875.1
GCA_002442895.1
GCA_002442915.1
GCA_002442935.1
GCA_002442955.1
GCA_002442975.1
GCA_002442995.2
GCA_002443015.2
GCA_002443035.1
GCA_002443095.1
GCA_002443115.1
GCA_002443155.1
GCA_002443175.1
GCA_002446875.1
GCA_002446895.1
GCA_002446915.1
GCA_002446935.1
GCA_002446955.1
GCA_002446975.1
GCA_002446995.1
GCA_002447015.1
GCA_002447035.1
GCA_002447055.1
GCA_002447075.1
GCA_002447095.1
GCA_002447115.1
GCA_002447135.1
GCA_002447155.1
GCA_002447175.1
GCA_002447195.1
GCA_002447215.1
GCA_002447235.1
GCA_002447255.1
GCA_002447275.1
GCA_002447295.1
GCA_002447315.1
GCA_002447335.1
GCA_002447355.1
GCA_002447375.1
GCA_002447395.1
GCA_002447415.1

GCA_002447435.1
GCA_002447455.1
GCA_002447475.1
GCA_002447495.1
GCA_002447515.1
GCA_002447535.1
GCA_002447555.1
GCA_002447575.1
GCA_002447595.1
GCA_002447615.1
GCA_002447635.1
GCA_002447655.1
GCA_002447675.1
GCA_002447695.1
GCA_002447715.1
GCA_002447735.1
GCA_002447755.1
GCA_002447775.1
GCA_002447795.1
GCA_002447815.1
GCA_002447835.1
GCA_002447855.1
GCA_002447875.1
GCA_002447895.1
GCA_002447915.1
GCA_002447935.1
GCA_002447955.1
GCA_002447975.1
GCA_002447995.1
GCA_002448015.1
GCA_002448035.1
GCA_002448055.1
GCA_002448075.1
GCA_002448095.1
GCA_002448115.1
GCA_002448135.1
GCA_002448155.1
GCA_002448175.1
GCA_002448195.1
GCA_002448215.1
GCA_002456135.1
GCA_002494365.2
GCA_002499975.2
GCA_002501565.1
GCA_002501585.1
GCA_002504085.1
GCA_002504105.1
GCA_002504125.1
GCA_002504145.1
GCA_002504165.1
GCA_002504265.1
GCA_002504285.1
GCA_002504325.1
GCA_002504365.1

GCA_002504385.1
GCA_002507715.2
GCA_002507745.2
GCA_002507865.2
GCA_002507875.2
GCA_002507915.2
GCA_002531755.2
GCA_002549795.1
GCA_002549815.1
GCA_002549835.1
GCA_002556525.1
GCA_002556545.1
GCA_002556565.1
GCA_002556585.1
GCA_002557735.1
GCA_002557755.1
GCA_002557775.1
GCA_002557795.1
GCA_002557815.1
GCA_002568625.1
GCA_002576835.1
GCA_002586745.1
GCA_002586945.1
GCA_002587005.1
GCA_002587065.1
GCA_002587105.1
GCA_002587165.1
GCA_002587225.1
GCA_002587885.1
GCA_002588845.1
GCA_002589795.1
GCA_002591075.1
GCA_002591095.1
GCA_002591115.1
GCA_002591135.1
GCA_002591155.1
GCA_002591175.1
GCA_002591195.1
GCA_002591215.1
GCA_002591235.1
GCA_002591255.1
GCA_002591275.1
GCA_002591295.1
GCA_002591335.1
GCA_002591665.3
GCA_002600635.1
GCA_002633765.1
GCA_002633785.1
GCA_002633805.1
GCA_002633825.1
GCA_002633845.1
GCA_002633865.1
GCA_002688505.1
GCA_002688525.1

GCA_002688565.1
GCA_002688585.1
GCA_002688605.1
GCA_002688645.1
GCA_002688665.1
GCA_002688685.1
GCA_002688705.1
GCA_002706325.1
GCA_002706375.1
GCA_002706425.1
GCA_002706485.1
GCA_002706565.1
GCA_002706665.1
GCA_002706705.1
GCA_002706745.1
GCA_002706795.1
GCA_002715865.1
GCA_002716885.1
GCA_002716905.4
GCA_002716925.3
GCA_002716965.4
GCA_002717025.1
GCA_002734145.1
GCA_002735225.1
GCA_002735245.1
GCA_002735385.2
GCA_002735945.1
GCA_002736045.1
GCA_002736065.1
GCA_002736105.1
GCA_002736125.1
GCA_002736145.1
GCA_002736205.1
GCA_002736265.1
GCA_002736285.1
GCA_002741015.1
GCA_002741035.1
GCA_002741055.1
GCA_002741075.1
GCA_002741175.1
GCA_002741195.1
GCA_002741215.1
GCA_002741255.1
GCA_002741275.1
GCA_002741295.1
GCA_002741315.1
GCA_002741335.1
GCA_002741355.1
GCA_002741375.1
GCA_002741415.1
GCA_002741435.1
GCA_002741455.1
GCA_002741475.1
GCA_002741495.1

GCA_002741515.1
GCA_002741535.1
GCA_002741555.1
GCA_002741575.1
GCA_002741595.1
GCA_002741615.1
GCA_002741635.1
GCA_002741665.3
GCA_002741685.1
GCA_002741705.1
GCA_002741725.1
GCA_002741745.1
GCA_002741765.2
GCA_002741785.3
GCA_002741805.3
GCA_002741825.3
GCA_002741845.4
GCA_002741865.1
GCA_002741885.1
GCA_002741905.1
GCA_002742645.2
GCA_002749475.1
GCA_002749495.1
GCA_002749575.1
GCA_002749615.1
GCA_002749635.1
GCA_002749655.1
GCA_002749675.1
GCA_002752655.1
GCA_002752675.1
GCA_002752695.1
GCA_002752715.1
GCA_002752735.1
GCA_002752755.2
GCA_002752775.1
GCA_002752905.1
GCA_002752955.1
GCA_002752975.1
GCA_002752995.1
GCA_002753055.1
GCA_002753075.1
GCA_002753165.1
GCA_002753355.1
GCA_002753375.2
GCA_002753405.1
GCA_002753555.1
GCA_002753605.1
GCA_002753655.1
GCA_002753715.2
GCA_002753915.1
GCA_002753935.1
GCA_002753955.1
GCA_002753975.1
GCA_002753995.1

GCA_002754015.1
GCA_002754035.1
GCA_002754055.1
GCA_002754075.1
GCA_002754095.1
GCA_002754115.1
GCA_002754135.1
GCA_002754155.1
GCA_002754815.1
GCA_002754935.1
GCA_002759095.2
GCA_002759115.2
GCA_002759135.3
GCA_002759155.2
GCA_002759175.2
GCA_002759195.2
GCA_002759215.3
GCA_002759235.2
GCA_002759255.3
GCA_002759275.2
GCA_002759295.2
GCA_002759315.2
GCA_002759335.2
GCA_002759355.2
GCA_002759375.2
GCA_002759395.2
GCA_002759415.2
GCA_002760915.1
GCA_002760935.1
GCA_002760955.1
GCA_002760975.1
GCA_002760995.1
GCA_002761015.1
GCA_002761035.1
GCA_002761055.1
GCA_002761075.1
GCA_002761095.1
GCA_002761115.1
GCA_002761135.1
GCA_002761155.1
GCA_002761215.1
GCA_002761235.1
GCA_002761255.1
GCA_002761275.1
GCA_002761315.1
GCA_002761335.1
GCA_002761355.1
GCA_002761375.1
GCA_002761395.1
GCA_002761415.1
GCA_002761435.1
GCA_002761455.1
GCA_002761475.1
GCA_002761495.1

GCA_002761515.1
GCA_002761535.1
GCA_002761555.1
GCA_002761575.1
GCA_002761835.1
GCA_002761875.1
GCA_002762095.1
GCA_002762115.1
GCA_002762135.3
GCA_002762155.1
GCA_002762175.1
GCA_002762195.1
GCA_002762215.1
GCA_002762335.1
GCA_002762545.2
GCA_002763415.1
GCA_002763515.3
GCA_002763535.1
GCA_002763555.1
GCA_002763575.1
GCA_002763595.1
GCA_002763615.1
GCA_002763625.1
GCA_002763655.1
GCA_002763675.1
GCA_002763695.1
GCA_002763715.1
GCA_002763735.1
GCA_002763745.1
GCA_002763775.1
GCA_002763785.1
GCA_002763815.1
GCA_002763845.1
GCA_002763875.1
GCA_002763915.1
GCA_002763925.1
GCA_002763955.1
GCA_002763975.1
GCA_002763995.1
GCA_002764015.1
GCA_002764035.1
GCA_002764055.1
GCA_002764075.1
GCA_002764115.1
GCA_002764135.1
GCA_002770595.1
GCA_002776555.1
GCA_002776575.1
GCA_002776595.1
GCA_002776615.1
GCA_002776635.1
GCA_002776655.1
GCA_002776675.1
GCA_002776715.1

GCA_002776735.1
GCA_002776745.1
GCA_002776775.1
GCA_002776795.1
GCA_002776835.1
GCA_002776845.1
GCA_002776885.1
GCA_002776955.1
GCA_002776975.1
GCA_002776995.1
GCA_002777015.1
GCA_002777035.1
GCA_002777055.1
GCA_002777075.1
GCA_002777095.1
GCA_002777135.1
GCA_002777155.1
GCA_002777175.1
GCA_002777255.1
GCA_002777275.1
GCA_002777395.1
GCA_002777455.1
GCA_002777535.1
GCA_002786455.2
GCA_002786465.1
GCA_002786535.1
GCA_002786655.1
GCA_002786755.1
GCA_002786865.1
GCA_002787125.1
GCA_002787215.1
GCA_002787315.1
GCA_002787395.1
GCA_002787545.1
GCA_002787645.1
GCA_002787755.1
GCA_002787935.1
GCA_002788115.1
GCA_002788295.1
GCA_002794415.1
GCA_002794435.1
GCA_002795265.1
GCA_002795285.1
GCA_002795805.1
GCA_002795825.1
GCA_002795845.1
GCA_002795865.1
GCA_002795885.1
GCA_002796405.1
GCA_002796425.1
GCA_002796445.1
GCA_002796465.1
GCA_002796505.1
GCA_002796525.1

GCA_002796545.1
GCA_002796585.1
GCA_002796605.1
GCA_002803025.2
GCA_002803805.2
GCA_002803825.1
GCA_002803845.1
GCA_002803865.1
GCA_002803885.1
GCA_002803905.1
GCA_002803925.1
GCA_002803945.1
GCA_002803965.1
GCA_002803985.1
GCA_002804005.1
GCA_002804025.1
GCA_002804065.1
GCA_002804085.1
GCA_002804105.1
GCA_002804125.1
GCA_002804165.1
GCA_002804185.1
GCA_002804205.1
GCA_002804225.1
GCA_002804245.1
GCA_002804265.1
GCA_002804285.1
GCA_002804305.1
GCA_002808045.1
GCA_002810325.1
GCA_002811305.2
GCA_002811325.3
GCA_002811335.4
GCA_002811425.2
GCA_002811555.3
GCA_002812345.2
GCA_002812365.1
GCA_002812385.1
GCA_002812405.1
GCA_002812425.1
GCA_002812445.1
GCA_002812465.1
GCA_002812485.1
GCA_002812505.1
GCA_002812525.1
GCA_002812545.1
GCA_002812565.2
GCA_002812585.1
GCA_002812605.1
GCA_002812625.1
GCA_002812645.1
GCA_002812665.1
GCA_002812705.1
GCA_002812825.1

GCA_002812845.1
GCA_002812865.1
GCA_002812885.1
GCA_002812905.2
GCA_002812925.3
GCA_002813045.1
GCA_002813065.1
GCA_002813535.1
GCA_002813555.1
GCA_002813575.1
GCA_002813595.1
GCA_002813615.1
GCA_002813715.1
GCA_002813735.1
GCA_002813755.1
GCA_002813775.1
GCA_002813875.1
GCA_002813895.1
GCA_002813935.1
GCA_002813955.1
GCA_002813975.1
GCA_002813995.1
GCA_002814015.1
GCA_002814035.1
GCA_002814115.1
GCA_002814135.1
GCA_002814155.1
GCA_002831485.1
GCA_002831505.1
GCA_002831545.1
GCA_002831565.1
GCA_002831585.1
GCA_002831605.1
GCA_002831625.1
GCA_002831645.1
GCA_002831665.1
GCA_002831685.1
GCA_002832675.1
GCA_002833405.1
GCA_002833425.1
GCA_002833445.1
GCA_002833465.1
GCA_002833485.1
GCA_002838165.1
GCA_002838185.1
GCA_002838205.1
GCA_002838225.1
GCA_002838245.1
GCA_002838265.1
GCA_002838285.1
GCA_002838305.1
GCA_002838325.1
GCA_002838345.1
GCA_002838365.1

GCA_002838385.1
GCA_002838405.1
GCA_002838425.1
GCA_002838445.1
GCA_002838465.1
GCA_002838485.1
GCA_002838505.1
GCA_002838525.1
GCA_002838545.1
GCA_002838565.1
GCA_002838585.1
GCA_002838605.1
GCA_002838625.1
GCA_002838645.1
GCA_002838665.1
GCA_002838685.1
GCA_002838705.1
GCA_002838725.1
GCA_002838745.1
GCA_002838765.1
GCA_002843115.1
GCA_002843135.1
GCA_002843235.3
GCA_002843285.1
GCA_002843505.1
GCA_002843525.1
GCA_002843545.1
GCA_002843565.1
GCA_002843645.1
GCA_002843665.1
GCA_002843685.1
GCA_002843965.1
GCA_002843985.1
GCA_002844045.1
GCA_002844065.1
GCA_002844085.1
GCA_002844105.1
GCA_002844125.1
GCA_002844145.1
GCA_002844165.1
GCA_002844355.1
GCA_002844375.1
GCA_002844395.1
GCA_002844685.1
GCA_002844715.2
GCA_002844755.2
GCA_002845275.2
GCA_002845365.1
GCA_002845845.1
GCA_002846075.1
GCA_002846095.1
GCA_002846115.1
GCA_002846135.1
GCA_002846155.1

GCA_002846175.1
GCA_002846975.1
GCA_002846995.1
GCA_002847015.1
GCA_002847285.1
GCA_002847305.1
GCA_002847385.1
GCA_002847405.1
GCA_002847425.1
GCA_002847445.1
GCA_002847845.2
GCA_002848225.1
GCA_002848345.1
GCA_002848365.2
GCA_002848385.1
GCA_002848425.1
GCA_002848445.1
GCA_002848465.1
GCA_002848485.1
GCA_002848505.1
GCA_002848525.1
GCA_002848545.1
GCA_002848565.1
GCA_002848585.1
GCA_002848605.1
GCA_002848625.1
GCA_002848645.1
GCA_002848665.1
GCA_002848685.1
GCA_002848705.1
GCA_002848725.1
GCA_002848745.1
GCA_002849695.1
GCA_002849715.1
GCA_002849735.1
GCA_002849755.2
GCA_002849775.1
GCA_002849795.1
GCA_002849835.1
GCA_002849875.1
GCA_002849895.1
GCA_002849915.1
GCA_002849935.1
GCA_002849955.1
GCA_002849975.1
GCA_002849995.1
GCA_002850015.1
GCA_002850035.1
GCA_002850055.1
GCA_002850075.1
GCA_002850095.1
GCA_002850115.1
GCA_002850135.1
GCA_002850155.1

GCA_002850175.1
GCA_002850195.1
GCA_002850215.1
GCA_002850235.1
GCA_002850255.1
GCA_002850275.3
GCA_002850295.1
GCA_002850315.1
GCA_002850375.1
GCA_002850395.1
GCA_002850415.1
GCA_002850455.1
GCA_002850475.1
GCA_002850495.1
GCA_002850515.1
GCA_002850535.1
GCA_002850555.1
GCA_002850575.1
GCA_002850675.5
GCA_002850695.3
GCA_002852325.2
GCA_002852365.2
GCA_002852675.2
GCA_002852785.2
GCA_002852915.4
GCA_002852945.3
GCA_002852975.3
GCA_002852995.3
GCA_002853115.2
GCA_002853385.2
GCA_002853435.3
GCA_002853475.2
GCA_002853655.1
GCA_002853715.1
GCA_002853805.1
GCA_002853965.1
GCA_002854065.1
GCA_002855325.1
GCA_002855415.1
GCA_002859105.1
GCA_002859165.1
GCA_002859205.1
GCA_002859225.1
GCA_002859245.1
GCA_002859265.1
GCA_002859285.1
GCA_002859305.1
GCA_002859325.1
GCA_002859345.1
GCA_002859365.1
GCA_002859385.1
GCA_002859405.1
GCA_002859425.1
GCA_002859445.1

GCA_002859465.1
GCA_002859485.1
GCA_002859505.1
GCA_002859525.1
GCA_002859545.1
GCA_002859565.1
GCA_002859585.1
GCA_002859605.1
GCA_002859625.1
GCA_002859645.1
GCA_002859665.1
GCA_002859685.1
GCA_002859705.1
GCA_002859725.1
GCA_002859745.1
GCA_002859765.1
GCA_002859785.1
GCA_002859805.1
GCA_002859825.1
GCA_002859845.1
GCA_002859865.1
GCA_002859885.1
GCA_002859905.1
GCA_002859945.1
GCA_002859965.1
GCA_002859985.1
GCA_002860005.1
GCA_002860025.1
GCA_002860045.1
GCA_002860065.1
GCA_002860085.1
GCA_002860105.1
GCA_002860645.2
GCA_002863665.1
GCA_002863685.1
GCA_002863725.1
GCA_002863745.1
GCA_002863765.1
GCA_002863785.1
GCA_002863805.1
GCA_002863825.3
GCA_002863845.1
GCA_002863865.1
GCA_002863885.1
GCA_002863905.1
GCA_002865505.1
GCA_002865525.1
GCA_002865545.1
GCA_002865565.1
GCA_002865585.1
GCA_002865605.1
GCA_002865745.1
GCA_002865765.1
GCA_002865785.1

GCA_002865805.1
GCA_002865825.1
GCA_002865845.1
GCA_002865885.1
GCA_002865995.1
GCA_002866045.1
GCA_002866125.1
GCA_002866225.1
GCA_002866885.2
GCA_002868735.1
GCA_002868755.1
GCA_002868775.1
GCA_002869785.1
GCA_002869805.1
GCA_002869825.2
GCA_002870165.1
GCA_002871575.2
GCA_002871855.2
GCA_002871915.2
GCA_002871945.2
GCA_002871995.2
GCA_002872015.2
GCA_002872415.1
GCA_002872435.1
GCA_002872455.1
GCA_002872475.1
GCA_002874965.1
GCA_002879955.1
GCA_002879975.1
GCA_002879995.1
GCA_002880615.1
GCA_002880635.1
GCA_002881155.1
GCA_002881175.1
GCA_002881195.1
GCA_002881215.1
GCA_002881235.1
GCA_002881255.1
GCA_002881275.1
GCA_002881295.1
GCA_002881315.1
GCA_002881335.1
GCA_002881355.1
GCA_002881375.1
GCA_002881395.1
GCA_002881415.1
GCA_002881435.1
GCA_002881455.1
GCA_002881475.1
GCA_002881495.1
GCA_002881515.1
GCA_002881535.1
GCA_002881555.1
GCA_002881575.1

GCA_002881595.1
GCA_002881615.1
GCA_002881695.1
GCA_002881775.1
GCA_002881865.1
GCA_002881935.1
GCA_002882035.1
GCA_002882125.1
GCA_002882205.1
GCA_002882275.1
GCA_002882375.1
GCA_002882465.1
GCA_002882555.1
GCA_002882645.1
GCA_002882745.1
GCA_002882835.1
GCA_002886045.1
GCA_002886065.1
GCA_002886145.1
GCA_002886165.1
GCA_002886195.1
GCA_002886225.1
GCA_002886335.1
GCA_002886405.1
GCA_002886505.1
GCA_002886585.1
GCA_002886685.1
GCA_002886775.1
GCA_002886865.1
GCA_002886945.1
GCA_002887065.1
GCA_002887145.1
GCA_002887255.1
GCA_002887275.1
GCA_002887335.1
GCA_002887555.1
GCA_002887615.1
GCA_002887655.1
GCA_002887715.1
GCA_002888685.1
GCA_002888715.1
GCA_002891475.1
GCA_002891665.1
GCA_002891825.2
GCA_002891845.2
GCA_002891905.1
GCA_002891925.1
GCA_002891945.1
GCA_002891965.1
GCA_002891985.1
GCA_002892005.1
GCA_002892025.1
GCA_002892045.1
GCA_002892065.1

GCA_002892085.1
GCA_002892125.1
GCA_002892145.1
GCA_002892165.1
GCA_002892185.1
GCA_002892205.1
GCA_002892225.1
GCA_002892245.1
GCA_002892265.1
GCA_002892535.1
GCA_002892555.1
GCA_002892575.1
GCA_002892595.1
GCA_002892665.1
GCA_002892685.1
GCA_002892705.1
GCA_002892725.1
GCA_002892745.1
GCA_002892765.1
GCA_002892855.1
GCA_002893765.1
GCA_002893785.1
GCA_002893805.1
GCA_002893825.1
GCA_002893845.1
GCA_002893885.1
GCA_002893905.2
GCA_002893965.1
GCA_002894285.1
GCA_002894385.2
GCA_002895085.1
GCA_002895105.1
GCA_002895125.1
GCA_002895145.1
GCA_002895185.2
GCA_002895205.1
GCA_002895225.1
GCA_002895245.1
GCA_002895385.1
GCA_002895725.2
GCA_002896375.1
GCA_002896855.1
GCA_002896875.1
GCA_002899475.1
GCA_002899495.1
GCA_002899515.1
GCA_002899535.1
GCA_002900305.1
GCA_002900325.1
GCA_002900365.1
GCA_002900385.1
GCA_002902805.2
GCA_002902825.1
GCA_002902865.1

GCA_002902885.1
GCA_002902905.1
GCA_002902925.1
GCA_002902945.1
GCA_002902965.1
GCA_002903005.1
GCA_002903025.1
GCA_002903045.1
GCA_002903085.1
GCA_002903125.1
GCA_002903215.1
GCA_002903405.1
GCA_002903485.1
GCA_002905685.2
GCA_002905795.2
GCA_002905815.2
GCA_002906195.1
GCA_002906395.1
GCA_002906435.1
GCA_002906455.1
GCA_002906475.1
GCA_002906495.1
GCA_002906515.1
GCA_002906535.1
GCA_002906555.1
GCA_002906595.1
GCA_002906615.1
GCA_002906655.1
GCA_002906875.1
GCA_002909775.2
GCA_002910725.2
GCA_002910775.2
GCA_002918585.2
GCA_002920045.2
GCA_002921235.2
GCA_002925525.1
GCA_002934025.1
GCA_002934065.1
GCA_002934285.1
GCA_002934345.1
GCA_002934425.1
GCA_002934585.1
GCA_002934695.1
GCA_002934795.1
GCA_002934895.1
GCA_002934965.1
GCA_002935055.1
GCA_002935085.1
GCA_002943525.1
GCA_002943545.1
GCA_002943835.1
GCA_002943855.1
GCA_002943895.1
GCA_002943995.1

GCA_002944315.1
GCA_002944405.1
GCA_002944495.1
GCA_002944585.1
GCA_002944765.1
GCA_002944845.1
GCA_002944935.1
GCA_002944995.1
GCA_002945135.3
GCA_002945235.1
GCA_002945415.1
GCA_002945515.1
GCA_002945665.1
GCA_002945755.1
GCA_002945855.1
GCA_002945945.1
GCA_002946035.1
GCA_002946135.1
GCA_002946195.1
GCA_002946375.1
GCA_002946455.1
GCA_002946515.2
GCA_002946535.2
GCA_002946555.2
GCA_002946595.1
GCA_002946655.1
GCA_002946675.1
GCA_002946695.1
GCA_002946715.1
GCA_002946735.1
GCA_002946835.1
GCA_002946935.1
GCA_002947035.1
GCA_002947135.1
GCA_002947235.1
GCA_002947315.1
GCA_002947415.1
GCA_002947505.1
GCA_002947575.1
GCA_002947675.1
GCA_002947755.1
GCA_002947845.1
GCA_002947915.1
GCA_002947975.1
GCA_002948105.1
GCA_002948215.1
GCA_002948295.1
GCA_002948565.1
GCA_002948655.1
GCA_002948835.2
GCA_002948925.1
GCA_002948995.1
GCA_002949085.1
GCA_002949175.1

GCA_002949255.1
GCA_002949345.1
GCA_002949415.1
GCA_002949455.1
GCA_002949475.1
GCA_002949495.1
GCA_002949515.1
GCA_002949535.1
GCA_002949555.1
GCA_002949575.1
GCA_002949615.1
GCA_002949635.1
GCA_002949655.1
GCA_002949675.1
GCA_002949695.1
GCA_002949715.1
GCA_002949735.1
GCA_002949755.1
GCA_002949775.1
GCA_002949795.1
GCA_002949815.1
GCA_002949835.1
GCA_002949855.1
GCA_002949875.1
GCA_002949915.1
GCA_002949935.1
GCA_002949975.1
GCA_002949995.1
GCA_002950015.1
GCA_002950035.1
GCA_002950055.1
GCA_002950075.1
GCA_002950095.1
GCA_002950115.1
GCA_002950135.1
GCA_002950155.1
GCA_002950175.1
GCA_002950215.1
GCA_002950235.1
GCA_002950255.1
GCA_002950275.1
GCA_002950315.1
GCA_002950335.1
GCA_002950375.1
GCA_002950395.1
GCA_002950495.2
GCA_002950575.1
GCA_002950695.1
GCA_002950775.1
GCA_002950865.1
GCA_002950945.1
GCA_002951025.1
GCA_002951115.1
GCA_002951195.1

GCA_002951295.1
GCA_002951395.1
GCA_002951455.1
GCA_002951475.1
GCA_002951505.1
GCA_002951555.1
GCA_002951575.1
GCA_002951595.1
GCA_002951615.1
GCA_002951635.1
GCA_002951655.1
GCA_002951675.1
GCA_002951695.1
GCA_002951715.1
GCA_002951735.1
GCA_002951755.1
GCA_002951775.1
GCA_002951795.1
GCA_002951815.1
GCA_002951835.1
GCA_002951855.1
GCA_002951875.1
GCA_002951895.1
GCA_002951915.1
GCA_002951935.1
GCA_002951955.2
GCA_002951975.1
GCA_002951995.1
GCA_002952015.1
GCA_002952035.2
GCA_002952055.1
GCA_002952075.1
GCA_002952095.1
GCA_002952135.1
GCA_002952215.1
GCA_002952295.1
GCA_002952315.1
GCA_002952335.1
GCA_002952355.1
GCA_002952375.1
GCA_002952455.1
GCA_002952475.1
GCA_002952495.1
GCA_002952515.1
GCA_002952535.1
GCA_002952555.1
GCA_002952575.1
GCA_002952595.1
GCA_002952615.1
GCA_002952635.1
GCA_002952655.1
GCA_002952675.1
GCA_002952695.1
GCA_002952715.2

GCA_002952735.2
GCA_002952755.1
GCA_002952835.1
GCA_002952855.1
GCA_002952875.1
GCA_002952895.1
GCA_002952915.1
GCA_002952935.1
GCA_002952955.1
GCA_002952975.1
GCA_002952995.1
GCA_002953015.1
GCA_002953035.1
GCA_002953055.1
GCA_002953075.1
GCA_002953095.1
GCA_002953115.1
GCA_002953155.1
GCA_002953175.1
GCA_002953195.1
GCA_002953215.1
GCA_002953235.1
GCA_002953275.1
GCA_002953295.1
GCA_002953315.1
GCA_002953335.1
GCA_002953355.1
GCA_002953375.1
GCA_002953395.1
GCA_002953415.1
GCA_002953435.1
GCA_002953455.1
GCA_002953515.1
GCA_002953535.1
GCA_002953555.1
GCA_002953575.2
GCA_002953595.1
GCA_002953655.1
GCA_002953715.1
GCA_002953735.1
GCA_002953755.1
GCA_002953775.1
GCA_002953795.1
GCA_002953815.1
GCA_002953835.1
GCA_002953855.1
GCA_002953875.1
GCA_002953895.1
GCA_002953935.1
GCA_002953955.1
GCA_002953975.1
GCA_002954055.1
GCA_002954165.1
GCA_002954185.1

GCA_002954205.1
GCA_002960215.1
GCA_002966125.1
GCA_002966495.1
GCA_002966515.1
GCA_002966535.1
GCA_002966555.1
GCA_002966575.1
GCA_002966595.1
GCA_002966615.1
GCA_002966635.1
GCA_002966655.1
GCA_002966675.1
GCA_002966695.1
GCA_002966715.1
GCA_002966735.1
GCA_002966755.1
GCA_002966775.1
GCA_002966795.1
GCA_002966815.1
GCA_002966835.1
GCA_002966855.1
GCA_002966875.1
GCA_002966955.1
GCA_002967795.2
GCA_002967855.2
GCA_002967875.2
GCA_002967885.2
GCA_002967935.2
GCA_002967955.2
GCA_002967975.2
GCA_002968395.1
GCA_002968415.1
GCA_002968455.1
GCA_002968475.1
GCA_002968495.1
GCA_002968515.1
GCA_002968585.1
GCA_002968655.1
GCA_002968695.1
GCA_002968715.1
GCA_002968735.1
GCA_002968755.1
GCA_002968775.1
GCA_002968935.1
GCA_002968955.1
GCA_002969195.1
GCA_002970375.3
GCA_002970895.1
GCA_002970915.1
GCA_002970935.1
GCA_002973635.1
GCA_002973655.1
GCA_002973755.2

GCA_002975475.1
GCA_002980175.2
GCA_002980615.3
GCA_002982095.1
GCA_002982115.1
GCA_002982135.1
GCA_002982155.1
GCA_002982175.1
GCA_002982195.1
GCA_002983625.1
GCA_002983645.1
GCA_002983665.1
GCA_002983685.1
GCA_002983705.1
GCA_002983725.1
GCA_002983785.1
GCA_002983865.1
GCA_002983965.1
GCA_002984035.1
GCA_002984125.1
GCA_002984195.1
GCA_002984285.1
GCA_002984395.1
GCA_002984485.1
GCA_002984565.1
GCA_002988215.2
GCA_002988885.2
GCA_002993105.1
GCA_002993265.1
GCA_002993285.1
GCA_002993305.1
GCA_002993925.1
GCA_002993945.1
GCA_002995515.1
GCA_002995535.1
GCA_002995555.1
GCA_002996665.1
GCA_002996805.1
GCA_002996885.1
GCA_002996945.1
GCA_002997005.1
GCA_002997125.1
GCA_002997215.1
GCA_002997735.1
GCA_002997815.1
GCA_002997915.1
GCA_002997935.1
GCA_002997955.1
GCA_002997975.1
GCA_002997995.1
GCA_002998015.1
GCA_002998035.1
GCA_002998115.1
GCA_002998175.1

GCA_002998235.1
GCA_002998295.1
GCA_002998355.1
GCA_002998435.1
GCA_002998535.1
GCA_002998595.1
GCA_002998695.1
GCA_002998835.1
GCA_002998925.1
GCA_002999035.1
GCA_002999075.1
GCA_002999115.2
GCA_002999135.1
GCA_002999155.1
GCA_002999195.2
GCA_002999215.3
GCA_002999235.1
GCA_002999255.1
GCA_002999275.1
GCA_002999295.1
GCA_002999415.1
GCA_002999435.1
GCA_003003495.1
GCA_003003515.1
GCA_003003535.1
GCA_003003555.1
GCA_003003595.1
GCA_003003675.1
GCA_003003755.1
GCA_003003815.1
GCA_003003865.1
GCA_003003955.1
GCA_003004065.1
GCA_003004115.1
GCA_003004135.1
GCA_003004155.1
GCA_003004175.1
GCA_003004195.1
GCA_003004215.1
GCA_003004235.1
GCA_003004255.1
GCA_003004275.1
GCA_003004295.1
GCA_003004315.1
GCA_003004335.1
GCA_003006035.1
GCA_003006055.1
GCA_003006075.1
GCA_003006115.1
GCA_003006135.1
GCA_003006155.1
GCA_003006175.1
GCA_003006415.1
GCA_003006435.1

GCA_003008415.2
GCA_003008475.2
GCA_003008495.1
GCA_003008515.1
GCA_003008535.1
GCA_003008555.2
GCA_003008575.1
GCA_003008595.1
GCA_003008615.1
GCA_003008635.1
GCA_003010455.1
GCA_003010475.1
GCA_003010495.1
GCA_003010655.1
GCA_003010675.1
GCA_003010695.1
GCA_003010715.1
GCA_003010735.1
GCA_003010875.1
GCA_003010915.2
GCA_003012895.3
GCA_003012915.1
GCA_003013205.1
GCA_003013295.1
GCA_003013315.1
GCA_003013635.1
GCA_003013675.1
GCA_003013695.1
GCA_003014775.1
GCA_003015125.1
GCA_003015145.1
GCA_003015165.1
GCA_003015185.1
GCA_003015225.1
GCA_003015715.1
GCA_003017145.1
GCA_003017195.1
GCA_003017225.1
GCA_003017335.1
GCA_003017375.1
GCA_003017455.1
GCA_003017555.1
GCA_003017615.1
GCA_003017675.2
GCA_003017765.1
GCA_003017805.1
GCA_003017915.1
GCA_003017935.1
GCA_003017955.1
GCA_003017975.1
GCA_003017995.1
GCA_003018015.1
GCA_003018035.1
GCA_003018055.1

GCA_003018075.1
GCA_003018095.1
GCA_003018115.1
GCA_003018135.1
GCA_003018155.1
GCA_003018195.1
GCA_003018215.1
GCA_003018235.1
GCA_003018255.1
GCA_003018275.1
GCA_003018315.1
GCA_003018335.1
GCA_003018355.1
GCA_003018375.1
GCA_003018395.1
GCA_003018435.1
GCA_003018455.1
GCA_003018475.1
GCA_003018495.1
GCA_003018535.1
GCA_003018555.1
GCA_003018575.1
GCA_003018595.1
GCA_003018615.1
GCA_003018635.1
GCA_003018655.1
GCA_003018695.1
GCA_003018715.1
GCA_003018755.1
GCA_003018775.1
GCA_003018795.1
GCA_003018815.1
GCA_003018835.2
GCA_003018855.1
GCA_003018875.1
GCA_003018895.1
GCA_003018915.1
GCA_003018935.1
GCA_003018955.1
GCA_003018995.1
GCA_003019015.1
GCA_003019035.1
GCA_003019055.1
GCA_003019075.1
GCA_003019095.1
GCA_003019115.1
GCA_003019155.1
GCA_003019175.1
GCA_003019195.1
GCA_003019215.1
GCA_003019235.1
GCA_003019255.1
GCA_003019275.1
GCA_003019295.1

GCA_003019315.1
GCA_003019655.1
GCA_003019675.1
GCA_003019695.1
GCA_003019715.1
GCA_003019735.1
GCA_003019755.1
GCA_003019785.1
GCA_003019815.1
GCA_003019855.1
GCA_003019925.1
GCA_003019945.1
GCA_003019965.1
GCA_003019985.1
GCA_003020005.1
GCA_003020025.1
GCA_003020045.1
GCA_003020185.1
GCA_003020685.1
GCA_003020705.1
GCA_003020725.1
GCA_003020745.1
GCA_003020765.1
GCA_003020825.1
GCA_003020845.1
GCA_003020885.1
GCA_003024155.1
GCA_003024525.3
GCA_003025345.2
GCA_003025365.1
GCA_003028335.1
GCA_003028855.1
GCA_003029495.1
GCA_003029645.1
GCA_003030025.1
GCA_003030065.1
GCA_003030085.1
GCA_003030105.1
GCA_003030125.1
GCA_003030145.1
GCA_003030165.1
GCA_003030185.1
GCA_003030205.1
GCA_003030225.1
GCA_003030245.1
GCA_003030285.2
GCA_003030305.1
GCA_003030345.1
GCA_003030365.1
GCA_003030385.1
GCA_003030405.1
GCA_003030425.1
GCA_003030465.1
GCA_003030905.1

GCA_003030925.1
GCA_003030985.1
GCA_003031205.1
GCA_003031225.1
GCA_003031245.1
GCA_003031265.1
GCA_003031285.1
GCA_003031305.1
GCA_003031325.1
GCA_003031345.1
GCA_003031365.1
GCA_003031385.1
GCA_003031405.1
GCA_003031425.1
GCA_003031445.1
GCA_003031485.1
GCA_003031505.1
GCA_003031545.1
GCA_003031645.1
GCA_003031735.1
GCA_003031755.1
GCA_003031775.1
GCA_003031795.1
GCA_003031815.1
GCA_003031835.1
GCA_003031855.1
GCA_003031875.1
GCA_003031895.1
GCA_003031915.1
GCA_003031935.1
GCA_003031955.1
GCA_003031975.1
GCA_003031995.1
GCA_003032015.1
GCA_003032035.1
GCA_003032055.1
GCA_003032075.1
GCA_003032455.1
GCA_003032475.1
GCA_003032495.1
GCA_003033905.1
GCA_003034205.2
GCA_003036645.2
GCA_003037005.2
GCA_003037185.2
GCA_003037325.3
GCA_003037615.2
GCA_003038035.2
GCA_003038135.2
GCA_003038215.3
GCA_003043895.1
GCA_003043915.1
GCA_003044015.1
GCA_003044255.1

GCA_003045165.1
GCA_003047005.1
GCA_003047025.1
GCA_003047045.1
GCA_003047065.1
GCA_003047085.1
GCA_003047125.1
GCA_003047145.2
GCA_003047165.1
GCA_003047185.1
GCA_003047205.1
GCA_003048255.1
GCA_003048375.1
GCA_003048595.2
GCA_003048615.2
GCA_003048675.2
GCA_003048685.2
GCA_003048695.2
GCA_003048775.2
GCA_003048835.2
GCA_003048875.2
GCA_003049605.2
GCA_003049785.1
GCA_003049805.1
GCA_003050665.1
GCA_003050685.1
GCA_003051885.1
GCA_003051945.2
GCA_003051965.2
GCA_003051985.2
GCA_003052005.2
GCA_003052025.1
GCA_003052045.1
GCA_003052165.1
GCA_003052645.1
GCA_003052665.1
GCA_003052765.1
GCA_003052785.2
GCA_003053755.1
GCA_003054385.1
GCA_003054425.1
GCA_003054475.1
GCA_003054515.1
GCA_003054535.1
GCA_003054555.1
GCA_003054575.1
GCA_003054785.2
GCA_003055625.1
GCA_003055645.1
GCA_003055835.1
GCA_003057965.1
GCA_003058345.1
GCA_003058405.2
GCA_003058445.1

GCA_003058465.1
GCA_003060725.1
GCA_003060745.1
GCA_003060765.1
GCA_003060785.1
GCA_003060825.1
GCA_003060845.1
GCA_003060865.1
GCA_003061265.1
GCA_003061385.1
GCA_003061515.1
GCA_003063555.1
GCA_003063625.1
GCA_003063785.1
GCA_003063885.1
GCA_003064165.2
GCA_003064365.2
GCA_003064405.2
GCA_003065365.1
GCA_003065405.1
GCA_003065425.1
GCA_003065485.1
GCA_003065605.1
GCA_003067445.2
GCA_003067765.2
GCA_003067925.2
GCA_003067985.2
GCA_003068025.2
GCA_003068145.2
GCA_003069525.1
GCA_003070865.1
GCA_003070885.1
GCA_003071305.1
GCA_003071325.1
GCA_003071365.1
GCA_003071405.1
GCA_003071425.1
GCA_003071445.1
GCA_003071465.1
GCA_003071485.1
GCA_003071525.2
GCA_003071565.1
GCA_003071605.1
GCA_003071625.1
GCA_003071645.1
GCA_003071665.1
GCA_003072465.1
GCA_003072485.1
GCA_003072585.2
GCA_003072605.2
GCA_003072625.1
GCA_003072645.1
GCA_003073235.1
GCA_003073255.1

GCA_003073275.1
GCA_003073315.1
GCA_003073335.1
GCA_003073375.1
GCA_003073395.1
GCA_003073415.1
GCA_003073435.1
GCA_003073455.1
GCA_003073475.1
GCA_003073495.1
GCA_003073515.1
GCA_003073535.1
GCA_003073555.1
GCA_003073575.1
GCA_003073595.1
GCA_003073615.1
GCA_003073635.1
GCA_003073655.1
GCA_003073675.1
GCA_003073695.1
GCA_003073715.1
GCA_003073735.1
GCA_003073755.1
GCA_003073775.1
GCA_003073795.1
GCA_003073815.1
GCA_003073835.1
GCA_003073875.1
GCA_003073895.1
GCA_003073935.1
GCA_003073955.1
GCA_003073975.1
GCA_003073995.1
GCA_003074015.1
GCA_003074035.1
GCA_003074055.1
GCA_003074715.2
GCA_003074735.2
GCA_003074755.2
GCA_003074775.2
GCA_003074855.2
GCA_003074875.2
GCA_003074975.2
GCA_003074995.2
GCA_003076275.1
GCA_003076295.1
GCA_003076315.1
GCA_003076335.1
GCA_003076355.1
GCA_003076375.1
GCA_003076395.1
GCA_003076415.1
GCA_003076435.1
GCA_003076455.1

GCA_003076475.1
GCA_003076495.1
GCA_003076535.1
GCA_003076555.1
GCA_003076795.1
GCA_003076855.1
GCA_003076875.1
GCA_003076895.1
GCA_003085735.1
GCA_003086355.2
GCA_003086595.1
GCA_003095635.1
GCA_003095655.1
GCA_003095675.1
GCA_003095695.1
GCA_003096015.2
GCA_003096035.1
GCA_003096055.1
GCA_003096075.1
GCA_003096095.1
GCA_003096155.1
GCA_003096175.1
GCA_003096215.1
GCA_003097515.1
GCA_003097535.1
GCA_003097555.1
GCA_003097575.1
GCA_003097595.1
GCA_003097695.1
GCA_003099975.1
GCA_003100395.1
GCA_003100575.1
GCA_003101015.1
GCA_003111645.1
GCA_003111665.1
GCA_003111685.1
GCA_003111725.1
GCA_003111745.1
GCA_003111765.1
GCA_003111785.1
GCA_003112145.1
GCA_003112165.1
GCA_003112185.1
GCA_003112205.1
GCA_003112225.1
GCA_003112245.1
GCA_003112395.2
GCA_003113095.2
GCA_003113495.2
GCA_003114835.3
GCA_003119115.1
GCA_003119375.1
GCA_003122105.1
GCA_003122365.1

GCA_003122385.1
GCA_003122405.1
GCA_003122425.1
GCA_003122745.2
GCA_003129525.1
GCA_003130605.1
GCA_003130705.1
GCA_003130755.1
GCA_003130795.1
GCA_003143515.1
GCA_003143535.1
GCA_003143555.1
GCA_003143895.1
GCA_003143915.1
GCA_003144075.1
GCA_003144175.1
GCA_003146705.1
GCA_003147545.1
GCA_003147565.1
GCA_003148355.2
GCA_003148385.1
GCA_003148415.1
GCA_003149495.1
GCA_003149515.1
GCA_003149695.1
GCA_003149715.1
GCA_003149795.1
GCA_003150815.1
GCA_003150835.1
GCA_003150855.2
GCA_003150935.1
GCA_003151025.1
GCA_003151075.1
GCA_003151175.1
GCA_003151255.1
GCA_003171815.2
GCA_003172465.2
GCA_003172975.1
GCA_003172995.1
GCA_003173015.1
GCA_003173275.1
GCA_003173695.1
GCA_003173715.1
GCA_003173735.1
GCA_003173755.1
GCA_003173775.1
GCA_003176795.1
GCA_003176815.1
GCA_003176835.1
GCA_003176855.1
GCA_003176915.1
GCA_003177035.1
GCA_003177055.1
GCA_003177075.1

GCA_003177235.1
GCA_003177255.1
GCA_003180975.1
GCA_003180995.1
GCA_003181015.1
GCA_003181035.1
GCA_003181055.1
GCA_003181075.1
GCA_003181095.1
GCA_003181135.1
GCA_003181155.1
GCA_003181175.1
GCA_003182655.1
GCA_003183825.1
GCA_003183845.2
GCA_003184205.1
GCA_003184225.1
GCA_003184245.1
GCA_003184265.1
GCA_003184305.1
GCA_003184325.1
GCA_003184385.1
GCA_003184405.1
GCA_003184425.1
GCA_003184445.1
GCA_003184465.1
GCA_003184485.1
GCA_003184985.1
GCA_003185005.1
GCA_003185725.2
GCA_003186105.1
GCA_003186125.1
GCA_003186145.1
GCA_003186185.1
GCA_003186205.1
GCA_003186225.1
GCA_003186245.1
GCA_003186265.1
GCA_003186285.1
GCA_003186305.1
GCA_003186415.1
GCA_003186475.1
GCA_003186535.1
GCA_003186565.1
GCA_003186595.1
GCA_003186685.1
GCA_003186765.1
GCA_003193645.1
GCA_003193665.1
GCA_003193685.1
GCA_003193705.1
GCA_003193725.1
GCA_003193745.1
GCA_003193765.1

GCA_003193785.1
GCA_003193885.1
GCA_003193965.1
GCA_003194005.1
GCA_003194025.1
GCA_003194045.1
GCA_003194085.1
GCA_003194245.1
GCA_003194285.1
GCA_003194345.1
GCA_003194405.1
GCA_003199005.1
GCA_003203435.1
GCA_003203455.1
GCA_003203475.1
GCA_003203595.1
GCA_003203615.1
GCA_003203635.1
GCA_003203655.1
GCA_003203675.1
GCA_003203695.1
GCA_003203755.1
GCA_003204075.1
GCA_003204095.1
GCA_003204115.1
GCA_003204135.1
GCA_003204155.1
GCA_003204175.1
GCA_003204265.1
GCA_003204335.1
GCA_003204405.1
GCA_003204465.1
GCA_003204955.1
GCA_003205535.1
GCA_003205815.1
GCA_003205835.1
GCA_003206515.1
GCA_003206535.1
GCA_003208035.1
GCA_003209315.1
GCA_003209395.1
GCA_003213395.1
GCA_003213475.1
GCA_003213565.1
GCA_003213655.1
GCA_003213725.2
GCA_003213775.1
GCA_003213825.2
GCA_003213895.1
GCA_003214015.1
GCA_003214135.1
GCA_003214155.2
GCA_003214255.1
GCA_003214365.1

GCA_003214495.1
GCA_003214575.1
GCA_003214655.1
GCA_003214785.1
GCA_003214895.1
GCA_003215035.1
GCA_003215155.1
GCA_003215265.1
GCA_003215395.1
GCA_003215515.1
GCA_003224315.2
GCA_003224435.2
GCA_003226675.1
GCA_003226695.1
GCA_003226715.1
GCA_003226735.1
GCA_003226755.1
GCA_003226775.1
GCA_003226795.1
GCA_003226815.1
GCA_003226835.1
GCA_003226855.1
GCA_003226875.1
GCA_003227955.1
GCA_003228315.1
GCA_003231095.1
GCA_003231115.1
GCA_003233655.1
GCA_003233675.1
GCA_003233695.1
GCA_003234085.2
GCA_003234115.2
GCA_003234175.2
GCA_003234225.2
GCA_003234255.2
GCA_003234265.2
GCA_003234335.2
GCA_003234365.2
GCA_003234445.2
GCA_003234605.2
GCA_003234655.2
GCA_003234705.2
GCA_003234775.2
GCA_003234795.2
GCA_003234805.2
GCA_003234815.2
GCA_003234885.2
GCA_003234895.2
GCA_003240565.2
GCA_003244395.3
GCA_003253775.1
GCA_003253795.1
GCA_003253815.1
GCA_003254065.1

GCA_003254375.1
GCA_003254785.1
GCA_003254805.1
GCA_003254845.1
GCA_003255835.1
GCA_003255855.1
GCA_003255875.1
GCA_003258315.1
GCA_003258335.1
GCA_003258605.2
GCA_003258705.1
GCA_003260185.2
GCA_003260975.1
GCA_003261035.1
GCA_003261055.1
GCA_003261295.1
GCA_003261355.1
GCA_003261475.1
GCA_003261575.2
GCA_003261955.3
GCA_003264115.1
GCA_003264255.1
GCA_003264275.1
GCA_003264295.1
GCA_003264775.1
GCA_003264795.1
GCA_003264815.1
GCA_003264955.1
GCA_003265005.1
GCA_003265025.1
GCA_003265205.1
GCA_003265225.1
GCA_003265245.1
GCA_003265265.1
GCA_003265285.1
GCA_003265305.2
GCA_003267985.1
GCA_003268535.2
GCA_003268595.1
GCA_003268615.1
GCA_003268635.1
GCA_003268655.1
GCA_003268695.1
GCA_003268715.1
GCA_003269405.1
GCA_003269425.1
GCA_003269445.1
GCA_003269465.1
GCA_003285045.1
GCA_003285065.1
GCA_003285085.1
GCA_003285145.1
GCA_003285165.1
GCA_003285185.1

GCA_003285225.1
GCA_003285245.1
GCA_003285265.1
GCA_003286395.1
GCA_003286415.2
GCA_003286425.2
GCA_003286435.2
GCA_003286645.2
GCA_003286825.2
GCA_003286935.1
GCA_003286955.1
GCA_003286975.1
GCA_003286995.1
GCA_003287015.1
GCA_003287125.1
GCA_003287145.1
GCA_003287165.1
GCA_003287185.1
GCA_003287245.1
GCA_003287895.1
GCA_003287995.1
GCA_003288015.1
GCA_003288035.1
GCA_003288055.1
GCA_003288075.1
GCA_003288095.1
GCA_003288115.1
GCA_003288135.1
GCA_003288155.1
GCA_003288175.1
GCA_003288195.1
GCA_003288235.1
GCA_003288255.1
GCA_003288275.1
GCA_003288295.1
GCA_003288315.1
GCA_003288335.1
GCA_003288355.1
GCA_003288375.1
GCA_003288395.1
GCA_003288415.1
GCA_003288435.1
GCA_003288455.1
GCA_003288475.1
GCA_003288775.1
GCA_003290205.1
GCA_003290225.1
GCA_003290245.1
GCA_003290265.1
GCA_003290325.1
GCA_003290345.1
GCA_003290365.1
GCA_003290385.1
GCA_003290405.1

GCA_003290425.1
GCA_003290445.1
GCA_003293635.1
GCA_003293655.1
GCA_003293695.1
GCA_003293715.1
GCA_003294855.2
GCA_003294895.2
GCA_003294925.2
GCA_003296145.1
GCA_003296175.1
GCA_003296225.1
GCA_003308955.1
GCA_003308975.1
GCA_003312465.1
GCA_003312485.2
GCA_003312525.2
GCA_003312545.2
GCA_003312565.1
GCA_003312585.1
GCA_003312875.1
GCA_003312895.1
GCA_003312915.1
GCA_003313545.1
GCA_003313565.1
GCA_003313585.1
GCA_003314795.2
GCA_003315755.1
GCA_003315775.1
GCA_003316815.2
GCA_003316895.1
GCA_003316915.1
GCA_003316935.1
GCA_003316955.1
GCA_003319215.1
GCA_003319235.1
GCA_003323795.1
GCA_003323815.1
GCA_003323835.1
GCA_003324675.1
GCA_003324715.1
GCA_003324755.1
GCA_003324775.1
GCA_003324795.1
GCA_003324815.1
GCA_003324835.1
GCA_003324855.1
GCA_003324875.1
GCA_003324915.1
GCA_003324935.1
GCA_003324955.1
GCA_003324975.1
GCA_003324995.1
GCA_003325015.1

GCA_003325035.1
GCA_003325075.1
GCA_003325095.1
GCA_003325115.1
GCA_003325135.1
GCA_003325155.1
GCA_003325195.1
GCA_003325215.1
GCA_003325255.1
GCA_003325275.1
GCA_003325315.1
GCA_003325335.1
GCA_003325355.1
GCA_003325375.1
GCA_003325395.1
GCA_003325455.1
GCA_003325475.1
GCA_003325495.1
GCA_003325735.1
GCA_003325955.1
GCA_003325975.1
GCA_003326015.1
GCA_003330725.1
GCA_003330745.1
GCA_003330765.1
GCA_003330785.1
GCA_003330805.1
GCA_003330825.1
GCA_003330845.1
GCA_003330865.1
GCA_003330885.1
GCA_003330905.1
GCA_003330925.1
GCA_003330945.1
GCA_003330965.1
GCA_003332325.1
GCA_003332705.2
GCA_003335165.1
GCA_003336325.1
GCA_003336345.1
GCA_003336365.1
GCA_003336405.1
GCA_003339525.1
GCA_003339545.1
GCA_003339775.1
GCA_003342655.1
GCA_003342735.1
GCA_003342755.1
GCA_003342775.1
GCA_003343225.1
GCA_003343245.1
GCA_003343265.1
GCA_003343285.1
GCA_003343305.1

GCA_003344445.1
GCA_003344465.1
GCA_003344865.1
GCA_003344925.1
GCA_003344965.1
GCA_003345215.1
GCA_003345235.1
GCA_003345255.1
GCA_003345275.1
GCA_003345295.1
GCA_003345315.1
GCA_003345335.1
GCA_003345375.1
GCA_003346755.1
GCA_003346775.1
GCA_003346795.1
GCA_003346815.1
GCA_003346835.1
GCA_003347055.1
GCA_003347075.1
GCA_003347095.1
GCA_003347115.1
GCA_003347135.1
GCA_003350945.1
GCA_003351365.1
GCA_003351385.1
GCA_003351405.1
GCA_003351425.1
GCA_003351445.1
GCA_003351465.1
GCA_003351485.1
GCA_003351525.1
GCA_003351545.1
GCA_003351565.1
GCA_003351585.1
GCA_003351605.1
GCA_003351625.1
GCA_003351645.1
GCA_003351665.1
GCA_003351685.1
GCA_003351705.1
GCA_003351725.1
GCA_003351745.1
GCA_003351765.1
GCA_003351785.1
GCA_003351805.1
GCA_003351825.1
GCA_003351845.1
GCA_003351885.1
GCA_003351905.1
GCA_003351925.1
GCA_003351945.1
GCA_003351985.1
GCA_003352005.1

GCA_003352045.1
GCA_003352065.1
GCA_003352085.1
GCA_003352105.1
GCA_003352125.1
GCA_003352145.1
GCA_003352165.1
GCA_003352185.1
GCA_003352205.1
GCA_003352225.1
GCA_003352345.1
GCA_003352365.1
GCA_003352385.1
GCA_003352405.1
GCA_003352425.1
GCA_003352445.1
GCA_003352465.1
GCA_003352785.1
GCA_003352995.1
GCA_003353065.1
GCA_003353455.1
GCA_003354245.1
GCA_003354265.1
GCA_003354285.1
GCA_003354305.1
GCA_003354325.1
GCA_003354345.1
GCA_003354365.1
GCA_003354385.1
GCA_003354405.1
GCA_003354425.1
GCA_003354445.1
GCA_003354465.1
GCA_003354485.1
GCA_003354505.1
GCA_003354525.1
GCA_003354545.1
GCA_003354565.1
GCA_003354585.1
GCA_003354605.1
GCA_003354625.1
GCA_003354645.1
GCA_003354665.1
GCA_003354685.1
GCA_003354705.1
GCA_003354725.1
GCA_003354745.1
GCA_003354765.1
GCA_003354785.1
GCA_003354805.1
GCA_003354825.1
GCA_003354845.1
GCA_003354865.1
GCA_003354885.1

GCA_003354905.1
GCA_003354925.1
GCA_003354945.1
GCA_003354965.1
GCA_003354985.1
GCA_003355005.1
GCA_003355025.1
GCA_003355045.1
GCA_003355065.1
GCA_003355085.1
GCA_003355135.1
GCA_003355175.1
GCA_003355215.1
GCA_003355235.1
GCA_003355255.1
GCA_003355275.1
GCA_003355295.1
GCA_003355315.1
GCA_003355335.1
GCA_003355375.1
GCA_003355395.1
GCA_003355415.1
GCA_003355435.1
GCA_003355455.1
GCA_003355475.1
GCA_003355515.1
GCA_003359215.2
GCA_003359225.2
GCA_003359255.2
GCA_003359275.2
GCA_003359295.2
GCA_003359305.2
GCA_003359315.2
GCA_003359355.2
GCA_003359375.2
GCA_003359395.2
GCA_003359435.2
GCA_003359505.2
GCA_003363675.2
GCA_003363755.1
GCA_003363775.1
GCA_003366055.1
GCA_003366075.1
GCA_003367075.2
GCA_003367295.1
GCA_003367315.1
GCA_003367335.1
GCA_003367355.1
GCA_003367375.1
GCA_003367415.1
GCA_003367555.1
GCA_003367705.1
GCA_003367885.1
GCA_003367905.1

GCA_003368025.1
GCA_003368045.1
GCA_003368065.1
GCA_003368085.1
GCA_003368105.1
GCA_003368125.1
GCA_003368145.1
GCA_003368165.1
GCA_003368185.1
GCA_003368205.1
GCA_003368225.1
GCA_003368245.1
GCA_003368325.1
GCA_003369735.1
GCA_003369755.1
GCA_003369775.1
GCA_003369795.1
GCA_003382565.3
GCA_003382725.1
GCA_003382775.1
GCA_003382795.1
GCA_003382815.1
GCA_003382835.1
GCA_003382855.1
GCA_003382875.1
GCA_003382895.1
GCA_003382915.1
GCA_003382935.1
GCA_003383595.3
GCA_003390415.1
GCA_003390455.1
GCA_003390475.1
GCA_003390495.1
GCA_003390735.1
GCA_003390755.1
GCA_003390995.1
GCA_003391095.1
GCA_003391135.1
GCA_003391255.1
GCA_003391275.1
GCA_003391295.1
GCA_003391315.1
GCA_003391335.1
GCA_003391375.1
GCA_003391395.1
GCA_003391415.1
GCA_003394085.1
GCA_003394105.1
GCA_003399575.2
GCA_003399775.2
GCA_003399795.2
GCA_003399845.2
GCA_003399885.2
GCA_003399895.2

GCA_003399945.2
GCA_003400045.2
GCA_003402535.2
GCA_003402855.1
GCA_003402875.1
GCA_003402895.1
GCA_003402915.1
GCA_003402935.1
GCA_003402955.1
GCA_003403075.1
GCA_003403095.1
GCA_003403115.1
GCA_003403135.1
GCA_003403315.2
GCA_003408495.1
GCA_003408515.1
GCA_003408535.1
GCA_003408555.1
GCA_003408575.1
GCA_003408595.1
GCA_003408615.1
GCA_003408635.1
GCA_003409075.1
GCA_003410255.1
GCA_003410275.1
GCA_003410295.1
GCA_003410315.1
GCA_003410335.1
GCA_003410355.1
GCA_003410375.1
GCA_003410415.1
GCA_003411785.2
GCA_003413625.1
GCA_003425445.1
GCA_003425465.1
GCA_003425485.1
GCA_003425505.1
GCA_003425525.1
GCA_003425545.1
GCA_003425565.1
GCA_003425585.1
GCA_003425605.1
GCA_003425625.1
GCA_003425645.1
GCA_003425715.1
GCA_003425765.1
GCA_003425815.1
GCA_003425835.1
GCA_003425885.1
GCA_003425915.1
GCA_003425935.1
GCA_003425955.1
GCA_003425985.1
GCA_003426145.1

GCA_003427055.1
GCA_003427415.1
GCA_003428335.1
GCA_003428355.1
GCA_003428375.1
GCA_003428395.1
GCA_003428425.1
GCA_003428825.1
GCA_003428925.1
GCA_003428965.1
GCA_003429185.1
GCA_003429205.1
GCA_003429265.1
GCA_003429285.1
GCA_003429305.1
GCA_003429325.1
GCA_003429365.1
GCA_003429385.1
GCA_003429405.1
GCA_003429425.1
GCA_003429445.1
GCA_003429465.1
GCA_003429505.1
GCA_003429525.1
GCA_003429545.1
GCA_003429625.1
GCA_003430865.2
GCA_003431345.1
GCA_003431365.1
GCA_003431385.1
GCA_003431405.1
GCA_003431425.1
GCA_003431825.1
GCA_003431845.1
GCA_003431865.1
GCA_003431885.1
GCA_003431975.1
GCA_003432145.1
GCA_003432165.1
GCA_003432185.1
GCA_003432205.1
GCA_003432245.1
GCA_003432265.1
GCA_003432285.1
GCA_003432305.1
GCA_003432345.1
GCA_003432365.1
GCA_003432385.1
GCA_003432405.1
GCA_003432425.1
GCA_003433235.1
GCA_003433255.1
GCA_003433275.1
GCA_003433375.1

GCA_003433395.1
GCA_003433415.1
GCA_003433515.1
GCA_003441595.1
GCA_003441615.1
GCA_003443395.1
GCA_003443515.1
GCA_003443535.1
GCA_003443555.1
GCA_003443575.1
GCA_003443595.1
GCA_003443615.1
GCA_003443635.1
GCA_003443655.1
GCA_003443675.1
GCA_003443695.1
GCA_003443715.1
GCA_003443735.1
GCA_003443755.1
GCA_003443775.1
GCA_003443795.1
GCA_003443815.1
GCA_003443835.1
GCA_003443855.2
GCA_003443875.1
GCA_003444755.1
GCA_003444775.1
GCA_003445395.2
GCA_003445415.1
GCA_003454715.1
GCA_003454755.1
GCA_003454775.1
GCA_003454795.1
GCA_003456975.1
GCA_003457015.1
GCA_003457035.1
GCA_003481905.1
GCA_003481965.1
GCA_003482035.1
GCA_003482065.1
GCA_003482125.1
GCA_003482165.1
GCA_003482225.1
GCA_003482255.1
GCA_003482305.1
GCA_003482325.1
GCA_003482345.1
GCA_003482365.1
GCA_003485445.1
GCA_003490105.1
GCA_003490125.1
GCA_003491165.1
GCA_003491185.1
GCA_003491205.1

GCA_003491225.1
GCA_003491245.1
GCA_003491265.1
GCA_003491325.1
GCA_003491345.1
GCA_003491365.1
GCA_003491385.1
GCA_003491405.1
GCA_003491425.1
GCA_003515045.1
GCA_003515105.1
GCA_003515145.1
GCA_003515165.1
GCA_003515185.1
GCA_003515205.1
GCA_003515225.1
GCA_003515245.1
GCA_003515265.1
GCA_003515285.1
GCA_003515305.1
GCA_003515325.1
GCA_003515345.1
GCA_003515365.1
GCA_003515385.1
GCA_003515405.1
GCA_003515425.1
GCA_003515445.1
GCA_003515465.1
GCA_003515485.1
GCA_003515505.1
GCA_003515525.1
GCA_003515545.1
GCA_003515565.1
GCA_003515585.1
GCA_003515605.1
GCA_003515625.1
GCA_003515645.1
GCA_003515915.2
GCA_003515965.1
GCA_003515985.1
GCA_003516005.1
GCA_003516025.1
GCA_003516045.1
GCA_003516065.1
GCA_003516085.1
GCA_003516125.3
GCA_003516145.1
GCA_003516205.1
GCA_003522555.1
GCA_003522605.1
GCA_003522665.1
GCA_003522705.1
GCA_003522785.1
GCA_003522845.1

GCA_003522885.1
GCA_003522965.1
GCA_003534205.1
GCA_003544815.1
GCA_003544835.1
GCA_003544855.1
GCA_003544875.1
GCA_003544895.1
GCA_003544915.1
GCA_003544935.1
GCA_003546465.1
GCA_003546645.1
GCA_003546665.1
GCA_003546865.1
GCA_003546885.1
GCA_003546905.1
GCA_003546955.1
GCA_003546975.1
GCA_003547015.1
GCA_003547035.1
GCA_003547055.1
GCA_003547115.1
GCA_003555505.2
GCA_003555525.1
GCA_003555545.1
GCA_003568565.1
GCA_003568585.1
GCA_003568605.1
GCA_003568625.1
GCA_003568645.1
GCA_003568825.1
GCA_003568845.1
GCA_003568865.1
GCA_003569745.1
GCA_003570765.1
GCA_003571425.1
GCA_003571445.1
GCA_003571465.1
GCA_003571505.1
GCA_003571525.1
GCA_003571545.1
GCA_003571585.1
GCA_003571605.1
GCA_003571625.1
GCA_003571645.1
GCA_003571665.1
GCA_003571685.1
GCA_003571705.1
GCA_003571725.1
GCA_003571745.1
GCA_003571765.1
GCA_003571785.1
GCA_003571805.1
GCA_003571825.1

GCA_003573445.1
GCA_003573465.1
GCA_003573485.1
GCA_003573835.1
GCA_003573855.1
GCA_003573875.1
GCA_003573895.1
GCA_003573915.1
GCA_003573935.1
GCA_003573955.1
GCA_003573975.1
GCA_003573995.1
GCA_003574135.1
GCA_003574155.1
GCA_003574175.1
GCA_003574195.1
GCA_003574215.1
GCA_003574235.1
GCA_003574255.1
GCA_003574275.1
GCA_003574315.2
GCA_003574835.2
GCA_003574925.1
GCA_003574945.1
GCA_003574965.1
GCA_003574985.1
GCA_003575005.1
GCA_003576455.2
GCA_003581245.2
GCA_003584565.3
GCA_003584585.1
GCA_003584645.1
GCA_003584665.1
GCA_003584685.1
GCA_003584705.1
GCA_003584725.1
GCA_003584745.1
GCA_003585765.1
GCA_003586025.1
GCA_003586045.1
GCA_003586065.1
GCA_003586085.1
GCA_003586385.1
GCA_003586445.1
GCA_003586485.1
GCA_003586525.1
GCA_003589725.1
GCA_003589745.1
GCA_003589785.1
GCA_003589805.1
GCA_003589825.1
GCA_003589845.1
GCA_003589865.1
GCA_003589885.1

GCA_003589905.1
GCA_003589925.1
GCA_003589945.1
GCA_003590055.1
GCA_003590075.1
GCA_003590145.2
GCA_003590565.1
GCA_003590605.1
GCA_003590665.1
GCA_003590695.1
GCA_003590755.1
GCA_003591575.1
GCA_003591595.1
GCA_003591615.1
GCA_003594935.2
GCA_003595175.1
GCA_003595195.1
GCA_003595235.1
GCA_003595305.1
GCA_003595345.1
GCA_003595365.1
GCA_003595385.1
GCA_003595405.1
GCA_003595425.1
GCA_003595445.1
GCA_003595465.1
GCA_003595485.1
GCA_003595505.1
GCA_003595525.1
GCA_003595545.1
GCA_003595585.1
GCA_003595605.1
GCA_003595625.1
GCA_003595645.1
GCA_003595725.1
GCA_003596365.3
GCA_003597595.1
GCA_003597615.1
GCA_003597635.1
GCA_003597655.1
GCA_003597695.1
GCA_003597715.1
GCA_003597735.1
GCA_003597755.1
GCA_003600355.1
GCA_003600625.1
GCA_003600685.1
GCA_003606225.1
GCA_003606245.1
GCA_003606265.1
GCA_003606285.1
GCA_003606305.1
GCA_003606325.3
GCA_003606345.3

GCA_003606365.2
GCA_003606405.1
GCA_003606425.1
GCA_003609695.1
GCA_003609715.1
GCA_003609735.1
GCA_003609755.1
GCA_003609775.1
GCA_003609795.1
GCA_003609815.1
GCA_003609835.1
GCA_003609855.1
GCA_003609895.1
GCA_003609915.1
GCA_003609935.1
GCA_003609955.1
GCA_003609975.1
GCA_003609995.1
GCA_003610015.1
GCA_003610955.1
GCA_003610975.1
GCA_003610995.1
GCA_003611015.1
GCA_003611035.1
GCA_003611275.1
GCA_003612695.1
GCA_003612715.1
GCA_003612735.1
GCA_003612755.1
GCA_003612775.1
GCA_003612795.1
GCA_003612835.1
GCA_003612855.1
GCA_003612895.1
GCA_003612915.1
GCA_003612935.1
GCA_003612955.1
GCA_003613035.1
GCA_003613065.1
GCA_003613595.1
GCA_003614235.1
GCA_003614435.1
GCA_003614815.1
GCA_003614835.1
GCA_003626935.2
GCA_003626955.1
GCA_003626975.1
GCA_003626995.1
GCA_003627015.2
GCA_003627035.1
GCA_003627055.1
GCA_003627075.1
GCA_003627095.1
GCA_003627135.1

GCA_003627155.1
GCA_003627195.1
GCA_003627215.1
GCA_003627235.1
GCA_003627255.1
GCA_003627275.1
GCA_003627295.1
GCA_003627315.1
GCA_003627335.1
GCA_003627355.1
GCA_003627375.1
GCA_003627395.2
GCA_003627485.2
GCA_003627735.1
GCA_003627755.1
GCA_003627775.1
GCA_003627795.1
GCA_003627815.1
GCA_003627835.1
GCA_003627855.1
GCA_003627875.1
GCA_003627995.1
GCA_003628555.1
GCA_003628575.1
GCA_003628595.1
GCA_003628635.1
GCA_003628655.1
GCA_003628675.1
GCA_003628695.1
GCA_003628735.1
GCA_003628755.1
GCA_003628775.1
GCA_003636855.2
GCA_003640545.1
GCA_003640565.1
GCA_003640585.1
GCA_003640605.1
GCA_003641105.1
GCA_003641125.1
GCA_003641145.1
GCA_003641165.1
GCA_003641185.1
GCA_003641205.1
GCA_003641225.1
GCA_003641245.1
GCA_003641265.1
GCA_003641285.1
GCA_003641305.1
GCA_003641325.1
GCA_003660085.1
GCA_003660105.1
GCA_003660125.1
GCA_003660165.1
GCA_003660185.1

GCA_003660205.1
GCA_003663725.1
GCA_003663745.1
GCA_003663765.1
GCA_003665195.1
GCA_003665215.1
GCA_003665235.1
GCA_003665255.1
GCA_003665275.1
GCA_003665295.1
GCA_003665315.1
GCA_003665335.1
GCA_003665355.1
GCA_003665375.1
GCA_003665395.1
GCA_003665415.1
GCA_003665435.1
GCA_003665455.1
GCA_003665475.1
GCA_003665535.1
GCA_003665555.1
GCA_003665575.1
GCA_003665595.1
GCA_003665615.1
GCA_003665635.1
GCA_003665655.1
GCA_003665675.1
GCA_003666425.1
GCA_003666445.1
GCA_003666465.1
GCA_003667385.1
GCA_003667405.1
GCA_003667425.1
GCA_003667705.1
GCA_003667725.1
GCA_003667745.1
GCA_003667765.1
GCA_003667785.1
GCA_003667805.1
GCA_003667825.1
GCA_003667845.1
GCA_003667885.1
GCA_003667905.1
GCA_003667925.1
GCA_003667945.1
GCA_003667965.1
GCA_003668775.1
GCA_003668795.1
GCA_003668995.1
GCA_003669015.1
GCA_003669035.1
GCA_003671915.1
GCA_003671935.1
GCA_003671955.1

GCA_003671975.1
GCA_003671995.1
GCA_003675305.1
GCA_003675325.1
GCA_003675345.1
GCA_003675365.1
GCA_003687415.1
GCA_003687485.1
GCA_003688615.2
GCA_003691345.1
GCA_003691365.1
GCA_003691385.1
GCA_003691405.1
GCA_003691425.1
GCA_003691445.1
GCA_003691465.1
GCA_003691485.1
GCA_003691505.1
GCA_003691525.1
GCA_003691545.1
GCA_003691565.1
GCA_003691675.1
GCA_003691695.1
GCA_003692595.1
GCA_003692615.1
GCA_003692675.1
GCA_003692695.1
GCA_003697165.2
GCA_003697205.1
GCA_003697225.1
GCA_003697245.1
GCA_003697265.1
GCA_003698205.1
GCA_003698225.1
GCA_003703795.1
GCA_003704285.1
GCA_003704305.1
GCA_003710085.1
GCA_003710105.1
GCA_003710125.1
GCA_003710145.1
GCA_003710165.1
GCA_003710185.1
GCA_003710205.1
GCA_003710225.1
GCA_003711005.1
GCA_003711025.1
GCA_003711045.1
GCA_003711065.1
GCA_003711105.1
GCA_003711125.1
GCA_003711145.1
GCA_003711185.1
GCA_003711205.1

GCA_003711225.1
GCA_003711265.1
GCA_003711605.1
GCA_003713025.1
GCA_003713045.1
GCA_003713065.1
GCA_003713085.1
GCA_003713105.1
GCA_003716765.1
GCA_003716785.1
GCA_003716855.1
GCA_003716875.1
GCA_003716915.1
GCA_003716935.1
GCA_003716955.1
GCA_003716975.1
GCA_003716995.1
GCA_003717015.1
GCA_003717035.1
GCA_003717055.1
GCA_003717075.1
GCA_003717095.1
GCA_003717115.1
GCA_003717135.1
GCA_003717215.1
GCA_003717285.1
GCA_003717355.1
GCA_003717395.1
GCA_003717435.1
GCA_003717455.1
GCA_003717475.1
GCA_003717515.1
GCA_003717535.1
GCA_003717575.1
GCA_003717595.1
GCA_003717615.1
GCA_003717635.1
GCA_003717655.1
GCA_003717675.1
GCA_003717695.1
GCA_003717715.1
GCA_003717735.1
GCA_003717755.1
GCA_003717775.1
GCA_003717795.1
GCA_003717815.1
GCA_003717835.1
GCA_003717855.1
GCA_003717875.1
GCA_003717895.1
GCA_003717915.1
GCA_003717935.1
GCA_003717955.1
GCA_003717975.1

GCA_003717995.1
GCA_003718015.1
GCA_003718035.1
GCA_003718055.1
GCA_003718075.1
GCA_003718095.1
GCA_003718115.1
GCA_003718135.1
GCA_003718155.1
GCA_003718175.1
GCA_003718195.1
GCA_003718235.1
GCA_003718255.1
GCA_003718275.1
GCA_003718295.1
GCA_003718315.1
GCA_003718335.1
GCA_003718355.1
GCA_003718375.1
GCA_003718395.1
GCA_003718415.1
GCA_003718435.1
GCA_003718455.1
GCA_003718475.1
GCA_003718495.1
GCA_003718515.1
GCA_003718535.1
GCA_003718555.1
GCA_003718575.1
GCA_003718595.1
GCA_003718615.1
GCA_003718635.1
GCA_003718655.1
GCA_003718715.1
GCA_003718735.1
GCA_003719195.1
GCA_003719215.1
GCA_003719235.1
GCA_003719255.1
GCA_003719555.1
GCA_003719595.1
GCA_003719615.1
GCA_003719755.1
GCA_003719775.1
GCA_003721155.4
GCA_003721455.1
GCA_003721475.1
GCA_003721495.1
GCA_003722175.1
GCA_003722195.1
GCA_003722215.1
GCA_003722255.1
GCA_003722275.1
GCA_003722295.1

GCA_003722335.1
GCA_003722375.1
GCA_003726095.1
GCA_003726115.1
GCA_003726935.1
GCA_003732505.1
GCA_003732525.1
GCA_003790485.1
GCA_003790505.1
GCA_003790525.1
GCA_003795065.1
GCA_003795085.1
GCA_003795105.1
GCA_003795125.1
GCA_003795145.1
GCA_003797775.2
GCA_003798105.1
GCA_003798125.1
GCA_003798145.1
GCA_003798165.1
GCA_003798205.1
GCA_003798225.1
GCA_003798245.1
GCA_003798265.1
GCA_003798285.1
GCA_003798305.1
GCA_003798325.1
GCA_003798345.1
GCA_003798365.1
GCA_003798385.1
GCA_003798405.1
GCA_003798425.1
GCA_003798445.1
GCA_003798465.1
GCA_003812065.1
GCA_003812085.1
GCA_003812105.1
GCA_003812125.1
GCA_003812145.1
GCA_003812165.1
GCA_003812185.1
GCA_003812205.1
GCA_003812225.1
GCA_003812245.1
GCA_003812265.1
GCA_003812305.1
GCA_003812325.1
GCA_003812345.1
GCA_003812365.1
GCA_003812385.1
GCA_003812405.1
GCA_003812425.1
GCA_003812445.1
GCA_003812465.1

GCA_003812485.1
GCA_003812505.1
GCA_003812525.1
GCA_003812545.1
GCA_003812565.1
GCA_003812585.1
GCA_003812605.1
GCA_003812625.1
GCA_003812645.1
GCA_003812665.1
GCA_003812685.1
GCA_003812705.1
GCA_003812725.1
GCA_003812745.1
GCA_003812765.1
GCA_003812785.1
GCA_003812805.1
GCA_003812825.1
GCA_003812845.1
GCA_003812865.1
GCA_003812885.1
GCA_003812905.1
GCA_003812925.1
GCA_003812945.1
GCA_003812985.1
GCA_003813005.1
GCA_003813025.1
GCA_003813045.1
GCA_003813065.1
GCA_003813085.1
GCA_003813125.1
GCA_003813145.1
GCA_003813165.1
GCA_003813965.1
GCA_003813985.1
GCA_003814005.1
GCA_003814285.1
GCA_003814305.1
GCA_003814325.1
GCA_003814345.1
GCA_003814365.1
GCA_003814385.1
GCA_003814405.1
GCA_003814425.1
GCA_003815615.1
GCA_003815635.1
GCA_003815655.1
GCA_003815675.1
GCA_003815695.1
GCA_003815715.1
GCA_003815735.1
GCA_003815755.1
GCA_003815775.1
GCA_003815795.1

GCA_003815815.1
GCA_003815835.1
GCA_003815855.1
GCA_003815875.1
GCA_003815895.1
GCA_003815915.1
GCA_003815935.1
GCA_003815955.1
GCA_003815975.1
GCA_003815995.1
GCA_003816035.1
GCA_003827735.1
GCA_003827835.1
GCA_003827915.1
GCA_003850165.1
GCA_003850185.1
GCA_003850245.1
GCA_003850345.1
GCA_003850365.1
GCA_003850385.1
GCA_003850405.1
GCA_003850425.1
GCA_003850445.1
GCA_003850465.1
GCA_003850485.1
GCA_003850505.1
GCA_003850525.1
GCA_003850545.1
GCA_003850565.1
GCA_003850585.1
GCA_003850605.1
GCA_003851145.1
GCA_003851165.1
GCA_003851205.1
GCA_003851225.1
GCA_003851265.1
GCA_003851285.1
GCA_003851305.1
GCA_003851345.1
GCA_003851365.1
GCA_003851385.1
GCA_003851405.1
GCA_003851425.1
GCA_003851445.1
GCA_003851465.1
GCA_003851495.1
GCA_003851525.1
GCA_003851555.1
GCA_003851585.1
GCA_003851605.1
GCA_003851645.1
GCA_003851685.1
GCA_003851705.1
GCA_003851745.1

GCA_003851785.1
GCA_003851805.1
GCA_003851835.1
GCA_003851865.1
GCA_003851905.1
GCA_003851925.1
GCA_003851955.1
GCA_003851985.1
GCA_003852005.1
GCA_003852025.1
GCA_003852045.1
GCA_003852065.1
GCA_003852235.1
GCA_003852255.1
GCA_003852275.1
GCA_003852295.1
GCA_003852315.1
GCA_003852335.1
GCA_003852355.1
GCA_003852375.1
GCA_003854895.1
GCA_003854915.1
GCA_003854935.1
GCA_003855095.1
GCA_003855115.1
GCA_003855135.1
GCA_003855175.1
GCA_003855195.1
GCA_003855215.1
GCA_003855235.1
GCA_003855275.1
GCA_003855295.1
GCA_003855315.1
GCA_003855355.1
GCA_003855375.1
GCA_003855395.1
GCA_003855415.1
GCA_003855435.1
GCA_003855455.1
GCA_003855475.1
GCA_003855495.1
GCA_003855515.1
GCA_003855535.1
GCA_003855555.1
GCA_003855575.1
GCA_003855595.1
GCA_003855615.1
GCA_003855635.1
GCA_003855655.1
GCA_003856395.1
GCA_003856415.1
GCA_003856435.1
GCA_003856455.1
GCA_003856475.1

GCA_003856495.1
GCA_003856555.1
GCA_003856575.1
GCA_003856595.1
GCA_003856615.1
GCA_003856635.1
GCA_003856655.1
GCA_003856675.1
GCA_003856695.1
GCA_003856715.1
GCA_003856735.1
GCA_003856755.1
GCA_003856815.1
GCA_003856975.1
GCA_003856995.1
GCA_003860285.1
GCA_003860305.1
GCA_003860325.1
GCA_003860345.1
GCA_003860365.1
GCA_003860385.1
GCA_003860405.1
GCA_003860425.1
GCA_003860445.1
GCA_003860465.1
GCA_003860485.1
GCA_003860505.1
GCA_003860525.1
GCA_003860545.1
GCA_003860565.1
GCA_003860585.1
GCA_003860605.1
GCA_003860625.1
GCA_003860645.2
GCA_003864015.1
GCA_003864035.1
GCA_003864055.1
GCA_003864075.1
GCA_003864095.1
GCA_003864115.1
GCA_003864155.1
GCA_003866015.1
GCA_003868675.1
GCA_003900515.2
GCA_003902395.2
GCA_003925855.2
GCA_003925875.1
GCA_003925895.1
GCA_003925915.1
GCA_003925935.1
GCA_003925955.1
GCA_003925975.1
GCA_003931755.1
GCA_003931775.1

GCA_003931795.1
GCA_003931815.1
GCA_003931835.1
GCA_003931855.1
GCA_003931875.1
GCA_003932015.2
GCA_003932035.1
GCA_003932055.1
GCA_003932075.1
GCA_003932715.1
GCA_003932735.1
GCA_003932815.1
GCA_003932915.1
GCA_003932935.1
GCA_003932965.1
GCA_003932995.1
GCA_003934185.1
GCA_003935895.2
GCA_003937135.2
GCA_003938225.2
GCA_003938405.2
GCA_003939045.1
GCA_003939065.1
GCA_003939085.1
GCA_003939325.2
GCA_003939335.2
GCA_003939355.2
GCA_003940765.1
GCA_003940785.1
GCA_003940805.1
GCA_003940825.1
GCA_003942025.1
GCA_003944565.2
GCA_003944865.1
GCA_003945285.1
GCA_003945305.1
GCA_003945325.1
GCA_003945345.1
GCA_003945365.1
GCA_003945385.1
GCA_003945405.1
GCA_003945425.1
GCA_003945505.1
GCA_003945525.1
GCA_003945545.1
GCA_003945765.1
GCA_003948085.2
GCA_003948455.2
GCA_003948535.2
GCA_003949115.2
GCA_003949595.2
GCA_003949725.2
GCA_003949975.1
GCA_003950015.1

GCA_003950035.1
GCA_003950055.1
GCA_003950215.1
GCA_003950235.1
GCA_003950255.1
GCA_003950275.1
GCA_003952205.1
GCA_003952225.1
GCA_003952245.1
GCA_003952265.1
GCA_003952285.1
GCA_003952305.1
GCA_003952325.1
GCA_003952345.1
GCA_003952365.1
GCA_003952385.1
GCA_003952405.1
GCA_003952425.1
GCA_003952445.1
GCA_003952465.1
GCA_003952485.1
GCA_003952525.1
GCA_003952625.1
GCA_003952645.1
GCA_003952665.1
GCA_003952685.1
GCA_003952705.1
GCA_003952725.1
GCA_003952785.1
GCA_003952825.1
GCA_003952845.1
GCA_003952865.1
GCA_003952885.1
GCA_003953005.1
GCA_003953885.1
GCA_003953905.1
GCA_003955715.1
GCA_003955735.1
GCA_003955755.1
GCA_003955775.1
GCA_003955795.1
GCA_003955815.1
GCA_003955865.1
GCA_003955885.1
GCA_003955905.1
GCA_003955945.1
GCA_003955965.1
GCA_003956005.1
GCA_003956025.1
GCA_003956065.1
GCA_003956085.1
GCA_003956105.2
GCA_003956125.2
GCA_003956145.2

GCA_003956165.1
GCA_003956185.1
GCA_003956205.1
GCA_003956225.1
GCA_003956245.1
GCA_003956265.1
GCA_003956285.1
GCA_003956305.1
GCA_003956325.1
GCA_003956345.1
GCA_003956365.1
GCA_003956385.1
GCA_003956405.1
GCA_003956425.1
GCA_003956445.1
GCA_003956465.1
GCA_003956485.1
GCA_003957315.1
GCA_003957335.1
GCA_003957355.1
GCA_003957375.1
GCA_003957395.1
GCA_003957415.1
GCA_003957435.1
GCA_003957745.1
GCA_003957785.1
GCA_003957805.1
GCA_003957825.1
GCA_003963515.1
GCA_003963535.1
GCA_003963555.1
GCA_003964795.2
GCA_003965345.2
GCA_003966295.1
GCA_003966315.1
GCA_003966335.1
GCA_003966365.1
GCA_003966385.1
GCA_003966405.1
GCA_003966425.1
GCA_003966445.1
GCA_003966465.1
GCA_003966485.1
GCA_003966505.1
GCA_003966525.1
GCA_003966545.1
GCA_003966565.1
GCA_003966585.1
GCA_003966605.1
GCA_003966625.1
GCA_003966655.1
GCA_003966675.1
GCA_003966695.1
GCA_003966715.1

GCA_003966735.1
GCA_003966755.1
GCA_003966775.1
GCA_003966795.1
GCA_003966815.1
GCA_003966835.1
GCA_003966855.1
GCA_003966895.1
GCA_003966915.1
GCA_003966935.1
GCA_003966955.1
GCA_003966975.1
GCA_003967015.1
GCA_003967035.1
GCA_003967055.1
GCA_003967075.1
GCA_003967095.1
GCA_003967115.1
GCA_003967135.1
GCA_003967155.2
GCA_003967195.1
GCA_003967215.2
GCA_003967235.2
GCA_003967275.1
GCA_003970675.1
GCA_003970695.1
GCA_003970715.1
GCA_003970735.1
GCA_003970805.2
GCA_003971195.1
GCA_003971215.1
GCA_003971255.1
GCA_003971285.1
GCA_003971305.1
GCA_003971325.1
GCA_003971345.1
GCA_003971365.1
GCA_003971445.1
GCA_003971465.1
GCA_003971485.1
GCA_003971525.1
GCA_003971545.1
GCA_003971565.1
GCA_003971585.1
GCA_003986605.1
GCA_003986635.1
GCA_003986655.1
GCA_003986675.1
GCA_003990335.1
GCA_003990355.1
GCA_003990375.1
GCA_003990395.1
GCA_003990515.2
GCA_003990705.1

GCA_003991015.1
GCA_003991035.1
GCA_003991055.1
GCA_003991075.1
GCA_003991095.1
GCA_003991115.1
GCA_003991135.1
GCA_003991155.1
GCA_003991175.1
GCA_003991425.1
GCA_003991465.1
GCA_003991565.1
GCA_003991855.1
GCA_003991875.1
GCA_003991975.1
GCA_003992745.1
GCA_003994375.1
GCA_003994395.1
GCA_003994415.1
GCA_003994555.1
GCA_003994575.1
GCA_003994595.1
GCA_003994615.1
GCA_003994635.1
GCA_003994655.1
GCA_003994675.1
GCA_003994695.1
GCA_003994715.1
GCA_003994735.1
GCA_003994755.1
GCA_003994815.1
GCA_003994855.1
GCA_003994915.1
GCA_003999215.1
GCA_003999235.1
GCA_003999255.1
GCA_003999275.1
GCA_003999295.1
GCA_003999315.1
GCA_003999335.1
GCA_003999355.1
GCA_003999445.1
GCA_003999465.1
GCA_003999485.1
GCA_003999505.1
GCA_003999525.1
GCA_003999545.1
GCA_003999565.1
GCA_003999605.1
GCA_003999645.1
GCA_003999665.1
GCA_003999715.1
GCA_003999735.1
GCA_003999795.1

GCA_004000475.1
GCA_004000565.1
GCA_004000605.1
GCA_004001105.1
GCA_004001165.1
GCA_004001185.1
GCA_004001205.1
GCA_004001245.1
GCA_004001265.1
GCA_004001285.1
GCA_004001305.1
GCA_004001325.1
GCA_004005995.1
GCA_004006015.1
GCA_004006035.1
GCA_004006055.1
GCA_004006115.1
GCA_004006155.1
GCA_004006175.1
GCA_004006195.1
GCA_004006235.1
GCA_004006295.1
GCA_004006315.1
GCA_004006335.1
GCA_004006395.2
GCA_004006435.1
GCA_004006455.1
GCA_004006475.1
GCA_004006495.1
GCA_004006515.1
GCA_004006545.1
GCA_004006575.1
GCA_004006615.1
GCA_004006635.1
GCA_004006735.1
GCA_004006815.1
GCA_004006875.1
GCA_004006935.1
GCA_004007035.1
GCA_004007095.1
GCA_004007155.1
GCA_004007215.1
GCA_004007275.1
GCA_004007335.1
GCA_004007415.1
GCA_004007475.1
GCA_004007535.1
GCA_004007625.1
GCA_004008035.1
GCA_004008095.1
GCA_004008155.1
GCA_004008215.1
GCA_004008295.1
GCA_004008355.1

GCA_004008415.1
GCA_004008475.1
GCA_004008555.1
GCA_004008595.1
GCA_004008655.1
GCA_004008715.1
GCA_004008795.1
GCA_004008855.1
GCA_004008915.1
GCA_004008935.1
GCA_004008955.1
GCA_004008975.1
GCA_004008995.1
GCA_004009015.1
GCA_004009035.1
GCA_004009055.1
GCA_004009075.1
GCA_004009095.1
GCA_004009115.1
GCA_004009135.1
GCA_004009155.1
GCA_004009175.1
GCA_004009195.1
GCA_004009215.1
GCA_004009235.1
GCA_004009255.1
GCA_004009275.1
GCA_004009295.1
GCA_004009315.1
GCA_004009335.1
GCA_004009355.1
GCA_004009375.1
GCA_004009395.1
GCA_004009415.1
GCA_004009435.1
GCA_004009455.1
GCA_004009475.1
GCA_004009495.1
GCA_004009515.1
GCA_004009535.1
GCA_004009555.1
GCA_004009575.1
GCA_004009595.1
GCA_004009615.1
GCA_004009635.1
GCA_004009655.1
GCA_004009675.1
GCA_004009695.1
GCA_004009715.1
GCA_004009735.1
GCA_004009755.1
GCA_004009775.1
GCA_004009795.1
GCA_004009815.1

GCA_004010575.1
GCA_004010595.1
GCA_004010615.1
GCA_004010635.1
GCA_004010655.1
GCA_004010675.1
GCA_004010695.1
GCA_004010715.1
GCA_004010735.1
GCA_004010755.1
GCA_004010775.1
GCA_004010855.1
GCA_004010875.1
GCA_004010895.1
GCA_004010915.1
GCA_004010935.1
GCA_004010955.1
GCA_004010975.1
GCA_004010995.1
GCA_004011015.1
GCA_004011035.1
GCA_004011055.1
GCA_004011075.1
GCA_004011095.1
GCA_004011115.1
GCA_004011135.1
GCA_004011315.1
GCA_004011755.1
GCA_004011905.1
GCA_004014695.1
GCA_004014715.1
GCA_004014755.2
GCA_004014775.2
GCA_004014855.1
GCA_004014875.1
GCA_004014895.1
GCA_004014965.1
GCA_004014985.1
GCA_004015005.1
GCA_004015025.1
GCA_004015045.1
GCA_004015065.1
GCA_004015085.1
GCA_004015105.1
GCA_004015125.1
GCA_004015145.1
GCA_004015165.1
GCA_004015185.1
GCA_004015205.1
GCA_004015225.1
GCA_004015245.1
GCA_004015265.1
GCA_004015285.1
GCA_004015305.1

GCA_004015325.1
GCA_004015345.1
GCA_004022485.1
GCA_004022505.2
GCA_004022525.1
GCA_004022545.1
GCA_004022565.1
GCA_004023395.2
GCA_004026165.1
GCA_004026185.1
GCA_004027995.1
GCA_004028015.1
GCA_004028235.1
GCA_004028255.1
GCA_004028275.1
GCA_004028295.1
GCA_004028315.1
GCA_004028335.1
GCA_004028355.1
GCA_004028375.1
GCA_004063515.1
GCA_004063635.1
GCA_004063735.1
GCA_004087735.1
GCA_004087915.1
GCA_004087995.1
GCA_004088075.1
GCA_004088155.1
GCA_004088235.1
GCA_004088295.1
GCA_004088395.1
GCA_004089895.1
GCA_004101325.1
GCA_004101345.1
GCA_004101365.1
GCA_004101385.1
GCA_004101405.1
GCA_004101425.1
GCA_004101445.1
GCA_004101465.1
GCA_004101485.1
GCA_004101505.1
GCA_004101525.1
GCA_004101545.1
GCA_004101565.1
GCA_004101585.1
GCA_004101605.1
GCA_004101625.1
GCA_004101645.1
GCA_004101665.1
GCA_004101685.1
GCA_004101705.1
GCA_004101725.1
GCA_004101745.1

GCA_004101785.1
GCA_004101805.1
GCA_004101825.1
GCA_004101845.1
GCA_004101865.1
GCA_004101885.1
GCA_004101925.1
GCA_004101945.1
GCA_004102025.1
GCA_004102045.2
GCA_004102585.1
GCA_004102605.1
GCA_004102645.1
GCA_004102665.1
GCA_004102925.1
GCA_004102945.1
GCA_004103475.1
GCA_004103495.1
GCA_004103515.1
GCA_004103535.1
GCA_004103555.1
GCA_004103575.1
GCA_004103595.1
GCA_004103615.1
GCA_004103635.1
GCA_004103655.1
GCA_004103675.1
GCA_004103695.1
GCA_004103715.1
GCA_004103735.1
GCA_004103755.1
GCA_004103775.1
GCA_004103875.1
GCA_004104415.1
GCA_004104435.1
GCA_004104465.2
GCA_004104485.1
GCA_004104505.1
GCA_004104525.2
GCA_004114395.1
GCA_004114615.1
GCA_004114715.1
GCA_004114735.1
GCA_004114755.1
GCA_004114795.1
GCA_004114895.1
GCA_004114915.1
GCA_004114935.1
GCA_004114955.1
GCA_004114975.1
GCA_004115325.2
GCA_004116955.1
GCA_004116975.1
GCA_004117015.1

GCA_004117055.1
GCA_004117095.1
GCA_004117115.1
GCA_004117135.1
GCA_004118875.1
GCA_004118915.1
GCA_004118935.1
GCA_004118955.1
GCA_004118975.1
GCA_004118995.1
GCA_004119535.1
GCA_004119555.1
GCA_004119575.1
GCA_004119595.1
GCA_004119615.1
GCA_004119635.1
GCA_004119655.1
GCA_004119675.1
GCA_004119695.1
GCA_004119715.1
GCA_004119735.1
GCA_004119755.1
GCA_004119775.1
GCA_004119795.1
GCA_004119815.1
GCA_004119835.1
GCA_004119855.1
GCA_004119875.1
GCA_004119955.1
GCA_004119975.1
GCA_004119995.1
GCA_004120035.1
GCA_004120055.1
GCA_004120075.1
GCA_004120095.1
GCA_004120115.1
GCA_004120135.1
GCA_004120155.1
GCA_004120175.1
GCA_004123675.1
GCA_004123995.1
GCA_004124015.1
GCA_004124035.2
GCA_004124055.2
GCA_004124075.1
GCA_004124095.2
GCA_004124115.1
GCA_004124135.1
GCA_004124155.2
GCA_004124175.2
GCA_004124195.2
GCA_004124215.2
GCA_004124235.1
GCA_004124255.1

GCA_004124275.1
GCA_004124315.2
GCA_004126495.1
GCA_004135085.1
GCA_004135105.1
GCA_004135125.1
GCA_004135145.1
GCA_004135165.1
GCA_004135205.1
GCA_004135285.1
GCA_004135345.1
GCA_004135365.1
GCA_004135385.1
GCA_004135405.1
GCA_004135585.1
GCA_004135735.1
GCA_004135755.1
GCA_004135775.1
GCA_004135795.1
GCA_004135815.1
GCA_004135835.1
GCA_004135855.1
GCA_004135875.1
GCA_004135895.1
GCA_004135915.1
GCA_004135935.1
GCA_004135975.1
GCA_004136035.1
GCA_004136055.1
GCA_004136075.1
GCA_004136095.1
GCA_004136115.1
GCA_004136135.1
GCA_004136155.1
GCA_004136175.1
GCA_004136195.1
GCA_004136215.1
GCA_004136235.1
GCA_004136255.1
GCA_004136295.1
GCA_004136315.1
GCA_004136335.1
GCA_004136355.1
GCA_004136375.1
GCA_004136395.1
GCA_004136415.1
GCA_004136655.1
GCA_004137665.1
GCA_004138605.1
GCA_004139715.1
GCA_004141755.1
GCA_004141835.1
GCA_004141875.1
GCA_004141895.1

GCA_004141955.1
GCA_004141975.1
GCA_004142705.2
GCA_004151605.1
GCA_004153325.1
GCA_004153345.1
GCA_004153365.1
GCA_004153945.1
GCA_004153965.1
GCA_004153985.1
GCA_004154005.1
GCA_004154025.1
GCA_004154045.1
GCA_004154065.1
GCA_004154085.1
GCA_004154105.1
GCA_004154125.1
GCA_004154145.1
GCA_004154165.1
GCA_004154185.1
GCA_004154205.1
GCA_004154225.1
GCA_004154245.1
GCA_004154265.1
GCA_004154285.1
GCA_004154305.1
GCA_004154325.1
GCA_004154345.1
GCA_004154365.1
GCA_004154385.1
GCA_004154405.1
GCA_004154955.1
GCA_004168305.2
GCA_004168325.2
GCA_004168345.2
GCA_004168365.1
GCA_004171185.1
GCA_004171285.1
GCA_004193635.1
GCA_004193655.1
GCA_004193675.1
GCA_004193695.1
GCA_004193715.1
GCA_004193735.1
GCA_004193755.1
GCA_004193875.1
GCA_004193895.1
GCA_004193915.1
GCA_004193935.1
GCA_004193955.1
GCA_004194375.1
GCA_004194515.1
GCA_004194535.1
GCA_004194555.1

GCA_004194605.3
GCA_004194615.2
GCA_004194625.2
GCA_004194635.5
GCA_004208415.1
GCA_004208595.1
GCA_004208615.1
GCA_004208635.1
GCA_004208655.1
GCA_004208675.1
GCA_004208695.1
GCA_004209775.1
GCA_004209795.1
GCA_004209815.1
GCA_004209835.1
GCA_004210215.2
GCA_004210255.1
GCA_004214795.1
GCA_004214815.1
GCA_004214875.1
GCA_004214895.1
GCA_004216475.1
GCA_004216495.1
GCA_004224885.2
GCA_004291075.1
GCA_004291115.2
GCA_004291175.2
GCA_004291255.1
GCA_004293625.1
GCA_004295125.1
GCA_004295305.1
GCA_004295325.1
GCA_004295345.1
GCA_004295365.1
GCA_004295385.1
GCA_004295405.1
GCA_004295485.1
GCA_004295525.1
GCA_004295545.1
GCA_004295565.1
GCA_004295585.1
GCA_004295605.1
GCA_004295625.1
GCA_004295645.1
GCA_004295665.1
GCA_004296255.1
GCA_004296435.1
GCA_004296455.1
GCA_004296475.1
GCA_004296495.1
GCA_004296515.1
GCA_004296535.1
GCA_004299785.2
GCA_004299805.1

GCA_004299845.1
GCA_004300825.1
GCA_004301465.1
GCA_004307315.1
GCA_004319445.1
GCA_004319465.1
GCA_004319485.1
GCA_004319505.1
GCA_004319525.1
GCA_004319565.1
GCA_004319585.1
GCA_004319645.1
GCA_004319665.1
GCA_004322655.1
GCA_004322755.1
GCA_004322775.1
GCA_004322795.1
GCA_004322815.1
GCA_004322835.1
GCA_004322855.1
GCA_004322955.1
GCA_004322975.1
GCA_004322995.1
GCA_004323015.1
GCA_004323035.1
GCA_004323595.1
GCA_004323615.2
GCA_004323635.1
GCA_004323735.1
GCA_004323755.1
GCA_004323775.1
GCA_004323995.2
GCA_004324015.2
GCA_004324025.2
GCA_004324035.2
GCA_004324045.2
GCA_004324095.2
GCA_004324115.2
GCA_004324125.2
GCA_004324135.2
GCA_004324145.2
GCA_004324245.2
GCA_004324275.2
GCA_004324315.2
GCA_004328495.1
GCA_004328515.1
GCA_004328535.1
GCA_004328555.1
GCA_004328575.1
GCA_004328605.1
GCA_004328625.1
GCA_004328665.1
GCA_004328685.1
GCA_004328705.1

GCA_004328725.1
GCA_004328745.1
GCA_004328765.1
GCA_004328785.1
GCA_004328805.1
GCA_004328845.1
GCA_004328865.1
GCA_004328885.1
GCA_004328905.1
GCA_004328925.1
GCA_004328945.1
GCA_004328985.1
GCA_004329055.1
GCA_004331935.1
GCA_004331955.1
GCA_004332015.1
GCA_004332035.1
GCA_004332055.1
GCA_004332075.1
GCA_004332355.1
GCA_004332375.1
GCA_004332535.2
GCA_004332555.2
GCA_004337365.2
GCA_004337595.1
GCA_004337615.1
GCA_004337635.1
GCA_004337655.1
GCA_004337735.2
GCA_004337745.2
GCA_004345025.1
GCA_004345045.1
GCA_004345065.1
GCA_004345205.2
GCA_004346925.1
GCA_004347775.1
GCA_004348055.1
GCA_004348075.1
GCA_004348195.1
GCA_004353225.1
GCA_004353245.1
GCA_004353385.2
GCA_004353425.2
GCA_004353445.2
GCA_004353465.2
GCA_004353525.2
GCA_004353845.1
GCA_004353865.1
GCA_004354225.1
GCA_004354245.1
GCA_004354285.1
GCA_004354345.1
GCA_004354445.2
GCA_004355105.2

GCA_004355125.1
GCA_004355145.1
GCA_004355165.1
GCA_004355185.1
GCA_004355225.1
GCA_004355245.1
GCA_004355265.1
GCA_004355285.1
GCA_004355305.1
GCA_004355325.1
GCA_004355345.1
GCA_004355365.1
GCA_004355385.1
GCA_004355405.1
GCA_004355425.1
GCA_004355445.1
GCA_004355465.1
GCA_004355485.1
GCA_004355505.1
GCA_004355525.1
GCA_004355545.1
GCA_004355565.1
GCA_004355585.1
GCA_004355605.1
GCA_004355705.1
GCA_004355725.1
GCA_004355745.1
GCA_004355765.2
GCA_004355785.1
GCA_004355805.1
GCA_004355825.1
GCA_004355845.1
GCA_004355865.1
GCA_004355885.3
GCA_004358325.1
GCA_004358345.1
GCA_004358365.1
GCA_004358385.1
GCA_004358405.1
GCA_004358425.1
GCA_004358445.1
GCA_004358925.1
GCA_004358965.1
GCA_004358985.1
GCA_004359195.1
GCA_004359355.1
GCA_004359375.1
GCA_004367585.1
GCA_004367665.1
GCA_004367685.1
GCA_004367705.1
GCA_004367725.1
GCA_004367745.1
GCA_004367995.1

GCA_004368015.1
GCA_004368035.1
GCA_004368055.1
GCA_004376055.2
GCA_004377995.2
GCA_004379295.1
GCA_004379315.1
GCA_004379335.1
GCA_004379355.1
GCA_004405205.2
GCA_004421005.1
GCA_004421025.1
GCA_004421045.1
GCA_004421065.1
GCA_004421085.1
GCA_004421105.1
GCA_004519465.1
GCA_004519515.1
GCA_004521945.2
GCA_004524875.1
GCA_004526235.1
GCA_004526255.1
GCA_004526275.1
GCA_004526325.1
GCA_004526345.1
GCA_004526365.1
GCA_004535885.1
GCA_004551575.1
GCA_004551615.1
GCA_004551645.1
GCA_004551665.1
GCA_004563595.2
GCA_004563755.2
GCA_004564035.1
GCA_004564075.1
GCA_004564095.1
GCA_004564115.1
GCA_004564135.1
GCA_004564155.1
GCA_004564175.1
GCA_004564195.1
GCA_004564215.1
GCA_004564355.1
GCA_004564375.1
GCA_004571175.1
GCA_004571195.1
GCA_004614295.1
GCA_004614315.1
GCA_004634385.1
GCA_004634405.1
GCA_004634505.1
GCA_004634525.1
GCA_004634545.1
GCA_004634565.1

GCA_004634585.1
GCA_004634605.1
GCA_004634625.1
GCA_004634645.1
GCA_004634665.1
GCA_004634685.1
GCA_004634705.1
GCA_004634725.1
GCA_004634745.1
GCA_004634765.1
GCA_004634785.1
GCA_004634805.1
GCA_004634825.1
GCA_004634845.1
GCA_004634865.1
GCA_004634885.1
GCA_004634905.1
GCA_004634925.1
GCA_004635085.1
GCA_004635105.1
GCA_004635125.1
GCA_004635145.1
GCA_004635165.1
GCA_004635185.1
GCA_004635205.1
GCA_004635225.1
GCA_004635245.1
GCA_004635265.1
GCA_004635285.1
GCA_004635305.1
GCA_004635325.1
GCA_004635345.1
GCA_004635365.1
GCA_004635385.1
GCA_004635405.1
GCA_004635425.1
GCA_004635445.1
GCA_004635465.1
GCA_004635485.1
GCA_004635505.1
GCA_004635525.1
GCA_004635545.1
GCA_004635565.1
GCA_004635585.1
GCA_004635605.1
GCA_004635625.1
GCA_004635645.1
GCA_004635665.1
GCA_004635685.1
GCA_004635705.1
GCA_004635725.1
GCA_004635745.1
GCA_004635765.1
GCA_004635785.1

GCA_004635805.1
GCA_004635825.1
GCA_004635845.1
GCA_004635865.1
GCA_004635885.1
GCA_004635905.1
GCA_004635925.1
GCA_004635945.1
GCA_004635965.1
GCA_004635985.1
GCA_004636005.1
GCA_004636025.1
GCA_004636045.1
GCA_004636065.1
GCA_004636085.1
GCA_004636105.1
GCA_004636125.1
GCA_004636145.1
GCA_004636165.1
GCA_004636185.1
GCA_004636205.1
GCA_004636225.1
GCA_004636245.1
GCA_004636265.1
GCA_004636285.1
GCA_004636305.1
GCA_004636325.1
GCA_004636345.1
GCA_004636365.1
GCA_004636385.1
GCA_004636405.1
GCA_004636425.1
GCA_004636445.1
GCA_004636465.1
GCA_004636485.1
GCA_004636505.1
GCA_004636525.1
GCA_004636545.1
GCA_004636565.1
GCA_004636605.2
GCA_004636665.2
GCA_004636745.2
GCA_004636785.1
GCA_004636805.1
GCA_004636825.1
GCA_004636845.1
GCA_004636865.1
GCA_004636885.1
GCA_004636905.1
GCA_004636925.1
GCA_004636945.1
GCA_004636965.1
GCA_004636985.1
GCA_004637005.1

GCA_004637025.1
GCA_004637045.1
GCA_004637065.1
GCA_004637085.1
GCA_004637105.1
GCA_004637125.1
GCA_004637145.1
GCA_004637165.1
GCA_004637185.1
GCA_004637205.1
GCA_004637225.1
GCA_004637245.1
GCA_004637265.1
GCA_004683865.2
GCA_004683885.1
GCA_004683905.1
GCA_004683965.1
GCA_004684245.1
GCA_004684265.1
GCA_004684285.1
GCA_004684305.1
GCA_004684325.1
GCA_004684345.1
GCA_004684365.1
GCA_004730965.1
GCA_004751985.1
GCA_004752005.1
GCA_004758745.1
GCA_004758865.1
GCA_004759025.1
GCA_004763665.2
GCA_004766445.2
GCA_004768525.1
GCA_004768545.1
GCA_004768565.1
GCA_004768585.1
GCA_004768605.1
GCA_004768625.1
GCA_004768645.1
GCA_004768665.1
GCA_004768685.1
GCA_004768705.1
GCA_004768725.1
GCA_004768745.1
GCA_004771055.1
GCA_004771075.1
GCA_004771095.1
GCA_004771115.1
GCA_004771135.1
GCA_004771155.1
GCA_004771175.1
GCA_004771195.1
GCA_004771235.1
GCA_004771295.1

GCA_004771335.1
GCA_004771435.1
GCA_004771515.1
GCA_004771975.1
GCA_004772055.1
GCA_004772175.1
GCA_004772215.1
GCA_004777465.1
GCA_004785625.2
GCA_004785995.1
GCA_004786015.1
GCA_004786035.1
GCA_004792395.1
GCA_004792415.1
GCA_004792435.1
GCA_004792455.1
GCA_004792475.1
GCA_004792495.1
GCA_004792515.1
GCA_004792535.1
GCA_004792555.1
GCA_004792575.1
GCA_004792595.1
GCA_004792615.1
GCA_004792635.1
GCA_004794295.1
GCA_004795715.1
GCA_004795735.1
GCA_004795775.1
GCA_004795795.1
GCA_004795815.1
GCA_004795835.1
GCA_004795855.1
GCA_004795875.1
GCA_004795895.1
GCA_004795915.1
GCA_004795935.1
GCA_004795955.1
GCA_004795975.1
GCA_004797125.1
GCA_004797155.2
GCA_004797195.1
GCA_004798665.1
GCA_004798685.1
GCA_004798705.1
GCA_004798725.1
GCA_004798745.1
GCA_004798765.1
GCA_004799565.1
GCA_004799585.1
GCA_004799705.1
GCA_004801195.1
GCA_004801235.1
GCA_004801255.1

GCA_004801555.1
GCA_004801575.1
GCA_004802635.2
GCA_004803715.2
GCA_004803755.1
GCA_004803775.1
GCA_004803795.1
GCA_004803815.1
GCA_004803835.1
GCA_004803855.2
GCA_004803875.1
GCA_004803895.1
GCA_004804185.1
GCA_004804205.1
GCA_004804375.1
GCA_004804395.1
GCA_004842085.1
GCA_004843165.1
GCA_004843345.1
GCA_004843545.1
GCA_004843685.1
GCA_004843725.1
GCA_004847005.1
GCA_004847145.1
GCA_004847345.1
GCA_004847505.1
GCA_004847525.1
GCA_004847545.1
GCA_004847565.1
GCA_004847585.1
GCA_004847605.1
GCA_004847625.1
GCA_004847745.1
GCA_004847885.1
GCA_004848145.1
GCA_004848405.1
GCA_004848545.1
GCA_004848685.1
GCA_004848885.1
GCA_004851605.1
GCA_004916735.1
GCA_004919145.1
GCA_004919165.1
GCA_004919185.1
GCA_004919205.1
GCA_004919225.1
GCA_004919265.1
GCA_004919285.1
GCA_004919305.1
GCA_004919325.1
GCA_004919345.1
GCA_004919365.1
GCA_004919385.1
GCA_004919405.1

GCA_004919425.1
GCA_004919445.1
GCA_004919465.1
GCA_004919485.1
GCA_004919535.4
GCA_004923295.1
GCA_004923315.1
GCA_004923395.1
GCA_004923415.2
GCA_004924255.1
GCA_004924275.1
GCA_004924295.1
GCA_004924315.1
GCA_004924335.1
GCA_004924355.1
GCA_004936435.1
GCA_004941405.1
GCA_004941425.1
GCA_005014055.1
GCA_005014075.1
GCA_005037715.2
GCA_005037725.2
GCA_005037735.2
GCA_005037775.2
GCA_005037795.2
GCA_005037805.2
GCA_005037815.2
GCA_005037845.2
GCA_005080685.1
GCA_005080705.1
GCA_005080865.1
GCA_005080965.1
GCA_005081445.1
GCA_005082145.1
GCA_005082585.1
GCA_005083845.1
GCA_005083985.2
GCA_005094845.1
GCA_005116655.2
GCA_005116675.2
GCA_005121165.3
GCA_005144405.2
GCA_005144425.2
GCA_005144435.2
GCA_005144495.2
GCA_005144505.2
GCA_005144635.2
GCA_005144885.1
GCA_005144905.1
GCA_005144925.1
GCA_005144945.1
GCA_005144965.1
GCA_005144985.1
GCA_005145005.1

GCA_005145025.1
GCA_005145045.1
GCA_005145085.1
GCA_005153485.1
GCA_005154805.1
GCA_005155025.1
GCA_005155285.1
GCA_005155785.1
GCA_005155965.1
GCA_005156105.1
GCA_005156265.1
GCA_005156885.1
GCA_005157325.1
GCA_005158025.1
GCA_005158285.1
GCA_005160065.1
GCA_005160365.1
GCA_005160405.1
GCA_005160425.1
GCA_005160445.1
GCA_005160565.1
GCA_005160785.1
GCA_005160925.1
GCA_005161125.1
GCA_005161325.1
GCA_005161465.1
GCA_005161605.1
GCA_005161825.1
GCA_005161985.1
GCA_005162245.1
GCA_005162405.1
GCA_005162565.1
GCA_005162725.1
GCA_005162865.1
GCA_005163085.1
GCA_005163245.1
GCA_005163385.1
GCA_005163565.1
GCA_005163705.1
GCA_005163865.1
GCA_005164025.1
GCA_005164305.1
GCA_005164585.1
GCA_005164705.1
GCA_005164885.1
GCA_005165045.1
GCA_005165265.1
GCA_005165385.1
GCA_005165545.1
GCA_005166025.1
GCA_005166225.1
GCA_005166365.1
GCA_005217135.2
GCA_005217155.2

GCA_005221285.1
GCA_005221305.1
GCA_005221325.1
GCA_005221345.1
GCA_005221365.1
GCA_005221385.1
GCA_005221405.1
GCA_005221425.1
GCA_005221485.1
GCA_005221505.1
GCA_005221525.1
GCA_005221545.1
GCA_005221565.1
GCA_005221585.1
GCA_005221605.1
GCA_005221625.1
GCA_005221645.1
GCA_005221665.1
GCA_005221685.1
GCA_005221705.1
GCA_005221725.1
GCA_005221745.1
GCA_005221765.1
GCA_005221785.1
GCA_005221805.1
GCA_005221825.1
GCA_005221845.1
GCA_005221865.1
GCA_005221885.1
GCA_005221905.1
GCA_005221925.1
GCA_005221945.1
GCA_005221965.1
GCA_005221985.1
GCA_005222005.1
GCA_005222025.1
GCA_005222045.1
GCA_005222065.1
GCA_005222125.1
GCA_005222145.1
GCA_005222165.1
GCA_005222205.1
GCA_005222225.1
GCA_005222245.1
GCA_005222265.1
GCA_005222285.1
GCA_005222305.1
GCA_005222325.1
GCA_005222345.1
GCA_005222365.1
GCA_005222385.1
GCA_005222405.1
GCA_005222425.1
GCA_005222445.1

GCA_005222465.1
GCA_005222485.1
GCA_005222505.1
GCA_005222545.1
GCA_005222565.1
GCA_005222585.1
GCA_005222625.1
GCA_005234075.1
GCA_005234095.1
GCA_005234115.1
GCA_005234135.1
GCA_005237295.1
GCA_005280255.1
GCA_005280275.1
GCA_005280295.1
GCA_005280315.1
GCA_005280355.1
GCA_005280375.1
GCA_005280395.1
GCA_005280415.1
GCA_005280435.1
GCA_005280655.1
GCA_005280675.1
GCA_005280695.1
GCA_005280715.1
GCA_005280735.1
GCA_005280755.1
GCA_005281435.1
GCA_005281455.1
GCA_005281475.1
GCA_005304985.1
GCA_005305005.1
GCA_005311005.1
GCA_005311585.1
GCA_005341445.1
GCA_005341465.1
GCA_005347485.1
GCA_005347505.1
GCA_005377625.1
GCA_005383105.2
GCA_005389605.2
GCA_005391285.2
GCA_005393605.2
GCA_005396225.2
GCA_005406185.3
GCA_005406285.1
GCA_005406305.1
GCA_005457265.2
GCA_005473905.2
GCA_005484365.1
GCA_005484525.1
GCA_005484585.1
GCA_005484805.1
GCA_005484945.1

GCA_005484965.1
GCA_005484985.1
GCA_005489985.1
GCA_005490785.1
GCA_005490965.1
GCA_005490985.1
GCA_005491085.2
GCA_005491425.1
GCA_005517195.1
GCA_005517315.1
GCA_005517455.1
GCA_005518015.1
GCA_005518035.1
GCA_005518055.1
GCA_005518095.1
GCA_005518115.1
GCA_005519135.2
GCA_005519305.1
GCA_005519465.1
GCA_005537135.1
GCA_005537375.1
GCA_005549115.1
GCA_005576555.1
GCA_005576575.1
GCA_005576595.1
GCA_005576735.1
GCA_005576935.1
GCA_005577315.1
GCA_005577435.1
GCA_005601135.1
GCA_005670685.2
GCA_005671335.1
GCA_005671355.1
GCA_005671375.1
GCA_005671395.1
GCA_005696555.1
GCA_005696695.1
GCA_005696855.1
GCA_005697055.1
GCA_005697215.1
GCA_005697395.1
GCA_005697565.1
GCA_005705815.1
GCA_005706215.1
GCA_005706435.1
GCA_005706655.1
GCA_005707015.1
GCA_005707215.1
GCA_005707335.1
GCA_005707595.1
GCA_005843885.1
GCA_005843965.1
GCA_005843985.1
GCA_005844025.1

GCA_005844045.1
GCA_005844065.1
GCA_005845345.1
GCA_005845365.1
GCA_005845385.1
GCA_005848555.1
GCA_005848825.1
GCA_005848985.1
GCA_005849145.1
GCA_005849285.1
GCA_005849435.1
GCA_005853645.1
GCA_005853785.1
GCA_005854025.1
GCA_005854245.1
GCA_005860785.2
GCA_005860795.2
GCA_005860925.2
GCA_005862185.2
GCA_005862305.2
GCA_005862325.1
GCA_005877035.1
GCA_005877055.1
GCA_005885775.1
GCA_005885795.1
GCA_005885815.1
GCA_005885835.1
GCA_005885855.1
GCA_005885875.1
GCA_005885895.1
GCA_005885915.1
GCA_005885935.1
GCA_005885955.1
GCA_005885975.1
GCA_005886035.1
GCA_005886055.1
GCA_005886075.1
GCA_005886215.1
GCA_005886435.1
GCA_005886545.1
GCA_005886655.1
GCA_005886715.1
GCA_005886735.1
GCA_005886755.1
GCA_005886805.2
GCA_005887635.2
GCA_005889955.1
GCA_005889975.1
GCA_005889995.1
GCA_005890015.1
GCA_005890035.1
GCA_005890075.1
GCA_005890095.1
GCA_005890115.1

GCA_005890135.1
GCA_005890155.1
GCA_005890175.1
GCA_005930995.1
GCA_005931015.2
GCA_005931035.2
GCA_005931055.2
GCA_005931095.1
GCA_005937795.2
GCA_005937845.2
GCA_005937855.2
GCA_005937885.2
GCA_005937895.2
GCA_005937905.2
GCA_005937945.2
GCA_005937985.2
GCA_005937995.2
GCA_005938645.2
GCA_005938665.2
GCA_005943945.1
GCA_005944105.1
GCA_005944305.1
GCA_005952805.1
GCA_005952885.1
GCA_005954605.1
GCA_005954625.1
GCA_005954645.2
GCA_005954665.1
GCA_005954685.1
GCA_005954705.1
GCA_005954725.1
GCA_005960665.1
GCA_005979285.1
GCA_005979305.1
GCA_006007905.1
GCA_006007925.1
GCA_006007945.1
GCA_006007965.1
GCA_006016075.1
GCA_006051015.2
GCA_006064855.1
GCA_006065115.1
GCA_006065315.1
GCA_006088715.1
GCA_006088735.1
GCA_006088755.1
GCA_006088795.1
GCA_006088815.1
GCA_006088835.1
GCA_006088855.1
GCA_006088875.1
GCA_006094275.1
GCA_006094295.1
GCA_006094315.1

GCA_006094335.1
GCA_006094355.1
GCA_006094375.1
GCA_006094395.1
GCA_006094415.1
GCA_006094435.1
GCA_006094455.1
GCA_006094475.1
GCA_006094495.1
GCA_006094695.1
GCA_006094915.1
GCA_006113225.2
GCA_006149185.1
GCA_006151905.1
GCA_006151925.1
GCA_006158985.1
GCA_006159205.1
GCA_006165085.1
GCA_006165105.1
GCA_006165125.1
GCA_006165145.1
GCA_006165165.1
GCA_006165185.1
GCA_006165205.1
GCA_006165225.1
GCA_006165245.1
GCA_006165265.1
GCA_006165285.1
GCA_006228165.1
GCA_006228185.1
GCA_006228205.1
GCA_006228225.1
GCA_006228245.1
GCA_006228265.1
GCA_006228285.1
GCA_006228305.1
GCA_006228325.1
GCA_006228345.1
GCA_006228385.1
GCA_006228425.1
GCA_006228445.1
GCA_006228465.1
GCA_006228485.1
GCA_006228505.1
GCA_006228525.1
GCA_006228545.1
GCA_006228565.1
GCA_006229535.1
GCA_006232065.1
GCA_006232125.1
GCA_006274235.1
GCA_006274405.1
GCA_006280355.1
GCA_006304565.1

GCA_006337025.1
GCA_006337045.1
GCA_006337065.1
GCA_006337085.1
GCA_006337105.1
GCA_006337125.1
GCA_006337145.1
GCA_006337165.1
GCA_006337265.1
GCA_006337285.1
GCA_006337305.1
GCA_006337325.1
GCA_006337345.1
GCA_006337365.1
GCA_006337385.1
GCA_006337405.1
GCA_006337425.1
GCA_006337445.1
GCA_006337465.1
GCA_006337485.1
GCA_006337625.1
GCA_006337745.1
GCA_006337875.1
GCA_006338045.1
GCA_006338145.1
GCA_006338285.1
GCA_006338415.1
GCA_006338545.1
GCA_006338685.1
GCA_006338815.1
GCA_006338945.1
GCA_006339065.1
GCA_006339235.1
GCA_006339365.1
GCA_006349345.1
GCA_006349365.1
GCA_006349715.2
GCA_006349735.2
GCA_006351725.3
GCA_006351785.1
GCA_006351805.1
GCA_006351845.1
GCA_006351865.1
GCA_006351885.1
GCA_006351905.1
GCA_006351925.1
GCA_006351945.1
GCA_006351965.1
GCA_006351985.2
GCA_006352025.1
GCA_006352065.1
GCA_006352265.1
GCA_006363875.1
GCA_006364035.1

GCA_006364235.1
GCA_006364295.1
GCA_006364355.1
GCA_006364635.1
GCA_006364655.1
GCA_006364675.1
GCA_006364695.1
GCA_006364735.1
GCA_006364755.1
GCA_006364775.1
GCA_006364795.1
GCA_006364815.1
GCA_006365335.1
GCA_006378125.1
GCA_006378235.1
GCA_006384195.1
GCA_006384875.1
GCA_006384935.1
GCA_006384955.1
GCA_006384975.1
GCA_006385015.1
GCA_006385075.1
GCA_006385095.1
GCA_006385115.1
GCA_006385135.1
GCA_006385555.1
GCA_006385575.1
GCA_006385595.1
GCA_006385615.1
GCA_006385635.1
GCA_006385655.1
GCA_006385735.2
GCA_006385915.1
GCA_006385935.1
GCA_006400955.1
GCA_006401215.1
GCA_006401635.1
GCA_006402015.1
GCA_006402415.1
GCA_006402735.1
GCA_006402875.1
GCA_006439395.2
GCA_006440675.2
GCA_006442545.2
GCA_006442795.2
GCA_006442975.2
GCA_006459125.1
GCA_006459145.1
GCA_006459165.1
GCA_006461325.2
GCA_006461415.2
GCA_006489235.1
GCA_006489255.1
GCA_006489275.1

GCA_006494715.1
GCA_006494755.1
GCA_006494775.1
GCA_006494795.1
GCA_006494815.1
GCA_006494835.1
GCA_006494855.1
GCA_006494875.1
GCA_006494895.1
GCA_006494915.1
GCA_006496635.1
GCA_006516935.1
GCA_006516995.1
GCA_006517015.1
GCA_006517035.1
GCA_006517055.1
GCA_006517075.1
GCA_006517095.1
GCA_006517115.1
GCA_006517135.1
GCA_006517155.1
GCA_006517195.1
GCA_006517215.1
GCA_006517235.1
GCA_006517255.1
GCA_006517275.1
GCA_006517775.1
GCA_006517795.1
GCA_006517815.1
GCA_006536085.3
GCA_006537505.3
GCA_006542275.1
GCA_006542295.1
GCA_006542315.2
GCA_006542335.1
GCA_006542355.1
GCA_006542375.1
GCA_006542395.2
GCA_006542605.1
GCA_006542645.1
GCA_006542665.1
GCA_006542685.1
GCA_006542705.1
GCA_006542725.1
GCA_006542745.1
GCA_006546965.1
GCA_006547005.1
GCA_006547025.1
GCA_006547045.1
GCA_006547065.1
GCA_006575625.1
GCA_006575665.1
GCA_006704185.1
GCA_006704205.1

GCA_006704595.2
GCA_006711125.1
GCA_006711145.1
GCA_006711405.1
GCA_006711525.1
GCA_006711645.1
GCA_006716135.1
GCA_006716245.1
GCA_006716385.1
GCA_006716515.1
GCA_006716625.1
GCA_006716825.1
GCA_006738645.1
GCA_006739205.1
GCA_006739385.1
GCA_006739545.1
GCA_006740045.1
GCA_006740305.1
GCA_006740645.2
GCA_006740705.1
GCA_006740725.1
GCA_006740745.1
GCA_006740765.1
GCA_006741185.1
GCA_006741355.1
GCA_006741505.1
GCA_006741705.1
GCA_006741845.1
GCA_006742065.1
GCA_006742205.1
GCA_006742345.1
GCA_006742565.1
GCA_006742785.1
GCA_006757745.1
GCA_006766145.1
GCA_006770265.1
GCA_006770285.1
GCA_006770305.1
GCA_006770325.1
GCA_006770345.1
GCA_006770365.1
GCA_006770385.1
GCA_006770405.1
GCA_006770425.1
GCA_006770445.1
GCA_006770465.1
GCA_006770485.1
GCA_006770505.1
GCA_006770685.1
GCA_006770865.1
GCA_006771025.1
GCA_006777025.1
GCA_006777045.1
GCA_006777065.1

GCA_006777085.1
GCA_006777105.1
GCA_006842785.1
GCA_006843305.1
GCA_006874565.1
GCA_006874605.1
GCA_006874625.1
GCA_006874645.1
GCA_006874665.1
GCA_006874705.1
GCA_006874725.1
GCA_006874745.1
GCA_006874765.1
GCA_006874785.1
GCA_006874805.1
GCA_006965405.3
GCA_006965425.1
GCA_006965445.1
GCA_006965465.1
GCA_006965485.1
GCA_006965505.1
GCA_006965525.1
GCA_006965545.2
GCA_006965565.1
GCA_006969105.1
GCA_006969245.1
GCA_006970065.1
GCA_006970285.1
GCA_006970545.1
GCA_006970665.1
GCA_006970865.1
GCA_006971065.1
GCA_006971245.1
GCA_006971425.1
GCA_006971625.1
GCA_006971785.1
GCA_006974005.1
GCA_006974025.1
GCA_006974065.1
GCA_006974085.1
GCA_006974105.1
GCA_006974125.1
GCA_006974145.1
GCA_006974165.1
GCA_006974185.1
GCA_006974205.1
GCA_007000325.2
GCA_007004545.3
GCA_007005445.1
GCA_007005625.1
GCA_007012305.1
GCA_007012325.1
GCA_007035645.1
GCA_007035805.1

GCA_007035975.1
GCA_007036205.1
GCA_007106385.1
GCA_007106565.1
GCA_007106885.1
GCA_007107025.1
GCA_007107165.1
GCA_007107365.1
GCA_007107525.1
GCA_007113405.2
GCA_007113425.2
GCA_007113505.2
GCA_007113525.2
GCA_007113665.2
GCA_007163885.2
GCA_007164525.1
GCA_007164725.1
GCA_007165405.1
GCA_007165645.1
GCA_007179295.1
GCA_007182835.1
GCA_007182855.1
GCA_007182875.1
GCA_007182895.1
GCA_007183175.1
GCA_007185035.1
GCA_007197555.1
GCA_007197575.1
GCA_007197595.1
GCA_007197715.1
GCA_007197735.1
GCA_007197755.1
GCA_007197775.1
GCA_007197795.1
GCA_007197815.1
GCA_007197835.1
GCA_007221455.1
GCA_007289895.1
GCA_007290395.1
GCA_007292115.1
GCA_007301515.1
GCA_007361795.1
GCA_007361935.1
GCA_007362135.1
GCA_007362295.1
GCA_007362475.1
GCA_007362635.1
GCA_007362775.1
GCA_007363175.1
GCA_007363395.1
GCA_007410625.1
GCA_007421865.2
GCA_007421875.2
GCA_007421885.2

GCA_007430945.1
GCA_007431185.1
GCA_007431345.1
GCA_007431545.1
GCA_007431745.1
GCA_007475365.1
GCA_007475525.1
GCA_007556795.1
GCA_007556815.1
GCA_007556835.1
GCA_007558985.1
GCA_007559005.1
GCA_007559025.1
GCA_007559065.1
GCA_007559085.1
GCA_007559105.1
GCA_007559125.1
GCA_007567505.1
GCA_007567665.1
GCA_007632255.1
GCA_007632435.1
GCA_007633215.1
GCA_007633355.1
GCA_007637635.1
GCA_007641235.1
GCA_007641255.1
GCA_007647215.1
GCA_007723545.1
GCA_007723825.1
GCA_007724025.1
GCA_007724205.1
GCA_007724385.1
GCA_007724625.1
GCA_007724845.1
GCA_007725005.1
GCA_007725185.1
GCA_007726325.1
GCA_007726465.1
GCA_007726495.1
GCA_007726525.1
GCA_007726545.1
GCA_007726565.1
GCA_007741495.1
GCA_007741515.1
GCA_007741535.1
GCA_007741555.1
GCA_007747015.1
GCA_007747215.1
GCA_007747445.1
GCA_007747655.1
GCA_007747795.1
GCA_007747995.1
GCA_007748015.1
GCA_007748035.1

GCA_007748055.1
GCA_007748075.1
GCA_007748095.1
GCA_007748115.1
GCA_007750395.1
GCA_007750655.1
GCA_007750855.1
GCA_007751035.1
GCA_007751255.1
GCA_007751475.1
GCA_007751715.1
GCA_007751945.1
GCA_007752135.1
GCA_007752345.1
GCA_007753455.1
GCA_007753915.1
GCA_007794935.1
GCA_007795095.1
GCA_007813675.1
GCA_007813835.1
GCA_007814015.1
GCA_007814115.1
GCA_007814275.1
GCA_007814385.1
GCA_007814525.1
GCA_007814625.1
GCA_007814725.1
GCA_007814825.1
GCA_007814955.1
GCA_007826355.1
GCA_007827165.1
GCA_007833125.1
GCA_007833215.1
GCA_007833235.1
GCA_007833255.1
GCA_007833295.1
GCA_007833315.1
GCA_007833335.1
GCA_007833375.1
GCA_007833395.1
GCA_007833415.1
GCA_007833435.1
GCA_007833455.1
GCA_007833475.1
GCA_007833495.1
GCA_007833515.1
GCA_007833535.1
GCA_007833555.1
GCA_007833575.1
GCA_007833595.1
GCA_007833615.1
GCA_007833635.1
GCA_007833655.1
GCA_007833695.1

GCA_007833715.1
GCA_007833735.1
GCA_007833755.1
GCA_007833775.1
GCA_007833795.1
GCA_007833815.1
GCA_007833855.1
GCA_007833875.1
GCA_007833895.1
GCA_007833915.1
GCA_007833935.1
GCA_007833955.1
GCA_007836735.1
GCA_007836855.1
GCA_007843535.1
GCA_007843715.1
GCA_007843915.1
GCA_007844035.1
GCA_007844055.1
GCA_007844075.1
GCA_007855275.1
GCA_007855455.1
GCA_007855645.1
GCA_007855915.1
GCA_007856155.1
GCA_007858415.1
GCA_007858495.1
GCA_007858515.1
GCA_007858535.1
GCA_007858555.1
GCA_007858575.1
GCA_007858975.2
GCA_007859575.1
GCA_007859615.1
GCA_007859635.1
GCA_007859655.1
GCA_007859675.1
GCA_007873865.1
GCA_007874125.1
GCA_007904085.1
GCA_007904105.1
GCA_007917035.3
GCA_007917315.3
GCA_007922615.2
GCA_007922635.1
GCA_007922655.1
GCA_007923045.4
GCA_007923125.1
GCA_007923145.1
GCA_007923165.1
GCA_007923185.1
GCA_007923205.1
GCA_007923785.2
GCA_007923865.2

GCA_007923885.2
GCA_007923905.2
GCA_007923925.2
GCA_007923945.2
GCA_007923965.2
GCA_007923985.1
GCA_007954215.2
GCA_007954405.1
GCA_007954425.1
GCA_007954445.1
GCA_007954465.1
GCA_007954485.1
GCA_007954525.1
GCA_007954565.1
GCA_007954585.1
GCA_007954605.1
GCA_007954625.1
GCA_007954645.1
GCA_007954665.1
GCA_007954685.1
GCA_007954705.1
GCA_007954725.1
GCA_007954765.1
GCA_007954785.1
GCA_007954925.2
GCA_007955015.2
GCA_007955105.2
GCA_007963885.1
GCA_007964065.1
GCA_007964225.1
GCA_007964465.1
GCA_007964685.1
GCA_007964805.1
GCA_007964925.1
GCA_007965105.1
GCA_007965355.1
GCA_007965585.1
GCA_007965745.1
GCA_007965905.1
GCA_007966105.1
GCA_007970185.1
GCA_007970465.1
GCA_007970665.1
GCA_007970805.1
GCA_007971025.1
GCA_007971225.1
GCA_007971385.1
GCA_007971525.1
GCA_007971685.1
GCA_007972595.1
GCA_007972745.1
GCA_007973265.1
GCA_007973365.1
GCA_007973645.1

GCA_007973825.1
GCA_007974025.1
GCA_007974045.1
GCA_007974065.1
GCA_007974085.1
GCA_007974105.1
GCA_007974125.1
GCA_007974145.1
GCA_007974165.1
GCA_007974375.1
GCA_007974525.1
GCA_007974745.1
GCA_007974925.1
GCA_007975085.1
GCA_007975225.1
GCA_007975365.1
GCA_007975575.1
GCA_007975765.1
GCA_007976025.1
GCA_007976245.1
GCA_007976445.1
GCA_007976625.1
GCA_007976765.1
GCA_007976905.1
GCA_007977085.1
GCA_007977225.1
GCA_007977495.1
GCA_007977645.1
GCA_007977825.1
GCA_007978045.1
GCA_007978185.1
GCA_007978365.1
GCA_007978505.1
GCA_007978725.1
GCA_007978905.1
GCA_007979065.1
GCA_007979225.1
GCA_007979365.1
GCA_007979525.1
GCA_007979665.1
GCA_007979905.1
GCA_007980045.1
GCA_007980145.1
GCA_007980285.1
GCA_007980445.1
GCA_007980645.1
GCA_007980785.1
GCA_007980905.1
GCA_007980965.1
GCA_007980985.1
GCA_007981005.1
GCA_007981025.1
GCA_007981045.1
GCA_007981265.1

GCA_007989425.1
GCA_007990365.1
GCA_007990385.1
GCA_007990405.1
GCA_007990425.1
GCA_007990445.1
GCA_007990505.1
GCA_007990525.1
GCA_007990545.2
GCA_007990575.1
GCA_007990635.1
GCA_007990655.1
GCA_007993715.1
GCA_007993755.1
GCA_007993775.1
GCA_007993795.1
GCA_007995155.1
GCA_007995175.1
GCA_007997855.1
GCA_007998005.1
GCA_007998065.1
GCA_007998085.1
GCA_007998105.1
GCA_007998125.1
GCA_007998145.1
GCA_007998985.1
GCA_007999005.1
GCA_008000755.1
GCA_008000795.1
GCA_008000815.1
GCA_008000835.1
GCA_008000855.1
GCA_008000875.1
GCA_008000895.1
GCA_008000915.1
GCA_008000935.1
GCA_008000975.1
GCA_008000995.1
GCA_008001015.1
GCA_008001035.1
GCA_008001235.1
GCA_008033115.1
GCA_008033135.1
GCA_008033155.1
GCA_008033175.1
GCA_008033195.1
GCA_008033215.1
GCA_008033235.1
GCA_008033255.2
GCA_008033295.1
GCA_008033315.1
GCA_008033725.1
GCA_008033745.1
GCA_008033765.1

GCA_008041895.1
GCA_008041975.1
GCA_008041995.1
GCA_008042015.2
GCA_008064855.1
GCA_008064875.1
GCA_008064895.1
GCA_008064915.1
GCA_008064995.1
GCA_008065015.2
GCA_008065095.1
GCA_008065135.1
GCA_008065155.1
GCA_008065415.1
GCA_008065435.1
GCA_008065455.1
GCA_008080655.1
GCA_008083195.1
GCA_008085695.1
GCA_008085715.1
GCA_008085735.1
GCA_008085755.1
GCA_008085775.1
GCA_008085795.1
GCA_008085815.1
GCA_008085835.1
GCA_008085855.1
GCA_008085875.1
GCA_008086305.1
GCA_008086325.1
GCA_008086345.1
GCA_008086365.1
GCA_008086385.1
GCA_008086405.1
GCA_008086425.1
GCA_008086465.1
GCA_008086545.1
GCA_008087605.1
GCA_008087625.1
GCA_008087665.1
GCA_008118325.1
GCA_008118345.1
GCA_008118365.1
GCA_008119665.1
GCA_008121475.1
GCA_008121495.1
GCA_008121515.1
GCA_008121535.1
GCA_008122265.1
GCA_008122285.1
GCA_008122305.1
GCA_008123985.1
GCA_008124005.1
GCA_008124025.1

GCA_008124045.1
GCA_008124065.1
GCA_008124085.1
GCA_008124105.1
GCA_008124125.1
GCA_008124145.1
GCA_008124165.1
GCA_008124185.1
GCA_008124205.1
GCA_008124225.1
GCA_008124245.1
GCA_008124265.1
GCA_008124285.1
GCA_008124305.1
GCA_008124325.1
GCA_008124345.1
GCA_008124365.1
GCA_008124385.1
GCA_008124405.1
GCA_008124425.1
GCA_008151785.1
GCA_008152105.1
GCA_008152325.1
GCA_008152505.1
GCA_008152825.1
GCA_008153055.1
GCA_008153205.1
GCA_008153345.1
GCA_008180305.1
GCA_008195485.1
GCA_008195605.1
GCA_008195825.1
GCA_008244705.1
GCA_008244725.1
GCA_008244745.1
GCA_008244765.1
GCA_008244785.1
GCA_008244805.1
GCA_008244825.1
GCA_008244845.1
GCA_008244865.1
GCA_008244885.1
GCA_008244905.1
GCA_008244925.1
GCA_008245005.1
GCA_008245025.1
GCA_008245045.1
GCA_008245065.1
GCA_008245105.1
GCA_008245125.1
GCA_008245145.1
GCA_008245165.1
GCA_008245185.1
GCA_008247605.1

GCA_008247765.1
GCA_008247925.1
GCA_008248145.1
GCA_008253725.1
GCA_008253865.1
GCA_008254045.1
GCA_008271365.1
GCA_008271385.1
GCA_008271405.2
GCA_008271425.1
GCA_008274805.1
GCA_008274825.1
GCA_008274845.1
GCA_008274865.1
GCA_008274885.1
GCA_008274905.1
GCA_008274925.1
GCA_008274945.1
GCA_008274965.1
GCA_008275005.1
GCA_008275025.1
GCA_008275045.1
GCA_008275065.1
GCA_008275085.1
GCA_008275105.1
GCA_008275125.1
GCA_008281175.1
GCA_008298035.1
GCA_008305695.1
GCA_008306195.1
GCA_008313815.1
GCA_008326525.1
GCA_008327825.1
GCA_008329645.1
GCA_008329665.1
GCA_008329765.1
GCA_008329785.1
GCA_008329805.1
GCA_008329845.1
GCA_008329865.1
GCA_008329885.1
GCA_008329905.1
GCA_008329925.1
GCA_008329945.1
GCA_008329965.1
GCA_008329985.2
GCA_008330005.1
GCA_008330025.1
GCA_008330045.1
GCA_008330065.1
GCA_008330085.1
GCA_008330205.1
GCA_008330225.1
GCA_008330245.1

GCA_008330265.1
GCA_008330285.1
GCA_008330305.1
GCA_008330325.1
GCA_008330345.1
GCA_008330365.1
GCA_008330385.1
GCA_008330405.1
GCA_008330425.1
GCA_008330445.1
GCA_008330465.1
GCA_008330485.1
GCA_008330505.1
GCA_008330525.1
GCA_008330545.1
GCA_008330565.1
GCA_008330585.1
GCA_008330605.1
GCA_008330745.1
GCA_008330765.1
GCA_008330785.1
GCA_008330805.1
GCA_008330825.1
GCA_008330845.1
GCA_008330865.1
GCA_008330885.1
GCA_008330905.1
GCA_008330925.1
GCA_008330945.1
GCA_008330965.1
GCA_008364265.2
GCA_008364325.2
GCA_008365235.1
GCA_008365255.1
GCA_008365275.1
GCA_008365315.1
GCA_008365355.1
GCA_008367895.1
GCA_008369605.1
GCA_008369685.1
GCA_008369705.1
GCA_008369725.1
GCA_008369745.1
GCA_008369765.1
GCA_008369785.1
GCA_008369805.1
GCA_008370165.1
GCA_008370715.1
GCA_008370735.1
GCA_008370755.1
GCA_008370775.1
GCA_008370815.1
GCA_008370835.2
GCA_008370855.1

GCA_008375315.1
GCA_008375335.1
GCA_008386235.1
GCA_008386255.1
GCA_008386275.1
GCA_008386295.1
GCA_008386315.1
GCA_008386335.1
GCA_008386395.1
GCA_008432245.1
GCA_008432465.1
GCA_008461625.1
GCA_008461845.1
GCA_008462025.1
GCA_008462245.1
GCA_008462425.1
GCA_008505035.1
GCA_008534375.1
GCA_008586745.1
GCA_008586765.1
GCA_008586785.1
GCA_008586805.1
GCA_008586825.1
GCA_008586845.1
GCA_008593565.1
GCA_008605605.1
GCA_008619075.1
GCA_008619235.1
GCA_008619415.1
GCA_008619795.1
GCA_008619935.1
GCA_008620055.1
GCA_008620175.1
GCA_008630635.1
GCA_008630655.1
GCA_008630895.1
GCA_008630915.1
GCA_008630935.1
GCA_008632415.1
GCA_008632455.1
GCA_008632555.1
GCA_008632575.1
GCA_008632595.1
GCA_008632615.1
GCA_008632635.1
GCA_008632955.1
GCA_008639045.1
GCA_008639165.1
GCA_008642275.1
GCA_008642295.1
GCA_008642315.1
GCA_008642335.1
GCA_008642355.1
GCA_008642375.1

GCA_008642395.1
GCA_008642415.1
GCA_008693345.1
GCA_008693365.1
GCA_008693385.1
GCA_008693405.1
GCA_008693425.1
GCA_008693445.1
GCA_008693485.1
GCA_008693505.1
GCA_008693525.1
GCA_008693545.1
GCA_008693585.1
GCA_008693605.1
GCA_008693625.1
GCA_008693645.1
GCA_008693665.1
GCA_008693685.1
GCA_008693705.1
GCA_008693725.1
GCA_008693745.1
GCA_008693765.1
GCA_008693785.1
GCA_008693805.1
GCA_008693825.1
GCA_008693845.1
GCA_008693865.1
GCA_008693885.1
GCA_008693905.1
GCA_008693925.1
GCA_008693945.1
GCA_008693965.1
GCA_008693985.1
GCA_008694005.1
GCA_008694025.1
GCA_008694045.1
GCA_008694085.1
GCA_008694105.1
GCA_008704395.1
GCA_008704425.1
GCA_008704445.1
GCA_008704495.1
GCA_008704515.1
GCA_008704535.1
GCA_008704555.1
GCA_008704575.1
GCA_008704655.1
GCA_008704715.1
GCA_008704795.1
GCA_008704855.1
GCA_008704935.1
GCA_008704995.1
GCA_008705135.1
GCA_008705155.1

GCA_008705175.1
GCA_008705195.1
GCA_008705235.1
GCA_008705255.1
GCA_008726525.2
GCA_008727115.1
GCA_008727135.1
GCA_008727155.1
GCA_008727175.1
GCA_008727195.1
GCA_008727215.1
GCA_008727235.1
GCA_008727255.1
GCA_008727275.1
GCA_008727315.1
GCA_008727335.1
GCA_008727355.1
GCA_008727375.1
GCA_008727395.1
GCA_008727415.1
GCA_008727435.1
GCA_008727455.1
GCA_008727475.1
GCA_008727495.1
GCA_008727555.1
GCA_008727575.1
GCA_008727595.1
GCA_008727615.1
GCA_008727635.1
GCA_008727655.1
GCA_008727675.1
GCA_008727715.1
GCA_008727735.1
GCA_008727755.1
GCA_008727775.1
GCA_008727795.1
GCA_008727815.1
GCA_008727835.1
GCA_008728175.1
GCA_008728195.1
GCA_008728215.1
GCA_008728255.1
GCA_008728275.1
GCA_008728295.1
GCA_008728315.1
GCA_008728335.1
GCA_008728355.1
GCA_008728375.1
GCA_008728395.1
GCA_008728415.1
GCA_008728435.1
GCA_008728455.1
GCA_008728475.1
GCA_008728635.1

GCA_008728655.1
GCA_008728695.1
GCA_008728715.1
GCA_008728735.1
GCA_008728755.1
GCA_008728775.1
GCA_008728795.1
GCA_008728815.1
GCA_008728835.1
GCA_008728855.1
GCA_008728875.1
GCA_008728895.1
GCA_008728915.1
GCA_008728935.1
GCA_008761455.2
GCA_008761475.2
GCA_008761495.2
GCA_008761515.1
GCA_008761535.3
GCA_008761555.1
GCA_008761575.1
GCA_008761595.1
GCA_008761615.1
GCA_008761635.1
GCA_008761675.1
GCA_008801925.2
GCA_008802875.1
GCA_008802915.1
GCA_008802935.1
GCA_008803015.1
GCA_008805035.1
GCA_008806955.1
GCA_008806975.1
GCA_008806995.1
GCA_008807015.1
GCA_008807035.1
GCA_008807055.1
GCA_008807075.1
GCA_008807095.1
GCA_008807135.1
GCA_008807155.1
GCA_008807175.1
GCA_008807295.1
GCA_008807315.1
GCA_008807335.1
GCA_008807355.1
GCA_008807375.1
GCA_008807395.1
GCA_008807415.1
GCA_008807655.1
GCA_008807675.1
GCA_008807695.1
GCA_008807715.1
GCA_008807735.1

GCA_008807855.1
GCA_008807875.1
GCA_008807895.1
GCA_008807915.1
GCA_008807935.1
GCA_008807955.1
GCA_008807975.1
GCA_008807995.1
GCA_008808015.1
GCA_008808035.1
GCA_008808075.1
GCA_008808095.1
GCA_008823185.1
GCA_008824125.1
GCA_008824145.1
GCA_008824185.1
GCA_008824205.1
GCA_008831325.1
GCA_008831345.1
GCA_008831365.1
GCA_008831385.1
GCA_008831405.1
GCA_008831425.1
GCA_008831445.1
GCA_008831465.1
GCA_008831485.1
GCA_008831505.1
GCA_008831525.1
GCA_008838325.1
GCA_008843145.1
GCA_008843165.1
GCA_008876665.1
GCA_008925965.1
GCA_008926085.1
GCA_008926105.1
GCA_008926125.1
GCA_008926145.1
GCA_008926165.1
GCA_008926185.1
GCA_008926305.1
GCA_008926505.1
GCA_008931135.1
GCA_008931185.1
GCA_008931225.1
GCA_008931245.1
GCA_008931305.1
GCA_008931325.1
GCA_008931345.1
GCA_008931365.1
GCA_008931405.1
GCA_008931425.1
GCA_008931465.1
GCA_008931485.1
GCA_008931505.1

GCA_008931525.1
GCA_008931545.1
GCA_008931565.1
GCA_008931585.1
GCA_008931605.1
GCA_008931645.1
GCA_008931665.1
GCA_008931725.1
GCA_008931745.1
GCA_008931765.1
GCA_008931785.1
GCA_008931805.1
GCA_008931845.1
GCA_008931865.1
GCA_008932115.1
GCA_008932145.1
GCA_008932225.1
GCA_008932245.1
GCA_008935055.1
GCA_008935075.1
GCA_008949495.1
GCA_008973485.1
GCA_008995395.1
GCA_009002285.1
GCA_009002435.1
GCA_009002475.1
GCA_009002535.1
GCA_009017295.1
GCA_009017315.1
GCA_009017335.1
GCA_009017355.1
GCA_009017375.1
GCA_009017395.1
GCA_009017495.1
GCA_009025875.1
GCA_009025895.1
GCA_009025915.1
GCA_009026825.1
GCA_009035845.1
GCA_009036045.1
GCA_009036245.1
GCA_009036385.1
GCA_009068765.1
GCA_009156025.2
GCA_009176625.1
GCA_009176645.1
GCA_009176665.1
GCA_009176685.1
GCA_009176705.1
GCA_009176725.1
GCA_009176765.1
GCA_009176785.1
GCA_009176875.1
GCA_009176965.1

GCA_009177095.1
GCA_009177135.1
GCA_009177305.1
GCA_009177345.1
GCA_009183365.2
GCA_009184665.1
GCA_009184685.1
GCA_009184705.1
GCA_009184725.1
GCA_009184745.1
GCA_009184765.2
GCA_009184785.1
GCA_009184805.1
GCA_009184825.1
GCA_009184865.1
GCA_009189165.1
GCA_009189185.2
GCA_009191525.1
GCA_009193295.2
GCA_009193325.2
GCA_009217765.1
GCA_009218085.1
GCA_009223885.1
GCA_009224065.1
GCA_009258045.1
GCA_009258225.1
GCA_009295925.1
GCA_009295945.1
GCA_009295965.1
GCA_009295985.1
GCA_009296005.1
GCA_009296025.1
GCA_009296125.1
GCA_009296145.1
GCA_009299385.1
GCA_009301415.1
GCA_009362235.1
GCA_009362255.1
GCA_009362915.1
GCA_009362935.1
GCA_009362955.1
GCA_009362975.1
GCA_009362995.1
GCA_009363015.1
GCA_009363035.1
GCA_009363055.1
GCA_009363075.1
GCA_009363095.1
GCA_009363115.1
GCA_009363135.1
GCA_009363155.1
GCA_009363195.1
GCA_009363255.1
GCA_009363275.1

GCA_009363315.1
GCA_009363335.1
GCA_009363355.1
GCA_009363375.1
GCA_009363395.1
GCA_009363415.1
GCA_009363435.1
GCA_009363455.1
GCA_009363475.1
GCA_009363495.1
GCA_009363515.1
GCA_009363535.1
GCA_009363555.1
GCA_009363575.1
GCA_009363595.1
GCA_009363615.1
GCA_009363635.1
GCA_009363655.1
GCA_009363675.1
GCA_009363695.1
GCA_009363715.1
GCA_009363735.1
GCA_009363755.1
GCA_009363775.1
GCA_009363795.1
GCA_009363815.1
GCA_009363835.1
GCA_009363855.1
GCA_009363895.1
GCA_009372175.1
GCA_009372195.1
GCA_009387775.1
GCA_009387795.1
GCA_009387815.1
GCA_009387895.1
GCA_009387915.1
GCA_009387945.1
GCA_009388185.1
GCA_009388985.1
GCA_009394735.1
GCA_009395265.1
GCA_009428965.1
GCA_009429045.2
GCA_009429125.1
GCA_009429145.1
GCA_009431955.1
GCA_009432215.1
GCA_009432415.1
GCA_009432635.1
GCA_009432795.1
GCA_009432915.1
GCA_009433135.1
GCA_009455505.1
GCA_009495655.1

GCA_009495675.1
GCA_009496935.1
GCA_009496955.1
GCA_009496975.1
GCA_009497015.2
GCA_009497055.1
GCA_009497075.1
GCA_009497655.1
GCA_009497675.1
GCA_009497695.1
GCA_009497715.1
GCA_009497735.1
GCA_009497755.1
GCA_009497795.1
GCA_009497815.1
GCA_009497835.1
GCA_009497855.1
GCA_009497875.2
GCA_009497935.1
GCA_009497995.1
GCA_009498015.1
GCA_009498035.1
GCA_009498175.3
GCA_009498215.1
GCA_009498235.1
GCA_009498255.1
GCA_009498275.1
GCA_009498295.1
GCA_009498335.1
GCA_009498355.1
GCA_009498375.1
GCA_009498395.1
GCA_009498435.1
GCA_009498455.1
GCA_009498695.1
GCA_009556655.1
GCA_009557055.1
GCA_009557235.1
GCA_009557455.1
GCA_009557615.1
GCA_009577985.1
GCA_009578005.1
GCA_009586235.1
GCA_009588945.1
GCA_009601685.2
GCA_009601725.1
GCA_009601745.1
GCA_009601765.2
GCA_009617955.1
GCA_009618015.1
GCA_009618035.1
GCA_009619495.1
GCA_009625735.1
GCA_009625755.1

GCA_009625775.1
GCA_009625795.1
GCA_009625815.1
GCA_009625835.1
GCA_009625855.1
GCA_009625875.1
GCA_009625895.1
GCA_009625915.1
GCA_009625935.1
GCA_009625955.1
GCA_009625975.1
GCA_009625995.1
GCA_009626015.1
GCA_009626035.1
GCA_009626055.1
GCA_009626075.1
GCA_009626095.1
GCA_009626115.1
GCA_009626135.1
GCA_009626155.1
GCA_009626175.1
GCA_009626195.1
GCA_009626215.1
GCA_009626235.1
GCA_009626255.1
GCA_009626275.1
GCA_009626295.1
GCA_009626315.1
GCA_009626335.1
GCA_009626355.1
GCA_009626375.1
GCA_009626395.1
GCA_009626415.1
GCA_009626435.1
GCA_009626455.1
GCA_009626475.1
GCA_009626495.1
GCA_009626515.1
GCA_009626535.1
GCA_009626555.1
GCA_009626575.1
GCA_009626595.1
GCA_009626615.1
GCA_009626635.1
GCA_009626655.1
GCA_009626675.1
GCA_009626695.1
GCA_009626715.1
GCA_009626735.1
GCA_009626755.1
GCA_009626775.1
GCA_009626795.1
GCA_009626815.1
GCA_009626835.1

GCA_009626855.1
GCA_009626875.1
GCA_009626895.1
GCA_009626915.1
GCA_009626935.1
GCA_009626955.1
GCA_009626975.1
GCA_009626995.1
GCA_009627015.1
GCA_009627035.1
GCA_009627055.1
GCA_009627075.1
GCA_009627095.1
GCA_009627115.1
GCA_009627135.1
GCA_009627155.1
GCA_009627175.1
GCA_009627195.1
GCA_009627215.1
GCA_009627235.1
GCA_009627255.1
GCA_009627275.1
GCA_009627295.1
GCA_009627315.1
GCA_009627335.1
GCA_009627355.1
GCA_009627375.1
GCA_009627395.1
GCA_009627415.1
GCA_009627435.1
GCA_009627455.1
GCA_009627475.1
GCA_009627495.1
GCA_009627515.1
GCA_009627535.1
GCA_009627555.1
GCA_009627575.1
GCA_009627595.1
GCA_009627615.1
GCA_009627635.1
GCA_009627655.1
GCA_009627675.1
GCA_009627695.1
GCA_009627715.1
GCA_009627735.1
GCA_009627755.1
GCA_009627775.1
GCA_009627795.1
GCA_009627815.1
GCA_009627835.1
GCA_009627855.1
GCA_009627875.1
GCA_009627895.1
GCA_009627915.1

GCA_009627935.1
GCA_009627955.1
GCA_009627975.1
GCA_009646095.1
GCA_009646115.1
GCA_009646135.1
GCA_009646155.1
GCA_009648495.1
GCA_009648555.1
GCA_009648575.1
GCA_009648595.1
GCA_009648615.1
GCA_009648635.1
GCA_009648655.1
GCA_009648675.1
GCA_009648695.1
GCA_009648715.1
GCA_009648735.1
GCA_009648755.1
GCA_009648775.1
GCA_009648795.1
GCA_009648815.1
GCA_009648835.1
GCA_009648855.1
GCA_009648875.1
GCA_009648915.1
GCA_009648935.1
GCA_009648955.1
GCA_009648975.1
GCA_009648995.1
GCA_009649015.1
GCA_009649035.1
GCA_009649055.1
GCA_009649075.1
GCA_009649895.1
GCA_009649915.1
GCA_009649935.1
GCA_009649955.1
GCA_009649995.1
GCA_009650035.1
GCA_009650055.1
GCA_009650075.1
GCA_009650095.1
GCA_009650115.1
GCA_009650135.1
GCA_009650155.1
GCA_009650175.1
GCA_009650195.1
GCA_009650215.1
GCA_009650315.1
GCA_009650335.1
GCA_009650355.1
GCA_009650375.1
GCA_009650395.1

GCA_009650415.1
GCA_009650765.1
GCA_009660185.1
GCA_009660205.1
GCA_009660225.1
GCA_009660375.2
GCA_009661415.2
GCA_009661455.2
GCA_009661485.2
GCA_009661535.2
GCA_009661615.2
GCA_009661725.2
GCA_009662135.1
GCA_009662155.1
GCA_009662175.1
GCA_009662195.1
GCA_009662215.1
GCA_009662235.1
GCA_009662255.1
GCA_009662275.1
GCA_009662295.1
GCA_009662315.1
GCA_009662335.1
GCA_009662355.1
GCA_009662375.1
GCA_009662395.1
GCA_009662415.1
GCA_009662435.1
GCA_009662455.1
GCA_009662475.1
GCA_009662495.1
GCA_009663035.1
GCA_009663855.1
GCA_009664085.1
GCA_009664105.1
GCA_009664125.2
GCA_009664145.1
GCA_009664165.1
GCA_009664185.1
GCA_009664205.1
GCA_009664225.1
GCA_009664245.1
GCA_009664305.1
GCA_009664345.1
GCA_009664365.1
GCA_009664455.1
GCA_009664475.1
GCA_009664495.1
GCA_009664515.1
GCA_009664535.1
GCA_009664555.1
GCA_009664575.1
GCA_009664595.1
GCA_009664615.1

GCA_009664635.1
GCA_009664655.1
GCA_009664675.1
GCA_009664695.1
GCA_009664715.1
GCA_009664735.1
GCA_009664755.1
GCA_009664775.1
GCA_009664795.1
GCA_009664815.1
GCA_009664835.1
GCA_009664875.1
GCA_009664895.1
GCA_009665145.2
GCA_009665195.1
GCA_009665215.1
GCA_009665235.1
GCA_009665255.1
GCA_009665275.1
GCA_009665295.1
GCA_009665315.1
GCA_009665335.1
GCA_009665395.1
GCA_009665435.1
GCA_009665475.1
GCA_009665495.1
GCA_009667745.1
GCA_009667765.1
GCA_009667835.1
GCA_009667855.1
GCA_009670965.2
GCA_009671025.1
GCA_009671045.1
GCA_009671065.1
GCA_009671085.1
GCA_009671105.1
GCA_009671125.1
GCA_009671165.1
GCA_009671185.1
GCA_009671205.1
GCA_009676365.1
GCA_009676385.1
GCA_009676405.1
GCA_009676425.1
GCA_009676445.1
GCA_009676465.1
GCA_009676485.1
GCA_009676505.1
GCA_009676525.1
GCA_009676545.1
GCA_009676565.1
GCA_009676585.1
GCA_009676605.1
GCA_009676625.1

GCA_009676645.1
GCA_009676685.2
GCA_009676705.1
GCA_009676725.1
GCA_009676765.1
GCA_009676785.1
GCA_009676805.1
GCA_009676825.1
GCA_009676845.1
GCA_009676865.1
GCA_009676885.1
GCA_009676955.1
GCA_009684555.1
GCA_009684575.1
GCA_009684655.1
GCA_009684665.1
GCA_009684695.1
GCA_009684715.1
GCA_009685135.1
GCA_009685175.1
GCA_009685195.1
GCA_009688945.1
GCA_009688965.1
GCA_009688985.1
GCA_009689005.1
GCA_009690655.2
GCA_009696665.2
GCA_009696685.2
GCA_009697265.1
GCA_009697285.1
GCA_009707285.1
GCA_009707305.1
GCA_009707345.1
GCA_009707365.1
GCA_009707385.1
GCA_009707405.1
GCA_009707445.1
GCA_009707465.1
GCA_009707485.1
GCA_009707765.1
GCA_009708005.2
GCA_009708195.1
GCA_009708215.1
GCA_009708235.1
GCA_009708255.1
GCA_009708275.1
GCA_009708295.1
GCA_009708315.1
GCA_009708335.1
GCA_009708355.1
GCA_009708435.1
GCA_009708575.1
GCA_009708695.1
GCA_009708715.1

GCA_009708735.1
GCA_009708755.1
GCA_009708775.1
GCA_009708795.1
GCA_009708815.1
GCA_009708835.1
GCA_009708855.1
GCA_009708875.1
GCA_009708895.1
GCA_009708915.1
GCA_009708935.1
GCA_009708955.1
GCA_009708975.1
GCA_009708995.1
GCA_009709015.1
GCA_009709035.1
GCA_009709055.1
GCA_009709075.1
GCA_009709095.1
GCA_009709115.1
GCA_009709135.1
GCA_009709155.1
GCA_009709175.1
GCA_009709195.1
GCA_009709215.1
GCA_009709235.1
GCA_009709255.1
GCA_009709275.1
GCA_009709295.1
GCA_009709315.1
GCA_009709335.1
GCA_009709355.1
GCA_009709375.1
GCA_009709395.1
GCA_009709415.1
GCA_009709435.1
GCA_009709455.1
GCA_009709475.1
GCA_009709495.1
GCA_009709515.1
GCA_009709535.1
GCA_009720125.1
GCA_009720145.1
GCA_009720165.1
GCA_009720185.1
GCA_009720405.1
GCA_009720425.1
GCA_009720445.1
GCA_009720465.1
GCA_009720485.1
GCA_009720525.1
GCA_009720545.1
GCA_009720565.1
GCA_009720585.1

GCA_009720605.1
GCA_009720625.1
GCA_009724885.1
GCA_009724905.1
GCA_009728565.1
GCA_009728585.1
GCA_009728755.1
GCA_009728775.1
GCA_009728795.1
GCA_009728875.2
GCA_009728895.2
GCA_009728915.1
GCA_009728935.1
GCA_009728955.1
GCA_009728975.1
GCA_009729895.1
GCA_009729915.1
GCA_009729935.1
GCA_009729955.1
GCA_009729975.1
GCA_009729995.1
GCA_009730015.1
GCA_009730035.1
GCA_009730055.1
GCA_009730075.1
GCA_009730095.1
GCA_009730115.1
GCA_009730135.1
GCA_009730155.1
GCA_009730175.1
GCA_009730195.1
GCA_009730215.1
GCA_009730235.1
GCA_009730255.1
GCA_009730275.1
GCA_009730295.1
GCA_009730315.1
GCA_009730335.1
GCA_009730355.1
GCA_009730375.1
GCA_009730395.1
GCA_009730415.1
GCA_009730435.1
GCA_009730455.1
GCA_009730475.1
GCA_009730495.1
GCA_009730515.1
GCA_009730535.1
GCA_009730575.1
GCA_009730595.1
GCA_009730615.1
GCA_009730635.1
GCA_009730655.1
GCA_009730675.1

GCA_009731355.2
GCA_009731405.1
GCA_009731425.1
GCA_009731445.1
GCA_009731465.1
GCA_009731485.1
GCA_009731505.1
GCA_009731525.1
GCA_009731545.1
GCA_009731575.1
GCA_009731595.1
GCA_009732755.1
GCA_009732795.1
GCA_009732815.1
GCA_009732835.1
GCA_009733885.1
GCA_009734005.2
GCA_009734045.1
GCA_009734065.1
GCA_009734105.1
GCA_009734125.1
GCA_009734145.1
GCA_009734165.1
GCA_009734185.1
GCA_009734265.1
GCA_009734285.1
GCA_009734305.1
GCA_009734325.1
GCA_009734385.1
GCA_009734405.1
GCA_009734425.1
GCA_009734445.1
GCA_009734465.1
GCA_009734485.1
GCA_009738085.1
GCA_009738105.1
GCA_009738145.1
GCA_009738165.1
GCA_009738185.1
GCA_009738205.1
GCA_009738225.1
GCA_009738245.1
GCA_009738455.1
GCA_009738475.1
GCA_009738495.1
GCA_009738515.1
GCA_009738535.1
GCA_009738555.1
GCA_009738575.1
GCA_009738595.2
GCA_009739485.1
GCA_009739515.1
GCA_009739535.1
GCA_009739555.1

GCA_009739575.1
GCA_009739595.2
GCA_009739615.2
GCA_009739635.2
GCA_009739655.1
GCA_009739675.1
GCA_009739695.1
GCA_009739715.1
GCA_009739735.1
GCA_009739755.1
GCA_009739775.1
GCA_009739795.1
GCA_009739825.1
GCA_009739845.1
GCA_009739865.1
GCA_009739885.1
GCA_009739905.1
GCA_009739925.1
GCA_009739945.1
GCA_009739965.1
GCA_009739985.1
GCA_009740005.1
GCA_009740025.1
GCA_009740045.1
GCA_009740085.1
GCA_009740145.1
GCA_009740165.1
GCA_009740205.1
GCA_009740265.1
GCA_009740285.1
GCA_009740915.1
GCA_009741445.1
GCA_009745915.1
GCA_009746125.1
GCA_009749345.1
GCA_009754905.2
GCA_009755585.1
GCA_009755625.1
GCA_009755645.1
GCA_009755665.1
GCA_009755685.1
GCA_009755705.1
GCA_009755725.1
GCA_009755745.1
GCA_009755785.1
GCA_009756455.1
GCA_009756475.1
GCA_009756515.1
GCA_009756555.1
GCA_009756585.1
GCA_009756605.1
GCA_009756665.1
GCA_009758075.1
GCA_009758095.1

GCA_009759685.1
GCA_009759705.1
GCA_009759725.1
GCA_009759745.1
GCA_009759765.1
GCA_009759785.1
GCA_009759805.1
GCA_009759825.1
GCA_009759845.1
GCA_009761715.1
GCA_009761735.1
GCA_009762325.1
GCA_009762365.1
GCA_009762385.1
GCA_009762415.1
GCA_009762475.1
GCA_009762495.1
GCA_009762615.1
GCA_009762635.1
GCA_009762655.1
GCA_009762675.2
GCA_009762695.1
GCA_009762725.1
GCA_009762745.1
GCA_009762775.1
GCA_009762795.1
GCA_009762875.1
GCA_009762895.1
GCA_009762915.1
GCA_009762945.1
GCA_009762965.1
GCA_009762985.1
GCA_009763005.1
GCA_009763025.1
GCA_009763045.1
GCA_009763065.1
GCA_009763085.1
GCA_009763105.1
GCA_009763125.1
GCA_009763165.1
GCA_009763185.1
GCA_009763225.1
GCA_009763305.1
GCA_009763345.1
GCA_009763405.1
GCA_009763425.1
GCA_009763445.1
GCA_009763465.1
GCA_009763485.1
GCA_009763505.1
GCA_009763525.1
GCA_009763545.1
GCA_009763565.1
GCA_009763585.1

GCA_009763605.1
GCA_009763625.1
GCA_009763645.1
GCA_009763665.1
GCA_009763685.1
GCA_009763705.1
GCA_009763725.1
GCA_009763745.1
GCA_009763765.1
GCA_009763785.1
GCA_009763805.1
GCA_009763825.1
GCA_009763845.1
GCA_009763865.1
GCA_009763885.1
GCA_009763905.1
GCA_009763925.1
GCA_009763945.1
GCA_009763965.1
GCA_009763985.1
GCA_009764005.1
GCA_009764025.1
GCA_009764055.1
GCA_009764075.1
GCA_009764095.1
GCA_009764115.1
GCA_009764135.1
GCA_009764155.1
GCA_009764175.1
GCA_009764195.1
GCA_009764215.1
GCA_009769015.1
GCA_009769055.1
GCA_009769095.1
GCA_009769125.1
GCA_009769145.2
GCA_009769165.1
GCA_009769185.1
GCA_009769205.1
GCA_009789115.1
GCA_009789135.1
GCA_009789155.1
GCA_009789555.1
GCA_009789575.1
GCA_009789595.1
GCA_009789615.1
GCA_009789635.1
GCA_009789655.1
GCA_009789675.1
GCA_009791415.1
GCA_009791435.1
GCA_009791455.1
GCA_009791475.1
GCA_009791495.1

GCA_009791515.1
GCA_009792235.1
GCA_009792295.1
GCA_009792315.1
GCA_009792335.1
GCA_009792355.1
GCA_009796285.1
GCA_009796305.1
GCA_009796745.1
GCA_009796765.1
GCA_009796785.1
GCA_009796805.1
GCA_009796825.1
GCA_009796845.1
GCA_009796865.1
GCA_009797025.1
GCA_009797605.1
GCA_009797665.1
GCA_009799805.1
GCA_009799825.1
GCA_009799965.1
GCA_009800225.1
GCA_009806575.1
GCA_009806595.1
GCA_009806615.1
GCA_009806635.1
GCA_009806655.1
GCA_009806675.1
GCA_009806695.1
GCA_009806715.1
GCA_009806735.1
GCA_009810215.1
GCA_009817885.1
GCA_009818105.1
GCA_009818265.1
GCA_009827115.1
GCA_009827135.1
GCA_009828705.1
GCA_009831375.1
GCA_009831395.1
GCA_009831415.1
GCA_009831435.1
GCA_009831665.3
GCA_009832765.1
GCA_009832785.1
GCA_009832805.1
GCA_009832825.1
GCA_009832845.1
GCA_009832865.1
GCA_009832885.1
GCA_009832905.1
GCA_009832925.1
GCA_009832985.1
GCA_009833005.1

GCA_009833945.1
GCA_009833965.1
GCA_009834025.1
GCA_009834045.1
GCA_009834065.1
GCA_009834085.1
GCA_009834105.1
GCA_009834125.1
GCA_009834145.1
GCA_009834165.1
GCA_009834185.1
GCA_009834205.1
GCA_009834225.1
GCA_009834245.1
GCA_009834265.1
GCA_009834285.1
GCA_009834305.1
GCA_009834325.1
GCA_009834345.1
GCA_009834365.1
GCA_009834385.1
GCA_009834405.1
GCA_009834475.1
GCA_009834495.1
GCA_009834515.1
GCA_009834545.1
GCA_009834565.1
GCA_009834925.2
GCA_009846525.1
GCA_009846595.1
GCA_009856525.1
GCA_009856545.1
GCA_009856565.1
GCA_009856585.1
GCA_009856605.1
GCA_009856625.1
GCA_009856645.1
GCA_009856975.1
GCA_009856995.1
GCA_009857035.1
GCA_009857055.1
GCA_009857075.1
GCA_009857095.1
GCA_009857115.1
GCA_009857135.1
GCA_009857155.1
GCA_009857175.1
GCA_009857995.1
GCA_009858115.1
GCA_009858135.1
GCA_009858175.1
GCA_009858195.1
GCA_009858215.1
GCA_009859475.1

GCA_009859815.1
GCA_009859915.1
GCA_009861435.1
GCA_009866705.1
GCA_009866725.1
GCA_009866745.1
GCA_009866765.1
GCA_009866785.1
GCA_009866825.1
GCA_009866865.1
GCA_009866885.1
GCA_009866905.1
GCA_009866925.3
GCA_009866945.3
GCA_009867015.2
GCA_009867035.3
GCA_009867055.1
GCA_009867075.1
GCA_009867095.1
GCA_009867115.1
GCA_009867135.1
GCA_009873225.2
GCA_009873295.1
GCA_009873315.1
GCA_009873335.1
GCA_009873355.1
GCA_009873375.1
GCA_009873395.1
GCA_009873415.1
GCA_009873435.1
GCA_009873455.1
GCA_009873475.1
GCA_009873495.1
GCA_009883635.2
GCA_009883655.1
GCA_009883715.1
GCA_009883735.1
GCA_009883755.2
GCA_009883775.1
GCA_009883795.1
GCA_009883815.1
GCA_009883835.1
GCA_009883855.1
GCA_009883875.1
GCA_009883895.1
GCA_009884005.1
GCA_009884295.1
GCA_009884315.1
GCA_009884335.1
GCA_009884355.1
GCA_009884375.1
GCA_009884395.1
GCA_009884415.1
GCA_009884975.1

GCA_009892245.1
GCA_009892265.1
GCA_009892285.1
GCA_009892305.1
GCA_009892325.1
GCA_009892345.1
GCA_009892365.1
GCA_009892385.1
GCA_009901525.1
GCA_009903625.1
GCA_009905115.1
GCA_009905135.1
GCA_009905155.1
GCA_009905175.1
GCA_009905195.1
GCA_009905215.1
GCA_009905255.1
GCA_009905275.1
GCA_009905315.1
GCA_009905335.1
GCA_009905355.1
GCA_009906835.1
GCA_009906855.1
GCA_009906875.1
GCA_009906895.1
GCA_009906915.1
GCA_009906935.1
GCA_009906955.1
GCA_009909305.1
GCA_009909325.1
GCA_009909365.1
GCA_009909385.1
GCA_009909405.1
GCA_009909425.1
GCA_009909445.1
GCA_009909465.1
GCA_009909485.1
GCA_009909505.1
GCA_009910365.1
GCA_009911735.1
GCA_009911755.1
GCA_009911775.1
GCA_009911795.1
GCA_009911815.1
GCA_009911835.1
GCA_009911855.1
GCA_009911895.1
GCA_009911965.1
GCA_009912015.1
GCA_009912035.1
GCA_009912055.1
GCA_009912075.1
GCA_009912095.1
GCA_009912115.1

GCA_009912155.1
GCA_009912195.1
GCA_009912255.1
GCA_009912295.1
GCA_009912335.1
GCA_009912355.1
GCA_009912375.1
GCA_009912415.1
GCA_009912435.1
GCA_009912455.1
GCA_009912475.1
GCA_009912495.1
GCA_009912535.1
GCA_009912555.1
GCA_009912595.1
GCA_009912615.1
GCA_009912635.1
GCA_009912655.1
GCA_009912675.1
GCA_009912695.1
GCA_009912715.1
GCA_009912735.1
GCA_009912755.1
GCA_009912775.1
GCA_009912795.1
GCA_009912815.1
GCA_009912835.1
GCA_009912855.1
GCA_009912875.1
GCA_009912895.1
GCA_009912915.1
GCA_009912935.1
GCA_009912955.1
GCA_009912975.1
GCA_009912995.1
GCA_009913015.1
GCA_009913035.1
GCA_009913055.1
GCA_009913075.1
GCA_009913095.1
GCA_009913115.1
GCA_009913135.1
GCA_009913155.1
GCA_009913175.1
GCA_009913195.1
GCA_009913215.1
GCA_009913235.1
GCA_009913255.1
GCA_009913275.1
GCA_009913295.1
GCA_009913315.1
GCA_009913335.1
GCA_009913355.1
GCA_009913375.1

GCA_009913395.1
GCA_009913415.1
GCA_009913435.1
GCA_009913475.1
GCA_009913495.1
GCA_009913515.1
GCA_009913535.1
GCA_009913555.1
GCA_009913575.1
GCA_009913595.1
GCA_009913615.1
GCA_009913635.1
GCA_009913655.1
GCA_009913675.1
GCA_009913695.1
GCA_009913835.1
GCA_009913855.1
GCA_009913875.1
GCA_009913895.1
GCA_009913915.1
GCA_009913935.1
GCA_009913955.1
GCA_009913975.1
GCA_009913995.1
GCA_009914015.1
GCA_009914035.1
GCA_009914055.1
GCA_009914075.1
GCA_009914095.1
GCA_009914135.1
GCA_009914155.1
GCA_009914175.1
GCA_009914195.1
GCA_009914215.1
GCA_009914235.1
GCA_009914255.1
GCA_009914275.1
GCA_009914295.1
GCA_009914315.1
GCA_009914335.1
GCA_009914375.1
GCA_009914395.1
GCA_009914415.1
GCA_009914435.1
GCA_009914455.1
GCA_009914475.1
GCA_009914495.1
GCA_009914515.1
GCA_009914535.1
GCA_009914665.1
GCA_009930775.1
GCA_009930795.1
GCA_009930815.1
GCA_009930835.1

GCA_009930855.1
GCA_009930875.1
GCA_009930895.1
GCA_009930915.1
GCA_009930935.1
GCA_009930955.1
GCA_009930975.1
GCA_009930995.1
GCA_009931015.1
GCA_009931035.1
GCA_009931055.1
GCA_009931075.1
GCA_009931095.1
GCA_009931115.1
GCA_009931135.1
GCA_009931175.1
GCA_009931195.1
GCA_009931215.1
GCA_009931235.1
GCA_009931255.1
GCA_009931275.1
GCA_009931295.1
GCA_009931315.1
GCA_009931335.1
GCA_009931355.1
GCA_009931375.1
GCA_009931395.1
GCA_009931415.1
GCA_009931435.1
GCA_009931455.1
GCA_009931475.1
GCA_009931495.2
GCA_009931515.2
GCA_009931535.1
GCA_009931555.1
GCA_009931575.1
GCA_009931595.1
GCA_009931615.1
GCA_009931635.1
GCA_009931655.1
GCA_009931675.1
GCA_009931695.1
GCA_009931715.1
GCA_009932715.2
GCA_009932885.2
GCA_009932935.2
GCA_009933525.1
GCA_009933595.1
GCA_009936135.1
GCA_009936155.1
GCA_009936175.1
GCA_009936195.2
GCA_009936215.1
GCA_009936235.1

GCA_009936255.1
GCA_009936275.1
GCA_009936295.1
GCA_009936315.1
GCA_009936335.1
GCA_009936375.1
GCA_009937725.1
GCA_009937785.1
GCA_009937825.1
GCA_009937955.1
GCA_009937975.1
GCA_009937995.1
GCA_009938015.1
GCA_009938035.1
GCA_009938075.1
GCA_009938095.1
GCA_009938115.1
GCA_009938165.1
GCA_009938185.1
GCA_009938205.1
GCA_009938225.1
GCA_009938265.1
GCA_009938285.1
GCA_009938305.1
GCA_009938325.1
GCA_009939745.1
GCA_009939765.1
GCA_009939785.1
GCA_009939805.1
GCA_009939825.1
GCA_009939845.1
GCA_009939975.1
GCA_009940325.1
GCA_009940965.1
GCA_009941325.1
GCA_009941705.1
GCA_009942155.1
GCA_009942395.1
GCA_009942655.1
GCA_009942915.1
GCA_009943205.1
GCA_009943505.1
GCA_009943805.1
GCA_009944075.1
GCA_009944335.1
GCA_009944725.1
GCA_009945165.1
GCA_009945535.1
GCA_009945865.1
GCA_009946285.1
GCA_009946845.1
GCA_009947205.1
GCA_009947575.1
GCA_009947985.1

GCA_009948395.1
GCA_009948655.1
GCA_009949145.1
GCA_009949705.1
GCA_009950125.1
GCA_009950475.1
GCA_009950805.1
GCA_009951245.1
GCA_009951745.1
GCA_009952125.1
GCA_010061665.1
GCA_010092385.1
GCA_010092405.1
GCA_010092425.1
GCA_010092445.1
GCA_010092485.1
GCA_010092505.1
GCA_010092525.1
GCA_010092545.1
GCA_010092565.1
GCA_010092585.1
GCA_010092625.1
GCA_010092945.1
GCA_010092965.1
GCA_010092985.1
GCA_010093005.1
GCA_010093025.1
GCA_010093045.1
GCA_010093065.1
GCA_010103655.1
GCA_010104355.1
GCA_010119335.3
GCA_010119915.1
GCA_010119935.1
GCA_010119975.1
GCA_010120595.1
GCA_010120715.1
GCA_010120755.1
GCA_010183645.1
GCA_010183685.1
GCA_010196425.1
GCA_010201925.1
GCA_010202115.1
GCA_010202265.1
GCA_010202465.1
GCA_010202645.1
GCA_010202845.1
GCA_010223015.1
GCA_010223075.1
GCA_010223095.1
GCA_010223115.1
GCA_010223135.1
GCA_010223155.1
GCA_010223175.1

GCA_010223195.1
GCA_010223255.1
GCA_010223405.1
GCA_010223575.1
GCA_010229055.1
GCA_010229315.1
GCA_010229495.1
GCA_010229695.1
GCA_010229955.1
GCA_010230155.1
GCA_010230375.1
GCA_010230515.1
GCA_010230725.1
GCA_010230935.1
GCA_010231195.1
GCA_010231335.1
GCA_010231485.1
GCA_010231685.1
GCA_010232325.1
GCA_010232785.1
GCA_010232985.1
GCA_010233165.1
GCA_010233385.1
GCA_010287845.2
GCA_010318885.1
GCA_010319105.1
GCA_010319405.1
GCA_010319625.1
GCA_010319905.2
GCA_010320145.1
GCA_010320365.1
GCA_010365245.1
GCA_010365265.1
GCA_010365285.1
GCA_010365305.1
GCA_010365325.1
GCA_010365345.1
GCA_010365365.1
GCA_010365385.1
GCA_010365405.1
GCA_010365425.1
GCA_010365465.1
GCA_010365485.1
GCA_010365505.1
GCA_010365525.1
GCA_010365545.1
GCA_010365585.1
GCA_010365785.1
GCA_010365805.1
GCA_010365865.1
GCA_010367305.1
GCA_010384365.1
GCA_010442675.1
GCA_010442835.1

GCA_010443035.1
GCA_010443215.1
GCA_010448615.1
GCA_010448835.1
GCA_010450855.1
GCA_010450875.1
GCA_010450895.1
GCA_010450915.1
GCA_010469945.1
GCA_010508875.1
GCA_010509075.1
GCA_010509235.1
GCA_010509415.1
GCA_010509575.1
GCA_010509815.1
GCA_010523235.1
GCA_010537155.1
GCA_010537335.1
GCA_010586905.1
GCA_010586925.1
GCA_010586945.1
GCA_010586985.1
GCA_010587025.1
GCA_010587055.1
GCA_010587105.1
GCA_010587175.1
GCA_010587255.1
GCA_010587305.1
GCA_010587385.1
GCA_010668965.1
GCA_010668985.1
GCA_010669005.1
GCA_010669025.1
GCA_010669045.1
GCA_010669065.1
GCA_010669085.1
GCA_010669105.1
GCA_010669125.1
GCA_010669145.1
GCA_010669165.1
GCA_010669185.1
GCA_010669205.1
GCA_010669225.1
GCA_010669245.1
GCA_010669265.1
GCA_010669285.1
GCA_010669305.1
GCA_010681825.2
GCA_010692865.1
GCA_010692925.1
GCA_010694505.1
GCA_010706435.1
GCA_010724935.1
GCA_010725135.1

GCA_010725305.1
GCA_010725485.1
GCA_010725725.1
GCA_010725885.1
GCA_010726085.1
GCA_010726245.1
GCA_010726505.1
GCA_010726645.1
GCA_010726765.1
GCA_010726955.1
GCA_010727125.1
GCA_010727325.1
GCA_010727475.1
GCA_010727605.1
GCA_010727725.1
GCA_010727945.1
GCA_010728155.1
GCA_010728325.1
GCA_010728525.1
GCA_010728725.1
GCA_010728925.1
GCA_010729105.1
GCA_010729305.1
GCA_010729485.1
GCA_010729665.1
GCA_010729895.1
GCA_010730055.1
GCA_010730195.1
GCA_010730355.1
GCA_010730575.1
GCA_010730745.1
GCA_010731115.1
GCA_010731295.1
GCA_010731535.1
GCA_010731575.1
GCA_010731595.1
GCA_010731615.1
GCA_010731635.1
GCA_010731655.1
GCA_010731675.1
GCA_010731695.1
GCA_010731715.1
GCA_010731735.1
GCA_010731755.1
GCA_010731775.1
GCA_010731795.1
GCA_010731815.2
GCA_010731835.1
GCA_010731855.1
GCA_010731875.1
GCA_010731895.1
GCA_010731915.1
GCA_010731935.1
GCA_010748895.1

GCA_010748915.1
GCA_010748935.1
GCA_010748955.1
GCA_010917155.1
GCA_010917555.1
GCA_010917675.1
GCA_010917815.1
GCA_010918035.1
GCA_010918195.1
GCA_010918375.1
GCA_010918635.1
GCA_010918895.1
GCA_010919095.1
GCA_010919335.1
GCA_010977555.1
GCA_010977575.1
GCA_010978155.1
GCA_010983895.1
GCA_010993845.2
GCA_010993865.2
GCA_010993915.2
GCA_011006355.1
GCA_011006575.1
GCA_011008975.1
GCA_011009015.1
GCA_011022255.1
GCA_011022275.1
GCA_011022295.1
GCA_011032725.1
GCA_011032745.1
GCA_011032765.1
GCA_011032785.1
GCA_011035105.1
GCA_011035165.1
GCA_011038575.1
GCA_011038645.2
GCA_011038655.2
GCA_011038705.2
GCA_011038885.2
GCA_011039955.1
GCA_011040435.1
GCA_011040475.1
GCA_011040495.1
GCA_011044155.1
GCA_011044175.1
GCA_011044195.1
GCA_011044215.1
GCA_011044255.1
GCA_011044275.1
GCA_011044295.1
GCA_011044315.1
GCA_011044335.1
GCA_011044355.1
GCA_011044415.1

GCA_011044455.1
GCA_011044475.1
GCA_011044495.1
GCA_011044515.1
GCA_011045215.1
GCA_011045235.1
GCA_011045255.1
GCA_011045275.1
GCA_011045295.1
GCA_011045315.1
GCA_011045335.1
GCA_011045355.1
GCA_011045375.1
GCA_011045395.1
GCA_011045415.1
GCA_011045435.1
GCA_011045455.1
GCA_011045475.1
GCA_011045495.1
GCA_011045535.1
GCA_011045555.1
GCA_011045575.1
GCA_011045595.1
GCA_011045615.1
GCA_011045635.1
GCA_011045655.1
GCA_011045675.1
GCA_011045695.1
GCA_011045715.1
GCA_011045735.1
GCA_011045755.1
GCA_011045775.1
GCA_011045795.1
GCA_011045815.1
GCA_011045835.1
GCA_011045855.1
GCA_011045875.1
GCA_011045895.1
GCA_011046025.1
GCA_011046245.1
GCA_011046485.1
GCA_011046535.1
GCA_011046555.1
GCA_011046735.1
GCA_011046895.1
GCA_011046975.1
GCA_011047135.1
GCA_011047315.1
GCA_011057955.1
GCA_011057975.1
GCA_011058275.1
GCA_011058295.1
GCA_011058315.1
GCA_011058635.1

GCA_011058655.1
GCA_011058675.1
GCA_011058695.1
GCA_011058715.1
GCA_011058735.1
GCA_011058755.1
GCA_011058775.1
GCA_011058795.1
GCA_011064245.1
GCA_011064265.1
GCA_011064285.1
GCA_011064575.1
GCA_011064785.1
GCA_011064805.1
GCA_011064825.1
GCA_011064845.1
GCA_011065325.1
GCA_011065365.1
GCA_011065405.1
GCA_011066505.1
GCA_011066545.1
GCA_011067065.1
GCA_011067085.1
GCA_011067105.1
GCA_011067225.1
GCA_011067245.1
GCA_011068285.1
GCA_011068345.1
GCA_011068365.1
GCA_011068405.5
GCA_011082085.1
GCA_011082265.1
GCA_011082485.1
GCA_011082725.1
GCA_011100445.1
GCA_011100465.1
GCA_011100485.1
GCA_011106605.1
GCA_011106775.1
GCA_011106795.1
GCA_011106815.1
GCA_011106835.1
GCA_011106855.1
GCA_011129215.1
GCA_011149255.1
GCA_011149495.1
GCA_011149675.1
GCA_011193375.1
GCA_011207455.1
GCA_011212705.1
GCA_011220525.1
GCA_011290365.1
GCA_011290405.1
GCA_011290425.1

GCA_011290445.1
GCA_011290465.1
GCA_011290485.1
GCA_011290545.1
GCA_011290565.1
GCA_011290585.1
GCA_011290605.1
GCA_011290675.2
GCA_011292655.2
GCA_011299095.1
GCA_011299335.1
GCA_011299555.1
GCA_011299575.1
GCA_011299595.1
GCA_011299615.1
GCA_011300215.1
GCA_011300455.1
GCA_011300685.1
GCA_011300875.1
GCA_011301115.1
GCA_011301315.1
GCA_011301495.1
GCA_011301715.1
GCA_011301835.1
GCA_011302055.1
GCA_011302195.1
GCA_011302375.1
GCA_011302515.1
GCA_011302735.1
GCA_011302915.1
GCA_011303155.1
GCA_011303355.1
GCA_011303555.1
GCA_011303755.1
GCA_011303955.1
GCA_011304195.1
GCA_011304355.1
GCA_011304595.2
GCA_011304815.1
GCA_011305335.1
GCA_011305535.1
GCA_011305775.1
GCA_011305995.1
GCA_011306175.1
GCA_011326135.1
GCA_011330935.2
GCA_011331065.1
GCA_011331215.2
GCA_011382965.1
GCA_011382985.1
GCA_011383005.1
GCA_011383025.1
GCA_011383045.1
GCA_011383065.1

GCA_011383085.1
GCA_011386925.1
GCA_011386965.1
GCA_011387015.1
GCA_011387195.1
GCA_011388155.1
GCA_011388175.1
GCA_011388195.1
GCA_011388215.1
GCA_011390865.1
GCA_011393335.1
GCA_011396735.1
GCA_011397115.1
GCA_011397855.1
GCA_011398155.1
GCA_011398355.1
GCA_011398715.1
GCA_011398925.1
GCA_011399095.1
GCA_011399315.1
GCA_011399455.1
GCA_011400295.1
GCA_011403135.1
GCA_011403355.1
GCA_011404215.1
GCA_011404355.1
GCA_011404475.1
GCA_011404595.1
GCA_011404755.1
GCA_011404895.1
GCA_011405015.1
GCA_011405175.1
GCA_011405515.1
GCA_011405655.1
GCA_011420305.2
GCA_011455275.1
GCA_011455495.1
GCA_011455695.1
GCA_011455875.1
GCA_011456075.1
GCA_011456275.1
GCA_011456295.1
GCA_011456315.1
GCA_011456475.1
GCA_011456665.1
GCA_011456895.1
GCA_011457115.1
GCA_011457355.1
GCA_011457615.1
GCA_011457815.1
GCA_011458015.1
GCA_011458095.1
GCA_011458315.1
GCA_011462075.1

GCA_011462215.1
GCA_011462375.1
GCA_011462575.1
GCA_011462735.1
GCA_011462915.1
GCA_011463035.1
GCA_011463055.1
GCA_011463175.1
GCA_011463755.1
GCA_011463895.1
GCA_011463995.1
GCA_011464015.1
GCA_011464035.1
GCA_011464055.1
GCA_011464075.1
GCA_011464195.1
GCA_011464375.1
GCA_011464495.1
GCA_011464655.1
GCA_011464815.1
GCA_011464975.1
GCA_011465095.1
GCA_011465255.1
GCA_011465395.1
GCA_011465535.1
GCA_011465695.1
GCA_011465815.1
GCA_011465945.1
GCA_011466075.1
GCA_011466275.1
GCA_011466455.1
GCA_011466595.1
GCA_011466775.1
GCA_011466815.1
GCA_011466835.1
GCA_011466855.1
GCA_011466875.1
GCA_011492885.1
GCA_011492925.1
GCA_011492945.1
GCA_011492965.1
GCA_011492985.1
GCA_011493005.1
GCA_011516635.5
GCA_011516645.4
GCA_011516665.3
GCA_011578045.1
GCA_011578285.1
GCA_011578485.1
GCA_011600845.2
GCA_011600855.2
GCA_011600945.2
GCA_011600955.2
GCA_011600995.2

GCA_011601005.2
GCA_011601375.2
GCA_011602425.1
GCA_011602465.1
GCA_011602485.1
GCA_011602505.1
GCA_011604465.1
GCA_011604485.1
GCA_011604505.1
GCA_011604525.1
GCA_011604545.1
GCA_011604565.1
GCA_011604585.1
GCA_011604605.1
GCA_011604625.1
GCA_011604645.1
GCA_011604665.1
GCA_011604685.1
GCA_011604705.1
GCA_011604725.1
GCA_011604745.1
GCA_011604765.1
GCA_011604785.1
GCA_011604805.1
GCA_011604825.1
GCA_011611525.1
GCA_011611865.1
GCA_011612125.1
GCA_011612265.2
GCA_011612385.1
GCA_011612585.1
GCA_011612705.1
GCA_011617105.1
GCA_011617245.1
GCA_011617465.1
GCA_011617705.1
GCA_011650445.1
GCA_011691375.1
GCA_011691525.1
GCA_011694695.1
GCA_011694815.1
GCA_011742055.2
GCA_011742285.2
GCA_011742415.2
GCA_011742505.2
GCA_011742515.2
GCA_011742585.2
GCA_011745645.1
GCA_011745665.1
GCA_011751035.2
GCA_011751745.2
GCA_011751765.2
GCA_011751865.2
GCA_011751885.2

GCA_011751945.2
GCA_011752455.1
GCA_011752475.1
GCA_011752495.1
GCA_011764365.2
GCA_011764485.1
GCA_011764505.1
GCA_011764545.1
GCA_011764565.1
GCA_011764585.1
GCA_011764605.1
GCA_011764625.1
GCA_011765175.1
GCA_011765385.1
GCA_011765405.1
GCA_011765425.1
GCA_011765465.1
GCA_011765485.1
GCA_011765505.1
GCA_011765525.1
GCA_011765545.1
GCA_011765565.1
GCA_011765585.1
GCA_011765605.1
GCA_011765625.1
GCA_011769525.1
GCA_011769725.1
GCA_011769805.1
GCA_011769825.1
GCA_011769865.1
GCA_011769885.1
GCA_011769905.1
GCA_011769925.1
GCA_011769965.2
GCA_011769985.3
GCA_011770105.1
GCA_011784425.1
GCA_011784665.1
GCA_011784865.1
GCA_011784885.1
GCA_011801125.1
GCA_011801145.1
GCA_011801165.1
GCA_011801185.1
GCA_011801225.1
GCA_011801245.1
GCA_011801265.1
GCA_011801285.1
GCA_011801365.1
GCA_011801435.1
GCA_011801455.1
GCA_011801475.1
GCA_011820545.1
GCA_011995665.1

GCA_011995885.1
GCA_011996085.1
GCA_011996285.1
GCA_011996525.1
GCA_011996705.1
GCA_012029655.1
GCA_012029695.2
GCA_012029895.2
GCA_012029935.2
GCA_012029945.2
GCA_012029975.2
GCA_012030545.2
GCA_012030675.1
GCA_012030695.1
GCA_012044215.1
GCA_012044475.1
GCA_012044595.1
GCA_012044775.1
GCA_012044895.1
GCA_012045365.1
GCA_012045505.1
GCA_012049805.1
GCA_012050025.1
GCA_012050205.1
GCA_012050385.1
GCA_012050525.1
GCA_012050745.1
GCA_012050905.1
GCA_012051085.1
GCA_012051245.1
GCA_012051385.1
GCA_012051625.1
GCA_012052085.1
GCA_012052225.1
GCA_012052445.1
GCA_012052605.1
GCA_012052805.1
GCA_012052965.1
GCA_012053145.1
GCA_012053325.1
GCA_012053525.1
GCA_012053725.1
GCA_012053905.1
GCA_012054045.1
GCA_012109075.1
GCA_012109195.1
GCA_012109355.1
GCA_012113595.1
GCA_012113615.1
GCA_012113635.1
GCA_012113655.1
GCA_012149945.1
GCA_012151155.1
GCA_012151295.1

GCA_012151455.1
GCA_012151595.1
GCA_012166915.1
GCA_012167075.1
GCA_012167215.1
GCA_012167385.1
GCA_012168575.2
GCA_012169405.1
GCA_012219705.1
GCA_012219925.1
GCA_012220065.1
GCA_012220205.1
GCA_012220405.1
GCA_012220585.1
GCA_012220765.1
GCA_012220945.1
GCA_012221365.1
GCA_012221565.1
GCA_012221625.1
GCA_012221805.1
GCA_012222485.1
GCA_012222705.1
GCA_012222825.1
GCA_012222965.1
GCA_012224145.1
GCA_012224275.1
GCA_012224465.1
GCA_012224585.1
GCA_012224765.1
GCA_012224905.1
GCA_012225065.1
GCA_012225225.1
GCA_012225445.1
GCA_012225685.1
GCA_012225885.1
GCA_012226065.1
GCA_012241395.2
GCA_012271835.1
GCA_012272695.1
GCA_012272715.1
GCA_012272735.1
GCA_012272755.1
GCA_012272775.1
GCA_012272795.1
GCA_012272955.1
GCA_012272975.1
GCA_012273015.1
GCA_012273035.1
GCA_012273515.1
GCA_012273535.1
GCA_012273555.1
GCA_012273615.1
GCA_012275145.1
GCA_012275165.1

GCA_012275205.1
GCA_012276675.1
GCA_012276695.1
GCA_012277275.1
GCA_012277295.1
GCA_012277315.1
GCA_012277335.1
GCA_012278405.1
GCA_012278555.1
GCA_012278675.1
GCA_012278715.1
GCA_012278735.1
GCA_012295505.1
GCA_012295525.1
GCA_012295575.1
GCA_012295595.1
GCA_012295615.1
GCA_012317045.1
GCA_012317185.1
GCA_012317385.1
GCA_012317585.1
GCA_012427705.1
GCA_012427845.1
GCA_012516355.1
GCA_012516375.2
GCA_012516395.1
GCA_012516415.1
GCA_012516435.1
GCA_012516455.1
GCA_012516475.1
GCA_012516495.1
GCA_012516515.1
GCA_012524235.2
GCA_012530215.1
GCA_012530335.1
GCA_012530535.1
GCA_012530715.1
GCA_012534935.1
GCA_012537835.1
GCA_012558165.1
GCA_012562255.1
GCA_012562465.1
GCA_012562765.1
GCA_012563545.1
GCA_012571345.1
GCA_012572025.1
GCA_012572265.1
GCA_012584495.1
GCA_012584515.1
GCA_012593885.1
GCA_012594215.1
GCA_012647025.1
GCA_012647205.1
GCA_012647365.1

GCA_012647845.1
GCA_012648005.1
GCA_012648205.1
GCA_012648345.1
GCA_012676905.1
GCA_012689525.1
GCA_012689545.1
GCA_012689565.1
GCA_012767755.2
GCA_012769535.1
GCA_012844345.1
GCA_012844365.1
GCA_012844385.1
GCA_012844405.1
GCA_012848175.1
GCA_012848475.1
GCA_012848655.1
GCA_012848855.1
GCA_012849055.1
GCA_012849095.1
GCA_012849215.1
GCA_012849375.1
GCA_012849555.1
GCA_012849875.1
GCA_012850075.1
GCA_012850275.1
GCA_012850455.1
GCA_012850655.1
GCA_012850815.1
GCA_012850955.1
GCA_012851115.1
GCA_012851305.1
GCA_012871055.1
GCA_012889415.1
GCA_012889595.1
GCA_012889735.1
GCA_012889985.1
GCA_012890095.1
GCA_012897395.1
GCA_012897595.1
GCA_012897855.1
GCA_012898015.1
GCA_012913305.1
GCA_012913485.1
GCA_012913625.1
GCA_012913645.1
GCA_012923765.1
GCA_012923785.1
GCA_012931585.1
GCA_012931605.1
GCA_012931625.1
GCA_012931645.1
GCA_012931665.1
GCA_012931705.2

GCA_012931845.1
GCA_012932215.1
GCA_012932975.2
GCA_012932985.2
GCA_012933055.2
GCA_012933075.2
GCA_012933165.2
GCA_012933195.2
GCA_012933245.2
GCA_012933265.2
GCA_012933285.2
GCA_012933295.2
GCA_012933345.2
GCA_012933685.1
GCA_012934435.1
GCA_012934455.1
GCA_012934475.1
GCA_012934495.1
GCA_012934515.1
GCA_012934535.1
GCA_012934555.1
GCA_012934575.1
GCA_012934595.1
GCA_012934615.1
GCA_012934635.1
GCA_012934655.2
GCA_012934675.1
GCA_012934695.1
GCA_012934715.1
GCA_012934735.1
GCA_012934765.1
GCA_012934785.1
GCA_012934815.1
GCA_012934855.1
GCA_012934885.1
GCA_012934905.1
GCA_012934925.1
GCA_012934945.1
GCA_012934965.1
GCA_012934985.1
GCA_012935005.1
GCA_012935025.1
GCA_012935045.1
GCA_012935065.1
GCA_012935085.1
GCA_012935105.1
GCA_012935125.1
GCA_012935145.1
GCA_012935165.1
GCA_012935185.1
GCA_012935295.1
GCA_012935655.1
GCA_012935675.1
GCA_012935695.1

GCA_012935715.1
GCA_012935735.1
GCA_012935755.1
GCA_012935775.1
GCA_012944055.1
GCA_012955485.1
GCA_012955605.1
GCA_012970745.1
GCA_012971705.1
GCA_012971725.1
GCA_012971745.1
GCA_012971765.1
GCA_012972105.1
GCA_012972145.1
GCA_012974105.1
GCA_012974125.1
GCA_012974145.1
GCA_012974165.1
GCA_012974185.1
GCA_012974205.1
GCA_012974225.1
GCA_012974245.1
GCA_012974285.1
GCA_012974405.1
GCA_012974525.1
GCA_012974545.1
GCA_012974565.1
GCA_012974585.1
GCA_012974605.1
GCA_012974625.1
GCA_012974645.1
GCA_012974785.1
GCA_012974805.1
GCA_012974825.1
GCA_012974845.1
GCA_012974865.1
GCA_012975065.1
GCA_012975085.1
GCA_012979535.1
GCA_012980765.1
GCA_012980785.1
GCA_012980805.1
GCA_012980825.1
GCA_012980845.1
GCA_013000945.1
GCA_013000985.1
GCA_013001005.1
GCA_013001025.1
GCA_013003945.1
GCA_013003965.1
GCA_013003985.1
GCA_013004005.1
GCA_013004045.1
GCA_013004065.1

GCA_013004085.1
GCA_013004105.1
GCA_013004125.1
GCA_013008375.1
GCA_013008635.1
GCA_013008815.1
GCA_013009255.1
GCA_013009385.1
GCA_013009555.1
GCA_013009675.1
GCA_013009875.1
GCA_013010095.1
GCA_013010255.1
GCA_013010365.1
GCA_013010385.1
GCA_013014885.1
GCA_013030055.1
GCA_013030075.1
GCA_013030095.1
GCA_013030115.1
GCA_013030135.1
GCA_013046785.1
GCA_013046805.1
GCA_013046825.1
GCA_013046845.1
GCA_013046865.1
GCA_013046885.1
GCA_013047165.1
GCA_013084945.1
GCA_013085025.1
GCA_013085075.1
GCA_013085105.1
GCA_013085125.1
GCA_013085145.1
GCA_013085165.1
GCA_013085185.1
GCA_013085205.1
GCA_013085465.1
GCA_013085485.1
GCA_013085505.1
GCA_013085525.1
GCA_013085545.1
GCA_013085725.1
GCA_013085745.1
GCA_013085765.1
GCA_013085785.1
GCA_013085865.1
GCA_013085885.1
GCA_013085905.3
GCA_013098515.1
GCA_013098655.1
GCA_013098855.1
GCA_013099015.1
GCA_013099195.1

GCA_013100805.1
GCA_013100825.1
GCA_013100845.1
GCA_013112015.1
GCA_013112035.1
GCA_013112335.1
GCA_013112375.1
GCA_013112395.1
GCA_013112415.1
GCA_013113675.1
GCA_013113715.1
GCA_013113735.1
GCA_013113755.1
GCA_013113775.1
GCA_013114975.1
GCA_013116765.2
GCA_013122035.1
GCA_013122055.1
GCA_013122115.1
GCA_013122135.1
GCA_013122155.1
GCA_013122195.1
GCA_013122215.1
GCA_013122235.1
GCA_013122255.1
GCA_013122275.1
GCA_013122565.1
GCA_013122645.1
GCA_013127315.1
GCA_013127575.1
GCA_013127755.1
GCA_013127955.1
GCA_013128195.2
GCA_013137895.1
GCA_013137915.1
GCA_013137935.1
GCA_013137955.1
GCA_013138145.1
GCA_013138395.1
GCA_013146705.1
GCA_013146725.1
GCA_013146745.1
GCA_013146765.1
GCA_013146785.1
GCA_013146805.1
GCA_013146845.1
GCA_013147065.1
GCA_013147165.1
GCA_013147185.1
GCA_013147205.1
GCA_013154915.1
GCA_013154935.1
GCA_013155105.1
GCA_013155125.1

GCA_013155145.1
GCA_013161625.1
GCA_013161805.1
GCA_013162025.1
GCA_013162165.1
GCA_013162305.1
GCA_013162485.1
GCA_013166855.1
GCA_013166875.1
GCA_013166895.1
GCA_013166935.1
GCA_013166955.1
GCA_013166975.1
GCA_013166995.1
GCA_013167015.1
GCA_013167035.1
GCA_013167055.1
GCA_013167075.1
GCA_013167115.1
GCA_013167135.1
GCA_013167155.1
GCA_013167175.1
GCA_013167195.1
GCA_013167235.1
GCA_013167255.1
GCA_013167275.1
GCA_013167295.1
GCA_013167315.1
GCA_013167335.1
GCA_013167355.1
GCA_013167375.1
GCA_013167395.1
GCA_013167415.1
GCA_013167435.1
GCA_013167455.1
GCA_013167475.1
GCA_013167495.1
GCA_013167515.1
GCA_013167535.1
GCA_013167555.1
GCA_013167575.1
GCA_013167595.1
GCA_013167615.1
GCA_013167635.1
GCA_013167655.1
GCA_013167675.1
GCA_013167695.1
GCA_013167715.1
GCA_013167735.1
GCA_013167755.1
GCA_013167775.1
GCA_013167795.1
GCA_013167815.1
GCA_013167875.1

GCA_013167935.1
GCA_013167975.1
GCA_013167995.1
GCA_013168015.1
GCA_013168035.1
GCA_013168055.1
GCA_013168075.1
GCA_013168095.1
GCA_013168115.1
GCA_013168135.1
GCA_013168155.1
GCA_013168175.1
GCA_013168195.1
GCA_013168215.1
GCA_013168235.1
GCA_013168255.1
GCA_013168275.1
GCA_013168295.1
GCA_013170705.1
GCA_013170725.1
GCA_013170745.1
GCA_013170765.1
GCA_013170785.1
GCA_013170805.1
GCA_013170825.1
GCA_013170845.1
GCA_013170865.1
GCA_013171285.1
GCA_013171305.1
GCA_013171325.1
GCA_013177295.1
GCA_013177315.1
GCA_013177355.1
GCA_013177375.1
GCA_013177395.1
GCA_013177415.1
GCA_013177435.1
GCA_013177455.1
GCA_013177475.1
GCA_013177495.2
GCA_013177635.1
GCA_013177655.1
GCA_013177675.1
GCA_013181325.1
GCA_013181395.2
GCA_013181415.1
GCA_013184985.2
GCA_013187685.1
GCA_013200955.2
GCA_013201035.1
GCA_013201055.1
GCA_013201075.1
GCA_013201095.1
GCA_013201115.1

GCA_013201135.1
GCA_013201175.1
GCA_013201195.1
GCA_013201215.1
GCA_013201235.1
GCA_013201255.1
GCA_013201465.1
GCA_013201485.1
GCA_013201505.1
GCA_013201525.1
GCA_013201545.1
GCA_013201605.1
GCA_013201625.1
GCA_013201645.1
GCA_013201665.1
GCA_013201685.1
GCA_013201705.1
GCA_013201725.1
GCA_013201825.1
GCA_013201895.1
GCA_013201935.1
GCA_013201975.1
GCA_013205725.1
GCA_013206965.1
GCA_013207105.1
GCA_013234275.1
GCA_013255565.1
GCA_013255765.1
GCA_013255915.1
GCA_013256255.1
GCA_013256425.1
GCA_013256625.1
GCA_013256965.1
GCA_013260315.1
GCA_013260445.1
GCA_013260645.1
GCA_013265555.1
GCA_013265585.1
GCA_013267235.1
GCA_013267255.1
GCA_013267275.1
GCA_013267295.1
GCA_013267315.1
GCA_013267335.1
GCA_013267355.1
GCA_013267375.1
GCA_013267395.1
GCA_013267415.1
GCA_013267435.1
GCA_013267455.1
GCA_013267475.1
GCA_013267495.1
GCA_013267515.1
GCA_013267535.1

GCA_013267555.1
GCA_013267575.1
GCA_013267595.1
GCA_013267615.1
GCA_013267635.1
GCA_013267655.1
GCA_013267675.1
GCA_013267695.1
GCA_013267715.1
GCA_013267735.1
GCA_013267755.1
GCA_013267775.1
GCA_013267795.1
GCA_013267815.1
GCA_013267835.1
GCA_013282195.1
GCA_013282215.1
GCA_013282235.1
GCA_013282255.1
GCA_013282275.1
GCA_013282295.1
GCA_013282315.1
GCA_013282335.1
GCA_013282355.1
GCA_013282625.1
GCA_013282725.1
GCA_013282765.1
GCA_013282785.1
GCA_013282805.1
GCA_013283835.1
GCA_013283855.1
GCA_013283875.1
GCA_013283895.1
GCA_013283915.1
GCA_013283935.1
GCA_013283955.1
GCA_013283975.1
GCA_013283995.1
GCA_013284015.3
GCA_013284035.1
GCA_013284055.1
GCA_013284245.2
GCA_013284375.2
GCA_013284455.2
GCA_013284505.2
GCA_013284785.2
GCA_013285085.2
GCA_013285285.1
GCA_013285305.1
GCA_013285325.1
GCA_013285385.1
GCA_013285525.1
GCA_013305245.1
GCA_013305265.1

GCA_013305285.1
GCA_013305305.1
GCA_013305325.1
GCA_013305465.1
GCA_013305625.1
GCA_013305645.2
GCA_013305665.1
GCA_013305685.1
GCA_013305705.1
GCA_013305725.1
GCA_013305765.1
GCA_013305815.1
GCA_013305845.1
GCA_013305885.1
GCA_013305905.1
GCA_013305955.1
GCA_013306015.1
GCA_013306095.1
GCA_013306155.1
GCA_013306205.1
GCA_013306235.1
GCA_013306255.1
GCA_013306275.1
GCA_013306295.1
GCA_013306315.1
GCA_013306335.1
GCA_013306355.1
GCA_013306375.1
GCA_013306395.1
GCA_013306415.1
GCA_013306435.1
GCA_013306455.1
GCA_013306475.1
GCA_013306495.1
GCA_013306515.1
GCA_013306545.1
GCA_013306575.1
GCA_013306655.1
GCA_013306745.1
GCA_013306765.1
GCA_013306785.1
GCA_013306805.1
GCA_013306825.1
GCA_013306845.1
GCA_013306875.1
GCA_013306915.1
GCA_013306935.1
GCA_013306955.1
GCA_013306985.1
GCA_013307005.2
GCA_013307025.1
GCA_013307065.1
GCA_013307085.1
GCA_013307105.1

GCA_013307125.1
GCA_013307145.1
GCA_013307165.1
GCA_013307185.1
GCA_013307225.1
GCA_013307245.1
GCA_013307265.1
GCA_013307285.1
GCA_013307435.2
GCA_013315675.1
GCA_013315695.1
GCA_013315715.1
GCA_013315735.1
GCA_013315755.1
GCA_013315815.1
GCA_013315835.1
GCA_013315855.1
GCA_013315875.1
GCA_013315915.1
GCA_013315935.1
GCA_013315975.1
GCA_013315995.1
GCA_013317085.1
GCA_013317105.1
GCA_013317125.1
GCA_013317615.2
GCA_013317665.2
GCA_013317735.2
GCA_013317765.2
GCA_013317865.2
GCA_013317965.2
GCA_013318015.2
GCA_013318055.2
GCA_013318125.2
GCA_013318255.2
GCA_013318275.2
GCA_013318335.2
GCA_013318355.2
GCA_013328835.1
GCA_013328855.1
GCA_013331815.1
GCA_013334125.1
GCA_013334145.1
GCA_013334165.1
GCA_013334185.1
GCA_013334205.1
GCA_013340725.1
GCA_013340745.1
GCA_013340785.1
GCA_013340805.1
GCA_013340825.1
GCA_013340845.1
GCA_013341255.1
GCA_013341275.1

GCA_013341295.1
GCA_013341315.1
GCA_013341335.1
GCA_013341355.1
GCA_013341375.1
GCA_013341395.1
GCA_013341415.1
GCA_013341435.1
GCA_013341455.1
GCA_013342925.1
GCA_013342945.1
GCA_013342985.1
GCA_013343005.1
GCA_013343055.1
GCA_013343075.1
GCA_013343095.1
GCA_013343115.1
GCA_013343135.1
GCA_013343155.1
GCA_013343175.2
GCA_013343195.3
GCA_013343215.1
GCA_013343235.1
GCA_013343255.1
GCA_013343315.1
GCA_013343335.1
GCA_013343355.1
GCA_013343375.1
GCA_013343395.1
GCA_013343415.1
GCA_013343435.1
GCA_013343455.1
GCA_013343475.1
GCA_013343495.1
GCA_013343515.1
GCA_013343535.1
GCA_013343555.1
GCA_013343575.1
GCA_013343595.1
GCA_013343615.1
GCA_013343635.1
GCA_013344545.1
GCA_013344605.1
GCA_013344625.1
GCA_013344645.1
GCA_013344665.1
GCA_013344685.1
GCA_013347105.1
GCA_013347125.1
GCA_013347205.1
GCA_013347225.1
GCA_013347245.1
GCA_013347265.1
GCA_013347285.1

GCA_013347305.1
GCA_013347325.1
GCA_013347965.1
GCA_013348005.1
GCA_013348685.1
GCA_013348705.1
GCA_013348725.1
GCA_013348745.1
GCA_013348805.1
GCA_013348825.1
GCA_013348845.1
GCA_013348865.1
GCA_013348885.1
GCA_013348905.1
GCA_013348925.1
GCA_013348945.1
GCA_013348965.1
GCA_013348985.1
GCA_013349005.1
GCA_013349025.1
GCA_013349045.1
GCA_013349065.1
GCA_013349085.1
GCA_013357345.1
GCA_013357365.1
GCA_013357385.1
GCA_013357405.1
GCA_013357425.1
GCA_013357445.1
GCA_013357465.1
GCA_013357485.1
GCA_013357505.1
GCA_013357525.1
GCA_013357545.1
GCA_013357565.1
GCA_013357585.1
GCA_013357605.1
GCA_013357625.1
GCA_013357645.1
GCA_013357665.1
GCA_013357685.1
GCA_013357725.1
GCA_013357745.1
GCA_013357765.1
GCA_013357785.1
GCA_013357805.1
GCA_013357825.1
GCA_013357845.1
GCA_013357865.1
GCA_013357885.1
GCA_013358365.1
GCA_013358405.1
GCA_013358425.1
GCA_013358795.1

GCA_013363755.1
GCA_013363895.1
GCA_013363915.1
GCA_013363935.1
GCA_013364075.1
GCA_013364095.1
GCA_013364115.1
GCA_013364135.1
GCA_013364155.1
GCA_013364175.1
GCA_013364195.1
GCA_013364215.1
GCA_013364235.1
GCA_013364275.1
GCA_013364295.1
GCA_013364315.1
GCA_013364335.1
GCA_013364355.1
GCA_013364375.1
GCA_013365435.1
GCA_013365455.1
GCA_013365475.1
GCA_013365495.1
GCA_013365515.1
GCA_013365535.1
GCA_013366555.2
GCA_013366565.2
GCA_013366575.2
GCA_013366585.2
GCA_013366635.2
GCA_013367715.1
GCA_013367735.1
GCA_013367755.1
GCA_013367775.1
GCA_013367855.1
GCA_013367895.1
GCA_013367915.1
GCA_013367935.1
GCA_013367955.1
GCA_013367975.1
GCA_013367995.1
GCA_013368015.1
GCA_013368035.1
GCA_013368055.1
GCA_013368735.1
GCA_013368775.1
GCA_013371665.1
GCA_013371685.1
GCA_013371705.1
GCA_013371725.1
GCA_013371745.1
GCA_013372025.1
GCA_013372045.1
GCA_013372065.1

GCA_013372085.1
GCA_013372105.1
GCA_013372125.1
GCA_013372145.1
GCA_013372165.1
GCA_013372185.1
GCA_013372205.1
GCA_013372225.1
GCA_013372245.1
GCA_013372265.1
GCA_013372285.1
GCA_013372305.1
GCA_013372325.1
GCA_013372345.1
GCA_013372365.1
GCA_013372385.1
GCA_013372405.1
GCA_013372425.1
GCA_013373365.1
GCA_013373555.1
GCA_013373785.3
GCA_013373795.3
GCA_013374095.1
GCA_013374115.1
GCA_013374135.1
GCA_013374155.1
GCA_013374175.1
GCA_013374195.1
GCA_013374215.1
GCA_013374235.2
GCA_013374255.1
GCA_013374275.1
GCA_013374295.2
GCA_013374315.1
GCA_013374335.1
GCA_013374795.1
GCA_013374815.1
GCA_013374835.1
GCA_013374855.1
GCA_013374995.1
GCA_013375015.1
GCA_013375035.1
GCA_013375055.1
GCA_013375715.1
GCA_013375735.1
GCA_013375955.1
GCA_013375975.1
GCA_013376455.1
GCA_013376475.1
GCA_013376495.1
GCA_013376515.1
GCA_013376535.2
GCA_013376555.1
GCA_013376575.1

GCA_013376595.1
GCA_013376775.1
GCA_013376795.1
GCA_013376815.1
GCA_013376835.1
GCA_013376855.2
GCA_013376895.1
GCA_013376915.1
GCA_013376935.1
GCA_013377175.1
GCA_013377195.1
GCA_013377215.1
GCA_013377235.1
GCA_013377295.1
GCA_013377315.1
GCA_013377335.1
GCA_013377355.1
GCA_013377375.1
GCA_013377595.2
GCA_013377655.2
GCA_013377665.2
GCA_013377975.1
GCA_013377995.1
GCA_013378015.1
GCA_013378035.1
GCA_013378055.1
GCA_013378075.1
GCA_013378095.1
GCA_013378115.1
GCA_013378135.1
GCA_013378155.1
GCA_013378175.1
GCA_013378195.1
GCA_013378215.1
GCA_013378235.1
GCA_013378255.1
GCA_013378275.1
GCA_013378295.1
GCA_013378315.1
GCA_013378335.1
GCA_013378355.1
GCA_013388275.1
GCA_013388295.1
GCA_013388335.1
GCA_013388375.1
GCA_013389395.1
GCA_013389415.1
GCA_013389435.1
GCA_013389455.1
GCA_013389555.1
GCA_013389575.1
GCA_013389595.1
GCA_013389615.1
GCA_013389665.1

GCA_013389695.1
GCA_013389715.1
GCA_013389765.1
GCA_013390265.1
GCA_013390305.1
GCA_013390325.1
GCA_013391125.1
GCA_013391145.1
GCA_013391165.1
GCA_013391185.1
GCA_013391205.1
GCA_013391805.1
GCA_013391825.1
GCA_013391845.1
GCA_013393365.1
GCA_013393385.1
GCA_013393665.2
GCA_013393685.1
GCA_013393705.1
GCA_013393745.1
GCA_013393765.1
GCA_013393785.1
GCA_013393805.1
GCA_013393825.1
GCA_013393845.1
GCA_013393865.1
GCA_013393885.1
GCA_013393905.1
GCA_013393925.1
GCA_013393945.1
GCA_013394005.1
GCA_013394025.1
GCA_013394045.1
GCA_013394065.1
GCA_013394105.1
GCA_013394125.1
GCA_013394145.1
GCA_013394165.1
GCA_013394225.1
GCA_013394245.1
GCA_013394265.1
GCA_013394285.1
GCA_013394305.1
GCA_013394325.1
GCA_013394385.1
GCA_013394405.1
GCA_013394425.1
GCA_013394455.1
GCA_013394475.2
GCA_013394495.1
GCA_013394515.1
GCA_013394535.1
GCA_013394555.1
GCA_013394575.1

GCA_013394595.1
GCA_013394615.1
GCA_013394635.1
GCA_013394655.1
GCA_013394675.1
GCA_013394695.1
GCA_013394715.1
GCA_013394735.1
GCA_013394755.1
GCA_013394915.1
GCA_013395015.3
GCA_013395035.1
GCA_013395055.1
GCA_013395075.1
GCA_013401395.2
GCA_013401415.1
GCA_013401475.1
GCA_013401495.1
GCA_013401535.1
GCA_013402755.1
GCA_013402775.1
GCA_013402795.1
GCA_013402835.1
GCA_013402855.1
GCA_013402895.1
GCA_013403435.2
GCA_013403485.1
GCA_013403505.1
GCA_013403525.1
GCA_013403545.1
GCA_013403565.1
GCA_013403585.1
GCA_013407605.1
GCA_013407715.1
GCA_013407825.1
GCA_013407925.2
GCA_013408255.1
GCA_013408385.1
GCA_013408625.1
GCA_013408755.1
GCA_013408885.1
GCA_013409055.1
GCA_013409125.2
GCA_013409165.1
GCA_013409685.2
GCA_013410155.2
GCA_013414285.1
GCA_013414305.1
GCA_013414325.1
GCA_013414345.1
GCA_013414705.1
GCA_013414795.1
GCA_013414865.1
GCA_013415825.1

GCA_013415845.1
GCA_013415925.1
GCA_013415955.1
GCA_013415975.1
GCA_013415995.1
GCA_013416015.1
GCA_013416035.1
GCA_013416055.1
GCA_013416085.1
GCA_013416105.1
GCA_013416125.1
GCA_013416235.1
GCA_013416255.1
GCA_013416275.1
GCA_013423765.1
GCA_013423785.1
GCA_013423805.1
GCA_013423825.1
GCA_013423845.1
GCA_013423865.1
GCA_013423885.1
GCA_013423905.1
GCA_013423925.1
GCA_013425825.1
GCA_013425935.1
GCA_013425955.1
GCA_013425975.1
GCA_013425995.1
GCA_013426035.1
GCA_013426055.1
GCA_013426075.1
GCA_013426095.1
GCA_013426115.1
GCA_013426135.1
GCA_013426155.1
GCA_013426185.1
GCA_013426235.1
GCA_013426895.1
GCA_013426915.1
GCA_013427035.1
GCA_013456995.1
GCA_013457195.1
GCA_013457615.1
GCA_013457875.1
GCA_013458095.1
GCA_013458335.1
GCA_013458535.1
GCA_013458815.1
GCA_013459015.1
GCA_013459175.1
GCA_013459415.1
GCA_013459645.1
GCA_013459835.1
GCA_013460135.1

GCA_013460375.1
GCA_013460855.1
GCA_013461095.1
GCA_013461435.1
GCA_013461585.1
GCA_013462075.1
GCA_013462275.1
GCA_013462495.1
GCA_013462805.1
GCA_013462975.1
GCA_013463155.1
GCA_013463375.1
GCA_013463555.1
GCA_013465595.1
GCA_013465795.1
GCA_013466015.1
GCA_013466235.1
GCA_013466425.1
GCA_013466605.1
GCA_013466785.1
GCA_013466985.1
GCA_013467165.1
GCA_013467405.1
GCA_013467585.1
GCA_013467605.1
GCA_013467635.1
GCA_013467655.1
GCA_013487745.1
GCA_013487785.1
GCA_013487805.1
GCA_013487825.1
GCA_013487845.1
GCA_013487865.1
GCA_013487885.1
GCA_013487905.1
GCA_013487925.1
GCA_013487945.1
GCA_013487965.1
GCA_013487985.1
GCA_013488005.1
GCA_013488025.1
GCA_013488085.1
GCA_013488105.1
GCA_013488125.1
GCA_013488145.1
GCA_013488165.1
GCA_013488185.1
GCA_013488205.1
GCA_013488225.1
GCA_013591455.1
GCA_013596835.1
GCA_013610655.1
GCA_013611495.1
GCA_013615235.1

GCA_013615435.1
GCA_013624225.1
GCA_013624375.1
GCA_013624435.2
GCA_013624605.1
GCA_013624775.1
GCA_013624995.1
GCA_013625115.1
GCA_013625265.1
GCA_013625435.1
GCA_013625575.1
GCA_013625795.1
GCA_013629235.1
GCA_013629455.1
GCA_013629675.1
GCA_013635015.1
GCA_013635255.1
GCA_013635495.1
GCA_013635755.1
GCA_013636045.1
GCA_013636235.1
GCA_013636415.1
GCA_013693715.1
GCA_013693735.1
GCA_013693755.1
GCA_013693775.1
GCA_013693835.1
GCA_013693915.1
GCA_013693935.1
GCA_013693955.1
GCA_013694265.1
GCA_013694285.1
GCA_013694305.1
GCA_013694325.1
GCA_013694345.1
GCA_013694365.1
GCA_013694385.1
GCA_013694405.1
GCA_013694425.1
GCA_013694465.1
GCA_013701805.1
GCA_013702025.1
GCA_013702185.1
GCA_013702245.1
GCA_013702265.1
GCA_013704765.1
GCA_013704985.1
GCA_013724605.1
GCA_013724765.1
GCA_013724985.1
GCA_013725595.1
GCA_013725995.1
GCA_013726215.1
GCA_013726395.1

GCA_013726615.1
GCA_013727235.1
GCA_013727455.1
GCA_013727635.1
GCA_013728275.1
GCA_013728935.1
GCA_013729075.1
GCA_013730235.1
GCA_013730675.1
GCA_013731155.1
GCA_013731305.1
GCA_013731735.1
GCA_013731955.1
GCA_013732895.1
GCA_013733395.1
GCA_013734035.1
GCA_013734275.1
GCA_013734595.1
GCA_013734915.1
GCA_013735155.1
GCA_013735435.1
GCA_013735895.1
GCA_013736155.1
GCA_013737695.1
GCA_013738375.1
GCA_013739375.1
GCA_013739595.1
GCA_013739755.1
GCA_013740195.1
GCA_013740655.1
GCA_013740875.1
GCA_013741155.1
GCA_013741395.1
GCA_013742375.1
GCA_013742615.1
GCA_013743295.1
GCA_013743755.1
GCA_013743935.1
GCA_013744415.1
GCA_013744615.1
GCA_013744875.1
GCA_013745115.1
GCA_013745295.1
GCA_013745515.1
GCA_013746655.1
GCA_013747035.1
GCA_013747275.1
GCA_013747755.1
GCA_013748235.1
GCA_013748855.1
GCA_013749735.1
GCA_013750595.1
GCA_013751655.1
GCA_013751895.1

GCA_013752535.1
GCA_013752735.1
GCA_013781885.1
GCA_013781945.1
GCA_013781985.1
GCA_013782005.1
GCA_013782105.1
GCA_013782625.1
GCA_013783065.1
GCA_013783245.1
GCA_013784065.1
GCA_013784105.1
GCA_013784225.1
GCA_013784425.1
GCA_013784645.1
GCA_013785085.1
GCA_013785525.1
GCA_013786025.1
GCA_013786145.1
GCA_013786205.1
GCA_013786345.1
GCA_013786965.1
GCA_013787185.1
GCA_013790265.1
GCA_013791265.1
GCA_013791465.1
GCA_013791615.1
GCA_013792125.1
GCA_013792365.1
GCA_013792545.1
GCA_013796245.1
GCA_013796485.1
GCA_013797345.1
GCA_013797615.1
GCA_013798115.1
GCA_013798355.1
GCA_013798785.1
GCA_013799825.1
GCA_013800985.1
GCA_013801425.1
GCA_013801705.1
GCA_013801845.1
GCA_013805705.1
GCA_013811445.1
GCA_013811845.1
GCA_013812875.1
GCA_013813035.1
GCA_013813205.1
GCA_013813425.1
GCA_013814485.1
GCA_013815045.1
GCA_013816515.1
GCA_013816795.1
GCA_013819085.1

GCA_013819325.1
GCA_013819565.1
GCA_013820225.1
GCA_013820465.1
GCA_013820845.1
GCA_013821535.1
GCA_013821695.1
GCA_013822105.1
GCA_013822785.1
GCA_013822985.1
GCA_013825725.1
GCA_013826045.1
GCA_013826985.1
GCA_013828645.1
GCA_013829265.1
GCA_013834165.1
GCA_013834525.1
GCA_013834745.1
GCA_013836145.1
GCA_013836325.1
GCA_013836525.1
GCA_013836745.1
GCA_013838265.1
GCA_013838465.1
GCA_013873515.1
GCA_013873655.1
GCA_013873945.1
GCA_013874375.1
GCA_013874595.1
GCA_013874835.1
GCA_013875015.1
GCA_013882995.1
GCA_013883595.1
GCA_013886195.1
GCA_013886335.1
GCA_013889015.1
GCA_013889875.1
GCA_013890155.1
GCA_013890595.1
GCA_013892435.1
GCA_013893835.1
GCA_013894595.1
GCA_013895695.1
GCA_013899365.1
GCA_013899685.1
GCA_013899845.1
GCA_013923185.1
GCA_014023125.1
GCA_014023275.1
GCA_014041505.2
GCA_014041645.2
GCA_014041735.2
GCA_014041845.2
GCA_014041875.1

GCA_014041895.1
GCA_014041915.1
GCA_014041935.1
GCA_014041955.1
GCA_014041975.1
GCA_014041995.1
GCA_014042015.1
GCA_014042035.1
GCA_014048085.1
GCA_014048285.1
GCA_014048385.1
GCA_014050515.2
GCA_014054525.1
GCA_014054725.1
GCA_014054885.1
GCA_014054945.1
GCA_014054965.1
GCA_014054985.1
GCA_014055005.1
GCA_014055025.1
GCA_014058405.1
GCA_014058425.1
GCA_014058445.2
GCA_014058465.1
GCA_014058485.1
GCA_014058585.1
GCA_014058605.1
GCA_014058625.1
GCA_014058645.1
GCA_014058665.1
GCA_014058685.1
GCA_014058705.1
GCA_014068355.1
GCA_014068615.1
GCA_014069315.1
GCA_014069575.1
GCA_014069875.1
GCA_014070215.1
GCA_014070435.1
GCA_014070455.1
GCA_014075335.1
GCA_014075475.1
GCA_014075595.1
GCA_014075795.1
GCA_014075915.1
GCA_014075995.1
GCA_014076395.1
GCA_014076415.1
GCA_014076435.1
GCA_014076455.1
GCA_014076475.1
GCA_014076495.1
GCA_014076515.1
GCA_014076535.1

GCA_014076555.1
GCA_014076685.2
GCA_014081765.1
GCA_014081885.1
GCA_014082005.1
GCA_014083905.1
GCA_014083925.1
GCA_014083945.1
GCA_014083965.1
GCA_014083985.1
GCA_014084005.1
GCA_014084065.1
GCA_014084105.1
GCA_014084125.1
GCA_014107495.1
GCA_014107515.1
GCA_014107775.1
GCA_014108865.1
GCA_014109805.1
GCA_014109825.1
GCA_014109845.1
GCA_014109865.1
GCA_014117285.1
GCA_014117325.1
GCA_014117345.2
GCA_014117365.1
GCA_014117385.1
GCA_014117405.1
GCA_014117425.1
GCA_014117445.1
GCA_014117505.1
GCA_014123345.1
GCA_014126615.1
GCA_014126825.1
GCA_014127105.1
GCA_014127285.1
GCA_014127485.1
GCA_014131675.1
GCA_014131695.1
GCA_014131715.1
GCA_014131735.1
GCA_014131755.1
GCA_014131795.1
GCA_014151305.1
GCA_014151465.1
GCA_014151665.1
GCA_014151825.1
GCA_014151925.1
GCA_014152105.1
GCA_014152305.1
GCA_014152385.1
GCA_014152565.1
GCA_014155905.1
GCA_014156975.1

GCA_014157395.1
GCA_014158455.1
GCA_014159355.1
GCA_014160155.1
GCA_014161425.1
GCA_014161955.1
GCA_014161995.1
GCA_014162015.1
GCA_014162235.1
GCA_014162535.1
GCA_014162935.2
GCA_014163415.1
GCA_014163435.1
GCA_014163455.1
GCA_014163475.1
GCA_014163495.1
GCA_014168535.1
GCA_014168575.1
GCA_014168595.1
GCA_014168615.1
GCA_014168635.1
GCA_014168655.1
GCA_014168675.1
GCA_014168695.1
GCA_014168715.1
GCA_014168735.1
GCA_014168755.1
GCA_014168775.1
GCA_014168815.1
GCA_014168835.1
GCA_014168855.1
GCA_014168875.1
GCA_014168895.1
GCA_014168935.1
GCA_014168955.1
GCA_014168975.1
GCA_014168995.1
GCA_014169015.1
GCA_014169035.1
GCA_014169055.1
GCA_014169075.1
GCA_014169095.1
GCA_014169115.1
GCA_014169155.1
GCA_014169175.1
GCA_014169195.1
GCA_014169215.1
GCA_014169235.1
GCA_014169255.1
GCA_014169275.1
GCA_014169295.1
GCA_014169335.1
GCA_014169355.1
GCA_014169395.1

GCA_014169415.1
GCA_014169435.1
GCA_014169455.1
GCA_014169475.1
GCA_014169495.1
GCA_014169575.1
GCA_014169595.1
GCA_014169615.1
GCA_014169635.1
GCA_014169655.1
GCA_014169675.1
GCA_014169695.1
GCA_014169715.1
GCA_014169735.1
GCA_014169755.1
GCA_014169775.1
GCA_014169795.1
GCA_014169815.1
GCA_014169835.1
GCA_014169855.1
GCA_014169875.1
GCA_014169895.1
GCA_014169915.1
GCA_014169935.1
GCA_014169955.1
GCA_014169975.1
GCA_014169995.1
GCA_014170015.1
GCA_014170055.1
GCA_014170075.1
GCA_014170115.1
GCA_014170595.1
GCA_014170615.1
GCA_014170655.1
GCA_014170675.1
GCA_014170695.1
GCA_014170715.1
GCA_014170735.1
GCA_014170855.1
GCA_014170935.1
GCA_014170945.1
GCA_014170975.1
GCA_014171015.1
GCA_014171135.1
GCA_014171475.1
GCA_014171495.1
GCA_014171515.1
GCA_014171535.1
GCA_014171555.1
GCA_014171575.1
GCA_014171595.1
GCA_014171775.1
GCA_014171935.1
GCA_014180705.2

GCA_014189245.1
GCA_014189265.1
GCA_014189285.1
GCA_014189335.1
GCA_014189355.1
GCA_014189375.1
GCA_014189395.1
GCA_014189415.1
GCA_014189435.1
GCA_014189455.1
GCA_014189505.1
GCA_014189535.1
GCA_014189555.1
GCA_014189595.1
GCA_014189615.1
GCA_014189635.1
GCA_014191545.1
GCA_014191695.1
GCA_014191835.1
GCA_014192005.1
GCA_014192175.1
GCA_014192345.1
GCA_014192525.1
GCA_014192695.1
GCA_014192895.1
GCA_014211855.1
GCA_014211875.1
GCA_014211895.1
GCA_014211915.1
GCA_014211935.1
GCA_014211955.1
GCA_014211975.1
GCA_014211995.1
GCA_014216315.1
GCA_014216335.1
GCA_014217135.1
GCA_014217155.1
GCA_014217215.1
GCA_014217235.1
GCA_014217275.1
GCA_014217295.1
GCA_014217315.1
GCA_014217335.1
GCA_014217355.1
GCA_014217375.1
GCA_014217415.1
GCA_014217485.1
GCA_014217505.1
GCA_014217525.1
GCA_014217545.1
GCA_014217565.1
GCA_014217585.1
GCA_014217605.1
GCA_014217625.1

GCA_014217645.1
GCA_014217665.1
GCA_014217685.1
GCA_014217705.1
GCA_014217745.1
GCA_014217765.1
GCA_014217785.1
GCA_014217805.1
GCA_014217835.1
GCA_014217875.1
GCA_014217915.1
GCA_014217935.1
GCA_014217975.1
GCA_014217995.1
GCA_014218015.1
GCA_014218055.1
GCA_014218075.1
GCA_014218095.1
GCA_014218115.1
GCA_014218135.1
GCA_014218155.1
GCA_014218175.1
GCA_014218195.1
GCA_014218215.1
GCA_014218255.1
GCA_014218275.1
GCA_014218295.1
GCA_014218315.1
GCA_014218335.1
GCA_014218355.1
GCA_014233485.1
GCA_014235165.1
GCA_014235185.1
GCA_014236775.1
GCA_014236795.1
GCA_014236815.1
GCA_014236835.1
GCA_014236855.1
GCA_014249995.1
GCA_014250155.1
GCA_014250355.1
GCA_014250375.1
GCA_014250415.1
GCA_014250435.1
GCA_014250515.1
GCA_014250535.1
GCA_014251255.1
GCA_014251275.1
GCA_014251355.1
GCA_014251515.1
GCA_014251575.1
GCA_014251635.1
GCA_014251675.1
GCA_014251735.1

GCA_014251775.1
GCA_014251795.1
GCA_014251815.1
GCA_014251975.1
GCA_014251995.1
GCA_014252015.1
GCA_014252055.1
GCA_014252235.1
GCA_014252255.1
GCA_014252315.1
GCA_014252375.1
GCA_014252415.1
GCA_014262665.1
GCA_014262945.1
GCA_014263185.1
GCA_014263355.1
GCA_014263375.1
GCA_014263395.1
GCA_014267365.1
GCA_014267385.1
GCA_014268205.2
GCA_014268275.3
GCA_014268375.2
GCA_014268445.2
GCA_014268455.2
GCA_014268485.2
GCA_014268495.2
GCA_014268585.2
GCA_014268595.2
GCA_014268695.2
GCA_014268755.2
GCA_014268885.2
GCA_014268975.2
GCA_014269025.2
GCA_014269225.2
GCA_014274185.1
GCA_014274465.1
GCA_014274685.1
GCA_014279535.1
GCA_014279555.1
GCA_014279595.1
GCA_014279615.1
GCA_014279635.1
GCA_014279655.1
GCA_014279755.1
GCA_014279775.1
GCA_014279795.1
GCA_014279815.1
GCA_014279835.1
GCA_014279855.1
GCA_014279875.1
GCA_014279895.1
GCA_014279955.1
GCA_014279975.1

GCA_014280035.1
GCA_014280075.1
GCA_014280095.1
GCA_014280115.1
GCA_014280175.1
GCA_014280195.1
GCA_014280215.1
GCA_014280235.1
GCA_014280435.1
GCA_014280455.1
GCA_014287515.2
GCA_014295015.1
GCA_014295035.1
GCA_014295215.1
GCA_014295235.1
GCA_014295255.1
GCA_014295275.1
GCA_014295295.1
GCA_014295315.1
GCA_014295415.1
GCA_014297575.1
GCA_014297595.1
GCA_014298115.1
GCA_014301985.1
GCA_014302175.1
GCA_014302295.1
GCA_014302495.1
GCA_014302635.1
GCA_014302835.1
GCA_014302995.1
GCA_014303395.1
GCA_014303495.1
GCA_014303735.1
GCA_014303795.1
GCA_014303955.1
GCA_014304635.1
GCA_014304675.1
GCA_014304695.1
GCA_014304775.1
GCA_014304795.1
GCA_014304815.1
GCA_014304835.1
GCA_014322245.1
GCA_014323565.1
GCA_014334075.1
GCA_014334095.1
GCA_014334115.1
GCA_014334135.1
GCA_014334155.1
GCA_014334175.1
GCA_014334195.1
GCA_014334235.1
GCA_014334255.1
GCA_014334275.1

GCA_014334295.1
GCA_014334375.1
GCA_014334715.1
GCA_014334735.1
GCA_014337155.1
GCA_014337175.1
GCA_014337195.1
GCA_014337215.1
GCA_014337235.1
GCA_014337255.1
GCA_014338425.3
GCA_014338485.1
GCA_014338505.1
GCA_014338525.1
GCA_014352855.1
GCA_014352875.1
GCA_014352895.1
GCA_014352915.1
GCA_014352935.1
GCA_014352955.1
GCA_014353015.1
GCA_014353035.1
GCA_014353055.1
GCA_014357995.1
GCA_014358035.1
GCA_014361115.1
GCA_014361285.1
GCA_014394705.1
GCA_014394985.2
GCA_014395225.1
GCA_014395425.1
GCA_014395645.1
GCA_014395785.1
GCA_014395975.1
GCA_014396165.1
GCA_014396385.1
GCA_014396585.1
GCA_014396785.1
GCA_014396975.1
GCA_014397115.1
GCA_014397255.1
GCA_014397415.1
GCA_014400405.1
GCA_014466955.1
GCA_014466985.1
GCA_014467015.1
GCA_014467035.1
GCA_014467055.1
GCA_014467075.1
GCA_014467095.1
GCA_014483895.1
GCA_014489335.1
GCA_014489355.1
GCA_014489395.1

GCA_014489415.1
GCA_014489455.1
GCA_014489475.1
GCA_014489495.1
GCA_014489515.1
GCA_014489535.1
GCA_014489555.1
GCA_014489575.1
GCA_014489595.1
GCA_014489615.1
GCA_014489635.1
GCA_014489955.1
GCA_014489975.1
GCA_014490015.1
GCA_014490035.1
GCA_014490055.1
GCA_014490515.1
GCA_014490535.1
GCA_014490555.1
GCA_014490575.1
GCA_014490595.1
GCA_014490645.1
GCA_014490665.1
GCA_014490685.2
GCA_014490785.1
GCA_014490805.1
GCA_014490825.1
GCA_014490845.1
GCA_014495705.1
GCA_014495725.1
GCA_014495745.1
GCA_014495765.1
GCA_014495785.1
GCA_014495805.1
GCA_014495825.1
GCA_014495845.1
GCA_014495865.1
GCA_014495885.1
GCA_014495905.1
GCA_014495925.1
GCA_014495945.1
GCA_014495965.1
GCA_014495985.1
GCA_014521465.1
GCA_014522205.1
GCA_014522225.1
GCA_014522245.1
GCA_014522265.1
GCA_014522285.1
GCA_014522305.1
GCA_014522325.1
GCA_014522345.1
GCA_014524485.1
GCA_014524505.1

GCA_014524525.1
GCA_014524545.1
GCA_014524565.1
GCA_014524585.1
GCA_014524605.1
GCA_014524625.1
GCA_014524645.1
GCA_014529625.2
GCA_014529665.2
GCA_014529675.2
GCA_014541045.1
GCA_014541205.1
GCA_014595995.2
GCA_014596425.2
GCA_014605875.1
GCA_014607515.1
GCA_014607535.1
GCA_014607555.1
GCA_014607575.1
GCA_014621655.1
GCA_014621675.1
GCA_014621695.1
GCA_014621755.1
GCA_014621775.1
GCA_014622645.1
GCA_014622665.1
GCA_014622685.1
GCA_014622705.1
GCA_014622725.1
GCA_014622745.1
GCA_014622925.2
GCA_014622945.2
GCA_014622965.1
GCA_014622985.1
GCA_014623185.1
GCA_014623205.1
GCA_014623225.1
GCA_014623245.1
GCA_014623405.1
GCA_014623425.1
GCA_014623465.1
GCA_014656565.1
GCA_014656585.1
GCA_014656605.1
GCA_014656625.1
GCA_014672695.1
GCA_014672715.2
GCA_014672735.1
GCA_014672755.1
GCA_014672775.1
GCA_014672795.1
GCA_014672815.1
GCA_014672835.1
GCA_014672855.1

GCA_014672875.1
GCA_014673475.1
GCA_014673495.1
GCA_014678725.1
GCA_014678925.1
GCA_014679145.1
GCA_014679325.1
GCA_014679335.1
GCA_014679505.1
GCA_014679585.1
GCA_014679705.1
GCA_014679775.1
GCA_014680085.1
GCA_014681765.1
GCA_014692735.1
GCA_014692875.1
GCA_014696255.2
GCA_014696415.2
GCA_014696535.2
GCA_014696685.2
GCA_014696835.2
GCA_014696975.2
GCA_014697095.1
GCA_014697215.1
GCA_014700555.1
GCA_014701095.1
GCA_014701115.1
GCA_014701195.1
GCA_014701215.1
GCA_014701235.1
GCA_014701265.1
GCA_014702225.1
GCA_014702245.1
GCA_014705785.1
GCA_014705805.1
GCA_014705825.1
GCA_014705845.1
GCA_014705865.1
GCA_014705885.1
GCA_014705905.1
GCA_014705925.2
GCA_014705945.2
GCA_014705965.1
GCA_014705985.1
GCA_014706535.1
GCA_014706555.1
GCA_014706575.1
GCA_014706595.1
GCA_014706615.1
GCA_014717515.1
GCA_014717695.1
GCA_014717935.1
GCA_014718155.1
GCA_014718355.1

GCA_014718535.1
GCA_014718755.1
GCA_014718955.1
GCA_014719215.1
GCA_014719435.1
GCA_014719655.1
GCA_014719835.1
GCA_014720075.1
GCA_014720295.1
GCA_014720495.1
GCA_014720715.1
GCA_014720855.1
GCA_014721075.1
GCA_014721215.1
GCA_014721415.1
GCA_014721575.1
GCA_014721815.1
GCA_014722015.1
GCA_014722275.1
GCA_014722535.1
GCA_014722775.1
GCA_014723035.1
GCA_014723055.1
GCA_014723075.1
GCA_014723095.1
GCA_014723115.1
GCA_014723135.1
GCA_014723155.1
GCA_014723175.1
GCA_014723195.1
GCA_014723215.1
GCA_014725695.1
GCA_014725715.1
GCA_014725735.1
GCA_014725775.1
GCA_014725795.1
GCA_014725815.1
GCA_014725915.1
GCA_014764305.1
GCA_014764325.1
GCA_014764345.1
GCA_014764365.1
GCA_014764385.1
GCA_014764405.1
GCA_014764425.1
GCA_014764445.1
GCA_014770185.1
GCA_014770315.1
GCA_014770435.1
GCA_014770655.1
GCA_014770895.1
GCA_014771055.1
GCA_014771275.1
GCA_014771415.1

GCA_014775655.1
GCA_014775675.1
GCA_014775695.1
GCA_014779555.2
GCA_014784055.1
GCA_014788525.1
GCA_014788765.1
GCA_014788985.1
GCA_014789145.1
GCA_014789345.1
GCA_014789585.1
GCA_014789785.1
GCA_014791945.1
GCA_014792065.1
GCA_014792105.1
GCA_014792125.1
GCA_014792805.1
GCA_014805785.2
GCA_014805805.1
GCA_014805825.1
GCA_014825845.1
GCA_014826015.1
GCA_014826045.1
GCA_014826095.1
GCA_014838715.1
GCA_014840915.2
GCA_014840935.1
GCA_014840955.1
GCA_014840975.1
GCA_014840995.1
GCA_014841015.1
GCA_014841035.1
GCA_014841055.1
GCA_014841075.1
GCA_014842815.3
GCA_014843055.1
GCA_014843075.1
GCA_014843095.1
GCA_014843115.1
GCA_014843135.1
GCA_014843155.1
GCA_014843175.1
GCA_014843195.1
GCA_014843215.1
GCA_014843235.1
GCA_014843255.1
GCA_014843275.1
GCA_014843295.1
GCA_014843315.1
GCA_014843335.1
GCA_014843375.1
GCA_014843395.1
GCA_014843515.1
GCA_014843995.1

GCA_014844015.1
GCA_014844035.1
GCA_014844055.1
GCA_014844075.1
GCA_014844095.1
GCA_014844115.1
GCA_014844155.1
GCA_014844195.1
GCA_014844215.1
GCA_014844235.1
GCA_014844255.1
GCA_014844275.1
GCA_014844295.1
GCA_014844315.1
GCA_014844335.1
GCA_014844355.1
GCA_014844375.1
GCA_014844395.1
GCA_014844415.1
GCA_014844435.1
GCA_014844455.1
GCA_014844475.1
GCA_014844495.1
GCA_014844515.1
GCA_014844535.1
GCA_014844555.1
GCA_014844575.1
GCA_014844595.1
GCA_014844615.1
GCA_014844635.1
GCA_014844655.1
GCA_014844675.1
GCA_014844695.1
GCA_014844735.1
GCA_014844755.1
GCA_014844775.1
GCA_014844995.1
GCA_014852565.2
GCA_014854555.1
GCA_014854575.1
GCA_014854595.1
GCA_014854615.1
GCA_014854635.1
GCA_014854655.1
GCA_014854675.1
GCA_014854695.1
GCA_014854715.1
GCA_014854735.1
GCA_014854755.1
GCA_014854775.1
GCA_014854795.1
GCA_014854815.1
GCA_014854835.1
GCA_014855475.1

GCA_014856545.2
GCA_014857065.1
GCA_014858525.1
GCA_014858545.1
GCA_014858565.1
GCA_014858815.1
GCA_014858865.1
GCA_014858895.1
GCA_014858915.1
GCA_014858935.1
GCA_014859005.1
GCA_014863505.1
GCA_014863545.1
GCA_014863585.1
GCA_014863605.1
GCA_014863625.1
GCA_014863645.1
GCA_014863665.1
GCA_014863685.1
GCA_014863705.1
GCA_014863725.1
GCA_014863745.1
GCA_014863765.1
GCA_014863785.1
GCA_014863805.1
GCA_014863825.1
GCA_014863845.1
GCA_014872735.1
GCA_014873095.1
GCA_014874555.1
GCA_014874575.1
GCA_014874615.1
GCA_014876755.1
GCA_014879275.1
GCA_014879295.1
GCA_014879315.1
GCA_014879975.1
GCA_014883875.1
GCA_014883895.1
GCA_014883915.1
GCA_014883935.1
GCA_014883955.1
GCA_014884405.1
GCA_014884425.1
GCA_014884445.1
GCA_014884465.1
GCA_014884485.1
GCA_014884505.1
GCA_014884525.1
GCA_014884545.1
GCA_014884565.1
GCA_014884585.1
GCA_014884605.1
GCA_014884625.1

GCA_014884645.1
GCA_014884665.1
GCA_014884685.1
GCA_014884705.1
GCA_014884725.1
GCA_014884745.1
GCA_014892695.1
GCA_014893095.1
GCA_014896945.1
GCA_014898075.1
GCA_014898095.1
GCA_014898115.1
GCA_014898135.1
GCA_014898155.1
GCA_014898175.1
GCA_014898195.1
GCA_014898215.1
GCA_014898235.1
GCA_014899185.1
GCA_014899205.1
GCA_014899225.1
GCA_014899245.1
GCA_014899265.1
GCA_014899285.1
GCA_014899305.1
GCA_014899325.1
GCA_014899345.1
GCA_014899365.1
GCA_014899385.1
GCA_014899405.1
GCA_014899425.1
GCA_014899445.1
GCA_014899465.1
GCA_014899485.1
GCA_014899505.1
GCA_014899525.1
GCA_014899545.1
GCA_014899565.1
GCA_014899585.1
GCA_014899605.1
GCA_014899625.1
GCA_014899645.1
GCA_014899665.1
GCA_014899685.1
GCA_014899705.1
GCA_014899725.1
GCA_014899745.1
GCA_014899765.1
GCA_014899785.1
GCA_014899805.1
GCA_014899825.1
GCA_014899845.1
GCA_014899865.1
GCA_014899885.1

GCA_014899905.1
GCA_014899925.1
GCA_014899945.1
GCA_014899965.1
GCA_014899985.1
GCA_014900005.1
GCA_014900055.1
GCA_014900075.1
GCA_014900095.1
GCA_014900115.1
GCA_014900135.1
GCA_014900155.1
GCA_014900175.1
GCA_014900195.1
GCA_014900215.1
GCA_014900235.1
GCA_014900255.1
GCA_014900275.1
GCA_014900295.1
GCA_014900315.1
GCA_014900335.1
GCA_014900355.1
GCA_014900375.1
GCA_014900395.1
GCA_014900415.1
GCA_014900435.1
GCA_014900455.1
GCA_014900475.1
GCA_014900495.1
GCA_014900515.1
GCA_014900535.1
GCA_014900555.1
GCA_014900595.1
GCA_014900615.1
GCA_014900635.1
GCA_014900655.1
GCA_014900675.1
GCA_014900695.1
GCA_014900715.1
GCA_014900735.1
GCA_014900755.1
GCA_014900775.1
GCA_014900795.1
GCA_014900815.1
GCA_014900835.1
GCA_014900855.1
GCA_014900895.1
GCA_014900965.1
GCA_014901035.1
GCA_014901055.1
GCA_014901075.1
GCA_014901095.1
GCA_014901115.1
GCA_014901135.1

GCA_014901155.1
GCA_014904135.2
GCA_014904145.2
GCA_014904175.2
GCA_014904185.2
GCA_014904215.2
GCA_014904235.2
GCA_014904265.2
GCA_014904315.2
GCA_014904435.2
GCA_014905035.1
GCA_014905055.1
GCA_014905075.1
GCA_014905095.1
GCA_014905115.1
GCA_014905135.1
GCA_014905815.1
GCA_014905885.1
GCA_014905905.1
GCA_014906145.1
GCA_014906165.1
GCA_014906185.1
GCA_014906205.1
GCA_014926385.1
GCA_014926585.1
GCA_014930875.1
GCA_014930895.1
GCA_014930915.1
GCA_014930935.1
GCA_014930955.1
GCA_014930975.1
GCA_014930995.1
GCA_014931015.1
GCA_014931035.1
GCA_014931055.1
GCA_014931075.1
GCA_014931095.1
GCA_014931155.1
GCA_014931175.1
GCA_014931215.1
GCA_014931255.1
GCA_014931275.1
GCA_014931295.1
GCA_014931315.1
GCA_014931335.1
GCA_014931355.1
GCA_014931375.1
GCA_014931395.1
GCA_014931415.1
GCA_014931435.1
GCA_014931455.1
GCA_014931475.1
GCA_014931495.1
GCA_014931515.1

GCA_014931565.1
GCA_014931585.1
GCA_014931605.1
GCA_014931645.1
GCA_014931665.1
GCA_014931695.1
GCA_014931715.1
GCA_014946775.2
GCA_014946825.2
GCA_014960965.1
GCA_014961145.1
GCA_014961485.1
GCA_014961685.1
GCA_014961825.1
GCA_014961985.1
GCA_014962425.1
GCA_014962565.1
GCA_014983505.1
GCA_014983645.1
GCA_014983945.1
GCA_014984185.1
GCA_014987905.1
GCA_015022235.2
GCA_015024285.2
GCA_015033265.1
GCA_015034585.1
GCA_015034605.1
GCA_015040095.1
GCA_015040335.1
GCA_015040615.1
GCA_015052745.1
GCA_015052765.1
GCA_015052785.1
GCA_015052805.1
GCA_015052825.1
GCA_015074805.1
GCA_015074825.1
GCA_015074845.1
GCA_015074865.1
GCA_015074885.1
GCA_015074905.1
GCA_015074925.1
GCA_015074945.1
GCA_015097055.1
GCA_015097275.1
GCA_015097455.1
GCA_015097735.1
GCA_015097935.1
GCA_015098135.1
GCA_015098255.1
GCA_015098475.1
GCA_015098595.1
GCA_015098755.1
GCA_015099395.1

GCA_015099595.1
GCA_015100215.1
GCA_015100395.1
GCA_015101725.1
GCA_015101885.1
GCA_015102035.1
GCA_015102215.1
GCA_015134075.1
GCA_015134315.1
GCA_015134335.1
GCA_015134355.1
GCA_015134395.1
GCA_015134415.1
GCA_015134435.1
GCA_015134695.1
GCA_015134935.1
GCA_015135075.1
GCA_015135215.1
GCA_015135355.1
GCA_015135515.1
GCA_015135595.1
GCA_015135855.1
GCA_015136055.1
GCA_015136315.1
GCA_015136515.1
GCA_015136715.1
GCA_015136895.1
GCA_015137095.1
GCA_015137255.1
GCA_015137465.1
GCA_015137655.1
GCA_015137995.1
GCA_015138175.1
GCA_015138375.1
GCA_015138575.1
GCA_015138775.1
GCA_015138955.1
GCA_015139155.1
GCA_015139375.1
GCA_015139575.1
GCA_015139735.1
GCA_015139955.1
GCA_015140235.1
GCA_015140255.1
GCA_015140455.1
GCA_015159555.1
GCA_015159575.1
GCA_015159595.1
GCA_015159615.2
GCA_015159775.2
GCA_015160855.1
GCA_015160875.1
GCA_015160895.1
GCA_015160915.1

GCA_015167415.1
GCA_015167655.1
GCA_015168335.1
GCA_015168595.1
GCA_015168795.1
GCA_015169015.1
GCA_015169395.1
GCA_015169775.1
GCA_015169995.1
GCA_015172915.1
GCA_015190465.1
GCA_015190655.1
GCA_015191005.1
GCA_015200345.1
GCA_015200565.1
GCA_015209725.1
GCA_015209745.1
GCA_015209765.1
GCA_015209785.1
GCA_015217905.1
GCA_015218165.1
GCA_015218525.1
GCA_015218885.1
GCA_015219785.1
GCA_015219805.1
GCA_015219825.1
GCA_015219885.1
GCA_015219905.1
GCA_015219925.1
GCA_015220695.1
GCA_015221735.2
GCA_015223545.3
GCA_015224645.1
GCA_015224665.2
GCA_015238555.1
GCA_015238575.1
GCA_015238615.1
GCA_015238635.1
GCA_015238655.1
GCA_015238895.1
GCA_015239135.1
GCA_015239295.1
GCA_015239615.1
GCA_015239775.1
GCA_015240065.1
GCA_015240255.1
GCA_015240635.1
GCA_015240855.1
GCA_015240995.1
GCA_015241115.1
GCA_015243235.1
GCA_015243435.1
GCA_015243575.1
GCA_015243835.1

GCA_015244035.1
GCA_015244315.1
GCA_015244435.1
GCA_015245165.1
GCA_015256915.3
GCA_015257555.2
GCA_015257785.3
GCA_015257795.3
GCA_015265455.1
GCA_015265475.1
GCA_015265495.2
GCA_015277495.1
GCA_015277515.1
GCA_015277535.1
GCA_015277555.1
GCA_015277575.1
GCA_015277635.1
GCA_015277755.1
GCA_015277775.1
GCA_015277815.1
GCA_015277835.1
GCA_015277855.1
GCA_015277875.1
GCA_015277895.1
GCA_015277915.1
GCA_015277935.1
GCA_015277955.1
GCA_015277975.1
GCA_015277995.1
GCA_015278015.1
GCA_015278035.1
GCA_015278055.1
GCA_015278075.1
GCA_015278095.1
GCA_015283665.1
GCA_015283885.1
GCA_015284065.1
GCA_015284185.1
GCA_015284345.1
GCA_015284485.1
GCA_015284665.1
GCA_015284925.1
GCA_015285145.1
GCA_015285425.1
GCA_015285645.1
GCA_015285825.1
GCA_015286005.1
GCA_015286025.1
GCA_015286225.1
GCA_015286445.1
GCA_015286725.1
GCA_015286965.1
GCA_015287155.1
GCA_015287425.1

GCA_015287605.1
GCA_015287805.1
GCA_015288045.1
GCA_015288205.1
GCA_015288445.1
GCA_015288605.1
GCA_015288785.1
GCA_015288985.1
GCA_015289145.1
GCA_015289325.1
GCA_015289545.1
GCA_015289765.1
GCA_015289985.1
GCA_015290145.1
GCA_015290365.1
GCA_015290565.1
GCA_015290725.1
GCA_015290925.1
GCA_015290945.1
GCA_015290965.1
GCA_015290985.1
GCA_015291305.1
GCA_015291565.1
GCA_015291625.1
GCA_015291645.1
GCA_015291665.1
GCA_015291685.1
GCA_015291705.1
GCA_015291725.1
GCA_015291745.1
GCA_015291765.1
GCA_015291785.1
GCA_015291805.1
GCA_015291825.1
GCA_015291845.1
GCA_015291865.1
GCA_015291885.1
GCA_015291905.1
GCA_015291925.1
GCA_015325925.1
GCA_015326295.1
GCA_015326725.1
GCA_015326965.1
GCA_015327065.1
GCA_015336085.1
GCA_015336265.1
GCA_015336465.1
GCA_015336695.1
GCA_015336865.1
GCA_015337085.2
GCA_015337285.1
GCA_015337445.1
GCA_015337645.1
GCA_015337825.2

GCA_015338045.2
GCA_015338205.1
GCA_015338385.1
GCA_015351935.2
GCA_015351965.2
GCA_015352015.2
GCA_015352045.2
GCA_015352115.2
GCA_015352155.2
GCA_015353075.1
GCA_015353095.1
GCA_015353115.1
GCA_015353135.1
GCA_015355835.1
GCA_015355855.1
GCA_015355875.1
GCA_015355895.1
GCA_015355915.1
GCA_015355935.1
GCA_015355955.1
GCA_015355975.1
GCA_015355995.1
GCA_015356015.1
GCA_015356035.1
GCA_015356055.1
GCA_015356075.1
GCA_015356095.1
GCA_015356115.1
GCA_015356815.2
GCA_015377145.2
GCA_015377485.1
GCA_015377505.1
GCA_015377525.1
GCA_015377585.1
GCA_015377745.1
GCA_015377765.1
GCA_015377785.1
GCA_015377805.1
GCA_015475555.1
GCA_015475575.1
GCA_015475595.1
GCA_015475615.1
GCA_015475915.1
GCA_015475935.1
GCA_015475955.1
GCA_015475975.1
GCA_015475995.1
GCA_015476015.1
GCA_015476035.1
GCA_015476055.1
GCA_015476075.1
GCA_015476095.1
GCA_015476115.1
GCA_015476135.1

GCA_015476155.1
GCA_015476175.1
GCA_015476195.1
GCA_015476235.1
GCA_015476275.1
GCA_015476295.1
GCA_015482585.1
GCA_015482775.1
GCA_015482805.1
GCA_015482825.1
GCA_015500055.1
GCA_015500075.1
GCA_015500095.1
GCA_015508545.2
GCA_015508805.1
GCA_015509005.1
GCA_015509285.1
GCA_015533855.1
GCA_015534735.1
GCA_015534755.1
GCA_015534775.1
GCA_015534795.1
GCA_015534815.1
GCA_015534835.1
GCA_015534935.1
GCA_015534955.1
GCA_015534975.1
GCA_015534995.1
GCA_015535015.1
GCA_015535055.1
GCA_015535655.1
GCA_015535675.1
GCA_015565735.1
GCA_015565955.1
GCA_015566135.1
GCA_015571535.1
GCA_015571555.1
GCA_015571575.1
GCA_015571595.1
GCA_015571615.1
GCA_015571635.1
GCA_015571655.1
GCA_015571675.1
GCA_015571695.1
GCA_015571715.1
GCA_015571735.1
GCA_015571755.1
GCA_015571775.1
GCA_015571795.1
GCA_015571815.1
GCA_015571835.1
GCA_015571855.1
GCA_015571875.1
GCA_015571895.1

GCA_015571915.1
GCA_015571935.1
GCA_015571955.1
GCA_015571975.1
GCA_015582655.1
GCA_015583955.1
GCA_015594925.2
GCA_015601345.1
GCA_015601545.1
GCA_015602625.1
GCA_015602645.1
GCA_015602665.1
GCA_015602685.1
GCA_015602705.1
GCA_015624425.1
GCA_015624465.1
GCA_015624485.1
GCA_015624505.1
GCA_015643545.1
GCA_015644725.1
GCA_015644745.1
GCA_015644765.1
GCA_015649805.1
GCA_015650045.1
GCA_015654125.1
GCA_015654165.1
GCA_015654185.1
GCA_015654205.1
GCA_015654285.1
GCA_015655285.1
GCA_015679185.1
GCA_015679205.1
GCA_015679225.1
GCA_015679245.1
GCA_015679265.1
GCA_015679285.1
GCA_015679665.1
GCA_015679705.1
GCA_015679735.1
GCA_015679775.1
GCA_015679795.1
GCA_015679815.1
GCA_015679845.1
GCA_015679865.1
GCA_015679885.1
GCA_015679905.1
GCA_015679925.1
GCA_015679945.1
GCA_015679965.1
GCA_015679985.1
GCA_015680005.1
GCA_015680025.1
GCA_015680045.1
GCA_015680915.2

GCA_015689015.1
GCA_015689035.1
GCA_015689075.1
GCA_015689095.1
GCA_015689115.1
GCA_015689175.1
GCA_015689195.1
GCA_015689335.1
GCA_015689355.1
GCA_015689375.1
GCA_015689395.1
GCA_015689415.1
GCA_015689435.1
GCA_015689455.1
GCA_015689475.1
GCA_015689495.1
GCA_015693865.1
GCA_015693885.1
GCA_015693905.1
GCA_015693925.1
GCA_015693945.1
GCA_015693965.1
GCA_015693985.1
GCA_015694005.1
GCA_015694025.1
GCA_015694045.1
GCA_015694065.1
GCA_015694085.1
GCA_015697215.1
GCA_015697465.1
GCA_015697605.1
GCA_015697645.1
GCA_015697665.1
GCA_015697685.1
GCA_015697745.1
GCA_015697765.1
GCA_015697785.1
GCA_015697805.1
GCA_015697825.1
GCA_015697925.1
GCA_015698285.1
GCA_015698325.1
GCA_015698345.1
GCA_015698365.1
GCA_015698385.1
GCA_015708295.1
GCA_015708605.1
GCA_015708635.1
GCA_015708655.1
GCA_015708675.1
GCA_015709185.1
GCA_015709495.1
GCA_015709575.1
GCA_015709595.1

GCA_015709615.1
GCA_015709635.1
GCA_015709675.1
GCA_015709715.1
GCA_015710575.2
GCA_015710815.1
GCA_015710975.1
GCA_015710995.1
GCA_015711015.1
GCA_015711475.2
GCA_015732435.1
GCA_015732455.1
GCA_015732475.1
GCA_015732535.1
GCA_015732555.1
GCA_015732575.1
GCA_015732665.1
GCA_015732685.1
GCA_015767475.1
GCA_015767495.1
GCA_015767515.1
GCA_015767555.1
GCA_015767575.1
GCA_015767595.1
GCA_015767615.1
GCA_015767635.1
GCA_015767655.1
GCA_015767695.1
GCA_015767715.1
GCA_015767735.1
GCA_015767755.1
GCA_015767775.1
GCA_015767795.1
GCA_015767815.1
GCA_015767835.1
GCA_015767855.1
GCA_015767875.1
GCA_015767895.1
GCA_015767915.1
GCA_015767935.1
GCA_015774815.1
GCA_015774835.1
GCA_015774855.1
GCA_015774875.1
GCA_015774895.1
GCA_015774955.1
GCA_015775135.1
GCA_015775275.1
GCA_015775515.1
GCA_015775755.1
GCA_015775975.1
GCA_015779285.1
GCA_015832035.1
GCA_015832055.1

GCA_015832075.1
GCA_015832095.1
GCA_015832115.1
GCA_015832135.1
GCA_015832305.1
GCA_015832325.1
GCA_015838875.1
GCA_015839035.1
GCA_015839235.1
GCA_015839415.1
GCA_015839575.1
GCA_015839775.1
GCA_015839915.1
GCA_015840115.1
GCA_015840335.1
GCA_015840525.1
GCA_015840715.1
GCA_015840915.1
GCA_015841135.1
GCA_015841355.1
GCA_015843075.1
GCA_015870765.1
GCA_015870965.1
GCA_015871245.1
GCA_015871485.1
GCA_015908885.1
GCA_015908985.1
GCA_015910145.1
GCA_015910165.1
GCA_015910185.1
GCA_015910205.1
GCA_015910245.1
GCA_015910265.1
GCA_015910285.1
GCA_015910325.1
GCA_015910345.1
GCA_015910365.1
GCA_015910385.1
GCA_015910445.1
GCA_015910475.1
GCA_015992245.1
GCA_015992265.1
GCA_015992285.1
GCA_015992305.1
GCA_015992325.1
GCA_015992345.1
GCA_015992365.1
GCA_015992385.1
GCA_015992405.1
GCA_015992425.1
GCA_015992445.1
GCA_015999365.1
GCA_015999385.1
GCA_015999405.1

GCA_015999425.1
GCA_015999445.1
GCA_015999465.1
GCA_015999505.1
GCA_015999565.1
GCA_015999605.1
GCA_015999625.1
GCA_016025055.1
GCA_016025375.1
GCA_016025395.1
GCA_016025415.2
GCA_016025435.1
GCA_016025875.1
GCA_016026035.1
GCA_016026055.1
GCA_016026195.1
GCA_016026215.1
GCA_016026235.1
GCA_016026335.1
GCA_016026375.1
GCA_016026395.1
GCA_016026415.1
GCA_016026535.1
GCA_016026555.1
GCA_016026575.1
GCA_016026595.1
GCA_016026615.1
GCA_016026635.1
GCA_016026675.1
GCA_016026695.1
GCA_016026715.1
GCA_016026735.1
GCA_016026835.1
GCA_016026855.1
GCA_016026875.1
GCA_016026895.1
GCA_016026915.1
GCA_016026975.1
GCA_016026995.1
GCA_016027015.1
GCA_016027035.1
GCA_016027055.1
GCA_016027075.1
GCA_016027095.1
GCA_016027115.1
GCA_016027135.1
GCA_016027155.1
GCA_016027375.1
GCA_016027415.1
GCA_016027435.1
GCA_016027455.1
GCA_016027575.1
GCA_016027595.1
GCA_016027615.1

GCA_016027675.1
GCA_016027695.1
GCA_016027715.1
GCA_016027775.1
GCA_016027815.1
GCA_016027835.1
GCA_016027855.1
GCA_016027975.1
GCA_016028175.1
GCA_016028235.1
GCA_016028255.1
GCA_016028275.1
GCA_016028295.1
GCA_016028435.1
GCA_016028475.1
GCA_016028495.1
GCA_016028515.1
GCA_016028635.1
GCA_016028655.1
GCA_016028675.1
GCA_016028695.1
GCA_016028715.1
GCA_016028735.1
GCA_016028755.1
GCA_016028775.1
GCA_016028795.1
GCA_016028815.1
GCA_016028835.1
GCA_016028855.1
GCA_016064595.1
GCA_016064815.1
GCA_016065215.1
GCA_016065415.1
GCA_016065635.1
GCA_016066025.1
GCA_016066295.1
GCA_016066475.1
GCA_016066695.1
GCA_016066915.1
GCA_016067215.1
GCA_016067395.1
GCA_016067635.1
GCA_016067835.1
GCA_016070795.1
GCA_016070975.1
GCA_016071195.1
GCA_016071375.1
GCA_016071555.1
GCA_016071735.1
GCA_016105505.1
GCA_016105665.1
GCA_016105885.1
GCA_016107485.1
GCA_016107505.1

GCA_016110345.1
GCA_016117655.1
GCA_016126655.1
GCA_016126675.1
GCA_016126695.1
GCA_016126715.1
GCA_016126735.1
GCA_016126755.1
GCA_016126775.1
GCA_016126795.1
GCA_016126815.2
GCA_016126835.1
GCA_016126855.1
GCA_016126895.1
GCA_016126915.1
GCA_016126935.1
GCA_016126955.1
GCA_016126975.1
GCA_016126995.1
GCA_016127015.1
GCA_016127035.1
GCA_016127055.1
GCA_016127075.1
GCA_016127095.1
GCA_016127115.1
GCA_016127135.1
GCA_016127155.1
GCA_016127175.1
GCA_016127195.1
GCA_016127215.1
GCA_016127235.1
GCA_016127255.1
GCA_016127275.1
GCA_016127295.1
GCA_016127315.1
GCA_016127335.1
GCA_016127355.1
GCA_016127395.1
GCA_016127415.1
GCA_016127435.1
GCA_016127455.1
GCA_016127475.1
GCA_016127495.1
GCA_016127515.1
GCA_016127535.1
GCA_016127555.1
GCA_016127575.1
GCA_016127595.1
GCA_016127615.1
GCA_016127635.1
GCA_016127655.1
GCA_016127675.1
GCA_016127695.1
GCA_016127715.1

GCA_016127735.1
GCA_016127755.1
GCA_016127775.1
GCA_016127795.1
GCA_016127815.1
GCA_016127835.1
GCA_016127855.1
GCA_016127875.1
GCA_016127895.1
GCA_016127915.1
GCA_016127935.1
GCA_016127955.1
GCA_016127975.1
GCA_016127995.1
GCA_016128015.1
GCA_016128035.1
GCA_016128055.1
GCA_016128075.1
GCA_016128095.1
GCA_016128115.1
GCA_016128135.1
GCA_016128175.1
GCA_016128195.1
GCA_016128215.1
GCA_016128235.1
GCA_016128275.1
GCA_016128295.1
GCA_016128315.1
GCA_016132425.1
GCA_016132445.1
GCA_016132505.1
GCA_016132545.1
GCA_016134045.1
GCA_016134915.1
GCA_016134935.2
GCA_016237705.1
GCA_016237985.1
GCA_016250455.1
GCA_016267665.1
GCA_016313165.1
GCA_016313185.1
GCA_016313205.1
GCA_016313225.1
GCA_016350005.1
GCA_016350065.1
GCA_016350085.1
GCA_016350105.1
GCA_016350125.1
GCA_016350165.1
GCA_016403065.1
GCA_016403085.1
GCA_016403105.1
GCA_016403125.1
GCA_016403145.1

GCA_016403625.2
GCA_016403675.2
GCA_016403875.2
GCA_016403975.2
GCA_016404015.2
GCA_016404165.2
GCA_016404325.2
GCA_016404405.2
GCA_016404485.2
GCA_016406035.3
GCA_016406105.1
GCA_016406125.1
GCA_016406145.1
GCA_016406165.1
GCA_016406185.1
GCA_016406205.1
GCA_016406225.1
GCA_016406285.1
GCA_016406305.1
GCA_016406325.1
GCA_016406345.1
GCA_016406365.1
GCA_016406385.1
GCA_016406405.1
GCA_016406425.1
GCA_016406445.1
GCA_016406465.1
GCA_016406485.1
GCA_016406505.1
GCA_016406525.1
GCA_016406545.1
GCA_016406565.1
GCA_016406585.1
GCA_016406605.1
GCA_016406625.1
GCA_016406645.1
GCA_016406665.1
GCA_016406685.1
GCA_016413685.1
GCA_016413705.1
GCA_016413725.1
GCA_016415005.1
GCA_016415025.1
GCA_016415045.1
GCA_016415085.1
GCA_016415105.1
GCA_016415125.1
GCA_016415145.1
GCA_016415165.1
GCA_016415185.1
GCA_016415225.1
GCA_016415245.1
GCA_016415285.1
GCA_016415305.1

GCA_016415325.1
GCA_016415345.1
GCA_016415365.1
GCA_016415385.1
GCA_016415405.1
GCA_016415425.1
GCA_016415445.1
GCA_016415465.1
GCA_016415485.1
GCA_016415505.1
GCA_016415545.1
GCA_016415565.1
GCA_016415585.1
GCA_016415605.1
GCA_016415705.1
GCA_016415725.1
GCA_016432785.1
GCA_016432805.1
GCA_016446285.1
GCA_016446305.1
GCA_016446335.1
GCA_016446355.1
GCA_016446395.1
GCA_016446415.2
GCA_016451985.1
GCA_016452005.1
GCA_016452025.1
GCA_016452045.1
GCA_016452065.1
GCA_016452105.1
GCA_016452165.1
GCA_016454165.1
GCA_016454185.1
GCA_016454205.1
GCA_016454225.1
GCA_016454245.1
GCA_016454265.1
GCA_016454285.1
GCA_016454305.1
GCA_016454325.1
GCA_016454345.1
GCA_016454365.1
GCA_016454385.1
GCA_016454425.1
GCA_016454445.1
GCA_016454465.1
GCA_016454505.1
GCA_016454525.1
GCA_016454545.1
GCA_016454585.1
GCA_016454605.1
GCA_016454625.1
GCA_016454645.1
GCA_016454665.1

GCA_016454705.1
GCA_016454725.1
GCA_016454765.1
GCA_016454805.1
GCA_016454965.1
GCA_016454985.1
GCA_016455005.1
GCA_016455025.1
GCA_016455065.1
GCA_016455085.1
GCA_016455105.1
GCA_016455145.1
GCA_016455165.1
GCA_016455205.1
GCA_016455225.1
GCA_016455245.1
GCA_016455265.1
GCA_016455285.1
GCA_016455325.1
GCA_016458785.1
GCA_016458805.1
GCA_016458825.1
GCA_016458845.1
GCA_016458865.1
GCA_016458885.1
GCA_016458905.1
GCA_016458925.1
GCA_016458945.1
GCA_016466415.2
GCA_016467295.1
GCA_016469335.1
GCA_016495405.1
GCA_016495605.1
GCA_016495705.1
GCA_016495725.1
GCA_016495865.1
GCA_016496025.1
GCA_016510335.1
GCA_016549335.1
GCA_016549355.1
GCA_016549375.1
GCA_016549395.1
GCA_016576845.1
GCA_016576965.1
GCA_016584325.1
GCA_016584355.1
GCA_016584375.1
GCA_016584405.1
GCA_016584425.1
GCA_016584445.1
GCA_016584725.1
GCA_016591715.1
GCA_016591975.1
GCA_016591995.1

GCA_016592015.1
GCA_016592155.1
GCA_016592295.1
GCA_016592435.1
GCA_016592555.1
GCA_016592575.1
GCA_016592595.1
GCA_016592615.1
GCA_016592635.1
GCA_016592875.1
GCA_016593055.1
GCA_016593175.1
GCA_016593195.1
GCA_016593215.1
GCA_016593235.1
GCA_016593255.1
GCA_016598555.1
GCA_016598575.1
GCA_016598595.1
GCA_016598615.1
GCA_016598635.1
GCA_016598655.1
GCA_016598675.1
GCA_016598695.1
GCA_016598715.1
GCA_016598735.1
GCA_016598755.1
GCA_016598775.1
GCA_016598815.1
GCA_016598875.1
GCA_016598895.1
GCA_016598915.1
GCA_016599575.1
GCA_016599595.1
GCA_016599615.1
GCA_016599635.1
GCA_016599655.1
GCA_016599675.2
GCA_016599695.1
GCA_016599715.1
GCA_016599735.1
GCA_016599755.1
GCA_016599775.1
GCA_016599795.1
GCA_016599815.1
GCA_016599835.1
GCA_016599855.1
GCA_016600905.1
GCA_016600925.1
GCA_016605905.1
GCA_016605985.1
GCA_016607565.1
GCA_016613475.2
GCA_016613535.2

GCA_016615645.1
GCA_016615815.1
GCA_016615955.1
GCA_016616095.2
GCA_016616235.1
GCA_016616425.1
GCA_016616645.1
GCA_016616885.1
GCA_016617695.1
GCA_016617715.1
GCA_016618215.1
GCA_016618235.1
GCA_016626125.1
GCA_016628525.2
GCA_016628735.2
GCA_016629445.1
GCA_016629465.1
GCA_016629485.1
GCA_016629505.1
GCA_016629525.1
GCA_016629545.1
GCA_016629565.1
GCA_016629585.1
GCA_016638525.1
GCA_016638585.1
GCA_016638705.1
GCA_016647575.1
GCA_016647595.1
GCA_016649195.2
GCA_016653495.1
GCA_016653515.1
GCA_016653555.1
GCA_016653595.1
GCA_016653615.1
GCA_016653635.2
GCA_016653655.1
GCA_016653675.1
GCA_016653695.1
GCA_016656965.1
GCA_016657085.1
GCA_016658865.1
GCA_016659085.1
GCA_016659185.1
GCA_016694755.2
GCA_016694775.1
GCA_016694795.1
GCA_016694815.1
GCA_016694895.1
GCA_016694995.1
GCA_016695175.1
GCA_016695385.1
GCA_016696665.1
GCA_016698685.1
GCA_016698705.1

GCA_016699045.1
GCA_016699065.1
GCA_016699085.1
GCA_016699105.1
GCA_016699125.1
GCA_016699145.1
GCA_016699165.1
GCA_016699185.1
GCA_016699205.1
GCA_016699225.1
GCA_016699245.1
GCA_016699265.1
GCA_016699285.1
GCA_016699305.1
GCA_016699365.1
GCA_016699385.1
GCA_016699405.1
GCA_016699425.1
GCA_016699445.1
GCA_016699465.1
GCA_016699515.1
GCA_016699575.1
GCA_016699595.1
GCA_016699655.1
GCA_016699675.1
GCA_016699715.1
GCA_016699735.1
GCA_016699755.1
GCA_016699775.1
GCA_016699795.1
GCA_016699815.1
GCA_016699835.1
GCA_016699855.1
GCA_016699875.1
GCA_016699895.1
GCA_016699915.1
GCA_016699935.1
GCA_016699955.1
GCA_016699975.1
GCA_016699995.1
GCA_016700015.1
GCA_016700035.1
GCA_016700055.1
GCA_016700075.1
GCA_016700095.1
GCA_016700115.1
GCA_016700135.1
GCA_016700155.1
GCA_016700175.1
GCA_016700195.1
GCA_016700315.1
GCA_016700335.1
GCA_016700355.1
GCA_016700375.1

GCA_016700395.1
GCA_016700415.1
GCA_016722835.2
GCA_016724565.1
GCA_016724665.1
GCA_016724785.1
GCA_016724805.1
GCA_016724825.1
GCA_016724845.1
GCA_016724865.1
GCA_016724885.1
GCA_016725005.1
GCA_016725165.1
GCA_016725185.1
GCA_016725205.1
GCA_016725225.1
GCA_016725245.1
GCA_016725265.1
GCA_016725285.1
GCA_016725305.1
GCA_016725325.1
GCA_016725465.1
GCA_016725645.1
GCA_016725825.1
GCA_016725865.1
GCA_016725905.1
GCA_016725925.1
GCA_016725945.1
GCA_016726045.1
GCA_016726205.1
GCA_016726245.1
GCA_016726265.1
GCA_016726285.1
GCA_016726305.1
GCA_016726325.1
GCA_016726345.1
GCA_016726365.1
GCA_016726485.1
GCA_016726625.1
GCA_016726765.1
GCA_016726885.1
GCA_016726945.1
GCA_016726965.1
GCA_016726985.1
GCA_016727005.1
GCA_016727125.1
GCA_016727265.1
GCA_016727305.1
GCA_016727325.1
GCA_016727345.1
GCA_016727365.1
GCA_016727385.1
GCA_016727405.1
GCA_016727425.1

GCA_016727445.1
GCA_016727585.1
GCA_016727765.1
GCA_016727905.1
GCA_016728005.1
GCA_016728025.1
GCA_016728045.1
GCA_016728065.1
GCA_016728085.1
GCA_016728105.1
GCA_016728205.1
GCA_016728365.1
GCA_016728505.1
GCA_016728625.1
GCA_016728645.1
GCA_016728665.1
GCA_016728685.1
GCA_016728705.1
GCA_016728725.1
GCA_016728745.1
GCA_016728765.1
GCA_016728785.1
GCA_016728805.1
GCA_016728825.1
GCA_016728865.1
GCA_016743035.1
GCA_016743055.1
GCA_016743075.1
GCA_016743095.1
GCA_016743115.1
GCA_016743155.1
GCA_016743795.2
GCA_016743815.1
GCA_016743835.1
GCA_016743855.1
GCA_016743875.1
GCA_016743895.1
GCA_016745095.1
GCA_016745115.1
GCA_016745135.1
GCA_016745155.1
GCA_016745175.1
GCA_016745195.1
GCA_016745215.1
GCA_016745295.1
GCA_016745355.1
GCA_016747875.1
GCA_016747995.1
GCA_016748085.1
GCA_016748175.1
GCA_016748265.1
GCA_016748415.1
GCA_016748555.1
GCA_016748675.1

GCA_016748795.1
GCA_016748925.1
GCA_016749025.1
GCA_016749085.1
GCA_016749215.1
GCA_016749335.1
GCA_016749455.1
GCA_016749575.1
GCA_016749715.1
GCA_016749815.1
GCA_016749935.1
GCA_016755615.1
GCA_016755635.1
GCA_016755655.1
GCA_016755675.1
GCA_016755695.1
GCA_016755715.1
GCA_016755735.1
GCA_016755755.1
GCA_016755775.1
GCA_016755795.1
GCA_016755815.1
GCA_016755915.1
GCA_016755935.1
GCA_016755955.1
GCA_016755975.1
GCA_016755995.1
GCA_016756015.1
GCA_016756035.1
GCA_016756055.1
GCA_016756075.1
GCA_016756095.1
GCA_016756115.1
GCA_016756135.1
GCA_016756155.1
GCA_016756175.1
GCA_016756195.1
GCA_016756215.1
GCA_016756235.1
GCA_016756255.1
GCA_016756275.1
GCA_016756295.1
GCA_016756315.1
GCA_016756335.1
GCA_016756355.1
GCA_016756375.1
GCA_016756395.1
GCA_016756455.1
GCA_016756475.1
GCA_016756495.1
GCA_016756515.1
GCA_016756535.1
GCA_016756555.1
GCA_016756575.1

GCA_016756595.1
GCA_016756615.1
GCA_016756635.1
GCA_016756655.1
GCA_016757535.1
GCA_016757555.1
GCA_016757575.1
GCA_016757595.1
GCA_016757635.1
GCA_016757655.1
GCA_016757675.1
GCA_016757695.1
GCA_016757715.1
GCA_016757735.1
GCA_016757755.1
GCA_016757775.1
GCA_016757795.1
GCA_016758865.1
GCA_016765655.2
GCA_016766595.1
GCA_016766615.1
GCA_016766635.1
GCA_016766655.1
GCA_016766675.1
GCA_016766695.1
GCA_016766715.1
GCA_016766735.1
GCA_016766755.1
GCA_016766955.1
GCA_016766975.1
GCA_016766995.1
GCA_016767015.1
GCA_016767035.1
GCA_016767055.1
GCA_016767075.1
GCA_016767095.1
GCA_016767115.1
GCA_016767135.1
GCA_016767155.1
GCA_016767175.1
GCA_016767215.1
GCA_016767715.1
GCA_016767735.1
GCA_016767755.1
GCA_016767775.1
GCA_016767795.1
GCA_016767835.1
GCA_016767855.1
GCA_016772275.1
GCA_016772315.1
GCA_016772335.1
GCA_016772355.1
GCA_016772375.1
GCA_016772395.1

GCA_016772475.1
GCA_016772495.1
GCA_016772515.1
GCA_016772535.1
GCA_016772555.1
GCA_016772575.1
GCA_016772595.1
GCA_016774415.1
GCA_016774435.1
GCA_016774455.1
GCA_016774475.1
GCA_016774495.1
GCA_016774515.1
GCA_016774535.1
GCA_016774555.1
GCA_016774575.1
GCA_016775685.1
GCA_016775725.1
GCA_016775745.1
GCA_016775765.1
GCA_016775785.1
GCA_016775805.1
GCA_016775825.1
GCA_016775845.1
GCA_016775865.1
GCA_016775885.1
GCA_016775905.1
GCA_016775925.1
GCA_016775945.1
GCA_016775965.1
GCA_016775985.1
GCA_016776005.1
GCA_016776025.1
GCA_016776045.1
GCA_016776065.1
GCA_016776085.1
GCA_016776105.1
GCA_016776125.1
GCA_016776145.1
GCA_016776165.1
GCA_016776185.1
GCA_016776205.1
GCA_016776225.1
GCA_016776245.1
GCA_016776265.1
GCA_016776285.1
GCA_016776305.1
GCA_016776325.1
GCA_016776345.1
GCA_016776365.1
GCA_016776385.1
GCA_016779865.1
GCA_016780025.1
GCA_016780145.1

GCA_016780285.1
GCA_016780415.1
GCA_016780575.1
GCA_016780725.1
GCA_016798345.1
GCA_016800965.1
GCA_016801025.1
GCA_016801375.1
GCA_016801615.1
GCA_016801635.1
GCA_016801655.1
GCA_016801675.1
GCA_016801695.1
GCA_016801715.1
GCA_016801735.1
GCA_016801755.1
GCA_016801775.1
GCA_016802315.1
GCA_016802335.1
GCA_016802355.1
GCA_016802385.1
GCA_016802405.1
GCA_016802425.1
GCA_016802445.1
GCA_016802645.1
GCA_016803475.1
GCA_016803835.1
GCA_016803855.1
GCA_016803875.1
GCA_016803915.1
GCA_016803935.1
GCA_016803985.1
GCA_016804005.1
GCA_016804025.1
GCA_016804045.1
GCA_016804125.1
GCA_016804245.1
GCA_016804265.1
GCA_016804285.1
GCA_016804325.1
GCA_016804345.1
GCA_016804365.1
GCA_016805985.1
GCA_016806005.1
GCA_016806025.1
GCA_016806045.1
GCA_016806065.1
GCA_016806125.1
GCA_016806145.1
GCA_016806835.2
GCA_016807685.1
GCA_016807705.1
GCA_016807725.1
GCA_016807745.1

GCA_016807785.1
GCA_016807805.1
GCA_016811855.1
GCA_016811875.1
GCA_016811915.1
GCA_016811975.1
GCA_016811995.1
GCA_016812015.1
GCA_016812035.1
GCA_016812055.1
GCA_016812075.1
GCA_016812095.1
GCA_016834435.1
GCA_016834455.1
GCA_016834475.1
GCA_016834495.1
GCA_016834535.1
GCA_016835115.1
GCA_016835135.1
GCA_016838625.1
GCA_016838645.1
GCA_016838665.1
GCA_016838685.1
GCA_016838705.1
GCA_016839145.1
GCA_016839185.1
GCA_016839205.1
GCA_016839225.1
GCA_016845695.1
GCA_016845885.1
GCA_016845925.1
GCA_016858125.1
GCA_016858245.2
GCA_016858305.2
GCA_016859125.1
GCA_016859145.1
GCA_016859165.1
GCA_016859185.1
GCA_016859395.1
GCA_016861285.1
GCA_016861355.2
GCA_016861425.1
GCA_016861485.1
GCA_016861545.1
GCA_016861955.1
GCA_016864015.1
GCA_016864035.1
GCA_016864075.1
GCA_016864095.1
GCA_016864115.1
GCA_016864135.1
GCA_016864155.1
GCA_016864175.1
GCA_016864195.1

GCA_016864215.1
GCA_016864235.1
GCA_016864255.1
GCA_016864295.1
GCA_016864315.2
GCA_016864335.2
GCA_016864355.1
GCA_016864375.1
GCA_016864395.1
GCA_016864415.1
GCA_016864475.1
GCA_016864495.1
GCA_016864515.1
GCA_016864535.1
GCA_016864555.1
GCA_016864575.1
GCA_016864595.1
GCA_016864615.1
GCA_016864875.1
GCA_016864895.1
GCA_016864915.1
GCA_016864935.1
GCA_016864955.1
GCA_016864975.1
GCA_016864995.1
GCA_016865015.1
GCA_016865035.1
GCA_016865215.1
GCA_016865235.1
GCA_016865255.1
GCA_016865275.1
GCA_016865295.1
GCA_016865345.1
GCA_016865365.1
GCA_016865385.1
GCA_016865405.1
GCA_016865425.1
GCA_016865445.1
GCA_016865465.1
GCA_016865485.2
GCA_016865525.2
GCA_016887485.1
GCA_016887505.1
GCA_016887545.1
GCA_016887565.1
GCA_016887735.2
GCA_016888765.1
GCA_016888785.1
GCA_016888845.1
GCA_016888865.1
GCA_016888885.1
GCA_016888905.1
GCA_016888925.1
GCA_016888945.1

GCA_016888965.1
GCA_016888985.1
GCA_016889005.1
GCA_016889025.1
GCA_016889045.1
GCA_016889065.1
GCA_016889085.1
GCA_016889105.1
GCA_016889125.1
GCA_016889145.1
GCA_016889165.1
GCA_016889185.1
GCA_016889205.1
GCA_016889225.1
GCA_016889245.1
GCA_016889265.1
GCA_016889285.1
GCA_016889305.1
GCA_016889325.1
GCA_016889345.1
GCA_016889365.1
GCA_016889385.1
GCA_016889405.1
GCA_016889425.1
GCA_016889445.1
GCA_016889465.1
GCA_016889485.1
GCA_016889505.1
GCA_016889525.1
GCA_016889545.1
GCA_016889565.1
GCA_016889585.1
GCA_016889605.1
GCA_016889625.1
GCA_016889645.1
GCA_016889665.1
GCA_016889685.1
GCA_016889705.1
GCA_016889725.1
GCA_016889745.1
GCA_016889765.1
GCA_016889785.1
GCA_016889805.1
GCA_016889825.1
GCA_016889845.1
GCA_016889865.1
GCA_016889885.1
GCA_016889905.1
GCA_016889925.1
GCA_016889945.1
GCA_016889965.1
GCA_016889985.1
GCA_016890005.1
GCA_016890025.1

GCA_016890045.1
GCA_016890065.1
GCA_016890085.1
GCA_016890105.1
GCA_016894165.1
GCA_016894285.1
GCA_016894325.1
GCA_016894345.1
GCA_016894365.1
GCA_016894385.1
GCA_016894405.1
GCA_016894445.1
GCA_016899425.1
GCA_016899445.1
GCA_016903035.1
GCA_016903055.1
GCA_016903075.1
GCA_016903095.1
GCA_016903115.1
GCA_016903135.1
GCA_016903155.1
GCA_016903175.1
GCA_016903195.1
GCA_016903215.1
GCA_016903235.1
GCA_016903255.1
GCA_016903275.1
GCA_016903295.1
GCA_016903315.1
GCA_016903335.1
GCA_016903355.1
GCA_016903375.1
GCA_016903395.1
GCA_016903415.1
GCA_016903435.1
GCA_016903455.1
GCA_016903475.1
GCA_016903495.1
GCA_016903515.1
GCA_016903535.1
GCA_016903555.1
GCA_016903575.1
GCA_016903595.1
GCA_016903615.1
GCA_016903715.1
GCA_016903735.1
GCA_016903755.1
GCA_016903775.1
GCA_016903795.1
GCA_016903815.1
GCA_016903835.1
GCA_016903855.1
GCA_016903875.1
GCA_016903895.1

GCA_016903915.1
GCA_016903935.1
GCA_016903955.1
GCA_016903975.1
GCA_016903995.1
GCA_016904015.1
GCA_016904035.1
GCA_016904055.1
GCA_016904075.1
GCA_016904095.1
GCA_016904115.1
GCA_016904135.1
GCA_016904155.1
GCA_016904175.1
GCA_016904195.1
GCA_016904215.1
GCA_016904235.1
GCA_016904255.1
GCA_016904275.1
GCA_016904295.1
GCA_016904315.1
GCA_016904335.1
GCA_016904355.1
GCA_016904375.1
GCA_016904395.1
GCA_016904415.1
GCA_016904435.1
GCA_016904455.1
GCA_016904475.1
GCA_016904495.1
GCA_016904515.1
GCA_016904535.1
GCA_016904555.1
GCA_016904575.1
GCA_016904595.1
GCA_016904615.1
GCA_016904635.1
GCA_016904655.1
GCA_016904675.1
GCA_016904695.1
GCA_016904715.2
GCA_016904735.2
GCA_016904755.2
GCA_016904775.2
GCA_016904795.2
GCA_016904815.2
GCA_016904925.1
GCA_016906065.1
GCA_016906185.1
GCA_016906205.1
GCA_016906225.1
GCA_016906245.1
GCA_016906265.1
GCA_016917315.1

GCA_016917335.1
GCA_016917355.1
GCA_016917755.1
GCA_016917775.1
GCA_016917795.1
GCA_016918765.2
GCA_016919265.1
GCA_016919285.1
GCA_016919305.1
GCA_016919325.1
GCA_016919365.1
GCA_016919385.1
GCA_016919405.1
GCA_016919445.1
GCA_016919485.1
GCA_016919505.2
GCA_016919525.2
GCA_016919545.1
GCA_016919665.1
GCA_016919685.1
GCA_016919705.1
GCA_016920955.1
GCA_016920995.2
GCA_016921015.1
GCA_016921035.1
GCA_016921055.1
GCA_016924835.1
GCA_016924995.1
GCA_016925215.1
GCA_016925235.1
GCA_016925255.1
GCA_016925275.1
GCA_016925295.1
GCA_016925315.1
GCA_016925335.1
GCA_016925355.1
GCA_016925375.1
GCA_016925395.1
GCA_016925415.1
GCA_016925435.1
GCA_016925455.1
GCA_016925475.1
GCA_016925495.1
GCA_016925515.1
GCA_016925535.1
GCA_016925555.1
GCA_016925575.1
GCA_016925595.1
GCA_016925615.1
GCA_016925635.1
GCA_016925655.1
GCA_016925675.1
GCA_016939435.1
GCA_016939455.1

GCA_016939475.1
GCA_016939495.1
GCA_016939515.1
GCA_016939535.1
GCA_016939555.1
GCA_016939575.1
GCA_016939615.1
GCA_016939635.1
GCA_016939675.1
GCA_016939695.1
GCA_016939715.1
GCA_016939735.1
GCA_016939755.1
GCA_016939775.1
GCA_016939795.1
GCA_016939815.1
GCA_016939835.1
GCA_016942475.1
GCA_016942505.1
GCA_016942535.1
GCA_016942575.1
GCA_016942595.1
GCA_016942615.1
GCA_016942635.1
GCA_016942655.1
GCA_016944235.1
GCA_016944255.1
GCA_016944275.1
GCA_016944295.1
GCA_016944315.1
GCA_016944435.1
GCA_016944555.1
GCA_016944595.1
GCA_016944615.1
GCA_016944635.1
GCA_016944655.1
GCA_016944675.1
GCA_016944695.1
GCA_016944715.1
GCA_016944735.1
GCA_016944755.1
GCA_016944775.1
GCA_016952595.1
GCA_016952955.2
GCA_016952995.1
GCA_017068035.1
GCA_017068175.1
GCA_017068195.1
GCA_017068215.1
GCA_017068235.1
GCA_017068275.1
GCA_017068315.1
GCA_017068355.1
GCA_017068375.1

GCA_017068395.1
GCA_017068455.1
GCA_017084465.1
GCA_017084505.1
GCA_017086365.1
GCA_017086385.1
GCA_017086405.1
GCA_017086425.1
GCA_017086445.1
GCA_017086465.1
GCA_017086485.1
GCA_017086505.1
GCA_017086525.1
GCA_017086545.1
GCA_017086565.1
GCA_017086585.1
GCA_017086605.1
GCA_017088185.1
GCA_017088305.1
GCA_017088385.1
GCA_017088405.1
GCA_017088425.1
GCA_017088445.1
GCA_017088465.1
GCA_017088485.1
GCA_017094545.1
GCA_017094565.1
GCA_017094585.1
GCA_017094605.1
GCA_017096325.2
GCA_017096345.2
GCA_017096365.2
GCA_017096385.2
GCA_017096405.2
GCA_017096425.2
GCA_017098185.2
GCA_017098205.2
GCA_017098225.2
GCA_017098245.2
GCA_017098265.1
GCA_017100085.1
GCA_017101655.1
GCA_017132735.1
GCA_017132755.1
GCA_017132775.1
GCA_017132795.1
GCA_017134335.1
GCA_017134355.1
GCA_017134375.1
GCA_017134395.1
GCA_017134815.1
GCA_017161035.1
GCA_017161055.1
GCA_017161075.1

GCA_017161095.1
GCA_017161115.1
GCA_017161135.1
GCA_017161155.1
GCA_017161205.1
GCA_017161225.1
GCA_017161245.1
GCA_017161265.1
GCA_017161285.1
GCA_017161325.1
GCA_017161345.1
GCA_017161365.1
GCA_017161385.1
GCA_017161405.1
GCA_017161425.1
GCA_017161445.1
GCA_017161485.1
GCA_017161505.1
GCA_017161525.1
GCA_017161545.1
GCA_017161565.1
GCA_017161585.1
GCA_017161605.1
GCA_017161725.1
GCA_017161745.1
GCA_017161765.1
GCA_017161785.1
GCA_017161805.1
GCA_017161825.1
GCA_017161845.1
GCA_017161865.1
GCA_017164035.1
GCA_017164055.1
GCA_017164755.1
GCA_017164775.1
GCA_017164795.1
GCA_017164815.1
GCA_017164835.1
GCA_017164855.1
GCA_017164875.1
GCA_017164895.1
GCA_017164915.1
GCA_017164935.1
GCA_017164955.1
GCA_017164975.1
GCA_017164995.1
GCA_017165015.1
GCA_017165035.1
GCA_017165055.1
GCA_017165075.1
GCA_017165095.1
GCA_017165115.1
GCA_017165135.1
GCA_017165155.1

GCA_017165175.1
GCA_017165195.1
GCA_017165215.1
GCA_017165235.1
GCA_017165255.1
GCA_017165275.1
GCA_017165295.1
GCA_017165315.1
GCA_017165335.1
GCA_017165355.1
GCA_017165375.1
GCA_017165395.1
GCA_017165415.1
GCA_017165435.1
GCA_017165455.1
GCA_017165475.1
GCA_017165495.1
GCA_017165515.1
GCA_017165535.1
GCA_017165555.1
GCA_017167965.1
GCA_017167985.1
GCA_017183555.1
GCA_017183655.1
GCA_017183735.1
GCA_017183775.1
GCA_017183795.1
GCA_017183815.1
GCA_017183835.1
GCA_017183855.1
GCA_017183875.1
GCA_017183895.1
GCA_017183915.1
GCA_017183935.1
GCA_017183955.1
GCA_017183975.1
GCA_017189355.1
GCA_017189375.1
GCA_017189395.1
GCA_017189415.1
GCA_017189435.1
GCA_017190535.1
GCA_017190595.1
GCA_017190695.1
GCA_017190795.1
GCA_017190875.1
GCA_017220215.1
GCA_017277335.1
GCA_017277455.1
GCA_017280035.1
GCA_017280155.1
GCA_017280235.1
GCA_017298635.1
GCA_017298675.1

GCA_017298695.1
GCA_017298715.1
GCA_017298735.1
GCA_017298755.1
GCA_017298775.1
GCA_017298795.1
GCA_017298815.1
GCA_017298835.1
GCA_017298855.1
GCA_017298875.1
GCA_017298895.1
GCA_017298915.1
GCA_017298935.1
GCA_017298955.1
GCA_017298975.1
GCA_017298995.1
GCA_017299015.1
GCA_017299035.1
GCA_017299175.1
GCA_017299275.1
GCA_017299315.1
GCA_017299435.1
GCA_017299535.1
GCA_017299575.1
GCA_017299595.1
GCA_017301255.1
GCA_017301275.1
GCA_017301295.1
GCA_017301315.1
GCA_017301335.1
GCA_017301355.1
GCA_017301375.1
GCA_017301615.1
GCA_017301655.1
GCA_017301675.1
GCA_017301695.1
GCA_017301715.1
GCA_017301775.1
GCA_017301795.1
GCA_017301815.1
GCA_017301835.1
GCA_017301855.1
GCA_017301935.1
GCA_017309405.1
GCA_017309425.1
GCA_017309445.1
GCA_017309485.1
GCA_017309505.1
GCA_017309525.1
GCA_017309545.1
GCA_017309585.1
GCA_017309605.1
GCA_017309625.1
GCA_017309645.1

GCA_017309675.2
GCA_017309955.1
GCA_017309975.1
GCA_017309995.1
GCA_017310015.1
GCA_017310035.1
GCA_017310055.1
GCA_017310195.1
GCA_017310215.1
GCA_017310235.1
GCA_017310275.1
GCA_017310305.1
GCA_017310325.1
GCA_017310345.1
GCA_017310365.1
GCA_017310385.1
GCA_017310405.1
GCA_017310425.1
GCA_017310445.1
GCA_017310465.1
GCA_017310485.1
GCA_017310505.1
GCA_017310525.1
GCA_017310545.1
GCA_017310565.1
GCA_017311125.1
GCA_017311165.1
GCA_017315205.1
GCA_017329545.1
GCA_017338715.1
GCA_017338795.1
GCA_017338855.1
GCA_017346795.1
GCA_017346815.1
GCA_017346835.1
GCA_017346855.1
GCA_017346875.1
GCA_017347365.1
GCA_017347385.1
GCA_017347425.1
GCA_017347445.1
GCA_017347465.1
GCA_017347485.1
GCA_017347565.1
GCA_017347585.1
GCA_017347605.1
GCA_017347625.1
GCA_017348835.2
GCA_017348855.1
GCA_017348875.1
GCA_017348895.1
GCA_017348915.1
GCA_017348935.1
GCA_017348955.1

GCA_017348975.1
GCA_017351955.1
GCA_017351975.1
GCA_017351995.1
GCA_017352015.1
GCA_017352035.1
GCA_017352055.1
GCA_017352075.1
GCA_017352115.1
GCA_017352135.1
GCA_017352195.1
GCA_017352215.1
GCA_017352235.1
GCA_017352255.1
GCA_017352275.1
GCA_017352295.1
GCA_017352315.1
GCA_017352335.1
GCA_017354925.1
GCA_017354945.1
GCA_017354965.1
GCA_017354985.1
GCA_017355005.1
GCA_017355025.1
GCA_017355045.1
GCA_017355405.1
GCA_017355425.1
GCA_017355525.1
GCA_017355605.1
GCA_017356665.1
GCA_017356685.1
GCA_017356705.2
GCA_017356725.2
GCA_017356745.1
GCA_017356765.1
GCA_017356785.1
GCA_017356805.1
GCA_017356825.1
GCA_017356845.1
GCA_017356865.1
GCA_017356885.1
GCA_017356905.1
GCA_017356925.1
GCA_017356945.1
GCA_017356965.1
GCA_017357005.1
GCA_017357025.1
GCA_017357065.1
GCA_017357085.1
GCA_017357125.1
GCA_017357145.1
GCA_017357165.1
GCA_017357185.1
GCA_017357205.1

GCA_017357225.1
GCA_017357245.1
GCA_017357265.1
GCA_017357285.1
GCA_017357305.1
GCA_017357325.1
GCA_017357345.1
GCA_017357385.1
GCA_017357425.1
GCA_017357445.1
GCA_017357465.1
GCA_017357505.1
GCA_017357545.1
GCA_017357565.1
GCA_017357585.1
GCA_017357605.1
GCA_017357625.1
GCA_017357645.1
GCA_017357665.1
GCA_017357685.1
GCA_017357705.1
GCA_017357725.1
GCA_017357745.1
GCA_017357765.1
GCA_017357785.1
GCA_017357805.1
GCA_017357825.1
GCA_017357865.1
GCA_017357905.1
GCA_017357925.1
GCA_017358145.1
GCA_017358165.1
GCA_017359825.1
GCA_017359885.1
GCA_017360005.1
GCA_017360085.1
GCA_017360165.1
GCA_017360245.1
GCA_017360305.1
GCA_017363615.2
GCA_017363655.2
GCA_017372155.1
GCA_017372215.1
GCA_017372255.1
GCA_017372375.1
GCA_017376415.1
GCA_017377355.1
GCA_017377395.1
GCA_017377875.1
GCA_017378155.1
GCA_017378195.1
GCA_017378235.1
GCA_017378275.1
GCA_017378315.3

GCA_017378355.1
GCA_017378395.1
GCA_017378435.1
GCA_017378475.1
GCA_017378625.1
GCA_017378695.1
GCA_017378735.1
GCA_017474015.1
GCA_017474165.1
GCA_017487345.1
GCA_017487405.1
GCA_017488845.2
GCA_017493175.3
GCA_017498465.1
GCA_017498485.1
GCA_017498505.1
GCA_017498525.1
GCA_017498545.1
GCA_017498565.1
GCA_017498585.1
GCA_017498605.1
GCA_017498625.1
GCA_017498665.1
GCA_017498685.1
GCA_017504145.1
GCA_017514945.1
GCA_017526105.1
GCA_017565645.3
GCA_017565685.3
GCA_017569185.1
GCA_017569205.1
GCA_017569225.1
GCA_017569245.1
GCA_017569285.1
GCA_017569325.1
GCA_017569345.1
GCA_017569365.1
GCA_017569385.1
GCA_017569405.1
GCA_017569925.1
GCA_017570045.1
GCA_017570125.1
GCA_017570225.1
GCA_017570325.1
GCA_017570425.1
GCA_017570485.1
GCA_017570505.1
GCA_017570525.1
GCA_017570585.1
GCA_017570605.1
GCA_017570625.1
GCA_017570645.1
GCA_017570685.1
GCA_017576965.1

GCA_017577085.1
GCA_017577205.1
GCA_017577225.1
GCA_017579825.1
GCA_017579845.1
GCA_017579865.1
GCA_017584065.1
GCA_017584165.1
GCA_017584205.1
GCA_017584225.1
GCA_017584245.1
GCA_017592625.2
GCA_017603565.1
GCA_017603605.1
GCA_017603725.1
GCA_017603825.1
GCA_017603845.1
GCA_017603965.1
GCA_017604245.1
GCA_017604265.1
GCA_017604325.1
GCA_017607425.1
GCA_017638445.1
GCA_017638465.1
GCA_017638545.1
GCA_017638885.1
GCA_017638905.1
GCA_017638925.1
GCA_017638945.1
GCA_017638965.1
GCA_017638985.1
GCA_017639005.1
GCA_017639025.1
GCA_017639045.1
GCA_017639065.1
GCA_017639085.1
GCA_017639105.1
GCA_017639125.1
GCA_017639145.1
GCA_017639165.1
GCA_017639185.1
GCA_017639205.1
GCA_017639585.1
GCA_017639855.1
GCA_017639875.1
GCA_017639895.1
GCA_017654165.1
GCA_017654245.1
GCA_017654315.1
GCA_017654485.1
GCA_017654545.1
GCA_017654605.1
GCA_017655665.1
GCA_017655685.1

GCA_017655725.1
GCA_017655745.1
GCA_017655785.1
GCA_017655805.1
GCA_017655825.1
GCA_017655845.1
GCA_017655865.1
GCA_017655885.1
GCA_017655905.1
GCA_017655925.1
GCA_017655945.1
GCA_017655965.1
GCA_017656055.1
GCA_017656075.1
GCA_017671885.1
GCA_017723855.1
GCA_017723895.1
GCA_017723975.1
GCA_017724035.1
GCA_017724115.1
GCA_017724155.1
GCA_017724195.1
GCA_017724215.1
GCA_017726495.1
GCA_017726555.1
GCA_017726575.1
GCA_017726655.1
GCA_017742795.1
GCA_017742815.1
GCA_017742855.1
GCA_017742875.1
GCA_017742895.1
GCA_017742915.1
GCA_017742935.1
GCA_017742955.1
GCA_017742975.1
GCA_017742995.1
GCA_017743015.1
GCA_017743035.1
GCA_017743055.1
GCA_017743075.1
GCA_017743095.1
GCA_017743115.1
GCA_017743135.1
GCA_017743155.1
GCA_017743175.1
GCA_017743195.1
GCA_017743215.1
GCA_017743235.1
GCA_017748085.1
GCA_017750945.1
GCA_017751145.1
GCA_017751165.1
GCA_017751185.1

GCA_017751205.1
GCA_017751225.1
GCA_017751245.1
GCA_017751265.1
GCA_017753625.1
GCA_017753665.1
GCA_017753785.1
GCA_017792025.1
GCA_017792185.1
GCA_017792285.1
GCA_017795485.1
GCA_017795605.1
GCA_017795725.1
GCA_017795885.1
GCA_017797845.1
GCA_017797885.1
GCA_017798005.1
GCA_017798125.1
GCA_017798145.1
GCA_017798165.1
GCA_017798205.1
GCA_017798225.1
GCA_017798245.1
GCA_017798265.1
GCA_017798285.1
GCA_017798305.1
GCA_017808555.1
GCA_017809215.1
GCA_017809835.1
GCA_017815515.1
GCA_017815535.1
GCA_017815555.1
GCA_017815575.1
GCA_017815595.1
GCA_017815615.1
GCA_017815635.1
GCA_017815655.1
GCA_017815675.1
GCA_017815695.1
GCA_017815715.1
GCA_017815735.1
GCA_017815775.1
GCA_017815795.1
GCA_017815815.1
GCA_017815835.1
GCA_017821195.1
GCA_017821235.1
GCA_017821275.1
GCA_017821355.1
GCA_017821475.1
GCA_017821535.1
GCA_017821595.1
GCA_017821615.1
GCA_017821635.1

GCA_017821655.1
GCA_017840535.1
GCA_017840575.1
GCA_017858835.1
GCA_017863935.1
GCA_017868515.1
GCA_017868775.1
GCA_017868935.1
GCA_017869285.1
GCA_017869415.1
GCA_017873595.1
GCA_017893505.1
GCA_017893605.1
GCA_017893625.1
GCA_017893945.1
GCA_017893965.1
GCA_017893985.1
GCA_017894285.1
GCA_017894305.1
GCA_017894325.1
GCA_017894345.1
GCA_017894365.1
GCA_017894385.1
GCA_017896245.1
GCA_017896265.1
GCA_017896285.1
GCA_017896305.1
GCA_017896325.1
GCA_017896345.1
GCA_017896365.1
GCA_017896385.1
GCA_017896405.1
GCA_017897965.1
GCA_017898005.1
GCA_017898025.1
GCA_017900915.1
GCA_017900955.1
GCA_017900975.1
GCA_017900995.1
GCA_017901015.1
GCA_017901035.1
GCA_017901055.1
GCA_017901075.1
GCA_017901095.1
GCA_017901115.1
GCA_017901135.1
GCA_017901155.1
GCA_017901175.1
GCA_017901195.1
GCA_017903455.1
GCA_017909015.1
GCA_017909135.1
GCA_017909235.1
GCA_017909295.1

GCA_017909395.1
GCA_017909435.1
GCA_017909455.1
GCA_017910955.1
GCA_017915135.1
GCA_017916195.1
GCA_017916215.1
GCA_017916255.1
GCA_017922675.1
GCA_017922915.1
GCA_017922975.1
GCA_017923175.1
GCA_017923375.1
GCA_017927795.1
GCA_017933115.1
GCA_017933235.1
GCA_017933255.1
GCA_017945845.1
GCA_017945865.1
GCA_017948225.1
GCA_017948245.1
GCA_017948265.1
GCA_017948285.1
GCA_017948305.1
GCA_017948325.1
GCA_017948345.1
GCA_017948365.1
GCA_017948385.1
GCA_017948405.1
GCA_017968845.1
GCA_017968865.1
GCA_017968885.1
GCA_017976505.1
GCA_018024475.1
GCA_018024495.1
GCA_018064205.1
GCA_018064225.1
GCA_018069625.1
GCA_018069645.1
GCA_018069665.1
GCA_018069705.1
GCA_018074945.1
GCA_018075005.1
GCA_018075085.1
GCA_018075145.1
GCA_018075245.1
GCA_018075265.1
GCA_018075285.1
GCA_018075305.1
GCA_018075325.1
GCA_018075345.1
GCA_018075365.1
GCA_018080005.1
GCA_018085225.1

GCA_018093065.1
GCA_018093105.1
GCA_018093205.1
GCA_018093225.1
GCA_018106265.1
GCA_018106285.1
GCA_018106305.1
GCA_018106325.1
GCA_018107685.1
GCA_018114805.1
GCA_018127705.1
GCA_018127725.1
GCA_018127745.1
GCA_018127765.1
GCA_018127785.1
GCA_018127805.1
GCA_018127825.1
GCA_018127845.1
GCA_018127865.1
GCA_018127885.1
GCA_018127905.1
GCA_018127925.1
GCA_018127945.1
GCA_018127965.1
GCA_018127985.1
GCA_018128005.1
GCA_018128045.1
GCA_018128065.1
GCA_018128085.1
GCA_018128105.1
GCA_018128125.1
GCA_018128145.1
GCA_018128165.1
GCA_018128185.1
GCA_018128205.1
GCA_018128225.1
GCA_018128245.1
GCA_018128265.1
GCA_018128285.1
GCA_018128305.1
GCA_018128325.1
GCA_018128345.1
GCA_018128365.1
GCA_018128405.1
GCA_018128425.1
GCA_018128445.1
GCA_018128465.1
GCA_018128905.1
GCA_018135545.1
GCA_018135565.1
GCA_018135585.1
GCA_018135605.1
GCA_018135625.1
GCA_018135645.1

GCA_018135955.1
GCA_018137945.1
GCA_018137965.1
GCA_018137985.1
GCA_018138045.1
GCA_018138065.1
GCA_018138085.1
GCA_018138105.1
GCA_018138125.1
GCA_018138305.2
GCA_018138925.1
GCA_018138945.1
GCA_018138965.1
GCA_018138985.1
GCA_018139005.1
GCA_018139025.1
GCA_018139045.1
GCA_018139065.1
GCA_018139085.1
GCA_018139105.1
GCA_018139125.1
GCA_018140535.1
GCA_018140555.1
GCA_018140575.1
GCA_018140595.1
GCA_018140615.1
GCA_018140655.1
GCA_018140675.1
GCA_018140905.1
GCA_018140925.1
GCA_018140965.1
GCA_018140985.1
GCA_018141005.1
GCA_018141025.1
GCA_018141045.1
GCA_018141085.1
GCA_018141105.1
GCA_018141125.1
GCA_018141145.1
GCA_018141165.1
GCA_018141205.1
GCA_018141225.1
GCA_018141245.1
GCA_018141265.1
GCA_018141285.1
GCA_018141305.1
GCA_018141325.1
GCA_018141345.1
GCA_018141365.1
GCA_018141385.1
GCA_018141425.1
GCA_018141445.1
GCA_018141465.1
GCA_018141485.1

GCA_018141505.2
GCA_018141525.1
GCA_018141545.1
GCA_018141565.1
GCA_018141585.1
GCA_018141605.1
GCA_018141625.1
GCA_018141645.1
GCA_018141665.1
GCA_018141705.1
GCA_018141745.1
GCA_018141765.1
GCA_018141785.1
GCA_018141805.1
GCA_018176575.1
GCA_018176655.1
GCA_018176755.1
GCA_018176875.1
GCA_018176975.1
GCA_018195715.2
GCA_018199955.1
GCA_018199975.1
GCA_018199995.1
GCA_018200035.1
GCA_018204675.1
GCA_018205975.1
GCA_018207515.1
GCA_018208355.1
GCA_018208375.1
GCA_018219155.1
GCA_018219175.1
GCA_018219225.1
GCA_018219245.1
GCA_018219265.1
GCA_018219285.1
GCA_018219325.1
GCA_018219415.1
GCA_018219435.1
GCA_018219455.1
GCA_018223345.1
GCA_018223585.1
GCA_018223605.1
GCA_018223625.1
GCA_018223665.1
GCA_018223685.1
GCA_018223705.1
GCA_018223725.1
GCA_018223745.1
GCA_018223765.1
GCA_018223785.1
GCA_018223805.1
GCA_018223845.1
GCA_018228345.1
GCA_018228385.1

GCA_018228525.1
GCA_018228545.1
GCA_018228565.1
GCA_018228585.1
GCA_018228605.1
GCA_018228625.1
GCA_018228725.1
GCA_018228745.1
GCA_018243215.1
GCA_018243235.1
GCA_018255755.1
GCA_018255775.1
GCA_018255795.1
GCA_018255815.1
GCA_018255835.1
GCA_018255875.1
GCA_018263855.1
GCA_018274525.2
GCA_018278725.1
GCA_018278905.1
GCA_018278985.1
GCA_018279085.1
GCA_018279125.1
GCA_018279145.1
GCA_018279165.1
GCA_018279185.1
GCA_018279205.1
GCA_018279225.1
GCA_018279245.1
GCA_018279265.1
GCA_018279285.1
GCA_018279305.1
GCA_018279325.1
GCA_018279465.1
GCA_018279565.1
GCA_018279585.1
GCA_018279705.1
GCA_018279805.1
GCA_018279895.1
GCA_018281725.1
GCA_018282115.1
GCA_018283505.2
GCA_018283575.2
GCA_018286375.1
GCA_018286435.1
GCA_018286575.1
GCA_018286635.1
GCA_018286655.1
GCA_018288775.1
GCA_018288975.1
GCA_018289035.1
GCA_018289135.1
GCA_018289175.1
GCA_018289275.1

GCA_018289295.1
GCA_018289315.1
GCA_018289335.1
GCA_018289355.1
GCA_018289375.1
GCA_018291785.1
GCA_018291825.1
GCA_018291945.1
GCA_018292045.1
GCA_018292105.1
GCA_018292125.1
GCA_018292145.1
GCA_018292165.1
GCA_018292185.1
GCA_018292205.1
GCA_018292225.1
GCA_018292245.1
GCA_018292265.1
GCA_018304665.1
GCA_018304685.1
GCA_018304705.1
GCA_018304725.1
GCA_018304745.1
GCA_018304765.1
GCA_018304785.1
GCA_018304805.1
GCA_018304825.1
GCA_018308945.1
GCA_018309085.1
GCA_018309125.1
GCA_018309145.1
GCA_018309165.1
GCA_018310415.1
GCA_018310435.1
GCA_018310455.1
GCA_018310495.1
GCA_018310515.1
GCA_018310535.1
GCA_018310555.1
GCA_018310575.1
GCA_018314115.1
GCA_018314145.1
GCA_018314255.1
GCA_018316615.1
GCA_018316655.1
GCA_018316795.1
GCA_018316915.2
GCA_018323885.1
GCA_018323945.1
GCA_018324045.1
GCA_018324105.1
GCA_018324205.1
GCA_018324255.1
GCA_018324325.1

GCA_018324385.1
GCA_018324425.1
GCA_018324505.1
GCA_018324565.1
GCA_018324625.1
GCA_018324685.1
GCA_018325805.1
GCA_018325865.1
GCA_018325905.1
GCA_018325965.1
GCA_018326005.1
GCA_018326065.1
GCA_018326105.1
GCA_018326145.1
GCA_018326245.1
GCA_018326265.1
GCA_018326285.1
GCA_018326305.1
GCA_018326325.1
GCA_018326345.1
GCA_018326365.1
GCA_018326405.1
GCA_018326425.1
GCA_018326605.1
GCA_018336255.1
GCA_018336275.1
GCA_018336295.1
GCA_018336315.1
GCA_018336415.1
GCA_018336435.1
GCA_018336495.1
GCA_018336515.1
GCA_018336535.1
GCA_018336555.1
GCA_018336635.1
GCA_018336655.1
GCA_018336675.1
GCA_018336695.1
GCA_018336715.1
GCA_018336735.1
GCA_018336755.1
GCA_018336775.1
GCA_018336795.1
GCA_018336815.1
GCA_018336835.1
GCA_018336855.1
GCA_018336875.1
GCA_018336915.1
GCA_018336935.1
GCA_018336995.1
GCA_018337015.1
GCA_018338735.1
GCA_018340585.1
GCA_018343775.1

GCA_018343795.1
GCA_018343815.1
GCA_018343835.1
GCA_018343855.1
GCA_018350285.1
GCA_018351295.1
GCA_018361045.1
GCA_018361065.1
GCA_018361085.1
GCA_018361105.1
GCA_018361125.1
GCA_018361145.1
GCA_018361165.1
GCA_018361185.1
GCA_018361205.1
GCA_018361225.1
GCA_018361245.1
GCA_018361365.1
GCA_018362975.1
GCA_018362995.1
GCA_018363015.1
GCA_018363035.1
GCA_018363055.1
GCA_018363075.1
GCA_018363095.1
GCA_018388425.1
GCA_018388445.1
GCA_018388465.1
GCA_018388485.1
GCA_018388505.1
GCA_018388525.1
GCA_018388545.1
GCA_018388565.1
GCA_018388585.1
GCA_018388625.1
GCA_018388785.1
GCA_018389385.1
GCA_018389405.1
GCA_018389545.1
GCA_018389565.1
GCA_018389585.1
GCA_018389605.1
GCA_018389625.1
GCA_018389645.1
GCA_018389665.1
GCA_018389685.1
GCA_018389705.1
GCA_018389725.1
GCA_018389745.1
GCA_018389765.1
GCA_018389805.1
GCA_018389825.1
GCA_018394055.1
GCA_018394175.1

GCA_018394295.1
GCA_018394315.1
GCA_018394335.1
GCA_018394355.1
GCA_018394375.1
GCA_018394395.1
GCA_018394415.1
GCA_018398475.1
GCA_018398935.1
GCA_018398955.1
GCA_018398975.1
GCA_018398995.1
GCA_018399015.1
GCA_018399035.1
GCA_018399055.1
GCA_018399075.1
GCA_018399095.1
GCA_018399115.1
GCA_018402785.1
GCA_018402805.1
GCA_018402825.1
GCA_018402845.1
GCA_018403805.1
GCA_018403825.1
GCA_018406445.1
GCA_018406465.1
GCA_018406485.1
GCA_018406505.1
GCA_018406545.1
GCA_018406605.1
GCA_018406645.1
GCA_018408275.1
GCA_018408455.1
GCA_018408575.1
GCA_018408705.1
GCA_018409035.1
GCA_018409145.1
GCA_018409365.1
GCA_018409405.1
GCA_018409485.1
GCA_018409545.1
GCA_018415955.1
GCA_018415975.1
GCA_018415995.1
GCA_018417475.1
GCA_018417515.1
GCA_018417535.1
GCA_018417555.1
GCA_018417575.1
GCA_018417595.1
GCA_018417635.1
GCA_018417715.1
GCA_018436065.1
GCA_018436125.1

GCA_018436245.1
GCA_018437505.1
GCA_018437525.1
GCA_018437695.1
GCA_018437745.1
GCA_018438165.1
GCA_018448495.1
GCA_018448985.1
GCA_018449005.1
GCA_018449025.1
GCA_018449045.1
GCA_018449065.1
GCA_018454365.1
GCA_018454385.1
GCA_018454405.1
GCA_018454425.1
GCA_018459925.1
GCA_018460045.1
GCA_018460065.1
GCA_018467115.1
GCA_018467135.1
GCA_018487825.1
GCA_018491625.2
GCA_018491735.2
GCA_018499805.2
GCA_018499845.2
GCA_018502505.1
GCA_018502525.1
GCA_018502565.1
GCA_018502585.1
GCA_018502625.1
GCA_018511305.1
GCA_018516745.1
GCA_018516785.1
GCA_018516845.1
GCA_018516925.1
GCA_018517025.1
GCA_018517045.1
GCA_018517065.1
GCA_018517085.1
GCA_018517105.1
GCA_018517125.1
GCA_018517145.1
GCA_018517165.1
GCA_018517185.1
GCA_018517205.1
GCA_018540425.1
GCA_018582665.1
GCA_018588605.2
GCA_018588615.2
GCA_018588665.2
GCA_018595675.2
GCA_018595685.2
GCA_018596355.1

GCA_018596375.1
GCA_018596855.2
GCA_018598285.3
GCA_018603395.1
GCA_018603435.1
GCA_018603455.1
GCA_018603475.1
GCA_018603495.1
GCA_018604105.1
GCA_018604125.1
GCA_018604145.1
GCA_018604165.1
GCA_018604185.1
GCA_018604205.1
GCA_018604225.1
GCA_018604245.1
GCA_018604265.1
GCA_018604285.1
GCA_018604305.1
GCA_018604325.1
GCA_018604345.1
GCA_018604365.1
GCA_018604385.1
GCA_018604545.1
GCA_018604565.1
GCA_018604625.1
GCA_018604665.1
GCA_018604685.1
GCA_018604745.1
GCA_018604765.1
GCA_018622495.1
GCA_018622615.1
GCA_018622675.1
GCA_018622755.1
GCA_018622855.1
GCA_018622975.1
GCA_018622995.1
GCA_018623015.1
GCA_018623035.1
GCA_018623335.1
GCA_018628815.1
GCA_018629015.1
GCA_018634035.1
GCA_018636735.1
GCA_018638795.1
GCA_018672315.1
GCA_018672415.1
GCA_018672515.1
GCA_018685275.1
GCA_018685335.1
GCA_018685415.1
GCA_018685455.1
GCA_018685555.1
GCA_018685605.1

GCA_018687075.1
GCA_018687195.1
GCA_018687275.1
GCA_018687415.1
GCA_018687455.1
GCA_018687475.1
GCA_018687495.1
GCA_018687515.1
GCA_018687535.1
GCA_018687555.1
GCA_018687575.1
GCA_018687595.1
GCA_018687615.1
GCA_018687675.1
GCA_018687735.1
GCA_018687815.1
GCA_018687975.1
GCA_018688155.1
GCA_018688255.1
GCA_018688315.1
GCA_018688335.1
GCA_018688375.1
GCA_018688455.1
GCA_018689955.1
GCA_018690035.1
GCA_018690095.1
GCA_018729255.1
GCA_018731965.1
GCA_018732085.1
GCA_018732145.1
GCA_018732165.1
GCA_018732185.1
GCA_018732205.1
GCA_018732225.1
GCA_018732245.1
GCA_018733955.1
GCA_018734065.1
GCA_018734165.1
GCA_018734225.1
GCA_018734265.1
GCA_018734285.1
GCA_018734305.1
GCA_018734325.1
GCA_018735785.1
GCA_018735945.1
GCA_018736025.1
GCA_018736045.1
GCA_018736065.1
GCA_018736085.1
GCA_018736105.1
GCA_018736125.1
GCA_018736145.1
GCA_018736705.1
GCA_018736725.1

GCA_018736745.1
GCA_018736765.1
GCA_018739305.1
GCA_018739365.1
GCA_018739385.1
GCA_018739445.1
GCA_018739485.1
GCA_018739505.1
GCA_018739525.1
GCA_018739545.1
GCA_018739605.1
GCA_018739625.1
GCA_018739645.1
GCA_018739665.1
GCA_018739685.1
GCA_018739705.1
GCA_018740945.1
GCA_018741005.1
GCA_018741085.1
GCA_018741125.1
GCA_018741145.1
GCA_018741165.1
GCA_018741285.1
GCA_018741405.1
GCA_018741465.1
GCA_018741505.1
GCA_018741525.1
GCA_018741545.1
GCA_018741565.1
GCA_018741585.1
GCA_018741605.1
GCA_018741625.1
GCA_018741645.1
GCA_018741985.1
GCA_018742045.1
GCA_018742105.1
GCA_018742185.1
GCA_018742205.1
GCA_018742225.1
GCA_018742245.1
GCA_018742265.1
GCA_018742285.1
GCA_018751995.1
GCA_018771265.1
GCA_018771365.1
GCA_018771465.1
GCA_018771545.1
GCA_018771565.1
GCA_018771585.1
GCA_018771605.1
GCA_018771625.1
GCA_018771645.1
GCA_018771665.1
GCA_018771685.1

GCA_018771705.1
GCA_018775385.1
GCA_018779945.3
GCA_018798845.1
GCA_018798865.1
GCA_018798885.1
GCA_018798905.1
GCA_018802225.1
GCA_018802245.1
GCA_018802265.1
GCA_018802285.1
GCA_018802305.1
GCA_018802325.1
GCA_018802345.1
GCA_018802365.1
GCA_018802385.1
GCA_018802405.1
GCA_018802425.1
GCA_018802445.1
GCA_018802465.1
GCA_018802485.1
GCA_018802505.1
GCA_018802525.1
GCA_018802545.1
GCA_018802565.1
GCA_018802585.1
GCA_018802605.1
GCA_018802645.1
GCA_018802765.1
GCA_018808925.1
GCA_018831125.1
GCA_018831285.1
GCA_018831305.1
GCA_018831325.1
GCA_018831345.1
GCA_018831365.1
GCA_018831385.1
GCA_018831405.1
GCA_018831425.1
GCA_018831445.1
GCA_018831465.1
GCA_018831485.1
GCA_018831525.1
GCA_018831545.1
GCA_018831565.1
GCA_018831585.1
GCA_018831605.1
GCA_018831625.1
GCA_018845095.1
GCA_018846995.1
GCA_018847015.1
GCA_018847035.1
GCA_018847055.1
GCA_018847075.1

GCA_018847095.1
GCA_018847115.1
GCA_018847135.1
GCA_018847155.1
GCA_018847175.1
GCA_018847195.1
GCA_018847215.1
GCA_018847235.1
GCA_018847255.1
GCA_018847275.1
GCA_018847295.1
GCA_018847315.1
GCA_018847335.1
GCA_018847355.1
GCA_018847375.1
GCA_018847395.1
GCA_018847415.1
GCA_018847435.1
GCA_018847455.1
GCA_018847475.1
GCA_018847495.1
GCA_018847515.1
GCA_018847535.1
GCA_018847555.1
GCA_018847575.1
GCA_018847595.1
GCA_018847615.1
GCA_018847695.1
GCA_018847755.1
GCA_018847795.1
GCA_018847895.1
GCA_018847935.1
GCA_018847975.1
GCA_018861215.2
GCA_018861235.2
GCA_018861255.2
GCA_018861275.1
GCA_018866205.1
GCA_018866225.1
GCA_018866245.1
GCA_018866265.1
GCA_018866305.1
GCA_018866325.1
GCA_018866345.1
GCA_018866365.1
GCA_018873185.1
GCA_018884065.1
GCA_018884105.1
GCA_018884125.1
GCA_018884165.1
GCA_018884185.1
GCA_018884205.1
GCA_018884225.1
GCA_018884245.1

GCA_018884265.1
GCA_018884285.1
GCA_018884305.1
GCA_018884325.1
GCA_018884345.1
GCA_018884365.1
GCA_018884385.1
GCA_018884405.1
GCA_018884425.1
GCA_018884445.1
GCA_018884465.1
GCA_018884485.1
GCA_018884505.1
GCA_018884525.1
GCA_018884545.1
GCA_018884565.1
GCA_018884585.1
GCA_018884605.1
GCA_018884665.1
GCA_018884705.1
GCA_018884725.1
GCA_018884745.1
GCA_018884765.1
GCA_018884825.1
GCA_018884845.1
GCA_018884865.1
GCA_018884885.1
GCA_018884905.1
GCA_018884925.1
GCA_018884945.1
GCA_018884965.1
GCA_018885045.1
GCA_018885065.1
GCA_018885085.1
GCA_018885105.1
GCA_018885125.1
GCA_018885145.1
GCA_018885165.1
GCA_018885185.1
GCA_018885205.1
GCA_018885225.1
GCA_018885245.1
GCA_018885265.1
GCA_018885285.1
GCA_018885305.1
GCA_018885325.1
GCA_018885345.1
GCA_018885365.1
GCA_018885385.1
GCA_018889915.1
GCA_018889955.1
GCA_018889995.1
GCA_018904205.1
GCA_018905915.1

GCA_018906035.1
GCA_018924625.1
GCA_018966485.1
GCA_018966505.1
GCA_018966525.1
GCA_018966765.1
GCA_018966785.1
GCA_018966805.1
GCA_018966825.1
GCA_018966845.1
GCA_018966865.1
GCA_018968645.1
GCA_018968665.1
GCA_018968685.1
GCA_018968705.1
GCA_018968725.1
GCA_018968745.1
GCA_018972025.2
GCA_018972045.1
GCA_018972085.1
GCA_018972105.1
GCA_018972125.1
GCA_018972145.1
GCA_018972165.1
GCA_018972185.1
GCA_018972205.1
GCA_018972225.1
GCA_018972245.1
GCA_018972265.1
GCA_018982885.2
GCA_018986715.1
GCA_018986735.1
GCA_018986755.2
GCA_018986795.1
GCA_018986815.1
GCA_018986835.1
GCA_018986855.1
GCA_018986875.1
GCA_018986895.1
GCA_018986915.1
GCA_018986935.1
GCA_018987235.1
GCA_018987265.1
GCA_018987285.1
GCA_018987305.1
GCA_018987325.1
GCA_018987345.1
GCA_018987365.1
GCA_018988345.1
GCA_018988385.1
GCA_019021805.1
GCA_019021925.1
GCA_019038085.1
GCA_019038105.1

GCA_019038575.1
GCA_019039255.1
GCA_019039795.1
GCA_019046905.1
GCA_019046925.1
GCA_019046945.1
GCA_019046965.1
GCA_019046985.1
GCA_019047025.1
GCA_019047045.1
GCA_019047065.1
GCA_019047085.1
GCA_019047105.1
GCA_019047125.1
GCA_019047185.1
GCA_019047205.1
GCA_019047325.1
GCA_019047465.1
GCA_019047505.1
GCA_019047545.1
GCA_019047765.1
GCA_019047785.1
GCA_019047805.1
GCA_019047825.1
GCA_019047885.1
GCA_019047925.1
GCA_019047945.1
GCA_019047985.1
GCA_019048025.1
GCA_019048045.1
GCA_019048065.1
GCA_019048085.1
GCA_019048105.1
GCA_019048125.1
GCA_019048165.1
GCA_019048185.1
GCA_019048225.1
GCA_019048245.1
GCA_019048265.1
GCA_019048305.1
GCA_019048365.1
GCA_019048385.1
GCA_019048405.1
GCA_019048465.1
GCA_019048485.1
GCA_019048525.1
GCA_019048545.1
GCA_019048565.1
GCA_019048585.1
GCA_019048625.1
GCA_019048645.1
GCA_019048665.1
GCA_019048685.1
GCA_019048705.1

GCA_019048725.1
GCA_019048745.1
GCA_019048805.1
GCA_019048825.1
GCA_019048845.1
GCA_019048865.1
GCA_019048885.1
GCA_019048905.1
GCA_019048925.1
GCA_019048945.1
GCA_019048965.1
GCA_019048985.1
GCA_019056395.1
GCA_019056415.1
GCA_019056455.1
GCA_019056475.1
GCA_019056495.1
GCA_019056515.1
GCA_019056535.1
GCA_019056555.1
GCA_019056575.1
GCA_019056595.1
GCA_019056615.1
GCA_019056635.1
GCA_019056655.1
GCA_019056675.1
GCA_019063845.1
GCA_019063885.1
GCA_019063905.1
GCA_019076685.1
GCA_019076805.1
GCA_019076885.1
GCA_019076985.1
GCA_019078745.1
GCA_019086825.1
GCA_019090985.1
GCA_019091005.1
GCA_019095205.1
GCA_019095765.1
GCA_019107685.1
GCA_019107705.1
GCA_019112525.1
GCA_019134555.1
GCA_019134595.1
GCA_019134615.1
GCA_019134635.1
GCA_019134655.1
GCA_019134675.1
GCA_019134695.1
GCA_019134715.1
GCA_019134735.1
GCA_019134755.1
GCA_019134775.1
GCA_019134795.1

GCA_019134815.1
GCA_019134835.1
GCA_019137555.1
GCA_019137575.1
GCA_019139535.1
GCA_019139675.1
GCA_019139795.1
GCA_019139815.1
GCA_019139835.1
GCA_019139855.1
GCA_019139875.1
GCA_019139895.1
GCA_019141525.1
GCA_019141545.1
GCA_019163475.1
GCA_019164215.1
GCA_019175365.1
GCA_019175405.1
GCA_019175425.1
GCA_019175445.1
GCA_019175465.1
GCA_019175485.1
GCA_019175505.1
GCA_019175525.1
GCA_019182265.1
GCA_019186805.1
GCA_019192625.1
GCA_019192645.1
GCA_019195415.1
GCA_019195435.1
GCA_019203145.1
GCA_019203165.1
GCA_019203185.1
GCA_019203205.1
GCA_019203745.1
GCA_019203765.1
GCA_019203785.1
GCA_019203805.1
GCA_019203945.1
GCA_019203965.1
GCA_019211725.1
GCA_019211745.1
GCA_019211765.1
GCA_019211785.1
GCA_019211805.1
GCA_019211825.1
GCA_019211845.1
GCA_019211865.1
GCA_019211885.1
GCA_019212025.1
GCA_019212165.1
GCA_019222685.1
GCA_019222705.1
GCA_019222725.1

GCA_019222745.1
GCA_019222765.1
GCA_019222785.1
GCA_019222805.1
GCA_019226825.1
GCA_019242835.1
GCA_019242955.1
GCA_019243055.1
GCA_019243165.1
GCA_019243235.1
GCA_019243335.1
GCA_019243435.1
GCA_019243485.1
GCA_019243605.1
GCA_019243695.1
GCA_019243775.1
GCA_019252525.1
GCA_019263765.1
GCA_019263785.1
GCA_019263805.1
GCA_019264705.1
GCA_019264725.1
GCA_019272915.1
GCA_019272935.1
GCA_019278425.1
GCA_019278445.1
GCA_019278465.1
GCA_019278485.1
GCA_019278505.1
GCA_019278525.1
GCA_019278545.1
GCA_019278565.1
GCA_019278585.1
GCA_019285515.1
GCA_019285535.1
GCA_019285555.1
GCA_019285575.1
GCA_019285595.1
GCA_019285615.1
GCA_019285655.1
GCA_019285675.1
GCA_019285695.1
GCA_019285715.1
GCA_019285735.1
GCA_019285755.1
GCA_019285775.1
GCA_019286375.1
GCA_019286455.1
GCA_019286515.1
GCA_019286635.1
GCA_019286695.1
GCA_019286755.1
GCA_019286855.1
GCA_019286955.1

GCA_019287035.1
GCA_019287095.1
GCA_019287195.1
GCA_019287315.1
GCA_019295365.2
GCA_019297755.1
GCA_019297775.1
GCA_019297795.1
GCA_019297815.1
GCA_019297835.1
GCA_019297855.1
GCA_019316745.1
GCA_019316905.1
GCA_019316985.1
GCA_019317005.1
GCA_019317025.1
GCA_019317045.1
GCA_019317065.1
GCA_019317085.1
GCA_019317105.1
GCA_019317125.1
GCA_019317145.1
GCA_019317165.1
GCA_019317185.1
GCA_019317205.1
GCA_019317225.1
GCA_019317245.1
GCA_019317265.1
GCA_019317285.1
GCA_019317325.1
GCA_019317345.1
GCA_019321785.1
GCA_019321805.1
GCA_019329245.1
GCA_019329325.1
GCA_019329365.1
GCA_019329405.1
GCA_019329425.1
GCA_019329505.1
GCA_019329525.1
GCA_019329545.1
GCA_019329565.1
GCA_019329585.1
GCA_019329605.1
GCA_019329625.1
GCA_019329645.1
GCA_019329665.1
GCA_019329685.1
GCA_019329705.1
GCA_019329725.1
GCA_019329745.1
GCA_019329765.1
GCA_019329785.1
GCA_019329805.1

GCA_019330045.1
GCA_019330105.1
GCA_019330125.1
GCA_019330145.1
GCA_019330165.1
GCA_019330185.1
GCA_019330205.1
GCA_019330225.1
GCA_019330245.1
GCA_019330265.1
GCA_019331605.1
GCA_019331655.1
GCA_019332005.1
GCA_019334125.1
GCA_019334145.1
GCA_019334365.1
GCA_019334385.1
GCA_019334405.1
GCA_019334425.1
GCA_019334445.1
GCA_019334485.1
GCA_019334505.1
GCA_019334525.1
GCA_019334545.1
GCA_019334565.1
GCA_019334585.1
GCA_019334605.1
GCA_019334625.1
GCA_019334645.1
GCA_019334665.1
GCA_019334685.1
GCA_019334705.1
GCA_019334725.1
GCA_019334745.1
GCA_019334765.1
GCA_019334785.1
GCA_019334805.1
GCA_019334825.1
GCA_019339365.1
GCA_019339425.1
GCA_019339445.1
GCA_019339465.1
GCA_019339485.1
GCA_019339505.1
GCA_019342845.1
GCA_019342865.1
GCA_019342885.1
GCA_019342905.1
GCA_019342925.1
GCA_019342945.1
GCA_019342965.1
GCA_019342985.1
GCA_019343005.1
GCA_019343025.1

GCA_019343045.1
GCA_019343065.1
GCA_019343085.1
GCA_019343105.1
GCA_019343125.1
GCA_019343455.1
GCA_019343475.1
GCA_019343495.1
GCA_019344315.1
GCA_019355055.1
GCA_019355135.1
GCA_019355235.1
GCA_019355355.1
GCA_019355375.1
GCA_019355395.1
GCA_019355415.1
GCA_019355435.1
GCA_019355455.1
GCA_019355475.1
GCA_019355495.1
GCA_019355535.1
GCA_019355555.1
GCA_019355575.1
GCA_019355615.1
GCA_019355735.1
GCA_019355815.1
GCA_019355895.1
GCA_019356015.1
GCA_019356035.1
GCA_019356055.1
GCA_019356075.1
GCA_019356215.1
GCA_019356235.1
GCA_019356355.1
GCA_019356415.1
GCA_019356535.1
GCA_019357415.1
GCA_019357435.1
GCA_019357455.1
GCA_019357475.1
GCA_019357495.1
GCA_019357515.1
GCA_019357535.1
GCA_019357555.1
GCA_019357575.1
GCA_019357695.1
GCA_019364615.1
GCA_019375055.2
GCA_019378875.1
GCA_019378895.1
GCA_019378915.1
GCA_019378935.1
GCA_019379355.1
GCA_019380255.1

GCA_019380275.1
GCA_019395145.1
GCA_019395165.1
GCA_019396865.1
GCA_019396925.1
GCA_019397265.1
GCA_019399915.1
GCA_019404925.1
GCA_019405085.1
GCA_019425695.1
GCA_019426245.1
GCA_019426265.1
GCA_019428465.1
GCA_019428485.1
GCA_019428505.1
GCA_019428525.1
GCA_019428545.1
GCA_019428565.1
GCA_019428585.1
GCA_019428605.1
GCA_019428625.1
GCA_019428645.1
GCA_019428665.1
GCA_019428685.1
GCA_019428705.1
GCA_019428725.1
GCA_019428745.1
GCA_019428765.1
GCA_019428785.1
GCA_019431145.1
GCA_019431165.1
GCA_019431185.1
GCA_019431205.1
GCA_019431245.1
GCA_019431295.1
GCA_019431315.1
GCA_019431335.1
GCA_019431355.1
GCA_019434075.1
GCA_019434095.1
GCA_019434115.1
GCA_019434135.1
GCA_019434155.1
GCA_019434175.1
GCA_019434195.1
GCA_019434215.1
GCA_019434235.1
GCA_019434255.1
GCA_019434275.1
GCA_019434295.1
GCA_019434315.1
GCA_019434335.1
GCA_019434355.1
GCA_019434375.1

GCA_019443265.1
GCA_019443285.1
GCA_019443305.1
GCA_019443325.1
GCA_019443365.1
GCA_019443405.1
GCA_019443425.1
GCA_019443445.1
GCA_019443465.1
GCA_019443485.1
GCA_019443505.1
GCA_019443525.1
GCA_019443545.1
GCA_019443565.1
GCA_019443585.1
GCA_019443605.1
GCA_019443665.1
GCA_019443685.1
GCA_019443705.1
GCA_019443725.1
GCA_019443745.1
GCA_019443765.1
GCA_019443785.1
GCA_019443805.1
GCA_019443825.1
GCA_019443845.1
GCA_019443925.1
GCA_019443945.1
GCA_019443965.1
GCA_019444095.1
GCA_019444145.1
GCA_019444195.1
GCA_019444635.1
GCA_019448175.1
GCA_019448195.1
GCA_019448235.1
GCA_019448255.1
GCA_019448315.1
GCA_019448355.1
GCA_019449395.1
GCA_019453995.1
GCA_019454065.1
GCA_019454085.1
GCA_019454345.1
GCA_019454385.1
GCA_019455345.1
GCA_019455365.1
GCA_019456555.1
GCA_019456575.1
GCA_019456595.1
GCA_019456615.1
GCA_019456655.1
GCA_019456675.1
GCA_019456695.2

GCA_019457435.1
GCA_019457455.1
GCA_019457475.1
GCA_019457495.1
GCA_019457515.1
GCA_019457535.1
GCA_019457555.1
GCA_019457575.1
GCA_019457595.1
GCA_019457615.1
GCA_019457635.1
GCA_019457655.1
GCA_019457715.1
GCA_019458445.1
GCA_019458465.1
GCA_019458485.1
GCA_019458525.1
GCA_019464335.1
GCA_019464375.1
GCA_019464415.1
GCA_019464455.1
GCA_019464495.1
GCA_019464535.1
GCA_019464555.1
GCA_019464575.1
GCA_019464595.1
GCA_019464635.1
GCA_019466035.1
GCA_019466095.1
GCA_019466145.1
GCA_019468565.1
GCA_019469165.1
GCA_019469185.1
GCA_019469205.1
GCA_019469225.1
GCA_019469245.1
GCA_019469265.1
GCA_019469325.1
GCA_019469345.1
GCA_019469365.1
GCA_019469425.1
GCA_019469465.1
GCA_019469485.1
GCA_019469505.1
GCA_019469525.1
GCA_019469545.1
GCA_019469565.1
GCA_019504365.1
GCA_019504385.1
GCA_019504425.1
GCA_019550795.1
GCA_019550875.1
GCA_019550945.1
GCA_019551035.1

GCA_019551095.1
GCA_019551175.1
GCA_019551235.1
GCA_019551315.1
GCA_019551355.1
GCA_019551375.1
GCA_019551415.1
GCA_019551475.1
GCA_019551675.1
GCA_019551735.1
GCA_019551815.1
GCA_019551835.1
GCA_019551855.1
GCA_019551945.1
GCA_019552005.1
GCA_019552045.1
GCA_019552065.1
GCA_019552085.1
GCA_019552105.1
GCA_019552125.1
GCA_019552145.1
GCA_019552165.1
GCA_019552185.1
GCA_019552205.1
GCA_019552325.1
GCA_019552345.1
GCA_019552405.1
GCA_019565455.1
GCA_019575955.1
GCA_019575995.1
GCA_019576055.1
GCA_019576095.1
GCA_019576155.1
GCA_019581175.1
GCA_019597925.1
GCA_019598965.1
GCA_019599005.1
GCA_019599025.1
GCA_019599045.1
GCA_019599065.1
GCA_019599085.1
GCA_019599105.1
GCA_019599125.1
GCA_019599145.1
GCA_019602835.1
GCA_019602855.1
GCA_019603315.1
GCA_019603335.1
GCA_019603355.1
GCA_019603375.1
GCA_019613795.1
GCA_019613955.1
GCA_019614295.2
GCA_019614475.1

GCA_019614655.1
GCA_019614825.1
GCA_019623805.1
GCA_019623905.1
GCA_019645795.1
GCA_019645815.1
GCA_019645835.1
GCA_019645855.1
GCA_019645875.1
GCA_019645895.1
GCA_019645915.1
GCA_019645935.1
GCA_019645955.1
GCA_019645975.1
GCA_019645995.1
GCA_019646015.1
GCA_019646095.1
GCA_019653615.1
GCA_019665765.1
GCA_019665785.1
GCA_019665805.1
GCA_019668485.1
GCA_019668505.1
GCA_019668525.1
GCA_019668545.1
GCA_019668565.1
GCA_019668585.1
GCA_019668605.1
GCA_019668625.1
GCA_019668645.1
GCA_019668665.1
GCA_019668685.1
GCA_019668705.1
GCA_019668825.1
GCA_019668925.1
GCA_019669005.1
GCA_019669025.1
GCA_019669085.1
GCA_019669165.1
GCA_019669245.1
GCA_019669345.1
GCA_019669385.1
GCA_019669885.1
GCA_019669905.1
GCA_019669985.1
GCA_019670005.1
GCA_019670025.1
GCA_019670105.1
GCA_019670125.1
GCA_019670145.1
GCA_019670165.1
GCA_019670185.1
GCA_019670205.1
GCA_019670225.1

GCA_019670245.1
GCA_019670265.1
GCA_019670285.1
GCA_019670305.1
GCA_019670325.1
GCA_019670345.1
GCA_019670365.1
GCA_019670385.1
GCA_019670405.1
GCA_019670425.1
GCA_019670445.1
GCA_019670465.1
GCA_019670485.1
GCA_019670505.1
GCA_019670525.1
GCA_019670545.1
GCA_019670585.1
GCA_019670605.1
GCA_019670625.1
GCA_019670645.1
GCA_019670665.1
GCA_019670685.1
GCA_019670705.1
GCA_019670725.1
GCA_019670745.1
GCA_019693235.1
GCA_019693255.1
GCA_019702485.1
GCA_019702905.1
GCA_019702925.1
GCA_019702945.1
GCA_019702965.1
GCA_019702985.1
GCA_019703005.1
GCA_019703025.1
GCA_019703045.1
GCA_019703065.1
GCA_019703085.1
GCA_019703105.1
GCA_019703125.1
GCA_019703145.1
GCA_019703165.1
GCA_019703185.1
GCA_019703205.1
GCA_019703225.1
GCA_019703245.1
GCA_019703265.1
GCA_019703285.1
GCA_019703305.1
GCA_019703325.1
GCA_019703365.1
GCA_019703385.1
GCA_019703405.1
GCA_019703425.1

GCA_019703455.1
GCA_019703495.1
GCA_019703525.1
GCA_019703545.1
GCA_019703575.1
GCA_019703595.1
GCA_019703615.1
GCA_019703635.1
GCA_019703655.1
GCA_019703675.1
GCA_019703695.1
GCA_019703715.1
GCA_019703735.1
GCA_019703755.1
GCA_019703775.1
GCA_019703795.1
GCA_019703835.1
GCA_019703855.1
GCA_019703875.1
GCA_019703895.1
GCA_019703915.1
GCA_019703975.1
GCA_019703995.1
GCA_019704015.1
GCA_019704055.1
GCA_019704075.1
GCA_019704095.1
GCA_019704115.1
GCA_019704135.1
GCA_019704155.2
GCA_019704175.1
GCA_019704195.1
GCA_019704215.1
GCA_019704235.1
GCA_019704255.1
GCA_019704275.1
GCA_019704335.1
GCA_019704355.1
GCA_019704375.1
GCA_019704395.1
GCA_019704415.1
GCA_019704435.1
GCA_019704455.1
GCA_019704475.1
GCA_019704495.1
GCA_019704515.1
GCA_019704535.1
GCA_019704555.1
GCA_019704575.1
GCA_019710375.1
GCA_019710395.3
GCA_019710415.1
GCA_019710455.1
GCA_019710495.1

GCA_019710535.1
GCA_019711195.1
GCA_019711215.1
GCA_019720735.1
GCA_019720755.1
GCA_019720775.1
GCA_019720795.1
GCA_019720815.1
GCA_019720835.1
GCA_019720855.1
GCA_019720875.1
GCA_019722725.1
GCA_019738975.1
GCA_019738995.2
GCA_019739075.1
GCA_019739095.1
GCA_019739335.1
GCA_019740355.2
GCA_019754015.1
GCA_019754155.1
GCA_019754215.1
GCA_019754235.1
GCA_019754255.1
GCA_019754275.1
GCA_019774515.1
GCA_019774535.1
GCA_019774555.1
GCA_019774575.1
GCA_019774595.1
GCA_019774615.1
GCA_019774635.1
GCA_019787625.1
GCA_019787645.1
GCA_019787665.1
GCA_019793575.1
GCA_019793595.1
GCA_019793795.1
GCA_019793815.1
GCA_019797765.1
GCA_019797785.1
GCA_019797805.1
GCA_019797825.1
GCA_019797845.1
GCA_019797865.1
GCA_019797905.1
GCA_019797925.1
GCA_019797945.1
GCA_019797985.1
GCA_019798005.1
GCA_019798025.1
GCA_019798185.1
GCA_019802685.1
GCA_019802705.1
GCA_019802725.1

GCA_019802745.1
GCA_019802765.1
GCA_019802785.1
GCA_019803045.1
GCA_019803065.1
GCA_019803445.1
GCA_019822885.1
GCA_019823005.1
GCA_019823025.1
GCA_019823045.1
GCA_019823065.1
GCA_019823085.1
GCA_019823765.1
GCA_019823785.1
GCA_019823805.1
GCA_019823835.1
GCA_019823855.1
GCA_019823875.1
GCA_019823895.1
GCA_019823915.1
GCA_019823935.1
GCA_019823955.1
GCA_019843995.1
GCA_019844015.1
GCA_019844035.1
GCA_019844055.1
GCA_019844075.1
GCA_019844095.1
GCA_019856235.1
GCA_019856255.1
GCA_019856275.1
GCA_019856315.1
GCA_019856335.1
GCA_019856355.1
GCA_019856375.1
GCA_019856395.1
GCA_019856415.1
GCA_019856435.1
GCA_019856455.1
GCA_019856475.1
GCA_019856495.1
GCA_019856515.1
GCA_019856535.1
GCA_019856555.1
GCA_019856595.1
GCA_019856615.1
GCA_019857245.1
GCA_019857265.1
GCA_019857285.1
GCA_019857305.1
GCA_019857325.1
GCA_019857345.1
GCA_019857365.1
GCA_019857385.1

GCA_019857405.1
GCA_019857425.1
GCA_019857445.1
GCA_019857465.1
GCA_019857485.1
GCA_019857505.1
GCA_019857525.1
GCA_019857545.1
GCA_019857565.1
GCA_019857585.1
GCA_019857605.1
GCA_019857625.1
GCA_019857645.1
GCA_019873825.1
GCA_019879085.1
GCA_019880205.1
GCA_019880265.1
GCA_019880285.1
GCA_019880305.1
GCA_019880325.1
GCA_019880345.1
GCA_019880365.1
GCA_019880385.1
GCA_019880405.1
GCA_019880425.1
GCA_019880465.1
GCA_019880485.1
GCA_019884625.1
GCA_019884725.1
GCA_019884785.1
GCA_019884805.1
GCA_019890815.1
GCA_019890835.1
GCA_019890855.1
GCA_019890875.1
GCA_019890895.1
GCA_019890915.1
GCA_019890935.1
GCA_019890955.1
GCA_019890975.1
GCA_019890995.1
GCA_019891015.1
GCA_019891035.1
GCA_019891175.1
GCA_019891395.1
GCA_019895115.1
GCA_019896115.1
GCA_019900285.2
GCA_019900305.2
GCA_019900325.2
GCA_019900345.2
GCA_019900745.2
GCA_019900765.2
GCA_019900785.2

GCA_019900805.2
GCA_019900825.1
GCA_019900845.1
GCA_019903155.1
GCA_019903175.1
GCA_019903195.1
GCA_019903215.1
GCA_019903235.1
GCA_019904175.1
GCA_019904195.1
GCA_019904215.1
GCA_019904235.1
GCA_019904255.1
GCA_019904275.1
GCA_019904295.1
GCA_019904315.1
GCA_019915265.1
GCA_019915285.1
GCA_019915305.1
GCA_019915325.1
GCA_019915345.1
GCA_019915365.1
GCA_019915385.1
GCA_019915405.1
GCA_019915425.1
GCA_019915445.1
GCA_019915465.1
GCA_019915485.1
GCA_019915505.1
GCA_019915525.1
GCA_019915545.1
GCA_019915565.1
GCA_019915585.1
GCA_019915985.1
GCA_019916005.1
GCA_019916025.1
GCA_019916085.1
GCA_019923545.1
GCA_019923565.1
GCA_019923585.1
GCA_019924095.1
GCA_019924635.1
GCA_019928625.2
GCA_019930545.1
GCA_019930565.1
GCA_019930585.1
GCA_019930605.1
GCA_019930625.1
GCA_019930645.1
GCA_019930665.1
GCA_019930685.1
GCA_019930705.1
GCA_019930725.1
GCA_019930745.1

GCA_019930765.1
GCA_019930785.1
GCA_019930805.1
GCA_019930825.1
GCA_019930845.1
GCA_019930865.1
GCA_019930885.1
GCA_019930905.1
GCA_019930925.1
GCA_019930945.1
GCA_019930965.1
GCA_019930985.1
GCA_019931005.1
GCA_019931025.1
GCA_019931045.1
GCA_019931575.1
GCA_019931595.1
GCA_019931635.1
GCA_019931655.3
GCA_019931675.1
GCA_019931695.1
GCA_019931715.1
GCA_019931735.1
GCA_019931755.1
GCA_019933155.2
GCA_019933235.1
GCA_019967715.1
GCA_019967855.1
GCA_019967875.1
GCA_019967915.1
GCA_019967935.1
GCA_019967955.1
GCA_019967975.1
GCA_019967995.1
GCA_019968015.1
GCA_019968035.1
GCA_019968055.1
GCA_019968075.1
GCA_019968605.1
GCA_019968665.1
GCA_019968685.1
GCA_019968705.1
GCA_019968725.1
GCA_019968745.1
GCA_019968765.1
GCA_019968785.1
GCA_019968805.1
GCA_019968825.1
GCA_019968845.1
GCA_019968865.1
GCA_019968885.1
GCA_019968905.1
GCA_019969195.1
GCA_019969215.1

GCA_019969235.1
GCA_019969255.1
GCA_019969485.1
GCA_019969505.1
GCA_019969525.1
GCA_019969545.1
GCA_019969565.1
GCA_019969585.1
GCA_019969605.1
GCA_019969625.1
GCA_019969645.1
GCA_019970895.1
GCA_019970955.1
GCA_019970975.1
GCA_019970995.1
GCA_019971015.1
GCA_019971035.1
GCA_019971055.1
GCA_019971075.1
GCA_019971115.1
GCA_019972775.2
GCA_019973615.1
GCA_019973635.1
GCA_019973655.1
GCA_019973675.1
GCA_019973695.1
GCA_019973715.1
GCA_019973735.1
GCA_019973775.1
GCA_019973795.1
GCA_019973835.2
GCA_019973855.1
GCA_019973875.1
GCA_019973995.1
GCA_019974055.1
GCA_019974075.1
GCA_019974095.1
GCA_019974115.1
GCA_019974135.1
GCA_019974155.1
GCA_019974175.1
GCA_019974315.1
GCA_019974355.1
GCA_019974375.1
GCA_019974395.1
GCA_019974435.1
GCA_019976755.1
GCA_019976835.1
GCA_019976915.1
GCA_019976975.1
GCA_019977195.1
GCA_019977235.1
GCA_019977255.1
GCA_019977275.1

GCA_019977295.1
GCA_019977315.1
GCA_019977335.1
GCA_019977355.1
GCA_019977375.1
GCA_019977395.1
GCA_019977495.1
GCA_019977575.1
GCA_019977655.1
GCA_019977755.1
GCA_019977775.1
GCA_019977795.1
GCA_019977815.1
GCA_019977835.1
GCA_019997865.1
GCA_020002245.1
GCA_020002265.1
GCA_020002285.1
GCA_020002305.1
GCA_020002335.2
GCA_020003145.1
GCA_020003165.1
GCA_020008065.1
GCA_020008085.1
GCA_020008105.1
GCA_020023255.1
GCA_020023425.1
GCA_020023445.1
GCA_020023465.1
GCA_020023735.2
GCA_020023755.1
GCA_020023775.1
GCA_020023795.1
GCA_020024955.1
GCA_020025155.1
GCA_020026305.1
GCA_020034535.1
GCA_020034555.1
GCA_020034575.1
GCA_020034595.1
GCA_020034615.1
GCA_020034635.1
GCA_020035395.2
GCA_020035495.1
GCA_020035515.1
GCA_020035535.1
GCA_020041945.1
GCA_020042005.1
GCA_020042125.1
GCA_020042225.1
GCA_020042285.1
GCA_020042345.1
GCA_020042445.1
GCA_020042525.1

GCA_020042585.1
GCA_020042605.1
GCA_020042625.1
GCA_020042665.1
GCA_020042685.1
GCA_020042705.1
GCA_020042725.1
GCA_020043425.2
GCA_020047135.1
GCA_020047155.1
GCA_020079945.1
GCA_020080045.1
GCA_020080085.1
GCA_020080125.1
GCA_020080145.1
GCA_020084865.1
GCA_020084885.1
GCA_020084945.1
GCA_020091305.1
GCA_020091325.1
GCA_020091345.1
GCA_020091365.1
GCA_020091385.1
GCA_020091405.1
GCA_020091425.1
GCA_020091445.1
GCA_020091465.1
GCA_020091485.1
GCA_020091505.1
GCA_020091525.1
GCA_020091545.1
GCA_020091565.1
GCA_020091585.1
GCA_020091645.1
GCA_020091665.1
GCA_020096995.1
GCA_020097015.1
GCA_020097035.1
GCA_020097055.1
GCA_020097075.1
GCA_020097095.1
GCA_020097115.1
GCA_020097135.1
GCA_020097155.1
GCA_020097175.1
GCA_020097195.1
GCA_020097215.1
GCA_020097235.1
GCA_020097255.1
GCA_020097275.1
GCA_020097295.1
GCA_020097315.1
GCA_020097335.1
GCA_020097355.1

GCA_020097375.1
GCA_020097395.1
GCA_020097415.1
GCA_020097435.1
GCA_020097455.1
GCA_020097475.1
GCA_020097495.1
GCA_020097615.1
GCA_020097655.1
GCA_020097755.1
GCA_020099175.1
GCA_020099215.1
GCA_020099235.1
GCA_020099255.1
GCA_020099275.1
GCA_020099295.1
GCA_020099315.1
GCA_020099335.1
GCA_020099355.1
GCA_020099375.1
GCA_020099395.1
GCA_020105775.1
GCA_020132085.1
GCA_020132105.1
GCA_020138335.1
GCA_020138355.1
GCA_020138375.1
GCA_020138395.1
GCA_020138415.1
GCA_020138435.1
GCA_020138455.1
GCA_020138475.1
GCA_020138495.1
GCA_020138515.1
GCA_020138535.1
GCA_020138555.1
GCA_020138575.1
GCA_020138595.1
GCA_020138615.1
GCA_020138635.1
GCA_020138655.1
GCA_020138675.1
GCA_020138695.1
GCA_020138715.1
GCA_020138735.1
GCA_020138755.1
GCA_020138775.1
GCA_020138795.1
GCA_020139215.1
GCA_020139235.1
GCA_020139255.1
GCA_020139275.1
GCA_020139295.1
GCA_020143085.2

GCA_020149465.1
GCA_020149505.1
GCA_020149525.1
GCA_020149545.1
GCA_020149575.1
GCA_020149605.1
GCA_020149625.1
GCA_020149645.1
GCA_020149665.1
GCA_020149685.1
GCA_020149705.1
GCA_020149765.1
GCA_020149785.1
GCA_020149805.1
GCA_020149825.1
GCA_020149875.1
GCA_020149895.1
GCA_020149915.1
GCA_020149935.1
GCA_020149955.1
GCA_020149975.1
GCA_020149995.1
GCA_020150015.1
GCA_020150035.1
GCA_020150295.1
GCA_020150375.1
GCA_020150455.1
GCA_020150655.1
GCA_020150755.1
GCA_020150915.1
GCA_020151015.1
GCA_020162095.1
GCA_020162115.1
GCA_020162135.1
GCA_020162155.1
GCA_020162175.1
GCA_020162195.1
GCA_020162215.1
GCA_020162235.1
GCA_020162255.1
GCA_020162275.1
GCA_020162295.1
GCA_020172745.1
GCA_020172765.2
GCA_020177155.3
GCA_020181355.1
GCA_020181375.1
GCA_020181395.1
GCA_020181415.1
GCA_020181435.1
GCA_020181455.1
GCA_020181475.1
GCA_020181495.1
GCA_020181515.1

GCA_020181535.1
GCA_020181555.1
GCA_020181575.1
GCA_020181655.1
GCA_020181675.1
GCA_020181695.1
GCA_020190405.1
GCA_020204845.1
GCA_020215625.1
GCA_020215645.1
GCA_020215665.1
GCA_020217385.1
GCA_020217405.1
GCA_020217445.1
GCA_020217465.1
GCA_020221715.1
GCA_020221755.1
GCA_020221775.1
GCA_020221795.1
GCA_020229655.1
GCA_020229695.1
GCA_020229715.1
GCA_020229735.1
GCA_020268605.1
GCA_020268625.1
GCA_020268645.1
GCA_020271605.1
GCA_020271725.1
GCA_020271745.1
GCA_020271765.1
GCA_020276565.1
GCA_020276585.1
GCA_020276605.1
GCA_020276625.1
GCA_020276645.1
GCA_020309925.1
GCA_020309945.1
GCA_020309965.1
GCA_020309985.1
GCA_020310005.1
GCA_020310025.1
GCA_020310045.1
GCA_020311615.1
GCA_020311635.1
GCA_020341595.1
GCA_020341615.1
GCA_020341635.1
GCA_020341655.1
GCA_020342335.1
GCA_020342415.1
GCA_020342455.1
GCA_020353915.1
GCA_020386595.1
GCA_020386615.1

GCA_020386635.1
GCA_020388055.1
GCA_020388075.1
GCA_020388095.1
GCA_020388115.1
GCA_020388135.1
GCA_020388155.1
GCA_020388175.1
GCA_020388195.1
GCA_020388215.1
GCA_020388235.1
GCA_020388255.1
GCA_020388275.1
GCA_020388295.1
GCA_020388315.1
GCA_020388335.1
GCA_020388355.1
GCA_020388395.1
GCA_020388555.1
GCA_020388655.1
GCA_020388675.1
GCA_020388695.1
GCA_020388715.1
GCA_020388735.1
GCA_020388765.1
GCA_020388785.1
GCA_020388805.1
GCA_020388825.1
GCA_020388845.1
GCA_020388885.1
GCA_020388905.1
GCA_020388925.1
GCA_020388965.1
GCA_020388985.1
GCA_020389005.1
GCA_020389025.2
GCA_020389165.1
GCA_020389265.1
GCA_020389325.1
GCA_020389425.1
GCA_020389445.1
GCA_020389485.1
GCA_020389505.1
GCA_020389525.1
GCA_020389545.1
GCA_020389565.1
GCA_020401845.1
GCA_020405145.1
GCA_020405165.1
GCA_020405265.1
GCA_020405285.1
GCA_020405305.1
GCA_020405325.1
GCA_020405345.1

GCA_020405365.1
GCA_020405385.1
GCA_020410665.1
GCA_020410745.1
GCA_020410765.1
GCA_020410785.1
GCA_020410805.1
GCA_020410865.1
GCA_020410885.1
GCA_020412405.1
GCA_020412425.1
GCA_020412445.1
GCA_020412465.1
GCA_020412485.1
GCA_020422885.1
GCA_020422905.1
GCA_020422925.2
GCA_020422945.1
GCA_020422965.1
GCA_020422985.1
GCA_020423005.1
GCA_020423025.1
GCA_020423045.1
GCA_020423065.1
GCA_020423085.1
GCA_020423105.1
GCA_020423125.1
GCA_020423145.1
GCA_020423165.1
GCA_020423185.1
GCA_020423205.1
GCA_020423225.1
GCA_020424045.1
GCA_020424085.1
GCA_020424105.1
GCA_020424345.1
GCA_020424365.1
GCA_020428325.2
GCA_020447105.2
GCA_020447175.2
GCA_020447305.2
GCA_020450285.1
GCA_020459125.1
GCA_020459305.1
GCA_020462325.1
GCA_020463755.1
GCA_020463795.1
GCA_020494055.1
GCA_020494085.1
GCA_020497585.1
GCA_020497635.1
GCA_020510245.1
GCA_020510525.1
GCA_020510545.1

GCA_020510565.1
GCA_020510585.1
GCA_020510605.1
GCA_020510625.1
GCA_020510645.1
GCA_020510665.1
GCA_020510685.1
GCA_020510705.1
GCA_020511155.1
GCA_020519665.1
GCA_020520145.1
GCA_020520165.1
GCA_020520185.1
GCA_020520205.1
GCA_020520245.1
GCA_020520265.1
GCA_020520285.1
GCA_020520305.1
GCA_020520325.1
GCA_020520345.1
GCA_020520365.1
GCA_020520385.1
GCA_020520405.1
GCA_020525465.1
GCA_020525485.1
GCA_020525505.1
GCA_020525525.1
GCA_020525545.1
GCA_020525565.1
GCA_020525585.1
GCA_020525605.1
GCA_020525625.1
GCA_020525645.1
GCA_020525705.1
GCA_020525785.1
GCA_020525805.1
GCA_020525825.1
GCA_020525845.1
GCA_020525865.1
GCA_020525885.1
GCA_020525905.1
GCA_020525925.1
GCA_020525945.1
GCA_020525965.1
GCA_020525985.1
GCA_020526005.1
GCA_020526025.1
GCA_020526045.1
GCA_020526065.1
GCA_020526085.1
GCA_020526105.1
GCA_020526725.1
GCA_020526745.1
GCA_020526765.1

GCA_020526785.1
GCA_020526805.1
GCA_020526825.1
GCA_020526845.1
GCA_020535145.1
GCA_020535165.1
GCA_020535185.1
GCA_020535205.1
GCA_020535225.1
GCA_020535245.1
GCA_020535385.1
GCA_020535405.1
GCA_020535425.1
GCA_020535465.1
GCA_020535545.1
GCA_020535565.1
GCA_020540925.1
GCA_020540945.1
GCA_020540965.1
GCA_020540985.1
GCA_020541005.1
GCA_020541025.1
GCA_020541125.1
GCA_020541225.1
GCA_020541245.1
GCA_020541265.1
GCA_020541285.1
GCA_020541305.1
GCA_020541325.1
GCA_020541345.1
GCA_020541825.1
GCA_020541865.1
GCA_020541885.1
GCA_020541905.1
GCA_020541925.1
GCA_020541945.1
GCA_020541965.1
GCA_020542005.1
GCA_020542025.1
GCA_020542045.1
GCA_020542785.1
GCA_020544305.1
GCA_020544345.1
GCA_020544365.1
GCA_020544385.1
GCA_020544405.1
GCA_020544425.1
GCA_020544445.1
GCA_020544465.1
GCA_020544545.1
GCA_020546665.1
GCA_020546685.1
GCA_020546705.1
GCA_020546785.1

GCA_020546805.1
GCA_020547025.1
GCA_020547085.1
GCA_020549965.1
GCA_020550085.1
GCA_020595105.1
GCA_020614015.1
GCA_020614055.1
GCA_020614175.1
GCA_020614295.1
GCA_020614335.1
GCA_020614355.1
GCA_020616155.1
GCA_020616175.1
GCA_020616195.1
GCA_020616235.1
GCA_020616335.1
GCA_020616475.1
GCA_020616495.1
GCA_020616515.1
GCA_020616535.1
GCA_020616555.1
GCA_020616575.1
GCA_020616595.1
GCA_020616615.1
GCA_020616635.1
GCA_020616775.1
GCA_020616875.1
GCA_020616935.1
GCA_020618415.1
GCA_020623585.1
GCA_020628355.1
GCA_020628455.1
GCA_020628555.1
GCA_020628665.1
GCA_020628735.1
GCA_020637575.2
GCA_020640955.1
GCA_020640975.1
GCA_020640995.1
GCA_020641015.1
GCA_020641075.2
GCA_020641085.2
GCA_020641095.2
GCA_020641355.2
GCA_020641365.2
GCA_020641395.2
GCA_020641415.2
GCA_020641425.2
GCA_020641455.2
GCA_020673335.1
GCA_020683005.1
GCA_020683025.1
GCA_020683045.1

GCA_020683065.1
GCA_020683085.1
GCA_020683105.1
GCA_020683125.1
GCA_020683145.1
GCA_020683185.1
GCA_020683205.1
GCA_020683245.1
GCA_020683265.1
GCA_020684805.1
GCA_020684825.1
GCA_020691705.1
GCA_020691725.1
GCA_020691745.1
GCA_020694005.1
GCA_020695365.1
GCA_020695485.1
GCA_020695545.1
GCA_020695585.1
GCA_020695605.1
GCA_020695665.1
GCA_020695685.1
GCA_020695705.1
GCA_020695725.1
GCA_020702135.2
GCA_020702515.2
GCA_020702535.2
GCA_020702545.2
GCA_020702575.2
GCA_020702585.2
GCA_020702615.2
GCA_020714625.1
GCA_020714645.1
GCA_020714665.1
GCA_020714685.1
GCA_020714705.1
GCA_020714725.1
GCA_020714745.1
GCA_020714765.1
GCA_020714785.1
GCA_020714805.1
GCA_020714825.1
GCA_020714845.1
GCA_020714865.1
GCA_020714885.1
GCA_020714905.1
GCA_020714925.1
GCA_020715045.1
GCA_020715165.1
GCA_020715225.1
GCA_020715265.1
GCA_020715285.1
GCA_020715305.1
GCA_020715325.1

GCA_020715345.1
GCA_020715365.1
GCA_020715385.1
GCA_020715405.1
GCA_020715425.1
GCA_020715445.1
GCA_020715465.1
GCA_020715485.1
GCA_020715505.1
GCA_020715525.1
GCA_020715545.1
GCA_020715565.1
GCA_020715685.1
GCA_020716645.1
GCA_020716665.1
GCA_020717785.1
GCA_020718705.1
GCA_020718885.1
GCA_020719015.1
GCA_020719145.1
GCA_020719285.1
GCA_020719435.1
GCA_020719565.1
GCA_020719705.1
GCA_020719855.1
GCA_020720025.1
GCA_020720295.1
GCA_020720445.1
GCA_020720575.1
GCA_020730965.2
GCA_020731415.2
GCA_020735285.1
GCA_020735345.1
GCA_020735365.1
GCA_020735385.1
GCA_020735405.1
GCA_020735425.1
GCA_020735445.1
GCA_020735465.1
GCA_020735485.1
GCA_020735505.1
GCA_020735525.1
GCA_020735545.1
GCA_020735565.1
GCA_020735585.1
GCA_020735605.1
GCA_020735625.1
GCA_020735645.1
GCA_020735705.1
GCA_020735725.1
GCA_020735745.1
GCA_020735765.1
GCA_020735785.1
GCA_020735805.1

GCA_020735825.1
GCA_020735845.1
GCA_020735865.1
GCA_020735885.1
GCA_020735905.1
GCA_020735925.1
GCA_020735945.1
GCA_020735965.1
GCA_020735985.1
GCA_020736005.1
GCA_020736025.1
GCA_020736045.1
GCA_020736065.1
GCA_020736085.1
GCA_020736105.1
GCA_020736125.1
GCA_020736145.1
GCA_020736165.1
GCA_020736185.1
GCA_020736205.1
GCA_020736225.1
GCA_020736245.1
GCA_020736265.1
GCA_020736285.1
GCA_020736305.1
GCA_020736325.1
GCA_020736345.1
GCA_020736465.1
GCA_020736585.1
GCA_020736625.1
GCA_020740535.1
GCA_020771675.1
GCA_020771755.1
GCA_020773035.1
GCA_020783215.1
GCA_020783235.1
GCA_020783255.1
GCA_020783275.1
GCA_020783295.1
GCA_020783315.1
GCA_020783335.1
GCA_020783355.1
GCA_020783375.1
GCA_020783435.1
GCA_020783455.1
GCA_020783475.1
GCA_020783495.1
GCA_020783515.1
GCA_020783535.1
GCA_020783555.1
GCA_020783575.1
GCA_020783595.1
GCA_020783615.1
GCA_020783635.1

GCA_020790095.1
GCA_020790115.1
GCA_020790135.1
GCA_020790175.1
GCA_020790195.1
GCA_020792675.1
GCA_020808945.1
GCA_020808965.1
GCA_020808985.1
GCA_020809005.1
GCA_020809045.1
GCA_020809065.1
GCA_020809085.1
GCA_020809105.1
GCA_020809125.1
GCA_020809145.1
GCA_020809165.1
GCA_020809185.1
GCA_020809205.1
GCA_020809245.1
GCA_020809365.1
GCA_020809385.1
GCA_020809405.1
GCA_020809485.1
GCA_020810595.1
GCA_020810655.1
GCA_020810675.1
GCA_020826335.1
GCA_020826915.1
GCA_020826935.1
GCA_020826955.1
GCA_020826975.1
GCA_020826995.1
GCA_020827015.1
GCA_020827035.1
GCA_020827055.1
GCA_020827075.1
GCA_020827095.1
GCA_020827115.1
GCA_020827135.1
GCA_020827275.1
GCA_020827555.1
GCA_020827575.1
GCA_020827595.1
GCA_020827615.1
GCA_020827635.1
GCA_020827655.1
GCA_020827895.1
GCA_020843905.1
GCA_020843925.1
GCA_020843945.1
GCA_020843965.1
GCA_020843985.1
GCA_020844005.1

GCA_020844025.1
GCA_020844125.1
GCA_020861345.1
GCA_020865525.1
GCA_020865565.1
GCA_020865585.1
GCA_020866785.1
GCA_020866845.1
GCA_020866865.1
GCA_020866885.1
GCA_020868245.1
GCA_020871915.1
GCA_020872015.1
GCA_020881835.1
GCA_020881875.1
GCA_020881895.1
GCA_020881935.1
GCA_020881955.1
GCA_020881975.1
GCA_020882015.1
GCA_020882035.1
GCA_020882055.1
GCA_020882075.1
GCA_020882095.1
GCA_020882115.1
GCA_020882135.1
GCA_020882195.1
GCA_020882585.1
GCA_020882605.1
GCA_020882625.1
GCA_020882645.1
GCA_020882925.2
GCA_020883195.1
GCA_020883215.1
GCA_020883235.1
GCA_020883255.1
GCA_020883275.1
GCA_020883295.1
GCA_020883315.1
GCA_020883335.1
GCA_020883355.1
GCA_020883375.1
GCA_020883395.1
GCA_020883415.1
GCA_020883435.1
GCA_020883455.1
GCA_020883475.1
GCA_020883495.1
GCA_020883515.1
GCA_020883535.1
GCA_020885515.1
GCA_020885575.1
GCA_020885655.1
GCA_020885715.1

GCA_020885775.1
GCA_020885855.1
GCA_020885975.1
GCA_020886055.1
GCA_020886115.1
GCA_020886175.1
GCA_020886235.1
GCA_020886375.2
GCA_020886435.2
GCA_020886495.2
GCA_020886575.2
GCA_020886635.1
GCA_020886695.1
GCA_020886775.1
GCA_020887015.1
GCA_020887095.1
GCA_020887155.1
GCA_020887215.1
GCA_020889625.1
GCA_020889645.1
GCA_020889665.1
GCA_020892075.1
GCA_020892095.1
GCA_020892115.1
GCA_020892835.1
GCA_020904955.2
GCA_020904975.2
GCA_020904985.2
GCA_020904995.2
GCA_020905035.2
GCA_020905095.2
GCA_020905105.2
GCA_020905635.1
GCA_020905655.1
GCA_020905675.1
GCA_020905695.1
GCA_020905715.1
GCA_020905735.1
GCA_020905755.1
GCA_020905775.1
GCA_020905795.1
GCA_020905815.1
GCA_020905835.1
GCA_020905855.1
GCA_020905875.1
GCA_020905895.1
GCA_020905915.1
GCA_020905935.1
GCA_020905955.1
GCA_020905975.1
GCA_020905995.1
GCA_020906015.1
GCA_020906035.1
GCA_020906055.1

GCA_020906075.1
GCA_020906095.1
GCA_020906115.1
GCA_020906135.1
GCA_020906155.1
GCA_020906175.1
GCA_020906195.1
GCA_020906215.1
GCA_020906235.1
GCA_020906255.1
GCA_020906275.1
GCA_020906295.1
GCA_020906315.1
GCA_020906335.1
GCA_020906355.1
GCA_020906375.1
GCA_020906395.1
GCA_020906415.1
GCA_020906435.1
GCA_020906455.1
GCA_020906475.1
GCA_020906495.1
GCA_020906545.1
GCA_020906565.1
GCA_020906585.1
GCA_020906605.1
GCA_020906765.2
GCA_020906775.2
GCA_020906835.2
GCA_020906975.2
GCA_020911725.1
GCA_020911745.1
GCA_020911785.1
GCA_020911805.1
GCA_020911825.1
GCA_020911845.1
GCA_020911865.1
GCA_020911885.1
GCA_020911905.1
GCA_020911925.1
GCA_020911945.1
GCA_020911965.1
GCA_020911985.1
GCA_020912005.1
GCA_020917325.1
GCA_020923435.1
GCA_020923475.1
GCA_020923495.1
GCA_020971565.1
GCA_020971705.1
GCA_020971725.1
GCA_020971745.1
GCA_020971765.1
GCA_020971785.1

GCA_020974905.1
GCA_020978345.1
GCA_020985225.1
GCA_020985245.1
GCA_020985265.1
GCA_020985285.1
GCA_020985305.1
GCA_020985325.1
GCA_020985345.1
GCA_020985365.1
GCA_020990385.1
GCA_020990405.1
GCA_020990425.1
GCA_020990445.1
GCA_020990465.1
GCA_020990485.1
GCA_020990505.1
GCA_020990525.1
GCA_020990545.1
GCA_020990565.1
GCA_020990585.1
GCA_020990605.1
GCA_020990625.1
GCA_020990645.1
GCA_020990665.1
GCA_020990685.1
GCA_020990705.1
GCA_020990725.1
GCA_020990745.1
GCA_020990765.1
GCA_020990785.1
GCA_020990805.1
GCA_020991025.1
GCA_020991045.1
GCA_020991065.1
GCA_020991085.1
GCA_020991105.1
GCA_020991125.1
GCA_020991145.1
GCA_020991185.1
GCA_020991205.1
GCA_020991225.1
GCA_020995365.1
GCA_020995385.1
GCA_020995405.1
GCA_020995455.1
GCA_020995475.1
GCA_021018745.1
GCA_021018765.1
GCA_021018785.1
GCA_021028635.1
GCA_021029445.1
GCA_021029465.1
GCA_021029665.1

GCA_021029685.1
GCA_021029705.2
GCA_021029855.2
GCA_021044545.1
GCA_021044565.1
GCA_021044585.1
GCA_021044625.1
GCA_021044645.1
GCA_021044665.1
GCA_021044685.1
GCA_021044705.1
GCA_021049225.1
GCA_021049245.1
GCA_021049265.1
GCA_021049285.1
GCA_021049305.1
GCA_021049325.1
GCA_021052245.1
GCA_021052265.1
GCA_021052285.1
GCA_021052305.1
GCA_021052325.1
GCA_021052345.1
GCA_021052365.1
GCA_021052425.1
GCA_021052565.1
GCA_021052665.1
GCA_021052705.1
GCA_021054785.1
GCA_021057185.1
GCA_021057265.1
GCA_021065005.1
GCA_021065045.1
GCA_021065065.1
GCA_021088345.1
GCA_021089875.1
GCA_021091115.1
GCA_021117075.1
GCA_021117155.1
GCA_021117175.1
GCA_021117195.1
GCA_021117215.1
GCA_021117235.1
GCA_021117255.1
GCA_021117275.1
GCA_021117295.1
GCA_021117345.1
GCA_021117365.1
GCA_021117385.1
GCA_021117405.1
GCA_021117425.1
GCA_021135615.1
GCA_021137615.1
GCA_021137635.1

GCA_021137655.1
GCA_021137675.1
GCA_021137695.1
GCA_021137715.1
GCA_021137735.1
GCA_021137755.1
GCA_021137775.1
GCA_021137795.1
GCA_021137815.1
GCA_021137835.1
GCA_021137855.1
GCA_021137875.1
GCA_021137895.1
GCA_021137915.1
GCA_021137935.1
GCA_021137955.1
GCA_021137975.1
GCA_021138035.1
GCA_021138155.1
GCA_021165735.1
GCA_021165755.1
GCA_021165775.1
GCA_021165815.1
GCA_021165835.1
GCA_021165855.1
GCA_021165875.1
GCA_021165915.1
GCA_021165935.1
GCA_021165955.1
GCA_021165975.1
GCA_021165995.1
GCA_021166015.1
GCA_021166035.1
GCA_021166055.1
GCA_021166075.1
GCA_021166095.1
GCA_021166115.1
GCA_021166135.1
GCA_021166155.1
GCA_021166175.1
GCA_021166195.1
GCA_021166215.1
GCA_021166235.1
GCA_021166255.2
GCA_021166275.2
GCA_021166295.2
GCA_021166315.2
GCA_021166335.2
GCA_021166355.2
GCA_021166375.2
GCA_021166395.1
GCA_021166415.1
GCA_021166435.1
GCA_021172025.1

GCA_021172045.1
GCA_021172065.1
GCA_021172085.1
GCA_021172105.3
GCA_021172125.1
GCA_021172145.1
GCA_021172165.1
GCA_021172185.1
GCA_021172805.1
GCA_021172825.1
GCA_021172845.1
GCA_021172865.1
GCA_021172885.1
GCA_021172905.1
GCA_021172925.1
GCA_021172945.1
GCA_021173085.1
GCA_021183565.1
GCA_021183585.1
GCA_021183605.1
GCA_021183625.1
GCA_021183645.1
GCA_021183665.1
GCA_021183685.1
GCA_021183705.1
GCA_021183725.1
GCA_021183745.1
GCA_021183765.1
GCA_021183785.1
GCA_021183805.1
GCA_021183825.1
GCA_021184005.1
GCA_021184025.1
GCA_021184065.1
GCA_021184105.1
GCA_021184125.1
GCA_021184145.1
GCA_021184165.1
GCA_021184205.1
GCA_021184225.1
GCA_021184245.1
GCA_021184265.1
GCA_021193625.2
GCA_021216655.1
GCA_021216675.1
GCA_021222645.1
GCA_021223605.1
GCA_021227995.1
GCA_021228015.1
GCA_021228035.1
GCA_021228055.1
GCA_021228615.1
GCA_021228635.1
GCA_021228675.1

GCA_021228695.1
GCA_021228735.1
GCA_021228755.1
GCA_021228795.1
GCA_021228815.1
GCA_021228835.1
GCA_021228855.1
GCA_021228875.1
GCA_021228895.1
GCA_021228915.1
GCA_021228935.1
GCA_021228955.1
GCA_021228975.1
GCA_021228995.1
GCA_021233055.1
GCA_021233075.1
GCA_021233135.1
GCA_021233235.1
GCA_021233395.1
GCA_021233455.1
GCA_021249245.1
GCA_021249265.1
GCA_021249285.1
GCA_021249305.1
GCA_021249325.1
GCA_021249365.1
GCA_021249385.1
GCA_021251405.1
GCA_021253825.1
GCA_021266225.1
GCA_021266245.1
GCA_021266265.1
GCA_021266285.1
GCA_021266305.1
GCA_021266325.1
GCA_021266345.1
GCA_021266365.1
GCA_021266405.1
GCA_021266425.1
GCA_021266445.1
GCA_021266465.1
GCA_021266585.1
GCA_021266605.1
GCA_021276285.1
GCA_021278925.1
GCA_021278945.1
GCA_021278965.1
GCA_021278985.1
GCA_021279005.2
GCA_021282525.2
GCA_021282625.2
GCA_021282645.2
GCA_021283055.2
GCA_021283075.2

GCA_021283125.2
GCA_021283165.2
GCA_021283365.2
GCA_021283375.2
GCA_021283465.2
GCA_021283545.2
GCA_021284745.1
GCA_021284765.1
GCA_021290385.1
GCA_021294815.2
GCA_021310975.1
GCA_021310995.1
GCA_021329025.1
GCA_021347605.1
GCA_021353295.2
GCA_021359565.1
GCA_021359585.1
GCA_021365465.1
GCA_021365485.1
GCA_021365505.1
GCA_021365535.1
GCA_021365555.1
GCA_021378215.1
GCA_021378295.1
GCA_021378315.1
GCA_021378335.1
GCA_021378355.1
GCA_021378375.1
GCA_021378395.1
GCA_021378415.1
GCA_021378605.1
GCA_021378625.1
GCA_021378645.1
GCA_021378665.1
GCA_021378685.1
GCA_021378705.1
GCA_021378725.1
GCA_021379005.1
GCA_021383585.1
GCA_021383745.1
GCA_021383865.1
GCA_021383925.1
GCA_021389915.1
GCA_021389935.1
GCA_021389955.1
GCA_021389975.1
GCA_021389995.1
GCA_021390015.1
GCA_021390035.1
GCA_021390055.1
GCA_021390075.1
GCA_021390095.1
GCA_021390115.1
GCA_021390135.1

GCA_021390155.1
GCA_021390175.1
GCA_021390195.1
GCA_021390295.2
GCA_021391295.1
GCA_021391315.1
GCA_021391335.1
GCA_021391355.1
GCA_021391375.1
GCA_021391395.1
GCA_021391415.1
GCA_021391435.1
GCA_021391455.1
GCA_021391475.1
GCA_021391495.1
GCA_021391515.1
GCA_021391535.1
GCA_021391555.1
GCA_021391575.1
GCA_021391595.1
GCA_021391615.1
GCA_021391635.1
GCA_021391655.1
GCA_021391675.1
GCA_021391695.1
GCA_021391715.1
GCA_021397585.1
GCA_021397605.1
GCA_021397625.1
GCA_021397645.1
GCA_021397675.1
GCA_021397695.1
GCA_021397715.1
GCA_021397735.1
GCA_021397755.1
GCA_021397795.1
GCA_021398115.1
GCA_021398135.1
GCA_021398155.1
GCA_021398215.1
GCA_021398235.1
GCA_021398265.1
GCA_021398285.1
GCA_021398305.1
GCA_021398325.1
GCA_021398345.1
GCA_021398365.1
GCA_021398395.1
GCA_021398445.1
GCA_021398465.1
GCA_021398485.1
GCA_021398505.1
GCA_021398545.1
GCA_021398615.1

GCA_021398655.1
GCA_021398715.1
GCA_021398735.1
GCA_021398755.1
GCA_021398835.1
GCA_021398855.1
GCA_021398895.1
GCA_021398915.1
GCA_021398935.1
GCA_021398955.1
GCA_021398975.1
GCA_021398995.1
GCA_021399015.1
GCA_021399035.1
GCA_021399055.1
GCA_021399075.1
GCA_021399095.1
GCA_021399115.1
GCA_021399135.1
GCA_021399155.1
GCA_021399175.1
GCA_021399195.1
GCA_021399215.1
GCA_021399235.1
GCA_021399255.1
GCA_021401425.1
GCA_021431865.1
GCA_021431905.1
GCA_021431945.1
GCA_021431965.1
GCA_021431985.1
GCA_021432005.1
GCA_021432025.1
GCA_021432045.1
GCA_021432065.1
GCA_021432085.1
GCA_021432105.1
GCA_021432125.1
GCA_021432145.1
GCA_021432165.1
GCA_021432185.1
GCA_021432745.1
GCA_021432765.1
GCA_021441825.1
GCA_021441845.1
GCA_021441865.1
GCA_021441885.1
GCA_021441905.1
GCA_021441925.1
GCA_021441945.1
GCA_021441965.1
GCA_021441985.1
GCA_021442005.1
GCA_021442025.1

GCA_021442045.1
GCA_021442065.1
GCA_021442085.1
GCA_021442105.1
GCA_021442325.1
GCA_021459805.1
GCA_021459825.1
GCA_021459845.1
GCA_021459865.1
GCA_021459885.1
GCA_021459905.1
GCA_021459925.1
GCA_021459945.1
GCA_021459965.1
GCA_021459985.1
GCA_021460035.1
GCA_021460055.1
GCA_021460095.1
GCA_021460115.1
GCA_021460135.1
GCA_021460155.1
GCA_021460175.1
GCA_021460195.1
GCA_021460215.1
GCA_021462285.1
GCA_021464465.1
GCA_021474145.1
GCA_021474165.1
GCA_021474185.1
GCA_021474205.1
GCA_021474225.1
GCA_021474245.1
GCA_021474265.1
GCA_021474285.1
GCA_021474305.1
GCA_021474325.1
GCA_021474345.1
GCA_021484745.1
GCA_021484765.1
GCA_021484785.1
GCA_021484805.1
GCA_021484825.1
GCA_021484845.1
GCA_021484865.1
GCA_021484885.1
GCA_021484905.1
GCA_021484925.1
GCA_021484945.1
GCA_021484965.1
GCA_021484985.1
GCA_021485005.1
GCA_021485025.1
GCA_021491615.1
GCA_021491635.1

GCA_021491675.1
GCA_021491715.1
GCA_021491735.1
GCA_021491775.1
GCA_021491835.1
GCA_021491895.1
GCA_021491915.1
GCA_021491935.1
GCA_021491955.1
GCA_021495345.2
GCA_021495975.1
GCA_021495995.1
GCA_021496015.1
GCA_021496035.1
GCA_021496075.1
GCA_021496095.1
GCA_021496115.1
GCA_021496135.1
GCA_021496155.1
GCA_021496175.1
GCA_021496195.1
GCA_021496215.1
GCA_021496235.1
GCA_021496255.1
GCA_021496275.1
GCA_021496295.1
GCA_021496325.1
GCA_021496345.1
GCA_021496365.1
GCA_021496385.1
GCA_021496405.1
GCA_021496425.1
GCA_021496445.1
GCA_021496465.1
GCA_021496485.1
GCA_021496505.1
GCA_021496525.1
GCA_021496545.1
GCA_021496665.1
GCA_021496725.1
GCA_021496745.1
GCA_021496765.1
GCA_021496785.1
GCA_021496805.1
GCA_021496825.1
GCA_021496845.1
GCA_021496865.1
GCA_021496885.1
GCA_021496905.1
GCA_021496945.1
GCA_021496965.1
GCA_021496985.1
GCA_021497025.1
GCA_021497045.1

GCA_021497065.1
GCA_021497085.1
GCA_021497105.1
GCA_021497125.1
GCA_021497145.1
GCA_021497165.1
GCA_021497185.1
GCA_021497225.1
GCA_021497265.1
GCA_021497285.1
GCA_021497305.1
GCA_021497325.1
GCA_021497345.1
GCA_021497365.1
GCA_021497385.1
GCA_021497405.1
GCA_021497425.1
GCA_021497445.1
GCA_021497465.1
GCA_021497485.1
GCA_021497505.1
GCA_021497525.1
GCA_021497545.1
GCA_021497605.1
GCA_021497625.1
GCA_021497645.1
GCA_021497885.1
GCA_021497945.1
GCA_021497965.1
GCA_021497985.1
GCA_021498005.1
GCA_021498025.2
GCA_021498045.1
GCA_021498145.1
GCA_021498165.1
GCA_021498225.1
GCA_021498245.1
GCA_021498265.1
GCA_021498285.1
GCA_021498305.1
GCA_021513015.1
GCA_021513035.1
GCA_021513055.1
GCA_021513075.1
GCA_021513095.1
GCA_021513115.1
GCA_021513155.1
GCA_021513175.1
GCA_021513195.1
GCA_021513215.1
GCA_021513235.1
GCA_021513255.1
GCA_021513275.1
GCA_021513295.1

GCA_021513315.1
GCA_021513335.1
GCA_021513355.1
GCA_021513375.1
GCA_021513395.1
GCA_021513415.1
GCA_021513435.1
GCA_021513455.1
GCA_021513475.1
GCA_021513495.1
GCA_021513515.1
GCA_021513535.1
GCA_021513555.1
GCA_021513575.1
GCA_021513595.1
GCA_021513615.1
GCA_021513635.1
GCA_021513675.1
GCA_021534845.1
GCA_021534885.1
GCA_021534905.1
GCA_021534925.1
GCA_021534945.1
GCA_021534965.1
GCA_021534985.1
GCA_021535005.1
GCA_021535025.1
GCA_021535045.1
GCA_021535065.1
GCA_021535085.1
GCA_021535105.1
GCA_021535135.1
GCA_021535155.1
GCA_021537535.1
GCA_021545335.1
GCA_021545785.1
GCA_021545825.1
GCA_021545845.1
GCA_021545865.1
GCA_021554655.1
GCA_021559635.1
GCA_021559655.1
GCA_021559675.1
GCA_021559695.1
GCA_021559715.1
GCA_021559735.1
GCA_021559815.1
GCA_021559835.1
GCA_021559855.1
GCA_021559875.1
GCA_021559895.1
GCA_021559915.1
GCA_021559955.1
GCA_021560015.1

GCA_021560035.1
GCA_021560055.1
GCA_021560075.1
GCA_021560095.1
GCA_021560115.1
GCA_021560135.1
GCA_021560175.1
GCA_021560195.1
GCA_021560215.1
GCA_021560235.1
GCA_021560255.1
GCA_021560275.1
GCA_021560295.1
GCA_021560315.1
GCA_021560335.1
GCA_021560385.1
GCA_021560405.1
GCA_021560425.1
GCA_021560445.1
GCA_021560465.1
GCA_021560485.1
GCA_021560505.1
GCA_021560525.1
GCA_021560555.1
GCA_021560575.1
GCA_021560595.1
GCA_021560615.1
GCA_021560635.1
GCA_021560655.1
GCA_021560675.1
GCA_021560695.1
GCA_021562195.1
GCA_021582295.1
GCA_021582875.1
GCA_021582895.2
GCA_021586915.1
GCA_021590045.1
GCA_021595545.1
GCA_021596945.1
GCA_021609905.1
GCA_021609965.1
GCA_021609985.1
GCA_021610005.1
GCA_021610025.1
GCA_021610105.1
GCA_021611515.1
GCA_021614775.1
GCA_021614795.1
GCA_021631985.1
GCA_021650705.1
GCA_021650815.1
GCA_021650835.1
GCA_021650855.1
GCA_021650875.1

GCA_021650895.1
GCA_021650915.1
GCA_021650935.1
GCA_021650955.1
GCA_021650975.2
GCA_021651035.1
GCA_021654315.1
GCA_021654335.1
GCA_021654355.1
GCA_021654375.1
GCA_021654455.1
GCA_021654515.1
GCA_021654535.1
GCA_021654555.1
GCA_021654575.1
GCA_021654595.1
GCA_021654615.1
GCA_021654635.1
GCA_021654655.1
GCA_021654675.1
GCA_021654695.1
GCA_021654715.1
GCA_021654735.1
GCA_021654755.1
GCA_021654775.1
GCA_021654795.1
GCA_021654855.1
GCA_021654875.1
GCA_021654895.1
GCA_021654915.1
GCA_021654935.1
GCA_021654975.1
GCA_021654995.1
GCA_021655015.1
GCA_021655035.1
GCA_021655055.1
GCA_021655135.1
GCA_021655155.1
GCA_021655175.1
GCA_021655195.1
GCA_021655215.1
GCA_021655235.1
GCA_021655255.1
GCA_021655275.1
GCA_021655295.1
GCA_021655315.1
GCA_021655335.1
GCA_021655355.1
GCA_021655415.1
GCA_021655435.1
GCA_021655455.1
GCA_021655475.1
GCA_021655495.1
GCA_021655515.1

GCA_021655535.1
GCA_021655555.1
GCA_021655575.1
GCA_021655595.1
GCA_021655635.1
GCA_021655715.1
GCA_021655765.1
GCA_021655785.1
GCA_021655845.1
GCA_021665875.1
GCA_021713095.1
GCA_021713115.1
GCA_021725415.1
GCA_021725435.1
GCA_021725455.1
GCA_021725475.1
GCA_021729365.1
GCA_021729385.1
GCA_021729405.1
GCA_021729425.1
GCA_021729445.1
GCA_021729465.1
GCA_021729485.1
GCA_021729505.1
GCA_021729525.1
GCA_021729565.1
GCA_021729585.1
GCA_021729605.1
GCA_021729625.1
GCA_021729645.1
GCA_021729665.1
GCA_021729685.1
GCA_021729705.1
GCA_021729725.1
GCA_021729745.1
GCA_021729765.1
GCA_021729785.1
GCA_021729805.1
GCA_021729845.1
GCA_021729865.1
GCA_021729885.1
GCA_021729905.1
GCA_021729925.1
GCA_021729945.1
GCA_021729965.1
GCA_021729985.1
GCA_021730005.1
GCA_021730025.1
GCA_021730045.1
GCA_021730065.1
GCA_021730085.1
GCA_021730105.1
GCA_021730125.1
GCA_021730145.1

GCA_021730165.1
GCA_021730185.1
GCA_021730205.1
GCA_021730225.1
GCA_021730245.1
GCA_021730265.1
GCA_021730305.1
GCA_021730325.1
GCA_021730415.1
GCA_021733105.1
GCA_021733125.1
GCA_021733145.1
GCA_021733585.3
GCA_021764685.1
GCA_021764725.1
GCA_021765875.1
GCA_021766015.1
GCA_021768305.1
GCA_021768325.1
GCA_021768345.1
GCA_021768365.1
GCA_021768385.1
GCA_021768405.1
GCA_021768425.1
GCA_021777195.1
GCA_021777455.1
GCA_021789175.1
GCA_021789315.1
GCA_021800405.1
GCA_021801725.1
GCA_021869545.1
GCA_021869625.1
GCA_021869645.1
GCA_021869665.1
GCA_021869685.1
GCA_021869705.1
GCA_021869725.1
GCA_021869805.1
GCA_021869995.1
GCA_021870015.1
GCA_021870035.1
GCA_021899435.1
GCA_021899455.1
GCA_021899575.1
GCA_021899615.1
GCA_021899715.1
GCA_021899755.1
GCA_021899855.1
GCA_021903275.1
GCA_021903295.1
GCA_021903335.1
GCA_021903355.1
GCA_021903375.1
GCA_021903395.1

GCA_021903515.1
GCA_021903635.1
GCA_021906975.1
GCA_021906995.1
GCA_021907015.1
GCA_021918005.1
GCA_021919125.1
GCA_021919185.1
GCA_021919285.1
GCA_021919345.1
GCA_021922925.1
GCA_021922985.1
GCA_021923065.1
GCA_021923125.1
GCA_021952465.1
GCA_021952485.1
GCA_021952605.1
GCA_021952645.1
GCA_021952745.1
GCA_021952765.1
GCA_021952865.1
GCA_021952945.1
GCA_021953005.1
GCA_021953085.1
GCA_021953125.1
GCA_021953185.1
GCA_021953265.1
GCA_021957025.1
GCA_021957045.1
GCA_021957065.1
GCA_021957085.1
GCA_021957105.1
GCA_021957125.1
GCA_021957145.1
GCA_021957165.1
GCA_021957185.1
GCA_021957205.1
GCA_022009655.1
GCA_022009815.1
GCA_022009895.1
GCA_022009915.1
GCA_022010015.1
GCA_022014615.1
GCA_022014635.1
GCA_022014655.1
GCA_022014675.1
GCA_022014695.1
GCA_022014715.1
GCA_022014735.1
GCA_022014755.1
GCA_022014775.1
GCA_022014795.1
GCA_022014815.1
GCA_022023755.1

GCA_022023775.1
GCA_022023795.1
GCA_022023815.1
GCA_022023835.1
GCA_022023855.1
GCA_022023875.1
GCA_022023895.1
GCA_022023915.1
GCA_022023955.1
GCA_022023995.1
GCA_022024015.1
GCA_022024035.1
GCA_022024055.1
GCA_022024075.1
GCA_022024095.1
GCA_022024115.1
GCA_022024135.1
GCA_022024175.1
GCA_022024195.1
GCA_022024215.1
GCA_022024235.1
GCA_022024255.1
GCA_022024335.1
GCA_022024355.1
GCA_022026035.1
GCA_022026155.1
GCA_022026295.1
GCA_022026395.1
GCA_022049065.2
GCA_022058185.1
GCA_022058205.1
GCA_022058225.1
GCA_022059845.1
GCA_022059865.1
GCA_022059885.1
GCA_022059905.1
GCA_022063185.1
GCA_022068225.1
GCA_022068245.1
GCA_022068265.1
GCA_022068285.1
GCA_022068405.1
GCA_022068525.1
GCA_022068645.1
GCA_022068765.1
GCA_022068885.1
GCA_022069005.1
GCA_022069085.1
GCA_022069205.1
GCA_022069325.1
GCA_022069445.1
GCA_022069485.1
GCA_022069505.1
GCA_022069545.1

GCA_022069645.1
GCA_022069785.1
GCA_022069905.1
GCA_022070005.1
GCA_022070225.1
GCA_022070425.1
GCA_022070465.1
GCA_022070485.1
GCA_022070505.1
GCA_022070545.1
GCA_022070565.1
GCA_022075545.1
GCA_022075645.1
GCA_022075745.1
GCA_022107375.2
GCA_022117515.1
GCA_022117535.1
GCA_022117635.1
GCA_022117655.1
GCA_022162985.1
GCA_022163245.2
GCA_022163365.1
GCA_022163485.1
GCA_022163605.1
GCA_022170785.1
GCA_022170805.1
GCA_022170825.1
GCA_022174665.1
GCA_022175585.1
GCA_022179365.1
GCA_022179425.1
GCA_022179485.1
GCA_022179545.1
GCA_022179625.1
GCA_022200785.1
GCA_022206215.1
GCA_022212845.1
GCA_022212865.1
GCA_022212885.1
GCA_022212905.1
GCA_022212925.1
GCA_022212945.1
GCA_022212965.1
GCA_022212985.1
GCA_022213005.1
GCA_022213025.1
GCA_022213165.2
GCA_022213385.1
GCA_022213405.2
GCA_022213425.1
GCA_022213445.1
GCA_022220265.1
GCA_022220285.1
GCA_022220305.1

GCA_022220325.1
GCA_022220345.1
GCA_022220365.1
GCA_022220385.1
GCA_022220405.1
GCA_022220425.1
GCA_022220445.1
GCA_022220465.1
GCA_022220485.1
GCA_022220505.1
GCA_022220525.1
GCA_022220545.1
GCA_022220565.1
GCA_022220585.1
GCA_022220605.1
GCA_022220625.1
GCA_022220645.1
GCA_022220665.1
GCA_022220685.1
GCA_022220705.1
GCA_022220725.1
GCA_022220745.1
GCA_022220765.1
GCA_022220785.1
GCA_022220805.1
GCA_022220825.1
GCA_022220845.1
GCA_022220865.1
GCA_022220885.1
GCA_022220905.1
GCA_022220925.1
GCA_022220945.1
GCA_022220965.1
GCA_022220985.1
GCA_022221005.1
GCA_022221025.1
GCA_022221045.1
GCA_022221065.1
GCA_022221085.1
GCA_022221105.1
GCA_022221125.1
GCA_022221145.1
GCA_022221165.1
GCA_022221185.1
GCA_022221205.1
GCA_022221225.1
GCA_022221245.1
GCA_022221265.1
GCA_022221285.1
GCA_022221305.1
GCA_022221325.1
GCA_022221345.1
GCA_022221365.1
GCA_022221385.1

GCA_022221405.1
GCA_022221425.1
GCA_022221445.1
GCA_022221465.1
GCA_022221485.1
GCA_022221505.1
GCA_022221525.1
GCA_022221545.1
GCA_022221565.1
GCA_022221585.1
GCA_022226895.1
GCA_022226915.1
GCA_022226975.1
GCA_022226995.1
GCA_022227035.1
GCA_022259695.1
GCA_022267555.1
GCA_022313395.1
GCA_022313415.2
GCA_022317005.1
GCA_022318385.1
GCA_022318405.1
GCA_022318425.1
GCA_022318445.1
GCA_022318465.1
GCA_022318485.1
GCA_022318505.1
GCA_022318525.1
GCA_022318545.1
GCA_022343085.1
GCA_022343105.1
GCA_022343125.1
GCA_022343145.1
GCA_022343165.1
GCA_022343185.1
GCA_022343205.1
GCA_022343225.1
GCA_022343245.1
GCA_022343265.1
GCA_022343685.1
GCA_022343705.1
GCA_022343725.1
GCA_022343745.1
GCA_022343765.1
GCA_022343785.1
GCA_022343805.1
GCA_022343825.1
GCA_022343945.1
GCA_022343965.1
GCA_022343985.1
GCA_022344005.1
GCA_022344025.1
GCA_022344045.1
GCA_022349925.1

GCA_022349945.1
GCA_022349965.1
GCA_022349985.1
GCA_022350005.1
GCA_022350025.1
GCA_022353525.1
GCA_022353545.1
GCA_022353565.1
GCA_022353585.1
GCA_022353605.1
GCA_022353625.1
GCA_022353645.1
GCA_022353665.1
GCA_022353685.1
GCA_022353705.1
GCA_022353725.1
GCA_022353745.1
GCA_022353765.1
GCA_022353785.1
GCA_022353805.1
GCA_022353825.1
GCA_022353845.1
GCA_022353865.1
GCA_022353885.1
GCA_022353905.1
GCA_022353925.1
GCA_022353945.1
GCA_022353965.1
GCA_022353985.1
GCA_022354005.1
GCA_022354025.1
GCA_022354045.1
GCA_022354065.1
GCA_022354085.1
GCA_022354105.1
GCA_022354125.1
GCA_022354165.1
GCA_022354185.1
GCA_022354205.1
GCA_022354225.1
GCA_022354245.1
GCA_022354265.1
GCA_022354285.1
GCA_022354305.1
GCA_022354325.1
GCA_022354345.1
GCA_022354365.1
GCA_022354385.1
GCA_022354405.1
GCA_022354425.1
GCA_022354445.1
GCA_022354465.1
GCA_022354485.1
GCA_022354505.1

GCA_022354525.1
GCA_022354545.1
GCA_022354565.1
GCA_022354585.1
GCA_022354605.1
GCA_022354625.1
GCA_022354645.1
GCA_022354665.1
GCA_022354685.1
GCA_022354705.1
GCA_022354725.1
GCA_022354745.1
GCA_022354765.1
GCA_022354785.1
GCA_022354825.1
GCA_022354845.1
GCA_022368815.1
GCA_022368895.1
GCA_022369015.1
GCA_022369175.1
GCA_022369275.1
GCA_022369355.1
GCA_022369415.1
GCA_022369435.1
GCA_022369455.1
GCA_022369475.1
GCA_022369495.1
GCA_022369535.1
GCA_022369595.1
GCA_022369695.1
GCA_022369815.1
GCA_022369925.1
GCA_022370375.1
GCA_022370395.1
GCA_022370415.1
GCA_022370435.1
GCA_022370455.1
GCA_022370515.1
GCA_022370635.3
GCA_022370755.2
GCA_022370835.2
GCA_022370935.1
GCA_022370975.1
GCA_022371095.1
GCA_022371215.1
GCA_022372495.1
GCA_022374875.3
GCA_022374895.2
GCA_022374915.3
GCA_022374935.2
GCA_022376295.1
GCA_022376675.1
GCA_022376795.1
GCA_022376815.1

GCA_022376835.1
GCA_022385175.1
GCA_022385195.1
GCA_022385215.1
GCA_022385235.1
GCA_022385255.1
GCA_022385275.1
GCA_022385295.1
GCA_022385315.1
GCA_022385335.1
GCA_022392325.1
GCA_022392805.1
GCA_022394535.1
GCA_022394675.1
GCA_022394795.1
GCA_022404575.1
GCA_022404595.1
GCA_022404615.1
GCA_022404635.1
GCA_022404655.1
GCA_022404675.1
GCA_022404695.1
GCA_022404715.1
GCA_022404735.1
GCA_022404755.1
GCA_022404775.1
GCA_022404795.1
GCA_022404815.1
GCA_022404835.1
GCA_022404855.1
GCA_022404875.1
GCA_022404895.1
GCA_022404915.1
GCA_022404935.1
GCA_022404955.1
GCA_022404975.1
GCA_022404995.1
GCA_022405015.1
GCA_022405035.1
GCA_022405055.1
GCA_022405075.1
GCA_022405145.1
GCA_022405185.1
GCA_022405205.1
GCA_022405235.1
GCA_022405255.1
GCA_022405275.1
GCA_022405295.1
GCA_022405315.1
GCA_022405335.1
GCA_022405355.1
GCA_022405375.1
GCA_022405395.1
GCA_022405415.1

GCA_022405435.1
GCA_022405455.1
GCA_022405475.1
GCA_022405495.1
GCA_022405515.1
GCA_022405535.1
GCA_022405555.1
GCA_022405575.1
GCA_022405595.1
GCA_022405615.1
GCA_022405655.1
GCA_022405675.1
GCA_022405735.1
GCA_022405755.1
GCA_022405775.1
GCA_022405795.1
GCA_022405815.1
GCA_022405835.1
GCA_022405855.1
GCA_022405875.1
GCA_022405895.1
GCA_022405915.1
GCA_022405935.1
GCA_022405955.1
GCA_022405975.1
GCA_022405995.1
GCA_022406015.1
GCA_022406035.1
GCA_022406055.1
GCA_022406075.1
GCA_022406125.1
GCA_022406315.1
GCA_022406435.1
GCA_022406555.1
GCA_022406675.1
GCA_022406795.1
GCA_022406815.1
GCA_022406855.1
GCA_022406915.1
GCA_022406935.1
GCA_022406955.1
GCA_022406975.1
GCA_022406995.1
GCA_022407015.1
GCA_022407035.1
GCA_022407055.1
GCA_022407075.1
GCA_022407095.1
GCA_022407115.1
GCA_022407135.1
GCA_022407155.1
GCA_022407175.1
GCA_022407195.1
GCA_022407215.1

GCA_022407235.1
GCA_022407255.1
GCA_022407275.1
GCA_022407295.1
GCA_022407315.1
GCA_022407335.1
GCA_022407355.1
GCA_022407375.1
GCA_022407395.1
GCA_022407415.1
GCA_022407435.1
GCA_022407455.1
GCA_022407475.1
GCA_022407495.1
GCA_022407515.1
GCA_022407535.1
GCA_022407655.1
GCA_022407775.1
GCA_022407895.1
GCA_022408035.1
GCA_022409135.1
GCA_022409155.1
GCA_022409175.1
GCA_022409195.1
GCA_022409215.1
GCA_022409235.1
GCA_022409255.1
GCA_022409275.1
GCA_022409295.1
GCA_022409315.1
GCA_022409335.1
GCA_022409355.1
GCA_022409375.1
GCA_022409395.1
GCA_022409415.1
GCA_022409435.1
GCA_022409455.1
GCA_022409475.1
GCA_022409495.1
GCA_022409515.1
GCA_022409535.1
GCA_022409555.1
GCA_022409575.1
GCA_022409595.1
GCA_022409615.1
GCA_022410435.1
GCA_022429565.1
GCA_022429585.1
GCA_022429605.1
GCA_022429645.1
GCA_022430505.1
GCA_022430525.1
GCA_022430545.2
GCA_022433565.1

GCA_022437945.1
GCA_022439445.1
GCA_022439465.1
GCA_022439485.1
GCA_022439505.1
GCA_022439525.1
GCA_022439565.1
GCA_022439585.1
GCA_022453545.1
GCA_022453565.1
GCA_022453585.1
GCA_022453605.1
GCA_022453625.1
GCA_022453645.1
GCA_022453665.1
GCA_022453685.1
GCA_022453705.1
GCA_022453725.1
GCA_022453745.1
GCA_022453765.1
GCA_022453785.1
GCA_022453805.1
GCA_022453825.1
GCA_022453845.1
GCA_022453865.1
GCA_022453915.1
GCA_022453935.2
GCA_022459015.1
GCA_022459055.1
GCA_022459075.1
GCA_022459095.1
GCA_022459115.2
GCA_022459135.1
GCA_022459155.1
GCA_022459175.1
GCA_022459295.1
GCA_022459415.1
GCA_022459595.1
GCA_022459755.2
GCA_022459875.1
GCA_022460015.1
GCA_022460155.2
GCA_022460295.1
GCA_022460435.2
GCA_022460575.1
GCA_022460745.2
GCA_022460875.2
GCA_022461025.2
GCA_022466815.1
GCA_022466975.1
GCA_022467135.1
GCA_022467255.1
GCA_022467375.2
GCA_022467435.1

GCA_022467535.1
GCA_022467655.1
GCA_022467735.1
GCA_022467805.1
GCA_022467905.1
GCA_022467975.1
GCA_022468075.1
GCA_022468195.1
GCA_022468295.1
GCA_022468355.1
GCA_022468455.1
GCA_022468565.1
GCA_022468645.2
GCA_022468755.1
GCA_022468795.1
GCA_022468835.2
GCA_022468855.2
GCA_022468985.2
GCA_022469075.1
GCA_022477975.1
GCA_022478075.1
GCA_022488185.1
GCA_022488205.1
GCA_022488405.1
GCA_022488425.1
GCA_022488445.1
GCA_022488465.1
GCA_022492855.1
GCA_022492875.1
GCA_022492895.1
GCA_022492915.1
GCA_022492935.1
GCA_022492955.1
GCA_022492975.1
GCA_022492995.1
GCA_022493015.1
GCA_022493095.1
GCA_022493115.1
GCA_022493135.1
GCA_022493155.1
GCA_022493175.1
GCA_022493195.1
GCA_022493215.1
GCA_022493235.1
GCA_022493255.1
GCA_022493275.1
GCA_022493295.1
GCA_022493315.1
GCA_022493335.1
GCA_022493355.1
GCA_022493375.1
GCA_022493395.1
GCA_022493415.1
GCA_022493435.1

GCA_022493455.1
GCA_022493475.1
GCA_022493495.1
GCA_022493515.1
GCA_022493535.1
GCA_022493555.1
GCA_022493575.1
GCA_022493595.1
GCA_022493615.1
GCA_022493635.1
GCA_022493655.1
GCA_022493675.1
GCA_022493695.1
GCA_022493715.1
GCA_022493735.1
GCA_022493755.1
GCA_022493775.1
GCA_022493795.1
GCA_022493815.1
GCA_022493835.1
GCA_022493855.1
GCA_022493875.1
GCA_022493895.1
GCA_022493915.1
GCA_022493955.1
GCA_022493975.1
GCA_022493995.1
GCA_022494015.1
GCA_022494035.1
GCA_022494055.1
GCA_022494075.1
GCA_022494095.1
GCA_022494115.1
GCA_022494135.1
GCA_022494155.1
GCA_022494175.1
GCA_022494195.1
GCA_022494215.1
GCA_022494235.1
GCA_022494255.1
GCA_022494275.1
GCA_022494295.1
GCA_022494315.1
GCA_022494335.1
GCA_022494355.1
GCA_022494375.1
GCA_022494395.1
GCA_022494415.1
GCA_022494435.1
GCA_022494455.1
GCA_022494475.1
GCA_022494495.1
GCA_022494515.1
GCA_022494545.1

GCA_022494565.1
GCA_022494585.1
GCA_022494605.1
GCA_022494625.1
GCA_022494645.1
GCA_022494665.1
GCA_022494685.1
GCA_022494705.1
GCA_022494725.1
GCA_022494745.1
GCA_022494765.1
GCA_022494785.1
GCA_022494805.1
GCA_022494825.1
GCA_022494855.1
GCA_022494905.1
GCA_022494925.1
GCA_022494945.1
GCA_022494965.1
GCA_022514105.1
GCA_022530585.1
GCA_022531845.1
GCA_022531965.1
GCA_022532085.1
GCA_022532105.1
GCA_022532125.1
GCA_022532145.1
GCA_022533465.1
GCA_022533485.1
GCA_022533505.1
GCA_022533525.1
GCA_022533545.1
GCA_022533565.1
GCA_022533585.1
GCA_022537035.1
GCA_022537925.1
GCA_022539405.1
GCA_022539425.1
GCA_022539555.1
GCA_022544835.1
GCA_022548575.1
GCA_022557175.1
GCA_022557195.1
GCA_022557215.1
GCA_022557235.1
GCA_022557255.1
GCA_022557275.1
GCA_022558365.1
GCA_022558405.1
GCA_022558425.1
GCA_022558445.1
GCA_022558505.1
GCA_022558525.1
GCA_022558565.1

GCA_022558585.1
GCA_022558605.1
GCA_022558625.1
GCA_022558645.1
GCA_022558665.1
GCA_022558685.1
GCA_022558705.1
GCA_022558725.1
GCA_022558745.1
GCA_022558765.1
GCA_022558785.1
GCA_022558805.1
GCA_022558825.1
GCA_022558845.1
GCA_022558865.1
GCA_022558885.1
GCA_022558905.1
GCA_022558925.1
GCA_022558945.1
GCA_022558965.1
GCA_022558985.1
GCA_022559005.1
GCA_022559025.1
GCA_022559045.1
GCA_022559065.1
GCA_022559245.1
GCA_022559265.1
GCA_022559285.1
GCA_022559305.1
GCA_022559325.1
GCA_022559345.1
GCA_022559365.1
GCA_022559385.1
GCA_022559405.1
GCA_022559425.1
GCA_022559445.1
GCA_022559465.1
GCA_022559485.1
GCA_022559505.1
GCA_022559525.1
GCA_022559545.1
GCA_022559585.1
GCA_022559645.1
GCA_022559685.1
GCA_022559705.1
GCA_022559725.1
GCA_022559745.1
GCA_022559765.1
GCA_022559785.1
GCA_022559805.1
GCA_022559825.1
GCA_022559855.1
GCA_022559875.1
GCA_022569415.1

GCA_022569435.1
GCA_022569455.1
GCA_022569475.1
GCA_022569495.1
GCA_022569515.1
GCA_022569535.1
GCA_022569555.1
GCA_022569575.1
GCA_022569595.1
GCA_022569615.1
GCA_022569635.1
GCA_022569655.1
GCA_022569675.1
GCA_022569695.1
GCA_022569795.1
GCA_022569815.1
GCA_022569835.1
GCA_022569855.1
GCA_022569895.1
GCA_022569915.1
GCA_022569935.1
GCA_022569975.1
GCA_022569995.1
GCA_022570115.1
GCA_022570235.1
GCA_022570415.1
GCA_022570435.1
GCA_022570455.1
GCA_022570475.1
GCA_022570515.1
GCA_022570695.1
GCA_022570715.1
GCA_022570755.1
GCA_022576045.1
GCA_022576915.1
GCA_022577075.1
GCA_022591215.1
GCA_022591335.1
GCA_022591475.1
GCA_022591595.1
GCA_022591735.1
GCA_022591895.1
GCA_022592015.1
GCA_022592155.1
GCA_022592275.1
GCA_022592395.1
GCA_022592575.1
GCA_022592735.1
GCA_022605145.1
GCA_022605305.1
GCA_022605325.1
GCA_022605345.1
GCA_022625045.1
GCA_022630555.1

GCA_022630575.1
GCA_022631175.1
GCA_022631195.1
GCA_022631215.1
GCA_022631235.2
GCA_022637295.1
GCA_022637315.1
GCA_022637335.1
GCA_022637355.1
GCA_022637375.1
GCA_022637395.1
GCA_022637415.1
GCA_022637435.1
GCA_022637455.1
GCA_022637475.1
GCA_022637495.1
GCA_022637515.1
GCA_022637535.1
GCA_022637555.1
GCA_022637595.1
GCA_022637755.1
GCA_022637915.1
GCA_022638055.1
GCA_022638175.1
GCA_022646275.1
GCA_022646745.1
GCA_022646885.1
GCA_022647025.1
GCA_022647185.1
GCA_022647325.1
GCA_022647505.1
GCA_022647665.1
GCA_022647825.1
GCA_022647945.1
GCA_022648125.1
GCA_022648285.1
GCA_022648425.1
GCA_022648595.1
GCA_022648765.1
GCA_022648965.1
GCA_022649105.1
GCA_022649245.1
GCA_022649565.1
GCA_022654015.1
GCA_022654245.1
GCA_022654545.1
GCA_022654795.1
GCA_022655055.1
GCA_022655355.1
GCA_022655485.1
GCA_022655595.1
GCA_022655645.1
GCA_022655665.1
GCA_022655685.1

GCA_022655705.1
GCA_022670655.1
GCA_022670675.1
GCA_022670695.1
GCA_022670715.1
GCA_022670735.1
GCA_022670755.1
GCA_022670775.1
GCA_022670795.1
GCA_022670815.1
GCA_022670835.1
GCA_022674245.1
GCA_022682045.1
GCA_022682305.1
GCA_022682325.1
GCA_022682355.1
GCA_022691325.1
GCA_022691345.1
GCA_022691365.1
GCA_022691385.1
GCA_022691405.1
GCA_022691425.1
GCA_022691445.1
GCA_022691465.1
GCA_022691485.1
GCA_022691665.1
GCA_022691685.1
GCA_022693165.1
GCA_022693185.1
GCA_022693205.1
GCA_022693225.1
GCA_022693245.1
GCA_022693265.1
GCA_022693285.1
GCA_022693305.1
GCA_022693325.1
GCA_022693345.1
GCA_022695595.1
GCA_022695635.1
GCA_022695655.1
GCA_022695865.1
GCA_022699225.1
GCA_022699245.1
GCA_022699265.1
GCA_022699285.1
GCA_022699305.1
GCA_022699325.1
GCA_022699345.1
GCA_022699365.1
GCA_022699385.1
GCA_022699405.1
GCA_022699425.1
GCA_022699445.1
GCA_022699465.1

GCA_022699485.1
GCA_022699505.1
GCA_022699525.1
GCA_022699545.1
GCA_022699565.1
GCA_022699585.1
GCA_022699605.1
GCA_022700635.2
GCA_022700735.1
GCA_022700755.1
GCA_022700775.1
GCA_022700795.1
GCA_022700815.1
GCA_022700835.1
GCA_022700855.1
GCA_022700875.1
GCA_022700895.1
GCA_022700915.1
GCA_022700935.1
GCA_022700955.1
GCA_022700975.1
GCA_022700995.1
GCA_022701015.1
GCA_022701035.1
GCA_022701055.1
GCA_022701095.1
GCA_022701115.1
GCA_022701135.1
GCA_022701175.1
GCA_022701195.1
GCA_022701215.1
GCA_022701235.1
GCA_022701295.1
GCA_022701315.1
GCA_022703095.1
GCA_022749195.1
GCA_022749215.1
GCA_022749295.1
GCA_022749455.1
GCA_022749475.1
GCA_022749495.1
GCA_022749515.1
GCA_022749535.1
GCA_022749555.1
GCA_022749575.1
GCA_022749595.1
GCA_022749615.1
GCA_022749635.1
GCA_022749655.1
GCA_022751235.1
GCA_022751255.1
GCA_022759505.1
GCA_022759525.1
GCA_022759545.1

GCA_022759565.1
GCA_022759585.1
GCA_022759605.1
GCA_022759625.1
GCA_022759645.1
GCA_022759665.1
GCA_022759685.1
GCA_022759705.1
GCA_022759725.1
GCA_022759745.1
GCA_022759765.1
GCA_022759785.1
GCA_022759805.1
GCA_022759825.1
GCA_022759845.1
GCA_022759865.1
GCA_022759885.1
GCA_022759905.1
GCA_022759925.1
GCA_022759945.1
GCA_022759965.1
GCA_022759985.1
GCA_022760005.1
GCA_022760025.1
GCA_022760135.1
GCA_022760155.1
GCA_022760175.1
GCA_022760195.1
GCA_022760215.1
GCA_022760255.1
GCA_022760275.1
GCA_022760295.1
GCA_022788635.1
GCA_022788655.1
GCA_022788675.1
GCA_022788695.1
GCA_022788715.1
GCA_022788735.1
GCA_022807935.1
GCA_022807955.1
GCA_022807975.1
GCA_022807995.1
GCA_022808015.1
GCA_022809675.1
GCA_022809695.1
GCA_022809715.1
GCA_022809755.1
GCA_022809775.1
GCA_022809795.1
GCA_022809815.1
GCA_022809835.2
GCA_022809855.1
GCA_022809875.1
GCA_022810625.1

GCA_022810665.1
GCA_022810685.1
GCA_022810705.1
GCA_022810725.1
GCA_022810745.1
GCA_022810765.1
GCA_022810785.1
GCA_022810805.1
GCA_022810825.1
GCA_022810865.1
GCA_022810885.1
GCA_022810905.1
GCA_022810925.1
GCA_022810945.1
GCA_022810965.1
GCA_022810985.1
GCA_022811005.1
GCA_022811025.1
GCA_022811045.1
GCA_022811065.1
GCA_022811105.1
GCA_022811525.1
GCA_022811545.1
GCA_022811565.1
GCA_022811585.1
GCA_022811605.1
GCA_022811625.1
GCA_022811645.1
GCA_022811665.1
GCA_022811685.1
GCA_022811705.1
GCA_022811725.1
GCA_022811805.1
GCA_022811825.1
GCA_022811865.1
GCA_022811885.1
GCA_022811905.1
GCA_022811925.1
GCA_022811945.1
GCA_022812045.1
GCA_022812155.1
GCA_022812285.1
GCA_022813675.1
GCA_022814725.1
GCA_022814825.1
GCA_022815905.2
GCA_022816845.1
GCA_022818495.1
GCA_022819225.1
GCA_022819245.1
GCA_022819265.1
GCA_022819285.1
GCA_022819305.1
GCA_022819325.1

GCA_022819405.1
GCA_022819425.1
GCA_022819445.1
GCA_022819565.1
GCA_022819715.1
GCA_022819915.1
GCA_022820045.1
GCA_022820235.1
GCA_022820405.1
GCA_022820565.1
GCA_022827545.1
GCA_022827705.1
GCA_022827865.1
GCA_022828425.1
GCA_022828475.1
GCA_022828525.1
GCA_022828555.1
GCA_022828575.1
GCA_022828975.1
GCA_022832715.1
GCA_022832735.1
GCA_022832755.1
GCA_022832775.1
GCA_022832815.1
GCA_022832835.1
GCA_022832855.1
GCA_022832875.1
GCA_022832895.1
GCA_022832915.1
GCA_022832935.1
GCA_022832955.1
GCA_022832975.1
GCA_022833015.1
GCA_022833145.1
GCA_022836875.1
GCA_022836895.1
GCA_022836935.1
GCA_022836955.1
GCA_022845595.1
GCA_022845615.1
GCA_022845635.1
GCA_022845675.1
GCA_022845695.1
GCA_022845715.1
GCA_022845735.1
GCA_022845755.1
GCA_022845775.1
GCA_022845795.1
GCA_022845815.1
GCA_022845835.1
GCA_022845935.1
GCA_022845955.1
GCA_022845975.1
GCA_022846015.1

GCA_022846035.1
GCA_022846055.1
GCA_022846075.1
GCA_022846095.1
GCA_022846115.1
GCA_022846135.1
GCA_022846195.1
GCA_022846215.1
GCA_022846235.1
GCA_022846255.1
GCA_022846275.1
GCA_022846295.1
GCA_022846315.1
GCA_022846335.1
GCA_022846355.1
GCA_022846375.1
GCA_022846395.1
GCA_022846415.1
GCA_022846435.1
GCA_022846455.1
GCA_022846475.1
GCA_022846495.1
GCA_022846515.1
GCA_022846535.1
GCA_022846575.1
GCA_022846615.1
GCA_022846655.1
GCA_022846675.1
GCA_022865125.1
GCA_022867795.2
GCA_022869105.1
GCA_022869125.1
GCA_022869145.1
GCA_022869165.1
GCA_022869565.1
GCA_022869585.1
GCA_022869605.1
GCA_022869625.1
GCA_022869645.1
GCA_022869665.1
GCA_022869685.1
GCA_022869705.1
GCA_022869765.1
GCA_022869785.1
GCA_022869825.1
GCA_022869845.1
GCA_022869865.1
GCA_022869885.1
GCA_022869905.1
GCA_022869925.1
GCA_022869945.1
GCA_022869965.1
GCA_022869985.1
GCA_022870005.1

GCA_022870025.1
GCA_022870045.1
GCA_022870065.1
GCA_022870085.1
GCA_022870105.1
GCA_022870125.1
GCA_022870145.1
GCA_022870165.1
GCA_022870185.1
GCA_022870205.1
GCA_022870225.1
GCA_022870245.1
GCA_022870265.1
GCA_022870285.1
GCA_022870305.1
GCA_022870325.1
GCA_022870345.1
GCA_022870365.1
GCA_022870385.1
GCA_022870405.1
GCA_022870425.1
GCA_022870445.1
GCA_022870465.1
GCA_022870525.1
GCA_022870545.1
GCA_022870565.1
GCA_022870585.1
GCA_022870605.1
GCA_022870625.1
GCA_022870645.1
GCA_022870685.1
GCA_022870705.1
GCA_022870725.1
GCA_022870745.1
GCA_022870765.1
GCA_022870785.1
GCA_022870805.1
GCA_022870825.1
GCA_022870845.1
GCA_022870865.1
GCA_022870885.1
GCA_022870905.1
GCA_022870925.1
GCA_022870945.1
GCA_022870965.1
GCA_022870985.1
GCA_022871005.1
GCA_022871025.1
GCA_022871045.1
GCA_022871065.1
GCA_022871085.1
GCA_022871105.1
GCA_022871125.1
GCA_022871145.1

GCA_022879575.1
GCA_022879595.1
GCA_022879615.1
GCA_022879815.1
GCA_022916135.2
GCA_022916555.2
GCA_022918835.1
GCA_022919035.1
GCA_022919055.1
GCA_022919095.1
GCA_022919115.1
GCA_022919135.1
GCA_022919155.1
GCA_022919175.1
GCA_022919195.1
GCA_022919215.1
GCA_022919335.1
GCA_022919455.1
GCA_022919575.1
GCA_022919715.1
GCA_022919835.1
GCA_022919895.1
GCA_022919915.1
GCA_022921035.1
GCA_022921055.1
GCA_022921075.1
GCA_022921095.1
GCA_022921115.1
GCA_022921135.1
GCA_022921155.1
GCA_022921175.1
GCA_022921195.1
GCA_022921215.1
GCA_022921235.1
GCA_022921255.1
GCA_022921275.1
GCA_022921295.1
GCA_022921315.1
GCA_022921335.1
GCA_022921355.1
GCA_022921575.1
GCA_022921695.1
GCA_022921755.1
GCA_022921775.1
GCA_022921795.1
GCA_022921815.1
GCA_022921835.1
GCA_022921855.1
GCA_022921875.1
GCA_022921895.1
GCA_022921915.1
GCA_022921935.1
GCA_022921955.1
GCA_022921975.1

GCA_022921995.1
GCA_022922015.1
GCA_022922035.1
GCA_022922055.1
GCA_022922175.1
GCA_022922295.1
GCA_022922415.1
GCA_022922515.1
GCA_022922655.1
GCA_022922775.1
GCA_022922915.1
GCA_022922955.1
GCA_022922975.1
GCA_022923015.1
GCA_022923035.1
GCA_022923055.1
GCA_022923095.1
GCA_022923115.1
GCA_022923135.1
GCA_022923155.1
GCA_022923275.1
GCA_022923395.1
GCA_022923515.1
GCA_022923555.1
GCA_022923575.1
GCA_022923595.1
GCA_022923615.1
GCA_022923635.1
GCA_022923655.1
GCA_022923675.1
GCA_022923695.1
GCA_022923775.1
GCA_022923915.1
GCA_022924035.1
GCA_022924175.1
GCA_022924275.1
GCA_022924355.1
GCA_022924375.1
GCA_022924395.1
GCA_022924415.1
GCA_022924435.1
GCA_022924455.1
GCA_022924475.1
GCA_022924495.1
GCA_022924515.1
GCA_022924535.1
GCA_022924555.1
GCA_022924575.1
GCA_022924655.1
GCA_022924735.1
GCA_022924875.1
GCA_022924995.1
GCA_022925175.1
GCA_022925215.1

GCA_022925255.1
GCA_022925275.1
GCA_022925295.1
GCA_022925415.1
GCA_022925575.1
GCA_022925675.1
GCA_022925755.1
GCA_022925775.1
GCA_022925795.1
GCA_022925835.1
GCA_022925855.1
GCA_022925875.1
GCA_022925905.1
GCA_022925925.1
GCA_022925945.1
GCA_022930885.1
GCA_022936085.1
GCA_022936265.1
GCA_022936345.1
GCA_022936365.1
GCA_022936385.1
GCA_022936425.1
GCA_022936485.1
GCA_022964775.1
GCA_022964795.1
GCA_022964815.1
GCA_022964835.1
GCA_022964855.1
GCA_022964875.1
GCA_022964895.1
GCA_022964935.1
GCA_022964955.1
GCA_022964995.1
GCA_022965695.1
GCA_022965715.1
GCA_022965735.1
GCA_022965755.1
GCA_022966105.1
GCA_022969555.2
GCA_022982415.1
GCA_022982435.1
GCA_022982555.1
GCA_022982575.1
GCA_022982595.1
GCA_022982615.1
GCA_022982715.1
GCA_022982835.1
GCA_022982975.1
GCA_022983135.1
GCA_022983255.1
GCA_022984115.1
GCA_022984195.1
GCA_022985515.1
GCA_022985535.1

GCA_022985555.1
GCA_022985575.1
GCA_022985595.1
GCA_022985615.1
GCA_022985635.2
GCA_023008185.1
GCA_023008225.1
GCA_023008245.1
GCA_023008265.1
GCA_023008285.1
GCA_023008305.1
GCA_023008325.1
GCA_023008345.1
GCA_023008365.1
GCA_023008385.1
GCA_023008405.1
GCA_023008425.1
GCA_023016265.1
GCA_023016285.1
GCA_023016305.1
GCA_023016345.1
GCA_023016365.1
GCA_023016385.1
GCA_023016405.1
GCA_023016425.1
GCA_023016445.1
GCA_023022885.1
GCA_023022905.1
GCA_023022925.1
GCA_023022945.1
GCA_023022965.1
GCA_023022985.1
GCA_023023005.1
GCA_023023025.1
GCA_023023045.1
GCA_023023065.1
GCA_023023085.1
GCA_023023125.1
GCA_023023145.1
GCA_023023265.1
GCA_023023665.1
GCA_023023685.1
GCA_023023705.1
GCA_023023725.1
GCA_023023745.1
GCA_023023865.1
GCA_023023985.1
GCA_023024425.1
GCA_023024545.1
GCA_023025805.1
GCA_023035175.1
GCA_023035295.1
GCA_023035415.1
GCA_023035575.1

GCA_023035675.1
GCA_023035795.1
GCA_023035815.1
GCA_023035835.1
GCA_023035855.1
GCA_023035875.1
GCA_023037315.1
GCA_023038235.1
GCA_023038255.1
GCA_023052945.1
GCA_023053515.1
GCA_023054035.2
GCA_023065975.1
GCA_023066175.1
GCA_023066195.1
GCA_023066605.1
GCA_023066625.1
GCA_023066645.1
GCA_023066665.1
GCA_023066685.1
GCA_023066705.1
GCA_023066725.1
GCA_023066745.1
GCA_023066765.1
GCA_023066785.1
GCA_023066805.1
GCA_023066825.1
GCA_023066845.1
GCA_023066865.1
GCA_023078355.1
GCA_023078375.1
GCA_023078395.1
GCA_023078415.1
GCA_023078435.1
GCA_023078455.1
GCA_023078475.1
GCA_023078495.1
GCA_023078515.1
GCA_023078835.1
GCA_023092945.2
GCA_023093135.2
GCA_023093835.1
GCA_023093855.1
GCA_023093875.1
GCA_023093895.1
GCA_023093915.1
GCA_023093935.1
GCA_023093975.1
GCA_023093995.1
GCA_023094015.1
GCA_023094055.1
GCA_023100665.1
GCA_023100685.1
GCA_023100705.1

GCA_023100725.1
GCA_023100745.1
GCA_023100765.1
GCA_023100785.1
GCA_023100805.1
GCA_023100825.1
GCA_023100845.1
GCA_023100865.1
GCA_023100885.1
GCA_023100905.1
GCA_023100925.1
GCA_023100945.1
GCA_023100965.1
GCA_023100985.1
GCA_023101005.1
GCA_023101025.1
GCA_023101045.1
GCA_023101065.1
GCA_023101085.1
GCA_023101105.1
GCA_023101125.1
GCA_023101145.1
GCA_023101245.1
GCA_023101265.1
GCA_023101285.1
GCA_023101305.1
GCA_023101325.1
GCA_023101345.1
GCA_023101665.1
GCA_023101685.1
GCA_023101705.1
GCA_023115375.1
GCA_023115425.1
GCA_023115465.1
GCA_023117055.1
GCA_023151555.2
GCA_023158855.1
GCA_023158875.1
GCA_023158895.3
GCA_023158915.1
GCA_023158935.1
GCA_023159095.1
GCA_023159115.1
GCA_023159135.1
GCA_023159325.1
GCA_023159405.1
GCA_023161985.2
GCA_023162015.2
GCA_023167125.1
GCA_023167545.1
GCA_023167605.1
GCA_023167685.1
GCA_023168305.1
GCA_023168325.1

GCA_023168345.1
GCA_023168365.1
GCA_023168385.1
GCA_023168405.1
GCA_023169525.1
GCA_023169585.1
GCA_023169605.1
GCA_023169625.1
GCA_023169645.1
GCA_023169665.1
GCA_023169785.1
GCA_023169805.1
GCA_023169825.1
GCA_023169845.1
GCA_023169865.1
GCA_023169885.1
GCA_023169905.1
GCA_023169925.1
GCA_023169945.1
GCA_023169965.1
GCA_023170005.1
GCA_023170025.1
GCA_023170045.1
GCA_023170065.1
GCA_023170545.1
GCA_023182775.2
GCA_023182815.2
GCA_023182835.2
GCA_023184535.1
GCA_023184555.1
GCA_023195715.1
GCA_023195735.1
GCA_023195755.1
GCA_023195775.1
GCA_023195795.1
GCA_023195815.2
GCA_023195835.1
GCA_023204675.1
GCA_023204875.1
GCA_023204955.1
GCA_023204975.1
GCA_023204995.1
GCA_023205015.1
GCA_023205035.1
GCA_023205055.1
GCA_023205075.1
GCA_023205095.1
GCA_023205265.1
GCA_023205415.1
GCA_023205595.1
GCA_023205755.1
GCA_023205795.1
GCA_023205815.1
GCA_023205835.1

GCA_023205855.1
GCA_023205895.1
GCA_023205915.1
GCA_023205935.1
GCA_023205955.1
GCA_023205975.1
GCA_023205995.1
GCA_023206015.1
GCA_023206035.1
GCA_023206055.1
GCA_023206215.1
GCA_023206395.1
GCA_023206515.1
GCA_023206595.1
GCA_023206615.1
GCA_023206635.1
GCA_023206655.1
GCA_023206675.1
GCA_023206695.1
GCA_023206715.1
GCA_023206735.1
GCA_023206755.1
GCA_023206775.1
GCA_023206795.1
GCA_023207855.1
GCA_023207975.1
GCA_023207995.1
GCA_023208015.1
GCA_023208035.1
GCA_023208055.1
GCA_023208075.1
GCA_023208095.1
GCA_023208115.1
GCA_023208135.1
GCA_023208155.1
GCA_023208175.1
GCA_023208195.1
GCA_023212325.2
GCA_023212385.2
GCA_023212425.2
GCA_023212445.2
GCA_023212565.2
GCA_023212585.2
GCA_023212925.1
GCA_023213175.1
GCA_023213195.1
GCA_023213215.1
GCA_023213235.1
GCA_023221535.1
GCA_023221555.1
GCA_023221575.1
GCA_023221595.1
GCA_023221615.1
GCA_023221635.1

GCA_023221655.1
GCA_023221695.1
GCA_023223475.1
GCA_023238245.1
GCA_023238285.1
GCA_023238325.1
GCA_023238365.1
GCA_023238405.1
GCA_023238485.1
GCA_023238525.1
GCA_023238565.1
GCA_023238625.1
GCA_023238665.1
GCA_023242175.1
GCA_023242195.1
GCA_023242235.1
GCA_023242925.1
GCA_023243115.1
GCA_023272715.1
GCA_023272735.1
GCA_023272755.1
GCA_023272775.1
GCA_023272795.1
GCA_023272815.1
GCA_023273835.1
GCA_023273855.1
GCA_023273875.1
GCA_023273895.1
GCA_023277345.1
GCA_023277365.1
GCA_023277445.1
GCA_023277465.1
GCA_023277485.1
GCA_023277505.1
GCA_023277525.1
GCA_023277545.1
GCA_023277565.1
GCA_023277585.1
GCA_023277605.1
GCA_023277625.1
GCA_023277645.1
GCA_023277665.1
GCA_023277685.1
GCA_023277705.1
GCA_023277725.1
GCA_023277745.1
GCA_023277765.1
GCA_023277785.1
GCA_023277805.1
GCA_023277825.1
GCA_023277845.1
GCA_023277865.1
GCA_023277925.1
GCA_023277945.1

GCA_023277965.1
GCA_023278005.1
GCA_023278025.1
GCA_023278045.1
GCA_023278065.1
GCA_023278085.1
GCA_023278105.1
GCA_023278125.1
GCA_023278145.1
GCA_023278165.1
GCA_023278185.1
GCA_023278205.1
GCA_023278225.1
GCA_023278245.1
GCA_023278265.1
GCA_023278285.1
GCA_023278305.1
GCA_023278325.1
GCA_023278365.1
GCA_023278385.1
GCA_023278405.1
GCA_023278425.1
GCA_023278445.1
GCA_023278465.1
GCA_023278485.1
GCA_023278505.1
GCA_023278525.1
GCA_023278545.1
GCA_023278615.1
GCA_023278635.1
GCA_023279165.1
GCA_023299185.1
GCA_023299225.1
GCA_023299325.1
GCA_023299345.1
GCA_023299425.1
GCA_023299445.1
GCA_023299465.1
GCA_023299485.1
GCA_023299505.1
GCA_023299525.1
GCA_023299545.1
GCA_023299565.1
GCA_023299585.1
GCA_023299605.1
GCA_023299625.1
GCA_023299645.1
GCA_023299665.1
GCA_023299685.1
GCA_023299705.1
GCA_023299725.1
GCA_023299745.1
GCA_023299765.1
GCA_023299785.1

GCA_023299805.1
GCA_023300025.1
GCA_023300045.1
GCA_023300165.1
GCA_023300185.1
GCA_023302925.1
GCA_023303005.1
GCA_023303065.1
GCA_023311875.1
GCA_023311895.1
GCA_023311915.1
GCA_023314135.1
GCA_023330585.1
GCA_023330605.1
GCA_023330625.1
GCA_023330665.1
GCA_023330685.1
GCA_023330705.1
GCA_023330725.1
GCA_023330765.1
GCA_023330785.1
GCA_023330805.1
GCA_023330825.1
GCA_023330845.1
GCA_023330865.1
GCA_023330885.1
GCA_023330905.1
GCA_023333675.1
GCA_023347215.1
GCA_023347235.1
GCA_023347255.1
GCA_023347275.1
GCA_023347295.1
GCA_023347315.1
GCA_023347335.1
GCA_023347355.1
GCA_023347535.1
GCA_023347665.1
GCA_023347685.1
GCA_023347705.1
GCA_023347725.1
GCA_023348385.1
GCA_023348465.1
GCA_023348525.1
GCA_023360975.1
GCA_023360995.1
GCA_023361015.1
GCA_023361035.1
GCA_023361055.1
GCA_023361075.1
GCA_023361095.1
GCA_023361115.1
GCA_023361135.1
GCA_023361155.1

GCA_023361175.1
GCA_023361195.1
GCA_023361215.1
GCA_023361235.1
GCA_023361255.1
GCA_023361275.1
GCA_023361815.1
GCA_023361835.1
GCA_023361855.1
GCA_023361875.1
GCA_023361895.1
GCA_023361915.1
GCA_023361935.1
GCA_023361955.1
GCA_023361975.1
GCA_023361995.1
GCA_023362015.1
GCA_023362035.1
GCA_023362055.1
GCA_023362075.1
GCA_023362095.1
GCA_023362115.1
GCA_023362135.1
GCA_023362155.1
GCA_023362175.1
GCA_023362195.1
GCA_023362215.1
GCA_023362235.1
GCA_023362255.1
GCA_023362275.1
GCA_023369715.1
GCA_023370075.1
GCA_023370095.1
GCA_023370115.1
GCA_023370155.1
GCA_023370175.1
GCA_023373485.1
GCA_023373505.1
GCA_023373525.1
GCA_023373545.1
GCA_023373565.1
GCA_023373585.1
GCA_023373605.1
GCA_023373625.1
GCA_023373645.1
GCA_023373665.1
GCA_023373685.1
GCA_023373725.1
GCA_023373745.1
GCA_023373765.1
GCA_023373785.1
GCA_023374165.1
GCA_023374185.1
GCA_023374205.1

GCA_023374225.1
GCA_023374245.1
GCA_023374275.1
GCA_023374295.1
GCA_023374315.1
GCA_023374335.1
GCA_023374355.1
GCA_023374375.1
GCA_023374395.1
GCA_023374415.1
GCA_023374435.1
GCA_023374455.1
GCA_023374475.1
GCA_023374495.1
GCA_023374515.1
GCA_023374535.1
GCA_023374575.1
GCA_023374595.1
GCA_023374615.1
GCA_023374635.1
GCA_023374655.1
GCA_023374675.1
GCA_023374695.1
GCA_023374715.1
GCA_023374735.1
GCA_023374755.1
GCA_023374775.1
GCA_023374795.1
GCA_023374815.1
GCA_023374835.1
GCA_023374855.1
GCA_023374875.1
GCA_023374895.1
GCA_023374935.1
GCA_023374955.1
GCA_023374975.1
GCA_023374995.1
GCA_023375015.1
GCA_023375035.1
GCA_023375055.1
GCA_023375075.1
GCA_023375105.1
GCA_023375135.1
GCA_023375155.1
GCA_023375175.1
GCA_023375205.1
GCA_023375225.1
GCA_023375245.1
GCA_023375265.1
GCA_023375285.1
GCA_023375305.1
GCA_023375325.1
GCA_023375345.1
GCA_023375365.1

GCA_023375385.1
GCA_023375405.1
GCA_023375425.1
GCA_023375445.1
GCA_023375465.1
GCA_023375485.1
GCA_023375505.1
GCA_023375525.1
GCA_023375545.1
GCA_023375565.1
GCA_023375585.1
GCA_023375605.1
GCA_023375625.1
GCA_023375815.1
GCA_023376025.1
GCA_023376055.1
GCA_023376095.1
GCA_023379965.1
GCA_023379985.1
GCA_023380005.1
GCA_023380025.1
GCA_023380045.1
GCA_023380065.1
GCA_023380185.1
GCA_023380205.1
GCA_023380225.1
GCA_023380245.1
GCA_023380265.1
GCA_023497965.3
GCA_023497985.1
GCA_023498005.1
GCA_023499255.1
GCA_023499275.1
GCA_023503985.1
GCA_023508855.1
GCA_023508875.1
GCA_023509015.1
GCA_023509255.1
GCA_023509275.1
GCA_023509295.1
GCA_023509315.1
GCA_023515845.1
GCA_023515865.1
GCA_023515885.1
GCA_023515905.1
GCA_023515935.1
GCA_023515955.1
GCA_023515975.1
GCA_023515995.1
GCA_023516035.1
GCA_023516055.1
GCA_023516075.1
GCA_023516095.1
GCA_023516115.1

GCA_023516135.1
GCA_023516155.1
GCA_023516175.1
GCA_023516195.1
GCA_023516215.1
GCA_023516235.1
GCA_023516255.1
GCA_023516275.1
GCA_023516295.1
GCA_023516315.1
GCA_023516335.1
GCA_023516355.1
GCA_023516375.1
GCA_023516395.1
GCA_023516415.1
GCA_023516435.1
GCA_023517155.1
GCA_023517175.1
GCA_023517195.1
GCA_023517215.1
GCA_023517235.1
GCA_023517255.1
GCA_023517695.1
GCA_023517715.1
GCA_023517735.1
GCA_023517775.1
GCA_023517795.1
GCA_023517815.1
GCA_023517835.1
GCA_023517855.1
GCA_023517875.1
GCA_023517895.1
GCA_023517915.1
GCA_023517935.1
GCA_023518055.1
GCA_023518135.1
GCA_023518155.1
GCA_023518175.1
GCA_023518195.1
GCA_023518215.1
GCA_023518295.1
GCA_023518315.1
GCA_023518335.1
GCA_023518355.1
GCA_023518395.1
GCA_023520575.1
GCA_023520735.1
GCA_023520755.1
GCA_023520775.1
GCA_023520795.1
GCA_023520815.1
GCA_023520835.1
GCA_023520855.1
GCA_023520895.1

GCA_023521015.1
GCA_023521175.1
GCA_023521275.1
GCA_023521475.1
GCA_023521595.1
GCA_023521615.1
GCA_023521635.1
GCA_023521655.1
GCA_023523445.1
GCA_023523545.1
GCA_023523715.1
GCA_023523795.1
GCA_023525255.1
GCA_023525915.1
GCA_023538935.1
GCA_023538955.1
GCA_023538975.1
GCA_023538995.1
GCA_023539015.1
GCA_023539035.1
GCA_023539135.1
GCA_023539285.1
GCA_023546765.1
GCA_023546785.1
GCA_023546805.1
GCA_023546845.1
GCA_023546865.1
GCA_023546885.1
GCA_023546985.1
GCA_023547005.1
GCA_023547025.1
GCA_023547085.1
GCA_023547105.1
GCA_023547125.1
GCA_023547145.1
GCA_023547225.1
GCA_023547315.1
GCA_023547405.1
GCA_023547485.1
GCA_023556315.2
GCA_023558335.1
GCA_023558355.1
GCA_023558885.1
GCA_023559125.1
GCA_023559145.1
GCA_023559245.1
GCA_023571465.1
GCA_023571485.1
GCA_023572865.1
GCA_023572885.1
GCA_023572905.1
GCA_023572925.1
GCA_023572945.1
GCA_023572965.1

GCA_023572985.1
GCA_023573005.1
GCA_023573025.1
GCA_023573045.1
GCA_023573065.1
GCA_023573085.1
GCA_023573105.1
GCA_023573125.1
GCA_023573145.1
GCA_023573165.1
GCA_023573205.1
GCA_023573225.1
GCA_023573245.1
GCA_023573265.1
GCA_023573285.1
GCA_023573325.1
GCA_023573345.1
GCA_023573505.1
GCA_023573545.1
GCA_023573565.1
GCA_023573585.1
GCA_023573605.1
GCA_023573625.1
GCA_023573645.1
GCA_023573665.1
GCA_023573685.1
GCA_023573705.1
GCA_023573725.1
GCA_023573745.1
GCA_023573765.1
GCA_023573785.1
GCA_023573805.1
GCA_023573825.1
GCA_023573845.1
GCA_023573865.1
GCA_023573885.1
GCA_023585685.1
GCA_023585705.1
GCA_023585725.1
GCA_023585745.1
GCA_023585765.1
GCA_023585785.1
GCA_023585805.1
GCA_023585825.1
GCA_023585845.1
GCA_023585865.1
GCA_023585885.1
GCA_023585905.1
GCA_023585925.1
GCA_023586085.1
GCA_023586245.1
GCA_023586365.1
GCA_023586525.1
GCA_023586645.2

GCA_023586685.2
GCA_023586705.2
GCA_023586725.2
GCA_023586745.3
GCA_023586765.3
GCA_023586785.3
GCA_023586805.1
GCA_023586825.2
GCA_023586845.3
GCA_023586865.1
GCA_023586965.1
GCA_023587085.1
GCA_023587205.1
GCA_023587305.1
GCA_023587465.1
GCA_023587585.1
GCA_023587605.1
GCA_023587625.1
GCA_023587645.1
GCA_023587665.1
GCA_023587685.1
GCA_023587705.1
GCA_023587725.1
GCA_023588825.1
GCA_023588965.1
GCA_023611505.1
GCA_023611705.1
GCA_023611765.1
GCA_023611785.1
GCA_023611805.1
GCA_023611825.1
GCA_023611845.1
GCA_023611865.1
GCA_023611885.1
GCA_023611925.1
GCA_023612035.1
GCA_023612055.1
GCA_023612075.1
GCA_023612115.1
GCA_023612135.1
GCA_023612155.1
GCA_023612175.1
GCA_023612195.1
GCA_023612215.1
GCA_023612255.1
GCA_023612275.1
GCA_023612295.1
GCA_023612315.1
GCA_023612335.1
GCA_023612355.1
GCA_023612395.1
GCA_023612415.1
GCA_023612435.1
GCA_023612455.1

GCA_023612475.1
GCA_023612495.1
GCA_023612615.1
GCA_023612635.1
GCA_023612655.1
GCA_023612675.1
GCA_023612695.1
GCA_023612715.1
GCA_023612735.1
GCA_023612755.1
GCA_023612775.1
GCA_023612795.1
GCA_023612875.1
GCA_023612895.1
GCA_023614235.1
GCA_023614255.1
GCA_023614275.1
GCA_023614295.1
GCA_023614465.1
GCA_023614485.1
GCA_023614505.1
GCA_023614525.1
GCA_023615345.2
GCA_023615705.2
GCA_023615745.2
GCA_023617245.1
GCA_023634805.2
GCA_023639195.1
GCA_023639215.1
GCA_023639785.1
GCA_023650435.1
GCA_023650455.1
GCA_023650475.1
GCA_023650495.1
GCA_023650515.1
GCA_023650615.1
GCA_023650665.1
GCA_023650685.1
GCA_023650735.1
GCA_023650755.1
GCA_023650775.1
GCA_023650795.1
GCA_023650815.1
GCA_023650835.1
GCA_023650855.1
GCA_023650875.1
GCA_023650895.1
GCA_023650915.1
GCA_023650935.1
GCA_023650955.1
GCA_023651955.1
GCA_023653825.1
GCA_023653845.1
GCA_023653865.1

GCA_023653885.1
GCA_023653905.1
GCA_023653925.1
GCA_023657535.1
GCA_023657555.1
GCA_023657575.1
GCA_023657595.1
GCA_023657615.1
GCA_023657635.1
GCA_023657655.1
GCA_023657675.1
GCA_023657695.1
GCA_023657715.1
GCA_023657735.1
GCA_023657795.1
GCA_023657815.1
GCA_023657835.1
GCA_023657855.1
GCA_023657875.1
GCA_023657895.1
GCA_023657915.1
GCA_023657935.1
GCA_023657955.1
GCA_023657975.1
GCA_023657995.1
GCA_023658015.1
GCA_023658035.1
GCA_023658085.1
GCA_023658105.1
GCA_023658125.1
GCA_023658145.1
GCA_023658165.1
GCA_023658185.1
GCA_023658205.1
GCA_023658225.1
GCA_023658245.1
GCA_023658265.1
GCA_023658285.1
GCA_023658305.1
GCA_023658325.1
GCA_023658345.1
GCA_023658365.1
GCA_023658385.1
GCA_023658405.1
GCA_023658425.1
GCA_023658595.1
GCA_023658665.1
GCA_023674385.1
GCA_023674425.1
GCA_023674445.1
GCA_023674465.1
GCA_023674485.1
GCA_023674545.1
GCA_023699965.1

GCA_023700225.1
GCA_023702085.1
GCA_023702105.1
GCA_023702145.1
GCA_023702165.1
GCA_023702495.1
GCA_023702515.1
GCA_023702555.1
GCA_023702575.1
GCA_023702595.1
GCA_023702635.1
GCA_023702655.1
GCA_023702675.1
GCA_023702695.1
GCA_023702715.1
GCA_023702735.1
GCA_023702755.1
GCA_023703515.1
GCA_023703535.1
GCA_023703555.1
GCA_023703575.1
GCA_023703595.1
GCA_023703615.1
GCA_023703635.1
GCA_023703655.1
GCA_023703675.1
GCA_023703755.1
GCA_023703815.1
GCA_023703835.1
GCA_023703855.1
GCA_023703875.1
GCA_023703895.1
GCA_023703915.1
GCA_023703935.1
GCA_023703955.1
GCA_023703975.1
GCA_023703995.1
GCA_023704015.1
GCA_023704035.1
GCA_023704175.1
GCA_023704195.1
GCA_023704215.1
GCA_023704235.1
GCA_023704295.1
GCA_023715965.1
GCA_023715985.1
GCA_023716005.1
GCA_023716445.1
GCA_023716465.1
GCA_023716485.1
GCA_023716525.1
GCA_023716545.1
GCA_023716565.1
GCA_023716585.1

GCA_023716605.1
GCA_023716625.1
GCA_023716645.1
GCA_023716665.1
GCA_023716685.1
GCA_023716705.1
GCA_023716725.1
GCA_023716745.1
GCA_023716765.1
GCA_023716785.1
GCA_023716805.1
GCA_023716825.1
GCA_023716845.1
GCA_023716865.1
GCA_023733635.1
GCA_023734095.1
GCA_023734115.1
GCA_023734135.1
GCA_023734195.1
GCA_023734215.1
GCA_023734235.1
GCA_023734255.1
GCA_023734275.1
GCA_023734295.1
GCA_023734315.1
GCA_023734335.1
GCA_023734355.1
GCA_023734735.1
GCA_023734755.1
GCA_023734775.1
GCA_023734795.1
GCA_023734815.1
GCA_023734835.1
GCA_023734855.1
GCA_023734875.1
GCA_023734895.1
GCA_023734915.1
GCA_023734935.1
GCA_023734955.1
GCA_023734975.1
GCA_023734995.1
GCA_023735015.1
GCA_023735035.1
GCA_023735055.1
GCA_023735075.1
GCA_023735155.1
GCA_023735195.1
GCA_023735215.1
GCA_023735235.1
GCA_023735255.1
GCA_023735275.1
GCA_023735295.1
GCA_023735315.1
GCA_023735335.1

GCA_023735355.1
GCA_023735375.1
GCA_023735395.1
GCA_023735435.1
GCA_023735475.1
GCA_023735495.1
GCA_023735515.1
GCA_023735535.1
GCA_023735615.1
GCA_023735695.1
GCA_023735715.1
GCA_023735735.1
GCA_023735755.1
GCA_023735775.1
GCA_023735795.1
GCA_023735815.1
GCA_023735835.1
GCA_023735855.1
GCA_023735875.1
GCA_023735895.1
GCA_023735915.1
GCA_023735935.1
GCA_023735955.1
GCA_023735975.1
GCA_023735995.1
GCA_023736015.1
GCA_023746535.1
GCA_023746555.1
GCA_023746575.1
GCA_023746615.1
GCA_023746725.1
GCA_023757925.1
GCA_023796775.1
GCA_023796915.1
GCA_023805395.1
GCA_023805415.1
GCA_023805435.1
GCA_023809625.1
GCA_023809705.1
GCA_023809725.1
GCA_023809745.1
GCA_023821965.1
GCA_023821985.2
GCA_023822005.2
GCA_023822025.1
GCA_023822045.1
GCA_023822065.1
GCA_023822085.1
GCA_023822125.1
GCA_023822145.1
GCA_023822165.1
GCA_023822185.1
GCA_023822205.1
GCA_023822225.1

GCA_023822305.1
GCA_023822425.1
GCA_023822445.1
GCA_023822465.1
GCA_023822485.1
GCA_023822665.1
GCA_023822685.1
GCA_023823085.1
GCA_023823105.1
GCA_023823125.1
GCA_023823145.1
GCA_023823715.1
GCA_023823735.1
GCA_023823755.1
GCA_023823775.1
GCA_023823815.1
GCA_023823835.1
GCA_023823855.1
GCA_023823875.1
GCA_023823895.1
GCA_023823915.1
GCA_023823935.1
GCA_023823955.1
GCA_023823975.1
GCA_023823995.1
GCA_023824015.1
GCA_023824035.1
GCA_023824055.1
GCA_023824075.1
GCA_023824095.1
GCA_023824115.1
GCA_023824135.1
GCA_023824155.1
GCA_023824175.1
GCA_023824195.1
GCA_023824315.1
GCA_023845475.1
GCA_023848535.1
GCA_023848615.1
GCA_023848645.1
GCA_023897035.1
GCA_023898405.1
GCA_023898465.1
GCA_023898485.1
GCA_023898505.1
GCA_023898525.1
GCA_023898645.1
GCA_023898665.1
GCA_023898705.1
GCA_023898725.1
GCA_023898745.1
GCA_023898805.1
GCA_023913535.1
GCA_023913595.1

GCA_023920005.1
GCA_023920025.1
GCA_023920045.1
GCA_023920065.1
GCA_023920085.1
GCA_023920105.1
GCA_023920145.1
GCA_023920185.1
GCA_023920205.1
GCA_023920225.1
GCA_023920245.1
GCA_023920325.1
GCA_023920605.1
GCA_023920705.1
GCA_023921085.1
GCA_023921125.1
GCA_023921145.1
GCA_023921165.1
GCA_023921185.1
GCA_023921205.1
GCA_023921225.1
GCA_023921245.1
GCA_023921265.1
GCA_023921285.1
GCA_023921405.1
GCA_023921525.1
GCA_023921665.1
GCA_023921805.1
GCA_023922005.1
GCA_023923085.1
GCA_023923185.1
GCA_023923205.1
GCA_023923225.1
GCA_023923245.1
GCA_023934045.1
GCA_023935105.1
GCA_023935825.1
GCA_023970615.1
GCA_023972895.1
GCA_023973045.1
GCA_023973105.1
GCA_023973125.1
GCA_023973165.1
GCA_023980805.1
GCA_023981045.1
GCA_023983475.1
GCA_023983615.1
GCA_023988885.1
GCA_024022895.1
GCA_024022955.1
GCA_024022975.1
GCA_024023035.1
GCA_024023055.1
GCA_024023075.1

GCA_024029335.1
GCA_024029355.1
GCA_024029435.1
GCA_024029595.1
GCA_024029675.1
GCA_024029775.1
GCA_024029855.1
GCA_024029875.1
GCA_024029895.1
GCA_024029915.1
GCA_024053515.2
GCA_024054255.2
GCA_024055505.2
GCA_024072135.1
GCA_024072215.1
GCA_024072235.1
GCA_024072255.1
GCA_024072275.1
GCA_024072295.1
GCA_024072315.1
GCA_024072335.1
GCA_024072355.1
GCA_024072375.1
GCA_024072395.1
GCA_024072415.1
GCA_024072595.1
GCA_024072715.1
GCA_024081805.1
GCA_024083765.1
GCA_024086695.1
GCA_024089335.1
GCA_024089355.1
GCA_024089375.1
GCA_024089395.1
GCA_024106315.2
GCA_024112375.1
GCA_024112395.1
GCA_024123775.2
GCA_024123855.1
GCA_024123875.1
GCA_024123895.1
GCA_024123915.1
GCA_024123935.1
GCA_024123955.1
GCA_024125655.1
GCA_024125675.1
GCA_024125695.1
GCA_024125715.1
GCA_024125815.1
GCA_024126235.1
GCA_024126255.1
GCA_024126275.1
GCA_024126295.1
GCA_024126315.1

GCA_024126335.1
GCA_024126355.1
GCA_024134505.1
GCA_024134525.1
GCA_024134545.1
GCA_024134625.1
GCA_024134645.1
GCA_024134665.1
GCA_024134685.1
GCA_024137845.1
GCA_024137865.1
GCA_024137905.1
GCA_024137925.1
GCA_024137945.1
GCA_024137965.1
GCA_024137985.1
GCA_024138075.1
GCA_024138105.1
GCA_024138415.1
GCA_024138435.1
GCA_024138555.1
GCA_024138575.1
GCA_024138675.1
GCA_024138695.1
GCA_024138715.1
GCA_024138735.1
GCA_024138755.1
GCA_024138795.1
GCA_024138815.1
GCA_024138835.1
GCA_024138855.1
GCA_024138895.1
GCA_024138975.1
GCA_024138995.1
GCA_024139015.1
GCA_024139035.1
GCA_024139055.1
GCA_024139075.1
GCA_024139095.1
GCA_024158105.2
GCA_024158965.2
GCA_024172225.1
GCA_024172245.1
GCA_024172265.1
GCA_024172285.1
GCA_024172305.1
GCA_024172325.1
GCA_024172345.1
GCA_024172365.1
GCA_024172385.1
GCA_024172405.1
GCA_024172425.1
GCA_024172445.1
GCA_024172525.1

GCA_024172545.1
GCA_024172585.1
GCA_024172605.1
GCA_024172625.1
GCA_024172645.1
GCA_024172665.1
GCA_024172705.1
GCA_024181005.1
GCA_024181025.1
GCA_024181045.1
GCA_024181065.1
GCA_024181085.1
GCA_024181105.1
GCA_024181125.1
GCA_024181145.1
GCA_024181165.1
GCA_024181185.1
GCA_024181205.1
GCA_024181225.1
GCA_024181245.1
GCA_024181265.1
GCA_024181285.1
GCA_024181305.1
GCA_024181325.1
GCA_024181345.1
GCA_024181365.1
GCA_024181385.1
GCA_024181405.1
GCA_024181425.1
GCA_024181445.1
GCA_024181465.1
GCA_024181485.1
GCA_024181505.1
GCA_024181545.1
GCA_024181565.1
GCA_024181585.1
GCA_024181605.1
GCA_024181625.1
GCA_024181645.1
GCA_024181665.1
GCA_024181685.1
GCA_024181705.1
GCA_024181725.1
GCA_024181745.1
GCA_024181765.1
GCA_024182005.1
GCA_024182025.1
GCA_024182575.1
GCA_024195245.1
GCA_024204625.1
GCA_024204645.1
GCA_024204685.1
GCA_024204705.1
GCA_024204725.1

GCA_024204745.1
GCA_024204765.1
GCA_024204785.1
GCA_024204805.1
GCA_024204825.1
GCA_024204845.1
GCA_024204865.1
GCA_024204885.1
GCA_024204905.1
GCA_024204925.1
GCA_024204945.1
GCA_024204965.1
GCA_024204985.1
GCA_024205025.1
GCA_024205045.1
GCA_024205065.1
GCA_024205085.1
GCA_024205105.1
GCA_024205125.1
GCA_024205145.1
GCA_024205165.1
GCA_024205185.1
GCA_024205205.1
GCA_024205225.1
GCA_024205245.1
GCA_024205265.1
GCA_024205285.1
GCA_024205305.1
GCA_024205325.1
GCA_024205425.1
GCA_024205445.1
GCA_024205465.1
GCA_024205485.1
GCA_024205505.1
GCA_024205525.1
GCA_024205545.1
GCA_024205565.1
GCA_024205585.1
GCA_024205605.1
GCA_024205625.1
GCA_024205645.1
GCA_024205665.1
GCA_024205685.1
GCA_024205705.1
GCA_024205725.1
GCA_024205745.1
GCA_024205765.1
GCA_024205785.1
GCA_024205805.1
GCA_024205825.1
GCA_024205845.1
GCA_024205865.1
GCA_024205885.1
GCA_024205905.1

GCA_024205925.1
GCA_024205945.1
GCA_024205965.1
GCA_024206005.1
GCA_024206595.1
GCA_024206615.1
GCA_024206635.1
GCA_024206655.1
GCA_024206675.1
GCA_024206695.1
GCA_024206715.1
GCA_024206735.1
GCA_024206755.1
GCA_024206775.1
GCA_024206795.1
GCA_024207115.1
GCA_024218535.1
GCA_024218715.1
GCA_024218735.1
GCA_024218755.1
GCA_024218835.1
GCA_024218855.1
GCA_024219955.1
GCA_024220035.1
GCA_024220055.1
GCA_024220075.1
GCA_024220095.1
GCA_024220115.1
GCA_024220135.1
GCA_024220155.1
GCA_024220175.1
GCA_024223055.1
GCA_024223415.1
GCA_024223795.1
GCA_024224055.1
GCA_024224355.1
GCA_024224615.1
GCA_024224875.1
GCA_024225115.1
GCA_024225435.1
GCA_024225755.1
GCA_024226085.1
GCA_024226435.1
GCA_024226755.1
GCA_024227055.1
GCA_024266125.1
GCA_024266145.1
GCA_024266165.1
GCA_024266185.1
GCA_024266205.1
GCA_024266225.1
GCA_024266245.1
GCA_024266265.1
GCA_024266285.1

GCA_024266305.1
GCA_024266345.1
GCA_024266365.1
GCA_024266385.1
GCA_024266405.1
GCA_024266425.1
GCA_024266445.1
GCA_024266465.1
GCA_024266505.1
GCA_024266525.1
GCA_024266545.1
GCA_024266565.1
GCA_024266585.1
GCA_024266605.1
GCA_024266625.1
GCA_024266645.1
GCA_024266665.1
GCA_024266685.1
GCA_024266725.1
GCA_024266785.1
GCA_024266805.1
GCA_024266825.1
GCA_024266875.1
GCA_024266895.1
GCA_024266915.1
GCA_024266935.1
GCA_024266955.1
GCA_024267595.1
GCA_024267655.1
GCA_024271825.1
GCA_024271895.1
GCA_024296785.1
GCA_024296805.1
GCA_024296825.1
GCA_024296845.1
GCA_024296865.1
GCA_024296885.1
GCA_024296905.1
GCA_024296925.1
GCA_024296945.1
GCA_024296965.1
GCA_024296985.1
GCA_024297005.1
GCA_024297025.1
GCA_024297045.1
GCA_024297065.1
GCA_024297105.1
GCA_024297125.1
GCA_024298465.1
GCA_024298485.1
GCA_024298505.1
GCA_024298525.1
GCA_024298545.1
GCA_024298565.1

GCA_024298585.1
GCA_024298605.1
GCA_024298625.1
GCA_024298645.1
GCA_024298665.1
GCA_024298685.1
GCA_024298705.1
GCA_024298725.1
GCA_024298745.1
GCA_024298765.1
GCA_024298785.1
GCA_024298805.1
GCA_024298925.1
GCA_024298945.1
GCA_024298965.1
GCA_024298985.1
GCA_024299005.1
GCA_024299025.1
GCA_024299105.1
GCA_024299245.1
GCA_024300645.1
GCA_024300685.1
GCA_024300705.1
GCA_024300765.1
GCA_024300785.1
GCA_024300805.1
GCA_024300845.1
GCA_024321955.1
GCA_024328725.2
GCA_024329775.2
GCA_024329785.2
GCA_024330025.2
GCA_024330145.2
GCA_024331345.2
GCA_024331375.2
GCA_024331425.2
GCA_024331445.2
GCA_024331475.2
GCA_024331495.2
GCA_024331525.2
GCA_024331535.2
GCA_024331585.2
GCA_024333985.1
GCA_024334005.1
GCA_024334025.1
GCA_024334045.1
GCA_024334065.1
GCA_024338845.1
GCA_024339025.1
GCA_024339125.1
GCA_024346585.1
GCA_024346605.1
GCA_024346655.1
GCA_024346675.1

GCA_024346695.1
GCA_024346715.1
GCA_024346735.1
GCA_024346755.1
GCA_024346775.1
GCA_024346795.1
GCA_024346815.1
GCA_024346855.1
GCA_024346875.1
GCA_024346895.1
GCA_024346915.1
GCA_024346955.1
GCA_024346975.1
GCA_024346995.1
GCA_024347035.1
GCA_024347055.1
GCA_024347075.1
GCA_024347095.1
GCA_024347115.1
GCA_024347135.1
GCA_024347155.1
GCA_024347175.1
GCA_024347195.1
GCA_024347215.1
GCA_024347235.1
GCA_024347255.1
GCA_024347275.1
GCA_024347295.1
GCA_024347315.1
GCA_024347335.1
GCA_024347355.1
GCA_024347375.1
GCA_024347395.1
GCA_024347415.1
GCA_024347435.1
GCA_024347455.1
GCA_024347475.1
GCA_024347495.1
GCA_024347515.1
GCA_024347535.1
GCA_024347555.1
GCA_024347575.1
GCA_024347595.1
GCA_024347615.1
GCA_024347635.1
GCA_024347655.1
GCA_024347675.1
GCA_024347695.1
GCA_024347715.1
GCA_024347755.1
GCA_024347775.1
GCA_024347795.1
GCA_024347815.1
GCA_024347835.1

GCA_024347855.1
GCA_024349685.1
GCA_024349705.1
GCA_024349725.1
GCA_024349745.1
GCA_024349765.1
GCA_024349785.1
GCA_024349805.1
GCA_024360925.2
GCA_024362185.1
GCA_024362205.1
GCA_024362225.1
GCA_024362245.1
GCA_024362265.1
GCA_024362285.1
GCA_024362325.1
GCA_024362345.1
GCA_024362365.1
GCA_024362385.1
GCA_024362715.1
GCA_024362765.1
GCA_024362785.1
GCA_024362805.1
GCA_024362845.1
GCA_024363255.1
GCA_024363285.1
GCA_024363305.1
GCA_024363325.1
GCA_024363345.1
GCA_024363365.1
GCA_024363385.1
GCA_024363445.1
GCA_024363465.1
GCA_024363485.1
GCA_024363505.1
GCA_024363545.1
GCA_024364825.1
GCA_024364845.1
GCA_024364865.1
GCA_024364885.1
GCA_024364905.1
GCA_024364925.1
GCA_024364945.1
GCA_024364965.1
GCA_024364985.1
GCA_024365005.1
GCA_024365025.1
GCA_024365045.1
GCA_024371385.1
GCA_024372495.1
GCA_024380035.1
GCA_024380055.1
GCA_024381035.1
GCA_024396435.1

GCA_024396455.1
GCA_024396535.1
GCA_024396555.1
GCA_024396575.1
GCA_024396595.1
GCA_024396695.1
GCA_024396775.1
GCA_024396795.1
GCA_024396815.1
GCA_024396855.1
GCA_024396875.1
GCA_024396895.1
GCA_024397075.1
GCA_024397235.1
GCA_024397275.1
GCA_024397295.1
GCA_024397315.1
GCA_024397335.1
GCA_024397355.1
GCA_024397375.1
GCA_024397395.1
GCA_024397415.1
GCA_024397455.1
GCA_024397675.1
GCA_024397795.1
GCA_024397855.1
GCA_024397895.1
GCA_024397915.1
GCA_024397935.1
GCA_024397955.1
GCA_024397975.1
GCA_024397995.1
GCA_024398015.1
GCA_024398035.1
GCA_024398055.1
GCA_024398135.1
GCA_024398395.1
GCA_024398575.1
GCA_024398735.1
GCA_024398905.1
GCA_024399055.1
GCA_024399315.1
GCA_024399335.1
GCA_024399355.1
GCA_024399375.1
GCA_024399395.1
GCA_024399415.1
GCA_024399435.1
GCA_024399455.1
GCA_024399595.1
GCA_024399875.1
GCA_024400215.1
GCA_024400375.1
GCA_024400535.1

GCA_024400725.1
GCA_024400855.1
GCA_024401005.1
GCA_024401155.1
GCA_024408125.1
GCA_024426085.1
GCA_024426665.1
GCA_024426685.1
GCA_024442115.1
GCA_024442155.1
GCA_024442175.1
GCA_024442195.1
GCA_024442215.1
GCA_024442235.1
GCA_024442255.1
GCA_024442275.1
GCA_024442295.1
GCA_024442315.1
GCA_024442335.1
GCA_024448255.1
GCA_024449095.1
GCA_024452365.1
GCA_024453785.1
GCA_024453815.1
GCA_024453835.1
GCA_024466835.1
GCA_024467035.1
GCA_024494485.1
GCA_024494505.1
GCA_024494525.1
GCA_024494545.1
GCA_024496025.1
GCA_024496085.1
GCA_024496105.1
GCA_024496125.1
GCA_024496145.1
GCA_024496165.1
GCA_024496185.1
GCA_024496205.1
GCA_024496225.1
GCA_024496245.1
GCA_024496265.1
GCA_024496285.1
GCA_024498155.1
GCA_024498215.1
GCA_024498235.1
GCA_024498255.1
GCA_024498275.1
GCA_024498295.1
GCA_024498315.1
GCA_024498355.1
GCA_024498375.1
GCA_024498395.1
GCA_024498415.1

GCA_024498575.1
GCA_024498615.1
GCA_024498635.1
GCA_024498655.1
GCA_024498675.1
GCA_024498695.1
GCA_024498715.1
GCA_024498735.1
GCA_024498755.1
GCA_024498775.1
GCA_024498795.1
GCA_024498815.1
GCA_024498835.1
GCA_024498855.1
GCA_024498875.1
GCA_024498895.1
GCA_024498915.1
GCA_024498935.1
GCA_024498955.1
GCA_024498995.1
GCA_024499015.1
GCA_024499055.1
GCA_024499075.1
GCA_024499095.1
GCA_024499115.1
GCA_024499135.1
GCA_024499155.1
GCA_024499175.1
GCA_024499195.1
GCA_024499245.1
GCA_024499265.1
GCA_024499285.1
GCA_024499305.1
GCA_024499525.1
GCA_024499545.1
GCA_024505625.1
GCA_024505665.1
GCA_024507815.1
GCA_024507835.1
GCA_024507855.1
GCA_024507875.1
GCA_024507895.1
GCA_024507915.1
GCA_024507935.1
GCA_024507955.1
GCA_024507975.1
GCA_024507995.1
GCA_024508015.1
GCA_024508035.1
GCA_024508055.1
GCA_024508075.1
GCA_024508095.1
GCA_024508115.1
GCA_024508135.1

GCA_024508155.1
GCA_024508175.1
GCA_024508195.1
GCA_024508215.1
GCA_024508255.1
GCA_024508275.1
GCA_024508295.1
GCA_024508315.1
GCA_024508335.1
GCA_024509915.1
GCA_024510645.1
GCA_024526015.1
GCA_024526695.1
GCA_024529955.1
GCA_024532035.1
GCA_024532055.1
GCA_024532075.1
GCA_024532095.1
GCA_024532115.1
GCA_024532135.1
GCA_024532155.1
GCA_024532175.1
GCA_024532195.1
GCA_024532225.1
GCA_024532245.1
GCA_024532265.1
GCA_024532305.1
GCA_024532325.1
GCA_024532345.1
GCA_024532365.1
GCA_024532385.1
GCA_024532405.1
GCA_024532425.1
GCA_024579755.1
GCA_024579775.1
GCA_024579795.1
GCA_024579815.1
GCA_024579835.1
GCA_024582675.1
GCA_024582695.1
GCA_024582715.1
GCA_024582735.1
GCA_024582755.1
GCA_024582775.1
GCA_024582795.1
GCA_024582815.1
GCA_024582835.1
GCA_024582875.1
GCA_024582915.1
GCA_024582935.1
GCA_024582955.1
GCA_024582975.1
GCA_024582995.1
GCA_024583015.1

GCA_024583035.1
GCA_024583055.1
GCA_024583075.1
GCA_024583095.1
GCA_024583115.1
GCA_024583135.1
GCA_024583155.1
GCA_024583175.1
GCA_024584435.1
GCA_024584455.1
GCA_024584475.1
GCA_024584495.1
GCA_024584515.1
GCA_024584565.1
GCA_024584585.1
GCA_024584605.1
GCA_024584625.1
GCA_024584645.1
GCA_024584665.1
GCA_024584725.1
GCA_024584745.1
GCA_024584785.1
GCA_024584825.1
GCA_024584845.1
GCA_024584865.1
GCA_024584885.1
GCA_024584905.1
GCA_024584925.1
GCA_024584945.1
GCA_024584965.1
GCA_024584985.1
GCA_024585005.1
GCA_024585025.1
GCA_024585045.1
GCA_024585285.1
GCA_024585325.1
GCA_024585365.1
GCA_024585385.1
GCA_024585405.1
GCA_024585425.1
GCA_024585445.1
GCA_024585465.1
GCA_024585485.1
GCA_024585505.1
GCA_024585525.1
GCA_024585545.1
GCA_024585565.1
GCA_024585585.1
GCA_024585625.1
GCA_024585665.1
GCA_024585685.1
GCA_024585705.1
GCA_024585725.1
GCA_024585745.1

GCA_024585765.1
GCA_024585785.1
GCA_024585805.1
GCA_024585825.1
GCA_024585845.1
GCA_024585865.1
GCA_024585885.1
GCA_024585905.1
GCA_024585925.1
GCA_024585945.1
GCA_024585965.1
GCA_024585985.1
GCA_024599655.1
GCA_024599795.1
GCA_024599915.1
GCA_024599935.1
GCA_024599955.1
GCA_024599975.1
GCA_024599995.1
GCA_024600015.1
GCA_024600035.1
GCA_024600055.1
GCA_024600175.1
GCA_024600315.1
GCA_024600455.1
GCA_024600575.1
GCA_024600715.1
GCA_024605785.1
GCA_024605895.1
GCA_024606005.1
GCA_024610595.1
GCA_024610615.1
GCA_024610635.1
GCA_024610675.1
GCA_024610975.1
GCA_024611115.1
GCA_024611135.1
GCA_024611995.1
GCA_024612015.1
GCA_024612035.1
GCA_024612055.1
GCA_024628725.1
GCA_024628805.1
GCA_024628825.1
GCA_024628845.1
GCA_024628865.1
GCA_024628885.1
GCA_024637875.1
GCA_024637895.1
GCA_024637915.1
GCA_024637935.1
GCA_024637955.1
GCA_024637975.1
GCA_024637995.1

GCA_024638015.1
GCA_024638035.1
GCA_024652825.1
GCA_024652845.1
GCA_024652865.1
GCA_024652885.1
GCA_024652905.1
GCA_024652925.1
GCA_024652945.3
GCA_024661895.1
GCA_024661975.1
GCA_024661995.1
GCA_024662015.1
GCA_024662035.1
GCA_024662055.1
GCA_024662075.1
GCA_024662135.1
GCA_024662155.1
GCA_024662175.1
GCA_024662195.1
GCA_024662235.1
GCA_024662255.1
GCA_024662315.1
GCA_024662335.1
GCA_024662355.1
GCA_024662375.1
GCA_024662395.1
GCA_024662415.1
GCA_024662435.1
GCA_024662455.1
GCA_024662475.1
GCA_024662615.1
GCA_024662735.1
GCA_024662895.1
GCA_024663015.1
GCA_024663135.1
GCA_024663255.1
GCA_024663375.1
GCA_024663615.1
GCA_024663775.1
GCA_024663895.1
GCA_024663975.1
GCA_024663995.1
GCA_024664015.1
GCA_024664035.1
GCA_024664055.1
GCA_024664075.1
GCA_024664095.1
GCA_024664115.1
GCA_024664135.1
GCA_024664155.1
GCA_024664175.1
GCA_024664195.1
GCA_024664215.1

GCA_024664235.1
GCA_024664255.1
GCA_024664275.1
GCA_024665075.1
GCA_024665195.1
GCA_024665315.1
GCA_024665435.1
GCA_024665555.1
GCA_024665595.1
GCA_024665615.1
GCA_024665635.1
GCA_024665655.1
GCA_024665675.1
GCA_024665815.1
GCA_024665995.1
GCA_024666265.1
GCA_024666385.1
GCA_024666505.1
GCA_024700745.2
GCA_024700815.2
GCA_024700835.2
GCA_024700875.2
GCA_024700885.2
GCA_024701375.2
GCA_024703115.1
GCA_024703815.1
GCA_024703835.1
GCA_024703855.1
GCA_024703875.1
GCA_024703895.1
GCA_024703915.1
GCA_024703935.1
GCA_024703955.1
GCA_024704525.1
GCA_024704545.1
GCA_024706255.1
GCA_024707505.1
GCA_024707525.1
GCA_024707545.1
GCA_024717335.1
GCA_024721175.1
GCA_024721545.1
GCA_024721565.1
GCA_024722255.1
GCA_024722275.1
GCA_024722295.1
GCA_024722315.1
GCA_024722355.1
GCA_024722375.1
GCA_024722395.1
GCA_024722415.1
GCA_024722435.1
GCA_024722455.1
GCA_024722475.1

GCA_024722495.1
GCA_024722515.1
GCA_024723375.2
GCA_024730565.1
GCA_024730645.1
GCA_024730665.1
GCA_024730685.1
GCA_024730705.1
GCA_024730725.1
GCA_024730745.1
GCA_024730765.1
GCA_024730785.1
GCA_024730805.1
GCA_024730825.1
GCA_024730945.1
GCA_024731085.1
GCA_024731205.1
GCA_024731345.1
GCA_024731685.1
GCA_024732225.1
GCA_024732285.1
GCA_024732305.1
GCA_024732325.1
GCA_024732345.1
GCA_024732365.1
GCA_024732385.1
GCA_024732405.1
GCA_024732425.1
GCA_024734075.1
GCA_024734095.1
GCA_024734115.1
GCA_024734135.1
GCA_024734155.1
GCA_024734175.1
GCA_024734195.1
GCA_024734215.1
GCA_024734235.1
GCA_024734255.1
GCA_024734275.1
GCA_024734295.1
GCA_024734405.1
GCA_024734625.1
GCA_024734645.1
GCA_024734665.1
GCA_024734685.1
GCA_024734705.1
GCA_024734725.1
GCA_024734755.1
GCA_024734775.1
GCA_024734795.1
GCA_024734815.1
GCA_024734835.1
GCA_024734895.1
GCA_024735225.1

GCA_024735245.1
GCA_024741355.1
GCA_024741375.1
GCA_024741395.1
GCA_024741415.1
GCA_024741435.1
GCA_024741455.1
GCA_024741475.1
GCA_024741495.1
GCA_024741515.1
GCA_024741535.1
GCA_024741555.1
GCA_024741575.1
GCA_024741595.1
GCA_024741615.1
GCA_024741635.1
GCA_024741655.1
GCA_024741675.1
GCA_024741695.1
GCA_024741715.1
GCA_024741735.1
GCA_024741755.1
GCA_024741775.1
GCA_024741795.1
GCA_024741815.1
GCA_024741855.1
GCA_024746795.1
GCA_024746815.1
GCA_024746835.1
GCA_024746855.1
GCA_024748535.1
GCA_024748555.1
GCA_024748575.1
GCA_024748595.1
GCA_024748615.1
GCA_024748635.1
GCA_024748655.1
GCA_024748675.1
GCA_024748695.1
GCA_024748715.1
GCA_024748735.1
GCA_024748755.1
GCA_024748775.1
GCA_024748795.1
GCA_024748815.1
GCA_024748895.1
GCA_024748925.1
GCA_024748945.1
GCA_024748965.1
GCA_024748985.1
GCA_024749005.1
GCA_024749025.1
GCA_024749045.1
GCA_024749065.1

GCA_024749085.1
GCA_024749105.1
GCA_024749125.1
GCA_024749145.1
GCA_024749165.1
GCA_024749185.1
GCA_024749205.1
GCA_024749225.1
GCA_024749245.1
GCA_024749265.1
GCA_024749285.1
GCA_024749305.1
GCA_024749325.1
GCA_024749345.1
GCA_024749365.1
GCA_024749385.1
GCA_024749405.1
GCA_024749425.1
GCA_024749445.1
GCA_024749465.1
GCA_024749485.1
GCA_024749505.1
GCA_024749525.1
GCA_024749545.1
GCA_024749605.1
GCA_024749625.1
GCA_024749645.1
GCA_024749665.1
GCA_024749685.1
GCA_024749745.1
GCA_024749765.1
GCA_024750555.1
GCA_024750575.1
GCA_024752495.1
GCA_024752515.1
GCA_024752535.1
GCA_024752555.1
GCA_024757965.1
GCA_024757985.1
GCA_024758005.1
GCA_024758045.1
GCA_024758065.1
GCA_024758085.1
GCA_024758165.1
GCA_024758205.1
GCA_024758225.1
GCA_024758245.1
GCA_024758345.1
GCA_024758365.1
GCA_024758405.1
GCA_024758425.1
GCA_024758445.1
GCA_024758505.1
GCA_024758565.1

GCA_024758585.1
GCA_024758605.1
GCA_024758625.1
GCA_024758645.1
GCA_024758665.1
GCA_024758685.1
GCA_024758705.1
GCA_024758725.1
GCA_024758745.1
GCA_024758765.1
GCA_024758785.1
GCA_024758805.1
GCA_024758825.1
GCA_024758865.1
GCA_024758885.1
GCA_024758905.1
GCA_024759085.1
GCA_024759125.1
GCA_024759185.1
GCA_024759205.1
GCA_024759225.1
GCA_024759245.1
GCA_024759265.1
GCA_024759285.1
GCA_024759345.1
GCA_024759365.1
GCA_024759385.1
GCA_024759405.1
GCA_024759425.1
GCA_024759485.1
GCA_024759505.1
GCA_024759525.1
GCA_024759545.1
GCA_024759565.1
GCA_024759585.1
GCA_024759625.1
GCA_024759645.1
GCA_024759745.1
GCA_024759765.1
GCA_024759785.1
GCA_024759845.1
GCA_024759865.1
GCA_024759965.1
GCA_024759985.1
GCA_024760045.1
GCA_024760065.1
GCA_024760085.1
GCA_024760125.1
GCA_024760145.1
GCA_024760205.1
GCA_024760225.1
GCA_024760245.1
GCA_024760285.1
GCA_024760305.1

GCA_024760325.1
GCA_024760445.1
GCA_024760465.1
GCA_024760565.1
GCA_024760585.1
GCA_024760605.1
GCA_024760625.1
GCA_024760645.1
GCA_024762135.1
GCA_024762155.1
GCA_024762175.1
GCA_024762195.1
GCA_024799685.1
GCA_024799725.1
GCA_024799785.1
GCA_024799805.1
GCA_024799845.1
GCA_024799865.1
GCA_024799885.1
GCA_024800105.1
GCA_024800505.1
GCA_024800585.1
GCA_024800605.1
GCA_024800625.1
GCA_024800645.1
GCA_024800665.1
GCA_024800685.1
GCA_024800705.1
GCA_024802385.1
GCA_024802585.1
GCA_024802605.1
GCA_024802625.1
GCA_024803445.1
GCA_024803465.1
GCA_024803545.1
GCA_024803565.1
GCA_024803585.1
GCA_024803605.1
GCA_024803805.1
GCA_024804185.1
GCA_024805795.2
GCA_024817015.1
GCA_024817035.1
GCA_024817055.1
GCA_024817075.1
GCA_024817095.1
GCA_024817115.1
GCA_024817135.1
GCA_024817155.1
GCA_024817175.1
GCA_024817195.1
GCA_024817215.1
GCA_024817235.1
GCA_024817335.1

GCA_024817475.1
GCA_024817655.1
GCA_024817955.1
GCA_024817975.1
GCA_024817995.1
GCA_024818015.1
GCA_024818035.1
GCA_024818095.1
GCA_024818115.1
GCA_024818135.1
GCA_024818515.1
GCA_024818655.1
GCA_024820095.1
GCA_024820175.1
GCA_024832785.2
GCA_024860185.1
GCA_024917115.1
GCA_024917135.1
GCA_024917155.1
GCA_024917175.1
GCA_024917195.1
GCA_024917215.1
GCA_024917235.1
GCA_024917255.1
GCA_024917275.1
GCA_024917295.1
GCA_024917315.1
GCA_024917335.1
GCA_024917355.1
GCA_024917375.1
GCA_024917395.1
GCA_024917415.1
GCA_024917435.1
GCA_024917455.1
GCA_024917475.1
GCA_024917495.1
GCA_024917515.1
GCA_024917535.1
GCA_024917555.1
GCA_024917575.1
GCA_024917595.1
GCA_024917615.1
GCA_024917635.1
GCA_024917655.1
GCA_024917675.1
GCA_024917695.1
GCA_024917715.1
GCA_024917735.1
GCA_024917755.1
GCA_024917775.1
GCA_024917795.1
GCA_024917815.1
GCA_024917835.1
GCA_024917855.1

GCA_024917875.1
GCA_024917895.1
GCA_024917915.1
GCA_024917935.1
GCA_024917955.1
GCA_024917975.1
GCA_024917995.1
GCA_024918015.1
GCA_024918035.1
GCA_024918055.1
GCA_024918075.1
GCA_024918095.1
GCA_024918115.1
GCA_024918135.1
GCA_024918155.1
GCA_024918175.1
GCA_024918195.1
GCA_024918215.1
GCA_024918235.1
GCA_024918255.1
GCA_024918275.1
GCA_024918295.1
GCA_024918315.1
GCA_024918335.1
GCA_024918375.1
GCA_024918395.1
GCA_024918415.1
GCA_024918435.1
GCA_024918455.1
GCA_024918475.1
GCA_024918715.1
GCA_024918755.1
GCA_024918775.1
GCA_024918795.1
GCA_024918815.1
GCA_024918835.1
GCA_024918855.1
GCA_024918875.1
GCA_024918895.1
GCA_024918915.1
GCA_024918935.1
GCA_024918955.1
GCA_024918975.1
GCA_024918995.1
GCA_024919015.1
GCA_024919035.1
GCA_024919055.1
GCA_024919075.1
GCA_024919095.1
GCA_024919115.1
GCA_024919195.1
GCA_024919215.1
GCA_024919235.1
GCA_024919255.1

GCA_024922185.2
GCA_024925305.1
GCA_024925425.1
GCA_024925445.1
GCA_024925465.1
GCA_024925485.1
GCA_024925505.1
GCA_024927865.1
GCA_024927925.1
GCA_024927945.1
GCA_024927965.1
GCA_024930265.1
GCA_024968165.1
GCA_024968185.1
GCA_024968205.1
GCA_024968225.1
GCA_024968245.1
GCA_024968265.1
GCA_024968285.1
GCA_024968305.1
GCA_024968325.1
GCA_024968345.1
GCA_024968365.1
GCA_024968385.1
GCA_024968405.1
GCA_024968525.1
GCA_024968705.1
GCA_024968865.1
GCA_024969025.1
GCA_024969205.1
GCA_024969365.1
GCA_024969385.1
GCA_024969405.1
GCA_024969425.1
GCA_024969445.1
GCA_024969465.1
GCA_024969485.1
GCA_024969505.1
GCA_024969525.1
GCA_024969545.1
GCA_024969565.1
GCA_024969715.1
GCA_024969905.1
GCA_024970065.1
GCA_024970125.1
GCA_024970145.1
GCA_024970165.1
GCA_024970185.1
GCA_024970245.1
GCA_024970305.1
GCA_024970325.1
GCA_024970345.1
GCA_024970535.1
GCA_024970695.1

GCA_024970895.1
GCA_024971055.1
GCA_024971095.1
GCA_024971755.1
GCA_024971795.1
GCA_024971815.1
GCA_024971835.1
GCA_024971855.1
GCA_024971875.1
GCA_024971895.1
GCA_024971915.1
GCA_024971935.1
GCA_024971955.1
GCA_024971975.1
GCA_024971995.1
GCA_024972115.1
GCA_024972275.1
GCA_024972415.1
GCA_024972555.1
GCA_024972795.1
GCA_024972815.1
GCA_024972835.1
GCA_024972855.1
GCA_024972975.1
GCA_024981335.1
GCA_025021485.1
GCA_025021505.1
GCA_025021565.1
GCA_025021745.1
GCA_025021925.1
GCA_025035865.1
GCA_025079255.1
GCA_025118245.1
GCA_025118435.1
GCA_025118455.1
GCA_025133195.1
GCA_025133575.1
GCA_025135795.1
GCA_025135955.1
GCA_025136135.1
GCA_025136295.1
GCA_025136435.1
GCA_025137375.1
GCA_025137495.1
GCA_025137635.1
GCA_025137775.1
GCA_025137935.1
GCA_025138055.1
GCA_025138255.1
GCA_025138375.1
GCA_025138535.1
GCA_025138695.1
GCA_025138865.1
GCA_025139025.1

GCA_025139165.1
GCA_025139345.1
GCA_025139485.1
GCA_025139665.1
GCA_025139835.1
GCA_025139955.1
GCA_025140155.1
GCA_025140295.1
GCA_025140475.1
GCA_025140615.1
GCA_025140835.1
GCA_025141015.1
GCA_025141175.1
GCA_025141335.1
GCA_025141525.1
GCA_025141675.1
GCA_025141835.1
GCA_025141855.1
GCA_025141875.1
GCA_025141935.1
GCA_025142135.1
GCA_025142275.1
GCA_025142455.1
GCA_025142595.1
GCA_025142675.1
GCA_025142695.1
GCA_025142715.1
GCA_025142735.1
GCA_025142755.1
GCA_025142775.1
GCA_025142795.1
GCA_025142815.1
GCA_025142835.1
GCA_025142855.1
GCA_025143105.1
GCA_025143215.1
GCA_025143325.1
GCA_025143405.1
GCA_025143505.1
GCA_025143665.1
GCA_025143825.1
GCA_025143985.1
GCA_025144145.1
GCA_025144335.1
GCA_025144465.1
GCA_025144505.1
GCA_025144525.1
GCA_025144545.1
GCA_025144665.1
GCA_025144995.1
GCA_025145285.1
GCA_025145645.1
GCA_025145845.1
GCA_025146005.1

GCA_025146135.1
GCA_025146315.1
GCA_025146415.1
GCA_025146565.1
GCA_025146775.1
GCA_025147085.1
GCA_025147325.1
GCA_025147485.1
GCA_025147655.1
GCA_025147765.1
GCA_025147905.1
GCA_025148125.1
GCA_025148285.1
GCA_025148445.1
GCA_025148635.1
GCA_025148785.1
GCA_025149125.1
GCA_025149285.1
GCA_025149465.1
GCA_025149625.1
GCA_025149785.1
GCA_025149915.1
GCA_025150085.1
GCA_025150245.1
GCA_025150425.1
GCA_025150565.1
GCA_025150745.1
GCA_025150895.1
GCA_025151045.1
GCA_025151215.1
GCA_025151385.1
GCA_025151535.1
GCA_025151715.1
GCA_025151995.1
GCA_025152275.1
GCA_025152405.1
GCA_025152575.1
GCA_025152605.1
GCA_025152625.1
GCA_025152645.1
GCA_025152665.1
GCA_025152685.1
GCA_025152705.1
GCA_025152725.1
GCA_025152745.1
GCA_025152765.1
GCA_025158995.1
GCA_025159015.1
GCA_025159055.1
GCA_025159075.1
GCA_025159115.1
GCA_025161015.1
GCA_025163535.1
GCA_025163575.1

GCA_025169475.1
GCA_025169495.2
GCA_025169515.2
GCA_025169565.2
GCA_025169585.1
GCA_025169605.2
GCA_025169685.2
GCA_025169745.2
GCA_025169825.2
GCA_025170155.2
GCA_025170325.2
GCA_025170505.2
GCA_025170685.2
GCA_025170705.2
GCA_025170725.2
GCA_025170885.2
GCA_025171075.1
GCA_025171215.2
GCA_025171275.1
GCA_025171295.2
GCA_025171315.2
GCA_025200555.1
GCA_025200575.1
GCA_025200595.1
GCA_025200615.1
GCA_025200635.1
GCA_025200655.1
GCA_025200675.1
GCA_025200695.1
GCA_025200715.1
GCA_025200735.1
GCA_025200775.1
GCA_025200825.1
GCA_025200845.1
GCA_025200865.1
GCA_025200885.1
GCA_025200905.1
GCA_025200925.1
GCA_025200945.1
GCA_025200965.1
GCA_025201235.1
GCA_025213355.1
GCA_025215495.1
GCA_025215645.1
GCA_025215665.1
GCA_025231465.1
GCA_025231525.1
GCA_025232045.1
GCA_025232125.1
GCA_025232205.1
GCA_025232235.1
GCA_025232255.1
GCA_025232275.1
GCA_025232295.1

GCA_025232315.1
GCA_025232335.1
GCA_025232355.1
GCA_025232395.1
GCA_025232415.1
GCA_025232435.1
GCA_025232455.1
GCA_025232475.1
GCA_025232495.1
GCA_025232515.1
GCA_025232535.1
GCA_025232555.1
GCA_025232575.1
GCA_025232595.1
GCA_025232615.1
GCA_025232635.1
GCA_025232655.1
GCA_025232675.1
GCA_025232695.1
GCA_025244625.1
GCA_025244645.1
GCA_025244665.1
GCA_025244685.1
GCA_025244705.1
GCA_025244725.1
GCA_025244745.1
GCA_025244765.1
GCA_025244785.1
GCA_025244805.1
GCA_025244825.1
GCA_025244845.1
GCA_025244865.1
GCA_025244885.1
GCA_025244905.1
GCA_025244925.1
GCA_025244965.1
GCA_025244985.1
GCA_025252265.1
GCA_025252345.1
GCA_025252385.1
GCA_025252405.1
GCA_025252425.1
GCA_025252445.1
GCA_025254305.1
GCA_025254465.1
GCA_025254605.1
GCA_025254745.1
GCA_025258665.1
GCA_025258705.1
GCA_025258745.1
GCA_025258765.1
GCA_025258785.1
GCA_025258805.1
GCA_025258845.1

GCA_025258925.1
GCA_025258945.1
GCA_025259065.1
GCA_025259185.1
GCA_025259345.1
GCA_025259645.1
GCA_025259665.1
GCA_025259685.1
GCA_025263125.1
GCA_025263265.1
GCA_025263485.1
GCA_025263565.1
GCA_025263585.1
GCA_025263605.1
GCA_025263625.1
GCA_025263645.1
GCA_025263665.1
GCA_025263705.1
GCA_025263725.1
GCA_025263745.1
GCA_025263785.1
GCA_025263845.1
GCA_025264005.1
GCA_025264145.1
GCA_025264285.1
GCA_025264445.1
GCA_025264605.1
GCA_025264625.1
GCA_025264645.1
GCA_025264665.1
GCA_025264685.1
GCA_025264705.1
GCA_025264725.1
GCA_025266285.1
GCA_025266575.1
GCA_025266735.1
GCA_025266795.1
GCA_025266855.1
GCA_025268635.1
GCA_025268675.1
GCA_025272695.1
GCA_025272815.1
GCA_025273655.1
GCA_025273675.1
GCA_025309455.1
GCA_025309535.1
GCA_025309655.1
GCA_025309735.1
GCA_025309755.1
GCA_025309775.1
GCA_025310955.1
GCA_025311075.1
GCA_025311235.1
GCA_025311295.1

GCA_025311435.1
GCA_025311455.1
GCA_025311475.1
GCA_025311495.1
GCA_025311515.1
GCA_025311535.1
GCA_025311555.1
GCA_025311595.1
GCA_025319965.1
GCA_025340125.1
GCA_025340225.1
GCA_025340245.1
GCA_025340265.1
GCA_025345525.1
GCA_025345545.1
GCA_025345565.1
GCA_025349925.1
GCA_025349945.1
GCA_025349965.1
GCA_025369675.1
GCA_025369695.1
GCA_025369715.1
GCA_025369735.1
GCA_025369755.1
GCA_025369775.1
GCA_025369795.1
GCA_025369815.1
GCA_025369835.1
GCA_025369855.1
GCA_025369875.1
GCA_025369895.1
GCA_025369915.1
GCA_025369935.1
GCA_025369955.1
GCA_025369975.1
GCA_025370055.1
GCA_025370075.1
GCA_025370975.1
GCA_025397955.1
GCA_025397975.1
GCA_025398015.1
GCA_025398035.1
GCA_025398055.1
GCA_025398075.1
GCA_025398095.1
GCA_025398115.1
GCA_025398135.1
GCA_025398155.2
GCA_025398175.1
GCA_025398355.1
GCA_025398375.1
GCA_025398395.2
GCA_025398435.1
GCA_025398455.1

GCA_025398675.1
GCA_025398695.1
GCA_025398715.1
GCA_025398755.1
GCA_025398795.1
GCA_025398815.1
GCA_025398835.1
GCA_025398855.1
GCA_025398875.1
GCA_025398895.1
GCA_025398915.1
GCA_025398935.1
GCA_025398955.1
GCA_025398975.1
GCA_025398995.1
GCA_025399015.1
GCA_025399035.1
GCA_025399055.1
GCA_025399075.1
GCA_025399095.1
GCA_025399115.1
GCA_025399135.1
GCA_025402735.1
GCA_025402755.1
GCA_025402775.1
GCA_025402795.1
GCA_025402815.1
GCA_025402835.1
GCA_025402855.1
GCA_025402875.1
GCA_025402895.1
GCA_025402915.1
GCA_025402935.1
GCA_025402955.1
GCA_025402975.1
GCA_025403485.1
GCA_025426095.1
GCA_025426135.1
GCA_025426155.1
GCA_025426175.1
GCA_025426195.1
GCA_025426215.1
GCA_025426235.1
GCA_025426255.1
GCA_025426275.1
GCA_025449135.1
GCA_025449155.1
GCA_025449175.1
GCA_025449195.1
GCA_025449215.1
GCA_025449235.1
GCA_025449275.1
GCA_025449295.1
GCA_025449315.1

GCA_025449335.1
GCA_025449355.1
GCA_025451515.1
GCA_025451635.1
GCA_025451795.1
GCA_025451935.1
GCA_025452035.1
GCA_025452095.1
GCA_025452115.1
GCA_025452135.1
GCA_025452195.1
GCA_025452215.1
GCA_025452235.1
GCA_025452255.1
GCA_025490355.1
GCA_025490495.1
GCA_025490515.1
GCA_025490535.1
GCA_025490555.1
GCA_025490575.1
GCA_025502485.1
GCA_025502505.1
GCA_025502525.1
GCA_025502545.1
GCA_025502565.1
GCA_025502585.1
GCA_025558055.1
GCA_025558115.1
GCA_025558185.1
GCA_025558205.1
GCA_025558225.1
GCA_025558245.1
GCA_025558265.1
GCA_025558285.1
GCA_025558305.1
GCA_025558325.1
GCA_025558345.1
GCA_025558365.1
GCA_025558385.1
GCA_025558405.1
GCA_025558425.1
GCA_025558445.1
GCA_025558485.1
GCA_025558525.1
GCA_025558545.1
GCA_025558565.1
GCA_025558585.1
GCA_025558605.1
GCA_025558625.1
GCA_025558645.1
GCA_025558665.1
GCA_025558685.1
GCA_025558705.1
GCA_025558725.1

GCA_025558745.1
GCA_025558765.1
GCA_025558785.1
GCA_025558805.1
GCA_025558825.1
GCA_025558845.1
GCA_025558865.1
GCA_025558905.1
GCA_025558945.1
GCA_025558965.1
GCA_025559005.1
GCA_025559045.1
GCA_025559085.1
GCA_025559125.1
GCA_025559165.1
GCA_025559225.1
GCA_025559265.1
GCA_025559325.1
GCA_025559405.1
GCA_025559465.1
GCA_025559525.1
GCA_025559565.1
GCA_025559585.1
GCA_025559605.1
GCA_025559625.1
GCA_025559645.1
GCA_025559665.1
GCA_025559685.1
GCA_025559705.1
GCA_025559725.1
GCA_025559745.1
GCA_025559765.1
GCA_025559785.1
GCA_025559805.1
GCA_025559845.1
GCA_025559885.1
GCA_025559945.1
GCA_025560005.1
GCA_025560085.1
GCA_025560125.1
GCA_025560165.1
GCA_025560205.1
GCA_025560225.1
GCA_025560245.1
GCA_025560285.1
GCA_025560305.1
GCA_025560345.1
GCA_025560405.1
GCA_025560445.1
GCA_025560485.1
GCA_025560525.1
GCA_025560545.1
GCA_025560585.1
GCA_025560665.1

GCA_025560685.1
GCA_025560745.1
GCA_025560785.1
GCA_025560805.1
GCA_025560825.1
GCA_025560845.1
GCA_025560865.1
GCA_025560885.1
GCA_025560905.1
GCA_025560925.1
GCA_025560945.1
GCA_025560965.1
GCA_025561005.1
GCA_025561025.1
GCA_025561045.1
GCA_025561085.1
GCA_025561145.1
GCA_025561165.1
GCA_025561205.1
GCA_025561265.1
GCA_025561305.1
GCA_025561365.1
GCA_025561425.1
GCA_025561445.1
GCA_025561465.1
GCA_025561505.1
GCA_025561545.1
GCA_025561565.1
GCA_025561585.1
GCA_025561605.1
GCA_025561625.1
GCA_025561645.1
GCA_025561665.1
GCA_025562635.2
GCA_025562675.2
GCA_025562755.1
GCA_025562795.1
GCA_025562815.1
GCA_025562895.2
GCA_025562955.3
GCA_025563435.1
GCA_025563475.1
GCA_025563515.1
GCA_025579125.2
GCA_025579405.2
GCA_025579425.2
GCA_025579445.2
GCA_025579525.2
GCA_025583295.2
GCA_025583345.2
GCA_025583375.2
GCA_025583505.2
GCA_025583565.2
GCA_025583585.2

GCA_025583665.1
GCA_025600135.2
GCA_025600175.2
GCA_025617515.1
GCA_025628925.2
GCA_025639805.1
GCA_025639885.1
GCA_025641845.1
GCA_025641865.1
GCA_025642095.1
GCA_025642115.1
GCA_025642135.1
GCA_025642155.1
GCA_025642175.1
GCA_025642195.1
GCA_025642215.1
GCA_025642235.1
GCA_025642255.1
GCA_025642275.1
GCA_025642295.1
GCA_025642315.1
GCA_025642335.1
GCA_025642355.1
GCA_025642375.1
GCA_025642395.1
GCA_025642415.1
GCA_025642435.1
GCA_025642455.1
GCA_025642475.1
GCA_025642995.1
GCA_025643015.1
GCA_025643035.1
GCA_025643055.1
GCA_025643075.1
GCA_025643095.1
GCA_025643115.1
GCA_025643135.1
GCA_025643175.1
GCA_025643195.1
GCA_025643275.1
GCA_025643295.1
GCA_025643315.1
GCA_025643355.1
GCA_025643375.1
GCA_025643395.1
GCA_025643415.1
GCA_025643435.1
GCA_025643455.1
GCA_025643475.1
GCA_025643495.1
GCA_025643515.1
GCA_025643555.1
GCA_025643575.1
GCA_025643595.1

GCA_025643615.1
GCA_025643635.1
GCA_025643655.1
GCA_025643675.1
GCA_025643735.1
GCA_025665255.1
GCA_025665275.1
GCA_025665295.1
GCA_025665315.1
GCA_025665335.1
GCA_025665355.1
GCA_025665375.1
GCA_025665395.1
GCA_025665415.1
GCA_025665435.1
GCA_025665475.1
GCA_025666195.1
GCA_025666215.1
GCA_025666235.1
GCA_025666255.1
GCA_025676565.2
GCA_025676585.2
GCA_025676845.2
GCA_025677645.1
GCA_025677665.1
GCA_025677685.1
GCA_025677705.1
GCA_025677725.1
GCA_025677745.1
GCA_025677765.1
GCA_025677785.1
GCA_025677855.1
GCA_025716785.1
GCA_025720855.1
GCA_025722955.1
GCA_025722975.1
GCA_025723025.1
GCA_025723085.1
GCA_025723105.1
GCA_025723125.1
GCA_025723145.1
GCA_025723165.1
GCA_025723205.1
GCA_025723225.1
GCA_025723245.1
GCA_025725645.1
GCA_025725665.1
GCA_025725685.1
GCA_025725705.1
GCA_025725725.1
GCA_025725745.1
GCA_025727575.1
GCA_025732135.1
GCA_025732155.1

GCA_025732175.1
GCA_025734435.1
GCA_025736815.1
GCA_025736875.1
GCA_025736895.1
GCA_025736935.1
GCA_025739165.1
GCA_025739325.1
GCA_025757485.1
GCA_025757525.1
GCA_025757545.1
GCA_025757585.1
GCA_025757605.1
GCA_025757645.1
GCA_025757665.1
GCA_025757685.1
GCA_025757705.1
GCA_025757725.1
GCA_025757765.1
GCA_025757805.1
GCA_025757845.1
GCA_025757905.1
GCA_025757925.1
GCA_025757985.1
GCA_025758025.1
GCA_025758045.1
GCA_025758065.1
GCA_025758085.1
GCA_025758105.1
GCA_025758125.1
GCA_025758145.1
GCA_025758165.1
GCA_025758185.1
GCA_025758205.1
GCA_025758225.1
GCA_025758245.1
GCA_025758395.1
GCA_025758415.1
GCA_025765755.1
GCA_025765795.1
GCA_025765815.1
GCA_025765835.1
GCA_025765855.1
GCA_025765875.1
GCA_025765895.1
GCA_025765915.1
GCA_025765935.1
GCA_025765955.1
GCA_025790825.1
GCA_025790845.1
GCA_025790865.1
GCA_025790885.1
GCA_025790925.1
GCA_025790945.1

GCA_025790965.1
GCA_025791005.1
GCA_025792455.1
GCA_025792515.1
GCA_025792535.1
GCA_025792555.1
GCA_025792575.1
GCA_025792595.1
GCA_025792615.1
GCA_025792635.1
GCA_025792655.1
GCA_025792675.1
GCA_025808175.1
GCA_025808195.1
GCA_025808215.1
GCA_025808235.1
GCA_025808255.1
GCA_025808275.1
GCA_025808295.1
GCA_025808315.1
GCA_025808335.1
GCA_025808355.1
GCA_025808375.1
GCA_025809195.1
GCA_025809235.1
GCA_025809255.1
GCA_025809295.1
GCA_025809335.1
GCA_025809355.1
GCA_025809395.1
GCA_025811215.1
GCA_025811355.1
GCA_025811415.1
GCA_025811435.1
GCA_025813515.1
GCA_025813715.1
GCA_025813735.1
GCA_025820815.1
GCA_025836975.1
GCA_025836995.1
GCA_025837015.1
GCA_025837075.1
GCA_025837095.1
GCA_025837115.1
GCA_025837135.1
GCA_025837155.1
GCA_025853815.1
GCA_025853835.1
GCA_025853855.1
GCA_025853875.1
GCA_025853895.1
GCA_025853915.1
GCA_025853935.1
GCA_025853955.1

GCA_025853975.1
GCA_025853995.1
GCA_025854015.1
GCA_025854035.1
GCA_025854055.1
GCA_025854075.1
GCA_025854095.1
GCA_025854115.1
GCA_025854135.1
GCA_025854155.1
GCA_025854175.1
GCA_025854195.1
GCA_025854215.1
GCA_025854235.1
GCA_025854255.1
GCA_025854275.1
GCA_025854315.1
GCA_025854335.1
GCA_025854375.1
GCA_025857195.1
GCA_025857215.1
GCA_025859515.1
GCA_025859615.1
GCA_025859635.1
GCA_025860405.1
GCA_025884075.1
GCA_025884095.1
GCA_025884115.1
GCA_025884135.1
GCA_025884155.1
GCA_025884175.1
GCA_025884195.1
GCA_025884215.1
GCA_025884235.1
GCA_025884255.1
GCA_025884275.1
GCA_025884395.1
GCA_025884415.1
GCA_025884515.1
GCA_025884555.1
GCA_025905425.1
GCA_025905445.1
GCA_025905465.1
GCA_025905485.1
GCA_025905525.1
GCA_025908215.1
GCA_025908235.1
GCA_025908255.1
GCA_025908275.1
GCA_025908295.1
GCA_025908315.1
GCA_025908395.1
GCA_025908435.1
GCA_025908455.1

GCA_025908475.1
GCA_025913555.1
GCA_025913595.1
GCA_025913615.1
GCA_025913635.1
GCA_025913655.1
GCA_025913675.1
GCA_025913695.1
GCA_025913735.1
GCA_025913755.1
GCA_025913855.1
GCA_025914055.1
GCA_025914075.1
GCA_025914095.1
GCA_025916135.1
GCA_025916175.1
GCA_025916215.1
GCA_025916235.1
GCA_025917005.1
GCA_025917315.1
GCA_025917635.1
GCA_025917685.1
GCA_025917705.1
GCA_025917725.1
GCA_025917745.1
GCA_025917765.1
GCA_025919585.1
GCA_025919605.1
GCA_025919725.1
GCA_025919745.1
GCA_025919765.1
GCA_025920025.1
GCA_025920045.1
GCA_025920065.1
GCA_025920085.1
GCA_025920785.1
GCA_025946485.2
GCA_025946525.1
GCA_025946545.1
GCA_025946565.1
GCA_025946585.1
GCA_025946725.1
GCA_025946745.1
GCA_025946765.1
GCA_025946785.1
GCA_025946805.1
GCA_025946825.1
GCA_025946845.1
GCA_025946865.1
GCA_025946885.1
GCA_025946905.1
GCA_025946925.1
GCA_025946945.1
GCA_025946965.1

GCA_025946985.1
GCA_025947025.1
GCA_025947045.1
GCA_025947065.1
GCA_025947085.1
GCA_025947105.1
GCA_025947125.1
GCA_025947145.1
GCA_025947165.1
GCA_025947185.1
GCA_025947205.1
GCA_025947225.1
GCA_025947245.1
GCA_025947265.1
GCA_025947285.1
GCA_025947305.1
GCA_025947325.1
GCA_025947345.1
GCA_025947365.1
GCA_025947385.1
GCA_025947955.1
GCA_025948295.1
GCA_025957665.1
GCA_025957845.1
GCA_025957925.1
GCA_025957945.1
GCA_025957965.1
GCA_025957985.1
GCA_025958005.1
GCA_025958025.1
GCA_025958045.1
GCA_025958065.1
GCA_025958085.1
GCA_025958125.1
GCA_025958325.1
GCA_025958445.1
GCA_025958625.1
GCA_025958785.1
GCA_025958805.1
GCA_025958825.1
GCA_025958845.1
GCA_025958865.1
GCA_025958885.1
GCA_025958905.1
GCA_025958925.1
GCA_025960205.1
GCA_025960245.1
GCA_025960285.1
GCA_025960305.1
GCA_025960325.1
GCA_025960345.1
GCA_025960365.1
GCA_025960385.1
GCA_025962575.1

GCA_025979705.1
GCA_025979885.1
GCA_025980045.1
GCA_025983385.1
GCA_025983525.1
GCA_025983625.1
GCA_025983645.1
GCA_025983665.1
GCA_025984245.1
GCA_025984365.1
GCA_025984385.1
GCA_025984405.1
GCA_025984425.1
GCA_025984445.1
GCA_025984465.1
GCA_025984485.1
GCA_025984525.1
GCA_025984565.1
GCA_025984585.1
GCA_025984605.1
GCA_025984625.1
GCA_025984845.1
GCA_025984965.1
GCA_025985165.1
GCA_025985185.1
GCA_025985205.1
GCA_025985225.1
GCA_025985245.1
GCA_025985265.1
GCA_025985285.1
GCA_025985305.1
GCA_025985325.1
GCA_025985345.1
GCA_025985365.1
GCA_025985385.1
GCA_025985405.1
GCA_025985425.1
GCA_025985465.1
GCA_025985485.1
GCA_025985505.1
GCA_025985525.1
GCA_025987645.1
GCA_025987665.1
GCA_025987685.1
GCA_025987705.1
GCA_025987725.1
GCA_025987745.1
GCA_025987765.1
GCA_025987785.1
GCA_025987805.1
GCA_025987825.1
GCA_025998455.1
GCA_026013235.1
GCA_026013255.1

GCA_026013275.1
GCA_026013295.1
GCA_026013315.1
GCA_026013645.1
GCA_026013665.1
GCA_026013705.1
GCA_026013725.1
GCA_026013745.1
GCA_026013765.1
GCA_026013785.1
GCA_026013805.1
GCA_026013825.1
GCA_026013845.1
GCA_026013865.1
GCA_026013885.1
GCA_026013905.1
GCA_026013945.1
GCA_026013965.1
GCA_026013985.1
GCA_026014005.1
GCA_026014025.1
GCA_026015925.1
GCA_026015945.1
GCA_026015985.1
GCA_026016005.1
GCA_026016025.1
GCA_026016045.1
GCA_026016065.1
GCA_026016085.1
GCA_026016105.1
GCA_026016125.1
GCA_026016145.1
GCA_026016165.1
GCA_026016185.1
GCA_026016225.1
GCA_026016265.1
GCA_026016285.1
GCA_026016305.1
GCA_026016325.1
GCA_026016345.1
GCA_026016365.1
GCA_026016385.1
GCA_026016405.1
GCA_026016425.1
GCA_026016445.1
GCA_026016465.1
GCA_026016485.1
GCA_026016505.1
GCA_026016525.1
GCA_026016545.1
GCA_026016565.1
GCA_026016605.1
GCA_026016665.1
GCA_026016705.1

GCA_026016725.1
GCA_026016745.1
GCA_026016765.1
GCA_026016785.1
GCA_026016825.1
GCA_026016845.1
GCA_026020075.1
GCA_026057535.1
GCA_026057715.1
GCA_026057735.1
GCA_026062495.1
GCA_026062515.1
GCA_026069065.2
GCA_026069095.2
GCA_026072635.1
GCA_026072755.1
GCA_026072915.1
GCA_026073035.1
GCA_026073115.1
GCA_026073255.1
GCA_026073355.1
GCA_026073375.1
GCA_026073395.1
GCA_026073415.1
GCA_026073435.1
GCA_026073455.1
GCA_026073475.1
GCA_026073495.1
GCA_026073515.1
GCA_026073535.1
GCA_026073555.1
GCA_026073625.1
GCA_026073735.1
GCA_026073915.1
GCA_026074015.1
GCA_026074235.1
GCA_026075575.1
GCA_026104315.1
GCA_026104335.1
GCA_026104355.1
GCA_026109735.1
GCA_026109755.1
GCA_026122595.1
GCA_026122615.1
GCA_026151165.2
GCA_026153115.1
GCA_026153295.1
GCA_026153315.1
GCA_026153335.1
GCA_026153355.1
GCA_026153375.1
GCA_026153395.1
GCA_026153435.1
GCA_026153455.1

GCA_026153475.1
GCA_026153495.1
GCA_026153515.1
GCA_026153535.1
GCA_026153555.1
GCA_026153595.1
GCA_026153615.1
GCA_026153635.1
GCA_026167525.1
GCA_026167545.1
GCA_026167565.1
GCA_026167585.1
GCA_026167605.1
GCA_026167625.1
GCA_026167645.1
GCA_026167665.1
GCA_026167725.1
GCA_026167765.1
GCA_026167785.1
GCA_026167805.1
GCA_026168515.1
GCA_026168555.1
GCA_026170095.1
GCA_026183345.1
GCA_026183365.1
GCA_026183385.1
GCA_026183415.1
GCA_026183435.1
GCA_026183455.1
GCA_026183475.1
GCA_026184215.1
GCA_026184235.1
GCA_026184355.1
GCA_026184435.1
GCA_026185255.2
GCA_026185275.1
GCA_026191725.2
GCA_026194135.2
GCA_026194635.2
GCA_026210435.1
GCA_026210455.1
GCA_026210475.1
GCA_026210495.1
GCA_026210515.1
GCA_026210615.1
GCA_026210635.1
GCA_026210655.1
GCA_026210675.1
GCA_026210695.1
GCA_026210715.1
GCA_026230175.1
GCA_026239475.1
GCA_026239495.1
GCA_026239515.1

GCA_026239535.1
GCA_026240615.1
GCA_026240635.1
GCA_026240655.1
GCA_026240675.1
GCA_026240695.1
GCA_026240755.1
GCA_026240775.1
GCA_026240795.1
GCA_026240815.1
GCA_026240835.1
GCA_026247685.1
GCA_026247825.1
GCA_026247845.1
GCA_026247865.1
GCA_026247885.1
GCA_026247905.1
GCA_026247925.1
GCA_026247945.1
GCA_026247965.1
GCA_026247985.1
GCA_026248005.1
GCA_026248225.1
GCA_026248385.1
GCA_026248545.1
GCA_026248825.1
GCA_026248845.1
GCA_026248865.1
GCA_026248885.1
GCA_026250525.1
GCA_026254485.1
GCA_026261605.1
GCA_026261625.1
GCA_026261685.1
GCA_026261705.1
GCA_026314255.1
GCA_026314275.1
GCA_026314295.1
GCA_026314315.1
GCA_026314335.1
GCA_026314355.1
GCA_026314945.1
GCA_026315005.1
GCA_026315025.1
GCA_026315045.1
GCA_026315065.1
GCA_026315085.1
GCA_026315125.1
GCA_026315145.1
GCA_026315165.1
GCA_026315225.1
GCA_026409165.1
GCA_026409185.1
GCA_026409205.1

GCA_026409225.1
GCA_026409245.1
GCA_026409265.1
GCA_026409285.1
GCA_026409305.1
GCA_026409325.1
GCA_026409345.1
GCA_026409365.1
GCA_026409565.1
GCA_026410045.1
GCA_026410065.1
GCA_026410105.1
GCA_026410125.1
GCA_026410845.1
GCA_026410865.1
GCA_026410905.1
GCA_026427355.1
GCA_026427375.1
GCA_026427395.1
GCA_026427415.1
GCA_026427435.1
GCA_026427455.1
GCA_026427475.1
GCA_026427495.1
GCA_026427515.1
GCA_026427535.1
GCA_026427555.1
GCA_026427575.1
GCA_026427595.1
GCA_026427615.1
GCA_026427635.1
GCA_026427675.1
GCA_026427695.1
GCA_026427715.1
GCA_026427735.1
GCA_026427755.1
GCA_026427775.1
GCA_026428215.1
GCA_026428235.1
GCA_026428255.1
GCA_026428275.1
GCA_026428295.1
GCA_026428315.1
GCA_026428335.1
GCA_026430575.1
GCA_026435115.1
GCA_026435135.1
GCA_026435155.1
GCA_026435175.1
GCA_026435195.1
GCA_026435215.1
GCA_026435235.1
GCA_026435255.1
GCA_026435275.1

GCA_026435295.1
GCA_026435315.1
GCA_026435335.1
GCA_026435495.1
GCA_026435655.1
GCA_026435835.1
GCA_026506315.1
GCA_026506335.1
GCA_026506355.1
GCA_026506375.1
GCA_026506395.1
GCA_026506415.1
GCA_026546985.1
GCA_026547005.1
GCA_026547035.1
GCA_026547055.1
GCA_026547075.1
GCA_026547095.1
GCA_026547145.1
GCA_026547185.1
GCA_026547205.1
GCA_026547225.1
GCA_026559675.1
GCA_026559715.1
GCA_026559735.1
GCA_026559755.1
GCA_026559775.1
GCA_026559795.1
GCA_026625025.1
GCA_026625045.1
GCA_026625065.1
GCA_026625085.1
GCA_026625105.1
GCA_026625125.1
GCA_026625145.1
GCA_026625185.1
GCA_026625205.1
GCA_026625225.1
GCA_026625245.1
GCA_026625265.1
GCA_026625285.1
GCA_026625305.1
GCA_026625325.1
GCA_026625345.1
GCA_026625365.1
GCA_026625385.1
GCA_026625405.1
GCA_026625425.1
GCA_026625445.1
GCA_026625465.1
GCA_026625485.1
GCA_026625505.1
GCA_026625525.1
GCA_026625545.1

GCA_026625905.1
GCA_026625925.1
GCA_026625945.1
GCA_026625965.1
GCA_026625985.1
GCA_026626005.1
GCA_026626025.1
GCA_026626045.1
GCA_026626065.1
GCA_026626085.1
GCA_026626105.1
GCA_026626125.1
GCA_026626145.1
GCA_026626165.1
GCA_026626185.1
GCA_026626205.1
GCA_026626225.1
GCA_026626245.1
GCA_026626265.1
GCA_026626285.1
GCA_026635925.1
GCA_026636035.1
GCA_026636135.1
GCA_026636195.1
GCA_026636215.1
GCA_026636235.1
GCA_026636255.1
GCA_026639055.1
GCA_026639155.1
GCA_026639235.1
GCA_026639315.1
GCA_026642215.1
GCA_026642235.1
GCA_026642255.1
GCA_026642275.1
GCA_026642295.1
GCA_026642315.1
GCA_026642335.1
GCA_026642355.1
GCA_026642375.1
GCA_026642395.1
GCA_026642415.1
GCA_026642435.1
GCA_026642455.1
GCA_026642475.1
GCA_026642495.1
GCA_026642515.1
GCA_026642535.1
GCA_026642555.1
GCA_026642575.1
GCA_026642595.1
GCA_026642615.1
GCA_026642635.1
GCA_026642655.1

GCA_026642675.1
GCA_026642795.1
GCA_026642855.1
GCA_026642955.1
GCA_026643035.1
GCA_026643175.1
GCA_026643295.1
GCA_026643355.1
GCA_026643375.1
GCA_026643395.1
GCA_026643415.1
GCA_026643455.1
GCA_026643475.1
GCA_026643495.1
GCA_026643515.1
GCA_026643535.1
GCA_026643555.1
GCA_026643575.1
GCA_026643595.1
GCA_026643615.1
GCA_026644015.1
GCA_026644225.1
GCA_026644375.1
GCA_026644475.1
GCA_026644635.1
GCA_026644785.1
GCA_026647725.1
GCA_026647855.1
GCA_026647995.1
GCA_026648075.1
GCA_026648185.1
GCA_026648235.1
GCA_026648315.1
GCA_026648355.1
GCA_026648415.1
GCA_026683895.1
GCA_026683915.1
GCA_026683935.1
GCA_026683955.1
GCA_026684115.1
GCA_026684135.1
GCA_026684155.1
GCA_026684175.1
GCA_026684195.1
GCA_026684215.1
GCA_026684235.1
GCA_026684255.1
GCA_026684275.1
GCA_026686735.1
GCA_026689315.1
GCA_026689335.1
GCA_026689355.1
GCA_026689375.1
GCA_026689395.1

GCA_026689415.1
GCA_026689435.1
GCA_026689455.1
GCA_026689475.1
GCA_026689495.1
GCA_026689515.1
GCA_026692605.1
GCA_026692645.1
GCA_026723705.1
GCA_026723725.1
GCA_026723745.1
GCA_026723765.1
GCA_026723805.1
GCA_026723825.1
GCA_026723845.1
GCA_026723865.1
GCA_026725475.1
GCA_026725495.1
GCA_026727575.1
GCA_026727615.1
GCA_026727675.1
GCA_026727695.1
GCA_026727715.1
GCA_026727735.1
GCA_026727755.1
GCA_026801915.1
GCA_026801935.1
GCA_026801955.1
GCA_026801975.1
GCA_026801995.1
GCA_026802015.1
GCA_026802035.1
GCA_026802055.1
GCA_026802075.1
GCA_026802115.1
GCA_026802135.1
GCA_026802175.1
GCA_026802195.1
GCA_026802215.1
GCA_026802235.1
GCA_026802345.1
GCA_026802365.1
GCA_026802385.1
GCA_026810085.1
GCA_026810205.1
GCA_026810225.1
GCA_026810245.1
GCA_026810365.2
GCA_026810445.1
GCA_026810505.1
GCA_026821715.1
GCA_026821955.1
GCA_026870135.1
GCA_026870155.3

GCA_026870175.2
GCA_026873305.1
GCA_026873325.1
GCA_026873345.1
GCA_026873365.1
GCA_026873385.1
GCA_026873405.1
GCA_026873425.1
GCA_026873445.1
GCA_026873465.1
GCA_026873485.1
GCA_026873505.1
GCA_026873525.1
GCA_026874105.1
GCA_026874125.1
GCA_026874145.1
GCA_026898095.1
GCA_026898115.1
GCA_026898135.1
GCA_026898155.1
GCA_026898175.1
GCA_026898195.1
GCA_026958255.1
GCA_026965535.1
GCA_026965555.1
GCA_026967475.1
GCA_026967495.1
GCA_026967515.2
GCA_026967535.1
GCA_026967555.1
GCA_026967575.1
GCA_026967595.1
GCA_026967615.1
GCA_026967635.1
GCA_026967655.1
GCA_026967675.1
GCA_026967695.1
GCA_026967715.1
GCA_026967735.1
GCA_026967755.1
GCA_026970855.1
GCA_026970895.1
GCA_026970915.1
GCA_026975895.1
GCA_026976075.1
GCA_026976255.1
GCA_026976295.1
GCA_026976315.1
GCA_027105035.1
GCA_027105055.1
GCA_027105095.1
GCA_027107535.1
GCA_027111095.2
GCA_027111255.2

GCA_027111275.3
GCA_027111295.2
GCA_027111315.2
GCA_027111335.2
GCA_027111355.2
GCA_027116395.1
GCA_027116415.1
GCA_027116435.1
GCA_027116455.1
GCA_027116475.1
GCA_027116495.1
GCA_027116515.1
GCA_027116535.1
GCA_027116555.1
GCA_027116575.1
GCA_027118995.1
GCA_027125355.1
GCA_027126715.1
GCA_027171385.1
GCA_027171405.1
GCA_027186265.1
GCA_027186285.1
GCA_027186305.1
GCA_027186325.1
GCA_027239755.1
GCA_027239995.1
GCA_027247405.1
GCA_027252975.2
GCA_027257035.1
GCA_027270315.1
GCA_027271155.1
GCA_027271175.1
GCA_027271235.1
GCA_027285985.1
GCA_027286005.1
GCA_027286085.1
GCA_027286105.1
GCA_027286125.1
GCA_027286145.1
GCA_027286165.1
GCA_027286185.1
GCA_027286205.1
GCA_027286225.1
GCA_027286245.1
GCA_027286265.1
GCA_027286285.1
GCA_027286305.1
GCA_027286325.1
GCA_027286345.1
GCA_027286365.1
GCA_027286385.1
GCA_027359115.1
GCA_027359235.1
GCA_027359355.1

GCA_027359375.1
GCA_027359395.1
GCA_027359415.1
GCA_027359475.1
GCA_027359525.1
GCA_027359575.1
GCA_027359595.1
GCA_027359675.1
GCA_027359925.1
GCA_027360555.1
GCA_027362595.1
GCA_027366395.1
GCA_027366555.1
GCA_027474365.1
GCA_027474385.1
GCA_027474405.1
GCA_027474425.1
GCA_027474445.1
GCA_027474465.1
GCA_027474485.1
GCA_027474505.1
GCA_027474525.2
GCA_027474565.1
GCA_027474845.2
GCA_027474865.2
GCA_027474905.1
GCA_027474925.1
GCA_027474945.1
GCA_027474965.1
GCA_027474985.1
GCA_027475005.1
GCA_027475025.1
GCA_027497435.1
GCA_027497475.1
GCA_027497495.1
GCA_027498035.1
GCA_027498055.1
GCA_027503885.1
GCA_027504005.1
GCA_027504065.1
GCA_027504085.1
GCA_027504105.1
GCA_027557445.1
GCA_027557545.1
GCA_027557615.1
GCA_027557655.1
GCA_027557675.1
GCA_027557715.1
GCA_027557735.1
GCA_027557755.1
GCA_027557955.1
GCA_027558075.1
GCA_027558275.1
GCA_027558435.1

GCA_027558615.1
GCA_027558775.1
GCA_027558815.1
GCA_027558835.1
GCA_027558855.1
GCA_027563115.1
GCA_027563495.1
GCA_027569775.1
GCA_027569975.1
GCA_027570015.1
GCA_027570235.2
GCA_027570415.1
GCA_027570435.1
GCA_027570455.1
GCA_027570475.1
GCA_027570495.1
GCA_027570515.1
GCA_027570535.1
GCA_027570555.1
GCA_027570575.1
GCA_027570595.1
GCA_027570615.1
GCA_027570635.1
GCA_027570655.1
GCA_027570675.1
GCA_027570695.1
GCA_027570715.1
GCA_027570735.1
GCA_027570755.1
GCA_027570775.1
GCA_027570795.1
GCA_027570815.1
GCA_027570835.1
GCA_027570855.1
GCA_027570875.1
GCA_027570895.1
GCA_027570915.1
GCA_027570935.1
GCA_027570955.1
GCA_027570975.1
GCA_027570995.1
GCA_027571015.1
GCA_027571035.1
GCA_027571055.1
GCA_027571075.1
GCA_027571095.1
GCA_027571115.1
GCA_027571135.1
GCA_027571155.1
GCA_027571175.1
GCA_027571195.1
GCA_027571215.1
GCA_027571285.1
GCA_027571305.1

GCA_027571325.1
GCA_027571345.1
GCA_027571365.1
GCA_027571385.1
GCA_027571405.1
GCA_027577885.1
GCA_027594365.1
GCA_027594385.1
GCA_027594405.1
GCA_027594425.1
GCA_027594445.1
GCA_027594465.1
GCA_027594485.1
GCA_027594505.1
GCA_027594525.1
GCA_027594545.1
GCA_027594565.1
GCA_027594625.1
GCA_027594645.1
GCA_027594685.1
GCA_027594725.1
GCA_027594745.1
GCA_027594765.1
GCA_027594805.1
GCA_027594845.1
GCA_027594865.1
GCA_027594885.1
GCA_027594905.1
GCA_027594925.1
GCA_027594945.1
GCA_027594965.1
GCA_027594985.1
GCA_027595005.1
GCA_027595025.1
GCA_027595045.1
GCA_027595065.1
GCA_027595085.1
GCA_027595105.2
GCA_027595125.1
GCA_027595145.1
GCA_027595165.1
GCA_027595485.1
GCA_027595505.1
GCA_027595525.1
GCA_027595545.1
GCA_027595585.1
GCA_027595605.1
GCA_027595625.1
GCA_027595645.1
GCA_027595665.1
GCA_027595685.1
GCA_027595705.1
GCA_027595745.1
GCA_027595845.1

GCA_027595935.1
GCA_027625375.1
GCA_027625395.1
GCA_027625415.1
GCA_027625435.1
GCA_027625455.1
GCA_027625475.1
GCA_027625935.1
GCA_027625975.1
GCA_027625995.1
GCA_027626015.1
GCA_027626035.1
GCA_027626975.1
GCA_027627435.1
GCA_027627455.1
GCA_027627475.1
GCA_027627495.1
GCA_027627535.1
GCA_027627555.1
GCA_027627575.1
GCA_027627615.1
GCA_027627685.1
GCA_027627705.1
GCA_027627725.1
GCA_027627745.1
GCA_027627765.1
GCA_027627785.1
GCA_027701665.1
GCA_027721405.1
GCA_027857035.1
GCA_027886375.1
GCA_027886425.1
GCA_027886475.1
GCA_027886505.1
GCA_027886525.1
GCA_027886545.1
GCA_027886565.1
GCA_027886585.1
GCA_027886605.1
GCA_027886645.1
GCA_027886665.1
GCA_027886705.1
GCA_027912195.1
GCA_027912355.1
GCA_027912415.1
GCA_027912455.1
GCA_027912495.1
GCA_027912515.1
GCA_027912535.1
GCA_027912555.1
GCA_027912575.1
GCA_027912835.1
GCA_027913035.1
GCA_027913215.1

GCA_027913355.1
GCA_027915235.1
GCA_027915275.1
GCA_027915295.1
GCA_027919895.1
GCA_027920325.1
GCA_027920365.1
GCA_027920385.1
GCA_027920405.1
GCA_027920425.1
GCA_027920465.1
GCA_027920485.1
GCA_027920505.1
GCA_027920525.1
GCA_027920545.1
GCA_027920565.1
GCA_027920585.1
GCA_027920605.1
GCA_027920625.1
GCA_027920765.2
GCA_027920785.2
GCA_027921445.1
GCA_027923765.1
GCA_027924345.1
GCA_027924405.1
GCA_027924565.1
GCA_027925385.1
GCA_027938815.1
GCA_027941655.1
GCA_027941715.1
GCA_027941755.1
GCA_027941775.1
GCA_027941795.1
GCA_027941835.1
GCA_027941855.1
GCA_027941875.1
GCA_027941895.1
GCA_027941915.1
GCA_027941935.1
GCA_027941955.1
GCA_027941975.1
GCA_027941995.1
GCA_027942015.1
GCA_027942035.1
GCA_027942055.1
GCA_027942075.1
GCA_027942095.1
GCA_027942115.1
GCA_027942135.1
GCA_027942155.1
GCA_027942175.1
GCA_027942195.1
GCA_027942215.1
GCA_027942235.1

GCA_027942255.1
GCA_027942275.1
GCA_027942295.1
GCA_027942315.1
GCA_027942355.1
GCA_027942375.1
GCA_027942395.1
GCA_027942415.1
GCA_027942435.1
GCA_027942455.1
GCA_027943565.1
GCA_027943585.1
GCA_027943605.1
GCA_027943625.1
GCA_027943645.1
GCA_027943665.1
GCA_027943685.1
GCA_027943705.1
GCA_027943725.1
GCA_027943765.1
GCA_027943785.1
GCA_027943805.1
GCA_027943825.1
GCA_027943845.1
GCA_027943865.1
GCA_027943885.1
GCA_027943905.1
GCA_027943925.1
GCA_027943945.1
GCA_027943965.1
GCA_027943985.1
GCA_027944005.1
GCA_027944535.1
GCA_027944555.1
GCA_027944575.1
GCA_027944595.1
GCA_027944615.1
GCA_027944635.1
GCA_027944655.1
GCA_027944675.1
GCA_027944695.1
GCA_027944715.1
GCA_027944735.1
GCA_027944775.1
GCA_027944795.1
GCA_027944815.1
GCA_027944835.1
GCA_027944855.1
GCA_027944875.1
GCA_027944895.1
GCA_027944915.1
GCA_027944935.1
GCA_027944955.1
GCA_027944995.1

GCA_027945015.1
GCA_027945035.1
GCA_027945055.1
GCA_027945075.1
GCA_027945095.1
GCA_027945115.1
GCA_027945135.1
GCA_027945155.1
GCA_027945175.1
GCA_027945195.1
GCA_027945215.1
GCA_027945475.1
GCA_027945845.1
GCA_027946175.1
GCA_027947475.1
GCA_027947495.1
GCA_027947515.1
GCA_027947595.1
GCA_027947615.1
GCA_027947635.1
GCA_027947655.1
GCA_027947675.1
GCA_028009925.1
GCA_028010105.1
GCA_028010205.1
GCA_028010245.1
GCA_028010285.1
GCA_028021195.1
GCA_028215815.1
GCA_028215935.1
GCA_028215955.1
GCA_028215975.1
GCA_028215995.1
GCA_028219255.1
GCA_028219375.1
GCA_028219495.1
GCA_028219695.1
GCA_028219835.1
GCA_028219935.1
GCA_028219955.1
GCA_028219975.1
GCA_028219995.1
GCA_028220015.1
GCA_028220175.1
GCA_028220555.1
GCA_028220765.1
GCA_028220865.1
GCA_028220965.1
GCA_028220985.1
GCA_028221005.1
GCA_028221025.1
GCA_028221045.1
GCA_028221065.1
GCA_028221085.1

GCA_028221105.1
GCA_028221125.1
GCA_028221225.1
GCA_028221515.1
GCA_028221855.1
GCA_028222115.1
GCA_028222535.1
GCA_028222915.1
GCA_028223205.1
GCA_028223415.1
GCA_028223615.1
GCA_028223855.1
GCA_028224025.1
GCA_028224355.1
GCA_028224675.1
GCA_028224955.1
GCA_028225315.1
GCA_028225535.1
GCA_028225725.1
GCA_028225915.1
GCA_028226055.1
GCA_028226215.1
GCA_028226435.1
GCA_028226565.1
GCA_028226775.1
GCA_028226975.1
GCA_028227275.1
GCA_028227635.1
GCA_028228155.1
GCA_028228315.1
GCA_028228685.1
GCA_028335085.1
GCA_028335105.1
GCA_028335125.1
GCA_028335145.1
GCA_028335165.1
GCA_028335185.1
GCA_028335205.1
GCA_028335225.1
GCA_028335245.1
GCA_028335265.1
GCA_028335285.1
GCA_028335305.1
GCA_028335325.1
GCA_028335345.1
GCA_028335365.1
GCA_028335385.1
GCA_028335405.1
GCA_028335425.1
GCA_028335445.1
GCA_028335465.1
GCA_028335485.1
GCA_028335505.1
GCA_028335525.1

GCA_028335545.1
GCA_028335565.1
GCA_028335585.1
GCA_028335605.1
GCA_028335625.1
GCA_028335645.1
GCA_028335665.1
GCA_028335685.1
GCA_028335705.1
GCA_028335725.1
GCA_028335745.1
GCA_028335765.1
GCA_028335785.1
GCA_028335805.1
GCA_028355655.1
GCA_028355815.1
GCA_028355995.1
GCA_028356175.1
GCA_028356375.1
GCA_028356555.1
GCA_028356755.1
GCA_028356935.1
GCA_028358575.1
GCA_028370115.1
GCA_028370135.1
GCA_028370155.1
GCA_028370175.1
GCA_028370195.1
GCA_028370215.1
GCA_028370235.1
GCA_028370255.1
GCA_028370275.1
GCA_028370295.1
GCA_028403965.1
GCA_028403985.1
GCA_028404005.1
GCA_028404025.1
GCA_028404045.1
GCA_028404065.1
GCA_028404085.1
GCA_028404105.1
GCA_028411375.1
GCA_028411555.1
GCA_028416635.1
GCA_028416655.1
GCA_028416675.1
GCA_028416695.1
GCA_028421465.1
GCA_028421485.1
GCA_028421645.1
GCA_028451225.2
GCA_028463885.1
GCA_028463905.1
GCA_028463925.1

GCA_028463945.1
GCA_028463965.1
GCA_028463985.1
GCA_028464005.1
GCA_028464025.1
GCA_028464045.1
GCA_028464065.1
GCA_028471605.1
GCA_028471885.1
GCA_028471905.1
GCA_028471925.1
GCA_028471945.1
GCA_028471965.1
GCA_028471985.1
GCA_028472205.1
GCA_028472785.1
GCA_028472805.1
GCA_028472825.1
GCA_028472845.1
GCA_028472865.1
GCA_028472885.1
GCA_028472985.1
GCA_028473145.1
GCA_028473305.1
GCA_028473465.1
GCA_028473585.1
GCA_028473605.1
GCA_028473625.1
GCA_028473645.1
GCA_028473665.1
GCA_028473685.1
GCA_028473705.1
GCA_028473725.1
GCA_028473745.1
GCA_028473865.1
GCA_028475085.1
GCA_028475105.1
GCA_028483175.2
GCA_028488865.1
GCA_028532465.1
GCA_028532485.1
GCA_028532685.1
GCA_028532705.1
GCA_028538045.1
GCA_028540325.1
GCA_028540345.1
GCA_028540365.1
GCA_028540385.1
GCA_028540405.1
GCA_028540425.1
GCA_028540445.1
GCA_028540465.1
GCA_028540485.1
GCA_028540505.1

GCA_028540525.1
GCA_028540585.1
GCA_028540725.1
GCA_028540805.1
GCA_028540825.1
GCA_028540845.1
GCA_028540865.1
GCA_028540885.1
GCA_028540905.1
GCA_028540925.1
GCA_028540945.1
GCA_028540965.1
GCA_028540985.1
GCA_028541005.1
GCA_028541045.1
GCA_028541125.1
GCA_028541165.1
GCA_028541185.1
GCA_028541205.1
GCA_028541225.1
GCA_028541245.1
GCA_028541265.1
GCA_028541285.1
GCA_028541305.1
GCA_028541325.1
GCA_028541345.1
GCA_028541365.1
GCA_028541385.1
GCA_028541405.1
GCA_028541425.1
GCA_028541545.1
GCA_028541565.1
GCA_028541585.1
GCA_028551175.1
GCA_028551195.1
GCA_028551215.1
GCA_028551235.1
GCA_028551255.1
GCA_028551275.1
GCA_028551295.1
GCA_028551315.1
GCA_028551585.1
GCA_028553725.1
GCA_028553745.1
GCA_028553765.1
GCA_028553785.1
GCA_028553805.1
GCA_028553885.1
GCA_028553905.1
GCA_028554415.1
GCA_028595525.2
GCA_028595865.2
GCA_028596005.1
GCA_028596025.1

GCA_028596045.1
GCA_028596065.1
GCA_028596085.1
GCA_028596105.1
GCA_028596125.1
GCA_028596145.1
GCA_028596165.1
GCA_028596185.1
GCA_028596205.1
GCA_028596225.1
GCA_028596245.1
GCA_028598965.2
GCA_028606985.1
GCA_028607005.1
GCA_028607025.1
GCA_028607045.1
GCA_028607065.1
GCA_028607085.1
GCA_028607105.1
GCA_028609625.1
GCA_028609665.1
GCA_028609685.1
GCA_028609705.1
GCA_028609725.1
GCA_028609745.1
GCA_028609765.1
GCA_028609785.1
GCA_028609805.1
GCA_028609825.1
GCA_028609845.1
GCA_028609865.1
GCA_028609885.1
GCA_028610225.1
GCA_028621715.1
GCA_028621735.1
GCA_028621755.1
GCA_028621775.1
GCA_028621795.1
GCA_028621815.1
GCA_028621835.1
GCA_028621855.1
GCA_028621875.1
GCA_028621895.1
GCA_028621915.1
GCA_028621935.1
GCA_028621955.1
GCA_028621975.1
GCA_028621995.1
GCA_028622015.1
GCA_028622045.1
GCA_028622095.1
GCA_028622115.1
GCA_028622135.1
GCA_028622155.1

GCA_028622175.1
GCA_028622195.1
GCA_028622215.1
GCA_028622235.1
GCA_028622255.1
GCA_028622275.1
GCA_028622295.1
GCA_028622315.1
GCA_028622335.1
GCA_028622355.1
GCA_028622375.1
GCA_028622395.1
GCA_028622415.1
GCA_028622435.1
GCA_028622455.1
GCA_028622475.1
GCA_028622495.1
GCA_028622515.1
GCA_028622535.1
GCA_028622555.1
GCA_028622575.1
GCA_028622595.1
GCA_028622615.1
GCA_028622635.1
GCA_028622655.1
GCA_028622675.1
GCA_028622695.1
GCA_028622715.1
GCA_028622735.1
GCA_028622755.1
GCA_028622775.1
GCA_028622795.1
GCA_028622815.1
GCA_028622835.1
GCA_028622855.1
GCA_028622875.1
GCA_028622895.1
GCA_028622915.1
GCA_028622935.1
GCA_028622955.1
GCA_028623025.1
GCA_028623045.1
GCA_028623065.1
GCA_028623085.1
GCA_028623105.1
GCA_028623125.1
GCA_028623145.1
GCA_028623165.1
GCA_028623185.1
GCA_028657905.1
GCA_028657925.1
GCA_028657945.1
GCA_028657965.1
GCA_028662055.1

GCA_028735835.1
GCA_028735855.1
GCA_028735875.1
GCA_028735895.1
GCA_028735915.1
GCA_028735935.1
GCA_028735955.1
GCA_028735975.1
GCA_028735995.1
GCA_028736015.1
GCA_028736055.1
GCA_028736095.1
GCA_028736115.1
GCA_028736135.1
GCA_028736155.1
GCA_028736175.1
GCA_028736195.1
GCA_028736215.1
GCA_028736235.1
GCA_028736255.1
GCA_028736275.1
GCA_028736295.1
GCA_028736315.1
GCA_028743095.1
GCA_028743255.1
GCA_028743275.1
GCA_028743295.1
GCA_028743315.1
GCA_028743335.1
GCA_028743355.1
GCA_028743375.1
GCA_028743395.1
GCA_028743415.1
GCA_028743455.1
GCA_028743475.1
GCA_028743495.1
GCA_028743515.1
GCA_028743535.1
GCA_028743555.1
GCA_028743575.1
GCA_028743595.1
GCA_028743615.1
GCA_028743635.1
GCA_028743655.1
GCA_028743675.1
GCA_028743695.1
GCA_028743715.1
GCA_028743735.1
GCA_028743755.1
GCA_028743775.1
GCA_028743795.1
GCA_028747205.1
GCA_028747225.1
GCA_028747245.1

GCA_028747265.1
GCA_028747285.1
GCA_028747305.1
GCA_028747325.1
GCA_028747345.1
GCA_028747365.1
GCA_028747385.1
GCA_028747405.1
GCA_028747425.1
GCA_028747445.1
GCA_028747465.1
GCA_028747485.1
GCA_028747505.1
GCA_028747525.1
GCA_028747545.1
GCA_028747565.1
GCA_028747585.1
GCA_028747605.1
GCA_028747625.1
GCA_028747645.1
GCA_028747665.1
GCA_028747685.1
GCA_028747705.1
GCA_028747725.1
GCA_028747745.1
GCA_028747765.1
GCA_028747805.1
GCA_028747825.1
GCA_028747845.1
GCA_028747865.1
GCA_028747885.1
GCA_028747905.1
GCA_028747925.1
GCA_028747945.1
GCA_028747965.1
GCA_028747985.1
GCA_028748025.1
GCA_028748045.1
GCA_028748065.1
GCA_028748145.1
GCA_028748165.1
GCA_028748185.1
GCA_028748305.1
GCA_028748785.1
GCA_028748805.1
GCA_028748825.1
GCA_028748845.1
GCA_028748865.1
GCA_028749545.1
GCA_028749565.1
GCA_028749585.1
GCA_028749605.1
GCA_028749625.1
GCA_028749645.1

GCA_028749665.1
GCA_028749685.1
GCA_028749705.1
GCA_028749725.1
GCA_028749745.1
GCA_028749765.1
GCA_028749785.1
GCA_028749805.1
GCA_028749825.1
GCA_028749845.1
GCA_028749865.1
GCA_028749885.1
GCA_028749905.1
GCA_028749925.1
GCA_028749945.1
GCA_028750015.1
GCA_028750035.1
GCA_028750055.1
GCA_028750075.1
GCA_028750095.1
GCA_028750115.1
GCA_028750135.1
GCA_028750155.1
GCA_028750275.1
GCA_028750335.1
GCA_028750355.1
GCA_028750375.1
GCA_028750395.1
GCA_028750415.1
GCA_028750435.1
GCA_028750455.1
GCA_028750515.1
GCA_028750535.1
GCA_028750555.1
GCA_028750575.1
GCA_028750595.1
GCA_028750615.1
GCA_028750635.1
GCA_028750655.1
GCA_028751155.1
GCA_028751175.1
GCA_028751195.1
GCA_028751215.1
GCA_028751235.1
GCA_028751255.1
GCA_028751275.1
GCA_028751295.1
GCA_028751315.1
GCA_028751335.1
GCA_028751355.1
GCA_028751375.1
GCA_028751395.1
GCA_028751435.1
GCA_028751465.1

GCA_028751505.1
GCA_028751525.1
GCA_028751545.1
GCA_028751565.1
GCA_028751585.1
GCA_028751625.1
GCA_028751645.1
GCA_028751665.1
GCA_028751705.1
GCA_028751785.1
GCA_028751835.1
GCA_028751975.1
GCA_028752015.1
GCA_028752035.1
GCA_028752055.1
GCA_028752075.1
GCA_028752095.1
GCA_028752115.1
GCA_028752155.1
GCA_028752235.1
GCA_028752255.1
GCA_028752275.1
GCA_028752295.1
GCA_028752315.1
GCA_028752335.1
GCA_028752355.1
GCA_028752375.1
GCA_028752395.1
GCA_028752415.1
GCA_028752435.1
GCA_028752455.1
GCA_028752475.1
GCA_028752495.1
GCA_028752515.1
GCA_028752535.1
GCA_028752555.1
GCA_028752615.1
GCA_028752635.1
GCA_028752655.1
GCA_028752675.1
GCA_028752695.1
GCA_028752715.1
GCA_028752735.1
GCA_028752775.1
GCA_028752795.1
GCA_028752815.1
GCA_028752835.1
GCA_028752875.1
GCA_028753015.1
GCA_028753275.1
GCA_028753295.1
GCA_028768405.1
GCA_028768425.1
GCA_028768445.1

GCA_028768465.1
GCA_028768485.1
GCA_028768505.1
GCA_028768525.1
GCA_028768545.1
GCA_028771845.1
GCA_028831855.1
GCA_028856065.1
GCA_028856185.1
GCA_028856405.1
GCA_028856425.1
GCA_028864635.1
GCA_028864795.1
GCA_028864855.1
GCA_028865045.1
GCA_028865235.1
GCA_028865455.1
GCA_028867175.1
GCA_028867355.1
GCA_028867535.1
GCA_028867705.1
GCA_028867825.1
GCA_028868015.1
GCA_028868155.1
GCA_028868275.1
GCA_028868465.1
GCA_028868595.1
GCA_028868745.1
GCA_028868935.1
GCA_028869115.1
GCA_028869445.1
GCA_028870195.1
GCA_028870335.1
GCA_028870515.1
GCA_028871695.1
GCA_028872175.1
GCA_028872195.1
GCA_028872215.1
GCA_028872235.1
GCA_028877475.1
GCA_028884855.1
GCA_028884875.1
GCA_028884895.1
GCA_028885375.1
GCA_028885395.1
GCA_028885415.1
GCA_028885435.1
GCA_028885455.1
GCA_028885545.1
GCA_028885565.1
GCA_028885585.1
GCA_028885605.1
GCA_028891345.1
GCA_028891365.1

GCA_028891385.1
GCA_028891405.1
GCA_028891425.1
GCA_028891445.1
GCA_028891465.1
GCA_028891505.1
GCA_028891525.1
GCA_028892635.1
GCA_028892665.1
GCA_028892685.1
GCA_028892705.1
GCA_028892725.1
GCA_028892745.1
GCA_028892765.1
GCA_028892785.1
GCA_028892805.1
GCA_028892825.1
GCA_028892845.1
GCA_028892865.1
GCA_028892885.1
GCA_028892935.1
GCA_028892955.1
GCA_028892975.1
GCA_028892995.1
GCA_028893015.1
GCA_028893035.1
GCA_028893055.1
GCA_028893075.1
GCA_028893095.1
GCA_028893115.1
GCA_028893135.1
GCA_028893155.1
GCA_028893175.1
GCA_028893195.1
GCA_028893215.1
GCA_028893235.1
GCA_028893255.1
GCA_028893275.1
GCA_028893295.1
GCA_028893315.1
GCA_028893335.1
GCA_028893355.1
GCA_028893375.1
GCA_028893395.1
GCA_028893415.1
GCA_028893435.1
GCA_028893455.1
GCA_028893475.1
GCA_028898625.1
GCA_028898645.1
GCA_028898665.1
GCA_028898685.1
GCA_028898705.1
GCA_028898725.1

GCA_028898745.1
GCA_028898765.1
GCA_028898785.1
GCA_028898805.1
GCA_028898825.1
GCA_028898845.1
GCA_028898865.1
GCA_028898885.1
GCA_028898905.1
GCA_028898925.1
GCA_028898945.1
GCA_028961985.1
GCA_028994055.1
GCA_028994075.1
GCA_028994095.1
GCA_028994115.1
GCA_028994155.1
GCA_028994175.1
GCA_028994195.1
GCA_028994215.1
GCA_028994235.1
GCA_028994255.1
GCA_028994275.1
GCA_029000325.1
GCA_029000405.1
GCA_029000425.1
GCA_029001825.1
GCA_029010215.1
GCA_029010235.1
GCA_029010255.1
GCA_029010275.1
GCA_029011155.1
GCA_029011175.1
GCA_029011195.1
GCA_029011215.1
GCA_029011235.1
GCA_029011255.1
GCA_029011275.1
GCA_029011295.1
GCA_029011355.1
GCA_029011395.1
GCA_029011415.1
GCA_029011455.1
GCA_029011475.1
GCA_029011495.1
GCA_029011515.1
GCA_029011535.1
GCA_029011575.1
GCA_029011595.1
GCA_029011615.1
GCA_029011635.1
GCA_029011675.1
GCA_029011695.1
GCA_029011725.1

GCA_029011745.1
GCA_029011765.1
GCA_029011785.1
GCA_029011805.1
GCA_029011825.1
GCA_029011845.1
GCA_029011865.1
GCA_029011885.1
GCA_029011905.1
GCA_029011945.1
GCA_029011965.1
GCA_029011985.1
GCA_029012025.1
GCA_029016125.1
GCA_029016285.1
GCA_029016485.1
GCA_029016665.1
GCA_029023665.1
GCA_029023725.1
GCA_029023745.1
GCA_029023805.1
GCA_029026145.1
GCA_029026505.1
GCA_029027905.1
GCA_029027925.1
GCA_029027945.1
GCA_029028125.1
GCA_029030725.1
GCA_029094145.1
GCA_029094305.1
GCA_029094485.1
GCA_029094505.1
GCA_029094525.1
GCA_029094545.1
GCA_029101565.1
GCA_029101585.1
GCA_029167565.1
GCA_029167585.1
GCA_029167605.1
GCA_029167625.1
GCA_029167645.1
GCA_029167665.1
GCA_029167685.1
GCA_029167705.1
GCA_029167725.1
GCA_029168275.1
GCA_029168355.1
GCA_029168375.1
GCA_029168395.1
GCA_029168415.1
GCA_029168435.1
GCA_029168455.1
GCA_029168475.1
GCA_029168495.1

GCA_029168515.1
GCA_029168535.1
GCA_029168555.1
GCA_029168575.1
GCA_029168595.1
GCA_029201205.1
GCA_029201225.1
GCA_029201245.1
GCA_029201265.1
GCA_029201285.1
GCA_029201425.1
GCA_029201445.1
GCA_029201465.1
GCA_029201485.1
GCA_029201505.1
GCA_029201525.1
GCA_029201545.1
GCA_029201565.1
GCA_029201605.1
GCA_029201625.1
GCA_029201645.1
GCA_029201665.1
GCA_029202345.1
GCA_029202365.1
GCA_029202385.1
GCA_029202405.1
GCA_029202425.1
GCA_029202445.1
GCA_029202465.1
GCA_029202485.1
GCA_029202505.1
GCA_029202525.1
GCA_029202545.1
GCA_029202665.1
GCA_029202845.1
GCA_029202985.1
GCA_029203105.1
GCA_029203125.1
GCA_029203145.1
GCA_029203165.1
GCA_029203185.1
GCA_029203205.1
GCA_029203225.1
GCA_029203285.1
GCA_029203915.1
GCA_029203935.1
GCA_029203955.1
GCA_029204095.1
GCA_029204125.1
GCA_029204145.1
GCA_029204165.1
GCA_029204245.1
GCA_029204265.1
GCA_029204285.1

GCA_029215525.1
GCA_029215545.1
GCA_029215565.1
GCA_029215585.1
GCA_029215735.1
GCA_029215815.1
GCA_029215835.1
GCA_029215855.1
GCA_029215875.1
GCA_029215895.1
GCA_029215915.1
GCA_029219945.1
GCA_029219965.1
GCA_029219985.1
GCA_029220025.1
GCA_029220045.1
GCA_029220065.1
GCA_029220085.1
GCA_029223545.1
GCA_029223565.1
GCA_029223585.1
GCA_029223605.1
GCA_029223625.1
GCA_029223645.1
GCA_029223665.1
GCA_029223685.1
GCA_029223705.1
GCA_029223725.1
GCA_029223865.1
GCA_029223885.1
GCA_029223905.1
GCA_029223925.1
GCA_029223945.1
GCA_029223965.1
GCA_029223985.1
GCA_029224005.1
GCA_029224025.1
GCA_029224045.1
GCA_029224065.1
GCA_029224085.1
GCA_029224105.1
GCA_029224125.1
GCA_029227755.1
GCA_029227785.1
GCA_029227815.1
GCA_029227835.1
GCA_029227855.1
GCA_029227875.1
GCA_029227895.1
GCA_029229465.1
GCA_029229485.1
GCA_029229505.1
GCA_029277405.1
GCA_029277425.1

GCA_029277445.1
GCA_029277485.1
GCA_029277625.1
GCA_029277825.1
GCA_029277985.1
GCA_029278185.1
GCA_029278325.1
GCA_029278505.1
GCA_029278525.1
GCA_029278545.1
GCA_029278585.1
GCA_029278605.1
GCA_029278625.1
GCA_029280505.1
GCA_029280565.1
GCA_029280585.1
GCA_029282585.1
GCA_029282725.1
GCA_029318635.1
GCA_029318685.1
GCA_029318875.1
GCA_029318935.1
GCA_029318975.1
GCA_029319035.1
GCA_029319085.1
GCA_029319145.1
GCA_029319185.1
GCA_029320885.1
GCA_029320915.1
GCA_029320935.1
GCA_029320955.1
GCA_029320975.1
GCA_029320995.1
GCA_029321015.1
GCA_029323775.1
GCA_029323795.1
GCA_029338155.1
GCA_029338175.1
GCA_029338415.1
GCA_029338475.1
GCA_029339075.1
GCA_029339095.1
GCA_029369645.1
GCA_029369685.1
GCA_029369705.1
GCA_029369725.1
GCA_029369745.1
GCA_029369765.1
GCA_029369785.1
GCA_029371965.1
GCA_029371985.1
GCA_029391755.1
GCA_029391915.1
GCA_029391995.1

GCA_029392015.1
GCA_029392035.1
GCA_029392055.1
GCA_029392075.1
GCA_029392095.1
GCA_029392135.1
GCA_029392315.1
GCA_029392475.1
GCA_029392675.1
GCA_029392815.1
GCA_029392835.1
GCA_029392855.1
GCA_029392875.1
GCA_029392895.1
GCA_029392915.1
GCA_029392935.1
GCA_029392955.1
GCA_029392975.1
GCA_029393075.1
GCA_029393235.1
GCA_029395355.1
GCA_029395415.1
GCA_029395435.1
GCA_029395455.1
GCA_029395475.1
GCA_029395495.1
GCA_029395515.1
GCA_029395535.1
GCA_029395555.1
GCA_029395675.1
GCA_029398215.1
GCA_029448435.1
GCA_029457395.1
GCA_029457415.1
GCA_029457435.1
GCA_029457455.1
GCA_029457475.1
GCA_029457495.1
GCA_029457515.1
GCA_029457555.1
GCA_029457575.1
GCA_029457595.1
GCA_029457615.1
GCA_029457635.1
GCA_029457675.1
GCA_029459615.1
GCA_029459635.1
GCA_029459655.1
GCA_029459755.1
GCA_029459775.1
GCA_029492705.1
GCA_029495675.1
GCA_029510815.1
GCA_029536615.1

GCA_029536635.1
GCA_029536655.1
GCA_029536675.1
GCA_029536695.1
GCA_029536715.1
GCA_029536735.1
GCA_029536755.1
GCA_029536775.1
GCA_029536795.1
GCA_029536815.1
GCA_029536835.1
GCA_029536855.1
GCA_029536875.1
GCA_029536895.1
GCA_029536915.1
GCA_029536935.1
GCA_029536955.1
GCA_029536975.1
GCA_029536995.1
GCA_029537015.1
GCA_029537035.1
GCA_029537055.1
GCA_029537075.1
GCA_029537095.1
GCA_029537115.1
GCA_029537135.1
GCA_029537155.1
GCA_029537175.1
GCA_029537195.1
GCA_029537215.1
GCA_029537235.1
GCA_029537255.1
GCA_029537275.1
GCA_029537295.1
GCA_029537315.1
GCA_029537335.1
GCA_029537355.1
GCA_029537375.1
GCA_029537395.1
GCA_029537415.1
GCA_029541525.1
GCA_029541585.1
GCA_029541605.1
GCA_029541625.1
GCA_029541645.1
GCA_029541665.1
GCA_029541685.1
GCA_029541705.1
GCA_029541725.1
GCA_029541745.1
GCA_029541765.1
GCA_029541785.1
GCA_029541805.1
GCA_029541825.1

GCA_029541845.1
GCA_029541865.1
GCA_029541885.1
GCA_029541905.1
GCA_029541925.1
GCA_029541965.1
GCA_029541985.1
GCA_029542025.1
GCA_029542045.1
GCA_029542065.1
GCA_029542125.1
GCA_029542145.1
GCA_029542165.1
GCA_029542185.1
GCA_029542205.1
GCA_029542225.1
GCA_029542245.1
GCA_029542305.1
GCA_029542325.1
GCA_029542345.1
GCA_029542365.1
GCA_029542385.1
GCA_029542405.1
GCA_029542425.1
GCA_029542445.1
GCA_029542465.1
GCA_029542485.1
GCA_029542505.1
GCA_029542525.1
GCA_029542545.1
GCA_029542565.1
GCA_029542585.1
GCA_029542625.1
GCA_029542645.1
GCA_029542685.1
GCA_029542705.1
GCA_029542725.1
GCA_029542745.1
GCA_029543005.1
GCA_029543025.1
GCA_029543045.1
GCA_029543065.1
GCA_029543085.1
GCA_029580675.1
GCA_029580695.1
GCA_029580715.1
GCA_029580735.1
GCA_029580755.1
GCA_029580775.1
GCA_029580795.1
GCA_029580815.1
GCA_029580835.1
GCA_029580895.1
GCA_029580915.1

GCA_029580935.1
GCA_029580955.1
GCA_029580975.1
GCA_029580995.1
GCA_029581015.1
GCA_029581035.1
GCA_029581055.1
GCA_029581075.1
GCA_029581095.1
GCA_029581115.1
GCA_029581155.1
GCA_029581335.1
GCA_029581375.1
GCA_029581395.1
GCA_029581515.1
GCA_029581575.1
GCA_029582015.1
GCA_029590275.1
GCA_029590295.1
GCA_029590315.1
GCA_029590335.1
GCA_029590355.1
GCA_029590375.1
GCA_029590395.1
GCA_029590415.1
GCA_029590435.1
GCA_029590455.1
GCA_029590475.1
GCA_029590495.1
GCA_029590515.1
GCA_029590535.1
GCA_029590555.1
GCA_029591745.1
GCA_029591825.1
GCA_029591945.1
GCA_029592045.1
GCA_029592105.1
GCA_029592185.1
GCA_029593895.1
GCA_029593915.1
GCA_029594695.1
GCA_029607405.1
GCA_029607425.1
GCA_029607445.1
GCA_029624875.1
GCA_029624895.1
GCA_029624915.1
GCA_029624935.1
GCA_029624955.1
GCA_029624975.1
GCA_029624995.1
GCA_029625015.1
GCA_029625035.1
GCA_029625055.1

GCA_029625075.1
GCA_029625095.1
GCA_029625115.1
GCA_029625135.1
GCA_029625155.1
GCA_029625175.1
GCA_029625195.1
GCA_029625215.1
GCA_029625235.1
GCA_029625255.1
GCA_029625275.1
GCA_029625295.1
GCA_029625315.1
GCA_029625335.1
GCA_029625355.1
GCA_029625375.1
GCA_029625395.1
GCA_029625415.1
GCA_029625435.1
GCA_029625455.1
GCA_029625475.1
GCA_029625495.1
GCA_029632585.1
GCA_029632765.1
GCA_029632925.1
GCA_029632985.1
GCA_029633005.1
GCA_029633245.1
GCA_029633425.1
GCA_029633645.1
GCA_029633805.1
GCA_029633825.1
GCA_029633895.1
GCA_029633935.1
GCA_029633985.1
GCA_029634015.1
GCA_029634055.1
GCA_029634665.1
GCA_029634685.1
GCA_029634715.1
GCA_029634735.1
GCA_029634765.1
GCA_029634785.1
GCA_029634905.1
GCA_029635105.1
GCA_029635575.1
GCA_029635775.1
GCA_029635795.1
GCA_029635815.1
GCA_029635835.1
GCA_029635865.1
GCA_029635885.1
GCA_029636225.1
GCA_029636405.1

GCA_029636605.1
GCA_029636705.1
GCA_029636725.1
GCA_029636745.1
GCA_029636765.1
GCA_029636785.1
GCA_029636805.1
GCA_029636825.1
GCA_029636845.1
GCA_029636965.1
GCA_029637165.1
GCA_029637325.1
GCA_029637605.1
GCA_029637645.1
GCA_029637705.1
GCA_029637825.1
GCA_029639405.1
GCA_029639525.1
GCA_029639565.1
GCA_029643245.1
GCA_029643425.1
GCA_029674545.1
GCA_029674565.1
GCA_029674605.1
GCA_029674625.1
GCA_029674645.1
GCA_029674665.1
GCA_029674685.1
GCA_029674705.1
GCA_029674725.1
GCA_029674745.1
GCA_029674765.1
GCA_029674785.1
GCA_029674805.1
GCA_029674825.1
GCA_029674845.1
GCA_029677405.1
GCA_029677665.1
GCA_029677685.1
GCA_029677705.1
GCA_029677725.1
GCA_029677745.1
GCA_029677885.1
GCA_029677985.1
GCA_029678005.1
GCA_029678025.1
GCA_029689925.1
GCA_029689945.1
GCA_029689965.1
GCA_029689985.1
GCA_029690025.1
GCA_029690045.1
GCA_029690065.1
GCA_029690085.1

GCA_029690105.1
GCA_029690245.1
GCA_029690425.1
GCA_029690525.1
GCA_029691405.1
GCA_029691425.1
GCA_029691445.1
GCA_029691625.1
GCA_029691645.1
GCA_029691665.1
GCA_029691685.1
GCA_029691705.1
GCA_029691725.1
GCA_029691745.1
GCA_029691775.1
GCA_029691795.1
GCA_029691815.1
GCA_029691835.1
GCA_029691855.1
GCA_029691875.1
GCA_029691895.1
GCA_029691915.1
GCA_029691935.1
GCA_029691955.1
GCA_029691985.1
GCA_029692005.1
GCA_029692025.1
GCA_029692065.1
GCA_029692105.1
GCA_029713745.1
GCA_029713765.1
GCA_029713785.1
GCA_029713845.1
GCA_029713865.1
GCA_029713905.1
GCA_029713925.1
GCA_029713945.1
GCA_029713965.1
GCA_029714005.1
GCA_029714045.1
GCA_029714085.1
GCA_029714105.1
GCA_029714145.1
GCA_029714165.1
GCA_029714185.1
GCA_029714205.1
GCA_029714225.1
GCA_029714245.1
GCA_029714265.1
GCA_029714285.1
GCA_029714305.1
GCA_029714325.1
GCA_029714345.1
GCA_029714365.1

GCA_029714385.1
GCA_029714405.1
GCA_029714425.1
GCA_029714545.1
GCA_029714945.1
GCA_029715105.1
GCA_029715665.1
GCA_029716005.1
GCA_029716165.1
GCA_029716315.1
GCA_029716485.1
GCA_029716845.1
GCA_029717145.1
GCA_029717425.1
GCA_029717445.1
GCA_029717465.1
GCA_029717485.1
GCA_029717585.1
GCA_029717665.1
GCA_029717865.1
GCA_029717885.1
GCA_029717905.1
GCA_029717925.1
GCA_029717945.1
GCA_029717985.1
GCA_029718025.1
GCA_029718045.1
GCA_029718135.1
GCA_029718255.1
GCA_029718275.1
GCA_029718295.1
GCA_029718315.1
GCA_029718425.1
GCA_029718465.1
GCA_029718585.1
GCA_029718635.1
GCA_029718665.1
GCA_029718685.1
GCA_029718885.1
GCA_029718965.1
GCA_029719125.1
GCA_029719185.1
GCA_029719205.1
GCA_029719225.1
GCA_029719265.1
GCA_029719365.1
GCA_029719485.1
GCA_029719585.1
GCA_029719765.1
GCA_029719805.1
GCA_029719845.1
GCA_029719865.1
GCA_029719885.1
GCA_029719985.1

GCA_029720005.1
GCA_029720995.1
GCA_029721015.1
GCA_029721335.1
GCA_029726355.1
GCA_029743055.1
GCA_029748815.1
GCA_029761895.1
GCA_029761915.1
GCA_029761935.1
GCA_029761955.1
GCA_029761975.1
GCA_029761995.1
GCA_029762015.1
GCA_029762035.1
GCA_029762055.1
GCA_029762075.1
GCA_029762095.1
GCA_029762115.1
GCA_029762135.1
GCA_029762155.1
GCA_029762175.1
GCA_029762195.1
GCA_029762215.1
GCA_029762235.1
GCA_029762255.1
GCA_029762275.1
GCA_029762295.1
GCA_029762315.1
GCA_029762335.1
GCA_029762355.1
GCA_029762375.1
GCA_029762395.1
GCA_029762415.1
GCA_029762435.1
GCA_029762455.1
GCA_029762475.1
GCA_029772985.1
GCA_029773005.1
GCA_029773025.1
GCA_029773045.1
GCA_029773065.1
GCA_029773085.1
GCA_029773105.1
GCA_029773225.1
GCA_029773245.1
GCA_029773265.1
GCA_029773285.1
GCA_029773305.1
GCA_029773325.1
GCA_029773345.1
GCA_029773365.1
GCA_029773385.1
GCA_029773405.1

GCA_029773425.1
GCA_029773445.1
GCA_029773465.1
GCA_029773485.1
GCA_029773505.1
GCA_029773525.1
GCA_029773545.1
GCA_029773565.1
GCA_029773585.1
GCA_029773605.1
GCA_029773625.1
GCA_029773645.1
GCA_029773665.1
GCA_029773685.1
GCA_029773705.1
GCA_029773725.1
GCA_029773745.1
GCA_029773765.1
GCA_029773785.1
GCA_029773835.1
GCA_029773855.1
GCA_029773875.1
GCA_029773895.1
GCA_029773915.1
GCA_029773935.1
GCA_029773955.1
GCA_029773975.1
GCA_029773995.1
GCA_029774015.1
GCA_029774035.1
GCA_029774055.1
GCA_029774075.1
GCA_029774095.1
GCA_029774115.1
GCA_029774135.1
GCA_029774155.1
GCA_029774175.1
GCA_029774195.1
GCA_029774215.1
GCA_029774235.1
GCA_029774255.1
GCA_029774275.1
GCA_029774295.1
GCA_029774315.1
GCA_029774335.1
GCA_029774355.1
GCA_029775435.1
GCA_029783395.1
GCA_029814215.1
GCA_029814235.1
GCA_029814255.1
GCA_029814785.1
GCA_029814805.1
GCA_029814825.1

GCA_029814845.1
GCA_029814875.1
GCA_029814895.1
GCA_029814915.1
GCA_029814935.1
GCA_029814955.1
GCA_029814975.1
GCA_029814995.1
GCA_029815015.1
GCA_029815035.1
GCA_029834415.1
GCA_029834455.1
GCA_029834475.1
GCA_029834495.1
GCA_029834515.1
GCA_029834565.1
GCA_029834585.1
GCA_029834605.1
GCA_029834625.1
GCA_029849135.1
GCA_029849155.1
GCA_029849175.1
GCA_029850785.1
GCA_029851525.1
GCA_029851545.1
GCA_029851565.1
GCA_029851585.1
GCA_029851605.1
GCA_029851625.1
GCA_029854175.1
GCA_029854195.1
GCA_029854215.1
GCA_029854235.1
GCA_029854255.1
GCA_029854275.1
GCA_029854295.1
GCA_029854315.1
GCA_029854335.1
GCA_029854355.1
GCA_029854375.1
GCA_029854395.1
GCA_029854435.1
GCA_029854455.1
GCA_029854475.1
GCA_029854495.1
GCA_029854515.1
GCA_029854535.1
GCA_029854555.1
GCA_029854575.1
GCA_029854595.1
GCA_029854615.1
GCA_029854965.1
GCA_029854985.1
GCA_029855005.1

GCA_029855025.1
GCA_029855045.1
GCA_029855065.1
GCA_029855085.1
GCA_029855105.1
GCA_029855125.1
GCA_029855145.1
GCA_029855165.1
GCA_029855345.1
GCA_029855445.1
GCA_029855465.1
GCA_029855485.1
GCA_029855505.1
GCA_029855525.1
GCA_029855545.1
GCA_029855565.1
GCA_029855585.1
GCA_029855605.1
GCA_029855645.1
GCA_029855665.1
GCA_029855685.1
GCA_029855705.1
GCA_029855725.1
GCA_029855745.1
GCA_029855765.1
GCA_029855785.1
GCA_029855805.1
GCA_029855825.1
GCA_029855845.1
GCA_029855885.1
GCA_029855905.1
GCA_029856955.1
GCA_029856995.1
GCA_029857015.1
GCA_029866425.1
GCA_029866445.1
GCA_029866465.1
GCA_029866485.1
GCA_029866505.1
GCA_029866525.1
GCA_029866565.1
GCA_029866585.1
GCA_029866625.1
GCA_029866705.1
GCA_029866725.1
GCA_029866785.1
GCA_029866805.1
GCA_029866825.1
GCA_029866845.1
GCA_029866885.1
GCA_029866905.1
GCA_029866925.1
GCA_029866945.1
GCA_029866965.1

GCA_029867025.1
GCA_029867045.1
GCA_029867065.1
GCA_029867085.1
GCA_029867105.1
GCA_029867125.1
GCA_029867145.1
GCA_029867165.1
GCA_029867185.1
GCA_029867205.1
GCA_029867225.1
GCA_029867245.1
GCA_029867265.1
GCA_029869925.1
GCA_029872175.2
GCA_029872195.2
GCA_029872215.2
GCA_029872235.2
GCA_029872245.2
GCA_029873195.1
GCA_029873215.1
GCA_029873235.1
GCA_029873255.1
GCA_029873295.1
GCA_029873315.1
GCA_029873335.1
GCA_029873355.1
GCA_029873375.1
GCA_029873395.1
GCA_029873415.1
GCA_029873435.1
GCA_029873455.1
GCA_029873475.1
GCA_029873495.1
GCA_029873515.1
GCA_029873535.1
GCA_029873555.1
GCA_029873575.1
GCA_029889725.1
GCA_029889745.1
GCA_029891455.2
GCA_029891475.2
GCA_029891485.2
GCA_029906405.1
GCA_029906425.1
GCA_029906445.1
GCA_029906465.1
GCA_029906485.1
GCA_029906505.1
GCA_029906525.1
GCA_029906565.1
GCA_029909395.1
GCA_029909415.1
GCA_029909435.1

GCA_029909455.1
GCA_029909475.1
GCA_029909495.1
GCA_029909515.1
GCA_029909535.1
GCA_029909555.1
GCA_029909575.1
GCA_029909595.1
GCA_029909615.1
GCA_029909635.1
GCA_029909655.1
GCA_029909675.1
GCA_029909695.1
GCA_029909715.1
GCA_029909735.1
GCA_029909755.1
GCA_029909775.1
GCA_029909795.1
GCA_029909955.1
GCA_029909975.1
GCA_029909995.1
GCA_029910015.1
GCA_029910035.1
GCA_029910055.1
GCA_029910075.1
GCA_029910095.1
GCA_029910115.1
GCA_029910135.1
GCA_029910175.1
GCA_029910215.1
GCA_029910235.1
GCA_029910255.1
GCA_029910275.1
GCA_029910295.1
GCA_029910315.1
GCA_029910335.1
GCA_029910355.1
GCA_029910375.1
GCA_029910395.1
GCA_029910415.1
GCA_029910435.1
GCA_029910455.1
GCA_029910475.1
GCA_029910495.1
GCA_029910535.1
GCA_029910655.1
GCA_029916805.1
GCA_029916825.1
GCA_029916845.1
GCA_029916865.1
GCA_029916885.1
GCA_029916905.1
GCA_029916925.1
GCA_029916945.1

GCA_029916965.1
GCA_029916985.1
GCA_029917005.1
GCA_029917025.1
GCA_029917045.1
GCA_029917065.1
GCA_029917085.1
GCA_029917105.1
GCA_029919115.1
GCA_029919255.1
GCA_029931545.1
GCA_029948145.1
GCA_029948165.1
GCA_029948185.1
GCA_029952745.1
GCA_029952765.1
GCA_029952785.1
GCA_029952805.1
GCA_029952845.1
GCA_029953515.1
GCA_029953555.1
GCA_029953575.1
GCA_029953595.1
GCA_029953615.1
GCA_029953635.1
GCA_029953655.1
GCA_029958325.1
GCA_029958345.1
GCA_029958365.1
GCA_029958385.1
GCA_029958885.1
GCA_029958905.1
GCA_029958925.1
GCA_029961225.1
GCA_029961245.1
GCA_029961265.1
GCA_029961285.1
GCA_029961305.1
GCA_029961325.1
GCA_029961345.1
GCA_029961365.1
GCA_029961385.1
GCA_029961405.1
GCA_029961425.1
GCA_029961445.1
GCA_029961465.1
GCA_029961485.1
GCA_029961505.1
GCA_029961525.1
GCA_029961545.1
GCA_029961565.1
GCA_029961585.1
GCA_029961605.1
GCA_029961625.1

GCA_029961645.1
GCA_029961665.1
GCA_029961705.1
GCA_029961725.1
GCA_029961745.1
GCA_029961765.1
GCA_029961805.1
GCA_029962025.1
GCA_029962045.1
GCA_029962065.1
GCA_029962085.1
GCA_029962105.1
GCA_029962145.1
GCA_029962165.1
GCA_029962185.1
GCA_029962205.1
GCA_029962225.1
GCA_029962245.1
GCA_029962265.1
GCA_029962285.1
GCA_029962305.1
GCA_029962325.1
GCA_029962345.1
GCA_029962365.1
GCA_029962385.1
GCA_029962405.1
GCA_029962425.1
GCA_029962445.1
GCA_029962465.1
GCA_029962485.1
GCA_029962545.1
GCA_029962565.1
GCA_029962585.1
GCA_029962605.1
GCA_029962625.1
GCA_029964055.1
GCA_029972135.1
GCA_029972225.1
GCA_029981845.1
GCA_029982035.1
GCA_029983095.1
GCA_029987435.1
GCA_029987615.1
GCA_029990625.1
GCA_029991185.1
GCA_029991195.1
GCA_030007135.1
GCA_030007315.1
GCA_030007455.1
GCA_030007575.1
GCA_030007795.1
GCA_030007975.1
GCA_030008055.1
GCA_030011535.1

GCA_030011555.1
GCA_030011575.1
GCA_030011595.1
GCA_030011615.1
GCA_030011645.1
GCA_030012525.1
GCA_030012545.1
GCA_030012565.1
GCA_030012585.1
GCA_030012605.1
GCA_030012625.1
GCA_030012645.1
GCA_030012665.1
GCA_030012685.1
GCA_030012745.1
GCA_030012765.1
GCA_030012785.1
GCA_030012805.1
GCA_030012825.1
GCA_030012845.1
GCA_030012905.1
GCA_030012925.1
GCA_030012945.1
GCA_030013005.1
GCA_030013025.1
GCA_030013045.1
GCA_030013085.1
GCA_030013105.1
GCA_030013125.1
GCA_030013145.1
GCA_030013165.1
GCA_030013185.1
GCA_030013205.1
GCA_030013225.1
GCA_030013245.1
GCA_030013265.1
GCA_030013285.1
GCA_030013305.1
GCA_030013325.1
GCA_030013345.1
GCA_030013365.1
GCA_030013385.1
GCA_030013405.1
GCA_030013435.1
GCA_030013455.1
GCA_030013475.1
GCA_030013495.1
GCA_030013515.1
GCA_030013535.1
GCA_030013555.1
GCA_030013575.1
GCA_030013595.1
GCA_030013615.1
GCA_030013655.1

GCA_030013675.1
GCA_030013695.1
GCA_030013715.1
GCA_030013735.1
GCA_030013755.1
GCA_030013785.1
GCA_030013805.1
GCA_030013825.1
GCA_030013845.1
GCA_030013865.1
GCA_030013885.1
GCA_030013905.1
GCA_030013925.1
GCA_030013945.1
GCA_030013965.1
GCA_030013985.1
GCA_030014005.1
GCA_030014025.1
GCA_030014045.1
GCA_030014065.1
GCA_030014085.1
GCA_030014105.1
GCA_030015595.1
GCA_030020155.1
GCA_030020175.1
GCA_030020195.1
GCA_030020215.1
GCA_030020235.1
GCA_030020255.1
GCA_030020345.1
GCA_030020365.1
GCA_030020405.1
GCA_030020425.1
GCA_030020455.1
GCA_030020475.1
GCA_030020495.1
GCA_030020515.1
GCA_030020535.1
GCA_030020555.1
GCA_030020575.1
GCA_030020595.1
GCA_030020615.1
GCA_030020645.1
GCA_030020665.1
GCA_030020685.1
GCA_030020705.1
GCA_030020725.1
GCA_030020745.1
GCA_030020765.1
GCA_030020785.1
GCA_030020825.1
GCA_030020855.1
GCA_030020875.1
GCA_030020895.1

GCA_030020925.1
GCA_030027965.1
GCA_030027985.1
GCA_030028005.1
GCA_030028115.1
GCA_030028135.1
GCA_030028155.1
GCA_030028175.1
GCA_030028195.1
GCA_030028215.1
GCA_030028235.1
GCA_030028255.1
GCA_030028275.1
GCA_030028335.1
GCA_030028355.1
GCA_030028375.1
GCA_030028395.1
GCA_030028615.1
GCA_030028635.1
GCA_030034615.1
GCA_030034635.1
GCA_030034655.1
GCA_030034675.1
GCA_030034695.1
GCA_030034715.1
GCA_030034775.1
GCA_030035205.1
GCA_030035225.1
GCA_030035245.1
GCA_030035265.1
GCA_030035285.1
GCA_030035305.1
GCA_030035325.1
GCA_030035345.1
GCA_030035365.1
GCA_030035385.1
GCA_030035405.1
GCA_030035425.1
GCA_030035445.1
GCA_030035465.1
GCA_030035485.1
GCA_030035505.1
GCA_030035525.1
GCA_030035545.1
GCA_030035565.1
GCA_030035605.1
GCA_030035755.1
GCA_030036515.1
GCA_030036535.1
GCA_030036655.1
GCA_030036675.1
GCA_030036695.1
GCA_030036715.1
GCA_030036755.1

GCA_030036775.1
GCA_030036795.1
GCA_030036835.1
GCA_030036855.1
GCA_030036875.1
GCA_030036895.1
GCA_030036925.1
GCA_030037135.1
GCA_030037255.1
GCA_030037445.1
GCA_030037615.1
GCA_030037835.1
GCA_030037975.1
GCA_030038515.1
GCA_030038665.1
GCA_030038915.1
GCA_030039075.1
GCA_030039225.1
GCA_030039325.1
GCA_030039345.1
GCA_030039375.1
GCA_030039395.1
GCA_030039425.1
GCA_030053035.1
GCA_030053095.1
GCA_030053115.1
GCA_030053135.1
GCA_030053155.1
GCA_030053175.1
GCA_030053195.1
GCA_030053615.1
GCA_030053635.1
GCA_030053655.1
GCA_030053675.1
GCA_030053735.1
GCA_030053755.1
GCA_030053775.1
GCA_030053795.1
GCA_030053815.1
GCA_030053835.1
GCA_030053855.1
GCA_030053875.1
GCA_030053895.1
GCA_030059805.1
GCA_030059825.1
GCA_030059845.1
GCA_030060435.1
GCA_030060455.1
GCA_030060515.1
GCA_030060535.1
GCA_030060555.1
GCA_030060575.1
GCA_030060595.1
GCA_030060615.1

GCA_030060635.1
GCA_030060655.1
GCA_030060695.1
GCA_030060725.1
GCA_030060745.1
GCA_030060765.1
GCA_030060785.1
GCA_030060805.1
GCA_030060825.1
GCA_030060845.1
GCA_030060925.1
GCA_030061615.1
GCA_030061645.1
GCA_030061665.1
GCA_030061685.1
GCA_030061715.1
GCA_030061755.1
GCA_030061775.1
GCA_030061795.1
GCA_030061815.1
GCA_030061865.1
GCA_030061895.1
GCA_030061915.1
GCA_030061935.1
GCA_030061955.1
GCA_030061985.1
GCA_030062035.1
GCA_030062055.1
GCA_030062075.1
GCA_030062095.1
GCA_030062115.1
GCA_030062135.1
GCA_030062155.1
GCA_030062245.1
GCA_030062355.1
GCA_030062375.1
GCA_030062395.1
GCA_030062415.1
GCA_030062435.1
GCA_030062465.1
GCA_030062515.1
GCA_030062605.1
GCA_030062705.1
GCA_030062725.1
GCA_030062785.1
GCA_030062805.1
GCA_030062825.1
GCA_030062845.1
GCA_030062875.1
GCA_030062895.1
GCA_030062915.1
GCA_030062935.1
GCA_030063885.1
GCA_030063925.1

GCA_030063945.1
GCA_030063965.1
GCA_030063985.1
GCA_030064005.1
GCA_030064025.1
GCA_030064045.1
GCA_030064065.1
GCA_030064105.1
GCA_030064125.1
GCA_030064145.1
GCA_030064165.1
GCA_030064245.1
GCA_030064365.1
GCA_030064445.1
GCA_030064585.1
GCA_030064615.2
GCA_030064635.1
GCA_030064655.1
GCA_030064675.1
GCA_030064715.1
GCA_030064735.1
GCA_030067755.1
GCA_030067795.1
GCA_030067815.1
GCA_030067835.1
GCA_030067855.1
GCA_030067875.1
GCA_030077565.1
GCA_030077605.1
GCA_030077625.1
GCA_030078095.1
GCA_030078115.1
GCA_030078145.1
GCA_030078235.1
GCA_030078255.1
GCA_030078275.1
GCA_030078295.1
GCA_030078315.1
GCA_030078335.1
GCA_030078355.1
GCA_030078375.1
GCA_030078395.1
GCA_030078415.1
GCA_030078435.1
GCA_030122645.1
GCA_030122665.1
GCA_030122685.1
GCA_030122705.1
GCA_030122725.1
GCA_030122745.1
GCA_030122765.1
GCA_030122785.1
GCA_030122805.1
GCA_030122825.1

GCA_030122845.1
GCA_030122865.1
GCA_030122885.1
GCA_030122905.1
GCA_030122925.1
GCA_030122945.1
GCA_030122965.1
GCA_030122985.1
GCA_030123005.1
GCA_030123025.1
GCA_030123045.1
GCA_030123065.1
GCA_030123085.1
GCA_030123105.1
GCA_030123125.1
GCA_030123145.1
GCA_030123165.1
GCA_030123185.1
GCA_030123205.1
GCA_030123225.1
GCA_030123245.1
GCA_030123265.1
GCA_030123285.1
GCA_030123305.1
GCA_030123325.1
GCA_030123345.1
GCA_030123365.1
GCA_030123385.1
GCA_030123405.1
GCA_030123425.1
GCA_030123445.1
GCA_030123485.1
GCA_030123505.1
GCA_030123545.1
GCA_030123585.1
GCA_030123605.1
GCA_030123625.1
GCA_030126965.1
GCA_030126985.1
GCA_030127005.1
GCA_030127025.1
GCA_030127045.1
GCA_030127065.1
GCA_030127085.1
GCA_030127185.1
GCA_030127205.1
GCA_030127225.1
GCA_030127385.1
GCA_030127405.1
GCA_030127445.1
GCA_030128825.1
GCA_030128945.2
GCA_030142045.1
GCA_030142075.1

GCA_030142155.1
GCA_030142205.1
GCA_030142245.1
GCA_030142335.1
GCA_030142415.1
GCA_030142475.1
GCA_030142985.1
GCA_030144325.1
GCA_030144345.1
GCA_030144365.1
GCA_030144385.1
GCA_030144685.1
GCA_030144705.1
GCA_030144795.1
GCA_030144815.1
GCA_030161935.2
GCA_030162015.2
GCA_030162035.2
GCA_030162075.2
GCA_030162155.2
GCA_030162175.2
GCA_030162195.2
GCA_030166335.1
GCA_030166355.1
GCA_030166375.1
GCA_030166395.1
GCA_030166415.1
GCA_030166435.1
GCA_030166455.1
GCA_030166475.1
GCA_030166495.1
GCA_030166515.1
GCA_030166535.1
GCA_030166555.1
GCA_030166575.1
GCA_030166595.1
GCA_030166615.1
GCA_030166635.1
GCA_030166655.1
GCA_030166675.1
GCA_030166695.1
GCA_030166715.1
GCA_030166735.1
GCA_030166975.1
GCA_030166995.1
GCA_030167015.1
GCA_030167035.1
GCA_030167055.1
GCA_030167075.1
GCA_030167105.1
GCA_030167125.1
GCA_030167145.1
GCA_030167965.1
GCA_030167985.1

GCA_030168005.1
GCA_030168025.1
GCA_030168045.1
GCA_030168065.1
GCA_030168085.1
GCA_030168105.1
GCA_030168125.1
GCA_030168145.1
GCA_030168165.1
GCA_030168185.1
GCA_030168205.1
GCA_030168225.1
GCA_030168245.1
GCA_030168265.1
GCA_030168285.1
GCA_030168305.1
GCA_030168325.1
GCA_030168345.1
GCA_030168365.1
GCA_030168385.1
GCA_030168405.1
GCA_030168425.1
GCA_030168445.1
GCA_030168495.1
GCA_030168515.1
GCA_030168535.1
GCA_030168615.1
GCA_030168635.1
GCA_030168655.1
GCA_030168675.1
GCA_030168695.1
GCA_030168715.1
GCA_030168735.1
GCA_030168755.1
GCA_030168775.1
GCA_030168875.1
GCA_030168895.1
GCA_030168915.1
GCA_030168935.1
GCA_030168955.1
GCA_030168975.1
GCA_030169005.1
GCA_030169085.1
GCA_030169105.1
GCA_030169125.1
GCA_030169145.1
GCA_030169195.1
GCA_030169265.1
GCA_030169335.1
GCA_030169475.1
GCA_030169555.1
GCA_030169635.1
GCA_030169715.1
GCA_030169785.1

GCA_030169945.1
GCA_030177695.1
GCA_030177735.1
GCA_030177755.1
GCA_030177815.1
GCA_030177855.1
GCA_030177935.1
GCA_030178455.1
GCA_030178475.1
GCA_030179035.1
GCA_030179155.1
GCA_030179475.1
GCA_030179595.1
GCA_030179635.1
GCA_030179805.1
GCA_030179835.1
GCA_030219305.1
GCA_030219325.1
GCA_030219365.1
GCA_030219385.1
GCA_030219405.1
GCA_030219425.1
GCA_030219445.1
GCA_030219925.1
GCA_030219945.1
GCA_030219965.1
GCA_030220105.1
GCA_030220125.1
GCA_030220145.1
GCA_030220165.1
GCA_030246825.1
GCA_030246845.1
GCA_030246865.1
GCA_030246885.1
GCA_030246905.1
GCA_030246925.1
GCA_030246945.1
GCA_030246965.1
GCA_030246985.1
GCA_030252165.1
GCA_030252185.1
GCA_030252205.1
GCA_030252225.1
GCA_030252245.1
GCA_030252265.1
GCA_030252285.1
GCA_030252305.1
GCA_030252325.1
GCA_030252345.1
GCA_030252365.1
GCA_030252385.1
GCA_030252405.1
GCA_030252425.1
GCA_030252445.1

GCA_030252485.1
GCA_030252675.1
GCA_030252695.1
GCA_030252715.1
GCA_030252735.1
GCA_030252755.1
GCA_030252775.1
GCA_030252795.1
GCA_030253455.1
GCA_030253475.1
GCA_030253495.1
GCA_030253515.1
GCA_030253535.1
GCA_030253605.1
GCA_030253625.1
GCA_030254625.1
GCA_030254645.1
GCA_030254665.1
GCA_030254685.1
GCA_030254705.1
GCA_030254725.1
GCA_030254745.1
GCA_030254765.1
GCA_030254785.1
GCA_030254805.1
GCA_030255395.1
GCA_030263875.1
GCA_030263895.1
GCA_030263915.1
GCA_030263935.1
GCA_030263955.1
GCA_030263975.1
GCA_030264015.1
GCA_030264035.1
GCA_030264075.1
GCA_030264095.1
GCA_030264235.1
GCA_030264255.1
GCA_030264275.1
GCA_030264295.1
GCA_030264755.1
GCA_030271835.1
GCA_030271855.1
GCA_030271875.1
GCA_030271895.1
GCA_030271915.1
GCA_030271935.1
GCA_030271955.1
GCA_030271975.1
GCA_030271995.1
GCA_030272015.1
GCA_030272075.1
GCA_030272215.1
GCA_030272265.1

GCA_030272285.1
GCA_030273345.1
GCA_030273445.1
GCA_030285485.1
GCA_030285505.1
GCA_030285525.1
GCA_030285545.1
GCA_030285565.1
GCA_030285585.1
GCA_030285605.1
GCA_030285625.1
GCA_030285645.1
GCA_030285665.1
GCA_030285685.1
GCA_030285705.1
GCA_030285725.1
GCA_030285745.1
GCA_030285765.1
GCA_030285785.1
GCA_030285805.1
GCA_030285825.1
GCA_030285845.1
GCA_030285865.1
GCA_030285885.1
GCA_030285925.1
GCA_030285945.1
GCA_030286005.1
GCA_030286045.1
GCA_030290995.1
GCA_030291015.1
GCA_030291035.1
GCA_030291055.1
GCA_030291075.1
GCA_030291095.1
GCA_030291115.1
GCA_030291135.1
GCA_030291155.1
GCA_030291175.1
GCA_030291215.1
GCA_030291235.1
GCA_030291255.1
GCA_030291295.1
GCA_030291635.1
GCA_030291655.1
GCA_030291675.1
GCA_030291695.1
GCA_030291715.1
GCA_030291735.1
GCA_030291755.1
GCA_030291795.1
GCA_030291815.1
GCA_030291835.1
GCA_030291855.1
GCA_030291875.1

GCA_030291895.1
GCA_030291915.1
GCA_030291935.1
GCA_030291955.1
GCA_030291995.1
GCA_030292015.1
GCA_030292035.1
GCA_030292055.1
GCA_030292075.1
GCA_030292115.1
GCA_030292135.1
GCA_030292155.1
GCA_030292205.1
GCA_030292225.1
GCA_030294405.1
GCA_030294885.1
GCA_030294905.1
GCA_030294925.1
GCA_030294945.1
GCA_030294965.1
GCA_030294985.1
GCA_030295035.1
GCA_030295055.1
GCA_030295075.1
GCA_030295095.1
GCA_030295115.1
GCA_030295135.1
GCA_030295155.1
GCA_030295175.1
GCA_030295195.1
GCA_030295215.1
GCA_030295265.1
GCA_030295285.1
GCA_030295305.1
GCA_030295325.1
GCA_030295385.1
GCA_030295405.1
GCA_030295425.1
GCA_030295445.1
GCA_030295465.1
GCA_030295485.1
GCA_030295505.1
GCA_030295525.1
GCA_030295545.1
GCA_030295565.1
GCA_030295585.1
GCA_030295605.1
GCA_030295625.1
GCA_030295645.1
GCA_030295665.1
GCA_030295685.1
GCA_030295705.1
GCA_030295725.1
GCA_030295745.1

GCA_030295765.1
GCA_030295785.1
GCA_030295805.1
GCA_030295825.1
GCA_030295845.1
GCA_030295865.1
GCA_030295915.1
GCA_030295935.1
GCA_030295955.1
GCA_030295975.1
GCA_030295995.1
GCA_030296015.1
GCA_030296035.1
GCA_030296055.1
GCA_030296075.1
GCA_030296095.1
GCA_030296115.1
GCA_030296135.1
GCA_030296155.1
GCA_030296175.1
GCA_030296195.1
GCA_030296215.1
GCA_030296235.1
GCA_030296255.1
GCA_030296275.1
GCA_030296295.1
GCA_030296335.1
GCA_030296355.1
GCA_030296375.1
GCA_030296395.1
GCA_030296415.1
GCA_030296435.1
GCA_030296455.1
GCA_030296475.1
GCA_030296495.1
GCA_030296515.1
GCA_030296535.1
GCA_030296555.1
GCA_030296575.1
GCA_030296595.1
GCA_030296615.1
GCA_030296635.1
GCA_030296655.1
GCA_030296675.1
GCA_030296695.1
GCA_030296755.1
GCA_030296775.1
GCA_030296795.1
GCA_030296815.1
GCA_030296835.1
GCA_030296855.1
GCA_030296875.1
GCA_030296895.1
GCA_030296915.1

GCA_030296935.1
GCA_030296955.1
GCA_030296975.1
GCA_030296995.1
GCA_030297015.1
GCA_030297035.1
GCA_030297055.1
GCA_030297075.1
GCA_030297095.1
GCA_030297115.1
GCA_030297135.1
GCA_030297155.1
GCA_030297175.1
GCA_030297195.1
GCA_030297215.1
GCA_030297235.1
GCA_030297255.1
GCA_030297275.1
GCA_030297295.1
GCA_030297315.1
GCA_030297335.1
GCA_030297355.1
GCA_030297375.1
GCA_030297395.1
GCA_030297415.1
GCA_030297435.1
GCA_030297455.1
GCA_030297475.1
GCA_030297495.1
GCA_030297515.1
GCA_030297535.1
GCA_030297555.1
GCA_030297575.1
GCA_030297595.1
GCA_030297615.1
GCA_030297635.1
GCA_030297655.1
GCA_030297675.1
GCA_030297695.1
GCA_030297715.1
GCA_030297735.1
GCA_030315995.1
GCA_030316015.1
GCA_030316055.1
GCA_030316075.1
GCA_030316115.1
GCA_030316135.1
GCA_030316155.1
GCA_030316175.1
GCA_030316215.1
GCA_030316235.1
GCA_030316255.1
GCA_030316605.1
GCA_030322905.1

GCA_030322925.1
GCA_030322945.1
GCA_030322965.1
GCA_030322985.1
GCA_030323005.1
GCA_030323025.1
GCA_030323045.1
GCA_030323065.1
GCA_030323105.1
GCA_030323185.1
GCA_030323245.1
GCA_030323265.1
GCA_030323285.1
GCA_030323305.1
GCA_030323345.1
GCA_030323365.1
GCA_030323385.1
GCA_030323405.1
GCA_030323425.1
GCA_030323445.1
GCA_030323465.1
GCA_030323485.1
GCA_030323505.1
GCA_030323525.1
GCA_030323545.1
GCA_030323565.1
GCA_030323585.1
GCA_030323605.1
GCA_030323625.1
GCA_030323645.1
GCA_030323665.1
GCA_030323685.1
GCA_030323705.1
GCA_030323725.1
GCA_030323745.1
GCA_030323765.1
GCA_030323785.1
GCA_030323805.1
GCA_030323825.1
GCA_030323865.1
GCA_030324145.1
GCA_030324175.1
GCA_030324195.1
GCA_030344915.1
GCA_030344935.1
GCA_030344975.1
GCA_030344995.1
GCA_030345015.1
GCA_030345035.1
GCA_030345055.1
GCA_030345195.1
GCA_030345215.1
GCA_030345235.1
GCA_030345275.1

GCA_030345295.1
GCA_030345315.1
GCA_030345415.1
GCA_030345435.1
GCA_030345455.1
GCA_030345475.1
GCA_030345495.1
GCA_030345515.1
GCA_030345535.1
GCA_030345555.1
GCA_030345575.1
GCA_030345595.1
GCA_030345615.1
GCA_030345635.1
GCA_030345655.1
GCA_030346995.1
GCA_030347015.1
GCA_030347035.1
GCA_030347055.1
GCA_030347075.1
GCA_030347095.1
GCA_030347115.1
GCA_030347135.1
GCA_030347155.1
GCA_030347175.1
GCA_030347195.1
GCA_030347215.1
GCA_030347235.1
GCA_030347245.1
GCA_030347395.1
GCA_030369595.1
GCA_030369615.1
GCA_030369635.1
GCA_030369655.1
GCA_030369675.1
GCA_030369695.1
GCA_030369715.1
GCA_030369815.1
GCA_030369835.1
GCA_030369855.1
GCA_030369875.1
GCA_030369895.1
GCA_030369915.1
GCA_030369935.1
GCA_030369955.1
GCA_030369975.1
GCA_030370055.1
GCA_030370075.1
GCA_030370095.1
GCA_030370115.1
GCA_030370135.1
GCA_030370155.1
GCA_030370175.1
GCA_030370195.1

GCA_030370215.1
GCA_030370235.1
GCA_030370255.1
GCA_030370275.1
GCA_030370295.1
GCA_030370315.1
GCA_030370335.1
GCA_030370355.1
GCA_030370375.1
GCA_030370395.1
GCA_030370415.1
GCA_030370465.1
GCA_030370735.1
GCA_030374065.1
GCA_030374105.1
GCA_030374125.1
GCA_030378485.1
GCA_030384605.1
GCA_030384685.1
GCA_030384705.1
GCA_030384725.1
GCA_030384745.1
GCA_030384765.1
GCA_030384805.1
GCA_030384825.1
GCA_030384845.1
GCA_030384865.1
GCA_030384885.1
GCA_030388245.1
GCA_030388265.1
GCA_030388285.1
GCA_030388305.1
GCA_030388325.1
GCA_030388345.1
GCA_030388365.1
GCA_030388405.1
GCA_030388425.1
GCA_030388445.1
GCA_030388485.1
GCA_030388645.1
GCA_030388665.1
GCA_030388685.1
GCA_030388705.1
GCA_030388725.1
GCA_030388745.1
GCA_030388765.1
GCA_030388785.1
GCA_030388805.1
GCA_030389005.1
GCA_030389145.1
GCA_030389165.1
GCA_030389215.1
GCA_030389235.1
GCA_030389275.1

GCA_030389295.1
GCA_030389315.1
GCA_030389495.1
GCA_030389595.1
GCA_030389655.1
GCA_030389695.1
GCA_030389715.1
GCA_030389735.1
GCA_030389755.1
GCA_030389775.1
GCA_030389835.1
GCA_030389855.1
GCA_030389925.1
GCA_030389945.1
GCA_030389965.1
GCA_030406025.1
GCA_030406045.1
GCA_030406065.1
GCA_030406105.1
GCA_030406125.1
GCA_030406145.1
GCA_030406165.1
GCA_030406205.1
GCA_030406585.1
GCA_030406645.1
GCA_030406665.1
GCA_030406685.1
GCA_030406705.1
GCA_030406725.1
GCA_030406755.1
GCA_030406775.1
GCA_030406845.1
GCA_030406865.1
GCA_030406905.1
GCA_030407165.1
GCA_030408355.1
GCA_030408375.1
GCA_030408395.1
GCA_030408415.1
GCA_030408435.1
GCA_030408455.1
GCA_030408555.1
GCA_030408575.1
GCA_030408595.1
GCA_030408615.1
GCA_030408635.1
GCA_030408655.1
GCA_030408675.1
GCA_030408695.1
GCA_030408715.1
GCA_030408735.1
GCA_030408755.1
GCA_030408775.1
GCA_030408795.1

GCA_030408815.1
GCA_030408835.1
GCA_030408855.1
GCA_030408875.1
GCA_030408895.1
GCA_030408915.1
GCA_030408935.1
GCA_030408955.1
GCA_030408975.1
GCA_030408995.1
GCA_030409015.1
GCA_030409035.1
GCA_030412635.1
GCA_030413175.1
GCA_030413195.1
GCA_030413215.1
GCA_900002505.1
GCA_900002515.1
GCA_900004845.1
GCA_900004855.1
GCA_900005615.1
GCA_900009115.1
GCA_900009125.1
GCA_900010105.1
GCA_900010165.1
GCA_900010175.1
GCA_900010805.1
GCA_900011245.1
GCA_900012655.1
GCA_900014395.1
GCA_900015005.1
GCA_900016155.1
GCA_900016785.1
GCA_900017775.1
GCA_900019315.1
GCA_900029885.1
GCA_900039485.1
GCA_900044005.1
GCA_900044015.1
GCA_900048035.1
GCA_900048045.1
GCA_900066015.1
GCA_900066025.1
GCA_900069025.1
GCA_900070175.1
GCA_900070325.1
GCA_900070345.1
GCA_900070355.1
GCA_900070365.1
GCA_900070375.1
GCA_900074915.1
GCA_900078265.1
GCA_900078365.1
GCA_900078695.1

GCA_900079785.1
GCA_900079795.1
GCA_900086555.1
GCA_900086565.1
GCA_900086615.2
GCA_900086895.1
GCA_900086925.1
GCA_900087025.1
GCA_900087055.1
GCA_900087165.1
GCA_900087185.1
GCA_900087375.1
GCA_900087615.2
GCA_900087625.2
GCA_900087635.2
GCA_900087645.2
GCA_900087655.1
GCA_900087685.2
GCA_900087715.2
GCA_900087725.2
GCA_900087735.2
GCA_900087785.2
GCA_900087795.2
GCA_900087815.2
GCA_900087865.2
GCA_900087875.2
GCA_900087905.2
GCA_900088105.2
GCA_900088425.1
GCA_900088535.1
GCA_900088685.1
GCA_900088695.1
GCA_900088705.1
GCA_900089455.2
GCA_900090215.1
GCA_900092345.1
GCA_900092355.1
GCA_900092405.1
GCA_900092465.1
GCA_900092475.1
GCA_900092595.1
GCA_900092615.1
GCA_900092755.1
GCA_900093475.1
GCA_900093775.1
GCA_900094135.1
GCA_900094185.1
GCA_900094325.1
GCA_900094615.1
GCA_900095075.1
GCA_900095135.1
GCA_900095155.1
GCA_900095175.1
GCA_900095195.1

GCA_900095205.1
GCA_900095215.1
GCA_900095285.1
GCA_900095495.1
GCA_900095795.1
GCA_900095805.1
GCA_900095885.1
GCA_900096745.1
GCA_900096755.1
GCA_900096765.1
GCA_900096795.1
GCA_900096845.1
GCA_900097105.1
GCA_900097165.1
GCA_900097175.1
GCA_900097205.1
GCA_900097215.1
GCA_900097225.1
GCA_900097235.1
GCA_900097245.1
GCA_900097255.1
GCA_900097265.1
GCA_900116045.1
GCA_900116695.1
GCA_900116935.1
GCA_900119315.1
GCA_900119705.1
GCA_900119755.1
GCA_900119765.1
GCA_900119775.1
GCA_900119785.1
GCA_900119795.1
GCA_900119915.1
GCA_900119955.1
GCA_900119995.1
GCA_900120055.1
GCA_900120135.1
GCA_900120165.1
GCA_900120185.1
GCA_900120205.1
GCA_900120225.1
GCA_900120245.1
GCA_900120255.1
GCA_900120275.1
GCA_900120315.1
GCA_900120335.1
GCA_900120345.1
GCA_900120375.1
GCA_900128415.1
GCA_900128595.1
GCA_900128725.1
GCA_900128735.1
GCA_900129335.1
GCA_900130075.1

GCA_900130145.1
GCA_900143135.1
GCA_900143575.1
GCA_900149285.1
GCA_900149385.2
GCA_900149465.2
GCA_900149505.2
GCA_900149625.2
GCA_900149805.1
GCA_900149875.1
GCA_900149915.1
GCA_900155335.1
GCA_900155645.1
GCA_900155855.1
GCA_900157305.1
GCA_900161835.1
GCA_900166885.1
GCA_900166955.1
GCA_900168255.1
GCA_900168365.1
GCA_900169085.1
GCA_900169485.1
GCA_900169565.1
GCA_900174625.1
GCA_900174635.1
GCA_900175995.1
GCA_900176185.1
GCA_900176205.1
GCA_900183405.1
GCA_900183975.1
GCA_900183985.1
GCA_900183995.1
GCA_900184295.1
GCA_900184385.1
GCA_900184705.1
GCA_900184875.1
GCA_900185255.1
GCA_900185485.1
GCA_900185995.1
GCA_900186835.1
GCA_900186845.1
GCA_900186855.1
GCA_900186865.1
GCA_900186875.1
GCA_900186885.1
GCA_900186895.1
GCA_900186905.1
GCA_900186915.1
GCA_900186925.1
GCA_900186935.1
GCA_900186945.1
GCA_900186955.1
GCA_900186965.1
GCA_900186975.1

GCA_900186985.1
GCA_900186995.1
GCA_900187005.1
GCA_900187015.1
GCA_900187025.1
GCA_900187035.1
GCA_900187045.1
GCA_900187055.1
GCA_900187065.1
GCA_900187075.1
GCA_900187085.1
GCA_900187095.1
GCA_900187105.1
GCA_900187115.1
GCA_900187135.1
GCA_900187145.1
GCA_900187155.1
GCA_900187165.1
GCA_900187175.1
GCA_900187185.1
GCA_900187195.1
GCA_900187205.1
GCA_900187215.1
GCA_900187225.1
GCA_900187235.1
GCA_900187245.1
GCA_900187255.1
GCA_900187265.1
GCA_900187275.1
GCA_900187285.1
GCA_900187295.1
GCA_900187305.1
GCA_900187315.1
GCA_900187345.1
GCA_900187355.1
GCA_900196535.2
GCA_900196735.1
GCA_900198195.1
GCA_900199375.1
GCA_900205245.2
GCA_900205255.1
GCA_900205265.1
GCA_900205275.1
GCA_900205295.1
GCA_900205735.1
GCA_900205745.1
GCA_900209925.1
GCA_900217235.1
GCA_900231165.1
GCA_900231475.1
GCA_900231485.1
GCA_900232105.1
GCA_900233005.1
GCA_900234345.1

GCA_900234355.1
GCA_900234375.1
GCA_900234395.1
GCA_900234405.1
GCA_900234795.1
GCA_900235815.1
GCA_900235825.1
GCA_900235835.1
GCA_900235865.1
GCA_900235885.1
GCA_900235895.1
GCA_900235905.1
GCA_900236335.1
GCA_900236345.1
GCA_900236355.1
GCA_900236365.1
GCA_900236375.1
GCA_900236385.1
GCA_900236395.1
GCA_900236405.1
GCA_900236415.1
GCA_900236425.1
GCA_900236435.1
GCA_900236445.1
GCA_900236455.1
GCA_900236465.1
GCA_900236475.1
GCA_900236485.1
GCA_900236495.1
GCA_900236505.1
GCA_900236515.1
GCA_900236525.1
GCA_900236535.1
GCA_900236545.1
GCA_900236555.1
GCA_900236565.1
GCA_900236575.1
GCA_900236585.1
GCA_900239945.1
GCA_900239975.1
GCA_900240075.1
GCA_900243355.1
GCA_900243745.1
GCA_900275645.1
GCA_900289045.1
GCA_900289125.1
GCA_900292015.1
GCA_900312965.1
GCA_900312975.1
GCA_900322585.1
GCA_900322715.1
GCA_900322725.1
GCA_900323905.1
GCA_900323925.1

GCA_900323945.1
GCA_900323955.1
GCA_900323965.1
GCA_900324035.1
GCA_900324045.1
GCA_900324065.1
GCA_900324205.1
GCA_900324215.1
GCA_900324225.1
GCA_900324235.1
GCA_900324245.1
GCA_900324255.1
GCA_900324265.1
GCA_900324275.1
GCA_900324285.1
GCA_900324295.1
GCA_900324305.1
GCA_900324315.1
GCA_900324325.1
GCA_900324335.1
GCA_900324345.1
GCA_900324355.1
GCA_900324365.1
GCA_900324385.1
GCA_900324405.1
GCA_900324415.1
GCA_900324425.1
GCA_900324435.1
GCA_900324445.1
GCA_900324455.1
GCA_900324475.1
GCA_900327235.1
GCA_900327245.1
GCA_900327255.1
GCA_900327265.1
GCA_900327275.1
GCA_900327835.1
GCA_900327845.1
GCA_900327855.1
GCA_900327865.1
GCA_900327875.1
GCA_900332045.1
GCA_900343015.1
GCA_900343095.1
GCA_900343145.1
GCA_900416705.2
GCA_900416725.2
GCA_900458895.2
GCA_900465055.1
GCA_900465145.1
GCA_900465165.1
GCA_900465175.1
GCA_900465355.1
GCA_900465715.1

GCA_900465735.1
GCA_900465745.1
GCA_900465785.1
GCA_900465795.1
GCA_900465925.1
GCA_900474405.1
GCA_900474415.1
GCA_900474445.1
GCA_900474455.1
GCA_900474495.1
GCA_900474525.1
GCA_900474535.1
GCA_900474555.1
GCA_900474565.1
GCA_900474575.1
GCA_900474585.1
GCA_900474605.1
GCA_900474615.1
GCA_900474625.1
GCA_900474645.1
GCA_900474665.1
GCA_900474675.1
GCA_900474685.1
GCA_900474695.1
GCA_900474705.1
GCA_900474715.1
GCA_900474725.1
GCA_900474735.1
GCA_900474745.1
GCA_900474755.1
GCA_900474765.1
GCA_900474775.1
GCA_900474785.1
GCA_900474795.1
GCA_900474805.1
GCA_900474815.1
GCA_900474825.1
GCA_900474835.1
GCA_900474845.1
GCA_900474855.1
GCA_900474865.1
GCA_900474875.1
GCA_900474885.1
GCA_900474895.1
GCA_900474905.1
GCA_900474915.1
GCA_900474925.1
GCA_900474935.1
GCA_900474945.1
GCA_900474955.1
GCA_900474965.1
GCA_900474975.1
GCA_900474985.1
GCA_900474995.1

GCA_900475005.1
GCA_900475015.1
GCA_900475025.1
GCA_900475035.1
GCA_900475045.1
GCA_900475055.1
GCA_900475065.1
GCA_900475075.1
GCA_900475085.1
GCA_900475095.1
GCA_900475105.1
GCA_900475115.1
GCA_900475125.1
GCA_900475135.1
GCA_900475145.1
GCA_900475155.1
GCA_900475165.1
GCA_900475175.1
GCA_900475185.1
GCA_900475195.1
GCA_900475215.1
GCA_900475225.1
GCA_900475235.1
GCA_900475245.1
GCA_900475255.1
GCA_900475265.1
GCA_900475275.1
GCA_900475285.1
GCA_900475295.1
GCA_900475305.1
GCA_900475315.1
GCA_900475335.1
GCA_900475345.1
GCA_900475355.1
GCA_900475365.1
GCA_900475375.1
GCA_900475385.1
GCA_900475395.1
GCA_900475405.1
GCA_900475415.1
GCA_900475425.1
GCA_900475445.1
GCA_900475455.1
GCA_900475465.1
GCA_900475475.1
GCA_900475485.1
GCA_900475495.1
GCA_900475505.1
GCA_900475515.1
GCA_900475525.1
GCA_900475535.1
GCA_900475545.1
GCA_900475555.1
GCA_900475565.1

GCA_900475575.1
GCA_900475585.1
GCA_900475595.1
GCA_900475605.1
GCA_900475615.1
GCA_900475625.1
GCA_900475635.1
GCA_900475645.1
GCA_900475655.1
GCA_900475665.1
GCA_900475675.1
GCA_900475685.1
GCA_900475695.1
GCA_900475705.1
GCA_900475715.1
GCA_900475725.1
GCA_900475735.1
GCA_900475745.1
GCA_900475755.1
GCA_900475765.1
GCA_900475775.1
GCA_900475785.1
GCA_900475795.1
GCA_900475805.1
GCA_900475815.1
GCA_900475825.1
GCA_900475835.1
GCA_900475845.1
GCA_900475855.1
GCA_900475865.1
GCA_900475875.1
GCA_900475885.1
GCA_900475895.1
GCA_900475905.1
GCA_900475915.1
GCA_900475925.1
GCA_900475935.1
GCA_900475945.1
GCA_900475955.1
GCA_900475965.1
GCA_900475975.1
GCA_900475985.1
GCA_900475995.1
GCA_900476005.1
GCA_900476015.1
GCA_900476025.1
GCA_900476035.1
GCA_900476045.1
GCA_900476055.1
GCA_900476075.1
GCA_900476085.1
GCA_900476105.1
GCA_900476125.1
GCA_900476175.1

GCA_900476215.1
GCA_900476255.1
GCA_900476435.1
GCA_900476445.1
GCA_900476455.1
GCA_900476465.1
GCA_900476475.1
GCA_900476505.1
GCA_900477885.1
GCA_900477895.1
GCA_900477905.1
GCA_900477925.1
GCA_900477945.1
GCA_900477975.1
GCA_900477985.1
GCA_900477995.1
GCA_900478015.1
GCA_900478035.1
GCA_900478045.1
GCA_900478065.1
GCA_900478095.1
GCA_900478105.1
GCA_900478115.1
GCA_900478125.1
GCA_900478135.1
GCA_900478145.1
GCA_900478155.1
GCA_900478165.1
GCA_900478175.1
GCA_900478185.1
GCA_900478195.1
GCA_900478205.1
GCA_900478215.1
GCA_900478225.1
GCA_900478235.1
GCA_900478245.1
GCA_900478255.1
GCA_900478265.1
GCA_900478275.1
GCA_900478285.1
GCA_900478295.1
GCA_900478315.1
GCA_900478325.1
GCA_900478345.1
GCA_900478375.1
GCA_900478385.1
GCA_900478395.1
GCA_900478405.1
GCA_900478415.1
GCA_900478435.1
GCA_900478715.1
GCA_900478735.1
GCA_900478745.1
GCA_900478755.1

GCA_900489515.1
GCA_900489525.1
GCA_900489545.1
GCA_900489555.1
GCA_900489565.1
GCA_900489575.1
GCA_900489595.1
GCA_900489625.1
GCA_900489655.1
GCA_900489665.1
GCA_900489675.1
GCA_900489685.1
GCA_900489705.1
GCA_900489715.1
GCA_900489725.1
GCA_900489765.1
GCA_900489785.1
GCA_900489795.1
GCA_900489805.1
GCA_900489825.1
GCA_900492145.1
GCA_900492165.1
GCA_900492555.1
GCA_900497025.1
GCA_900497045.1
GCA_900497055.1
GCA_900497095.1
GCA_900497145.1
GCA_900497415.1
GCA_900497455.1
GCA_900497475.1
GCA_900497485.1
GCA_900497645.1
GCA_900519455.1
GCA_900519475.1
GCA_900520285.1
GCA_900520305.1
GCA_900520315.1
GCA_900520325.1
GCA_900520335.1
GCA_900520345.1
GCA_900520355.1
GCA_900520365.1
GCA_900520375.1
GCA_900520385.1
GCA_900520395.1
GCA_900536895.1
GCA_900537175.1
GCA_900537185.1
GCA_900537995.1
GCA_900538065.1
GCA_900538275.1
GCA_900560965.1
GCA_900564155.1

GCA_900574125.1
GCA_900603025.1
GCA_900604345.1
GCA_900604385.1
GCA_900604845.1
GCA_900607245.1
GCA_900607255.1
GCA_900607265.1
GCA_900607275.1
GCA_900607285.1
GCA_900607295.1
GCA_900607305.1
GCA_900608505.1
GCA_900618245.1
GCA_900618255.1
GCA_900618265.1
GCA_900618275.1
GCA_900618285.1
GCA_900618295.1
GCA_900618305.1
GCA_900618315.1
GCA_900618325.1
GCA_900618335.1
GCA_900618545.1
GCA_900618555.1
GCA_900618575.1
GCA_900618585.1
GCA_900619585.1
GCA_900619595.1
GCA_900619605.1
GCA_900619615.1
GCA_900619625.1
GCA_900620215.1
GCA_900620225.1
GCA_900620235.1
GCA_900620245.1
GCA_900620255.1
GCA_900622505.2
GCA_900622585.1
GCA_900622595.1
GCA_900622605.1
GCA_900622615.1
GCA_900622625.1
GCA_900622635.1
GCA_900622645.1
GCA_900622655.1
GCA_900622665.1
GCA_900622685.1
GCA_900622695.1
GCA_900631595.1
GCA_900631605.1
GCA_900631615.1
GCA_900634805.1
GCA_900635025.1

GCA_900635075.1
GCA_900635085.1
GCA_900635095.1
GCA_900635105.1
GCA_900635115.1
GCA_900635125.1
GCA_900635135.1
GCA_900635145.1
GCA_900635155.1
GCA_900635165.1
GCA_900635175.1
GCA_900635185.1
GCA_900635195.1
GCA_900635205.1
GCA_900635215.1
GCA_900635225.1
GCA_900635235.1
GCA_900635245.1
GCA_900635255.1
GCA_900635265.1
GCA_900635275.1
GCA_900635285.1
GCA_900635295.1
GCA_900635305.1
GCA_900635315.1
GCA_900635325.1
GCA_900635335.1
GCA_900635345.1
GCA_900635365.1
GCA_900635415.1
GCA_900635425.1
GCA_900635435.1
GCA_900635445.1
GCA_900635455.1
GCA_900635465.1
GCA_900635475.1
GCA_900635485.1
GCA_900635495.1
GCA_900635505.1
GCA_900635515.1
GCA_900635525.1
GCA_900635535.1
GCA_900635545.1
GCA_900635555.1
GCA_900635565.1
GCA_900635575.1
GCA_900635585.1
GCA_900635595.1
GCA_900635605.1
GCA_900635615.1
GCA_900635625.1
GCA_900635635.1
GCA_900635645.1
GCA_900635655.1

GCA_900635665.1
GCA_900635675.1
GCA_900635685.1
GCA_900635695.1
GCA_900635705.1
GCA_900635715.1
GCA_900635725.1
GCA_900635735.1
GCA_900635745.1
GCA_900635755.1
GCA_900635765.1
GCA_900635775.1
GCA_900635785.1
GCA_900635795.1
GCA_900635805.1
GCA_900635815.1
GCA_900635825.1
GCA_900635835.1
GCA_900635845.1
GCA_900635855.1
GCA_900635865.1
GCA_900635875.1
GCA_900635885.1
GCA_900635895.1
GCA_900635905.1
GCA_900635915.1
GCA_900635925.1
GCA_900635935.1
GCA_900635945.1
GCA_900635955.1
GCA_900635965.1
GCA_900635975.1
GCA_900635985.1
GCA_900635995.1
GCA_900636005.1
GCA_900636015.1
GCA_900636025.1
GCA_900636035.1
GCA_900636045.1
GCA_900636055.1
GCA_900636065.1
GCA_900636075.1
GCA_900636085.1
GCA_900636105.1
GCA_900636115.1
GCA_900636125.1
GCA_900636135.1
GCA_900636145.1
GCA_900636155.1
GCA_900636165.1
GCA_900636175.1
GCA_900636185.1
GCA_900636195.1
GCA_900636215.1

GCA_900636225.1
GCA_900636235.1
GCA_900636245.1
GCA_900636255.1
GCA_900636265.1
GCA_900636275.1
GCA_900636285.1
GCA_900636295.1
GCA_900636305.1
GCA_900636325.1
GCA_900636335.1
GCA_900636345.2
GCA_900636355.2
GCA_900636365.1
GCA_900636375.1
GCA_900636385.1
GCA_900636395.1
GCA_900636405.1
GCA_900636425.1
GCA_900636435.1
GCA_900636445.1
GCA_900636455.1
GCA_900636465.1
GCA_900636475.1
GCA_900636485.1
GCA_900636495.1
GCA_900636505.1
GCA_900636515.1
GCA_900636525.1
GCA_900636535.1
GCA_900636545.1
GCA_900636555.1
GCA_900636565.1
GCA_900636575.1
GCA_900636585.1
GCA_900636595.1
GCA_900636605.1
GCA_900636615.1
GCA_900636625.1
GCA_900636635.1
GCA_900636645.1
GCA_900636655.1
GCA_900636665.1
GCA_900636675.1
GCA_900636685.1
GCA_900636695.1
GCA_900636705.1
GCA_900636715.1
GCA_900636725.1
GCA_900636735.1
GCA_900636745.1
GCA_900636755.1
GCA_900636765.1
GCA_900636775.1

GCA_900636785.1
GCA_900636795.1
GCA_900636805.1
GCA_900636815.1
GCA_900636825.1
GCA_900636835.1
GCA_900636845.1
GCA_900636855.1
GCA_900636875.1
GCA_900636885.1
GCA_900636895.1
GCA_900636905.1
GCA_900636915.1
GCA_900636925.1
GCA_900636935.1
GCA_900636955.1
GCA_900636965.1
GCA_900636975.1
GCA_900636985.1
GCA_900636995.1
GCA_900637005.1
GCA_900637015.1
GCA_900637025.1
GCA_900637035.1
GCA_900637045.1
GCA_900637055.1
GCA_900637065.1
GCA_900637075.1
GCA_900637095.1
GCA_900637105.1
GCA_900637125.1
GCA_900637145.1
GCA_900637155.1
GCA_900637165.1
GCA_900637175.1
GCA_900637185.1
GCA_900637195.1
GCA_900637205.1
GCA_900637215.1
GCA_900637235.1
GCA_900637245.1
GCA_900637255.1
GCA_900637265.1
GCA_900637275.1
GCA_900637295.1
GCA_900637305.1
GCA_900637315.1
GCA_900637325.1
GCA_900637335.1
GCA_900637395.1
GCA_900637475.1
GCA_900637495.1
GCA_900637515.1
GCA_900637545.1

GCA_900637555.1
GCA_900637565.1
GCA_900637575.1
GCA_900637585.1
GCA_900637605.1
GCA_900637615.1
GCA_900637625.1
GCA_900637635.1
GCA_900637645.1
GCA_900637655.1
GCA_900637665.1
GCA_900637675.1
GCA_900637685.1
GCA_900637695.1
GCA_900637705.1
GCA_900637715.1
GCA_900637725.1
GCA_900637735.1
GCA_900637745.1
GCA_900637755.1
GCA_900637765.1
GCA_900637775.1
GCA_900637785.1
GCA_900637795.1
GCA_900637805.1
GCA_900637815.1
GCA_900637825.1
GCA_900637835.1
GCA_900637845.1
GCA_900637855.1
GCA_900637865.1
GCA_900637875.1
GCA_900637885.1
GCA_900637895.1
GCA_900637905.1
GCA_900637915.1
GCA_900637925.1
GCA_900637935.1
GCA_900637945.1
GCA_900637955.1
GCA_900637965.1
GCA_900637975.1
GCA_900637985.1
GCA_900637995.1
GCA_900638005.1
GCA_900638015.1
GCA_900638025.1
GCA_900638035.1
GCA_900638055.1
GCA_900638065.1
GCA_900638075.1
GCA_900638085.1
GCA_900638105.1
GCA_900638125.1

GCA_900638135.1
GCA_900638145.1
GCA_900638155.1
GCA_900638165.1
GCA_900638175.1
GCA_900638185.1
GCA_900638195.1
GCA_900638205.1
GCA_900638215.1
GCA_900638225.1
GCA_900638235.1
GCA_900638245.1
GCA_900638255.1
GCA_900638285.1
GCA_900638305.1
GCA_900638315.1
GCA_900638335.1
GCA_900638345.1
GCA_900638355.1
GCA_900638365.1
GCA_900638385.1
GCA_900638415.1
GCA_900638435.1
GCA_900638445.1
GCA_900638475.1
GCA_900638485.1
GCA_900638495.1
GCA_900638505.1
GCA_900638535.1
GCA_900638555.1
GCA_900638565.1
GCA_900638575.1
GCA_900638585.1
GCA_900638595.1
GCA_900638605.1
GCA_900638615.1
GCA_900638625.1
GCA_900638635.1
GCA_900638645.1
GCA_900638655.1
GCA_900638665.1
GCA_900638675.1
GCA_900638685.1
GCA_900638695.1
GCA_900638705.1
GCA_900638715.1
GCA_900638745.1
GCA_900638765.1
GCA_900638775.1
GCA_900638785.1
GCA_900638805.1
GCA_900638815.1
GCA_900638825.1
GCA_900639335.1

GCA_900639345.1
GCA_900639355.1
GCA_900639365.1
GCA_900639385.1
GCA_900639395.1
GCA_900639405.1
GCA_900639415.1
GCA_900639425.1
GCA_900639445.1
GCA_900639455.1
GCA_900639465.1
GCA_900639485.1
GCA_900639495.1
GCA_900639505.1
GCA_900639515.1
GCA_900639525.1
GCA_900639535.1
GCA_900639545.1
GCA_900639555.1
GCA_900639565.1
GCA_900639575.1
GCA_900639585.1
GCA_900639595.1
GCA_900639605.1
GCA_900639615.1
GCA_900639625.1
GCA_900639635.1
GCA_900639645.1
GCA_900639655.1
GCA_900639665.1
GCA_900639705.1
GCA_900639715.1
GCA_900639725.1
GCA_900639745.1
GCA_900640265.1
GCA_900654235.1
GCA_900659645.1
GCA_900659655.1
GCA_900659665.1
GCA_900659885.1
GCA_900660435.1
GCA_900660445.2
GCA_900660465.1
GCA_900660485.1
GCA_900660495.1
GCA_900660505.1
GCA_900660515.1
GCA_900660525.1
GCA_900660545.1
GCA_900660565.1
GCA_900660575.1
GCA_900660585.1
GCA_900660595.1
GCA_900660605.1

GCA_900660615.1
GCA_900660635.1
GCA_900660645.1
GCA_900660655.1
GCA_900660665.1
GCA_900660675.1
GCA_900660685.1
GCA_900660695.1
GCA_900660705.1
GCA_900660715.1
GCA_900660725.1
GCA_900660735.1
GCA_900660745.1
GCA_900660755.1
GCA_900682675.2
GCA_900683405.1
GCA_900683475.1
GCA_900683625.1
GCA_900683635.1
GCA_900683725.1
GCA_900683735.1
GCA_900683745.1
GCA_900683755.1
GCA_900683765.1
GCA_900683775.1
GCA_900692935.1
GCA_900693025.1
GCA_900693055.1
GCA_900693075.1
GCA_900698885.1
GCA_900698925.1
GCA_900698935.1
GCA_900698955.1
GCA_900698965.1
GCA_900698985.1
GCA_900698995.1
GCA_900699015.1
GCA_900699165.1
GCA_900699785.1
GCA_900747775.1
GCA_900747785.1
GCA_900749825.1
GCA_900795205.1
GCA_901421915.1
GCA_901421925.1
GCA_901421965.1
GCA_901421975.1
GCA_901422025.1
GCA_901422065.1
GCA_901446615.2
GCA_901446755.2
GCA_901457615.1
GCA_901457625.1
GCA_901457705.2

GCA_901457775.2
GCA_901457785.2
GCA_901457815.2
GCA_901457835.2
GCA_901472405.1
GCA_901472455.1
GCA_901472475.1
GCA_901472485.1
GCA_901472495.1
GCA_901472505.1
GCA_901472525.1
GCA_901472535.1
GCA_901472545.1
GCA_901472565.1
GCA_901472595.1
GCA_901482445.1
GCA_901482545.1
GCA_901482565.1
GCA_901482575.1
GCA_901482605.1
GCA_901482615.1
GCA_901482645.1
GCA_901482695.1
GCA_901521425.1
GCA_901538265.1
GCA_901538355.1
GCA_901542335.1
GCA_901542365.1
GCA_901542435.1
GCA_901542455.1
GCA_901542485.1
GCA_901543175.1
GCA_901543415.1
GCA_901543475.1
GCA_901543605.1
GCA_901543615.1
GCA_901543625.1
GCA_901543725.1
GCA_901543995.1
GCA_901544215.1
GCA_901544385.1
GCA_901553725.1
GCA_901553735.1
GCA_901553755.1
GCA_901733115.1
GCA_901827155.1
GCA_901827165.1
GCA_901827175.1
GCA_901827185.1
GCA_901827205.1
GCA_901846895.1
GCA_902109485.1
GCA_902141215.1
GCA_902141225.1

GCA_902141795.1
GCA_902141815.1
GCA_902141835.1
GCA_902167875.1
GCA_902167885.1
GCA_902172305.2
GCA_902379005.2
GCA_902381625.1
GCA_902381635.1
GCA_902381645.1
GCA_902381665.1
GCA_902381705.1
GCA_902381745.1
GCA_902381755.1
GCA_902381765.1
GCA_902381775.1
GCA_902381805.1
GCA_902381815.1
GCA_902385725.1
GCA_902385735.1
GCA_902385845.1
GCA_902385935.1
GCA_902386045.1
GCA_902386185.1
GCA_902386215.1
GCA_902386365.1
GCA_902386435.1
GCA_902386585.1
GCA_902386615.1
GCA_902386635.1
GCA_902386655.1
GCA_902386715.1
GCA_902386735.1
GCA_902386745.1
GCA_902386775.1
GCA_902386885.1
GCA_902386915.1
GCA_902386945.1
GCA_902387255.1
GCA_902387355.1
GCA_902387425.1
GCA_902387545.1
GCA_902387635.1
GCA_902387715.1
GCA_902387815.1
GCA_902387845.1
GCA_902387945.1
GCA_902387955.1
GCA_902387975.1
GCA_902388025.1
GCA_902388105.1
GCA_902388175.1
GCA_902388275.1
GCA_902459475.1

GCA_902459485.1
GCA_902459525.1
GCA_902459535.1
GCA_902459825.2
GCA_902498835.1
GCA_902502825.2
GCA_902509485.1
GCA_902509495.1
GCA_902509505.1
GCA_902509515.1
GCA_902509525.1
GCA_902509535.1
GCA_902702745.1
GCA_902702755.1
GCA_902702765.1
GCA_902702775.1
GCA_902702785.1
GCA_902702915.1
GCA_902702935.1
GCA_902703185.1
GCA_902703195.1
GCA_902703215.1
GCA_902703415.1
GCA_902705295.2
GCA_902713755.1
GCA_902723695.1
GCA_902723705.1
GCA_902729405.1
GCA_902806445.1
GCA_902806995.1
GCA_902807005.1
GCA_902807015.1
GCA_902809765.1
GCA_902809825.1
GCA_902809935.1
GCA_902810075.1
GCA_902810275.1
GCA_902810285.1
GCA_902810295.1
GCA_902810305.1
GCA_902810315.1
GCA_902810325.1
GCA_902810335.1
GCA_902810345.1
GCA_902810365.1
GCA_902810375.1
GCA_902810385.1
GCA_902810395.1
GCA_902810405.1
GCA_902810415.1
GCA_902810435.1
GCA_902810445.1
GCA_902813185.1
GCA_902825185.1

GCA_902825195.1
GCA_902825205.1
GCA_902825285.1
GCA_902825515.1
GCA_902825525.1
GCA_902827215.1
GCA_902829265.1
GCA_902829275.1
GCA_902860225.1
GCA_902983995.1
GCA_903064525.1
GCA_903815225.1
GCA_903815275.1
GCA_903884415.1
GCA_903885985.1
GCA_903886075.1
GCA_903886195.1
GCA_903886205.1
GCA_903886285.1
GCA_903886345.1
GCA_903886385.1
GCA_903886475.1
GCA_903886505.1
GCA_903886595.1
GCA_903886635.1
GCA_903886645.1
GCA_903886745.1
GCA_903890635.1
GCA_903890695.1
GCA_903890795.1
GCA_903890815.1
GCA_903890825.1
GCA_903890905.1
GCA_903890925.1
GCA_903890945.1
GCA_903891015.1
GCA_903891025.1
GCA_903891105.1
GCA_903891135.1
GCA_903891195.1
GCA_903891205.1
GCA_903891245.1
GCA_903891305.1
GCA_903932595.1
GCA_903932605.1
GCA_903960675.1
GCA_903969135.1
GCA_903978195.1
GCA_903978225.1
GCA_903987005.1
GCA_903987015.1
GCA_903989455.1
GCA_903989465.1
GCA_903989475.2

GCA_903989485.1
GCA_903992995.2
GCA_903993005.2
GCA_903993015.2
GCA_903993025.2
GCA_903993035.2
GCA_903993055.2
GCA_903993065.2
GCA_903993085.2
GCA_903993095.2
GCA_903993105.2
GCA_903993115.2
GCA_903993145.2
GCA_903993155.2
GCA_903993165.2
GCA_903993185.2
GCA_903993205.2
GCA_903993215.2
GCA_904066025.1
GCA_904066215.1
GCA_904129595.1
GCA_904425445.1
GCA_904425455.1
GCA_904425475.1
GCA_904426485.1
GCA_904440635.1
GCA_904440645.1
GCA_904528075.1
GCA_904711265.1
GCA_904711295.1
GCA_904711305.1
GCA_904799825.1
GCA_904810325.1
GCA_904810335.1
GCA_904810345.1
GCA_904810355.1
GCA_904810365.1
GCA_904830765.1
GCA_904830775.1
GCA_904830845.1
GCA_904830855.1
GCA_904830915.1
GCA_904830925.1
GCA_904830935.1
GCA_904830965.1
GCA_904830975.1
GCA_904831005.1
GCA_904831805.1
GCA_904831825.1
GCA_904831835.1
GCA_904831875.1
GCA_904833135.1
GCA_904842665.1
GCA_904848165.1

GCA_904848675.1
GCA_904859905.1
GCA_904863055.1
GCA_904863075.1
GCA_904863085.1
GCA_904863095.1
GCA_904863105.1
GCA_904863115.1
GCA_904863125.1
GCA_904863135.1
GCA_904863145.1
GCA_904863165.1
GCA_904863195.1
GCA_904863205.1
GCA_904863215.1
GCA_904863225.1
GCA_904863235.1
GCA_904863245.1
GCA_904863255.1
GCA_904863265.1
GCA_904863275.1
GCA_904863285.1
GCA_904863295.1
GCA_904863305.1
GCA_904863325.1
GCA_904863335.1
GCA_904863345.1
GCA_904863355.1
GCA_904863365.1
GCA_904863375.1
GCA_904863385.1
GCA_904863395.1
GCA_904863405.1
GCA_904863425.1
GCA_904863435.1
GCA_904863445.1
GCA_904864445.1
GCA_904864465.1
GCA_904864475.1
GCA_904864505.1
GCA_904864515.1
GCA_904864525.1
GCA_904864535.1
GCA_904864545.1
GCA_904864555.1
GCA_904864565.1
GCA_904864595.1
GCA_904864605.1
GCA_904864615.1
GCA_904864625.1
GCA_904864635.1
GCA_904864645.1
GCA_904865655.1
GCA_904865675.1

GCA_904865685.1
GCA_904865695.1
GCA_904865715.1
GCA_904865725.1
GCA_904865735.1
GCA_904865745.1
GCA_904865755.1
GCA_904865765.1
GCA_904865785.1
GCA_904865795.1
GCA_904865805.1
GCA_904865815.1
GCA_904865825.1
GCA_904865835.1
GCA_904865845.1
GCA_904866195.1
GCA_904866205.1
GCA_904866215.1
GCA_904866225.1
GCA_904866235.1
GCA_904866255.1
GCA_904866275.1
GCA_904866285.1
GCA_904866295.1
GCA_904866305.1
GCA_904866315.1
GCA_904866325.1
GCA_904866335.1
GCA_904866345.1
GCA_904866365.1
GCA_904866375.1
GCA_904866385.1
GCA_904866395.1
GCA_904866405.1
GCA_904866415.1
GCA_904866425.1
GCA_904866435.1
GCA_904866445.1
GCA_904866455.1
GCA_904866465.1
GCA_904866475.1
GCA_904866485.1
GCA_904866495.1
GCA_904866505.1
GCA_904866535.1
GCA_904866555.1
GCA_905071835.1
GCA_905071865.1
GCA_905071885.1
GCA_905143135.2
GCA_905175335.2
GCA_905175355.2
GCA_905175365.2
GCA_905175385.2

GCA_905186095.1
GCA_905186105.1
GCA_905186115.1
GCA_905186125.1
GCA_905186135.1
GCA_905186145.1
GCA_905186155.1
GCA_905186165.1
GCA_905187425.1
GCA_905188235.1
GCA_905218875.2
GCA_905218895.2
GCA_905219065.2
GCA_905219285.2
GCA_905219315.2
GCA_905219375.1
GCA_905219385.1
GCA_905220695.1
GCA_905220715.1
GCA_905220745.1
GCA_905220785.1
GCA_905220805.1
GCA_905231995.2
GCA_905232115.2
GCA_905232395.2
GCA_905232515.2
GCA_905232805.2
GCA_905310975.2
GCA_905310985.2
GCA_905310995.2
GCA_905311385.2
GCA_905311405.2
GCA_905311425.2
GCA_905311435.2
GCA_905312035.2
GCA_905315015.2
GCA_905315025.2
GCA_905315035.2
GCA_905315045.2
GCA_905315055.2
GCA_905315395.2
GCA_905315685.2
GCA_905315705.2
GCA_905315715.2
GCA_905318255.2
GCA_905319135.2
GCA_905319155.2
GCA_905319545.2
GCA_905319565.2
GCA_905322525.2
GCA_905322535.2
GCA_905329555.2
GCA_905329835.2
GCA_905329885.2

GCA_905330065.2
GCA_905330145.2
GCA_905330215.2
GCA_905330245.2
GCA_905330275.2
GCA_905330295.2
GCA_905330545.2
GCA_905330835.2
GCA_905330865.2
GCA_905330875.2
GCA_905330895.2
GCA_905330955.2
GCA_905331185.2
GCA_905331265.2
GCA_905331345.2
GCA_905331365.2
GCA_905332355.1
GCA_905332365.1
GCA_905332395.2
GCA_905333855.2
GCA_905333885.2
GCA_905335825.2
GCA_905336995.1
GCA_905337805.2
GCA_905337845.2
GCA_905338025.2
GCA_905338045.2
GCA_905367715.1
GCA_905367725.1
GCA_905367735.1
GCA_905367745.1
GCA_906464835.1
GCA_906464865.1
GCA_906464875.1
GCA_906464895.1
GCA_906464915.2
GCA_906464925.1
GCA_907163065.1
GCA_907163105.1
GCA_907163125.1
GCA_907163145.1
GCA_907163165.1
GCA_907163175.1
GCA_907163185.1
GCA_907163225.1
GCA_907163235.1
GCA_907163245.1
GCA_907163255.1
GCA_907163315.1
GCA_907164845.1
GCA_907164955.1
GCA_907165195.1
GCA_907165365.1
GCA_907176135.1

GCA_907176815.1
GCA_907177285.1
GCA_910574535.1
GCA_910589455.1
GCA_910589745.1
GCA_910591805.1
GCA_910593725.1
GCA_911728565.1
GCA_911810475.1
GCA_912579705.1
GCA_912579715.1
GCA_914590485.1
GCA_915401705.1
GCA_915401715.1
GCA_915401735.1
GCA_915401745.1
GCA_915401765.1
GCA_915402005.1
GCA_915402015.1
GCA_915403165.1
GCA_916098155.1
GCA_916098175.1
GCA_916098255.1
GCA_916098265.1
GCA_916618745.1
GCA_916618895.1
GCA_916618955.1
GCA_918258265.1
GCA_918320885.1
GCA_918342435.1
GCA_918378365.1
GCA_918593995.1
GCA_918697765.1
GCA_918731615.1
GCA_918797525.1
GCA_919162555.1
GCA_919654025.1
GCA_919946725.1
GCA_919949215.1
GCA_920103885.1
GCA_920939465.1
GCA_923868895.1
GCA_924899195.1
GCA_927312875.1
GCA_927797885.1
GCA_928371855.1
GCA_928375135.1
GCA_928375965.1
GCA_928721465.1
GCA_928721525.1
GCA_928721565.1
GCA_928721615.1
GCA_928721665.1
GCA_928721695.1

GCA_928721995.1
GCA_928722105.1
GCA_928722155.1
GCA_928722225.1
GCA_928852565.1
GCA_930986525.1
GCA_931972305.1
GCA_932126575.1
GCA_932527315.1
GCA_932527325.1
GCA_932527465.1
GCA_932530255.1
GCA_932530315.1
GCA_932530395.1
GCA_934924865.1
GCA_936269705.1
GCA_936270145.1
GCA_936981045.1
GCA_937000105.1
GCA_937000115.1
GCA_937425535.1
GCA_939576165.1
GCA_940588555.1
GCA_940596495.1
GCA_940599845.1
GCA_940618755.1
GCA_940620125.1
GCA_940631035.1
GCA_940670685.1
GCA_940677205.1
GCA_940796315.1
GCA_942548115.1
GCA_943181435.1
GCA_943184895.1
GCA_943733665.1
GCA_943846585.1
GCA_943846595.1
GCA_943846615.1
GCA_943846635.1
GCA_943846645.1
GCA_943846655.1
GCA_943846665.1
GCA_943846685.1
GCA_943846695.1
GCA_943846705.1
GCA_943846715.1
GCA_943846725.1
GCA_943846735.1
GCA_943846745.1
GCA_943846755.1
GCA_943846765.1
GCA_943846775.1
GCA_943846785.1
GCA_944407095.1

GCA_945605565.1
GCA_945836005.1
GCA_946888465.1
GCA_946893605.1
GCA_946900835.1
GCA_946902045.1
GCA_946902905.1
GCA_946903285.1
GCA_946966015.1
GCA_947034825.1
GCA_947054945.1
GCA_947054965.1
GCA_947055055.1
GCA_947055095.1
GCA_947055105.1
GCA_947055185.1
GCA_947055195.1
GCA_947055595.1
GCA_947055965.1
GCA_947090715.1
GCA_947090765.1
GCA_947090775.1
GCA_947090785.1
GCA_947090825.1
GCA_947096915.1
GCA_947097575.1
GCA_947098765.1
GCA_947098835.1
GCA_947098855.1
GCA_947099305.1
GCA_947163785.1
GCA_947164515.1
GCA_947241125.1
GCA_947250015.1
GCA_947250465.1
GCA_947250475.1
GCA_947250495.1
GCA_947250505.1
GCA_947250515.1
GCA_947250525.1
GCA_947250535.1
GCA_947250545.1
GCA_947250555.1
GCA_947250565.1
GCA_947250575.1
GCA_947250585.1
GCA_947250595.1
GCA_947250605.1
GCA_947250615.1
GCA_947250625.1
GCA_947250635.1
GCA_947250645.1
GCA_947250655.1
GCA_947250665.1

GCA_947250675.1
GCA_947250685.1
GCA_947250695.1
GCA_947250705.1
GCA_947250715.1
GCA_947250725.1
GCA_947250735.1
GCA_947250745.1
GCA_947250755.1
GCA_947250765.1
GCA_947250775.1
GCA_947250785.1
GCA_947250795.1
GCA_947250805.1
GCA_947250815.1
GCA_947251425.1
GCA_947251435.1
GCA_947251465.1
GCA_947251475.1
GCA_947251485.1
GCA_947251495.1
GCA_947251505.1
GCA_947251515.1
GCA_947251525.1
GCA_947251535.1
GCA_947251545.1
GCA_947251555.1
GCA_947251565.1
GCA_947251575.1
GCA_947251585.1
GCA_947251595.1
GCA_947251605.1
GCA_947251615.1
GCA_947251625.1
GCA_947251635.1
GCA_947251645.1
GCA_947251655.1
GCA_947251665.1
GCA_947251675.1
GCA_947251685.1
GCA_947251695.1
GCA_947251705.1
GCA_947251725.1
GCA_947251735.1
GCA_947251745.1
GCA_947251755.1
GCA_947251765.1
GCA_947251775.1
GCA_947251795.1
GCA_947251805.1
GCA_947251815.1
GCA_947251825.1
GCA_947251835.1
GCA_947251845.1

GCA_947251865.1
GCA_947251875.1
GCA_947251895.1
GCA_947251915.1
GCA_947251925.1
GCA_947251935.1
GCA_947251945.1
GCA_947251955.1
GCA_947251965.1
GCA_947251975.1
GCA_947311705.1
GCA_947313835.2
GCA_947331815.1
GCA_947383695.1
GCA_947533255.1
GCA_947538885.1
GCA_947539375.1
GCA_948151475.1
GCA_948331105.1
GCA_948472415.1
GCA_949127625.2
GCA_949152215.1
GCA_949281945.1
GCA_949282005.1
GCA_949282015.1
GCA_949282045.1
GCA_949282055.1
GCA_949282065.1
GCA_949297685.1
GCA_949299565.1
GCA_949361545.1
GCA_949769235.1
GCA_949769255.1
GCA_949769275.1
GCA_949769285.1
GCA_949774925.1
GCA_949787155.1
GCA_949787645.1
GCA_949787665.1
GCA_949787715.1
GCA_949788275.1
GCA_949788565.1
GCA_949788745.1
GCA_949788775.1
GCA_949788785.1
GCA_949788915.1
GCA_949788925.1
GCA_949788935.1
GCA_949788945.1
GCA_949789215.1
GCA_949789875.1
GCA_949789945.1
GCA_949790125.1
GCA_949790305.1

GCA_949790575.1
GCA_949790605.1
GCA_949790945.1
GCA_949791205.1
GCA_949792585.1
GCA_949793775.1
GCA_949794035.1
GCA_949794475.1
GCA_949794895.1
GCA_951229915.1
GCA_951392265.1
GCA_951691365.2
GCA_951802375.2
GCA_951802855.1
GCA_951802865.1
GCA_951803545.1
GCA_951805275.2
GCA_951812285.1
GCA_951812325.1
GCA_951812335.1
GCA_951812345.1
GCA_951812525.1
GCA_951812545.1
GCA_951812575.1
GCA_951812585.1
GCA_951812715.1
GCA_951812725.1
GCA_951812735.1
GCA_951812745.1
GCA_951812755.1
GCA_951812775.1
GCA_951812785.1
GCA_951812795.1
GCA_951812825.1
GCA_951812895.1
GCA_951812955.1
GCA_951812965.1
GCA_951813075.1
GCA_951813085.1
GCA_951813255.1
GCA_951813285.1
GCA_951813325.1
GCA_951813395.1
GCA_951813515.1
GCA_951813535.1
GCA_951813555.1
GCA_951813565.1
GCA_951813575.1
GCA_951813605.1
GCA_951813615.1
GCA_951813825.1
GCA_951813855.1
GCA_951828235.1
GCA_951828385.1

GCA_951828515.1
GCA_951828535.1
GCA_951828585.1
GCA_958267355.1
GCA_958295465.1
GCA_958295495.1
GCA_958295525.1
GCA_958295645.1
GCA_958295675.1
GCA_958295695.1
GCA_958295715.1
GCA_958295755.1
GCA_958295785.1
GCA_958295805.1
GCA_958295815.1
GCA_958295825.1
GCA_958295835.1
GCA_958295845.1
GCA_958295875.1
GCA_958295885.1
GCA_958295915.1
GCA_958295935.1
GCA_958295945.1
GCA_958295975.1
GCA_958296025.1
GCA_958296035.1
GCA_958296085.1
GCA_958296095.1
GCA_958296105.1
GCA_958296115.1
GCA_958296125.1
GCA_958296165.1
GCA_958296175.1
GCA_958296195.1
GCA_958296225.1
GCA_958296235.1
GCA_958296245.1
GCA_958296285.1
GCA_958296295.1
GCA_958296385.1
GCA_958296405.1
GCA_958296445.1
100 prokaryotic GCA of Fig 3A
GCA_000011265.1
GCA_000011325.1
GCA_000017305.1
GCA_000022245.1
GCA_000024665.1
GCA_000027145.1
GCA_000166695.1
GCA_000412755.1
GCA_000812165.1
GCA_001007915.1
GCA_001420975.1

GCA_001442475.1
GCA_001513695.1
GCA_001900945.1
GCA_001953695.1
GCA_002113865.1
GCA_002117325.1
GCA_002216815.1
GCA_002812505.1
GCA_002891665.1
GCA_003018795.1
GCA_003234175.2
GCA_003288175.1
GCA_003515225.1
GCA_003515305.1
GCA_003665275.1
GCA_003667765.1
GCA_003966935.1
GCA_004295565.1
GCA_004324115.2
GCA_006742345.1
GCA_008330485.1
GCA_008807915.1
GCA_009648815.1
GCA_009938265.1
GCA_011995885.1
GCA_013166995.1
GCA_013168075.1
GCA_013187685.1
GCA_013201035.1
GCA_013343375.1
GCA_013357405.1
GCA_013368015.1
GCA_013624605.1
GCA_014279655.1
GCA_014622965.1
GCA_014840915.2
GCA_015136315.1
GCA_015654285.1
GCA_016749085.1
GCA_016839145.1
GCA_017161585.1
GCA_017357225.1
GCA_017743035.1
GCA_018135585.1
GCA_018326605.1
GCA_018736765.1
GCA_018846995.1
GCA_019703615.1
GCA_020181355.1
GCA_020541325.1
GCA_021183785.1
GCA_021378685.1
GCA_021484765.1
GCA_022220945.1

GCA_022221445.1
GCA_022405295.1
GCA_022407255.1
GCA_022453825.1
GCA_022460295.1
GCA_022919915.1
GCA_022921935.1
GCA_022922035.1
GCA_023101665.1
GCA_023559145.1
GCA_023639785.1
GCA_023734255.1
GCA_024172665.1
GCA_024662035.1
GCA_024749145.1
GCA_024917915.1
GCA_025142595.1
GCA_025643035.1
GCA_026801935.1
GCA_027171405.1
GCA_027571305.1
GCA_028751175.1
GCA_029395475.1
GCA_029537215.1
GCA_029714185.1
GCA_029873215.1
GCA_029873235.1
GCA_029910115.1
GCA_030007315.1
GCA_030035425.1
GCA_030253625.1
GCA_900474965.1
GCA_900475465.1
GCA_900475495.1
GCA_947054945.1
100 prokaryotic GCA of Fig S2
GCA_000008445.1
GCA_000017545.1
GCA_000176915.2
GCA_000183425.1
GCA_000227665.3
GCA_000255235.1
GCA_000262305.1
GCA_000331165.2
GCA_000363905.1
GCA_000698715.2
GCA_000737555.1
GCA_000739375.1
GCA_001029795.1
GCA_001148125.2
GCA_001456315.1
GCA_001465815.1
GCA_001466725.1
GCA_001513655.1

GCA_001605055.1
GCA_001611135.1
GCA_001653435.1
GCA_001697325.1
GCA_001900435.1
GCA_002157265.1
GCA_002165305.2
GCA_002205755.1
GCA_002209305.1
GCA_002220095.1
GCA_002240075.1
GCA_002356255.1
GCA_002591115.1
GCA_002741685.1
GCA_002943855.1
GCA_003254375.1
GCA_003359375.2
GCA_003626995.1
GCA_003722215.1
GCA_003798165.1
GCA_003956225.1
GCA_008329865.1
GCA_008370735.1
GCA_009498375.1
GCA_009625895.1
GCA_009626815.1
GCA_009626955.1
GCA_009650355.1
GCA_009664715.1
GCA_009749345.1
GCA_009763545.1
GCA_009796825.1
GCA_009832805.1
GCA_010202845.1
GCA_011044335.1
GCA_013167175.1
GCA_013267835.1
GCA_015074905.1
GCA_016127955.1
GCA_016700075.1
GCA_017161785.1
GCA_018219435.1
GCA_018986875.1
GCA_019285735.1
GCA_019443265.1
GCA_020510245.1
GCA_021459965.1
GCA_021800405.1
GCA_022221105.1
GCA_022493435.1
GCA_022494095.1
GCA_022923275.1
GCA_023169785.1
GCA_023212325.2

GCA_023361195.1
GCA_024453815.1
GCA_025140155.1
GCA_025150565.1
GCA_025502545.1
GCA_025757685.1
GCA_026547075.1
GCA_027625395.1
GCA_027942055.1
GCA_028221005.1
GCA_028541005.1
GCA_028622915.1
GCA_028870515.1
GCA_029280505.1
GCA_029634765.1
GCA_029962565.1
GCA_030122685.1
GCA_030166515.1
GCA_030370115.1
GCA_900016785.1
GCA_900474755.1
GCA_900520375.1
GCA_900636545.1
GCA_900636735.1
GCA_902703215.1
GCA_902810325.1
GCA_904863055.1
GCA_947251915.1
100 prokaryotic GCA of Fig S3
GCA_000009725.1
GCA_000227685.3
GCA_000242895.3
GCA_000287295.1
GCA_000452385.2
GCA_000569035.1
GCA_000695095.2
GCA_000783995.2
GCA_001263395.1
GCA_001444325.1
GCA_001457615.1
GCA_001465595.2
GCA_001986915.1
GCA_002078335.1
GCA_002082215.1
GCA_002085735.1
GCA_002111085.1
GCA_002197125.1
GCA_002211505.1
GCA_002211685.1
GCA_002213825.1
GCA_002310295.2
GCA_003063885.1
GCA_003064365.2
GCA_003261575.2

GCA_003344465.1
GCA_003359435.2
GCA_003481905.1
GCA_003856655.1
GCA_004008995.1
GCA_004114735.1
GCA_004135795.1
GCA_004299805.1
GCA_005937885.2
GCA_006770445.1
GCA_006874645.1
GCA_007954445.1
GCA_009734125.1
GCA_009857035.1
GCA_011996525.1
GCA_013267695.1
GCA_013284785.2
GCA_013391145.1
GCA_013393785.1
GCA_013488205.1
GCA_013731735.1
GCA_013745115.1
GCA_014170855.1
GCA_016415465.1
GCA_016454645.1
GCA_016889365.1
GCA_017311125.1
GCA_018075345.1
GCA_018085225.1
GCA_019047885.1
GCA_019048965.1
GCA_019134755.1
GCA_019329805.1
GCA_019665805.1
GCA_019976975.1
GCA_020002285.1
GCA_020497585.1
GCA_020827635.1
GCA_021952745.1
GCA_022370395.1
GCA_022828575.1
GCA_022846495.1
GCA_022921295.1
GCA_023361995.1
GCA_023639785.1
GCA_023650735.1
GCA_024582695.1
GCA_024741555.1
GCA_024760585.1
GCA_024972275.1
GCA_025345525.1
GCA_025398935.1
GCA_025403485.1
GCA_025560165.1

GCA_025857195.1
GCA_026057535.1
GCA_026547095.1
GCA_027557675.1
GCA_028768545.1
GCA_028892955.1
GCA_029016665.1
GCA_029719865.1
GCA_030013125.1
GCA_030020825.1
GCA_030059845.1
GCA_030123065.1
GCA_030285805.1
GCA_030369815.1
GCA_030406775.1
GCA_900095885.1
GCA_900607255.1
GCA_900622665.1
GCA_900660705.1
GCA_902381775.1
GCA_951812965.1
